# Enzymatic combinatorial synthesis of E-64 and related cysteine protease inhibitors

**DOI:** 10.1038/s41589-025-01907-2

**Published:** 2025-05-09

**Authors:** Mengting Liu, Xin Zang, Niko W. Vlahakis, Jose A. Rodriguez, Masao Ohashi, Yi Tang

**Affiliations:** 1 Department of Chemical and Biomolecular Engineering, University of California, Los Angeles, Los Angeles, CA USA; 2 Department of Chemistry and Biochemistry, UCLA-DOE Institute for Genomics and Proteomics, STROBE, NSF Science and Technology Center, University of California, Los Angeles, Los Angeles, CA USA; 3 Department of Chemistry and Biochemistry, University of California, Los Angeles, Los Angeles, CA USA

**Keywords:** Biosynthesis, Biocatalysis, Natural products, Enzymes

## Abstract

E-64 is an irreversible cysteine protease inhibitor prominently used in chemical biology and drug discovery. Here we uncover a nonribosomal peptide synthetase-independent biosynthetic pathway for E-64, which is widely conserved in fungi. The pathway starts with epoxidation of fumaric acid to the warhead (2*S*,3*S*)-*trans*-epoxysuccinic acid with an Fe(II)/α-ketoglutarate-dependent oxygenase, followed by successive condensation with an l-amino acid by an adenosine triphosphate grasp enzyme and with an amine by the fungal example of amide bond synthetase. Both amide bond-forming enzymes display notable biocatalytic potential, including scalability, stereoselectivity toward the warhead and broader substrate scopes in forming the amide bonds. Biocatalytic cascade with these amide bond-forming enzymes generated a library of cysteine protease inhibitors, leading to more potent cathepsin inhibitors. Additionally, one-pot reactions enabled the preparative synthesis of clinically relevant inhibitors. Our work highlights the importance of biosynthetic investigation for enzyme discovery and the potential of amide bond-forming enzymes in synthesizing small-molecule libraries.

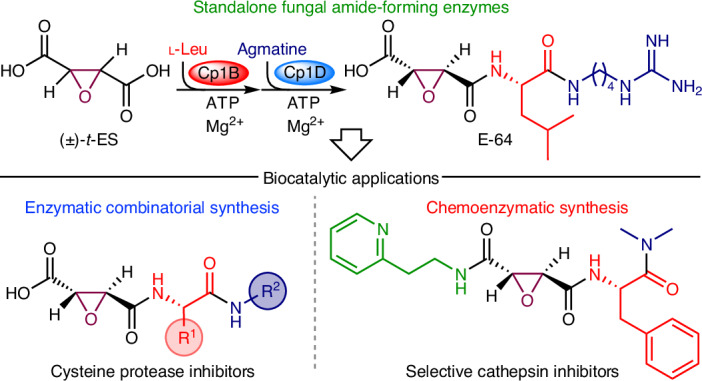

## Abstract (web summary)

Liu et al. discover amide-forming enzymes in the biosynthesis of E-64, a classic cysteine protease inhibitor. Enzymatic combinatorial biocatalysis creates a large compound library and leads to the identification of useful cathepsin inhibitors.

## Main

**Fig. 1:**
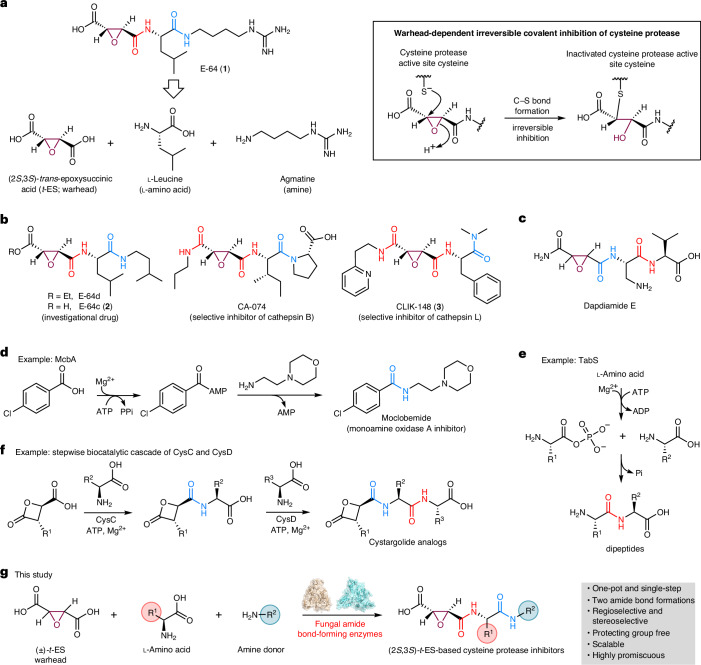
Amide bond-forming enzyme(s) are hypothesized to be involved in the biosynthesis of E-64. **a**, Structure, mode of action and retrobiosynthesis of **1**. E-64 contains the *t*-ES warhead that is the site of covalent inhibition of cysteine proteases. E-64 is proposed to form from the condensation of (2*S*,3*S*)-*t-*ES, l-Leu and agmatine. **b**, Structures of synthetic cysteine protease inhibitors based on **1**. **c**, Structure of dapdiamide E. **d**, Mechanism and application of bacterial ABSs such as McbA from the marinacarboline biosynthetic pathway. **e**, Mechanism and applications of bacterial ATP-grasp enzymes such as TabS from the tabtoxin biosynthetic pathway. **f**, Stepwise combination of two bacterial amide bond-forming enzymes CysC and CysD to synthesize cystargolide analogs. **g**, In this work, we discovered and biochemically characterized the fungal ATP-grasp enzyme and ABS from the biosynthesis of **1** and used the enzymes in the synthesis of diverse E-64 analogs.

E-64 (**1**) is a fungal natural product that has prominent roles in drug discovery and chemical biology^1–13^ (Fig. 1a). Isolated from *Aspergillus japonicus* TPR-64 in 1978, **1** is a classic *trans*-epoxysuccinic acid (*t*-ES)-based irreversible, potent and selective inhibitor against cysteine proteases such as papain, calpain and cysteine cathepsins^5,14,15^ (Fig. 1a). Cysteine proteases are ubiquitously conserved in all kingdoms of life, have multifaceted physiological roles and are potential drug targets for multiple diseases^2,3,11–13,16–19^. Upon binding to a cysteine protease, the electrophilic *t*-ES warhead in **1** is covalently captured by the thiolate of the catalytic cysteine^15,20^ (Fig. 1a), a feature that led to the development of probes based on **1** for activity-based protein profiling (ABPP)^6–8,10^. Numerous biosynthetic variants of **1** have been isolated from fungi^6,21–24^ (Supplementary Fig. 1). Extensive synthetic efforts have resulted in a variety of analogs such as CA-074 (ref. ^25^), CLIK-148 (**3**)^26^ and NYC-488 (ref. ^27^) that show selectivity toward cathepsin B, cathepsin L and calpain, respectively (Fig. 1b and Supplementary Fig. 1). E-64d (loxistatin), a prodrug for E-64c (**2**; loxistatin acid), was in phase 3 trials for the treatment of muscular dystrophy^2^ (Fig. 1b) and was also repurposed for treating viral infections such as coronavirus disease^1,28^. Despite such a decorated track record of **1**, the enzymes responsible for the formation of **1** have remained elusive.

**Extended Data Fig. 1:**
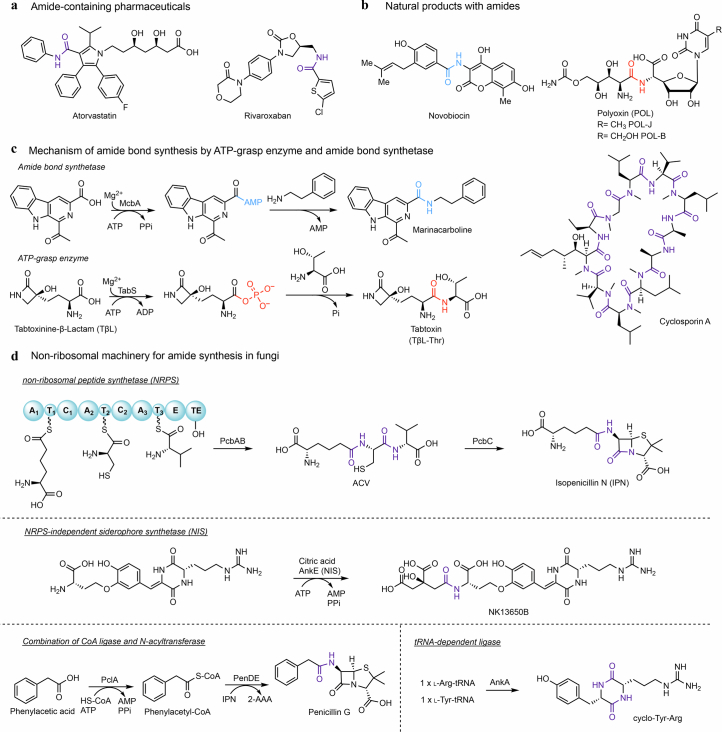
Significance and biosynthetic machinery of amide functionality. **a**, Selected top-selling drugs containing amide bond. **b**, Representative amide-containing natural products used in clinic and agriculture. **c**, The mechanisms of the biocatalytically competent amide bond synthetase McbA and the ATP-grasp enzyme TabS. **d**, Examples of non-ribosomal strateiges for amide synthesis in fungi. The amide bond formations in isopenicillin N (IPN) is catalyzed by three modules NRPS PcbAB. NRPS-independent siderophore synthetase (NIS) AnkE is proposed to catalyze the amide bond in NK13650B. The pair of CoA ligase PclA and *N*-acyltransferase PenDE forms the amide bond in the production of penicillin G and 2-aminoadipic acid (2-AAA). AnkA catalyzed the tRNA-dependent amidation to form cyclo-Tyr-Arg. Domain abbreviations: A: adenylation; T: peptidyl-carrier protein; C: condensation; E: epimerization; TE: thioesterase. While a few ATP-grasp enzymes were proposed to involve the biosynthesis of the fungal peptides (also see Extended Data Fig. 2), ABSs have not been identified in the fungal natural product biosynthesis.

**Extended Data Fig. 2:**
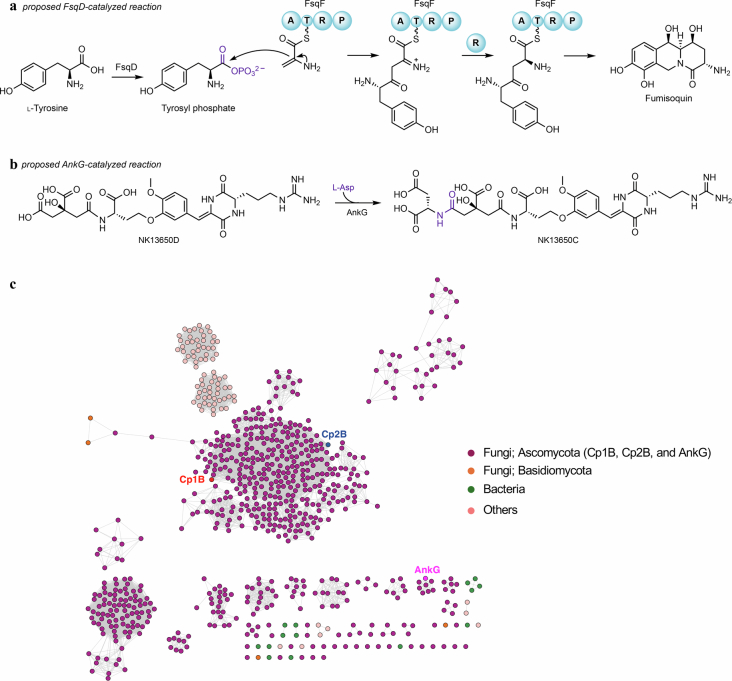
Limited examples of fungal ATP-grasp enzyme involved in natural product biosynthesis. **a**, FsqD from fumisoquin biosynthesis was proposed to activate L-tyrosine to form tyrosyl phosphate. The function of FsqD has not been biochemically characterized. **b**, AnkG was proposed to be an ATP-grasp enzyme that catalyzes the amide bond formation between L-aspartic acid and NK13650D to form NK13650C based on the in vivo experiments. **c**, Sequence similarity network analysis of Cp1B homologs from the UniProt database. Note that 5,000 maximum number target sequences from a blastp search with Cp1B as a query (expect threshold value: 5) were retrieved and subjected to SSN construction, with an alignment score threshold of 7. Cp1B and Cp2B in this study and the proposed ATP-grasp enzyme AnkG are highlighted. While Cp1B and Cp2B were located at a separate clade from AnkG, the SSN and the amino acid sequence identity between them ( ~ 30%) suggest these enzymes are distinct but distantly related. While no characterized enzymes were found in this SSN, the SSN showed that putative Cp1B-like ATP-grasp enzymes are conserved in not only many fungi (Ascomycota and Basidiomycota) but also in a few bacteria. Domain abbreviations: A: adenylation; T: peptidyl-carrier protein; R: reductase domain; P: pyridoxal phosphate binding domain.

Biosynthetically, **1** is formed from the stepwise condensation of three distinct building blocks, the warhead (2*S*,3*S*)-*t*-ES (a dicarboxylic acid), l-Leu (an amino acid) and agmatine (an amine) (Fig. 1a,b), using amide bond-forming enzyme(s) (Fig. 1a). Amide bond-forming enzymes have found widespread interest as biocatalysts for the synthesis of pharmaceuticals because of the ubiquity of the amide functionality^29,30^ (Extended Data Fig. 1a,b). Enzymatic synthesis of amides can obviate the protection–deprotection steps associated with functionally rich building blocks and can achieve chemoselectivity and regioselectivity with atom economy. Two particular classes of enzymes that are not associated with nonribosomal peptide synthetases (NRPSs) (Extended Data Fig. 1c,d and Supplementary Fig. 2), adenosine triphosphate (ATP) grasp enzymes and amide bond synthetases (ABSs), have attracted attention because of their direct activation and amidation of carboxylic acids without the need for partnering enzymes^30–35^. ATP-grasp enzymes phosphorylate the carboxylic acid partner followed by condensation with the amine donor, while ABSs adenylate the carboxylic acid followed by amidation with amine nucleophiles (Fig. 1d,e and Extended Data Fig. 1c). Recently discovered bacterial enzymes such as the ATP-grasp enzyme TabS that forms dipeptides^35^ and ABSs McbA^32,36^ and CfaL^34^ have been demonstrated to be synthetically useful (Fig. 1d,e and Extended Data Fig. 1c). Micklefield and coworkers demonstrated that two amide bond-forming enzymes CysC and CysD from the cystargolide biosynthetic pathway can be combined stepwise to synthesize diverse analogs at scale^37^ (Fig. 1f). In sharp contrast, only a few putative ATP-grasp enzymes have been reported^38,39^ and no ABSs have been identified from fungi (Extended Data Fig. 2a,b).

Notable bacterial natural products with partial structural resemblance to **1** are pseudotripeptide dapdiamides^40,41^ (Fig. 1c and Supplementary Fig. 3). Work by Walsh and Clardy showed that the two amide bonds are constructed by an NRPS-independent pathway including an ATP-grasp enzyme DdaF and an ABS DdaG (Supplementary Fig. 3). Inspired by such chemical logic, we hypothesize that the biosynthesis of the tripartite structure of **1** might involve standalone amide bond-forming enzymes from fungi. Here, we describe the discovery, characterization and application of an ATP-grasp enzyme and ABS from the E-64 biosynthetic pathway (Fig. 1g). Our work underscores the importance of biosynthetic studies in discovering powerful biocatalysts and paves the way for enzymatic combinatorial synthesis using amide bond-forming enzymes for the generation of large bioactive small-molecule libraries.

## Results

### The BGCs of E-64 and E-64 analogs

Directly searching for the biosynthetic gene clusters (BGCs) of E-64 using ATP-grasp enzymes and ABSs is challenging because of the lack of characterized examples in fungi. The polyamine fragments in natural E-64 analogs (Supplementary Fig. 1), which include putrescine, agmatine and cadaverine, are formed from the decarboxylation of l-Orn, l-Arg and l-Lys, respectively, by pyridoxal phosphate (PLP)-dependent decarboxylases^42^. Although those polyamines are known primary metabolites in fungi, we reasoned that the BGCs for **1** and related compounds might encode a dedicated PLP-dependent lysine or ornithine decarboxylase to increase supplies of polyamines as building blocks.

**Fig. 2:**
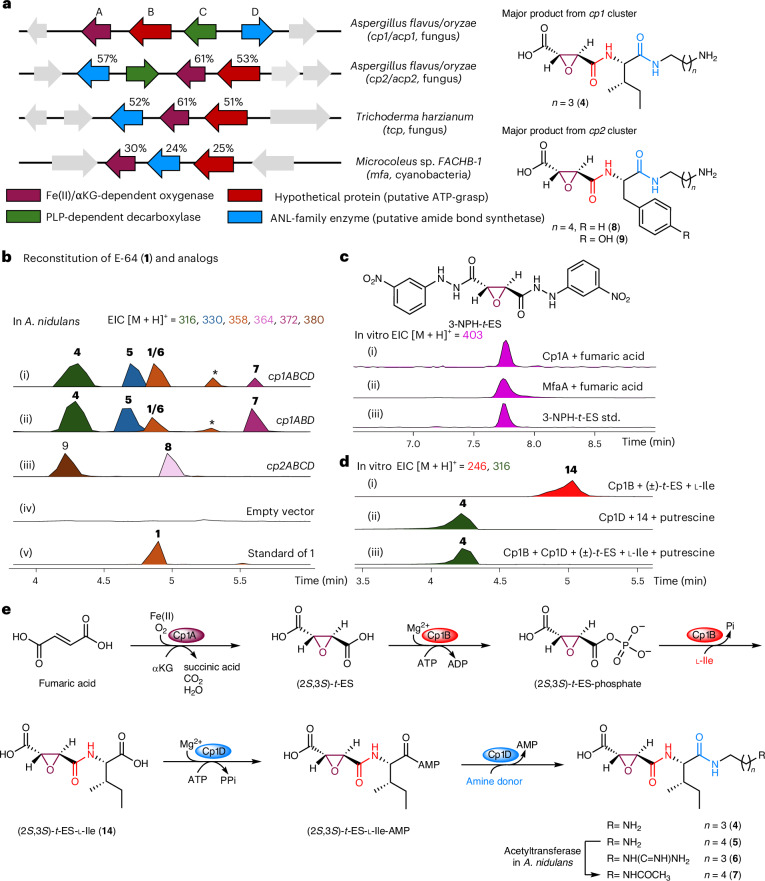
Biosynthetic pathway for E-64 and identifying standalone amide-forming enzymes. **a**, BGCs of **1** from *A.* *flavus* and *A.* *oryzae* and other homologous BGCs. The percentage amino acid sequence identity to each corresponding Cp1 enzyme is shown. **b**, LC–QTOF analysis of metabolites produced by different gene combinations from *cp1* and *cp2* clusters in the heterologous host *A.* *nidulans* is shown in (i)–(v). Selected ion chromatography traces presented on the same scale are shown and the colors of the traces match the indicated mass and compounds. The *y* axis represents ion counts. *Not isolated. **c**, Cp1A catalyzed the epoxidation on fumaric acid to *t*-ES. Assays were carried out at 30 °C for 3 h in 100 μl of 50 mM sodium phosphate buffer with 0.2 mM FeSO_4_, 2 mM αKG, 2 mM ascorbate, 1 mM fumaric acid and 10 μM Cp1A or MfaA. The products were derivatized with 3-NPH to increase MS sensitivity. Selected ion monitoring of 3-NPH-*t*-ES ([M + H]^+^ = 403) is shown. The *y* axis represents ion counts and the chromatograms are presented on the same scale. **d**, Enzyme assays with Cp1B and Cp1D. Reactions were performed at 30 °C for 16 h in 100 μl of 50 mM sodium phosphate buffer (pH 8.0). Reaction components for each reaction were as follows: (i) 25 μM Cp1B, 5 mM (±)-*t*-ES, 2.5 mM l-Ile, 10 mM ATP and 10 mM MgCl_2_; (ii) 10 μM Cp1D, 2 mM **14**, 2.5 mM putrescine, 10 mM ATP and 10 mM MgCl_2_; (iii) 25 μM Cp1B, 25 μM Cp1D, 5 mM (±)-*t*-ES, 2.5 mM l-Ile, 5 mM putrescine, 10 mM ATP and 10 mM MgCl_2_. Traces represent selected ion monitoring of **4** ([M + H]^+^ = 316) and **14** ([M + H]^+^ = 246). The *y* axis represents ion counts and the chromatograms are presented on the same scale. **e**, Biosynthetic pathway of **1** and the related compounds from the *cp1* pathway.

Using *N*-dimethyllysine decarboxylase FlvG^43^ as a beacon, we searched the genomes of fungal producers of **1** and analogs. Two putative BGCs (*acp1* and *acp2*) were identified from *A.* *oryzae* (Fig. 2a), which are also present in the closely related fungus *A.* *flavus* (*cp1* and *cp2*) (Fig. 2a). Each BGC encodes four proteins, including an Fe(II)/α-ketoglutarate (αKG)-dependent oxygenase (*cp1A*/*cp2A*), a hypothetical protein (HP) (*cp1B*/*cp2B*) with no detectable Pfam domain, a PLP-dependent decarboxylase (*cp1C*/*cp2C*) and a protein (*cp1D*/*cp2D*) that is predicted to belong to the adenylate-forming (ANL) family^44^, of which ABSs are also members (Supplementary Fig. 4). Each protein in the *cp1* BGC shares ~50% amino acid sequence identity to the corresponding homolog in the *cp2* BGC (Fig. 2a and Supplementary Table 1). A sequence similarity network (SSN)^45^ analysis of Cp1B and Cp2B revealed that there are many related uncharacterized proteins in Ascomycota (Extended Data Fig. 2b,c). Structural analysis of Cp1B and Cp2B by Foldseek^46^ identified closest structure homologs including homoglutathione (hGSH) synthetase, an ATP-grasp enzyme that catalyzes the condensation of γ-glutamylcysteine and β-alanine to form tripeptide hGSH^47^, and the recently characterized bacterial peptidylpolyamine synthetases YgiC and YjfC^48^ (Supplementary Fig. 5).

Heterologous expression of *cp1A–cp1D* genes in *A.* *nidulans* ΔEMΔST^49^ (Supplementary Fig. 6 and Supplementary Tables 2 and 3) led to the production of multiple hydrophilic metabolites with molecular weights (MWs) of 315 (**4**), 329 (**5**), 357 (**1** and **6**) and 371 (**7**) (Fig. 2b). Isolation and nuclear magnetic resonance (NMR) analysis showed that **4**, **5** and **6** are analogs of **1** containing a *t*-ES*-*l-Ile condensed with putrescine, cadaverine and agmatine, respectively^21^ (Supplementary Tables 6 and 8–10 and Supplementary Figs. 35–39 and 47–61). Compound **6** is the l-Ile variant of **1** and coelutes with **1** during analysis. NMR analysis showed that **7** is a derivative of **5** that has been *N*-acetylated (Supplementary Table 11 and Supplementary Figs. 62–66), a reaction that is likely catalyzed by an endogenous acetyltransferase. The absolute stereochemistry of *t*-ES moiety in those compounds was deduced to be (2*S*,3*S*) on the basis of comparisons of NMR spectra and optical rotation to reported values^21^. In contrast, heterologous expression of the *cp2* BGC in *A.* *nidulans* afforded **8** and **9**, which were characterized to be *t*-ES-l-Phe-cadaverine and *t*-ES-l-Tyr-cadaverine, respectively (Fig. 2a,b, Supplementary Tables 12 and 13 and Supplementary Figs. 67–76). Two minor products **10** and **11** were also detected from this strain (Extended Data Fig. 4), of which **10** was characterized to be *N-*acetyl-**8** (Supplementary Table 14 and Supplementary Figs. 77–81) and **11** is proposed to be *N-*acetyl-**9** on the basis of MW.

**Extended Data Fig. 3:**
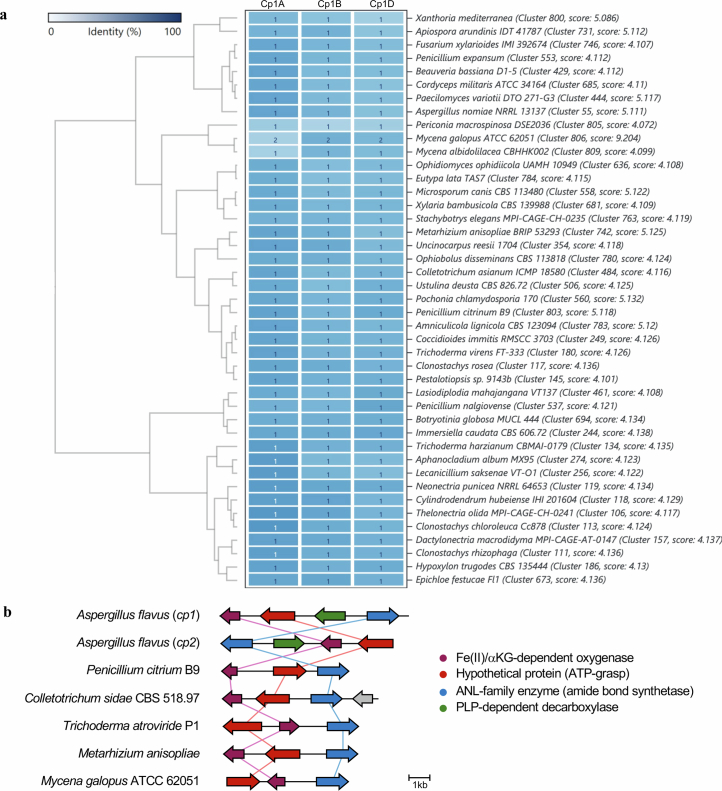
E-64-like biosynthetic gene clusters are widely conserved in fungi. **a**, Selected clusters are shown in a dendrogram (based on identity to query sequences) from cblaster search. A darker tint of blue indicates a higher percentage identity of the query in the output cluster. The three gene cassette (*cpA*, *cpB*, and *cpD*) is highly conserved in more than > 40 different fungal genera such as *Aspergillus spp*, *Penicillium spp*, *Metarhizium spp*, *Trichoderma spp*, and *Mycena* spp (Basidiomycota). Two copies of E-64 like cluster are also present in *Mycena galopus*. **b**. Selected E-64 homologous biosynthetic gene clusters in clinker visualization, including reported E-64 analog producing fungi (*Aspergillus oryzae*, *Penicillium citrinum*, and *Colletotrichium spp*) and fungi not known to produce E-64 (*Trichoderma atroviride*, *Metarhizium anisopliae*, and *Mycena galopus* ATCC 62051). Nearly all the genes in those clusters are conserved except for PLP-dependent decarboxylase (the homolog of Cp1C).

**Extended Data Fig. 4:**
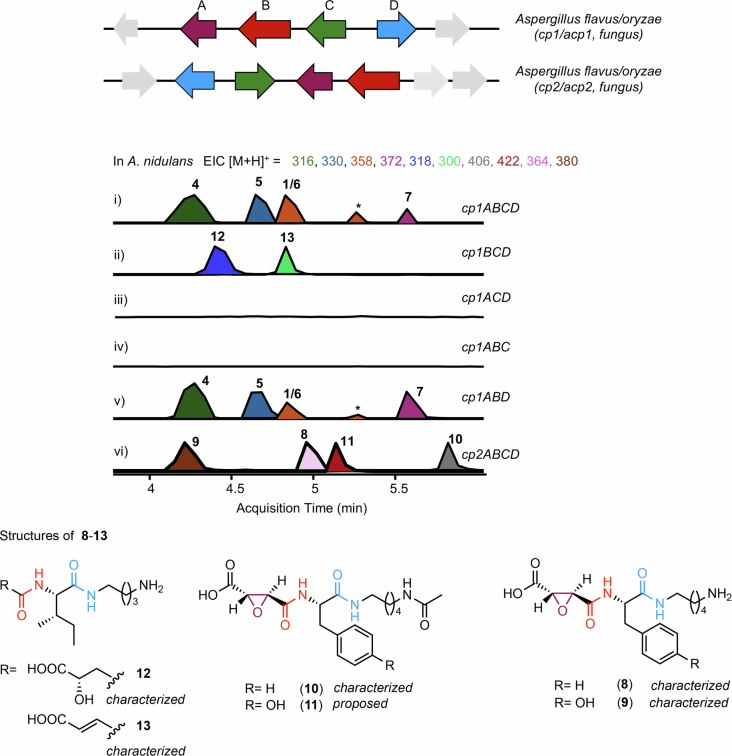
LC/MS analysis of extracts from the heterologous expression of *cp1* and *cp2* in *A. nidulans.* LC/MS analyses include *cp1ABCD* (i), *cp1BCD* (ii), *cp1ACD* (iii), *cp1ABC* (iv), *cp1ABD* (v), and *cp2ABCD* (vi). Selected ion chromatograms correspond to the [M + H]^+^ for **1** ([M + H]^+^ = 358), **4** ([M + H]^+^ = 316), **5** ([M + H]^+^ = 330), **6** ([M + H]^+^ = 358), **7** ([M + H]^+^ = 372), **8** ([M + H]^+^ = 364), **9** ([M + H]^+^ = 380), **10** ([M + H]^+^ = 406), **11** ([M + H]^+^ = 422), **12** ([M + H]^+^ = 318), and **13** ([M + H]^+^ = 300). Y-axis represents ion counts and the chromatograms are presented on the same scale. Heterologous expression of three gene cassette *cp1ABD* is sufficient for the biosynthesis of **1** and the analogs in *A. nidulans*. As polyamines are abundant primary metabolites in fungi, the PLP-dependent decarboxylase Cp1C is not essential for the biosynthesis of **1** and the analogs in heterologous host *A. nidulans*. Interestingly, the heterologous expression of *cp1BCD* led to the formation of malic acid (**12**) and fumaric acid (**13**) derivatives, suggesting the role of Cp1A as an epoxidase. This result further supported the promiscuous substrate specificities of both Cp1B and Cp1D. The structures of all compounds except **11** were determined by NMR.

These results confirmed that both *cp1* and *cp2* BGCs are responsible for the biosynthesis of **1** and analogs. Removing the PLP-dependent decarboxylase *cp1C* from each *A.* *nidulans* transformant did not affect the metabolic profiles (Fig. 2b and Extended Data Fig. 4). Omitting any of *cp1A*, *cp1B* or *cp1D* from the transformant abolished the production of *t*-ES-containing compounds related to **1** (Extended Data Fig. 4), thereby establishing that these three genes constitute a minimum biosynthetic cassette. Indeed, such three-gene BGCs can be found in >100 fungal species with >40 different genera, as well as in cyanobacteria such as *Microcoleus* spp., nearly all of which are not known to produce related compounds (Fig. 2a, Extended Data Fig. 3a,b and Supplementary Fig. 1). The transformant lacking *cp1A* produced the malic acid and fumaric acid analogs of **4**, which were characterized to be **12** and **13**, respectively (Extended Data Fig. 4, Supplementary Tables 15 and 16 and Supplementary Figs. 82–91), in agreement with the proposed role of Cp1A as the epoxidase.

### Cp1A is a (2*S*,3*S*)-*t*-ES synthase

**Extended Data Fig. 5:**
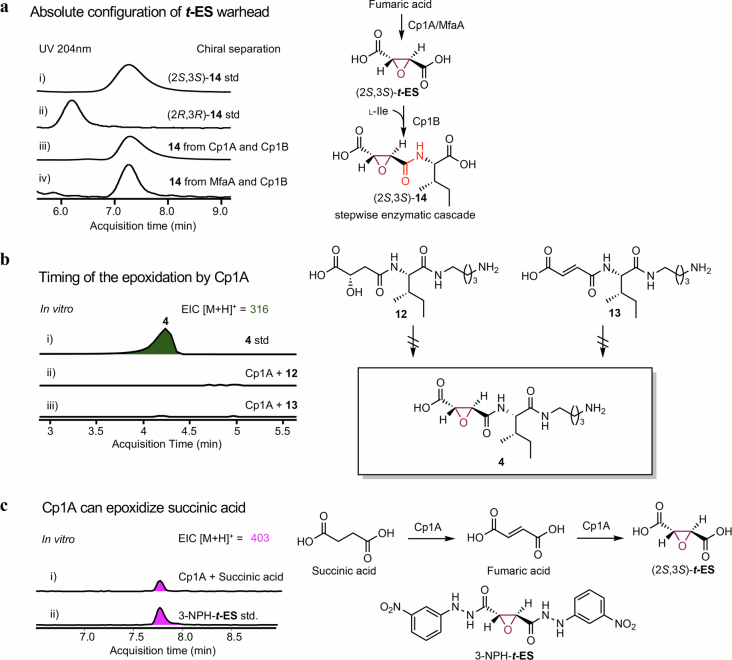
Absolute configuration of *t*-ES and substrate for Cp1A. **a**, Enzymatic synthesis of (2*S*,3*S*)-*t*-ES from Cp1A or MfaA. Briefly, the reaction was performed in 50 mM sodium phosphate buffer (pH 8.0) containing 0.2 mM FeSO_4_, 2 mM αKG, 2 mM ascorbate, 1 mM of substrate, and 10 μM of Cp1A or MfaA at 30 °C for 16 h. The protein was removed by Amicon concentrators (Millipore). Subsequently 10 μM Cp1B, 2.5 mM l-isoleucine, 10 mM ATP, 10 mM MgCl_2_ were added followed by incubation at 30 °C for 16 h. The enzymatic synthesis of (2*S*,3*S*)-**14** allowed determination of the absolute configuration of the epoxide to be (2*S*, 3*S*), based on retention times of standards. HPLC analysis was performed with a CHIRALPAK^®^ IA-3 column (150 ×4.6 mm, 3 μm) at room temperature (flow rate 1 mL/min, 40% MeCN–H_2_O with 0.1% trifluoroacetic acid). Y-axis represents UV absorption (λ=204 nm) and the chromatograms are not presented on the same scale. **b**, LC/MS analysis of reaction of Cp1A with **12** or **13**. 100 μL reactions were performed at 30 °C for 3 h, in 50 mM sodium phosphate buffer (pH 8.0) containing 0.2 mM FeSO_4_, 2 mM αKG, 2 mM ascorbate, 1 mM of substrate **12** or **13**, and 10 μM of Cp1A. **4** was not observed in enzymatic assay of Cp1A with **12** (ii) and **13** (iii) in the presence of αKG, ascorbate, and Fe^2+^. The traces show selected ion monitoring of **4** ([M + H]^+^ = 316). Y-axis represents ion counts and the chromatograms are presented on the same scale. **c**, Enzymatic reaction of Cp1A with succinic acid. The same reaction condition with Extended Data Fig. 5b was used except for succinic acid being used as the substrate. After overnight incubation at 30 °C, the product was derivatized with 3-NPH. Selected ion monitoring of 3-NPH-*t*-ES ([M + H]^+^ = 403) is shown. Y-axis represents ion counts and the chromatograms are presented on the same scale. Note that 3-NPH-fumaric acid was also observed when succinic acid was used as the substrate.

To confirm the function of Cp1A as an epoxide-forming enzyme, we overexpressed and purified the recombinant Cp1A from *Escherichia coli* BL21(DE3) (Supplementary Fig. 7). Enzyme assays were performed in the presence of fumaric acid and the products were derivatized with 3-nitrophenylhydrazine (3-NPH). Liquid chromatography–mass spectrometry (LC–MS) analysis showed that fumaric acid was efficiently converted into *t*-ES by Cp1A in the presence of cofactors required for an Fe(II)/αKG-dependent oxygenase (Fig. 2c). Using a coupled assay with Cp1A and Cp1B to form *t*-ES-l-Ile (vide infra), the stereochemistry of the epoxide formed by Cp1A was assigned to be (2*S*,3*S*) on the basis of a comparison of retention times to those of synthetic (2*S*,3*S*)-*t*-ES-l-Ile (**14**) and (2*R*,3*R*)-*t*-ES-l-Ile (Extended Data Fig. 5a). Cp1A is not able to catalyze the epoxidation of the malyl-l-Ile-putrescine (**12**) and fumaryl-l-Ile-putrescine (**13**) (Extended Data Fig. 5b), demonstrating that Cp1A stereoselectively epoxidizes free fumaric acid to give the warhead (2*S*,3*S*)-*t*-ES. When we used succinic acid instead of fumaric acid as the substrate of Cp1A, 3-NPH-*t*-ES and 3-NPH-fumaric acid were produced, albeit with lower efficiency (Extended Data Fig. 5c). This observation was further confirmed by repeating the Cp1A assay with succinic acid-*d*_*4*_, which showed the incorporation of deuterium atoms into 3-NPH-fumaric acid (Supplementary Fig. 8). Therefore, Cp1A is a multifunctional Fe(II)/αKG-dependent oxygenase that can catalyze the desaturation of succinic acid to fumaric acid and subsequent epoxidation to (2*S*,3*S*)-*t*-ES, a feature that resembles the reported AsqJ^50^.

An analog of **1**, circinamide^51^, was isolated from cyanobacteria *Anabaena* spp. (Supplementary Fig. 1). While the genome of *Anabaena* spp. is unavailable, homologous BGCs to *cp1* and *cp2* are present in cyanobacteria genomes including *Microcoleus* spp. (*mfa*), despite low identities (~20%) between Mfa and Cp1 enzymes. Recombinant MfaA (Supplementary Fig. 7) is also able to catalyze the formation of (2*S*,3*S*)-*t*-ES from fumaric acid (Fig. 2c and Extended Data Fig. 5a), representing a bacterial example of *t*-ES synthase.

### Cp1B and Cp2B are pseudodipeptide-forming ATP-grasp enzymes

**Extended Data Fig. 6:**
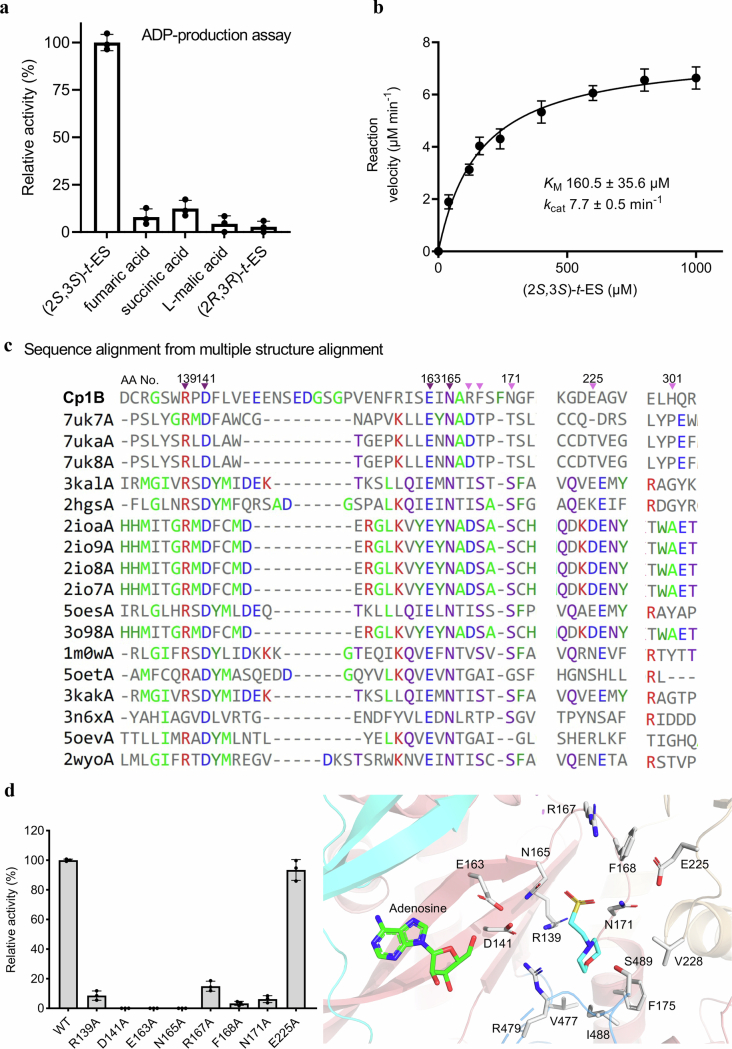
Cp1B is an ATP-grasp enzyme. **a**, Relative activity of ADP formation upon incubation of Cp1B with dicarboxylic acid substrates. Reactions were performed in 200 μL of 100 mM Tris-HCl (pH 8.0) containing 0.25 μM Cp1B, 10 mM ATP, 12 mM MgCl_2_, 300 μM NADH, 500 μM phosphoenolpyruvic acid (PEP), 41 units/mL pyruvate kinase (PK, Sigma), 59 units/mL lactate dehydrogenase (LDH, Sigma), 10 mM KCl with 1 mM acid donors and 5 mM l-Phe. The relative phosphorylation activities of Cp1B towards each substrate were derived by the consumption of NADH at the time point where each reaction mixture was incubated at 30 °C for 30 min. Values and error bars represent the average and s.d. of three independent replicates (black filled circles), respectively (*n* = 3). **b**, Apparent Michaelis-Menten plots for the Cp1B catalyzed phosphorylation of (2*S*,3*S*)-*t*-ES. The values represent means ± s.d., and error bars indicate s.d. of three independent replicates (*n* = 3). The reaction mixtures (100 μL) contained 1.0 μM Cp1B, 10 mM ATP, 12 mM MgCl_2_, 300 μM NADH, 500 μM phosphoenolpyruvic acid (PEP), 41 units/mL pyruvate kinase (PK, Sigma), 59 units/mL lactate dehydrogenase (LDH, Sigma), 10 mM KCl and 100 mM Tris-HCl (pH 8.0) with various concentration (0.04 mM to 1 mM) of (2*S*,3*S*)-*t*-ES and 5 mM l-Phe. The reaction mixture was incubated at 30 °C, and the consumption of NADH at 10 min was used to derive the reaction velocity for enzyme kinetics. Kinetic constants were derived from velocity versus substrate concentration data using a nonlinear regression fitting method with GraphPad Prism 9. **c**, Structure-based multiple sequence alignment of Cp1B with other characterized ATP-grasp enzymes. **d**, Activity of Cp1B mutants quantified by the formation of **14**. Reactions are performed at 30 °C for 20 min in 100 μL of 50 mM sodium phosphate buffer (pH 8.0). Reaction components are 25 μM Cp1B, 5 mM (±)-*t*-ES, 2.5 mM l-Ile, 10 mM ATP, and 10 mM MgCl_2_. Values and error bars represent the average and s.d. of three independent replicates (black filled circles), respectively (*n* = 3). Source data

The putative ATP-grasp enzyme Cp1B and the ANL-family enzyme Cp1D are responsible for the amide bond-forming steps to give **1** and analogs. Both recombinant enzymes were purified from *E.* *coli* (Supplementary Fig. 7). When assayed in the presence of (±)-*t*-ES, l-Ile, putrescine, ATP and MgCl_2_, formation of **4** was observed (Fig. 2d). When putrescine or Cp1D was omitted from the reaction mixture, formation of (2*S*,3*S*)-*t*-ES-l-Ile (**14**) was observed (Fig. 2d). Therefore, the first amide bond between (2*S*,3*S*)-*t*-ES and l-Ile is catalyzed by Cp1B, while formation of the second amide bond between **14** and putrescine is catalyzed by Cp1D (Fig. 2e). Cp1B is confirmed to be an ATP-grasp enzyme from the formation of adenosine diphosphate (ADP) in the reaction^47^ (Extended Data Fig. 6a). Replacing l-Ile with d-Ile did not lead to formation of (2*S*,3*S*)-*t*-ES-d-Ile, demonstrating the stereospecificity of Cp1B toward l-amino acids (Supplementary Fig. 9). Replacing sodium phosphate buffer with HEPES buffer did not result in a notable change in enzyme activity, confirming that phosphate does not inhibit the activity of Cp1B (Supplementary Fig. 10). Cp1B is also enantiospecific for (2*S*,3*S*)-*t*-ES and steady-state kinetics showed that Cp1B phosphorylated (2*S*,3*S*)-*t*-ES with *k*_cat_/*K*_M_ (apparent) of 48.0 mM^−1^ min^−1^ (Extended Data Fig. 6a,b). This property of Cp1B was used in a kinetic resolution of (±)-*t*-ES through the amide-forming reaction, during which (±)-*t*-ES was resolved through the formation of (2*S*,3*S*)-*t*-ES-l-Ile (**14**) with a diastereomeric ratio > 98:2 (Supplementary Fig. 11). Cp1B is, therefore, a suitable biocatalyst to monoamidate (2*S*,3*S*)-*t*-ES from racemic *t*-ES. In contrast, chemical synthesis of **1** and analogs requires asymmetric synthesis or resolution of stereochemically homogenous (2*S*,3*S*)-*t*-ES monoester for the subsequent monoamidation^6^ (Supplementary Fig. 12).

**Fig. 3:**
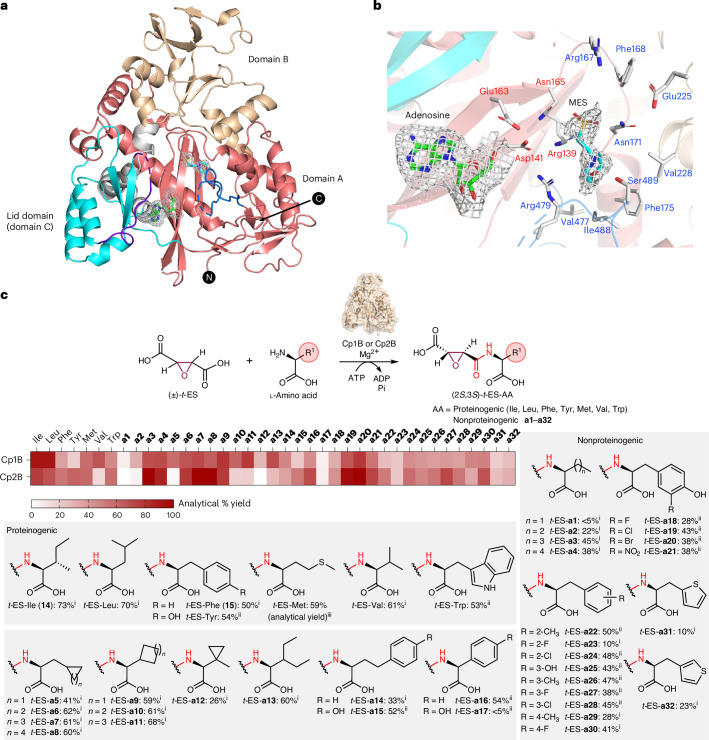
Stereoselective (2*S*,3*S*)-*t*-ES-l-amino acid synthesis by Cp1B and Cp2B. **a**, Crystal structure of Cp1B in a complex with adenosine and MES. Domains A (residues 5–200 and 444–502), B (residues 201–323) and C (residues 365–443) are shown as deep salmon, wheat and cyan, respectively. The helical connection (residues 324–364) between domains B and C is shown in gray. The P-loop and A-loop are highlighted in purple and blue, respectively. Adenosine is colored in light green and MES is colored in cyan. m*F*_o_ − D*F*_c_ polder omit maps for adenosine and MES are shown in gray mesh and contoured at 3.0*σ*. **b**, Enlarged view of the substrate-binding site. Conserved residues with hGSH synthetases are labeled in red, which are proposed to be involved in phosphorylation of substrate carboxylate. Residues surrounding MES that are possibly involved in substrate binding are shown in blue. **c**, Amino acid nucleophile (proteinogenic amino acids and **a1**–**a32**) scope for Cp1B and Cp2B. The heat map shows percentage yields in analytical-scale reactions catalyzed by Cp1B and Cp2B, estimated from the standard curves generated at *λ* = 204 nm of the purified compounds. The analytical-scale reactions were performed in 100 μl of 50 mM sodium phosphate buffer (pH 8.0) at 30 °C for 16 h. Each reaction contained 25 μM enzyme, 5 mM (±)-*t*-ES, 2.5 mM amino acid, 10 mM MgCl_2_ and 10 mM ATP. Isolated percentage yields from the preparative-scale reactions catalyzed using either Cp1B or Cp2B are shown under the structures. Preparative-scale reactions were performed in 20 ml of 50 mM sodium phosphate buffer (pH 8.0) at 30 °C for 16 h. Each reaction contained 2.5 μM Cp1B or Cp2B, 5 mM (±)-*t*-ES, 2.5 mM amino acid, 10 mM MgCl_2_ and 10 mM ATP. Notes on percentage yields: (i) isolated percentage yields with Cp1B; (ii) isolated percentage yields with Cp2B; (iii) analytical percentage yield estimated from the standard curves generated at *λ* = 204 nm of purified **14** is shown because *t*-ES-Met was not isolated from the preparative-scale reaction in this study.

**Extended Data Fig. 7:**
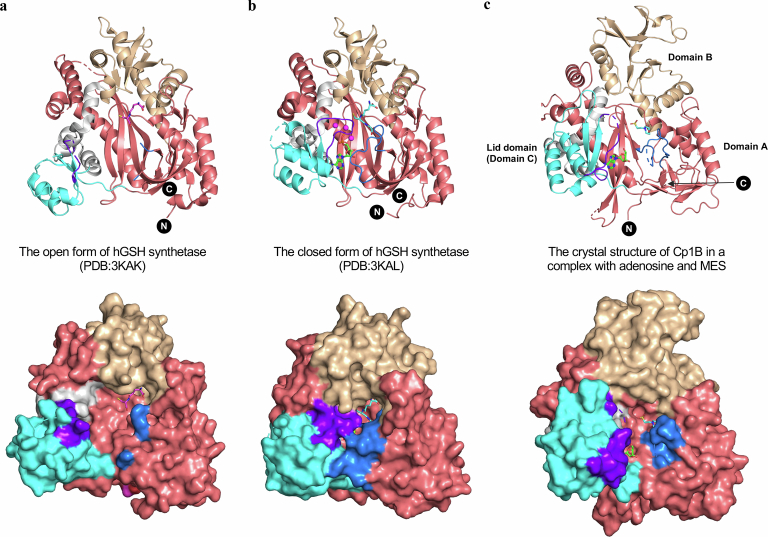
The crystal structure of Cp1B likely adopts a closed active site form. Comparisons of overall structures of (**a**) hGSH synthetase with the open active site form (3KAK), γ-glutamylcysteine is shown in magenta; (**b**) hGSH synthetase with closed active site form (3KAL). Mg^2+^ ions are shown as magenta spheres. ADP is shown in green and hGSH is shown in cyan; and (**c**) Cp1B in complex with adenosine shown in green and MES shown in cyan. Surface representations of the crystal structures are shown below. All structures have three characteristic domains typical of ATP-grasp enzymes: Domain A (deep salmon), Domain B (wheat), and lid domain (Domain C, cyan). P-loop (Gly-rich loop) and A-loop (Ala-rich loop) are shown in purple and blue, respectively. In the open form, the P-loop and A-loop are disordered in contrast to those in the closed form and in the Cp1B structure. Consequently, in the open form (3KAK), the nucleotide binding site open. In contrast, the lid domain with P-loop and A-loop enclose the active site in the closed form (3KAL) and partially in the Cp1B structure. These structural comparisons therefore suggested that the crystal structure of Cp1B adopts a closed active site, possibly as a result of MES binding in the active site.

The X-ray crystal structure of Cp1B complexed with adenosine and 2-morpholinoethanesulfonic acid (MES) was determined to 2.7-Å resolution (Fig. 3a and Supplementary Table 4). Despite having no detectable sequence identity with other characterized ATP-grasp enzymes, the structure of Cp1B displays the three-domain architecture (domains A, B and C) found in ATP-grasp enzymes such as hGSH synthetases, with a conserved ATP-binding site at the interface of domains A and C^47,52^ (Fig. 3a and Extended Data Fig. 7). Both open (Protein Data Bank (PDB) 3KAK) and closed (PDB 3KAL) active site forms of hGSH synthetases have been crystallized, with the phosphorylation and amide bond-forming steps proposed to be catalyzed in the closed form^52^. The ATP-binding site in Cp1B where adenosine resides is partially covered by the lid domain with the P-loop and the A-loop from domain A^52^ (Fig. 3a and Extended Data Fig. 7), indicating that the Cp1B structure is in the closed form. This unexpected closed form without a substrate or product could result from the binding of the buffer MES molecule in the active site. The morpholine moiety of MES is found to be housed in the hydrophobic pocket with F175, V228, V477 and I488 (Fig. 3b). Docking simulation of (2*S*,3*S*)-*t*-ES-l-Phe (**15**) with Cp1B showed that the side chain of l-Phe can also be housed in the same hydrophobic pocket, suggesting that the MES-binding site could be where the side chain of the l-amino acid binds (Supplementary Fig. 13). Similar to the ATP-binding site of hGSH synthetases, the adenosine in the Cp1B structure is sandwiched between the strands of two antiparallel β-sheets (Fig. 3a,b and Extended Data Figs. 6c and 7) and is surrounded by the conserved residues R139, D141, E163 and N165, which are likely involved in generation of the acyl-phosphate intermediate (Extended Data Fig. 6c and Supplementary Fig. 14). Individual substitutions of each residue to alanine either abolished or significantly attenuated enzymatic activity of Cp1B (Extended Data Fig. 6d and Supplementary Fig. 15). Hence, although annotated as a HP, our biochemical and structural characterization demonstrated that Cp1B is the biochemically validated example of an ATP-grasp enzyme in fungal natural product biosynthesis. Cp1B belongs to a family of ATP-grasp enzymes that does not require an α-amino acid as the electrophile, which is not commonly observed in characterized bacterial examples^30,31^.

We next explored the substrate scopes of Cp1B and Cp2B toward enzymatic synthesis of (2*S*,3*S*)-*t*-ES-AA, where AA is an l-amino acid. Cp1B and Cp2B preferentially monoamidated (2*S*,3*S*)-*t*-ES with hydrophobic amino acids, including l-Ile, l-Leu, l-Val, l-Met, l-Phe, l-Trp and l-Tyr, to varying yields (Fig. 3c). A wide range of nonproteinogenic and nonpolar amino acids (**a1**–**a32**) were also ligated to (2*S*,3*S*)-*t*-ES by Cp1B (Fig. 3c and Supplementary Fig. 16). Both enzymes, especially Cp2B, could also efficiently catalyze the monoamidation of (2*S*,3*S*)-*t*-ES with aromatic amino acids with diverse substitutions (**a18**–**a32**) (Fig. 3c). Nonaromatic amino acids with either nitrogen-containing or oxygen-containing side chains and α,α-disubstituted amino acids were not well tolerated by Cp1B and Cp2B (Supplementary Fig. 17). Overall, Cp1B preferred aliphatic amino acids and had a broader substrate scope than Cp2B, which preferred aromatic and bulkier amino acids (Fig. 3c). The complementary substrate scopes could, therefore, be leveraged toward the biocatalytic synthesis of a diverse array of (2*S*,3*S*)-*t*-ES-AA compounds. Indeed, 38 2,3-epoxyamides prepared with either Cp1B or Cp2B were isolated by a single-step purification from preparative enzymatic reactions and characterized by NMR (Fig. 3c, Supplementary Tables 17–54 and Supplementary Figs. 92–281).

### Cp1D and Cp2D are fungal ABSs

To confirm the role of Cp1D in catalyzing the second amide bond-forming reaction, Cp1D was directly assayed with (2*S*,3*S*)-*t*-ES-l-Ile (**14**) and an amine nucleophile. In the presence of putrescine, cadaverine or agmatine, Cp1D catalyzed the formation of **4**, **5** or **6**, respectively (Fig. 2d and Supplementary Fig. 18). Cp1D can efficiently amidate **14** to **6** with a *k*_cat_/*K*_M_ (apparent) of 79.8 mM^−1^ min^−1^, much more efficient compared to that toward (2*R*,3*R*)-*t*-ES-l-Ile (Supplementary Fig. 19). The mechanism of Cp1D was confirmed to be that of an ABS, as formation of **14**-adenosine monophosphate (AMP) in the presence of ATP was detected by LC–MS (Supplementary Fig. 19).

**Extended Data Fig. 8:**
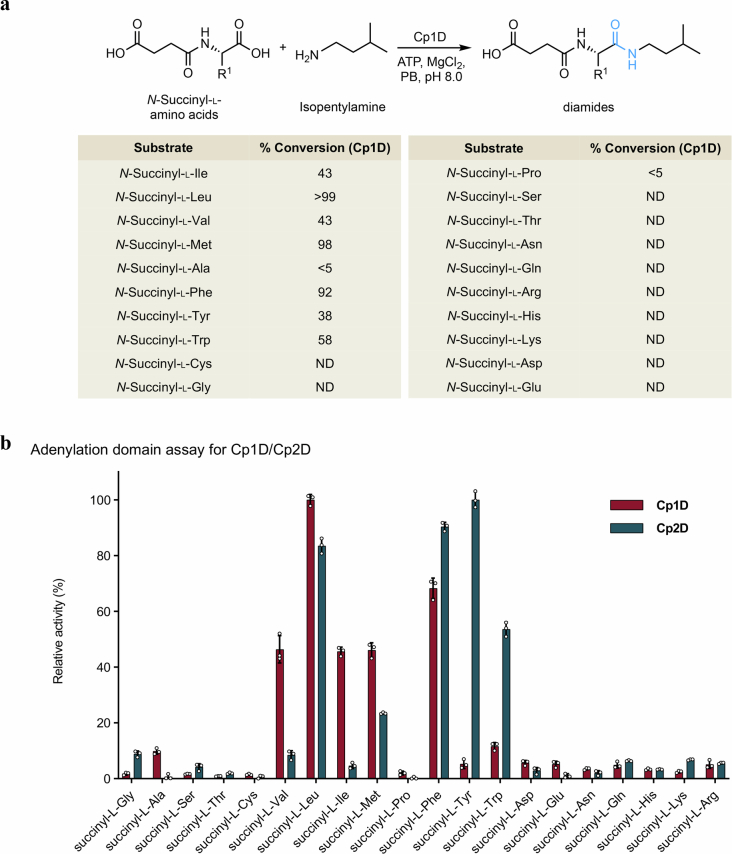
Substrate scope of Cp1D/Cp2D towards *N*-succinyl-AA. **a**, Substrate scope assay for Cp1D catalyzed amidation of *N*-succinyl-l-AA with isopentylamine. Cp1D was found to accept *N*-succinyl-l-AA of which the AA are hydrophobic amino acids (L, I, V, M, F, Y, and W). Assays were performed in 100 μL of 50 mM sodium phosphate buffer (pH 8.0) with 25 μM enzyme, 2 mM *N*-succinyl-l-AA, 5 mM isoamylamine, 10 mM MgCl_2_ and 10 mM ATP. Reactions were analyzed by LC/MS after incubation at 30 °C for 16 h. Analytical % conversion of *N*-succinyl-l-AA to the corresponding amide product was estimated from HPLC peak area ratios between product and starting material at λ = 204 nm (% Conversion = (peak area of product / (peak area of substrate + peak area of product)) × 100%). ND: not detected. **b**, Hydroxamate-based colorimetric assay was performed to assess adenylation specificity towards *N*-succinyl-l-AA for Cp1D/Cp2D. The reaction was performed in 150 μL of Tris buffer (pH 8.0) containing 20 μM of Cp1D or Cp2D, 15 mM of ATP, 5 mM of *N*-succinyl-l-AA, 200 mM hydroxylamine, and 10 mM MgCl_2_. After incubation for 8 h at 30 °C, the reaction was quenched by addition of equivalent volume of stopping solution (10% (w/v) FeCl_3_ and 3.3% (w/v) trichloroacetic acid dissolved in 0.7 M HCl). The precipitated enzyme was removed by centrifugation and the supernatant was measured for absorbance at 540 nm by a TECAN M200 plate reader. The absorbance at 540 nm was used to calculate the relative activity, and the absorbance of *N*-succinyl-l-Leu and *N*-succinyl-l-Tyr after the subtraction of that from each negative control (without Cp1D or Cp2D) were set as 100% activity for Cp1D and Cp2D, respectively. Values and error bars represent the average and s.d. of three independent replicates (white circles), respectively (*n* = 3). The assays confirmed that both Cp1D and Cp2D prefer hydrophobic l-amino acids in *N*-succinyl-l-AA, while Cp2D has a stronger preference for aromatic amino acids. Source data

The substrate scopes of Cp1D toward the pseudodipeptide electrophile and the amine nucleophile were comprehensively explored. First, 20 *N*-succinyl l-amino acids (suc-AA) were synthesized (Extended Data Fig. 8a and Supplementary Figs. 20 and 387–403) and assayed in the presence of ATP, MgCl_2_ and isopentylamine. Parallel to the substrate preference of Cp1B toward proteinogenic amino acids, Cp1D preferentially amidated seven suc-AA substrates containing hydrophobic amino acids (l-Ile, l-Leu, l-Val, l-Met, l-Phe, l-Trp and l-Tyr) (Extended Data Fig. 8a). Adenylation of suc-AA by Cp1D and Cp2D was also determined by the hydroxylamine-based colorimetric assay^53^ (Extended Data Fig. 8b). Cp2D adenylated suc-l-Trp and suc-l-Tyr more efficiently compared to Cp1D, in agreement with the preference for aromatic amino acids displayed by Cp2B over Cp1B. Using the 39 (2*S*,3*S*)-*t*-ES-AA prepared by Cp1B (Fig. 3c), Cp1D could amidate all with agmatine as judged by LC–MS analysis (Supplementary Fig. 21). Cp1D could also amidate the terminal carboxylate of l-Asp-l-Phe, l-Asp-l-Leu and glutaryl-l-Leu with moderate activity to form isopentyl amidated dipeptides (Supplementary Fig. 22). Cp1D and Cp2D do not have any sequence similarity to characterized bacterial ABSs such as McbA^32,36^, DdaF^40^, CfaL^34^ and CysC^37^ and can, therefore, be grouped into a distinct family of ABSs that activate *N*-acyl amino acids and dipeptides instead of simple organic acids by the bacterial ABSs (Fig. 1c and Supplementary Fig. 23).

**Fig. 4:**
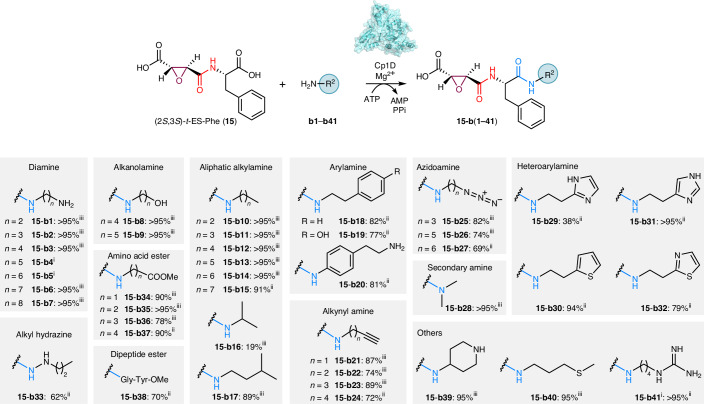
Substrate scope of Cp1D toward amine nucleophiles. Amine substrates (**b1**–**b41**) that were accepted by Cp1D as nucleophiles to form the corresponding diamides (**15**-**b1** to **15**-**b41**) using **15** as the electrophile. Analytical-scale reactions to estimate percentage yields were performed in 100 μl of 50 mM sodium phosphate buffer (pH 8.0) at 30 °C for 16 h. Each reaction contained 25 μM Cp1D, 2 mM **15**, 5 mM amine, 10 mM ATP and 10 mM MgCl_2_. The reactions were analyzed by LC–MS. Preparative-scale reactions to determine structures and isolated percentage yields were performed in 15 ml of 50 mM sodium phosphate buffer (pH 8.0) at 30 °C for 16 h. Each reaction contained 2.5 μM Cp1D, 2 mM **15**, 5 mM amine, 10 mM ATP and 10 mM MgCl_2_. Notes on analytical and isolated percentage yields: (i) analytical percentage yield was not determined as the product peak overlapped with **15** in the LC–MS chromatogram; (ii) isolated yields from preparative-scale reactions; (iii) estimated percentage yields of the amide products from analytical-scale reactions. The analytical percentage yields were estimated from the standard curves generated at *λ* = 204 nm of purified **15**-**b15** (for **15**-**b1** to **15**-**b17**, **15**-**b28**, **15**-**b39** and **15**-**b40**), **15**-**b24** (for **15**-**b21** to **15**-**b23**), **15**-**b27** (for **15**-**b25** to **15**-**b26**) or **15**-**b37** (for **15**-**b34** to **15**-**b36**).

The nucleophile scope of Cp1D was assessed using (2*S*,3*S*)-*t*-ES-l-Phe (**15**) and diverse amines (Fig. 4 and Supplementary Fig. 24). Remarkably, Cp1D could accept 41 of the selected amine nucleophiles with different chain lengths to form analogs of **1** with excellent yields (Fig. 4). These included primary amines such as diamines (**b1**–**b7**); alkanoamines (**b8**–**b9**), aliphatic amines (**b10**–**b17**), arylamines (**b18**–**b20**), alkynyl amines (**b21**–**b24**), azidoamines (**b25**–**b27**), heteroarylamines (**b29**–**b32**) and alkyl hydrazine (**b33**). To determine isolated yields of the products, we performed preparative-scale reactions to purify and characterize **15** condensed with **b15**, **b18**–**b20**, **b24**, **b27**, **b29**–**b33**, **b37**, **b38** and **b41** by NMR (Fig. 4, Supplementary Tables 55–68 and Supplementary Figs. 282–351). NMR analysis of **15**-**b20** revealed unexpected amine selectivity by Cp1D toward 4-(2-aminoethyl)aniline, as the amide bond was only formed through the aniline amine instead of the more nucleophilic aliphatic amine. Alkylamines with carboxylic acids at one end were not tolerated by Cp1D (Supplementary Fig. 25), although alkylamines capped with carboxylic acid methyl ester (**b34**–**b37**) could be efficiently incorporated into diamide products. Among secondary amine nucleophiles, dimethylamine (**b28**) was an excellent substrate for Cp1D, while bulkier amines were not accepted (Supplementary Fig. 25). Most amino acids and their methyl esters, except for Gly-OMe (**b34**), were not accepted as an amine donor by Cp1D. The dipeptide Gly-l-Tyr-OMe (**b38**) was well accepted by Cp1D as a nucleophile, suggesting that the substrate scope could be further explored toward synthesis of pseudotetrapeptides.

### Enzymatic combinatorial synthesis and inhibitor screening

Combinatorial biocatalysis is the enzymatic version of combinatorial synthesis, involving the use of cascaded enzymatic transformations for library construction^54–56^. Given the broad and orthogonal substrate scopes established for Cp1B and Cp1D, a library of more than 1,200 E-64 analogs can be generated in one-pot reactions. To access a fraction of that library in a proof-of-concept study, a 96-well assay containing (±)-*t*-ES, Cp1B and Cp1D, eight proteinogenic amino acids (l-Ile, l-Leu, l-Val, l-Met, l-Phe, l-Trp and l-Tyr, with l-His as a negative control) and 12 amine donors including additionally tested amines such as spermidine (**b42**), 4-(2-aminoethyl)-pyridine (**b43**) and tryptamine (**b44**) were performed. Masses correspond to the expected products were detected in all samples except those with l-His as the negative control (Supplementary Fig. 26).

**Fig. 5:**
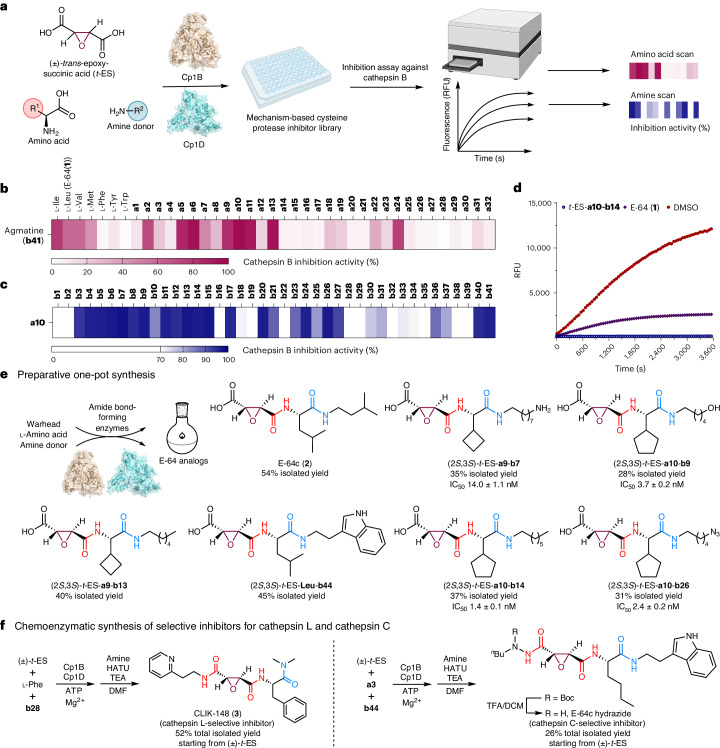
Biocatalytic platform for screening and synthesis of *t-*ES-based cysteine protease inhibitors. **a**, General workflow. Created with BioRender.com. **b**, Cathepsin B inhibitory activity of the crude reaction containing (2*S*,3*S*)-*t*-ES-AA-agmatine (**b41**) where AA is an amino acid (proteinogenic amino acids and **a1**–**a32**). **c**, Cathepsin B inhibitory activity of the crude reaction containing (2*S*,3*S*)-*t*-ES-**a10**-B where B is an amine (**b1**–**b41**). The average inhibition activity is presented as a percentage of the positive control (*n* = 2). **d**, Selected data output for fluorescence-based cathepsin B inhibition assay using a fluorogenic substrate *Z*-Phe-Arg-AMC. **e**, Biocatalytic one-pot synthesis of **2** and the identified cathepsin B inhibitors. The percentage isolated yields are shown for all synthesized compounds. IC_50_ values of the selected inhibitors against cathepsin B are also shown as the mean ± s.d. (*n* = 3). Reaction conditions for **b**,**c**: 25 μM Cp1B, 25 μM Cp1D, 2 mM (±)-*t*-ES, 1 mM l-amino acid, 1 mM amine, 10 mM ATP and 10 mM MgCl_2_ in 100 μl of 50 mM sodium phosphate buffer (pH 8.0) at 30 °C for 16 h. Reaction conditions for **e**: 5 mM (±)-*t*-ES, 2.5 mM l-amino acid, 2.5 mM amine donor, 10 mM ATP, 10 mM MgCl_2_, 2.5 μM Cp1B and 2.5 μM Cp1D in 50 mM sodium phosphate (pH 8.0) at 30 °C for 16 h. **f**, Chemoenzymatic synthesis of CLIK-148 (**3**) in preparative-scale reaction. After the biocatalytic synthesis of (2*S*,3*S*)-*t*-ES-**Phe**-**b28** using l-Phe and dimethylamine (**b28**), the crude mixture was directly used for the subsequent chemical condensation. The chemoenzymatic approach can also be applied to synthesize the cathepsin C-selective inhibitors starting with the biocatalytic synthesis of (2*S*,3*S*)-*t*-ES-**a3**-**b44**.

By coupling this biocatalytic platform with a fluorescence-based assay (Fig. 5a), we set out to identify potent inhibitors toward human cathepsin B. A library of 39 analogs of (2*S*,3*S*)-*t*-ES-AA-agmatine (**b41**) was generated by varying the amino acid component (Supplementary Fig. 21). The crude reaction mixtures in 96-well format were directly screened for cathepsin B inhibitory activity in a fluorometric endpoint assay^57^. In agreement with the reported structure–activity relationship for cathepsin B inhibitors^9,58^, analogs with an aliphatic amino acid such as l-cyclobutylalanine (**a6**), l-cyclopentylglycine (**a10**), l-cyclohexylglycine (**a11**) and 3-ethyl-l-norvaline (**a13**) exhibited the strongest cathepsin B inhibition (Fig. 5b). We then enzymatically prepared a second library with l-cyclopentylglycine (**a10**) as the amino acid building block and 41 different amine nucleophiles (Fig. 5c and Supplementary Fig. 27). While arylamines (**b18** and **b19**) and heteroarylamines (**b29**–**b32**) decreased the inhibitory activity compared to agmatine (**b41**), alkyl amines with terminal amine (**b7**), alcohol (**b9**), methyl (**b13**–**b15**), alkyne (**b24**) and azide (**b26**) groups all displayed comparable or more potent inhibition (Fig. 5c,d). Therefore, this combinatorial approach for cysteine protease inhibitor screening can be extended to other disease-relevant enzymes^59^.

### Preparative synthesis of E-64 analogs

**Extended Data Fig. 9:**
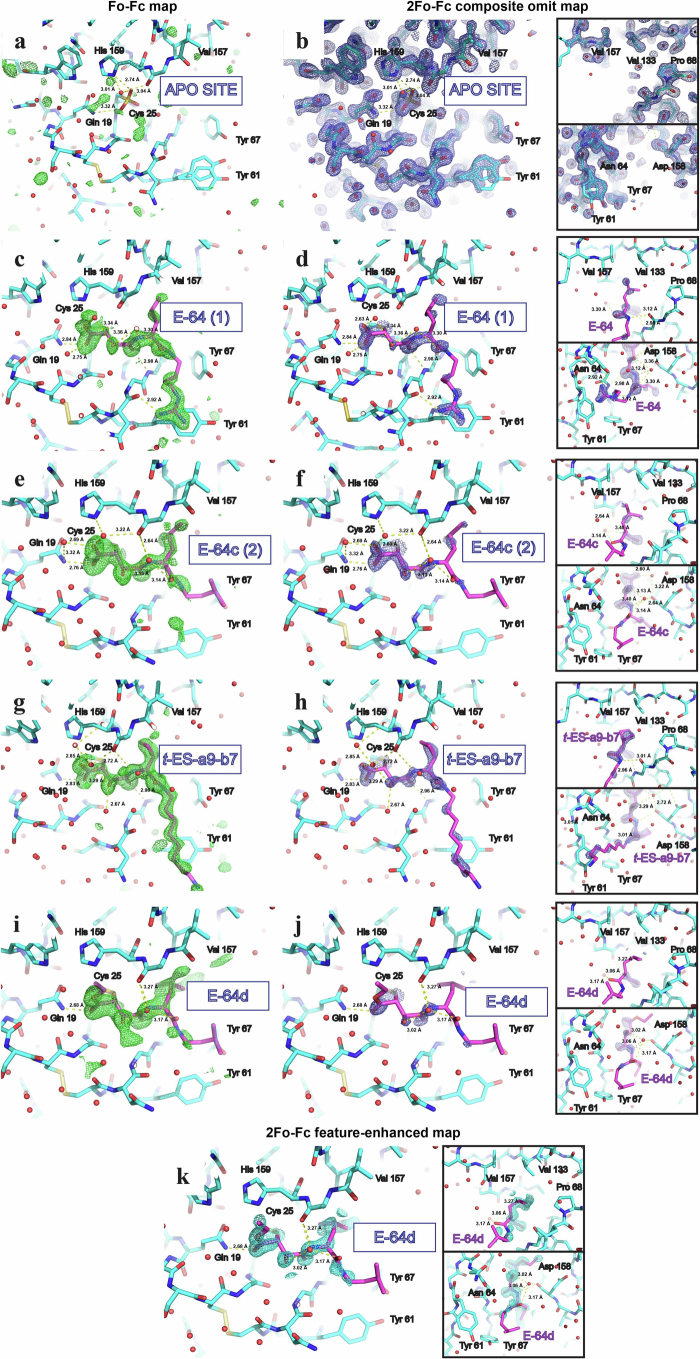
Structures of the active site of papain (no inhibitor bound, panels a-b) and papain bound to E64 analogs from X-ray diffraction (panels c-k). For the unliganded active site, atomic coordinates are superimposed on an Fo-Fc map at 3σ (green mesh) following complete modeling and refinement (**a**), and the 2Fo-Fc composite omit map (indigo mesh) generated following refinement at 1.5σ (**b**). For E-64 (**1**)-bound active site, atomic coordinates are superimposed on a ligand-omit Fo-Fc map at 3σ, revealing positive density that **1** (translucent magenta model) was modeled to fit (**c**), and the fully refined structure with the ligand modeled is superimposed on a 2Fo-Fc composite omit map at 1.5σ carved a 1.6 Angstrom radius from all ligand atoms (**d**). The same is shown for the papain-E-64c active site (**e-f**) the papain-(2*S*,3*S*)-*t*-ES-**a9**-**b7** active site (**g-h**), and the papain-E-64d active site (**i-j**). The same papain-E-64d structure is additionally superimposed against a 2Fo-Fc feature-enhanced map (cyan mesh) carved a 1.6 Angstrom radius from all E-64d atoms (**k**). Insets for each structure highlight a hydrophobic pocket adjacent to the active site occupied by hydrophobic side-chains of each ligand (top inset), and the solvent-facing region adjacent to the active site occupied by each inhibitor’s tail (bottom inset). Yellow dashed lines indicate potential hydrogen-bonding interactions.

To characterize the inhibitory activities of compounds identified from the biocatalytic platform, preparative-scale reactions with Cp1B and Cp1D in a single reaction vessel were performed. The targeted compounds included (2*S*,3*S*)-*t*-ES-**a9**-**b7**, (2*S*,3*S*)-*t*-ES-**a9**-**b13**, (2*S*,3*S*)-*t*-ES-**a10**-**b9**, (2*S*,3*S*)-*t*-ES-**a10**-**b14**, (2*S*,3*S*)-*t*-ES-**a10**-**b26**, (2*S*,3*S*)-*t*-ES-**Leu**-**b44** and the investigational drug E-64c (**2**) (Fig. 5e). Chemical synthesis of these compounds would require not only the preparation of the (2*S*,3*S*)-*t*-ES monoester but also subsequent multistep synthesis with protection–deprotection steps^6^ (Supplementary Fig. 28). In contrast, biocatalytic synthesis readily afforded the targeted compounds with isolated yields between 28% and 54% (Fig. 5e, Supplementary Tables 69–74 and Supplementary Figs. 40–41 and 352–381). Preparative synthesis of **2** coupled with an ATP regeneration system afforded nearly the same yields^60^ (Supplementary Fig. 29). Detailed kinetic characterization confirmed that the analogs (2*S*,3*S*)-*t*-ES-**a10**-**b9**, (2*S*,3*S*)-*t*-ES-**a10**-**b14** and (2*S*,3*S*)-*t*-ES-**a10**-**b26** all exhibited lower half-maximal inhibitory concentration (IC_50_) values toward cathepsin B than **1** (Fig. 5d,e and Supplementary Fig. 30). These three compounds also exhibited more potent or comparable inhibitory activities to that of **1** toward cathepsins L and K, demonstrating that these act as pan-cathepsin inhibitors rather than selective inhibitors (Supplementary Fig. 31). The covalent adduct between the (2*S*,3*S*)-*t*-ES moiety in these generated inhibitors and active site cysteines is expected to be the mechanism of irreversible inhibition^20^. This was confirmed by preincubation of (2*S*,3*S*)-*t*-ES-**a9**-**b7** with cathepsin B for 30 min at ten times the IC_50_, which completely inactivated cathepsin B when assayed with fluorogenic substrate following 100-fold dilution^57^ (Supplementary Fig. 32). Furthermore, when crystallized with the cysteine protease papain, (2*S*,3*S*)-*t*-ES-**a9**-**b7** was observed to be bound covalently to the active site cysteine in the same conformation as **1** (Extended Data Fig. 9 and Supplementary Table 5).

The one-pot reaction with Cp1B and Cp1D could not directly synthesize selective cathepsin L inhibitors such as CLIK-148, which have an additional amide attached to the *t*-ES warhead^26,61–63^ (Fig. 5f). A combination of biocatalytic and chemical syntheses was explored to access these compounds. For example, CLIK-148 could be obtained with ~50% total isolated yield from the biocatalytic synthesis of (2*S*,3*S*)-*t*-ES-**Phe**-**b28**, followed by chemical condensation with 2-pyridylethylamine (Fig. 5f, Supplementary Table 7 and Supplementary Figs. 42–46). This process is considerably simpler than the reported synthesis of CLIK-148 that requires more than six steps starting with the expensive (2*S*,3*S*)-*t*-ES diethyl ester^6,64^ (Supplementary Fig. 28). Similarly, the chemoenzymatic approach also led to the synthesis of a cathepsin C-selective inhibitor, a derivative of E-64c hydrazide^65^, with 26% total yield (Fig. 5f, Supplementary Table 75 and Supplementary Figs. 382–386). Therefore, such integration of chemical and biocatalytic synthesis can further expand the structure diversity of *t*-ES-based inhibitors that can be screened for desired potency and selectivity.

## Discussion

Biosynthetic investigation of natural products has contributed greatly to the identification of enzymes for synthesis and modification of bioactive compounds^42^. In this work, we discovered two families of amide bond-forming enzymes that have not been previously characterized from fungi: the ATP-grasp enzymes Cp1B and Cp2B that were initially annotated as HPs and the ABSs Cp1D and Cp2D annotated as ANL-family proteins. We uncovered the roles of these enzyme in catalyzing amide bond formations during the biosynthesis of **1** and analogs. It is somewhat surprising at the onset of this study that the biosynthesis of **1** remained elusive despite the important role of **1** and related compounds in chemical biology and drug discovery. The difficulties in the identification of the correct BGC could be because the biosynthetic pathway does not involve NRPSs for the amide-forming steps. In particular, NRPS-independent amide bond formation in the biosynthesis of fungal natural products is rare^42,66–68^. Our discovery and characterization of Cp1B and Cp1D, hence, establish this mode of amide bond formation in fungal secondary metabolism. Homologous BGCs are widely conserved in hundreds of Ascomycetes species, most of which are not known to produce E-64 analogs (Extended Data Figs. 2c and 3). Many homologs of Cp1B and/or Cp1D with >30% amino acid identity can be readily found fungal BGCs that are not homologous to that of **1** (Extended Data Fig. 2c and Supplementary Fig. 23). Genome-mining efforts using Cp1B and Cp1D as enzymatic beacons could lead to the discovery of amide bond-containing natural products.

The concept of enzymatic combinatorial synthesis has emerged as a promising technology for generating compound libraries in drug discovery^54–56^. Several ATP-grasp enzymes and ABSs have been identified from the biosynthetic pathways of bacterial bioactive oligopeptides^31,32,34^, including warhead-armed pseudopeptides dapdiamides^40^, rhizocticins^69^ and cystargolides^37,70^ (Supplementary Fig. 3). While these bacterial enzymes have been explored for the generation of natural product analogs or applications in biocatalysis, the broad utility of amide bond-forming enzymes in enzymatic combinatorial synthesis has not been fully realized because of unwanted cross-reactivities of the enzymes toward substrates and/or difficulties in finding partnering enzymes with comparable substrate promiscuity^56^. For example, during the biosynthesis of dapdiamide E, at least three separate enzymatic transformations are sandwiched between the first amide bond formation catalyzed by DdaG (ABS) and the second amide bond formation catalyzed by DdaF (ATP-grasp enzyme)^40,41^ (Supplementary Fig. 3). Consequently, the biocatalytic potential of DdaG and DdaF for library synthesis is limited by such pathway complexity and substrate scopes of the other enzymes. Micklefield and coworkers demonstrated the diversification of cystargolides using amide bond-forming enzymes CysC and CysD following β-lactone warhead formation^37^. While this demonstration of pairing different amide bond-forming enzymes toward bioactive molecule synthesis is impressive, judicious selection of building block combinations and a stepwise procedure were required because of the similar substrate scopes of CysC and CysD. In this study, leveraging the broad and orthogonal substrate scopes of Cp1B and Cp1D, we accomplished biocatalytic syntheses to create a large library of E-64 analogs in one-pot reactions (Fig. 5a). The orthogonality of the substrate scope of Cp1B and Cp1D reflects the tripartite structural features of E-64 (dicarboxylic acid, amino acid and amine). High-throughput enzymatic library generation was directly coupled to fluorescence-based screening to rapidly identify and more potent cathepsin inhibitors (Fig. 5). This panel of epoxy-diamides could also be adapted for screening other proteases and design of ABPP probes.

Compared to chemical synthesis of E-64 and related compounds^6^ (Supplementary Fig. 28), the combined Cp1B and Cp1D enzymatic synthesis has several key advantages. First, the enantiospecificity displayed by Cp1B toward (2*S*,3*S*)-*t*-ES enables the use of (±)-*t*-ES as a starting material, which is much more cost-effective and does not require stereoselective synthesis. Secondly, the lack of cross-reactivity between Cp1B and Cp1D toward the amino acid and amine substrates, respectively, obviates the need for protection and deprotections steps. Thirdly, the ATP required for amide bond formation can be regenerated using cofactor-recycling methods^60^. The scalable one-pot enzymatic synthesis was demonstrated in the preparation of analogs of **1**, which enabled the determination of IC_50_ values, and use in cocrystallization studies. We also showcased the facile coupling of enzymatic and chemical syntheses in the preparative synthesis of cathepsin L-selective inhibitor CLIK-148 (ref. ^26^) and a cathepsin C-selective inhibitor^65^ (Fig. 5f and Supplementary Fig. 1). Although not exhaustively explored here, the enzymes can be explored to synthesize non-epoxide-containing pseudopeptides that have different applications (Supplementary Figs. 22 and 33).

In conclusion, nearly four decades after its initial discovery^14^, a family of BGCs involved in the biosynthesis of **1** and related compounds has been characterized. The use of amide bond-forming enzymes as versatile biocatalysts illustrates the power of repurposing biosynthetic enzymes for catalysis. We anticipate that further discovery of amide bond-forming enzymes will expand the toolbox available to accelerate drug discovery and development of greener chemical synthesis.

## Methods

### Metabolite analysis and compound isolation

The preparation of the protoplasts of *A.* *nidulans* and transformation are described in the Supplementary Methods. For small-scale metabolite analysis in *A.* *nidulans*, transformants containing the desired plasmids were selected from CD sorbitol agar (2% glucose as carbon source) appropriately supplemented with riboflavin, uracil and/or pyridoxine. CD-ST agar was inoculated with spores and incubated at 28 °C for 4 days. The agar was then collected and extracted with acetone for 30 min with sonication. After centrifugation, the supernatant (200 μl) was concentrated and resuspended in methanol (100 μl). The sample was subjected to LC–QTOF (quadrupole time-of-flight) analysis with an Agilent 6545 QTOF equipped with a reverse-phase column (Agilent Poroshell, 120 EC-C18; 2.7 μm, 3.0 × 50 mm) using positive-mode electrospray ionization with 1% acetonitrile in H_2_O (containing 0.1% formic acid) for the first 2 min, then a linear gradient of 1–95% for 9 min and finally 95% acetonitrile for 3 min with a flow rate of 0.6 ml min^−1^. The data were collected and analyzed using MassHunter 10.0 (Agilent). Some LC–MS analyses were performed on an Agilent LC–MSD iQ (Agilent InfinityLab Poroshell 120 Aq-C18; 2.7 μm, 100 Å, 2.1 × 100 mm) using positive-mode and negative-mode electrospray ionization with a linear gradient of 1–99% acetonitrile in H_2_O supplemented with 0.1% (v/v) formic acid in 13.25 min followed by 99% acetonitrile for 3 min with a flow rate of 0.6 ml min^−1^. The data were collected and analyzed using OpenLab CDS 2.4 (Agilent). For large-scale analysis, *A.* *nidulans* transformants were inoculated on 40 plates each containing 50 ml of CD-ST agar and were placed in a 28 °C incubator for 3–4 days. After 4 days, the solid agar cultures were cut into small pieces and extracted extensively with acetone. The residual was loaded on a normal-phase CombiFlash system and subjected to flash chromatography with a gradient of CH_2_Cl_2_ and methanol for initial separation. Metabolites of interest, tracked by analytical high-performance LC (HPLC) and LC–MS, were purified from the corresponding fractions by reverse-phase semipreparative HPLC with a COSMOSIL column with a flow rate of 4 ml min^−1^ of solvents A (H_2_O with 0.1% trifluoroacetic acid) and B (acetonitrile). NMR spectra were obtained with a Bruker AV500 spectrometer with a 5-mm dual cryoprobe at the UCLA Molecular Instrumentation Center. (^1^H-NMR, 500 MHz; ^13^C-NMR, 125 MHz). High-resolution mass spectra were also recorded on a Agilent 6545 QTOF high-resolution MS instrument (UCLA Molecular Instrumentation Center). The mass and NMR spectra were analyzed using MassHunter 10.0 (Agilent) and MestReNova-9.0.1 (Mestrelab Research), respectively.

### Protein expression and purification

The intron-free open reading frames encoding Cp1A, Cp1B, Cp1D, Cp2B, Cp2C and Cp2D were amplified by PCR using complementary DNA from the corresponding *A.* *nidulans* transformant as a template and ligated to linear expression vector pET28a by Gibson assembly (New England Biolabs) according to the manufacturer’s protocol. The *mfaA* gene sequence was obtained from the National Center for Biotechnology Information database, was codon-optimized on the basis of the codon preference of *E.* *coli* and synthesized by Integrated DNA Technologies (IDT). The gene encoding CHU (polyphosphate kinase) was also synthesized by IDT. The plasmids were then transformed into *E.* *coli* BL21(DE3) individually and grown overnight in 5 ml of Luria–Bertani (LB) medium with 50 μg ml^−1^ kanamycin at 37 °C. The overnight cultures were used as seed cultures for 1 L of fresh LB medium containing 50 μg ml^−1^ kanamycin and incubated at 37 °C until the OD_600_ reached 0.8. The cultures were cooled on ice before the addition of 0.1 mM isopropyl-β-d-thiogalactopyranoside (GoldBio) to induce protein expression. The expression was performed at 16 °C for 20 h at 220 rpm. *E.* *coli* cells were harvested by centrifugation at 5,200*g* for 15 min and resuspended in 30 ml of A10 buffer (50 mM sodium phosphate buffer, 150 mM NaCl and 10 mM imidazole, pH 8.0) containing one tablet of Pierce protease inhibitor (Thermo Fisher Scientific). The cell suspension was lysed on ice by sonication and the lysate was centrifuged at 17,000*g* for 30 min at 4 °C to remove the insoluble cellular debris. Recombinant 6×His-tagged proteins were purified at 4 °C from corresponding soluble fractions by affinity chromatography with Ni-NTA agarose resin (GE Healthcare). Briefly, recombinant proteins on the resin was initially washed with wash buffer A1 (50 mM sodium phosphate, 150 mM NaCl and 10 mM imidazole, pH 8.0) until no protein was detected in the eluent using the Bradford reagent. Then, the same procedure was repeated with wash buffer A2 (50 mM sodium phosphate, 150 mM NaCl and 20 mM imidazole, pH 8.0). The target protein was eluted by elution buffer A (50 mM sodium phosphate, 150 mM NaCl and 250 mM imidazole, pH 8.0). The purified proteins were concentrated and exchanged into storage buffer (50 mM sodium phosphate, 200 mM NaCl and 10% glycerol, pH 8.0) with an Amicon Ultra concentrator (30-kDa cutoff; Merck Millipore). SDS–PAGE was performed to check the protein purity and a Bradford protein assay (Bio-Rad) was used to calculate protein concentration with BSA (Sigma) as the standard. The proteins were aliquoted and stored at −80 °C until used in in vitro assays. The plasmids used for protein purification are listed in Supplementary Table 3. Results of SDS–PAGE analysis are presented in Supplementary Figs. 7 and 15.

To purify Cp1B for crystallization, the cell pellet of *E.* *coli* BL21(DE3) was resuspended in Tris buffer (50 mM Tris and 500 mM NaCl, pH 8.0) and lysed by sonication on ice. Cell debris was removed by centrifugation at 17,000*g*, 4 °C for 30 min. Recombinant proteins in the supernatant were purified using nickel–Sepharose resin (GE Healthcare) and initially washed with wash buffer B1 (50 mM Tris, 500 mM NaCl and 30 mM imidazole, pH 8.0) until no protein was detected in the eluent using the Bradford reagent. Then, the target protein was eluted by elution buffer B (50 mM Tris, 500 mM NaCl and 300 mM imidazole, pH 8.0). The elution containing the target protein was concentrated to 2 ml for size-exclusion chromatography. The protein was then loaded onto a size-exclusion chromatograph (GE Healthcare, Superdex 200) in buffer of 50 mM Tris pH 8.0 with 100 mM NaCl through the Bio-Rad chromatography system. The purified Cp1B was concentrated to 15 mg ml^−1^ for crystallization.

### Enzymatic assays

To assay the activities of Cp1A and MfaA, 100-μl reactions were performed at 30 °C for 3 h in 50 mM sodium phosphate buffer (pH 8.0) containing 0.2 mM FeSO_4_, 2 mM αKG, 2 mM ascorbate, 1 mM substrate and 10 μM Cp1A or MfaA. The reaction mixture in the absence of protein was prepared as the negative control. Enzyme reactions were quenched by adding 100 μl of acetonitrile and centrifuged at 17,000*g* for 5 min, before being subjected to 3-NPH derivatization^71^. To perform 3-NPH derivatization for the detection of 1,2-dicarboxylic acid, all reagents for derivatization were freshly prepared before use. Then, 50 μl of reaction mixture was sequentially treated with 50 μl of 50 mM 3-NPH (Sigma) in methanol and H_2_O (70:30, v/v), 50 μl of 50 mM EDC (Oakwood Chemical) in methanol and H_2_O (70:30, v/v) and 50 μl of 7% v/v pyridine (Acros Organics) in methanol and H_2_O (70:30, v/v) and mixed thoroughly. Derivatization mixtures were incubated at 37 °C for 30 min and centrifuged at 17,000*g* for 10 min. The supernatant was subjected to LC–QTOF analysis with an Agilent 6545 QTOF equipped with a reverse-phase column (Agilent Poroshell, 120 EC-C18; 2.7 μm, 3.0 × 50 mm) using positive-mode electrospray ionization with 1% acetonitrile in H_2_O (containing 0.1% formic acid) for the first 2 min, then a linear gradient of 1–95% for 9 min and finally 95% acetonitrile for 3 min with a flow rate of 0.6 ml min^−1^. The data were collected and analyzed by MassHunter 10.0 (Agilent).

To assay the activities of Cp1B and Cp2B, assays were carried out in 50 mM sodium phosphate buffer (pH 8.0) or 50 mM HEPES buffer (pH 8.0). A typical reaction contained 25 μM enzyme, 5 mM (±)-*t*-ES or other dicarboxylic acid, 2.5 mM amino acid, 10 mM ATP and 10 mM MgCl_2_ in 100 μl of 50 mM sodium phosphate buffer (pH 8.0). After incubation at 30 °C for 16 h, the reaction was then quenched with 120 μl of acetonitrile and centrifuged at 17,000*g* for 5 min. The supernatant was subjected to LC–QTOF analysis with the same conditions as mentioned above or analyzed by an Agilent LC–MSD iQ with a reverse-phase column (Agilent InfinityLab Poroshell 120 Aq-C18; 2.7 μm, 100 Å, 2.1 × 100 mm) using positive-mode and negative-mode electrospray ionization with a linear gradient of 1–99% acetonitrile in H_2_O supplemented with 0.1% (v/v) formic acid with a flow rate of 0.6 ml min^−1^. The data were collected and analyzed by OpenLab CDS 2.4 (Agilent). The detailed gradient conditions are listed in the figure legends of the Supplementary Figures. The analytical percentage yields shown in Fig. 3c (heat map) were estimated from the standard curves of enzymatically prepared standards generated from peak areas at 204 nm by HPLC. To determine the diastereomeric ratio values, the sample was analyzed by chiral analytical HPLC with a CHIRALPAK IA-3 column (150 × 4.6 mm, 3 μm) at room temperature (flow rate 1 ml min^−1^, 40% acetonitrile in H_2_O with 0.1% trifluoroacetic acid).

Because of the insolubility of Cp1C when expressed from *E.* *coli* BL21(DE3), the decarboxylase Cp2C from *cp2* cluster was expressed for characterization instead. In vitro assays of Cp2C were performed in 50 μl of 50 mM sodium phosphate buffer (pH 8.0), containing 50 μM Cp2C, 100 μM PLP, 2 mM l-amino acid such as l-ornithine, l-lysine and l-arginine at 30 °C. After 1 h at 30 °C, all reactions were quenched with 50 μl of acetonitrile, centrifuged at 17,000*g* for 5 min, subjected to dansyl derivatization with dansyl chloride (Tokyo Chemical Industry) and analyzed by LC–MS. Dansyl derivatization was performed by adding 50 μl of 1 M borate buffer (pH 8.0) and dansyl chloride solution (10 mM final concentration). After incubation at 30 °C for 1 h, the resultant reaction mixture was centrifuged at 17,000*g* for 5 min. The resultant supernatant was subjected to LC–QTOF analysis using the same gradient methods described above (Supplementary Fig. 34).

To assay the activities of Cp1D, assays were performed in 50 mM sodium phosphate buffer (pH 8.0) or 50 mM HEPES buffer (pH 8.0). A typical reaction contains 10 μM or 25 μM Cp1D, 2 mM or 2.5 mM monoamide such as **14** or **15**, 2.5 mM or 5 mM amine, 10 mM ATP and 10 mM MgCl_2_ in 100 μl of 50 mM sodium phosphate buffer (pH 8.0). After incubation at 30 °C for 16 h, the subsequent sample preparation and LC–QTOF or LC–MS (Agilent LC–MSD iQ) analysis followed the same methods as described for the Cp1B reaction. The detailed gradient conditions for LC–MS analysis are shown in each figure legend in the Supplementary Figures. The analytical percentage yields shown in Fig. 4 were estimated from the standard curves generated at *λ* = 204 nm of purified **15**-**b15** (for **15**-**b1** to **15**-**b17**, **15**-**b28**, **15**-**b39** and **15**-**b40**), **15**-**b24** (for **15**-**b21** to **15**-**b23**), **15**-**b27** (for **15**-**b25** to **15**-**b26**) or **15**-**b37** (for **15**-**b34** to **15**-**b36**). For kinetics analysis of Cp1D and Cp2D for the amidation, a similar procedure to that mentioned above was used except for various concentrations of a single diastereomer (2*S*,3*S*)-*t*-ES-Ile or (2*R*,3*R*)-*t*-ES-Ile. Briefly, 100 µl of reaction mixture in 50 mM sodium phosphate buffer (pH 8.0) contained 10 mM MgCl_2_, 10 mM ATP, 5 mM agmatine, 4 μM Cp1D or Cp2D, various concentrations of (2*S*,3*S*)-*t*-ES-Ile or (2 *R*,3 *R*)-*t*-ES-Ile. The reaction was incubated at 30 °C for 5 min and quenched with 100 µl of acetonitrile, which was subjected to LC–MS analysis. The apparent kinetic constants are derived from the formation of the corresponding product (velocity) versus substrate concentration data using a nonlinear regression fitting method with GraphPad Prism 9.

To generate Cp1B mutants, the plasmid pML8010 containing the wild-type *cp1B* gene was used as the template for PCR-based site-directed mutagenesis. DNA sequencing was used to confirm the identities including the mutated positions of the expression plasmids. Following expression and purification, Cp1B mutants were subjected to activity assays as described above. Reactions were performed at 30 °C for 20 min in 100 μl of 50 mM sodium phosphate buffer (pH 8.0). Reaction components were 25 μM Cp1B or mutants, 5 mM (±)-*t*-ES, 2.5 mM l-Ile, 10 mM ATP and 10 mM MgCl_2_. The relative activities of Cp1B mutants were calculated by setting the activity of the wild type at 100%, quantified by the formation of **14**.

### Stepwise enzymatic assay with Cp1A/MfaA and Cp1B

Purified Cp1A or MfaA was added to 50 ml of 50 mM sodium phosphate buffer (pH 8.0) containing 0.2 mM FeSO_4_, 2 mM αKG, 2 mM ascorbate, 1 mM fumaric acid and 10 μM Cp1A or MfaA at 30 °C for 16 h. The enzyme was then removed by Amicon concentrators (Millipore). Subsequently 10 μM Cp1B, 2.5 mM l-isoleucine, ATP cofactor (10 mM) and MgCl_2_ (10 mM) were added, followed by incubation at 30 °C for 16 h. The enzyme in reaction solution was again removed by ultrafiltration. The filtrate was adjusted to pH ~3 with 4 M H_2_SO_4_ solution and extracted with ethyl acetate. Organic solvent was removed under reduced pressure and further purified using semipreparative HPLC. Purified product was subjected to NMR analysis and analysis using chiral analytical HPLC with a CHIRALPAK IA-3 column (150 × 4.6 mm, 3 μm) at room temperature (flow rate 1 ml min^−1^, 40% acetonitrile in H_2_O with 0.1% trifluoroacetic acid).

### Coupled activity assay with Cp1B and Cp1D

The coupled activity assay for Cp1B with Cp1D was typically performed in 100 μl of 50 mM sodium phosphate buffer (pH 8.0) containing 25 μM Cp1B, 25 μM Cp1D, 5 mM (±)-*t*-ES, 2.5 mM amino acid, 5 mM amine, 10 mM ATP cofactor and 10 mM MgCl_2_. The reaction mixture was incubated at 30 °C for 16 h before quenching the reaction. The subsequent sample preparation and LC–MS analysis followed the same methods as described for the Cp1B reaction.

### Detection of ADP from Cp1B assay

The reaction mixtures (200 μl) in 100 mM Tris-HCl buffer (pH 8.0) contained 0.25 μM Cp1B, 10 mM ATP, 12 mM MgCl_2_, 300 μM reduced nicotinamide adenine dinucleotide (NADH), 500 μM phosphoenolpyruvic acid (PEP), 41 U per ml pyruvate kinase (PK; Sigma), 59 U per ml lactate dehydrogenase (LDH; Sigma), 10 mM KCl with 1 mM ES or other acid donors and 5 mM l-Phe. PK and LDH were stored in 10 mM HEPES (pH 7.0) with 100 mM KCl and 0.1 mM EDTA with 50% glycerol. PK requires potassium ion as an essential cofactor for its activity. The reaction mixture was incubated at 30 °C and the consumption of NADH was monitored continuously for 60 min with a TECAN M200 plate reader by measuring the absorbance at 340 nm. The consumption of NADH reflects the formation of ADP upon Cp1B-catalyzed formation of acyl-phosphate. Therefore, the phosphorylation activity of Cp1B toward each substrate was derived from the consumption of NADH (the formation of ADP). The phosphorylation activity of Cp1B for (2*S*,3*S*)-*t*-ES was set as 100% activity to calculate the relative activity for the other substrates. The results are shown in Extended Data Fig. 6a.

For kinetics analysis of Cp1B for the phosphorylation, the similar procedure mentioned above was used except for various concentrations of (2*S*,3*S*)-*t*-ES being used. Briefly, the reaction mixtures (100 μl) in 100 mM Tris-HCl buffer (pH 8.0) contained 1.0 μM Cp1B, 10 mM ATP, 12 mM MgCl_2_, 300 μM NADH, 500 μM PEP, 41 U per ml PK (Sigma), 59 U per ml LDH (Sigma) and 10 mM KCl with various concentrations (0.04 mM to 1 mM) of (2*S*,3*S*)-*t*-ES and 5 mM l-Phe. The reaction mixture was incubated at 30 °C and the consumption of NADH at 10 min was used to derive the reaction rate (velocity) for phosphorylation. Kinetic constants (apparent) were derived from velocity versus substrate concentration data using a nonlinear regression fitting method with GraphPad Prism 9. The result is shown in Extended Data Fig. 6b.

### Hydroxamate-based colorimetric assay

A hydroxamate-based colorimetric assay^53^ was used to test substrate specificity toward *N*-succinyl amino acids for ABS Cp1D or Cp2D (ref. ^72^). The reaction was performed in 150 μl of 50 mM Tris buffer (pH 8.0) containing 20 μM Cp1D or Cp2D, 15 mM ATP, 5 mM *N*-succinyl amino acid substrate, 200 mM hydroxylamine and 10 mM MgCl_2_. The reaction was quenched after incubation for 8 h at 30 °C by addition of equivalent volume (150 μl) of stopping solution (10% (w/v) FeCl_3_ and 3.3% (w/v) trichloroacetic acid dissolved in 0.7 M HCl). The precipitated enzyme was removed by centrifugation, 200 μl of the supernatant was transferred to a 96-well plate and the absorbance of the ferric-hydroxamate complex at 540 nm was measured by a Tecan M200 plate reader. The absorbance at 540 nm was used to calculate the relative activity shown in Extended Data Fig. 8b and the absorbances of *N*-succinyl-l-Leu and *N*-succinyl-l-Tyr after subtracting that of the negative control (without Cp1D or Cp2D) were set as 100% activity for Cp1D and Cp2D, respectively.

### Enzymatic synthesis of *t-*ES amino acids

To obtain (2*S*,3*S*)-*t-*ES amino acids, a 20 ml reaction in 50 mM sodium phosphate buffer (pH 8.0) containing purified 2.5 μM Cp1B or Cp2B with 5 mM (±)-*t*-ES, 2.5 mM amino acid, 10 mM ATP and 10 mM MgCl_2_ was performed at 30 °C for 16 h. The protein was removed by Amicon concentrators (Millipore) and the concentrate was washed twice with three volumes of water. The filtrate was combined and was carefully adjusted to pH 2–3 with 4 M H_2_SO_4_ solution. The acidified filtrate was further extracted with an equal volume of ethyl acetate twice. The combined organic layer was washed with brine, dried over MgSO_4_ and filtered. The solvent was evaporated in vacuo to give a crude mixture that was further purified by HPLC (water and acetonitrile, both supplemented with 0.1% trifluoroacetic acid) on a Cosmosil C18 AR-II column (5.0 µm, 10 × 250 mm; Nacalai Tesque) to afford the products with varying isolated yields. The isolated yield for each compound is shown in Fig. 3c. All isolated compounds were characterized by NMR (Supplementary Tables 17–54 and Supplementary Figs. 92–281).

### Enzymatic synthesis of (2*S*,3*S*)-*t*-ES-Phe-amine

The 15-ml reactions in 50 mM sodium phosphate buffer (pH 8.0) containing 2.0 mM **15**, 5.0 mM amine donor and 2.5 μM Cp1D were performed at 30 °C for 16 h. Protein was removed by Amicon concentrators (Millipore) and the concentrate was washed twice with three volumes of water. For reactions containing **b20**, **b29**, **b31**, **b32** and **b41** as amine donors, the filtrate was evaporated in vacuo. The residue was dissolved in DMF and further purified with HPLC with a Cosmosil column (Nacalai Tesque, 5C18-AR-II; 10 × 250 mm) with a flow rate of 4 ml min^−1^ of solvents A (H_2_O with 0.1% trifluoroacetic acid) and B (acetonitrile with 0.1% trifluoroacetic acid). For other amine donors containing hydrophobic functional groups, the filtrate was combined and the pH was adjusted to around 2–3 with 4 M H_2_SO_4_ solution. The acidified filtrate was further extracted with equal volume ethyl acetate twice. The combined organic layer was washed with brine, dried over MgSO_4_ and filtered. The solvent was evaporated in vacuo to give a crude mixture that was further purified by HPLC (water and acetonitrile, both supplemented with 0.1% trifluoroacetic acid) on a Cosmosil, C18 AR-II column (5.0 µm, 10 × 250 mm; Nacalai Tesque) to afford the corresponding product with varying isolated yields as shown in Fig. 4. All compounds were characterized by NMR (Supplementary Tables 55–68 and Supplementary Figs. 282–351).

### Enzymatic synthesis of cysteine protease inhibitors

A large-scale 20-ml reaction in 50 mM sodium phosphate buffer (pH 8.0) containing 5 mM (±)-*t*-ES, 2.5 mM l-amino acid, 2.5 mM amine donor, 2.5 μM Cp1B and 2.5 μM Cp1D was carried out at 30 °C for 16 h. The protein was removed by Amicon concentrators (Millipore) and the concentrate was washed two times with three volumes of water. For diamine or alkanoamine as amine donor, the filtrate was evaporated in vacuo and then directly subject to reverse-phase CombiFlash system (Teledyne) with a gradient of acetonitrile in H_2_O (0–5 min, 0–5% acetonitrile; 5–10 min, 5–20% acetonitrile; 10–20 min, 20–60% acetonitrile; 20–25 min, 60% B; 25–35 min, 100% B). Fractions containing target compound were combined and further purified with HPLC with a Cosmosil column (Nacalai Tesque, 5C18-AR-II; 10 × 250 mm) with a flow rate of 4 ml min^−1^ of solvents A (H_2_O with 0.1% trifluoroacetic acid) and B (acetonitrile with 0.1% trifluoroacetic acid). For other amine donors containing hydrophobic functional groups, the filtrate was combined and pH was adjusted to around 2–3 with 4 M H_2_SO_4_ solution. The acidified filtrate was further extracted with an equal volume of ethyl acetate for two times. The combined organic layer was washed with brine, dried over MgSO_4_ and filtered. The solvent was evaporated in vacuo to give a crude mixture that was further purified by HPLC. The isolated yield for each compound is shown in Fig. 5e. For example, 8.5 mg of E-64c was obtained with 54% isolated yield from a one-pot reaction. The spectroscopic and physical properties of E-64c were identical to those reported in the literature^73^. Isolated yields for other compounds were as follows: (2*S*,3*S*)-*t*-ES-**a9**-**b7**, 6.5 mg and 35% yield; (2*S*,3*S*)-*t*-ES-**a9**-**b13**, 6.5 mg and 40% yield; (2*S*,3*S*)-*t*-ES-**a10**-**b9**, 4.8 mg and 28% yield; (2*S*,3*S*)-*t*-ES-**a10**-**b14**, 6.7 mg and 37% yield; (2*S*,3*S*)-*t*-ES-**a10**-**b26**, 5.7 mg and 31% yield; (2*S*,3*S*)-*t*-ES-**Leu**-**b44**, 8.7 mg and 45% yield. All compounds were characterized by NMR (Supplementary Tables 69–74 and Supplementary Figs. 40, 41 and 352–381).

### Chemoenzymatic synthesis of cysteine protease inhibitor

To chemoenzymatically synthesize selective cathepsin L inhibitor CLIK-148, a large-scale 20-ml reaction in 50 mM sodium phosphate buffer (pH 8.0) containing 5 mM (±)-*t*-ES, 2.5 mM l-Phe, 2.5 mM dimethylamine (**b28**), 2.5 μM Cp1B and 2.5 μM Cp1D was carried out at 30 °C for 16 h. Protein was removed by following the same procedure as mentioned above. The subsequent filtrate acidification and extraction followed the same methods as described for amine donors with a hydrophobic terminal. Solvent was evaporated in vacuo to give a crude mixture. The crude mixture was then dissolved in DMF, before adding 2-(2-aminoethyl) pyridine (Combi-Blocks; 1.2 equivalents), HATU (Combi-Blocks; 1.2 equivalents) and triethylamine (Sigma; 3 equivalents) at 0 °C. The resulting mixture was stirred at room temperature until all substrates were consumed. The reaction mixture was applied to reverse-phase HPLC chromatography using a Cosmosil column (Nacalai Tesque, 5C18 MS-II; 10 × 250 mm; flow rate of 4 ml min^−1^, acetonitrile in H_2_O with 0.1% trifluoroacetic acid) to yield 10.7 mg of CLIK-148 (52% total isolated yield).

To chemoenzymatically synthesize selective cathepsin C inhibitor E-64c hydrazide, a large-scale 40-ml reaction in 50 mM sodium phosphate buffer (pH 8.0) containing 5 mM (±)-*t*-ES, 2.5 mM l-norleucine (**a3**), 2.5 mM tryptamine (**b44**), 2.5 μM Cp1B and 2.5 μM Cp1D was carried out at 30 °C for 16 h. The removal of protein and the compound extraction were performed following the same procedure as mentioned above. Solvent was evaporated in vacuo to give a crude mixture. The crude mixture was then dissolved in DMF, before adding *tert*-butyl 1-butylhydrazine-1-carboxylate (Aaron Chemicals; 2 equivalents), HATU (Combi-Blocks; 2.4 equivalents) and triethylamine (Sigma; 10 equivalents) at 0 °C. The resulting mixture was stirred at room temperature overnight. The reaction mixture was applied to reverse-phase HPLC chromatography using a Cosmosil column (Nacalai Tesque, Cosmosil 3PBr; 10 × 250 mm; flow rate of 4 ml min^−1^, acetonitrile in H_2_O with 0.1% formic acid) to yield 20.0 mg of the Boc-protected E-64c hydrazide (36% isolated yield). Then, 20 μl of trifluoroacetic acid was added to the solution of 8.0 mg of the Boc-protected E-64c hydrazide in 200 μl of dichloromethane. The reaction mixture was stirred at room temperature. After 4 h, the solvent was evaporated in vacuo to give a crude mixture. The crude mixture was applied to reverse-phase HPLC chromatography using a Cosmosil column (Nacalai Tesque, 5C18-AR-II; 10 × 250 mm; flow rate of 4 ml min^−1^, acetonitrile in H_2_O with 0.1% trifluoroacetic acid) to yield 4.5 mg of the cathepsin C inhibitor, E-64c hydrazide (26% total isolated yield).

### Inhibition assay of cysteine cathepsin proteases

The cathepsin B inhibitor screening assay^57^ uses the ability of cathepsin B to cleave the synthetic AMC (7-amino-4-methylcoumarin)-based peptide substrate to release AMC, which can be quantified using a fluorometer or fluorescence microplate reader. In the presence of a cathepsin B inhibitor, the cleavage of the substrate is reduced or abolished, resulting in a decrease or total loss of the AMC fluorescence. Recombinant human procathepsin B (R&D systems) was activated to mature cathepsin B by incubation at 37 °C for 20 min in activation buffer (20 mM sodium acetate pH 5.5, 1 mM EDTA, 5 mM DTT and 100 mM NaCl). Cathepsin B activity was then assayed in final buffer conditions of 0.04 ng μl^−1^ cathepsin B, 40 μM *Z*-Phe-Arg-AMC (Sigma), 40 mM citrate phosphate (pH 5.5), 1 mM EDTA, 100 mM NaCl, 5 mM DTT and 0.01% Brij at 37 °C. Cleavage of *Z*-Phe-Arg-AMC to generate fluorescent AMC was monitored and the relative fluorescence units (RFU; excitation, 360 nm; emission, 460 nm) were recorded over a period of 30 min using an Infinite M200 PRO multimode microplate reader (Tecan). Two time points (*T*_1_ and *T*_2_) were chosen in the linear range of the plot to obtain the corresponding values for the fluorescence (RFU_1_ and RFU_2_). Slopes for inhibitor samples and enzyme control were calculated by dividing the net ΔRFU (RFU_2_ − RFU_1_) values by Δ*T* (*T*_2_ − *T*_1_). The percentage relative inhibition was calculated using the following equation (Eq. 1):

1 $$\% \,{\rm{Relative}}\,{\rm{inhibition}}=\frac{{\rm{Slope}}\,{\rm{of}}\,{\rm{enzyme}}\,{\rm{control}}-{{\rm{Slope}}\,{\rm{of}}\,{\rm{sample}}}}{{\rm{Slope}}\,{\rm{of}}\,{\rm{enzyme}}\,{\rm{control}}}\times 100$$

To test the effect of amino acid donor toward the inhibitory activity, *t*-ES-based compounds were obtained from coupled in vitro activity assays with 39 different amino acid donors while the amine donor was kept as agmatine. The typical assay contained 25 μM Cp1B, 25 μM Cp1D, 2 mM (±)-*t*-ES, 1 mM amino acid donor, 1 mM agmatine as the amine donor, 10 mM ATP and 10 mM MgCl_2_ in 100 μl of 50 mM sodium phosphate buffer (pH 8.0) at 30 °C for 16 h. The reaction was stopped by heat inactivation and centrifuged at 17,000*g* for 5 min. The supernatant was serially diluted and used (in theory, the final concentration of the corresponding E-64 analogs in the mixture was estimated to be less than 100 nM after serial dilution) for the cathepsin B inhibition assay.

To test the effect of amine donor toward the inhibitory activity, the *t*-ES-based compounds were obtained from coupled activity assays with 41 different amine donors while the amino acid used was **a10**. The assay contained 25 μM Cp1B, 25 μM Cp1D, 2 mM (±)-*t*-ES, 1 mM **a10**, 1 mM amine donor, 10 mM ATP and 10 mM MgCl_2_ in 100 μl of 50 mM sodium phosphate buffer (pH 8.0) at 30 °C for 16 h. The reaction was quenched and treated as mentioned above and the diluted sample was used for the cathepsin B inhibition assay.

Kinetic analyses of synthesized inhibitors of cathepsin B were conducted to determine IC_50_ values. The concentrations of selected compounds and E-64 ranged from 2,174 nM to 0.27 nM. IC_50_ values were calculated as the inhibitor concentration that reduced cathepsin B activity by 50%. Kinetic assays were performed in a 96-well plate format with three independent replicates. Data analysis was conducted using Prism GraphPad software.

The inhibitory potencies of synthesized inhibitors toward cathepsins L (R&D systems) and K (R&D systems) were assessed. Cathepsin L (0.03 ng μl^−1^) and cathepsin K (0.10 ng μl^−1^) activities were assayed with 40 μM *Z*-Phe-Arg-AMC. The fluorogenic assays followed the same protocol as described for cathepsin B.

### Crystallization of Cp1B, papain and papain–E-64 analog

#### Crystallization of Cp1B

The protein concentration used for crystallization was 15 mg ml^−1^ (0.26 mM). The protein was incubated with ATP (final concentration: 0.26 mM), MgCl_2_ (final concentration: 0.26 mM) and (±)-*t*-ES (final concentration: 1.3 mM) for 30 min on ice, corresponding to the ratio of 1:1:1:5. The sitting-drop vapor diffusion method was used for the initial screening at 22 °C. The protein (1 μl) was then combined with the reservoir solution (1 μl) in a ratio of 1:1. The total volume of protein mixture was 2 μl, which was equilibrated against 50 μl of reservoir solution. Commercially available screen reagents including Index (Hampton Research), Crystal Screen (Hampton Research), Grid Screen (Hampton Research), Morpheus (Molecular Dimensions), JCSG (Molecular Dimensions) and NeXtal (Molecular Dimensions) were used. The crystals were observed after 1 week in the condition of 0.1 M MES–imidazole pH 6.5, 10% w/v PEG 20000, 20% v/v PEG MME 550, 0.02 M sodium l-glutamate, 0.02 M dl-alanine, 0.02 M glycine, 0.02 M dl-lysine HCl and 0.02 M dl-serine. The crystallization solution contained cryoprotectant. We did not perform any additional cryoprotection of the crystals before flash freezing. The crystals were flash-cooled and stored in liquid nitrogen.

#### Crystallization of papain

Twice-crystallized papain from papaya latex was purchased from Sigma (P4762) as a buffered aqueous suspension approximately 25 mg ml^−1^ in protein concentration. Aliquots of this suspension were mixed with methanol, at a 1:2 volume ratio of papain suspension to methanol, in the sample wells of sitting-drop crystallization trays allowing up to 30 μl of sample per well. For all crystallization experiments, a total volume of 15 μl was targeted, although crystals were successfully grown in up to 30-μl volumes. These drops were incubated against a reservoir solution containing 59% methanol and 889 mM NaCl. The crystal used for determination of the unliganded papain structure was grown as above; all others were grown from seeds. For seeding, crystals were propagated by crushing up previously grown papain crystals by repeated pipetting in their mother liquor and transferring small fragments to freshly prepared sitting drops using a strand of horsehair. Papain crystals would typically appear in this condition between 48 and 72 h without seeding but formed in 24 h if seeded. All crystals adopted a prismatic, diamond-shaped morphology.

Papain was also cocrystallized with **1** and analogs using the same protocol as above, with the addition of the chosen inhibitor compound dissolved in solution to the crystallization well. E-64 was purchased from Sigma (E3132) and dissolved to 1.25 mg ml^−1^ (3.5 mM) in 66% methanol. E-64c and E-64d were each purchased from Selleck Chemicals (S7392 and S7393) and dissolved to 1.25 mg ml^−1^ (3.7 mM) in 66% methanol. Approximately 1 mg of purified amine (2*S*,3*S*)-*t*-ES-**a9**-**b7** was dissolved to 2.5 mg ml^−1^ (6.8 mM) in 66% methanol. For cocrystallization experiments with each compound, 2.4 μl of each compound solution was added to the crystallization well and mixed with protein solution. For these trials, the concentration of papain in each well was 0.35 mM, such that the estimated molar excess of inhibitor was 1.4× for **1**, 1.5× for E-64d and 2.7× for (2*S*,3*S*)-*t*-ES-**a9**-**b7**. For all cocrystallization experiments, crystals were seeded with fragments of unliganded papain crystals as described above and appeared after approximately 24 h. Crystal development in the presence of **1** or its analogs was inconsistent without seeding.

### Diffraction data collection

#### Cp1B structure

The crystals were mounted on Mitegen loops and flash-frozen under a 100-K nitrogen stream. The data were collected at beamline 17-ID-2 at the National Synchrotron Light Source II. Diffraction data were collected at the wavelength of 0.97933 Å. The detector distance was 250 mm. Data were collected over oscillations of 0.25° per exposure. Whole datasets of 1,440 frames were collected in 30 s.

#### Papain structures

Crystals were mounted on Mitegen loops and flash-frozen under a 100-K nitrogen stream. Full X-ray diffraction datasets were acquired using a Rigaku FRE+ rotating-anode X-ray diffractometer using a Cu Kα source (emitting X-ray photons 1.54 Å in wavelength) and equipped with a Rigaku HTC detector. All rotation datasets were collected taking 2-min integrated exposures over oscillations of 0.5° per exposure totaling approximately 26 h of data collection per crystal, at a detector distance of either 78 or 74 mm. This configuration enabled visualization of reflections up to 1.4 Å in resolution at the detector edge.

### Data processing, structure determination and refinement

#### Cp1B structure

Data frames in h5 file format were reduced in XDS and reflection intensities were scaled in XSCALE from the XDS package^74^. Data were converted to MTZ format using Pointless and resolution was determined by selecting the highest resolution shell where *I*/*σI* exceeded 2 using Aimless embedded in CCP4i2. The structure was solved by molecular replacement. The structure of Cp1B predicted using AlphaFold3 (ref. ^75^) (version 1; 10.5281/zenodo.14911266) was used as the search model by Phaser embedded in the PHENIX suite^76–78^. Refinement was performed using Refinement embedded in the PHENIX suite^76,77^. Briefly, Refinement was instructed to refine *XYZ* coordinates against both reciprocal space data and real-space maps, occupancies, individual *B* factors and translation, libration and screw parameters. No hydrogen atoms were modeled on any molecule. Three cycles of such refinement were performed before inspecting the agreement between the atomic coordinates and electron density map in Coot^79^ and building solvent molecules or adjusting side-chain positions to satisfy disagreements revealed in the difference Fourier map^79^. Water molecules were built manually and validated in Coot^79^. The statistics are summarized in Supplementary Table 4. The coordinates of the model were validated by Coot^79^. The images were drawn using PyMol.

#### Papain structures

Frames in OSC file format were reduced in XDS and reflection intensities were scaled in XSCALE and converted to MTZ format using XDSCONV^74,80^. Reflections extending to a resolution of 1.4–1.6 Å depending on the structure, determined by selecting for each dataset the highest resolution shell where completeness exceeded 90%, were included for integration, phasing and refinement. Phases were retrieved by molecular replacement using Phaser-MR through the PHENIX graphical user interface with a known X-ray diffraction structure of papain determined to 1.65-Å resolution (PDB 9PAP)^76–78,81^. For residue discrepancies between the protein sequence in this PDB entry and the sequence of papain reported in UniProt (P00784), the sequence from UniProt was adopted into the model for refinement.

Refinement of each structure was carried out in PHENIX. Briefly, PHENIX was instructed to refine *XYZ* coordinates against both reciprocal space data and real-space maps, occupancies and individual *B* factors. All protein and ligand atoms were treated as anisotropic in *B*-factor refinement if the structure was at least 1.4 Å in resolution, as for the papain–E-64d and papain–(2*S*,3*S*)-*t*-ES-**a9**-**b7** structures, although solvent atoms remained isotropic. For all other structures, protein and ligand atoms were considered isotropic in *B*-factor refinement. No hydrogen atoms were modeled on any molecule. Three cycles of such refinement were run before inspecting the agreement between the atomic coordinates and electron density map in Coot and building solvent molecules or adjusting side-chain positions to satisfy disagreements revealed in the difference Fourier map^79^. Water molecules were built in positions marked by positive-difference Fourier peaks exceeding 3*σ* levels where hydrogen-bonding partners were present within 2.5–3.3 Å. Methanol molecules were only built at sites where a water molecule did not fully abolish the difference Fourier density upon subsequent refinement cycles and where the carbon atom of the methanol molecule would not exist within 3.3 Å of any other nonbonded atoms. Following model adjustments, the coordinates were saved and the same refinement protocol in PHENIX was repeated.

For structures of papain complexed with **1** or its analogs, prominent positive-difference Fourier density indicating the presence of a peptidic molecule bound to the active Cys25 typically developed after one or two of these iterations. The respective ligand was modeled into this density using pre-existing monomers in the CCP4 library for **1**, E-64c and E-64d and a custom ligand built using Coot’s ligand builder for (2*S*,3*S*)-*t*-ES-**a9**-**b7** (ref. ^82^). Restraints for (2*S*,3*S*)-*t*-ES-**a9**-**b7** were generated using eLBOW in the PHENIX GUI, with the PDB file for the custom ligand modeled in Coot as input^76,77,79,83^. CIF files for each ligand were input as restraints for subsequent refinement of each respective complex structure. A bond length of 1.8 Å, with an allowed s.d. of 0.02 Å, was enforced for the covalent linkage between the sulfur atom of Cys25 and the ligand’s carbon C2 and the occupancy of all atoms in the ligand was refined in PHENIX as a single group^76,77^.

For the E-64d cocrystal structure only, we suspected that ester hydrolysis of this molecule might occur in the presence of polar solvents such as methanol or during binding of the epoxy warhead and we noted weak or absent density in all electron density maps corresponding to the possible leaving group. As such, the occupancies of C23 and C24 of E-64d were refined as a separate group from the rest of the ligand. As additional validation, ligand-omit maps were generated for each ligand-bound structure by deleting the ligand from the model and refining the original MTZ file naive to the presence of the ligand against it. Furthermore, composite omit 2*F*_o_ − *F*_c_ maps using simulated annealing were calculated in the PHENIX suite^76,77^ for each set of reflections against the corresponding final model to produce electron density maps less impacted by model bias^84^. For the E-64d cocrystal structure, where density in the 2*F*_o_ − *F*_c_ maps failed to cover all of the ligand’s aliphatic tail moieties, the pose of the ligand that was best accommodated by the density and gave the lowest *R*_free_ in refinement was selected and a feature-enhanced 2*F*_o_ − *F*_c_ map was calculated in PHENIX from the original reflections and the optimized model to better justify ligand placement^85^.

### Reporting summary

Further information on research design is available in the Nature Portfolio Reporting Summary linked to this article.

## Online content

Any methods, additional references, Nature Portfolio reporting summaries, source data, extended data, supplementary information, acknowledgements, peer review information; details of author contributions and competing interests; and statements of data and code availability are available at 10.1038/s41589-025-01907-2.

## Supplementary information

Supplementary Information Supplementary Methods, Tables 1–75 and Figs. 1–403.

Reporting Summary

## Source data

Source Data Extended Data Fig. 6 Statistical source data.

Source Data Extended Data Fig. 8 Statistical source data.

## Data Availability

The data that support the findings of this study are available within the paper and its Supplementary Information or from the corresponding authors upon request. The atomic coordinates of Cp1B with adenosine and MES, apo papain, papain with **1**, papain with E-64c, papain with E-64d and papain with (2*S*,3*S*)-*t*-ES-**a9**-**b7** were deposited to the PDB under accession codes 9CJN, 9CLH, 9CKT, 9EG7, 9CKW and 9CKY, respectively. The predicted structure of Cp1B (version 1), generated by AlphaFold3, is available from Zenodo (10.5281/zenodo.14911266)^86^. Source data are provided with this paper.
