# Supplementary material for: Enzymatic combinatorial synthesis of E-64 and related cysteine protease inhibitors
Source: Nat Chem Biol. 2025 May 9;21(11):1783–93. doi: 10.1038/s41589-025-01907-2 (PMC12568646; doi:10.1038/s41589-025-01907-2)
Supplement: Supplementary file 1 — Supplementary Methods, Tables 1–75 and Figs. 1–403. [file 41589_2025_1907_MOESM1_ESM.pdf]

nature chemical biology

Supplementary information https://doi.org/10.1038/s41589-025-01907-2

Enzymatic combinatorial synthesis of E-64 and related cysteine protease inhibitors

In the format provided by the authors and unedited

Table of Contents

Supplementary Methods
Chemicals and general spectroscopic analysis for synthetic compounds **11**
General molecular biology techniques **11**
Plasmid construction for heterologous expression in *A. nidulans* **11**
Preparation of protoplast of *A. nidulans* and transformation **12**
Synthesis of *N*-succinyl proteinogenic amino acids and (2*R*,3*R*)-*t*-ES-Ile **12**
Preparation of (2*S*,3*S*)-*t*-ES or (2*R*,3*R*)-*t*-ES standard **14**
Sequence information **15**
Isolated yield of *trans*-epoxysuccinyl amino acids by enzymatic synthesis with Cp1B or Cp2B **16**
Isolated yield of 15-b15, 15b-(18-20), 15-b24, 15-b27, 15-b(29-33), 15-b(37-38), and 15-b41 by enzymatic synthesis with Cp1D **17**
Spectroscopic data of E-64c, enzymatically synthesized with Cp1B and Cp1D **17**

Supplementary Tables
Table 1. Bioinformatic analysis of *cp1* gene cluster **18**
Table 2. Primers used in this study **19**
Table 3. Plasmids used in this study **21**
Table 4. X-ray data collection and refinement statistics of Cp1B **22**
Table 5. Statistics from crystallographic data reduction and refinement for papain with/without inhibitor **23**
Table 6. Spectroscopic data of compound **E-64 (1)** **24**
Table 7. Spectroscopic data of compound **CLIK-148 (3)** **25**
Table 8. Spectroscopic data of compound **CPI-2 (4)** **26**
Table 9. Spectroscopic data of compound **CPI-3 (5)** **27**
Table 10. Spectroscopic data of compound **6** **28**
Table 11. Spectroscopic data of compound **7** **29**
Table 12. Spectroscopic data of compound **8** **30**
Table 13. Spectroscopic data of compound **9** **31**
Table 14. Spectroscopic data of compound **10** **32**
Table 15. Spectroscopic data of compound **12** **33**
Table 16. Spectroscopic data of compound **13** **34**
Table 17. Spectroscopic data of compound **14** **35**
Table 18. Spectroscopic data of compound **15** **36**
Table 19. Spectroscopic data of compound (2*S*,3*S*)-***t*-ES-Leu** **37**
Table 20. Spectroscopic data of compound (2*S*,3*S*)-***t*-ES-Val** **38**
Table 21. Spectroscopic data of compound (2*S*,3*S*)-***t*-ES-Tyr** **39**
Table 22. Spectroscopic data of compound (2*S*,3*S*)-***t*-ES-Trp** **40**
Table 23. Spectroscopic data of compound (2*S*,3*S*)-***t*-ES-a1** **41**
Table 24. Spectroscopic data of compound (2*S*,3*S*)-***t*-ES-a2** **42**
Table 25. Spectroscopic data of compound (2*S*,3*S*)-***t*-ES-a3** **43**
Table 26. Spectroscopic data of compound (2*S*,3*S*)-***t*-ES-a4** **44**
Table 27. Spectroscopic data of compound (2*S*,3*S*)-***t*-ES-a5** **45**
Table 28. Spectroscopic data of compound (2*S*,3*S*)-***t*-ES-a6** **46**
Table 29. Spectroscopic data of compound (2*S*,3*S*)-***t*-ES-a7** **47**
Table 30. Spectroscopic data of compound (2*S*,3*S*)-***t*-ES-a8** **48**
Table 31. Spectroscopic data of compound (2*S*,3*S*)-***t*-ES-a9** **49**
Table 32. Spectroscopic data of compound (2*S*,3*S*)-***t*-ES-a10** **50**
Table 33. Spectroscopic data of compound (2*S*,3*S*)-***t*-ES-a11** **51**

Table 34. Spectroscopic data of compound (2*S*,3*S*)-***t*-ES-a12** **52**
Table 35. Spectroscopic data of compound (2*S*,3*S*)-***t*-ES-a13** **53**
Table 36. Spectroscopic data of compound (2*S*,3*S*)-***t*-ES-a14** **54**
Table 37. Spectroscopic data of compound (2*S*,3*S*)-***t*-ES-a15** **55**
Table 38. Spectroscopic data of compound (2*S*,3*S*)-***t*-ES-a16** **56**
Table 39. Spectroscopic data of compound (2*S*,3*S*)-***t*-ES-a17** **57**
Table 40. Spectroscopic data of compound (2*S*,3*S*)-***t*-ES-a18** **58**
Table 41. Spectroscopic data of compound (2*S*,3*S*)-***t*-ES-a19** **59**
Table 42. Spectroscopic data of compound (2*S*,3*S*)-***t*-ES-a20** **60**
Table 43. Spectroscopic data of compound (2*S*,3*S*)-***t*-ES-a21** **61**
Table 44. Spectroscopic data of compound (2*S*,3*S*)-***t*-ES-a22** **62**
Table 45. Spectroscopic data of compound (2*S*,3*S*)-***t*-ES-a23** **63**
Table 46. Spectroscopic data of compound (2*S*,3*S*)-***t*-ES-a24** **64**
Table 47. Spectroscopic data of compound (2*S*,3*S*)-***t*-ES-a25** **65**
Table 48. Spectroscopic data of compound (2*S*,3*S*)-***t*-ES-a26** **66**
Table 49. Spectroscopic data of compound (2*S*,3*S*)-***t*-ES-a27** **67**
Table 50. Spectroscopic data of compound (2*S*,3*S*)-***t*-ES-a28** **68**
Table 51. Spectroscopic data of compound (2*S*,3*S*)-***t*-ES-a29** **69**
Table 52. Spectroscopic data of compound (2*S*,3*S*)-***t*-ES-a30** **70**
Table 53. Spectroscopic data of compound (2*S*,3*S*)-***t*-ES-a31** **71**
Table 54. Spectroscopic data of compound (2*S*,3*S*)-***t*-ES-a32** **72**
Table 55. Spectroscopic data of compound (2*S*,3*S*)-***t*-ES-Phe-b15** **73**
Table 56. Spectroscopic data of compound (2*S*,3*S*)-***t*-ES-Phe-b18** **74**
Table 57. Spectroscopic data of compound (2*S*,3*S*)-***t*-ES-Phe-b19** **75**
Table 58. Spectroscopic data of compound (2*S*,3*S*)-***t*-ES-Phe-b20** **76**
Table 59. Spectroscopic data of compound (2*S*,3*S*)-***t*-ES-Phe-b24** **77**
Table 60. Spectroscopic data of compound (2*S*,3*S*)-***t*-ES-Phe-b27** **78**
Table 61. Spectroscopic data of compound (2*S*,3*S*)-***t*-ES-Phe-b29** **79**
Table 62. Spectroscopic data of compound (2*S*,3*S*)-***t*-ES-Phe-b30** **80**
Table 63. Spectroscopic data of compound (2*S*,3*S*)-***t*-ES-Phe-b31** **81**
Table 64. Spectroscopic data of compound (2*S*,3*S*)-***t*-ES-Phe-b32** **82**
Table 65. Spectroscopic data of compound (2*S*,3*S*)-***t*-ES-Phe-b33** **83**
Table 66. Spectroscopic data of compound (2*S*,3*S*)-***t*-ES-Phe-b37** **84**
Table 67. Spectroscopic data of compound (2*S*,3*S*)-***t*-ES-Phe-b38** **85**
Table 68. Spectroscopic data of compound (2*S*,3*S*)-***t*-ES-Phe-b41** **86**
Table 69. Spectroscopic data of compound (2*S*,3*S*)-***t*-ES-a9-b7** **87**
Table 70. Spectroscopic data of compound (2*S*,3*S*)-***t*-ES-a9-b13** **88**
Table 71. Spectroscopic data of compound (2*S*,3*S*)-***t*-ES-a10-b9** **89**
Table 72. Spectroscopic data of compound (2*S*,3*S*)-***t*-ES-a10-b14** **90**
Table 73. Spectroscopic data of compound (2*S*,3*S*)-***t*-ES-a10-b26** **91**
Table 74. Spectroscopic data of compound (2*S*,3*S*)-***t*-ES-Leu-b43** **92**
Table 75. Spectroscopic data of compound **E-64c-Hydrazide** **93**

Supplementary Figures

Figure 1. Natural and synthetic E-64 analogs **94**
Figure 2. Amide bond formation in fumaryl dipeptides and penilumamide by NRPS **95**
Figure 3. Structure and biosynthesis of warhead-armed bacterial pseudo tripeptides **96**
Figure 4. Bioinformatic analysis of Cp1D **97**
Figure 5. Bioinformatic analysis of Cp1B **98**
Figure 6. Plasmids used for heterologous expression **99**
Figure 7. SDS-PAGE gels of purified proteins used in this study **100**

Figure 8. Succinic acid can also be the substrate for Cp1A 101
Figure 9. D-amino acids are not substrate for Cp1B 102
Figure 10. Effect of in vitro enzymatic activity toward Cp1B or Cp1D in different buffers 103
Figure 11. Examination of the kinetic resolution potential for Cp1B 104
Figure 12. Previous work for the preparation of (2*S*, 3*S*)-*trans*-epoxy-succinic acid 105
Figure 13. Docking simulation of **15** with Cp1B and comparison of residues forming hydrophobic pocket between Cp1B and Cp2B 106
Figure 14. The comparison of nucleotide binding mode and surrounding residues between Cp1B and homoglutathione synthetase (PDB:3KAL). 107
Figure 15. SDS-PAGE gels of Cp1B mutants 108
Figure 16. HPLC traces of (2*S*,3*S*)-*t*-ES-amino acids formed by the Cp1B reaction 110
Figure 17. Structures of non-proteinogenic amino acids tested but could not be accepted by Cp1B/Cp2B 111
Figure 18. In vitro reaction of Cp1D with **14** and amines 112
Figure 19. Cp1D is a new family of diamide-forming amide bond synthetase not CoA ligase 113
Figure 20. Substrate scope test for Cp1D against coupling of isopentylamine with 20 synthesized *N*-succinyl proteinogenic amino acids 114
Figure 21. HPLC traces of combinatorial biocatalysis with agmatine and different L-amino acids 116
Figure 22. Structures of (pseudo)dipeptides tested for the acid acceptor of Cp1D 117
Figure 23. Non-E-64 biosynthetic gene clusters containing homologues of Cp1B and Cp1D 118
Figure 24. HPLC traces from the in vitro reaction of Cp1D with (2*S*,3*S*)-*t*-ES-L-Phe (**15**) and different amines 121
Figure 25. Structures of amine nucleophiles which could not be accepted by Cp1D or Cp2D 122
Figure 26. LC/MS analysis of one-pot synthesis E-64 analogs by Cp1B and Cp1D in 96-well plate 123
Figure 27. HPLC traces of combinatorial biocatalysis with **a10** and different amines 125
Figure 28. Reported chemical synthesis for E-64 (**1**) and CLIK-148 (**3**) 126
Figure 29. Preparative-scale synthesis of E-64c using ATP regeneration system 127
Figure 30. In vitro cathepsin B inhibition assay of enzymatically synthesized inhibitors 128
Figure 31. Inhibitory activity toward other cathepsin proteases 129
Figure 32. Irreversible inhibition of cathepsin B by **(2*S*,3*S*)-*t*-ES-a9-b7** 130
Figure 33. Dicarboxylic acids scope of Cp1B 131
Figure 34. In vitro assay of Cp2C 132
Figure 35. ^{1}H NMR spectrum of E-64 (**1**) in DMSO-d_6 132
Figure 36. ^{13}C NMR spectrum of E-64 (**1**) in DMSO-d_6 133
Figure 37. HSQC spectrum of E-64 (**1**) in DMSO-d_6 133
Figure 38. HMBC spectrum of E-64 (**1**) in DMSO-d_6 134
Figure 39. ^{1}H-^{1}H COSY spectrum of E-64 (**1**) in DMSO-d_6 134
Figure 40. ^{1}H NMR spectrum of E-64c (**2**) in DMSO-d_6 135
Figure 41. ^{13}C NMR spectrum of E-64c (**2**) in DMSO-d_6 135
Figure 42. ^{1}H NMR spectrum of CLIK148 (**3**) in $CDCl_3$ 136
Figure 43. ^{13}C NMR spectrum of CLIK148 (**3**) in $CDCl_3$ 136
Figure 44. HSQC spectrum of CLIK148 (**3**) in $CDCl_3$ 137
Figure 45. HMBC spectrum of CLIK148 (**3**) in $CDCl_3$ 137
Figure 46. ^{1}H-^{1}H COSY spectrum of CLIK148 (**3**) in $CDCl_3$ 138
Figure 47. ^{1}H NMR spectrum of CPI-2 (**4**) in DMSO-d_6 138
Figure 48. ^{13}C NMR spectrum of CPI-2 (**4**) in DMSO-d_6 139
Figure 49. HSQC spectrum of CPI-2 (**4**) in DMSO-d_6 139
Figure 50. HMBC spectrum of CPI-2 (**4**) in DMSO-d_6 140
Figure 51. ^{1}H-^{1}H COSY spectrum of CPI-2 (**4**) in DMSO-d_6 140
Figure 52. ^{1}H NMR spectrum of CPI-3 (**5**) in DMSO-d_6 141
Figure 53. ^{13}C NMR spectrum of CPI-3 (**5**) in DMSO-d_6 141

Figure 54. HSQC spectrum of CPI-3 (**5**) in DMSO-d_6 **142**
Figure 55. HMBC spectrum of CPI-3 (**5**) in DMSO-d_6 **142**
Figure 56. ^{1}H-^{1}H COSY spectrum of CPI-3 (**5**) in DMSO-d_6 **143**
Figure 57. ^{1}H NMR spectrum of compound **6** in DMSO-d_6 **143**
Figure 58. ^{13}C NMR spectrum of compound **6** in DMSO-d_6 **144**
Figure 59. HSQC spectrum of compound **6** in DMSO-d_6 **144**
Figure 60. HMBC spectrum of compound **6** in DMSO-d_6 **145**
Figure 61. ^{1}H-^{1}H COSY spectrum of compound **6** in DMSO-d_6 **145**
Figure 62. ^{1}H NMR spectrum of compound **7** in DMSO-d_6 **146**
Figure 63. ^{13}C NMR spectrum of compound **7** in DMSO-d_6 **146**
Figure 64. HSQC spectrum of compound **7** in DMSO-d_6 **147**
Figure 65. HMBC spectrum of compound **7** in DMSO-d_6 **147**
Figure 66. ^{1}H-^{1}H COSY spectrum of compound **7** in DMSO-d_6 **148**
Figure 67. ^{1}H NMR spectrum of compound **8** in DMSO-d_6 **148**
Figure 68. ^{13}C NMR spectrum of compound **8** in DMSO-d_6 **149**
Figure 69. HSQC spectrum of compound **8** in DMSO-d_6 **149**
Figure 70. HMBC spectrum of compound **8** in DMSO-d_6 **150**
Figure 71. ^{1}H-^{1}H COSY spectrum of compound **8** in DMSO-d_6 **150**
Figure 72. ^{1}H NMR spectrum of compound **9** in DMSO-d_6 **151**
Figure 73. ^{13}C NMR spectrum of compound **9** in DMSO-d_6 **151**
Figure 74. HSQC spectrum of compound **9** in DMSO-d_6 **152**
Figure 75. HMBC spectrum of compound **9** in DMSO-d_6 **152**
Figure 76. ^{1}H-^{1}H COSY spectrum of compound **9** in DMSO-d_6 **153**
Figure 77. ^{1}H NMR spectrum of compound **10** in DMSO-d_6 **153**
Figure 78. ^{13}C NMR spectrum of compound **10** in DMSO-d_6 **154**
Figure 79. HSQC spectrum of compound **10** in DMSO-d_6 **154**
Figure 80. HMBC spectrum of compound **10** in DMSO-d_6 **155**
Figure 81. ^{1}H-^{1}H COSY spectrum of compound **10** in DMSO-d_6 **155**
Figure 82. ^{1}H NMR spectrum of compound **12** in DMSO-d_6 **156**
Figure 83. ^{13}C NMR spectrum of compound **12** in DMSO-d_6 **156**
Figure 84. HSQC spectrum of compound **12** in DMSO-d_6 **157**
Figure 85. HMBC spectrum of compound **12** in DMSO-d_6 **157**
Figure 86. ^{1}H-^{1}H COSY spectrum of compound **12** in DMSO-d_6 **158**
Figure 87. ^{1}H NMR spectrum of compound **13** in DMSO-d_6 **158**
Figure 88. ^{13}C NMR spectrum of compound **13** in DMSO-d_6 **159**
Figure 89. HSQC spectrum of compound **13** in DMSO-d_6 **159**
Figure 90. HMBC spectrum of compound **13** in DMSO-d_6 **160**
Figure 91. ^{1}H-^{1}H COSY spectrum of compound **13** in DMSO-d_6 **160**
Figure 92. ^{1}H NMR spectrum of compound **14** in DMSO-d_6 **161**
Figure 93. ^{13}C NMR spectrum of compound **14** in DMSO-d_6 **161**
Figure 94. HSQC spectrum of compound **14** in DMSO-d_6 **162**
Figure 95. HMBC spectrum of compound **14** in DMSO-d_6 **162**
Figure 96. ^{1}H-^{1}H COSY spectrum of compound **14** in DMSO-d_6 **163**
Figure 97. ^{1}H NMR spectrum of compound **15** in DMSO-d_6 **163**
Figure 98. ^{13}C NMR spectrum of compound **15** in DMSO-d_6 **164**
Figure 99. HSQC spectrum of compound **15** in DMSO-d_6 **164**
Figure 100. HMBC spectrum of compound **15** in DMSO-d_6 **165**
Figure 101. ^{1}H-^{1}H COSY spectrum of compound **15** in DMSO-d_6 **165**
Figure 102. ^{1}H NMR spectrum of compound **(2*S*,3*S*)-*t*-ES-Leu** in DMSO-d_6 **166**
Figure 103. ^{13}C NMR spectrum of compound **(2*S*,3*S*)-*t*-ES-Leu** in DMSO-d_6 **166**
Figure 104. HSQC spectrum of compound **(2*S*,3*S*)-*t*-ES-Leu** in DMSO-d_6 **167**

Figure 105. HMBC spectrum of compound **(2*S*,3*S*)-*t*-ES-Leu** in DMSO-d_6 **167**
Figure 106. ^{1}H-^{1}H COSY spectrum of compound **(2*S*,3*S*)-*t*-ES-Leu** in DMSO-d_6 **168**
Figure 107. ^{1}H NMR spectrum of compound **(2*S*,3*S*)-*t*-ES-Val** in DMSO-d_6 **168**
Figure 108. ^{13}C NMR spectrum of compound **(2*S*,3*S*)-*t*-ES-Val** in DMSO-d_6 **169**
Figure 109. HSQC spectrum of compound **(2*S*,3*S*)-*t*-ES-Val** in DMSO-d_6 **169**
Figure 110. HMBC spectrum of compound **(2*S*,3*S*)-*t*-ES-Val** in DMSO-d_6 **170**
Figure 111. ^{1}H-^{1}H COSY spectrum of compound **(2*S*,3*S*)-*t*-ES-Val** in DMSO-d_6 **170**
Figure 112. ^{1}H NMR spectrum of compound **(2*S*,3*S*)-*t*-ES-Tyr** in DMSO-d_6 **171**
Figure 113. ^{13}C NMR spectrum of compound **(2*S*,3*S*)-*t*-ES-Tyr** in DMSO-d_6 **171**
Figure 114. HSQC spectrum of compound **(2*S*,3*S*)-*t*-ES-Tyr** in DMSO-d_6 **172**
Figure 115. HMBC spectrum of compound **(2*S*,3*S*)-*t*-ES-Tyr** in DMSO-d_6 **172**
Figure 116. ^{1}H-^{1}H COSY spectrum of compound **(2*S*,3*S*)-*t*-ES-Tyr** in DMSO-d_6 **173**
Figure 117. ^{1}H NMR spectrum of compound **(2*S*,3*S*)-*t*-ES-Trp** in DMSO-d_6 **173**
Figure 118. ^{13}C NMR spectrum of compound **(2*S*,3*S*)-*t*-ES-Trp** in DMSO-d_6 **174**
Figure 119. HSQC spectrum of compound **(2*S*,3*S*)-*t*-ES-Trp** in DMSO-d_6 **174**
Figure 120. HMBC spectrum of compound **(2*S*,3*S*)-*t*-ES-Trp** in DMSO-d_6 **175**
Figure 121. ^{1}H-^{1}H COSY spectrum of compound **(2*S*,3*S*)-*t*-ES-Trp** in DMSO-d_6 **175**
Figure 122. ^{1}H NMR spectrum of compound **(2*S*,3*S*)-*t*-ES-a1** in DMSO-d_6 **176**
Figure 123. ^{13}C NMR spectrum of compound **(2*S*,3*S*)-*t*-ES-a1** in DMSO-d_6 **176**
Figure 124. HSQC spectrum of compound **(2*S*,3*S*)-*t*-ES-a1** in DMSO-d_6 **177**
Figure 125. HMBC spectrum of compound **(2*S*,3*S*)-*t*-ES-a1** in DMSO-d_6 **177**
Figure 126. ^{1}H-^{1}H COSY spectrum of compound **(2*S*,3*S*)-*t*-ES-a1** in DMSO-d_6 **178**
Figure 127. ^{1}H NMR spectrum of compound **(2*S*,3*S*)-*t*-ES-a2** in DMSO-d_6 **178**
Figure 128. ^{13}C NMR spectrum of compound **(2*S*,3*S*)-*t*-ES-a2** in DMSO-d_6 **179**
Figure 129. HSQC spectrum of compound **(2*S*,3*S*)-*t*-ES-a2** in DMSO-d_6 **179**
Figure 130. HMBC spectrum of compound **(2*S*,3*S*)-*t*-ES-a2** in DMSO-d_6 **180**
Figure 131. ^{1}H-^{1}H COSY spectrum of compound **(2*S*,3*S*)-*t*-ES-a2** in DMSO-d_6 **180**
Figure 132. ^{1}H NMR spectrum of compound **(2*S*,3*S*)-*t*-ES-a3** in DMSO-d_6 **181**
Figure 133. ^{13}C NMR spectrum of compound **(2*S*,3*S*)-*t*-ES-a3** in DMSO-d_6 **181**
Figure 134. HSQC spectrum of compound **(2*S*,3*S*)-*t*-ES-a3** in DMSO-d_6 **182**
Figure 135. HMBC spectrum of compound **(2*S*,3*S*)-*t*-ES-a3** in DMSO-d_6 **182**
Figure 136. ^{1}H-^{1}H COSY spectrum of compound **(2*S*,3*S*)-*t*-ES-a3** in DMSO-d_6 **183**
Figure 137. ^{1}H NMR spectrum of compound **(2*S*,3*S*)-*t*-ES-a4** in DMSO-d_6 **183**
Figure 138. ^{13}C NMR spectrum of compound **(2*S*,3*S*)-*t*-ES-a4** in DMSO-d_6 **184**
Figure 139. HSQC spectrum of compound **(2*S*,3*S*)-*t*-ES-a4** in DMSO-d_6 **184**
Figure 140. HMBC spectrum of compound **(2*S*,3*S*)-*t*-ES-a4** in DMSO-d_6 **185**
Figure 141. ^{1}H-^{1}H COSY spectrum of compound **(2*S*,3*S*)-*t*-ES-a4** in DMSO-d_6 **185**
Figure 142. ^{1}H NMR spectrum of compound **(2*S*,3*S*)-*t*-ES-a5** in DMSO-d_6 **186**
Figure 143. ^{13}C NMR spectrum of compound **(2*S*,3*S*)-*t*-ES-a5** in DMSO-d_6 **186**
Figure 144. HSQC spectrum of compound **(2*S*,3*S*)-*t*-ES-a5** in DMSO-d_6 **187**
Figure 145. HMBC spectrum of compound **(2*S*,3*S*)-*t*-ES-a5** in DMSO-d_6 **187**
Figure 146. ^{1}H-^{1}H COSY spectrum of compound **(2*S*,3*S*)-*t*-ES-a5** in DMSO-d_6 **188**
Figure 147. ^{1}H NMR spectrum of compound **(2*S*,3*S*)-*t*-ES-a6** in DMSO-d_6 **188**
Figure 148. ^{13}C NMR spectrum of compound **(2*S*,3*S*)-*t*-ES-a6** in DMSO-d_6 **189**
Figure 149. HSQC spectrum of compound **(2*S*,3*S*)-*t*-ES-a6** in DMSO-d_6 **189**
Figure 150. HMBC spectrum of compound **(2*S*,3*S*)-*t*-ES-a6** in DMSO-d_6 **190**
Figure 151. ^{1}H-^{1}H COSY spectrum of compound **(2*S*,3*S*)-*t*-ES-a6** in DMSO-d_6 **190**
Figure 152. ^{1}H NMR spectrum of compound **(2*S*,3*S*)-*t*-ES-a7** in DMSO-d_6 **191**
Figure 153. ^{13}C NMR spectrum of compound **(2*S*,3*S*)-*t*-ES-a7** in DMSO-d_6 **191**
Figure 154. HSQC spectrum of compound **(2*S*,3*S*)-*t*-ES-a7** in DMSO-d_6 **192**
Figure 155. HMBC spectrum of compound **(2*S*,3*S*)-*t*-ES-a7** in DMSO-d_6 **192**

Figure 156. ^{1}H-^{1}H COSY spectrum of compound **(2*S*,3*S*)-*t*-ES-a7** in DMSO-d_6 **193**
Figure 157. ^{1}H NMR spectrum of compound **(2*S*,3*S*)-*t*-ES-a8** in DMSO-d_6 **193**
Figure 158. ^{13}C NMR spectrum of compound **(2*S*,3*S*)-*t*-ES-a8** in DMSO-d_6 **194**
Figure 159. HSQC spectrum of compound **(2*S*,3*S*)-*t*-ES-a8** in DMSO-d_6 **194**
Figure 160. HMBC spectrum of compound **(2*S*,3*S*)-*t*-ES-a8** in DMSO-d_6 **195**
Figure 161. ^{1}H-^{1}H COSY spectrum of compound **(2*S*,3*S*)-*t*-ES-a8** in DMSO-d_6 **195**
Figure 162. ^{1}H NMR spectrum of compound **(2*S*,3*S*)-*t*-ES-a9** in DMSO-d_6 **196**
Figure 163. ^{13}C NMR spectrum of compound **(2*S*,3*S*)-*t*-ES-a9** in DMSO-d_6 **196**
Figure 164. HSQC spectrum of compound **(2*S*,3*S*)-*t*-ES-a9** in DMSO-d_6 **197**
Figure 165. HMBC spectrum of compound **(2*S*,3*S*)-*t*-ES-a9** in DMSO-d_6 **197**
Figure 166. ^{1}H-^{1}H COSY spectrum of compound **(2*S*,3*S*)-*t*-ES-a9** in DMSO-d_6 **198**
Figure 167. ^{1}H NMR spectrum of compound **(2*S*,3*S*)-*t*-ES-a10** in DMSO-d_6 **198**
Figure 168. ^{13}C NMR spectrum of compound **(2*S*,3*S*)-*t*-ES-a10** in DMSO-d_6 **199**
Figure 169. HSQC spectrum of compound **(2*S*,3*S*)-*t*-ES-a10** in DMSO-d_6 **199**
Figure 170. HMBC spectrum of compound **(2*S*,3*S*)-*t*-ES-a10** in DMSO-d_6 **200**
Figure 171. ^{1}H-^{1}H COSY spectrum of compound **(2*S*,3*S*)-*t*-ES-a10** in DMSO-d_6 **200**
Figure 172. ^{1}H NMR spectrum of compound **(2*S*,3*S*)-*t*-ES-a11** in DMSO-d_6 **201**
Figure 173. ^{13}C NMR spectrum of compound **(2*S*,3*S*)-*t*-ES-a11** in DMSO-d_6 **201**
Figure 174. HSQC spectrum of compound **(2*S*,3*S*)-*t*-ES-a11** in DMSO-d_6 **202**
Figure 175. HMBC spectrum of compound **(2*S*,3*S*)-*t*-ES-a11** in DMSO-d_6 **202**
Figure 176. ^{1}H-^{1}H COSY spectrum of compound **(2*S*,3*S*)-*t*-ES-a11** in DMSO-d_6 **203**
Figure 177. ^{1}H NMR spectrum of compound **(2*S*,3*S*)-*t*-ES-a12** in DMSO-d_6 **203**
Figure 178. ^{13}C NMR spectrum of compound **(2*S*,3*S*)-*t*-ES-a12** in DMSO-d_6 **204**
Figure 179. HSQC spectrum of compound **(2*S*,3*S*)-*t*-ES-a12** in DMSO-d_6 **204**
Figure 180. HMBC spectrum of compound **(2*S*,3*S*)-*t*-ES-a12** in DMSO-d_6 **205**
Figure 181. ^{1}H-^{1}H COSY spectrum of compound **(2*S*,3*S*)-*t*-ES-a12** in DMSO-d_6 **205**
Figure 182. ^{1}H NMR spectrum of compound **(2*S*,3*S*)-*t*-ES-a13** in DMSO-d_6 **206**
Figure 183. ^{13}C NMR spectrum of compound **(2*S*,3*S*)-*t*-ES-a13** in DMSO-d_6 **206**
Figure 184. HSQC spectrum of compound **(2*S*,3*S*)-*t*-ES-a13** in DMSO-d_6 **207**
Figure 185. HMBC spectrum of compound **(2*S*,3*S*)-*t*-ES-a13** in DMSO-d_6 **207**
Figure 186. ^{1}H-^{1}H COSY spectrum of compound **(2*S*,3*S*)-*t*-ES-a13** in DMSO-d_6 **208**
Figure 187. ^{1}H NMR spectrum of compound **(2*S*,3*S*)-*t*-ES-a14** in DMSO-d_6 **208**
Figure 188. ^{13}C NMR spectrum of compound **(2*S*,3*S*)-*t*-ES-a14** in DMSO-d_6 **209**
Figure 189. HSQC spectrum of compound **(2*S*,3*S*)-*t*-ES-a14** in DMSO-d_6 **209**
Figure 190. HMBC spectrum of compound **(2*S*,3*S*)-*t*-ES-a14** in DMSO-d_6 **210**
Figure 191. ^{1}H-^{1}H COSY spectrum of compound **(2*S*,3*S*)-*t*-ES-a14** in DMSO-d_6 **210**
Figure 192. ^{1}H NMR spectrum of compound **(2*S*,3*S*)-*t*-ES-a15** in DMSO-d_6 **211**
Figure 193. ^{13}C NMR spectrum of compound **(2*S*,3*S*)-*t*-ES-a15** in DMSO-d_6 **211**
Figure 194. HSQC spectrum of compound **(2*S*,3*S*)-*t*-ES-a15** in DMSO-d_6 **212**
Figure 195. HMBC spectrum of compound **(2*S*,3*S*)-*t*-ES-a15** in DMSO-d_6 **212**
Figure 196. ^{1}H-^{1}H COSY spectrum of compound **(2*S*,3*S*)-*t*-ES-a15** in DMSO-d_6 **213**
Figure 197. ^{1}H NMR spectrum of compound **(2*S*,3*S*)-*t*-ES-a16** in DMSO-d_6 **213**
Figure 198. ^{13}C NMR spectrum of compound **(2*S*,3*S*)-*t*-ES-a16** in DMSO-d_6 **214**
Figure 199. HSQC spectrum of compound **(2*S*,3*S*)-*t*-ES-a16** in DMSO-d_6 **214**
Figure 200. HMBC spectrum of compound **(2*S*,3*S*)-*t*-ES-a16** in DMSO-d_6 **215**
Figure 201. ^{1}H-^{1}H COSY spectrum of compound **(2*S*,3*S*)-*t*-ES-a16** in DMSO-d_6 **215**
Figure 202. ^{1}H NMR spectrum of compound **(2*S*,3*S*)-*t*-ES-a16** in DMSO-d_6 **216**
Figure 203. ^{13}C NMR spectrum of compound **(2*S*,3*S*)-*t*-ES-a17** in DMSO-d_6 **216**
Figure 204. HSQC spectrum of compound **(2*S*,3*S*)-*t*-ES-a17** in DMSO-d_6 **217**
Figure 205. HMBC spectrum of compound **(2*S*,3*S*)-*t*-ES-a17** in DMSO-d_6 **217**
Figure 206. ^{1}H-^{1}H COSY spectrum of compound **(2*S*,3*S*)-*t*-ES-a17** in DMSO-d_6 **218**

Figure 207. ^{1}H NMR spectrum of compound **(2*S*,3*S*)-*t*-ES-a18** in DMSO-d_6 **218**
Figure 208. ^{13}C NMR spectrum of compound **(2*S*,3*S*)-*t*-ES-a18** in DMSO-d_6 **219**
Figure 209. HSQC spectrum of compound **(2*S*,3*S*)-*t*-ES-a18** in DMSO-d_6 **219**
Figure 210. HMBC spectrum of compound **(2*S*,3*S*)-*t*-ES-a18** in DMSO-d_6 **220**
Figure 211. ^{1}H-^{1}H COSY spectrum of compound **(2*S*,3*S*)-*t*-ES-a18** in DMSO-d_6 **220**
Figure 212. ^{1}H NMR spectrum of compound **(2*S*,3*S*)-*t*-ES-a19** in DMSO-d_6 **221**
Figure 213. ^{13}C NMR spectrum of compound **(2*S*,3*S*)-*t*-ES-a19** in DMSO-d_6 **221**
Figure 214. HSQC spectrum of compound **(2*S*,3*S*)-*t*-ES-a19** in DMSO-d_6 **222**
Figure 215. HMBC spectrum of compound **(2*S*,3*S*)-*t*-ES-a19** in DMSO-d_6 **222**
Figure 216. ^{1}H-^{1}H COSY spectrum of compound **(2*S*,3*S*)-*t*-ES-a19** in DMSO-d_6 **223**
Figure 217. ^{1}H NMR spectrum of compound **(2*S*,3*S*)-*t*-ES-a20** in DMSO-d_6 **223**
Figure 218. ^{13}C NMR spectrum of compound **(2*S*,3*S*)-*t*-ES-a20** in DMSO-d_6 **224**
Figure 219. HSQC spectrum of compound **(2*S*,3*S*)-*t*-ES-a20** in DMSO-d_6 **224**
Figure 220. HMBC spectrum of compound **(2*S*,3*S*)-*t*-ES-a20** in DMSO-d_6 **225**
Figure 221. ^{1}H-^{1}H COSY spectrum of compound **(2*S*,3*S*)-*t*-ES-a20** in DMSO-d_6 **225**
Figure 222. ^{1}H NMR spectrum of compound **(2*S*,3*S*)-*t*-ES-a21** in DMSO-d_6 **226**
Figure 223. ^{13}C NMR spectrum of compound **(2*S*,3*S*)-*t*-ES-a21** in DMSO-d_6 **226**
Figure 224. HSQC spectrum of compound **(2*S*,3*S*)-*t*-ES-a21** in DMSO-d_6 **227**
Figure 225. HMBC spectrum of compound **(2*S*,3*S*)-*t*-ES-a21** in DMSO-d_6 **227**
Figure 226. ^{1}H-^{1}H COSY spectrum of compound **(2*S*,3*S*)-*t*-ES-a21** in DMSO-d_6 **228**
Figure 227. ^{1}H NMR spectrum of compound **(2*S*,3*S*)-*t*-ES-a22** in DMSO-d_6 **228**
Figure 228. ^{13}C NMR spectrum of compound **(2*S*,3*S*)-*t*-ES-a22** in DMSO-d_6 **229**
Figure 229. HSQC spectrum of compound **(2*S*,3*S*)-*t*-ES-a22** in DMSO-d_6 **229**
Figure 230. HMBC spectrum of compound **(2*S*,3*S*)-*t*-ES-a22** in DMSO-d_6 **230**
Figure 231. ^{1}H-^{1}H COSY spectrum of compound **(2*S*,3*S*)-*t*-ES-a22** in DMSO-d_6 **230**
Figure 232. ^{1}H NMR spectrum of compound **(2*S*,3*S*)-*t*-ES-a23** in DMSO-d_6 **231**
Figure 233. ^{13}C NMR spectrum of compound **(2*S*,3*S*)-*t*-ES-a23** in DMSO-d_6 **231**
Figure 234. HSQC spectrum of compound **(2*S*,3*S*)-*t*-ES-a23** in DMSO-d_6 **232**
Figure 235. HMBC spectrum of compound **(2*S*,3*S*)-*t*-ES-a23** in DMSO-d_6 **232**
Figure 236. ^{1}H-^{1}H COSY spectrum of compound **(2*S*,3*S*)-*t*-ES-a23** in DMSO-d_6 **233**
Figure 237. ^{1}H NMR spectrum of compound **(2*S*,3*S*)-*t*-ES-a24** in DMSO-d_6 **233**
Figure 238. ^{13}C NMR spectrum of compound **(2*S*,3*S*)-*t*-ES-a24** in DMSO-d_6 **234**
Figure 239. HSQC spectrum of compound **(2*S*,3*S*)-*t*-ES-a24** in DMSO-d_6 **234**
Figure 240. HMBC spectrum of compound **(2*S*,3*S*)-*t*-ES-a24** in DMSO-d_6 **235**
Figure 241. ^{1}H-^{1}H COSY spectrum of compound **(2*S*,3*S*)-*t*-ES-a24** in DMSO-d_6 **235**
Figure 242. ^{1}H NMR spectrum of compound **(2*S*,3*S*)-*t*-ES-a25** in DMSO-d_6 **236**
Figure 243. ^{13}C NMR spectrum of compound **(2*S*,3*S*)-*t*-ES-a25** in DMSO-d_6 **236**
Figure 244. HSQC spectrum of compound **(2*S*,3*S*)-*t*-ES-a25** in DMSO-d_6 **237**
Figure 245. HMBC spectrum of compound **(2*S*,3*S*)-*t*-ES-a25** in DMSO-d_6 **237**
Figure 246. ^{1}H-^{1}H COSY spectrum of compound **(2*S*,3*S*)-*t*-ES-a25** in DMSO-d_6 **238**
Figure 247. ^{1}H NMR spectrum of compound **(2*S*,3*S*)-*t*-ES-a26** in DMSO-d_6 **238**
Figure 248. ^{13}C NMR spectrum of compound **(2*S*,3*S*)-*t*-ES-a26** in DMSO-d_6 **239**
Figure 249. HSQC spectrum of compound **(2*S*,3*S*)-*t*-ES-a26** in DMSO-d_6 **239**
Figure 250. HMBC spectrum of compound **(2*S*,3*S*)-*t*-ES-a26** in DMSO-d_6 **240**
Figure 251. ^{1}H-^{1}H COSY spectrum of compound **(2*S*,3*S*)-*t*-ES-a26** in DMSO-d_6 **240**
Figure 252. ^{1}H NMR spectrum of compound **(2*S*,3*S*)-*t*-ES-a27** in DMSO-d_6 **241**
Figure 253. ^{13}C NMR spectrum of compound **(2*S*,3*S*)-*t*-ES-a27** in DMSO-d_6 **241**
Figure 254. HSQC spectrum of compound **(2*S*,3*S*)-*t*-ES-a27** in DMSO-d_6 **242**
Figure 255. HMBC spectrum of compound **(2*S*,3*S*)-*t*-ES-a27** in DMSO-d_6 **242**
Figure 256. ^{1}H-^{1}H COSY spectrum of compound **(2*S*,3*S*)-*t*-ES-a27** in DMSO-d_6 **243**
Figure 257. ^{1}H NMR spectrum of compound **(2*S*,3*S*)-*t*-ES-a28** in DMSO-d_6 **243**

Figure 258. ^{13}C NMR spectrum of compound **(2*S*,3*S*)-*t*-ES-a28** in DMSO-d_6 **244**
Figure 259. HSQC spectrum of compound **(2*S*,3*S*)-*t*-ES-a28** in DMSO-d_6 **244**
Figure 260. HMBC spectrum of compound **(2*S*,3*S*)-*t*-ES-a28** in DMSO-d_6 **245**
Figure 261. ^{1}H-^{1}H COSY spectrum of compound **(2*S*,3*S*)-*t*-ES-a28** in DMSO-d_6 **245**
Figure 262. ^{1}H NMR spectrum of compound **(2*S*,3*S*)-*t*-ES-a29** in DMSO-d_6 **246**
Figure 263. ^{13}C NMR spectrum of compound **(2*S*,3*S*)-*t*-ES-a29** in DMSO-d_6 **246**
Figure 264. HSQC spectrum of compound **(2*S*,3*S*)-*t*-ES-a29** in DMSO-d_6 **247**
Figure 265. HMBC spectrum of compound **(2*S*,3*S*)-*t*-ES-a29** in DMSO-d_6 **247**
Figure 266. ^{1}H-^{1}H COSY spectrum of compound **(2*S*,3*S*)-*t*-ES-a29** in DMSO-d_6 **248**
Figure 267. ^{1}H NMR spectrum of compound **(2*S*,3*S*)-*t*-ES-a30** in DMSO-d_6 **248**
Figure 268. ^{1}H NMR spectrum of compound **(2*S*,3*S*)-*t*-ES-a30** in DMSO-d_6 **249**
Figure 269. HSQC spectrum of compound **(2*S*,3*S*)-*t*-ES-a30** in DMSO-d_6 **249**
Figure 270. HMBC spectrum of compound **(2*S*,3*S*)-*t*-ES-a30** in DMSO-d_6 **250**
Figure 271. ^{1}H-^{1}H COSY spectrum of compound **(2*S*,3*S*)-*t*-ES-a30** in DMSO-d_6 **250**
Figure 272. ^{1}H NMR spectrum of compound **(2*S*,3*S*)-*t*-ES-a31** in DMSO-d_6 **251**
Figure 273. ^{13}C NMR spectrum of compound **(2*S*,3*S*)-*t*-ES-a31** in DMSO-d_6 **251**
Figure 274. HSQC spectrum of compound **(2*S*,3*S*)-*t*-ES-a31** in DMSO-d_6 **252**
Figure 275. HMBC spectrum of compound **(2*S*,3*S*)-*t*-ES-a31** in DMSO-d_6 **252**
Figure 276. ^{1}H-^{1}H COSY spectrum of compound **(2*S*,3*S*)-*t*-ES-a31** in DMSO-d_6 **253**
Figure 277. ^{1}H NMR spectrum of compound **(2*S*,3*S*)-*t*-ES-a32** in DMSO-d_6 **253**
Figure 278. ^{13}C NMR spectrum of compound **(2*S*,3*S*)-*t*-ES-a32** in DMSO-d_6 **254**
Figure 279. HSQC spectrum of compound **(2*S*,3*S*)-*t*-ES-a32** in DMSO-d_6 **254**
Figure 280. HMBC spectrum of compound **(2*S*,3*S*)-*t*-ES-a32** in DMSO-d_6 **255**
Figure 281. ^{1}H-^{1}H COSY spectrum of compound **(2*S*,3*S*)-*t*-ES-a32** in DMSO-d_6 **255**
Figure 282. ^{1}H NMR spectrum of compound **(2*S*,3*S*)-*t*-ES-Phe-b15** in DMSO-d_6 **256**
Figure 283. ^{13}C NMR spectrum of compound **(2*S*,3*S*)-*t*-ES-Phe-b15** in DMSO-d_6 **256**
Figure 284. HSQC spectrum of compound **(2*S*,3*S*)-*t*-ES-Phe-b15** in DMSO-d_6 **257**
Figure 285. HMBC spectrum of compound **(2*S*,3*S*)-*t*-ES-Phe-b15** in DMSO-d_6 **257**
Figure 286. ^{1}H-^{1}H COSY spectrum of compound **(2*S*,3*S*)-*t*-ES-Phe-b15** in DMSO-d_6 **258**
Figure 287. ^{1}H NMR spectrum of compound **(2*S*,3*S*)-*t*-ES-Phe-b18** in DMSO-d_6 **258**
Figure 288. ^{13}C NMR spectrum of compound **(2*S*,3*S*)-*t*-ES-Phe-b18** in DMSO-d_6 **259**
Figure 289. HSQC spectrum of compound **(2*S*,3*S*)-*t*-ES-Phe-b18** in DMSO-d_6 **259**
Figure 290. HMBC spectrum of compound **(2*S*,3*S*)-*t*-ES-Phe-b18** in DMSO-d_6 **260**
Figure 291. ^{1}H-^{1}H COSY spectrum of compound **(2*S*,3*S*)-*t*-ES-Phe-b18** in DMSO-d_6 **260**
Figure 292. ^{1}H NMR spectrum of compound **(2*S*,3*S*)-*t*-ES-Phe-b19** in DMSO-d_6 **261**
Figure 293. ^{13}C NMR spectrum of compound **(2*S*,3*S*)-*t*-ES-Phe-b19** in DMSO-d_6 **261**
Figure 294. HSQC spectrum of compound **(2*S*,3*S*)-*t*-ES-Phe-b19** in DMSO-d_6 **262**
Figure 295. HMBC spectrum of compound **(2*S*,3*S*)-*t*-ES-Phe-b19** in DMSO-d_6 **262**
Figure 296. ^{1}H-^{1}H COSY spectrum of compound **(2*S*,3*S*)-*t*-ES-Phe-b19** in DMSO-d_6 **263**
Figure 297. ^{1}H NMR spectrum of compound **(2*S*,3*S*)-*t*-ES-Phe-b20** in DMSO-d_6 **263**
Figure 298. ^{13}C NMR spectrum of compound **(2*S*,3*S*)-*t*-ES-Phe-b20** in DMSO-d_6 **264**
Figure 299. HSQC spectrum of compound **(2*S*,3*S*)-*t*-ES-Phe-b20** in DMSO-d_6 **264**
Figure 300. HMBC spectrum of compound **(2*S*,3*S*)-*t*-ES-Phe-b20** in DMSO-d_6 **265**
Figure 301. ^{1}H-^{1}H COSY spectrum of compound **(2*S*,3*S*)-*t*-ES-Phe-b20** in DMSO-d_6 **265**
Figure 302. ^{1}H NMR spectrum of compound **(2*S*,3*S*)-*t*-ES-Phe-b24** in DMSO-d_6 **266**
Figure 303. ^{13}C NMR spectrum of compound **(2*S*,3*S*)-*t*-ES-Phe-b24** in DMSO-d_6 **266**
Figure 304. HSQC spectrum of compound **(2*S*,3*S*)-*t*-ES-Phe-b24** in DMSO-d_6 **267**
Figure 305. HMBC spectrum of compound **(2*S*,3*S*)-*t*-ES-Phe-b24** in DMSO-d_6 **267**
Figure 306. ^{1}H-^{1}H COSY spectrum of compound **(2*S*,3*S*)-*t*-ES-Phe-b24** in DMSO-d_6 **268**
Figure 307. ^{1}H NMR spectrum of compound **(2*S*,3*S*)-*t*-ES-Phe-b27** in DMSO-d_6 **268**
Figure 308. ^{13}C NMR spectrum of compound **(2*S*,3*S*)-*t*-ES-Phe-b27** in DMSO-d_6 **269**

Figure 309. HSQC spectrum of compound **(2*S*,3*S*)-*t*-ES-Phe-b27** in DMSO-d_6 **269**
Figure 310. HMBC spectrum of compound **(2*S*,3*S*)-*t*-ES-Phe-b27** in DMSO-d_6 **270**
Figure 311. ^{1}H-^{1}H COSY spectrum of compound **(2*S*,3*S*)-*t*-ES-Phe-b27** in DMSO-d_6 **270**
Figure 312. ^{1}H NMR spectrum of compound **(2*S*,3*S*)-*t*-ES-Phe-b29** in DMSO-d_6 **271**
Figure 313. ^{13}C NMR spectrum of compound **(2*S*,3*S*)-*t*-ES-Phe-b29** in DMSO-d_6 **271**
Figure 314. HSQC spectrum of compound **(2*S*,3*S*)-*t*-ES-Phe-b29** in DMSO-d_6 **272**
Figure 315. HMBC spectrum of compound **(2*S*,3*S*)-*t*-ES-Phe-b29** in DMSO-d_6 **272**
Figure 316. ^{1}H-^{1}H COSY spectrum of compound **(2*S*,3*S*)-*t*-ES-Phe-b29** in DMSO-d_6 **273**
Figure 317. ^{1}H NMR spectrum of compound **(2*S*,3*S*)-*t*-ES-Phe-b30** in DMSO-d_6 **273**
Figure 318. ^{13}C NMR spectrum of compound **(2*S*,3*S*)-*t*-ES-Phe-b30** in DMSO-d_6 **274**
Figure 319. HSQC spectrum of compound **(2*S*,3*S*)-*t*-ES-Phe-b30** in DMSO-d_6 **274**
Figure 320. HMBC spectrum of compound **(2*S*,3*S*)-*t*-ES-Phe-b30** in DMSO-d_6 **275**
Figure 321. ^{1}H-^{1}H COSY spectrum of compound **(2*S*,3*S*)-*t*-ES-Phe-b30** in DMSO-d_6 **275**
Figure 322. ^{1}H NMR spectrum of compound **(2*S*,3*S*)-*t*-ES-Phe-b31** in DMSO-d_6 **276**
Figure 323. ^{13}C NMR spectrum of compound **(2*S*,3*S*)-*t*-ES-Phe-b31** in DMSO-d_6 **276**
Figure 324. HSQC spectrum of compound **(2*S*,3*S*)-*t*-ES-Phe-b31** in DMSO-d_6 **277**
Figure 325. HMBC spectrum of compound **(2*S*,3*S*)-*t*-ES-Phe-b31** in DMSO-d_6 **277**
Figure 326. ^{1}H-^{1}H COSY spectrum of compound **(2*S*,3*S*)-*t*-ES-Phe-b31** in DMSO-d_6 **278**
Figure 327. ^{1}H NMR spectrum of compound **(2*S*,3*S*)-*t*-ES-Phe-b32** in DMSO-d_6 **278**
Figure 328. ^{13}C NMR spectrum of compound **(2*S*,3*S*)-*t*-ES-Phe-b32** in DMSO-d_6 **279**
Figure 329. HSQC spectrum of compound **(2*S*,3*S*)-*t*-ES-Phe-b32** in DMSO-d_6 **279**
Figure 330. HMBC spectrum of compound **(2*S*,3*S*)-*t*-ES-Phe-b32** in DMSO-d_6 **280**
Figure 331. ^{1}H-^{1}H COSY spectrum of compound **(2*S*,3*S*)-*t*-ES-Phe-b32** in DMSO-d_6 **280**
Figure 332. ^{1}H NMR spectrum of compound **(2*S*,3*S*)-*t*-ES-Phe-b33** in DMSO-d_6 **281**
Figure 333. ^{13}C NMR spectrum of compound **(2*S*,3*S*)-*t*-ES-Phe-b33** in DMSO-d_6 **281**
Figure 334. HSQC spectrum of compound **(2*S*,3*S*)-*t*-ES-Phe-b33** in DMSO-d_6 **282**
Figure 335. HMBC spectrum of compound **(2*S*,3*S*)-*t*-ES-Phe-b33** in DMSO-d_6 **282**
Figure 336. ^{1}H-^{1}H COSY spectrum of compound **(2*S*,3*S*)-*t*-ES-Phe-b33** in DMSO-d_6 **283**
Figure 337. ^{1}H NMR spectrum of compound **(2*S*,3*S*)-*t*-ES-Phe-b37** in DMSO-d_6 **283**
Figure 338. ^{13}C NMR spectrum of compound **(2*S*,3*S*)-*t*-ES-Phe-b37** in DMSO-d_6 **284**
Figure 339. HSQC spectrum of compound **(2*S*,3*S*)-*t*-ES-Phe-b37** in DMSO-d_6 **284**
Figure 340. HMBC spectrum of compound **(2*S*,3*S*)-*t*-ES-Phe-b37** in DMSO-d_6 **285**
Figure 341. ^{1}H-^{1}H COSY spectrum of compound **(2*S*,3*S*)-*t*-ES-Phe-b37** in DMSO-d_6 **285**
Figure 342. ^{1}H NMR spectrum of compound **(2*S*,3*S*)-*t*-ES-Phe-b38** in DMSO-d_6 **286**
Figure 343. ^{13}C NMR spectrum of compound **(2*S*,3*S*)-*t*-ES-Phe-b38** in DMSO-d_6 **286**
Figure 344. HSQC spectrum of compound **(2*S*,3*S*)-*t*-ES-Phe-b38** in DMSO-d_6 **287**
Figure 345. HMBC spectrum of compound **(2*S*,3*S*)-*t*-ES-Phe-b38** in DMSO-d_6 **287**
Figure 346. ^{1}H-^{1}H COSY spectrum of compound **(2*S*,3*S*)-*t*-ES-Phe-b38** in DMSO-d_6 **288**
Figure 347. ^{1}H NMR spectrum of compound **(2*S*,3*S*)-*t*-ES-Phe-b41** in DMSO-d_6 **288**
Figure 348. ^{13}C NMR spectrum of compound **(2*S*,3*S*)-*t*-ES-Phe-b41** in DMSO-d_6 **289**
Figure 349. HSQC spectrum of compound **(2*S*,3*S*)-*t*-ES-Phe-b41** in DMSO-d_6 **289**
Figure 350. HMBC spectrum of compound **(2*S*,3*S*)-*t*-ES-Phe-b41** in DMSO-d_6 **290**
Figure 351. ^{1}H-^{1}H COSY spectrum of compound **(2*S*,3*S*)-*t*-ES-Phe-b41** in DMSO-d_6 **290**
Figure 352. ^{1}H NMR spectrum of compound **(2*S*,3*S*)-*t*-ES-a9-b7** in DMSO-d_6 **291**
Figure 353. ^{13}C NMR spectrum of compound **(2*S*,3*S*)-*t*-ES-a9-b7** in DMSO-d_6 **291**
Figure 354. HSQC spectrum of compound **(2*S*,3*S*)-*t*-ES-a9-b7** in DMSO-d_6 **292**
Figure 355. HMBC spectrum of compound **(2*S*,3*S*)-*t*-ES-a9-b7** in DMSO-d_6 **292**
Figure 356. ^{1}H-^{1}H COSY spectrum of compound **(2*S*,3*S*)-*t*-ES-a9-b7** in DMSO-d_6 **293**
Figure 357. ^{1}H NMR spectrum of compound **(2*S*,3*S*)-*t*-ES-a9-b13** in DMSO-d_6 **293**
Figure 358. ^{13}C NMR spectrum of compound **(2*S*,3*S*)-*t*-ES-a9-b13** in DMSO-d_6 **294**
Figure 359. HSQC spectrum of compound **(2*S*,3*S*)-*t*-ES-a9-b13** in DMSO-d_6 **294**

Figure 360. HMBC spectrum of compound **(2*S*,3*S*)-*t*-ES-a9-b13** in DMSO-d_6 **295**
Figure 361. ^{1}H-^{1}H COSY spectrum of compound **(2*S*,3*S*)-*t*-ES-a9-b13** in DMSO-d_6 **295**
Figure 362. ^{1}H NMR spectrum of compound **(2*S*,3*S*)-*t*-ES-a10-b9** in DMSO-d_6 **296**
Figure 363. ^{13}C NMR spectrum of compound **(2*S*,3*S*)-*t*-ES-a10-b9** in DMSO-d_6 **296**
Figure 364. HSQC spectrum of compound **(2*S*,3*S*)-*t*-ES-a10-b9** in DMSO-d_6 **297**
Figure 365. HMBC spectrum of compound **(2*S*,3*S*)-*t*-ES-a10-b9** in DMSO-d_6 **297**
Figure 366. ^{1}H-^{1}H COSY spectrum of compound **(2*S*,3*S*)-*t*-ES-a10-b9** in DMSO-d_6 **298**
Figure 367. ^{1}H NMR spectrum of compound **(2*S*,3*S*)-*t*-ES-a10-b14** in acetone-d_6 **298**
Figure 368. ^{13}C NMR spectrum of compound **(2*S*,3*S*)-*t*-ES-a10-b14** in acetone-d_6 **299**
Figure 369. HSQC spectrum of compound **(2*S*,3*S*)-*t*-ES-a10-b14** in acetone-d_6 **299**
Figure 370. HMBC spectrum of compound **(2*S*,3*S*)-*t*-ES-a10-b14** in acetone-d_6 **300**
Figure 371. ^{1}H-^{1}H COSY spectrum of compound **(2*S*,3*S*)-*t*-ES-a10-b14** in acetone-d_6 **300**
Figure 372. ^{1}H NMR spectrum of compound **(2*S*,3*S*)-*t*-ES-a10-b26** in acetone-d_6 **301**
Figure 373. ^{13}C NMR spectrum of compound **(2*S*,3*S*)-*t*-ES-a10-b26** in acetone-d_6 **301**
Figure 374. HSQC spectrum of compound **(2*S*,3*S*)-*t*-ES-a10-b26** in acetone-d_6 **302**
Figure 375. HMBC spectrum of compound **(2*S*,3*S*)-*t*-ES-a10-b26** in acetone-d_6 **302**
Figure 376. ^{1}H-^{1}H COSY spectrum of compound **(2*S*,3*S*)-*t*-ES-a10-b26** in acetone-d_6 **303**
Figure 377. ^{1}H NMR spectrum of compound **(2*S*,3*S*)-*t*-ES-Leu-b43** in DMSO-d_6 **303**
Figure 378. ^{13}C NMR spectrum of compound **(2*S*,3*S*)-*t*-ES-Leu-b43** in DMSO-d_6 **304**
Figure 379. HSQC spectrum of compound **(2*S*,3*S*)-*t*-ES-Leu-b43** in DMSO-d_6 **304**
Figure 380. HMBC spectrum of compound **(2*S*,3*S*)-*t*-ES-Leu-b43** in DMSO-d_6 **305**
Figure 381. ^{1}H-^{1}H COSY spectrum of compound **(2*S*,3*S*)-*t*-ES-Leu-b43** in DMSO-d_6 **305**
Figure 382. ^{1}H NMR spectrum of compound **E-64c-Hydrazide** in DMSO-d_6 **306**
Figure 383. ^{13}C NMR spectrum of compound **E-64c-Hydrazide** in DMSO-d_6 **306**
Figure 384. HSQC spectrum of compound **E-64c-Hydrazide** in DMSO-d_6 **307**
Figure 385. HMBC spectrum of compound **E-64c-Hydrazide** in DMSO-d_6 **307**
Figure 386. ^{1}H-^{1}H COSY spectrum of compound **E-64c-Hydrazide** in DMSO-d_6 **308**
Figure 387. ^{1}H NMR spectrum of compound ***N*-succinyl-L-alanine** in DMSO-d_6 **308**
Figure 388. ^{13}C NMR spectrum of compound ***N*-succinyl-L-alanine** in DMSO-d_6 **309**
Figure 389. ^{1}H NMR spectrum of compound ***N*-succinyl-L-valine** in CD_3OD **309**
Figure 390. ^{13}C NMR spectrum of compound ***N*-succinyl-L-valine** in CD_3OD **310**
Figure 391. ^{1}H NMR spectrum of compound ***N*-succinyl-L-leucine** in D_2O **310**
Figure 392. ^{13}C NMR spectrum of compound ***N*-succinyl-L-leucine** in D_2O **311**
Figure 393. ^{1}H NMR spectrum of compound ***N*-succinyl-L-isoleucine** in CD_3OD **311**
Figure 394. ^{13}C NMR spectrum of compound ***N*-succinyl-L-isoleucine** in CD_3OD **312**
Figure 395. ^{1}H NMR spectrum of compound ***N*-succinyl-L-methionine** in DMSO-d_6 **312**
Figure 396. ^{13}C NMR spectrum of compound ***N*-succinyl-L-methionine** in DMSO-d_6 **313**
Figure 397. ^{1}H NMR spectrum of compound ***N*-succinyl-L-proline** in DMSO-d_6 **313**
Figure 398. ^{1}H NMR spectrum of compound ***N*-succinyl-L-phenylalanine** in CD_3OD **314**
Figure 399. ^{13}C NMR spectrum of compound ***N*-succinyl-L-phenylalanine** in CD_3OD **314**
Figure 400. ^{1}H NMR spectrum of compound ***N*-succinyl-L-tyrosine** in DMSO-d_6 **315**
Figure 401. ^{13}C NMR spectrum of compound ***N*-succinyl-L-tyrosine** in DMSO-d_6 **315**
Figure 402. ^{1}H NMR spectrum of compound ***N*-succinyl-L-tryptophan** in DMSO-d_6 **316**
Figure 403. ^{13}C NMR spectrum of compound ***N*-succinyl-L-tryptophan** in DMSO-d_6 **317**

Supplementary References **317**

Uncropped SDS−PAGE **320**

Supplementary Methods

Chemicals and general spectroscopic analysis for synthetic compounds

N-succinyl-Gly and 20 proteinogenic acids were purchased from Sigma-Aldrich. Succinic anhydride was purchased from Combi-Blocks. (2*S*,3*S*)-diethyl-2,3-epoxysuccinate and (2*R*,3*R*)-diethyl-2,3-epoxysuccinate were purchased from Enamine. NaOH was purchased from Fisher Chemical. Non-proteinogenic acids and amines used for enzymatic synthesis were purchased from Combi-Blocks, Enamine, and AA Blocks. NMR spectra were obtained with a Bruker AV500 spectrometer with a 5 mm dual cryoprobe at the UCLA Molecular Instrumentation Center. (^{1}HNMR 500 MHz, ^{13}CNMR 125 MHz). High resolution mass spectra were also recorded on a Agilent 6545 Quadrupole Time of Flight high resolution mass spectrometer (UCLA Molecular Instrumentation Center). The mass and NMR spectra were analysed by MassHunter 10.0 (Agilent) and MestReNova-9.0.1 (Mestrelab Research), respectively.

Strains and culture conditions

Aspergillus flavus NRRL3357 was obtained from the Agricultural Research Service Culture Collection (NRRL). It was maintained in liquid PDB medium (PDA medium without agar) at 28 °C for isolation of genomic DNA. *Aspergillus nidulans* A1145 ΔEMΔST host was previously developed in our lab[1]. The *A. nidulans* strain was grown at 28 °C in CD media (1 L: 10 g Glucose, 50 mL 20 × Nitrate salts, 1 mL Trace elements, pH 6.5, and 20 g/L Agar for solid cultivation) for sporulation or in CD-ST media (1L: 20 g Starch, 20 g Casamino acids, 50 mL 20 × Nitrate salts, 1 mL Trace elements, pH 6.5)[1] for heterologous expression of gene clusters, compound production and RNA extraction. *Escherichia coli* strains were cultivated either on lysogeny broth (LB) agar plates or in LB liquid medium. Growth media were supplied with antibiotics as required at the following concentrations: kanamycin (50 μg mL^{-1}), and ampicillin (100 μg mL^{-1}). *Saccharomyces cerevisiae* strain BJ5464-NpgA (*MATα ura3-52 his3-Δ200 leu2-Δ1 trp1 pep4::HIS3 prb1 Δ1.6R can1 GAL*) was used as the yeast host for *in vivo* homologous recombination to construct the *A. nidulans* plasmids.

General molecular biology techniques

E. coli TOP10 cells were used for cloning, following standard recombinant DNA techniques. *E. coli* BL21(DE3) (Novagen) was used as the *E. coli* host for protein expression. DNA restriction enzymes were used as recommended by the manufacturer (New England Biolabs, NEB). Genomic DNA from all fungal strains was prepared using LETS isolation buffer (10 mM Tris-HCI, pH 8.0, 20 mM EDTA, 0.5% SDS, 0.1 M LiCl). PCR was performed using Q5 High-Fidelity DNA Polymerase (NEB). The gene-specific primers are listed in Supplementary Table 2. PCR products were confirmed by DNA sequencing. For isolation of RNA from *A. nidulans* transformants, the strains were grown on CD-ST liquid for 3 days at 28 °C. The RNA extraction steps were performed using RiboPure™ Yeast RNA Isolation Kit (Ambion) following the manufacturer's instructions. Residual genomic DNA in the extracts was digested by DNase I (2 U/mL) (Invitrogen) at 37 °C for 4 hours. SuperScript III First-Strand Synthesis System (Invitrogen) was used for cDNA synthesis with Oligo-dT primers following directions from the user manual.

Plasmid construction for heterologous expression in *A. nidulans*

For heterologous expression in *A. nidulans*, three plasmid vectors, pYTU, pYTP, and pYTR containing

auxotrophic markers for uracil (*pyrG*), pyridoxine (*pyroA*), and riboflavin (*riboB*), respectively, were used to construct plasmids for *A. nidulans* heterologous expression. Genes in the *cp1* and *cp2* cluster were amplified with PCR from the genomic DNA of *A. flavus*. The *gpdA* promoters from *Penicillium oxalicum (*constitutive *POgpdA), A. niger* (constitutive *gpdA*, *glaA* induced by starch) and *Penicillium expansum (*constitutive *PEgpdA)* were amplified by PCR. pYTP and pYTR were digested with PacI/PspXI. pYTU was digested with PacI/NotI. The amplified gene fragments and the corresponding vectors were co-transformed into *S. cerevisiae* strain BJ5464-NpgA for homologous recombination. The yeast plasmids were extracted using Zymoprep^{TM} Yeast Plasmid Miniprep I (Zymo Inc. USA), and then electrically transformed into *E. coli* TOP10 to isolate single plasmids. The plasmids were extracted from *E. coli* using the Zyppy^{TM} Plasmid Miniprep Kit (Zymo Research) and confirmed with sequencing by Laragen and Primordium Lab.

Preparation of protoplast of *A. nidulans* and transformation

The transformation of *Aspergillus nidulans* A1145 ΔEMΔST[1], spores were inoculated into 50 mL liquid CD media in a 125-mL flask and germinated at 30 °C shaking at 250 rpm for ~9 h. The 20 X Nitrate salts solution was prepared by dissolving 120 g $NaNO_3$, 10.4 g KCl, 10.4 g $MgSO_4$•$7H_2O$, and 30.4 g KH_2PO_4 in 1 L double distilled water. The trace elements solution (100 mL) contained 2.20 g $ZnSO_4$•$7H_2O$, 1.10 g H_3BO_3, 0.50 g $MnCl_2$•$4H_2O$, 0.16 g $FeSO_4$•$7H_2O$, 0.16 g $CoCl_2$•$5H_2O$, 0.16 g $CuSO_4$•$5H_2O$, and 0.11 g $(NH_4)_6Mo_7O_{24}$•$4H_2O$. The dropout components for selection for the three expression vectors were uracil/uridine, pyridoxine and riboflavin. *A. nidulans* A1145 ΔEM was initially grown on CD agar plates containing 10 mM uridine, 5 mM uracil, 0.5 µg/mL pyridoxine HCl and 2.5 µg/mL riboflavin at 37°C for 5 days. The germinated spores were harvested by centrifugation at 3,500 rpm for 10 min, and washed with Osmotic buffer (10 mL, 1.2 M $MgSO_4$, 10 mM sodium phosphate buffer, pH 5.8). The mycelia were then mixed with Osmotic buffer (10 mL, 30 mg lysing enzymes from *Trichoderma*, 20 mg Yatalase) in a 125-mL flask. Protoplasts were prepared by incubating the mixture overnight at 30 °C with gentle shaking at 80 rpm. Cells were collected in a 30-mL Corex tube and overlaid gently by 10 mL of Trapping buffer (0.6 M sorbitol, 0.1 M Tris HCI, pH 7.0). Centrifugation at 3,500 rpm for 15 min at 4 °C layered the protoplasts at the interface of the two buffers. The protoplasts were then pipetted to a sterile 15-mL falcon tube and washed with STC buffer (10 mL, 1.2 M sorbitol, 10 mM $CaCl_2$, 10 mM Tris-HCI pH 7.5). The protoplasts were resuspended in STC buffer (1 mL).

For each transformation, 3 µL of each plasmid (>100 ng/µL) was added to 60 µL of the *A. nidulans* A1145 ΔSTΔEM protoplast suspension prepared as above, and the mixture was incubated for 1 h on ice. 600 µL PEG solution (60% PEG, 50 mM of $CaCl_2$, and 50 mM of Tris-HCl, pH 7.5) was added to the protoplast mixture, followed by additional incubation at room temperature for 20 min. The mixture was spread on the CD sorbitol plate (CD solid medium with 1.2 M sorbitol and the appropriate supplements: 10 mM of uridine, 5 mM of uracil, 0.5 µg/mL of pyridoxine HCl, and/or 2.5 µg/mL of riboflavin according to the markers in the transformed plasmids) and incubated at 37 °C for 3-4 days.

Synthesis of *N*-succinyl proteinogenic amino acids and (2*R*,3*R*)-*t*-ES-Ile

All *N*-succinyl amino acids except for *N*-succinyl-Gly (Sigma) were synthesized following the protocol reported by Sumida et al[2]. A stirring solution of amino acid (2.0 mmol) and 20% NaOH (0.4 mL) in H_2O (1.7 mL), succinic anhydride (0.21 g, 2.1 mmol) and 20% NaOH (4.2 mL) were separately added at room temperature. The reaction temperature was strictly maintained under 50 °C to prevent undesired racemization. After 2 h, 1 M HCl

was added to adjust the pH of the reaction mixture to ~ 3.0 and then extracted with equal volume ethyl acetate twice. The combined organic layer was washed with brine (15 mL), dried over $MgSO_4$, and filtered. The filtrate was evaporated to obtain semi-purified product. The solid residue was purified by HPLC using water (0.1% trifluoroacetic acid) and MeCN as mobile phase. (2*R*,3*R*)-*t*-ES-Ile was synthesized according to the reported procedure[3] starting from (2*R*,3*R*)-3-(Ethoxycarbonyl)oxirane-2-carboxylic acid. ^{1}H NMR (500 MHz, DMSO): 8.68 ppm (1H, d, *J*=8.4 Hz), 4.21 ppm (1H, dd, *J*=5.7, 8.3 Hz), 3.76 ppm (1H, d, *J*=1.8 Hz), 3.46 ppm (1H, d, *J*=1.8 Hz), 1.81 ppm (1H, m), 1.19 ppm (1H, m), 1.41 ppm (1H, m), 0.86 ppm (6H, m). ^{13}C NMR (125 MHz, DMSO) δ 172.3, 168.8, 165.4, 56.6, 52.3, 51.1, 36.3, 15.5, 24.7, 11.3. HRMS (ESI, M+H$^+$) calculated for $C_{10}H_{16}NO_6^+$ 246.0972; found 246.0993. $[\alpha]_D^{24}$ + 20° (*c* 0.1, MeOH).

Spectroscopic data of *N*-succinyl-L-alanine matched literature spectra[2].

^{1}H NMR (500 MHz, DMSO) δ 8.15 ppm (1H, t, *J* = 7.0 Hz), 4.17 ppm (1H, m), 2.37 ppm (4H, m), 1.24 ppm (3H, d, *J* = 7.3 Hz). ^{13}C NMR (125 MHz, DMSO) δ 174.3, 173.8, 170.8, 47.5, 29.8, 29.1, 17.3. HRMS (ESI, M+H$^+$) calculated for $C_7H_{12}NO_5^+$ 190.0710; found 190.0700.

Spectroscopic data of *N*-succinyl-L-valine agree with the reported literature value[4].

^{1}H NMR (500 MHz, CD_3OD): δ 4.35 ppm (1H, dd, *J* = 2.9, 5.6 Hz), 2.59 ppm (4H, m), 2.15 ppm (1H, m), 0.95 ppm (6H, m). ^{13}C NMR (125 MHz, CD_3OD) δ 176.3, 174.9, 174.7, 58.8, 31.6, 31.2, 30.2, 19.5, 18.2. HRMS (ESI, M+H$^+$) calculated for $C_9H_{16}NO_5^+$ 218.1023; found 218.1021.

Spectroscopic data of *N*-succinyl-L-leucine agree with the reported literature value[5].

^{1}H NMR (500 MHz, D_2O): δ 4.36 ppm (1H, dd, *J* = 5.6, 9.1Hz), 2.61 ppm (2H, m), 2.55 ppm (2H, m), 1.61 ppm (3H, m), 0.88 ppm (3H, d, *J* = 6.5 Hz), 0.84 ppm (3H, d, *J* = 6.5 Hz). ^{13}C NMR (125 MHz, CD_3OD) δ 178.1, 177.9, 176.0, 53.0, 41.5, 32.1, 31.8, 26.3, 24.2, 22.7. HRMS (ESI, M+H$^+$) calculated for $C_{10}H_{18}NO_5^+$ 232.1179; found 232.1173.

Spectroscopic data of *N*-succinyl-L-isoleucine agree with the reported literature value[2].

^{1}H NMR (500 MHz, CD_3OD): δ 4.37 (1H, t, *J* = 5.4 Hz), 2.59 (4H, m), 1.88 (1H, m), 1.52 (1H, m), 1.25 (1H, m), 0.93 (6H, m). ^{13}C NMR (125 MHz, CD_3OD) δ 176.3, 174.9, 174.6, 58.1, 38.4, 31.3, 30.3, 26.2, 16.0, 11.8. HRMS (ESI, M+H$^+$) calculated for $C_{10}H_{18}NO_5^+$ 232.1179; found 218.1180.

Spectroscopic data of *N*-succinyl-L-methionine agree with the reported literature value[2].

^{1}H NMR (500 MHz, DMSO): δ 8.12 ppm (1H, d, *J* = 7.9), 4.37 ppm (1H, m), 2.47 ppm (6H, m), 2.05 ppm (3H, s), 1.97 ppm (1H, m), 1.87 ppm (1H, m). ^{13}C NMR (125 MHz, DMSO) δ 174.5, 174.1, 172.2, 51.6, 31.5, 30.4, 30.3, 29.6, 15.1.

^{1}H NMR data of *N*-succinyl-L-proline agree with the reported literature value[2].

^{1}H NMR (500 MHz, DMSO): ^{1}H NMR (500 MHz, DMSO) δ 4.48 ppm (0.5H, dd, *J* = 8.5 Hz), 4.20 ppm (1H, dd, *J* = 3.6, 8.9 Hz), 3.51 ppm (2H, m), 2.42 ppm (4H, m), 2.10 ppm (1H, m), 1.90-1.75 ppm (3H, m). HRMS (ESI, M+H$^+$) calculated for $C_9H_{14}NO_5^+$ 216.0866; found 216.0854.

Spectroscopic data of *N*-succinyl-L-serine agree with the reported literature value[2].

^{1}H NMR (500 MHz, DMSO): δ 8.00 ppm (1H, d, *J* = 8.0 Hz), 4.24 ppm (1H, m), 3.60 ppm (3H, m), 2.38 ppm (4H, m). ^{13}C NMR (125 MHz, DMSO) δ 173.9, 172.2, 171.2, 61.5, 54.7, 29.8, 29.1. HRMS (ESI, M+H$^+$) calculated for $C_7H_{12}NO_6^+$ 206.0659; found 206.0665.

Spectroscopic data of *N*-succinyl-L-theronine agree with the reported literature value[2].

^1H NMR (500 MHz, DMSO): δ 7.77 ppm (1H, m), 4.19 ppm (1H, dd, J = 3.3, 8.8 Hz), 4.07 ppm (1H, m, J = 3.3, 6.4 Hz), 2.48 ppm (4H, m), 1.02 (3H, d, J = 6.4 Hz). ^{13}C NMR (125 MHz, DMSO) δ 173.8, 172.2, 171.5, 66.4, 57.6, 29.8, 29.2, 20.3. HRMS (ESI, M+H$^+$) calculated for $C_8H_{14}NO_6^+$ 220.0816; found 220.0811.

Spectroscopic data of *N*-succinyl-L-cysteine agree with the reported literature value[2].

H NMR (500 MHz, DMSO): δ 8.21 ppm (1H, d, J = 7.5 Hz), 4.36 ppm (1H, dt, J = 6.3, 13.3 Hz), 2.90 ppm (1H, d, J = 5.7, 13.1 Hz), 2.78 ppm (1H, J = 7.9, 13.1 Hz), 2.37 ppm (4H, m). HRMS (ESI, M+H$^+$) calculated for $C_7H_{12}SNO_5^+$ 222.0431; found 222.0425.

Spectroscopic data of *N*-succinyl-L-asparagine agree with the reported literature value[2].

H NMR (500 MHz, DMSO): 8.06 ppm (1H, d, J = 7.9 Hz), 7.31 ppm (1H, s), 6.86 ppm (1H, s), 4.46 ppm (1H, m), 2.48 ppm (6H, m). HRMS (ESI, M+H$^+$) calculated for $C_8H_{13}N_2O_6^+$ 233.0768; found 233.0752.

Spectroscopic data of *N*-succinyl-L-glutamine agree with the reported literature value[2].

^1H NMR (500 MHz, DMSO): δ 8.14 ppm (1H, d, J = 7.8 Hz), 7.26 ppm (1H, s), 6.77 ppm (1H, s), 4.14 ppm (1H, m), 2.39 ppm (4H, m), 2.10 ppm (2H, m), 1.92 ppm (1H, m), 1.73 ppm (1H, m). ^{13}C NMR (125 MHz, DMSO) δ 173.8, 173.5, 173.5, 171.2, 51.6, 30.8, 29.8, 29.1, 27.0. HRMS (ESI, M+H$^+$) calculated for $C_9H_{15}N_2O_6^+$ 247.0925; found 247.0925.

Spectroscopic data of *N*-succinyl-L-phenylalanine agree with the reported literature value[4].

^1H NMR (500 MHz, CD_3OD): δ 7.23 ppm (5H, m), 4.66 ppm (1H, dd, J = 5.2, 8.5 Hz), 3.18 ppm (1H, dd, J = 5.3, 13.9 Hz), 2.96 ppm (1H, dd, J = 8.6, 13.9), 2.48 ppm (4H, m). ^{13}C NMR (125 MHz, CD_3OD) δ 176.1, 174.6, 174.3, 138.3, 130.3, 129.4, 127.8, 55.0, 38.4, 31.3, 30.2. HRMS (ESI, M+H$^+$) calculated for $C_9H_{14}NO_5^+$ 216.0866; found 216.0854.

Spectroscopic data of *N*-succinyl-L-tyrosine agree with the reported literature value[2].

^1H NMR (500 MHz, DMSO): 8.10 ppm (1H, d, J = 8.0 Hz), 6.98 ppm (2H, m), 6.64 (2H, m), 4.32 ppm (1H, dd, J = 4.4, 8.8 Hz), 2.89 ppm (1H, dt, J = 3.7, 8.9 Hz), 2.73 ppm (1H, dt, J = 5.3, 8.8 Hz), 2.34 ppm (4H, m). ^{13}C NMR (125 MHz, CD_3OD) δ 174.2, 173.6, 171.4, 156.3, 130.5, 128.1, 115.4, 54.3, 36.6, 30.3, 29.5. HRMS (ESI, M+H$^+$) calculated for $C_{13}H_{16}NO_6^+$ 282.0972; found 282.0967.

Spectroscopic data of *N*-succinyl-L-tryptophan agree with the reported literature value[2].

^1H NMR (500 MHz, DMSO): 8.16 ppm (1H, d, J = 7.8 Hz), 7.55 ppm (1H, t, J = 7.3 Hz), 7.35 (1H, dd, J = 5.6, 8.1Hz), 7.17 ppm (1H, m), 7.07 ppm (1H, m), 6.99 ppm (1H, m), 4.52 ppm (1H, m), 3.19 ppm (1H, m), 3.05 ppm (1H, m), 2.39 ppm (4H, m). ^{13}C NMR (125 MHz, CD_3OD) δ 174.2, 173.8, 171.5, 136.4, 127.6, 123.9, 121.2, 118.7, 118.5, 111.7, 110.2, 53.4, 30.2, 29.3, 27.5. HRMS (ESI, M+H$^+$) calculated for $C_{15}H_{17}N_2O_5^+$ 305.1132; found 305.1130.

Spectroscopic data of *N*-succinyl-L-aspartate agree with the reported literature value[2].

^1H NMR (500 MHz, DMSO): 8.19 ppm (1H, d, J = 8.0 Hz), 4.49 ppm (1H, m), 2.64 ppm (1H, m), 2.52 ppm (1H, m), 2.36 ppm (4H, m). HRMS (ESI, M+H$^+$) calculated for $C_8H_{12}NO_7^+$ 234.0608; found 234.0600.

Spectroscopic data of *N*-succinyl-L-glutamic acid agree with the reported literature value[3].

^1H NMR (500 MHz, D_2O): 4.40 ppm (1H, dd, J = 5.1, 9.3), 2.58 ppm (4H, m), 2.47 ppm (2H, m), 2.18 ppm (1H, m), 1.98 ppm (1H, m). ^{13}C NMR (125 MHz, D_2O) δ 180.3, 180.1, 178.3, 178.2, 55.2, 33.2, 32.3, 32.0, 29.0. HRMS (ESI, M+H$^+$) calculated for $C_9H_{14}NO_7^+$ 248.0765; found 248.0760.

Spectroscopic data of *N*-succinyl-L-histidine agree with the reported literature value[2].

^1H NMR (500 MHz, D_2O): 8.47 ppm (1H, d, J = 5.7), 7.17 ppm (1H, d, J = 5.7), 4.65 ppm (1H, m), 3.22 ppm (1H, m), 3.05 ppm (1H, m), 2.48 ppm (4H, m). HRMS (ESI, M+H$^+$) calculated for $C_{10}H_{14}N_3O_5^+$ 256.0928; found 256.0927.

Spectroscopic data of *N*-succinyl-L-lysine agree with the reported literature value[2].

^{1}H NMR (500 MHz, D_2O): 4.25 ppm (1H, m), 2.86 ppm (1H, m), 2.52 ppm (4H, m), 1.79 ppm (1H, m), 1.65 ppm (1H, m), 1.55 ppm (2H, m), 1.34 ppm (2H, m). ^{13}C NMR (125 MHz, D_2O) δ 176.8, 175.7, 174.9, 52.3, 39.1, 29.9, 29.8, 29.0, 26.1, 21.9. HRMS (ESI, $M+H^+$) calculated for $C_{10}H_{19}N_2O_5^+$ 247.1288; found 247.1290.

Spectroscopic data of *N*-succinyl-L-arginine agree with the reported literature value[2].

^{1}H NMR (500 MHz, D_2O): 4.26 ppm (1H, m), 3.10 ppm (2H, t, J = 6.9 Hz), 2.55 ppm (4H, m), 1.81 ppm (1H, m), 1.65 ppm (1H, m), 1.54 ppm (2H, m). ^{13}C NMR (125 MHz, D_2O) δ 176.9, 175.6, 174.8, 156.7, 52.4, 30.2, 29.9, 29.0, 27.7, 24.2. HRMS (ESI, $M+H^+$) calculated for $C_{10}H_{19}N_4O_5^+$ 275.1350; found 275.1347.

Preparation of (2*S*,3*S*)-*t*-ES or (2*R*,3*R*)-*t*-ES standard

(2*S*,3*S*)-*t*-ES or (2*R*,3*R*)-*t*-ES was obtained by the hydrolysis from corresponding (2*S*,3*S*)-diethyl-2,3-epoxysuccinate and (2*R*,3*R*)-diethyl-2,3-epoxysuccinate, respectively. A solution of aqueous NaOH (40 mg, 2 eq.) was added to (2*S*,3*S*)-diethyl-2,3-epoxysuccinate (94 mg, 1 eq.) in an ice bath. The resulting solution was stirred for 2 h at 0°C, then for 30 min at room temperature, after which the solution was neutralized and lyophilized to yield (2*S*,3*S*)-*t*-ES. ^{1}H NMR (400 MHz, D_2O): 3.39 (s, 2H); ^{13}C NMR (100 MHz, D_2O): 175.1, 53.9. Similarly (2*R*,3*R*)-diethyl-2,3-epoxysuccinate was treated as described above to yield (2*R*,3*R*)-*t*-ES. ^{1}H NMR (400 MHz, D_2O): 3.45 (s, 2H); ^{13}C NMR (100 MHz, D_2O): 174.6, 53.9.

Sequence information

Cp1A amino acid sequence

MQQFVRNVNPARIGDITTQVSRVVPHLIASDLSCATRSSHVTEVSNALRKSGILKVSLQFKDDASKYLQNL
ILGLHKHHGHGLPITHSASRGWFWDIRPNSTTFQTPSHQARSETMQEFPWHTDCSYEEAPPKYFALQVLR
EDRCGGGTLSVMNVGKLSSMLSPSTCAALLRPQFRIDVPPEFVKNDASRHIIGSLMAADSSGAPNMLRFR
EDIMTPLNVEAAAALVELKDRLLGLEVQAETLHLTPDCLPRGSVVLMDNRRWLHARNEVMDPERHLRR
VRWDARPFPAMTM

Cp1B amino acid sequence

MKIPAPQQLQQLHVSLDGGHYEPVTTFDPAKATYLQDQEALQENLLRLCSVNGWHKSSRAACSPRPVLV
SSEHQRRWRELHEALVLAITDIVERWLTDPEARFPERMPLEPEEEDLLRWIDEQVPHNLPQYRDCRGSWR
PDFLVEEENSEDGSGPVENFRISEINARFSFNGFMFATCGQQAIHDMGICDNGNGLVGATDPAKILKGLLR
LFQPGLPLHLLKGDEAGVDIHMLVDFLDRYLGITPRFIMPADLRLLHEPQAKGGYKLCCVVKNPDSCDPA
TLIYHDGDILEEIHQVGLELHQREIRALEPEMLRQISLRCFNDMRTILLVHDKRMLGIVRQELENLVARNV
LTLSQAKILDKGIPETILPGSLDLDQAIARCKEMPELKDEYILKPIRSGKGDGIVFGEDLNSEEWISRLEGLR
SAQLIPGGGTCIVQRKVKQLLYDVVLRPTGVKTRYPLIGTYHSINGEFLGVGVWRSSPDRICAISHGGAWT
VSVMRDE

Cp1D amino acid sequence

MAPTTFSLKEVLAVAEIHPFYNPAVEYPPTPETIKSAIELADKRSTDIDLSSLPLVSKKDLYKAIARLTDDTSP
QNEYRRSSYVSITGGGSGGLPLMFVTDTKENRNQRAVFGEFLSTCGVVEPHDWILTTHTSGYFYRSLDLL
SEILENAGATVLSAGNYMTPAEVVHALAHYHVNVITGDGSQVVQVVHHISTLPAEEKAKIKLTKVLYTSE
PLTETQQIHIRATLGPVKICSVWGSAEAGPCALSDPDLTSPERPPGTMDFIFDTRQVVIEILPHSASEGDSSA
GVKSVPDGEEGIIVQTSLVRRRNPLVRYITGDVGSLQPLPEKARAIIPESELEHLRVLRLRGRDRRFSFKWF

GIYFEFENIVSFMQGDKTGVLQWQVILATLESSPQTKLEIRLLRQANNEHIMTKEELLNKLEKYFFILPENE
HLFQVTFLDDLSGFEKSSTGNKVMKFVDKVH

Cp2B amino acid sequence
MKYPTTGQLQQVHLGIGPKGYEPVASYQGDKQLYTQEHEILQASILGFCPEHLWHHGSNKASCPRPILVT
AKHQEQLEQLHNALVTAIVDIVKRWWTDLDARFPERMPLTRDEEDLLRWLEHQHSHNGVPYEARLGSW
RPDFLVGDYSGGPSTETYRLTEINARFCFNGFMHQAYGQEGLSDLGAGRNGLIHATDSSKILDGLLSLFNP
DRPLHLLKGEEPGIDIHMFIDFVYRHIGIKPRLITPADLRLIPDPQKKDGSKLCCLVKDQQNASLINESRLLV
TSKGEVVEEVHQVGLELHQHELFGLSREMLREISLRCFNDMRTILLVHDKRMLGIIKQEMPTLVARKVLT
HDQGEALERGISDSFIPGSSELNELIQTLTDSPELRKEYLLKPIRGGKGAGIIFGDEVGPDEWLSTLERLRNP
HFVSGNTMYVVQRRIWPRLYEVILNSSGDRGNYPLIGTYHTTNGQLLGLGTWRSSPDRICAVSHGGGWIC
SVLDEYAESSE

Cp2D amino acid sequence
MTTKSFSLSEVLAVAKRHPFYNPEIQYPLDETALQAVRDWAVKNQTEVDLRFQPLLHKNDIYKTVERLTH
DASPENVYRESSYMSITGGGSGGVPMMFAVDVHENRQQRAQMGKLLRNCGVIRRKDWVLSVHISGGFY
RSLDLTTETMENAGATVLSAGNYMEPEEVVQALAHYHVNVLTGDASQIVQLACYISTLPLERQRQIQINK
IIYTSEPLTGAQRAFLRATLGDVKICSVMGSSEAGPWALSNPDLVGEENLNSSSMDFVFDTRDMIIEILSPA
GLDDGKPPSDIDPLPLGETGIIVQTSLRRLRNPLVRYITGDLGSLHPLPEIASAVVPESERQYLRVLRMQGR
DRRFSFKWYGAYFEFEKMKALLQAEECGVLQWQVILDQLESSGLPTLQVRLLRAPSRADVLSEEQLVKR
VRTFFLVLPENEDVFSIVFVKNLDGFERSSTAGKVISFVDRLH

Epoxysuccinate synthase MfaA from *Microcoleus sp. FACHB-1* amino acid sequence
MIAAKKTDLLAIEENPLILPASCLFQIDTKNDIDFDAYASALFEAGIILLDLGFDNPDASIMTTIVEHLGTIDT
HDGKGMVIWDVKYDANVDQDKGTRSLTTKKFPIHTDASFEEPPPQYVALYVVAEDSLGGGITQLIDGRQI
LQHLSREAISVLQTKAFKFRVPQEFIKNKAYIEASILNGEGNFRYRQEVLILDDCTPQELQAIGELELLLAN
KSLIKSIFLKTGTIIIFDNGRFLHGRTKVRDKNRHLKRLRFQAKQTRFGVDCETYVYRKESGCLG

Polyphosphate kinase (CHU)
MATDFSKLSKYVETLRVKPKQSIDLKKDFDTDYDHKMLTKEEGEELLNLGISKLSEIQEKLYASGTKSVLI
VFQAMDAAGKDGTVKHIMTGLNPQGVKVTSFKVPSKIELSHDYLWRHYVALPATGEIGIFNRSHYENVL
VTRVHPEYLLSEQTSGVTAIEQVNQKFWDKRFQQINNFEQHISENGTIVLKFFLHVSKKEQKKRFIERIELD
TKNWKFSTGDLKERAHWKDYRNAYEDMLANTSTKQAPWFVIPADDKWFTRLLIAEIICTELEKLNLTFP
TVSLEQKAELEKAKAELVAEKSSD

Isolated yield of *trans*-epoxysuccinyl amino acids by enzymatic synthesis with Cp1B or Cp2B

Reaction conditions: 20 mL reaction in 50 mM sodium phosphate buffer (pH 8.0) containing purified 2.5 μM Cp1B or Cp2B with 5 mM (±)-***t*-ES**, 2.5 mM amino acid, 10 mM ATP, 10 mM $MgCl_2$ was incubated at 30 °C for 16 h. Isolated yield for the compounds shown in Fig. 3c is listed below. ***t*-ES**-Ile (9.0 mg, 73% with Cp1B), ***t*-ES**-Leu (8.6 mg, 70% yield with Cp1B), ***t*-ES**-Phe (7.0 mg, 50% yield with Cp1B), ***t*-ES**-Tyr (8.0 mg, 54% yield with Cp2B), ***t*-ES**-Val (7.0 mg, 61% yield with Cp1B), ***t*-ES**-Trp (8.3 mg, 53% yield with Cp2B), ***t*-ES-a1** (1.1 mg from

2 × 20 mL reaction, <5% yield with Cp1B), ***t*-ES-a2** (2.5 mg, 22% yield with Cp1B), ***t*-ES-a3** (5.5 mg, 45% yield with Cp1B), ***t*-ES-a4** (4.9 mg, 38% yield with Cp1B), ***t*-ES-a5** (5.0 mg, 41% yield with Cp1B), ***t*-ES-a6** (8.0 mg, 62% yield with Cp1B), ***t*-ES-a7** (8.3 mg, 61% yield with Cp1B), ***t*-ES-a8** (8.6 mg, 60% yield with Cp1B), ***t*-ES-a9** (7.2 mg, 59% yield with Cp1B), ***t*-ES-a10** (7.8 mg, 61% yield with Cp1B), ***t*-ES-a11** (9.2 mg, 68% yield with Cp1B), ***t*-ES-a12** (3.2 mg, 26% yield with Cp1B), ***t*-ES-a13** (7.8 mg, 60% yield with Cp1B), ***t*-ES-a14** (4.8 mg, 33% yield with Cp1B), ***t*-ES-a15** (8.0 mg, 52% yield with Cp2B), ***t*-ES-a16** (7.2 mg, 54% yield with Cp2B), ***t*-ES-a17** (1.4 mg from 2 × 20 mL reaction, <5% yield with Cp2B), ***t*-ES-a18** (4.4 mg, 28% yield with Cp2B), ***t*-ES-a19** (7.1 mg, 43% yield with Cp2B), ***t*-ES-a20** (7.1 mg, 38% yield with Cp2B), ***t*-ES-a21** (6.5 mg, 38% yield with Cp2B), ***t*-ES-a22** (7.3 mg, 50% yield with Cp2B), ***t*-ES-a23** (1.5 mg, 10% yield with Cp1B), ***t*-ES-a24** (7.5 mg, 48% yield with Cp2B), ***t*-ES-a25** (6.3 mg, 43% yield with Cp2B), ***t*-ES-a26** (6.9 mg, 47% yield with Cp2B), ***t*-ES-a27** (5.6 mg, 38% yield with Cp2B), ***t*-ES-a28** (7.1 mg, 45% yield with Cp2B), ***t*-ES-a29** (4.1 mg, 28% yield with Cp1B), ***t*-ES-a30** (6.1 mg, 41% yield with Cp1B), ***t*-ES-a31** (1.4 mg, 10% yield with Cp1B), ***t*-ES-a32** (3.3 mg, 23% yield with Cp1B).

Isolated yield of 15-b15, 15b-(18-20), 15-b24, 15-b27, 15-b(29-33), 15-b(37-38), and 15-b41 by enzymatic synthesis with Cp1D

Reaction conditions: 15 mL reaction in 50 mM sodium phosphate buffer (pH 8.0) containing 2.0 mM **15**, 5.0 mM amine donor, and 2.5 μM Cp1D was performed at 30 ºC for 16 h. Isolated yield for the selected compounds shown in Fig. 4 is listed below. **15-b15** (10.6 mg, 91% yield), **15-b18** (9.4 mg, 82% yield), **15-b19** (9.2 mg, 77% yield), **15-b20** (9.6 mg, 81% yield), **15-b24** (7.7 mg, 72% yield), **15-b27** (8.3 mg, 69% yield), **15-b29** (4.3 mg, 38% yield), **15-b30** (10.9 mg, 94% yield), **15-b31** (10.9 mg, >95% yield), **15-b32** (9.2 mg, 79% yield), **15-b33** (6.2 mg, 62% yield), **15-b37** (10.6 mg, 90% yield), **15-b38** (10.8 mg, 70% yield), **15-b41** (11.5 mg, >95% yield).

Spectroscopic data of E-64c, enzymatically synthesized with Cp1B and Cp1D

^{1}H NMR (DMSO-d_6, 500 MHz) δ 0.83–0.89 ppm (12H, m), 1.27 (2H, q, J = 7.1 Hz), 1.45–1.58 (4H, m, CH_2, 2 × CH), 3.04 (2H, m), 3.45 (1H, d, J = 1.8 Hz), 3.66 (1H, d, J = 1.9 Hz), 4.30 (1H, m), 8.03 (1H, t, J = 5.4 Hz), 8.56 (1H, d, J = 8.4 Hz); ^{13}C NMR (DMSO-d_6, 125 MHz) δ 171.0, 168.8, 164.9, 52.7, 51.2, 51.2, 41.1, 38.0, 36.8, 25.1, 24.3, 22.9, 22.4, 22.4, 21.7. HRMS (ESI, M+H$^+$) calculated for $C_{15}H_{27}N_2O_5^+$ 315.1914; found 315.1921. $[\alpha]_D^{24.1}$ + 43° (c 0.1, MeOH). The spectral data match the previous report[6].

Supplementary Tables

Supplementary Table 1. Comparative BLASTP analysis of the *cp1* gene cluster with homologous cluster from ascomycetes *Aspergillus flavus*, *Trichoderma harzianum*, and cyanobacterium *Microcoleus sp. FACHB-1*.

Aspergillus flavus cp1	*Aspergillus flavus* (% identity / % similarity) *cp2*	*Trichoderma harzianum* (% identity / % similarity) *tcp*	*Microcoleus sp. FACHB-1* (% identity / % similarity) *mfa*	Homolog in Swiss-Prot database (% identity)
Cp1A XP_041142152.1	Cp2A (61/76) XP_041145391.1	TcpA (61/76) KKO98516.1	MfaA (30/52) MBD2130326.1	L-asparagine oxygenase (29%) Q9Z4Z5.1
Cp1B XP_041142153.1	Cp2B (53 / 69) XP_041145392.1	TcpB (51/66) KKO98515.1	MfaB (25/41) MBD2130328.1	No hits
Cp1C XP_041142154.1	Cp2C (58/74) XP_041145390.1	*Not conserved*	*Not conserved*	FlvG (42%) PLP-dependent decarboxylase B8NHE2.1
Cp1D XP_041142155.1	Cp2D (57/74) XP_041145389.1	TcpD (52/70) KKO98517.1	MfaD (24/43) MBD2130327.1	No hits

Supplementary Table 2. Primers used in this study

Primers	Sequence (5'-3')
pML 8001 F1	CTGAGCTTCATCCCCAGCATCATTACACCTCAGCAATGCAACAATTCGTACGCAATGTC
pML 8001 R1	AGAATCAGTAAGCTCACATGTATTCCTGGAGCAAACTTCCTTGTGGACGAAATGCAGC
pML 8001 F2	TTTGCTCCAGGAATACATGTGAGCTTAC
pML 8001 R2	TTTTGCGATTGTTTGAAGTGTTCTGTATGC
pML 8001 F3	CAAGTGCATACAGAACACTTCAAACAATCGCAAAAATGAAGATTCCAGCTCCCCAAC
pML 8001 R3	ACACAGTGGAGGACATACCCGTAATTTTCTGATTTAAATGTGGTTGCCACTTAGTCAGGG
pML 8002 F1	CCTTCTCTGAACAATAAACCCCACAGAAGGCATTTATGATGGTGACGGACATGGC
pML 8002 R1	TCAGTAAGCTCACATGTATTCCTGGAGCAAAGCCGTGAACCCTAATATAAACATCAAAGC
pML 8002 F2	TTTGCTCCAGGAATACATGTGAGCTTAC
pML 8002 R2	TTTTGCGATTGTTTGAAGTGTTCTGTATGC
pML 8002 F3	CAAGTGCATACAGAACACTTCAAACAATCGCAAAAATGGCTCCTACCACATTTTCCC
pML 8002 R3	TGATGAGACCCAACAACCATGATACCAGGGGATTTAAATGTTCTTCTGCCGTTGTCGACG
pML 8003 F1	CTCTCCCTTCTCTGAACAATAAACCCCACAGAAGGCATTTATGATGGTGACGGACATGGC
pML 8003 R1	ATAGGTCGCCAGGTACGACCAGTTCGGAAGATCAGGAAATGTTCTTCTGCCGTTGTCGAC
pML 8003 F2	CGACATACGCTGCGTCGACAACGGCAGAAGAACATTTCCTGATCTTCCGAACTGGTCG
pML 8003 R2	TGCTGAGGTGTAATGATGCTGGGGATGA
pML 8003 F3	CTGAGCTTCATCCCCAGCATCATTACACCTCAGCAATGCAACAATTCGTACGCAATGTC
pML 8003 R3	AGTGATGAGACCCAACAACCATGATACCAGGGGATTTCTTCCTTGTGGACGAAATGCAGC
pML 8004 F1	CTCCCTTCTCTGAACAATAAACCCCACAGAAGGCATTTATGATGGTGACGGACATGGC
pML 8004 R1	GGTCTCTCCCGTCACCCAAATCAATTCACCGGAGTAAATGTTCTTCTGCCGTTGTCGAC
pML 8004 F2	ACGCTGCGTCGACAACGGCAGAAGAACATTTACTCCGGTGAATTGATTTGGGTG
pML 8004 R2	GTTGTTGGAGTTGTTGGGGAGCTGGAATCTTCATTGTTTAGATGTGTCTATGTGGCGGG
pML 8004 F3	CTAACCATTACCCCGCCACATAGACACATCTAAACAATGAAGATTCCAGCTCCCCAAC
pML 8004 R3	GGAGTGATGAGACCCAACAACCATGATACCAGGGGATTTGTGGTTGCCACTTAGTCAGGG
pML 8005 F1	GAGCTTCATCCCCAGCATCATTACACCTCAGCAATGCAACAATTCGTACGCAATGTC
pML 8005 R1	TAGCTCGTTCGGCACCTTTAATCAAATGTGGTTGCCACTTAGTCAGGGCT
pML 8005 F2	ATGGGCTTAAAGCCCTGACTAAGTGGCAACCACATTTGATTAAAGGTGCCGAACGAGCTA
pML 8005 R2	CACAGTGGAGGACATACCCGTAATTTTCTGATTTCGGAATGTCTCGGATTAATATCACCG
pML 8006 F1	GCTTCATCCCCAGCATCATTACACCTCAGCAATGCAACAATTCGTACGCAATGTC
pML 8006 R1	GGGTCTCTCCCGTCACCCAAATCAATTCACCGGAGTAAATGTGGTTGCCACTTAGTCAGG
pML 8006 F2	ATGGGCTTAAAGCCCTGACTAAGTGGCAACCACATTTACTCCGGTGAATTGATTTGGGTG
pML 8006 R2	GAACCTCCTTGAGGGAAAATGTGGTAGGAGCCATTGTTTAGATGTGTCTATGTGGCGGG
pML 8006 F3	CTAACCATTACCCCGCCACATAGACACATCTAAACAATGGCTCCTACCACATTTTCCC
pML 8006 R3	TCAACACAGTGGAGGACATACCCGTAATTTTCTGATTTAGTTCTTCTGCCGTTGTCGACG
pML 8007 F1	TTCTCTGAACAATAAACCCCACAGAAGGCATTTATGCAACATATAGGACGTCATTTCCAC
pML 8007 R1	AATCAGTAAGCTCACATGTATTCCTGGAGCAAACGGTTCTTGCGATTTGTTTTCTTTTCC
pML 8007 F2	TTTGCTCCAGGAATACATGTGAGCTTAC
pML 8007 R2	TTTTGCGATTGTTTGAAGTGTTCTGTATGC
pML 8007 F3	ACAAGTGCATACAGAACACTTCAAACAATCGCAAAAATGAAGTACCCTACTACCGGACAG
pML 8007 R3	GATGAGACCCAACAACCATGATACCAGGGGATTTAAATCTAGCTGCAGCTGAAGTACCAG
pML 8008 F1	CTGAGCTTCATCCCCAGCATCATTACACCTCAGCAATGGGAAGTGTGGGCATCTCT
pML 8008 R1	TAGAATCAGTAAGCTCACATGTATTCCTGGAGCAAATACTCTCTGCCAAGAGCAATAGGC
pML 8008 F2	TTTTCCTCGCCTATTGCTCTTGGCAGAGAGTATTTGCTCCAGGAATACATGTGAGCTTAC
pML 8008 R2	CCTCCGACAACGAGAAGCTTTTCGTGGTCATTTTTGCGATTGTTTGAAGTGTTCTGTATG
pML 8008 F3	TACAAGTGCATACAGAACACTTCAAACAATCGCAAAAATGACCACGAAAAGCTTCTCGT
pML 8008 R3	CAGTGGAGGACATACCCGTAATTTTCTGATTTAAATGAACACCAGGAATCCAGCATTAGC
pML 8009 F1	CATCATCACAGCAGCGGCCTGGTGCCGCGCGGCAGCATGCAACAATTCGTACGCAATGTC
pML 8009 R1	GGATCTCAGTGGTGGTGGTGGTGGTGCTCGAGTCACTACATAGTCATGGCAGGAAATGGC
pML 8010 F1	ATCATCATCACAGCAGCGGCCTGGTGCCGCGCGGCAGCATGAAGATTCCAGCTCCCCAAC
pML 8010 R1	GATCTCAGTGGTGGTGGTGGTGGTGCTCGAGTCACTATTCATCACGCATAACAGAAACCG
pML 8011 F1	ATCATCATCACAGCAGCGGCCTGGTGCCGCGCGGCAGCATGGCTCCTACCACATTTTCCC
pML 8011 R1	TCAGTGGTGGTGGTGGTGGTGCTCGAGTCATCAATGTACCTTGTCTACAAACTTCATGAC

Supplementary Table 2 (continued). Primers used in this study.

Primers	Sequence (5'-3')
pML 8012 F1	ATCATCATCACAGCAGCGGCCTGGTGCCGCGCGGCAGCATGGGAAGTGTGGGCATC
pML 8012 R1	GTGGTGGTGGTGGTGGTGCTCGAGTCATTAGAAATCTTGGTAAATTACAGTGTAGCTATT
pML 8013 F1	CATCATCACAGCAGCGGCCTGGTGCCGCGCGGCAGCATGAAGTACCCTACTACCGGACAG
pML 8013 R1	CCGGATCTCAGTGGTGGTGGTGGTGGTGCTCGAGTCATCACTCTGAGCTCTCCGCG
pML 8014 F1	CATCATCACAGCAGCGGCCTGGTGCCGCGCGGCAGCATGACCACGAAAAGCTTCTCGTTG
pML 8014 R1	AGTGGTGGTGGTGGTGGTGCTCGAGTCATCAATGCAAACGATCTACAAAGCTAATAACTT
pML 8015 F1	CATCACAGCAGCGGCCTGGTGCCGCGCGGCAGCATGATCGCCGCTAAAAAAACGGACT
pML 8015 R1	GCAGCCGGATCTCAGTGGTGGTGGTGGTGGTGCTCGAGTCACCCAAGGCACCCGGACTC
pML 8016 F1	AAATAATTTTGTTTAACTTTAAGAAGGAGATATACCATGGCAACCGATTTTAGCAAACTG
pML 8016 R1	GTTAGCAGCCGGATCTCAGTGGTGGTGGTGGTGGTGATCGCTTGATTTTTCTGCAACCAG

Supplementary Table 3. Plasmids used in this study

Plasmids	Vector	Genes
pML 8001	pYTU	*cp1A* (oxygenase)-*cp1B* (HP)
pML 8002	pYTP	*cp1C* (decarboxylase)-*cp1D* (AMP-binding)
pML 8003	pYTP	*cp1C* (decarboxylase)-*cp1D* (AMP-binding)-*cp1A* (oxygenase)
pML 8004	pYTP	*cp1C* (decarboxylase)-*cp1D* (AMP-binding)-*cp1B* (HP)
pML 8005	pYTU	*cp1A* (oxygenase)-*cp1B* (HP)-*cp1C* (decarboxylase)
pML 8006	pYTU	*cp1A* (oxygenase)-*cp1B* (HP)-*cp1D* (AMP-binding)
pML 8007	pYTP	*cp2A* (oxygenase)-*cp2D* (AMP-binding)
pML 8008	pYTU	*cp2C* (decarboxylase)-*cp2B* (HP)
pML 8009	pET28a	*cp1A* (oxygenase)
pML 8010	pET28a	*cp1B* (HP)
pML 8011	pET28a	*cp1D* (AMP-binding)
pML 8012	pET28a	*cp2C* (decarboxylase)
pML 8013	pET28a	*cp2B* (HP)
pML 8014	pET28a	*cp2D* (AMP-binding)
pML 8015	pET28a	*mfaA* (oxygenase)
pML 8016	pET28a	Polyphosphate kinase

Supplementary Table 4. X-ray data collection and refinement statistics of Cp1B.

	Cp1B (PDB 9CJN)
**Data collection**	
Space group	$P2_12_12_1$
Cell dimensions	
a, *b*, *c* (Å)	69.85, 96.23, 129.0
α, β, γ (°)	90.00, 90.00, 90.00
Resolution (Å)	77.14-2.70 (2.80-2.70)
R_{sym} or R_{merge}	0.130 (1.22)
$I / \sigma I$	16.0 (2.2)
CC(1/2)	0.999 (0.774)
Completeness (%)	99.9 (99.1)
Redundancy	13.1 (13.0)
**Refinement**	
Resolution (Å)	77.14-2.70 (2.79-2.70)
No. reflections	24508 (2358)
R_{work} / R_{free}	0.2021/0.2496
No. atoms	3896
Protein	3788
Ligand/ion	45
Water	63
B-factors (Å^2)	57.69
Protein	57.48
Ligand/ion	87.13
Water	49.33
R.m.s. deviations	
Bond lengths (Å)	0.007
Bond angles (°)	0.89
Ramachandran plot (%)	
Outliers	0.00
Favored	97.49
Allowed	2.51

The structure was obtained using diffraction data from a single crystal. Values in parentheses are for highest-resolution shell.

Supplementary Table 5. Table of statistics from crystallographic data reduction and refinement for structure of papain without inhibitor bound, or co-crystallized with E64, E64-c or E64-d, and (2*S*,3*S*)-***t*-ES-a9-b7**.

	Apo Papain (PDB, 9CLH)	Papain E-64 (**1**) (PDB, 9CKT)	Papain E64-c (PDB, 9EG7)	Papain (2*S*,3*S*)-***t*-ES-a9-b7** (PDB, 9CKY)	Papain E64-d (PDB, 9CKW)
Temperature	100 K	100 K	100 K	100 K	100 K
Wavelength	1.54 Å	1.54 Å	1.54 Å	1.54 Å	1.54 Å
Data processing					
Crystal system	Orthorhombic	Orthorhombic	Orthorhombic	Orthorhombic	Orthorhombic
Space group	$P2_12_12_1$	$P2_12_12_1$	$P2_12_12_1$	$P2_12_12_1$	$P2_12_12_1$
Unit cell constants:					
a, b, c (Å)	42.45, 49,23, 101.66	42.41, 48.83, 101.73	43.91, 49.13, 90.65	42.47, 48.95, 101.58	43.88, 49.30, 90.69
α, β, γ (°)	90.00, 90.00, 90.00	90.00, 90.00, 90.00	90.00, 90.00, 90.00	90.00, 90.00, 90.00	90.00, 90.00, 90.00
Resolution (Å)	50.83 – 1.60 (1.70 – 1.60)	39.14 – 1.50 (1.60 – 1.50)	32.74 – 1.50 (1.60 – 1.50)	35.25 – 1.40 (1.50 – 1.40)	26.56 – 1.40 (1.50 – 1.40)
No. unique reflections	51718 (8338)	34646 (5987)	32169 (5578)	42180 (7511)	39328 (7086)
R_{merge}	0.063 (0.118)	0.062 (0.260)	0.057 (0.141)	0.065 (0.653)	0.086 (0.186)
R_{meas}	0.067 (0.126)	0.064 (0.271)	0.059 (0.147)	0.068 (0.687)	0.09 (0.196)
Completeness (%)	95.0 (91.7)	99.8 (100.0)	100.0 (100.0)	99.0 (95.9)	99.5 (97.5)
Redundancy	7.25 (7.49)	12.90 (13,15)	13.28 (13.03)	12.56 (10.22)	12.70 (10.09)
I/σ	21.14 (13.32)	24.66 (9,42)	31.36 (17.33)	20.70 (4.07)	20.47 (9.76)
$CC_{1/2}$ (%)	99.8 (99.3)	99.9 (98.5)	0.999 (0.995)	99.9 (91.7)	99.8 (98.5)
Refinement program	*PHENIX*	*PHENIX*	*PHENIX*	*PHENIX*	PHENIX
R_{work}, R_{free}	0.1542, 0.1908	0.1855, 0.2190	0.1432, 0.1649	0.1597, 0.1988	0.1356, 0.1754
B-factors ($Å^2$)					
Protein	14.15	17.34	10.63	18.99	11.45
Ligand/ion/other	9.34	21.54	21.55	28.16	28.84
Water	25.11	26.13	25.32	34.50	30.23
RMSD Bonds (Å)	0.005	0.005	0.005	0.004	0.005
RMSD Angles (°)	0.732	0.768	0.792	0.749	0.783
Ramachandran statistics					
Outliers (%)	0.00	0.00	0.00	0.55	0.00
Favored (%)	97.58	98.10	99.03	98.35	98.10
Allowed (%)	2.42	1.90	0.97	1.10	1.90
Number of protein atoms	1821	1785	1801	1765	1810
Number of water atoms	268	287	376	319	332
Number of ligand/ion atoms	2	27	26	28	26
Occupancy of inhibitor atoms	N/A	80%	75%	81%	67% (53% for C23 and C24)

Supplementary Table 6. Spectroscopic data of compound **E-64 (1)**

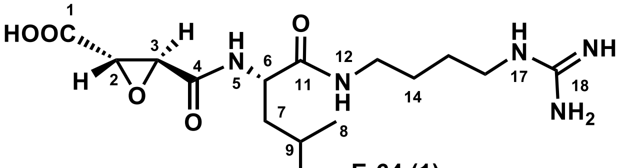

	E-64 (**1**) in DMSO-d_6	
Position	δ_H (*J* in Hz)	Position
1		170.8, C
2	3.02, d (1.9)	54.3, CH
3	3.35, d (1.8)	52.1, CH
4		167.2, C
5	8.75, d (8.4)	
6	4.25, t (5.5, 8.9)	51.5, CH
7	1.43, m	40.7, CH_2
8	1.56, m	24.3, CH
9	0.83, m	21.6, CH_3
10	0.86, m	22.9, CH_3
11		171.8, C
12	8.22, t (5.8)	
13	2.98, m	37.7, CH_2
	3.09, m	
14	1.39, m	26.1, CH_2
15	1.42, m	25.7, CH_2
16	3.08, m	40.3, CH_2
17	8.46, t (5.8)	
18		157.1, C

NMR spectrum (500 MHz) for ^{1}H, NMR spectrum (125 MHz) for ^{13}C, DMSO-d_6, “m” means overlapped or multiple with other signals. Chemical shifts are reported in ppm.

HRMS (ESI, $M+H^+$) calculated for $C_{15}H_{28}N_5O_5^+$ 358.2085; found 358.2079.

$[\alpha]_D^{24.1}$ + 56° (*c* 0.1, MeOH). Compound **1** showed the same positive optical rotation as reported E-64[7].

Supplementary Table 7. Spectroscopic data of compound **CLIK-148 (3)**

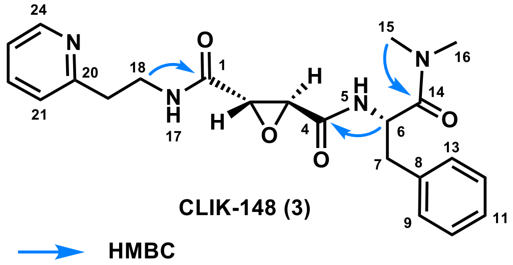

Position	**CLIK-148 (3)** in $CDCl_3$ δ_H (*J* in Hz)	δ_C, type
1		166.8, C
2	3.44, s	54.1, CH
3	3.32, m	54.0, CH
4		165.7, C
5	7.44, d (8.3)	
6	5.07, m	50.1, CH
7	2.86, m	39.3, CH_2
	2.96, m	
8		135.8, C
9	7.15, m	129.5, CH
10	7.26, m	128.7, CH
11	7.23, m	127.4, CH
12	7.26, m	128.7, CH
13	7.15, m	129.5, CH
14		170.8, C
15	2.88, s	35.9, CH_3
16	2.68, s	37.1, CH_3
17	7.97, d (6.3)	
18	3.73, m	38.5, CH_2
19	3.31, m	33.7, CH_2
20		155.4, C
21	7.79, d (8.0)	127.7, CH
22	8.28, t (7.8)	145.1, CH
23	7.74, t (6.8)	124.9, CH
24	8.72, d (5.7)	141.9, CH

NMR spectrum (500 MHz) for ^1H, NMR spectrum (125 MHz) for ^{13}C, $CDCl_3$, "m" means overlapped or multiple with other signals. Chemical shifts are reported in ppm.

HRMS (ESI, M+H$^+$) calculated for $C_{22}H_{27}N_4O_4^+$ 411.2027; found 411.2014.

$[\alpha]_D^{24.1}$ + 66° (*c* 0.1, MeOH). Compound **3** showed the same positive optical rotation as reported CLIK-148[8].

Supplementary Table 8. Spectroscopic data of compound **CPI-2 (4)**

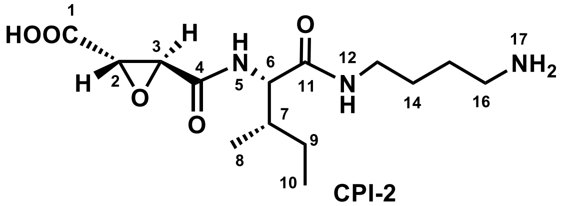

CPI-2

^{1}H NMR spectrum (500 MHz), ^{13}C NMR spectrum (125 MHz), DMSO-d_6

Position	CPI-2 (**4**) in DMSO-d_6 δ_H (J in Hz)	δ_C, type	Reported CPI-2 (**4**) in DMSO-d_6[9] δ_C, type
1		168.8, C	168.4, C
2	3.75, d (1.7)	52.5, CH	51.2, CH
3	3.46, d (1.7)	51.3, CH	52.5, CH
4		165.0, C	164.6, C
5	8.52, d (8.8)		
6	4.15 t (8.3)	57.1, CH	57.0, CH
7	1.72, d (9.8)	36.6, CH	36.6, CH
8	0.82 m	15.4, CH_3	15.4, CH_3
9	1.08, m; 1.43, m	24.4, CH_2	24.4, CH_2
10	0.82, m	10.9, CH_3	11.0, CH_3
11		170.3, C	169.9, C
12	8.17, t (5.8)		
13	3.01, m; 3.09, m	38.0, CH_2	37.9, CH_2
14	1.42, m	25.9, CH_2	25.9, CH_2
15	1.52, m	24.6, CH_2	24.6, CH_2
16	2.78, d (6.8)	38.5, CH_2	38.5, CH_2

NMR spectrum (500 MHz) for ^{1}H, NMR spectrum (125 MHz) for ^{13}C, DMSO-d_6, "m" means overlapped or multiple with other signals. Chemical shifts are reported in ppm.

HRMS (ESI, M+H$^+$) calculated for $C_{14}H_{26}N_3O_5^+$ 316.1867; found 316.1878.

$[\alpha]_D^{24.1}$ + 46° (c 0.1, MeOH). Compound **4** showed the same positive optical rotation as reported CPI-2[9].

Supplementary Table 9. Spectroscopic data of compound **CPI-3 (5)**

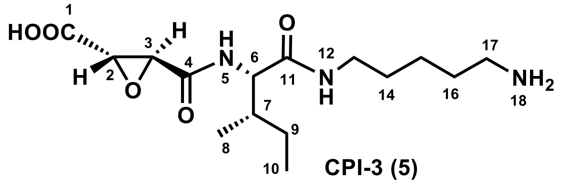

CPI-3 (5)

Position	CPI-3 (**5**) in DMSO-d_6 δ_H (J in Hz)	δ_C, type	Reported CPI-3 (**5**) in DMSO-d_6[9] δ_C, type
1		168.8, C	168.4, C
2	3.75, d (1.7)	52.5, CH	52.5, CH
3	3.46, d (1.7)	51.2, CH	51.2, CH
4		165.0, C	164.6, C
5	8.51, d (8.8)		
6	4.14 t (8.3)	57.1, CH	57.0, CH
7	1.73, m	36.6, CH	36.6, CH
8	0.82, m	15.3, CH_3	15.4, CH_3
9	1.08, m; 1.39, m	24.4, CH_2	24.4, CH_2
10	0.82, m	11.0, CH_3	11.0, CH_3
11		170.2, C	169.8, C
12	8.11, t (5.7)		
13	2.98, m; 3.10, m	38.2, CH_2	38.1, CH_2
14	1.38, m	28.3, CH_2	28.3, CH_2
15	1.26, m	23.1, CH_2	23.1, CH_2
16	1.52, m	26.6, CH_2	26.6, CH_2
17	2.74, d (6.8)	38.7, CH_2	38.7, CH_2

NMR spectrum (500 MHz) for ^{1}H, NMR spectrum (125 MHz) for ^{13}C, DMSO-d_6, "m" means overlapped or multiple with other signals. Chemical shifts are reported in ppm.

HRMS (ESI, M+H$^+$) calculated for $C_{15}H_{28}N_3O_5^+$ 330.2023; found 330.2009.

$[\alpha]_D^{24.1}$ + 42° (c 0.1, MeOH).

Compound **5** showed the same positive optical rotation as reported CPI-3[9].

Supplementary Table 10. Spectroscopic data of compound **6**

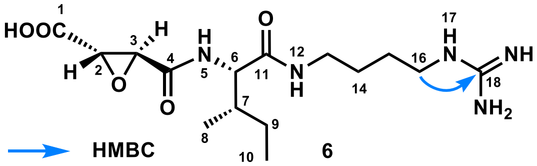

Position	**6** in DMSO-d_6 (500MHz) δ_H (J in Hz)	δ_C, type
1		168.9, C
2	3.74, d (1.9)	52.5, CH
3	3.44, d (1.8)	51.3, CH
4		165.1, C
5	8.51, d (8.8)	
6	4.15, t (8.2)	57.1, CH
7	1.71, m	36.6, CH
8	0.82, m	15.3, CH_3
9	1.07, m; 1.42, m	24.4, CH_2
10	0.82, m	10.9, CH_3
11		170.2, C
12	8.15, t (5.6)	
13	3.00, m; 3.08, m	38.0, CH_2
14	1.42, m	26.0, CH_2
15	1.42, m	26.1, CH_2
16	3.08, m	40.4, CH_2
17	7.62, brs	
18		156.7, C

NMR spectrum (500 MHz) for ^{1}H, NMR spectrum (125 MHz) for ^{13}C, DMSO-d_6, "m" means overlapped or multiple with other signals. Chemical shifts are reported in ppm.

HRMS (ESI, M+H$^+$) calculated for $C_{15}H_{28}N_5O_5^+$ 358.2085; found 358.2060.

$[\alpha]_D^{24.1}$ + 80° (c 0.1, MeOH).

Supplementary Table 11. Spectroscopic data of compound **7**

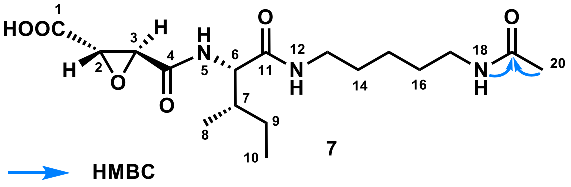

Position	**7** in DMSO-d_6 (500MHz) δ_H (J in Hz)	δ_C, type
1		168.8, C
2	3.75, d (1.7)	52.5, CH
3	3.46, d (1.7)	51.2, CH
4		164.9, C
5	8.51, d (8.9)	
6	4.16 t (8.2)	57.0, CH
7	1.71, m	36.7, CH
8	0.81, m	15.3, CH_3
9	1.07, m; 1.38, m	24.3, CH_2
10	0.81, m	11.0, CH_3
11		170.1, C
12	8.09, t (5.6)	
13	2.98, m; 3.09, m	38.4, CH_2
14	1.38, m	28.8, CH_2
15	1.23, m	23.8, CH_2
16	1.38, m	28.6, CH_2
17	2.98, m	38.4, CH_2
18	7.77 t (5.5)	
19		168.9, C
20	1.77, s	22.6, CH_3

NMR spectrum (500 MHz) for ^{1}H, NMR spectrum (125 MHz) for ^{13}C, DMSO-d_6, "m" means overlapped or multiple with other signals. Chemical shifts are reported in ppm.

HRMS (ESI, M+H$^+$) calculated for $C_{15}H_{28}N_5O_5^+$ 372.2129; found 372.2112.

$[\alpha]_D^{24.1}$ + 40° (c 0.1, MeOH).

Supplementary Table 12. Spectroscopic data of compound **8**

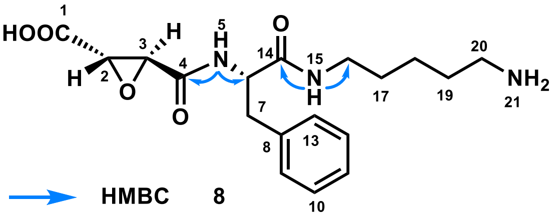

Position	**8** in DMSO-d_6 δ_H (J in Hz)	δ_C, type
1		168.7, C
2	3.59, s	52.6, CH
3	3.30, s	51.4, CH
4		165.0, C
5	8.63, d (8.5)	
6	4.49, td (5.4, 8.9)	54.2, CH
7	2.81, dd (9.4, 13.6); 3.05, m	37.8, CH_2
8		137.6, C
9	7.22, m	129.2, CH
10	7.27, m	128.1, CH
11	7.19, m	126.4, CH
12	7.27, m	128.1, CH
13	7.22, m	129.2, CH
14		170.2, C
15	8.10, t (5.6)	
16	2.96, m	38.3, CH_2
17	1.34, m	28.4, CH_2
18	1.22, m	23.1, CH_2
19	1.50, m	26.7, CH_2
20	2.73, m	38.7, CH_2

NMR spectrum (500 MHz) for ^1H, NMR spectrum (125 MHz) for ^{13}C, DMSO-d_6, "m" means overlapped or multiple with other signals. Chemical shifts are reported in ppm.

HRMS (ESI, M+H$^+$) calculated for $C_{18}H_{26}N_3O_5^+$ 364.1867; found 364.1860.

$[\alpha]_D^{24.1}$ + 136° (c 0.1, MeOH).

Supplementary Table 13. Spectroscopic data of compound **9**

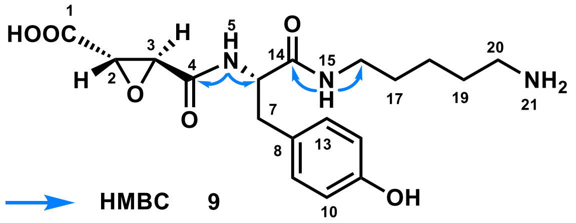

Position	**9** in DMSO-d_6 δ_H (J in Hz)	δ_C, type
1		168.7, C
2	3.59, s	52.6, CH
3	3.30, s	51.3, CH
4		164.9, C
5	8.54, d (8.5)	
6	4.39, td (5.3, 8.9)	54.5, CH
7	2.85, dd (5.3, 13.7); 3.05, m	37.1, CH_2
8		127.5, C
9	6.99, m	130.1, CH
10	6.64, m	114.9, CH
11		155.9, C
12	6.64, m	114.9, CH
13	6.99, m	130.1, CH
14		170.3, C
15	8.05, t (5.6)	
16	2.96, m	38.3, CH_2
17	1.35, m	28.4, CH_2
18	1.23, m	23.1, CH_2
19	1.50, m	26.7, CH_2
20	2.75, m	38.7, CH_2

NMR spectrum (500 MHz) for ^{1}H, NMR spectrum (125 MHz) for ^{13}C, DMSO-d_6, "m" means overlapped or multiple with other signals. Chemical shifts are reported in ppm.

HRMS (ESI, M+H$^+$) calculated for $C_{18}H_{26}N_3O_6^+$ 380.1816; found 380.1793.

$[\alpha]_D^{24.1}$ + 86° (c 0.1, MeOH).

Supplementary Table 14. Spectroscopic data of **10**

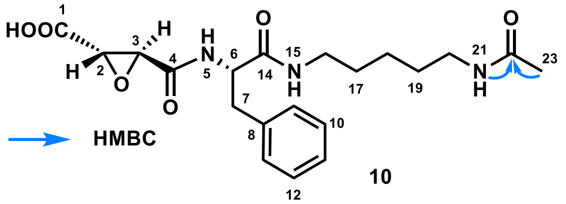

10

Position	**10** in DMSO-d_6 δ_H (J in Hz)	δ_C, type
1		168.9, C
2	3.59, s	52.6, CH
3	3.30, s	51.3, CH
4		164.9, C
5	8.61, d (8.5)	
6	4.50, td (5.3, 9.0)	54.1, CH
7	2.80, dd (9.4, 13.6); 3.05, m	37.9, CH_2
8		137.5, C
9	7.22, m	129.2, CH
10	7.26, m	128.4, CH
11	7.19, m	126.4, CH
12	7.26, m	128.4, CH
13	7.22, m	129.2, CH
14		170.1, C
15	8.07, t (5.6)	
16	2.98, m	38.4, CH_2
17	1.35, m	28.8, CH_2
18	1.19, m	23.7, CH_2
19	1.35, m	28.6, CH_2
20	2.98, m	38.5, CH_2
21	7.77, t (5.6)	
22		168.7, C
23	1.77, s	22.6, CH_3

NMR spectrum (500 MHz) for ^{1}H, NMR spectrum (125 MHz) for ^{13}C, DMSO-d_6, “m” means overlapped or multiple with other signals. Chemical shifts are reported in ppm.

HRMS (ESI, M+H$^+$) calculated for $C_{20}H_{28}N_3O_6^+$ 406.1973; found 406.1949.

$[\alpha]_D^{24.1}$ + 84° (c 0.1, MeOH).

Supplementary Table 15. Spectroscopic data of compound **12**

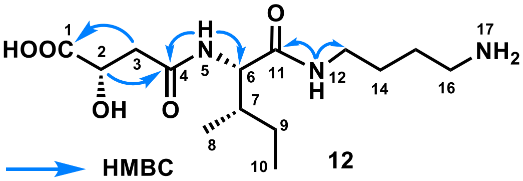

Position	**12** in DMSO-d_6 (500MHz) δ_H (J in Hz)	δ_C, type
1		172.1, C
2	4.22, d (1.7)	68.3, CH
3	2.36, m; 2.64, m	40.0, CH_2
4		172.1, C
5	7.49, d (6.0)	
6	4.14 t (8.3)	56.2, CH
7	1.67, d (9.8)	37.4, CH
8	0.82 m	15.3, CH_3
9	1.08, m; 1.43, m	24.2, CH_2
10	0.82, m	11.1, CH_3
11		170.5, C
12	8.15, m	
13	3.01, m; 3.09, m	37.9, CH_2
14	1.42, m	26.0, CH_2
15	1.52, m	24.6, CH_2
16	2.78, d (6.8)	38.6, CH_2

NMR spectrum (500 MHz) for ^1H, NMR spectrum (125 MHz) for ^{13}C, DMSO-d_6, "m" means overlapped or multiple with other signals. Chemical shifts are reported in ppm.

HRMS (ESI, M+H$^+$) calculated for $C_{14}H_{28}N_3O_5^+$ 318.2023; found 318.2000.

$[\alpha]_D^{24.1}$ + 56° (c 0.1, MeOH).

Supplementary Table 16. Spectroscopic data of compound **13**

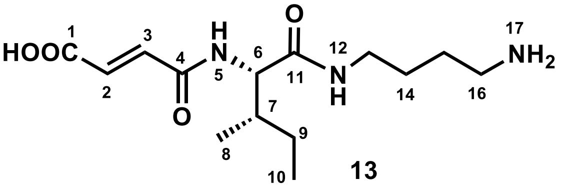

13

Position	**13** in DMSO-d_6 (500MHz) δ_H (*J* in Hz)	δ_C, type
1		166.5, C
2	7.12, d (15.5)	137.0, CH
3	6.51, d (15.5)	129.9, CH
4		163.0, C
5	8.58, d (8.8)	
6	4.19 t (8.3)	57.3, CH
7	1.74, m	36.5, CH
8	0.79 m	15.4, CH_3
9	1.09, m; 1.43, m	24.4, CH_2
10	0.79, m	10.9, CH_3
11		170.5, C
12	8.14, t (5.7)	
13	3.01, m; 3.09, m	37.9, CH_2
14	1.42, m	26.0, CH_2
15	1.52, m	24.6, CH_2
16	2.78, t (7.3)	38.5, CH_2

NMR spectrum (500 MHz) for ^{1}H, NMR spectrum (125 MHz) for ^{13}C, DMSO-d_6, "m" means overlapped or multiple with other signals. Chemical shifts are reported in ppm.

HRMS (ESI, M+H$^+$) calculated for $C_{14}H_{26}N_3O_4^+$ 300.1918; found 300.1898.

$[\alpha]_D^{24.1}$ + 98° (*c* 0.1, MeOH).

Supplementary Table 17. Spectroscopic data of (2*S*,3*S*)-*t*-ES-Ile (**14**)

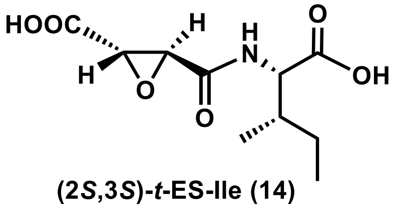

(2*S*,3*S*)-*t*-ES-Ile (14)

Position	(2*S*,3*S*)-*t*-ES-Ile (**14**) in DMSO-d_6 δ_H (*J* in Hz)	δ_C, type
1		169.0, C
2	3.67, d (1.9)	52.3, CH
3	3.35, d (1.8)	52.1, CH
4		165.9, C
5	8.54, d (8.3)	
6	4.22, dd (5.7, 8.4)	56.5, CH
7	1.81, m	36.4, CH
8	0.86, m	15.6, CH_3
9	1.19, m; 1.39, m	24.6, CH_2
10	0.86, m	11.3, CH_3
11		172.4, C

NMR spectrum (500 MHz) for ^1H, NMR spectrum (125 MHz) for ^{13}C, DMSO-d_6, "m" means overlapped or multiple with other signals. Chemical shifts are reported in ppm.

HRMS (ESI, M+H$^+$) calculated for $C_{10}H_{16}NO_6^+$ 246.0972; found 246.0946.

$[\alpha]_D^{24.1}$ + 104° (*c* 0.1, MeOH).

Supplementary Table 18. Spectroscopic data of (2*S*,3*S*)-*t*-ES-Phe (**15**)

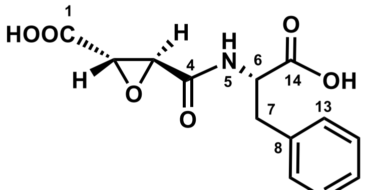

(2*S*,3*S*)-*t*-ES-Phe (15)

Position	(2*S*,3*S*)-*t*-ES-Phe (**15**) in DMSO-d_6 δ_H (*J* in Hz)	δ_C, type
1		168.6, C
2	3.59, d (1.8)	52.5, CH
3	3.30, d (1.8)	51.3, CH
4		165.3, C
5	8.67, d (8.3)	
6	4.49, m	53.4, CH
7	2.92, m; 3.10, m	36.5, CH_2
8		137.3, C
9	7.29, m	129.2, CH
10	7.22, m	128.3, CH
11	7.22, m	126.6, CH
12	7.29, m	128.3, CH
13	7.22, m	129.2, CH
14		172.3, C

NMR spectrum (500 MHz) for ^1H, NMR spectrum (125 MHz) for ^{13}C, DMSO-d_6, "m" means overlapped or multiple with other signals. Chemical shifts are reported in ppm.

HRMS (ESI, $M+H^+$) calculated for $C_{13}H_{14}NO_6^+$ 280.0816; found 280.0807.

$[\alpha]_D^{24.1}$ + 72° (*c* 0.1, MeOH).

Supplementary Table 19. Spectroscopic data of (2*S*,3*S*)-*t*-ES-Leu

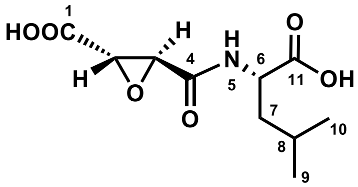

(2*S*,3*S*)-*t*-ES-Leu

Position	(2*S*,3*S*)-*t*-ES-Leu in DMSO-d_6 (500MHz) δ_H (*J* in Hz)	δ_C, type
1		168.7, C
2	3.63, d (1.9)	52.5, CH
3	3.45, d (1.8)	51.3, CH
4		165.3, C
5	8.69, d (8.0)	
6	4.26, m	50.4, CH
7	1.54, m	39.8, CH_2
8	1.62, m	24.3, CH
9	0.90, d (6.4)	22.8, CH_3
10	0.85, d (6.4)	21.2, CH_3
11		173.4, C

NMR spectrum (500 MHz) for ^1H, NMR spectrum (125 MHz) for ^{13}C, DMSO-d_6, "m" means overlapped or multiple with other signals. Chemical shifts are reported in ppm.

HRMS (ESI, M+H$^+$) calculated for $C_{10}H_{16}NO_6^+$ 246.0972; found 246.0965.

$[\alpha]_D^{24.1}$ + 90° (*c* 0.1, MeOH).

Supplementary Table 20. Spectroscopic data of (2*S*,3*S*)-*t*-ES-Val

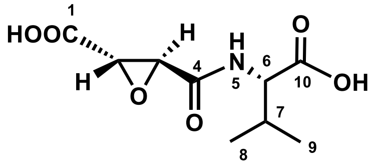

(2*S*,3*S*)-*t*-ES-Val

	(2*S*,3*S*)-*t*-ES-Val in DMSO-d_6 (500MHz)	
Position	δ_H (*J* in Hz)	δ_C, type
1		168.8, C
2	3.79, d (1.9)	52.4, CH
3	3.47, d (1.8)	51.2, CH
4		165.4, C
5	8.64, d (8.4)	
6	4.20, dd (5.6, 8.5)	57.4, CH
7	2.09, m	29.9, CH
8	0.89, m	17.8, CH_3
9	0.89, m	19.1, CH_3
10		172.4, C

NMR spectrum (500 MHz) for ^{1}H, NMR spectrum (125 MHz) for ^{13}C, DMSO-d_6, "m" means overlapped or multiple with other signals. Chemical shifts are reported in ppm.

HRMS (ESI, M+H$^+$) calculated for $C_{10}H_{16}NO_6^+$ 232.0816; found 232.0820.
$[\alpha]_D^{24.1}$ + 152° (*c* 0.1, MeOH).

Supplementary Table 21. Spectroscopic data of (2*S*,3*S*)-*t*-ES-Tyr

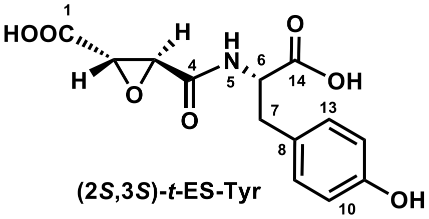

	(2*S*,3*S*)-*t*-ES-Tyr in DMSO-d_6 (500MHz)	
Position	δ_H (J in Hz)	δ_C, type
1		168.6, C
2	3.61, d (1.8)	52.5, CH
3	3.33, d (1.8)	51.3, CH
4		165.1, C
5	8.60, d (8.2)	
6	4.40, m	53.7, CH
7	2.79, dd (9.4, 13.9); 2.97, dd (4.8, 13.9)	35.8, CH_2
8		127.0, C
9	7.00, m	130.1, CH
10	6.66, m	115.0, CH
11		156.0, C
12	6.66, m	115.0, CH
13	7.00, m	130.1, CH
14		172.4, C

NMR spectrum (500 MHz) for ^1H, NMR spectrum (125 MHz) for ^{13}C, DMSO-d_6, “m” means overlapped or multiple with other signals. Chemical shifts are reported in ppm.

HRMS (ESI, M+H$^+$) calculated for $C_{13}H_{14}NO_7^+$ 296.0765; found 296.0777.

$[\alpha]_D^{24.1}$ + 72° (*c* 0.1, MeOH).

Supplementary Table 22. Spectroscopic data of (2*S*,3*S*)-*t*-ES-Trp

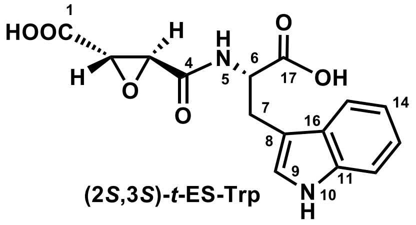

Position	(2*S*,3*S*)-*t*-ES-Trp in DMSO-d_6 (500MHz)	
	δ_H (*J* in Hz)	δ_C, type
1		168.6, C
2	3.63, d (1.8)	52.5, CH
3	3.34, d (1.9)	51.3, CH
4		165.2, C
5	8.64, d (8.0)	
6	4.52, td (4.9, 8.2)	53.1, CH
7	3.08, dd (4.0, 14.7); 3.20, dd (8.5, 14.7)	26.9, CH_2
8		136.1, C
9	7.15, d (2.3)	123.7, CH
10	10.88, d (2.4)	
11		136.1, C
12	7.34, d (8.1)	111.5, CH
13	7.07, m	121.0, CH
14	6.99, m	118.5, CH
15	7.53, d (7.9)	118.2, CH
16		127.2, C
17		172.7, C

NMR spectrum (500 MHz) for ^{1}H, NMR spectrum (125 MHz) for ^{13}C, DMSO-d_6, "m" means overlapped or multiple with other signals. Chemical shifts are reported in ppm.

HRMS (ESI, M+H$^+$) calculated for $C_{15}H_{15}N_2O_6^+$ 319.0925; found 319.0931.

$[\alpha]_D^{24.1}$ + 52° (*c* 0.1, MeOH).

Supplementary Table 23. Spectroscopic data of (2*S*,3*S*)-*t*-ES-a1

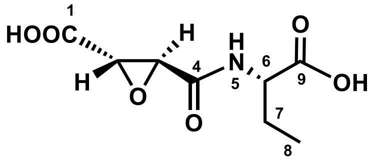

(2*S*,3*S*)-*t*-ES-a1

	(2*S*,3*S*)-*t*-ES-a1 in DMSO-d_6 (500MHz)	
Position	δ_H (*J* in Hz)	δ_C, type
1		168.7, C
2	3.69, d (1.8)	52.5, CH
3	3.47, d (1.8)	51.2, CH
4		165.3, C
5	8.68, d (7.7)	
6	4.18, dd (5.1, 8.1)	53.3, CH
7	1.65, m; 1.75, m	24.2, CH_2
8	0.88, m	10.2, CH_3
9		172.8, C

NMR spectrum (500 MHz) for ^{1}H, NMR spectrum (125 MHz) for ^{13}C, DMSO-d_6, "m" means overlapped or multiple with other signals. Chemical shifts are reported in ppm.

HRMS (ESI, M+H$^+$) calculated for $C_8H_{12}NO_6^+$ 218.0659; found 218.0645.

$[\alpha]_D^{24.1}$ + 56° (*c* 0.1, MeOH).

Supplementary Table 24. Spectroscopic data of (2*S*,3*S*)-*t*-ES-a2

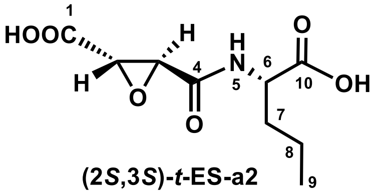

(2*S*,3*S*)-*t*-ES-a2

Position	(2*S*,3*S*)-*t*-ES-a2 in DMSO-d_6 (500MHz) δ_H (*J* in Hz)	δ_C, type
1		168.7, C
2	3.67, d (1.8)	52.5, CH
3	3.47, d (1.8)	51.2, CH
4		165.3, C
5	8.70, d (7.8)	
6	4.23, dd (5.0, 8.5)	51.7, CH
7	1.62, m; 1.69, m	32.9, CH_2
8	1.32, m	18.5, CH_2
9	0.87, m	13.5, CH_3
10		173.0, C

NMR spectrum (500 MHz) for ^{1}H, NMR spectrum (125 MHz) for ^{13}C, DMSO-d_6, "m" means overlapped or multiple with other signals. Chemical shifts are reported in ppm.

HRMS (ESI, M+H$^+$) calculated for $C_9H_{14}NO_6^+$ 232.0816; found 232.0819.
$[\alpha]_D^{24.1}$ + 48° (*c* 0.1, MeOH).

Supplementary Table 25. Spectroscopic data of (2*S*,3*S*)-*t*-ES-a3

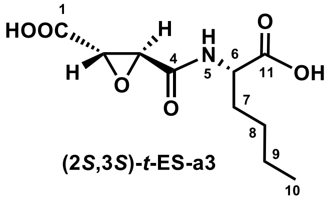

	(2*S*,3*S*)-*t*-ES-a3 in DMSO-d_6 (500MHz)	
Position	δ_H (*J* in Hz)	δ_C, type
1		168.7, C
2	3.67, d (1.8)	52.5, CH
3	3.46, d (1.8)	51.4, CH
4		165.3, C
5	8.69, d (7.8)	
6	4.22, dd (4.9, 8.3)	52.0, CH
7	1.63, m; 1.72, m	30.6, CH_2
8	1.27, m	27.4, CH_2
9	1.27, m	21.7, CH_2
10	0.86, m	13.8, CH_3
11		173.0, C

NMR spectrum (500 MHz) for ^1H, NMR spectrum (125 MHz) for ^{13}C, DMSO-d_6, "m" means overlapped or multiple with other signals. Chemical shifts are reported in ppm.

HRMS (ESI, M+H$^+$) calculated for $C_{10}H_{16}NO_6^+$ 246.0972; found 246.0967.

$[\alpha]_D^{24.1}$ + 70 (*c* 0.1, MeOH).

Supplementary Table 26. Spectroscopic data of (2*S*,3*S*)-*t*-ES-a4

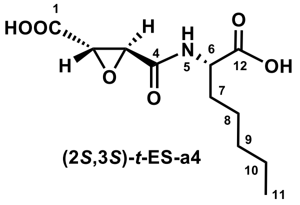

(2*S*,3*S*)-*t*-ES-a4

Position	(2*S*,3*S*)-*t*-ES-a4 in DMSO-d_6 (500MHz) δ_H (*J* in Hz)	δ_C, type
1		168.7, C
2	3.67, d (1.8)	52.5, CH
3	3.45, d (1.8)	51.3, CH
4		165.3, C
5	8.69, d (7.8)	
6	4.22, dd (4.9, 8.3)	52.0, CH
7	1.62, m; 1.71, m	30.8, CH_2
8	1.27, m	30.7, CH_2
9	1.27, m	24.9, CH_2
10	1.27, m	21.9, CH_2
11	0.86, t (6.8)	13.9, CH_3
12		173.0, C

NMR spectrum (500 MHz) for ^{1}H, NMR spectrum (125 MHz) for ^{13}C, DMSO-d_6, "m" means overlapped or multiple with other signals. Chemical shifts are reported in ppm.

HRMS (ESI, M+H$^+$) calculated for $C_{11}H_{18}NO_6^+$ 260.1129; found 260.1133.

$[\alpha]_D^{24.1}$ + 64° (*c* 0.1, MeOH).

Supplementary Table 27. Spectroscopic data of (2*S*,3*S*)-*t*-ES-a5

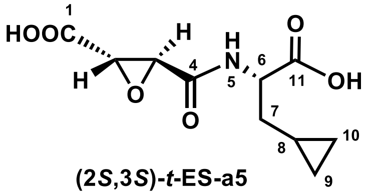

(2*S*,3*S*)-*t*-ES-a5

	(2*S*,3*S*)-*t*-ES-a5 in DMSO-d_6 (500MHz)	
Position	δ_H (*J* in Hz)	δ_C, type
1		168.7, C
2	3.69, d (1.8)	52.6, CH
3	3.48, d (1.8)	51.3, CH
4		165.2, C
5	8.72, d (7.9)	
6	4.30, dd (5.4, 8.0)	52.5, CH
7	1.60, m	35.7, CH_2
8	0.75, m	7.7, CH
9	0.40, m	4.5, CH_2
10	0.04, m; 0.14, m	3.9, CH_2
11		172.9, C

NMR spectrum (500 MHz) for ^{1}H, NMR spectrum (125 MHz) for ^{13}C, DMSO-d_6, "m" means overlapped or multiple with other signals. Chemical shifts are reported in ppm.

HRMS (ESI, M+H$^+$) calculated for $C_{10}H_{14}NO_6^+$ 244.0816; found 244.0788.

$[\alpha]_D^{24.1}$ + 120° (*c* 0.1, MeOH).

Supplementary Table 28. Spectroscopic data of (2*S*,3*S*)-*t*-ES-a6

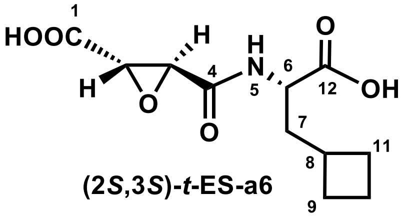

Position	(2*S*,3*S*)-*t*-ES-a6 in DMSO-d_6 (500MHz)	
	δ_H (*J* in Hz)	δ_C, type
1		169.1, C
2	3.65, d (2.0)	52.9, CH
3	3.46, d (1.9)	51.7, CH
4		165.6, C
5	8.65, d (8.2)	
6	4.14, m	51.1, CH
7	1.75, m; 1.81, m	38.3, CH_2
8	2.32, m	32.8, CH
9	1.62, m; 1.97, m	28.0, CH_2
10	1.76, m	18.5, CH_2
11	1.62, m; 1.97, m	28.2, CH_2
12		173.5, C

NMR spectrum (500 MHz) for ^{1}H, NMR spectrum (125 MHz) for ^{13}C, DMSO-d_6, "m" means overlapped or multiple with other signals. Chemical shifts are reported in ppm.

HRMS (ESI, M+H$^+$) calculated for $C_{11}H_{16}NO_6^+$ 258.0972; found 258.0965.
$[\alpha]_D^{24.1}$ + 100° (*c* 0.1, MeOH).

Supplementary Table 29. Spectroscopic data of (2*S*,3*S*)-*t*-ES-a7

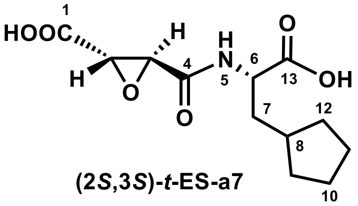

Position	(2*S*,3*S*)-*t*-ES-a7 in DMSO-d_6 (500MHz) δ_H (*J* in Hz)	δ_C, type
1		168.7, C
2	3.64, d (1.8)	52.5, CH
3	3.45, d (1.8)	51.6, CH
4		165.3, C
5	8.70, d (7.9)	
6	4.22, m	51.3, CH
7	1.69, m	37.1, CH_2
8	1.82, m	36.3, CH
9	1.72, m	32.2, CH_2
10	1.08, m	31.7, CH_2
11	1.56, m	24.7, CH_2
12	1.47, m	24.5, CH_2
13		173.3, C

NMR spectrum (500 MHz) for ^{1}H, NMR spectrum (125 MHz) for ^{13}C, DMSO-d_6, "m" means overlapped or multiple with other signals. Chemical shifts are reported in ppm.

HRMS (ESI, M+H$^+$) calculated for $C_{12}H_{17}NO_6^+$ 272.1129; found 272.1134.

$[\alpha]_D^{24.1}$ + 44° (*c* 0.1, MeOH).

Supplementary Table 30. Spectroscopic data of (2*S*,3*S*)-*t*-ES-a8

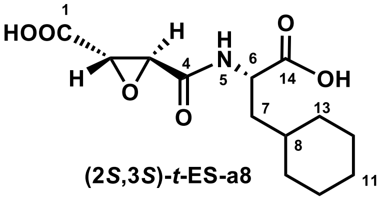

(2*S*,3*S*)-*t*-ES-a8

	(2*S*,3*S*)-*t*-ES-a8 in DMSO-d_6 (500MHz)	
Position	δ_H (*J* in Hz)	δ_C, type
1		168.7, C
2	3.64, d (1.8)	52.5, CH
3	3.44, d (1.8)	51.3, CH
4		165.3, C
5	8.70, d (7.9)	
6	4.28, m	49.7, CH
7	1.57, m	38.3, CH_2
8	1.31, m	33.6, CH
9	0.93, m; 1.64, m	33.1, CH_2
10	1.60, m	26.0, CH_2
11	1.13, m	25.7, CH_2
12	0.83, m; 1.69, m	31.5, CH_2
13	1.65, m	25.5, CH_2
14		173.5, C

NMR spectrum (500 MHz) for ^{1}H, NMR spectrum (125 MHz) for ^{13}C, DMSO-d_6, "m" means overlapped or multiple with other signals. Chemical shifts are reported in ppm.

HRMS (ESI, M+H$^+$) calculated for $C_{13}H_{20}NO_6^+$ 286.1285; found 286.1283.

$[\alpha]_D^{24.1}$ + 48° (*c* 0.1, MeOH).

Supplementary Table 31. Spectroscopic data of (2*S*,3*S*)-*t*-ES-a9

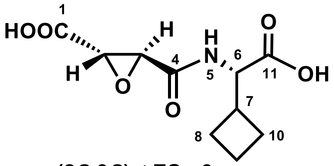

(2*S*,3*S*)-*t*-ES-a9

	(2*S*,3*S*)-*t*-ES-a9 in DMSO-d_6 (500MHz)	
Position	δ_H (J in Hz)	δ_C, type
1		168.7, C
2	3.72, d (1.8)	52.4, CH
3	3.46, d (1.8)	51.2, CH
4		165.4, C
5	8.70, d (8.0)	
6	4.22, t (8.2)	56.0, CH
7	2.63, m	36.3, CH
8	1.82, m	24.8, CH_2
9	1.94, m	24.5, CH_2
10	1.74, m	17.4, CH_2
11		172.0, C

NMR spectrum (500 MHz) for ^1H, NMR spectrum (125 MHz) for ^{13}C, DMSO-d_6, "m" means overlapped or multiple with other signals. Chemical shifts are reported in ppm.

HRMS (ESI, M+H$^+$) calculated for $C_{10}H_{14}NO_6^+$ 244.0816; found 244.0815.

$[\alpha]_D^{24.1}$ + 68° (c 0.1, MeOH).

Supplementary Table 32. Spectroscopic data of (2*S*,3*S*)-*t*-ES-a10

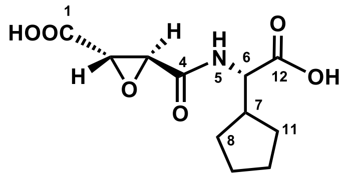

(2*S*,3*S*)-*t*-ES-a10

Position	(2*S*,3*S*)-*t*-ES-a10 in DMSO-d_6 (500MHz) δ_H (*J* in Hz)	δ_C, type
1		168.8, C
2	3.73, d (1.8)	52.4, CH
3	3.46, d (1.8)	51.2, CH
4		165.3, C
5	8.73, d (8.2)	
6	4.18, m	55.5, CH
7	2.21, m	41.1, CH
8	1.66, m	28.3, CH_2
9	1.49, m	24.8, CH_2
10	1.56, m	24.5, CH_2
11	1.29, m	28.6, CH_2
12		172.7, C

NMR spectrum (500 MHz) for ^{1}H, NMR spectrum (125 MHz) for ^{13}C, DMSO-d_6, "m" means overlapped or multiple with other signals. Chemical shifts are reported in ppm.

HRMS (ESI, M+H$^+$) calculated for $C_{11}H_{16}NO_6^+$ 258.0972; found 258.0964.

$[\alpha]_D^{24.1}$ + 104° (*c* 0.1, MeOH).

Supplementary Table 33. Spectroscopic data of (2*S*,3*S*)-*t*-ES-a11

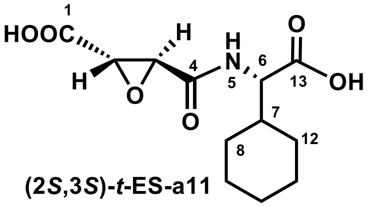

Position	(2*S*,3*S*)-*t*-ES-a11 in DMSO-d_6 (500MHz) δ_H (*J* in Hz)	δ_C, type
1		168.8, C
2	3.77, d (1.8)	52.4, CH
3	3.46, d (1.8)	51.1, CH
4		165.3, C
5	8.65, d (8.4)	
6	4.18, m	57.0, CH
7	1.71, m	39.0, CH
8	1.01, m; 1.59, m	29.1, CH_2
9	1.59, m	25.5, CH_2
10	1.66, m	25.6, CH_2
11	1.12, m	25.5, CH_2
12	1.10, m; 1.56, m	27.9, CH_2
13		172.3, C

NMR spectrum (500 MHz) for ^{1}H, NMR spectrum (125 MHz) for ^{13}C, DMSO-d_6, "m" means overlapped or multiple with other signals. Chemical shifts are reported in ppm.

HRMS (ESI, M+H$^+$) calculated for $C_{123}H_{18}NO_6^+$ 272.1129; found 272.1123.

$[\alpha]_D^{24.1}$ + 76° (*c* 0.1, MeOH).

Supplementary Table 34. Spectroscopic data of (2*S*,3*S*)-*t*-ES-a12

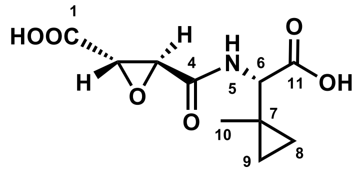

(2*S*,3*S*)-*t*-ES-a12

Position	(2*S*,3*S*)-*t*-ES-a12 in DMSO-d_6 (500MHz) δ_H (*J* in Hz)	δ_C, type
1		168.8, C
2	3.82, d (1.8)	52.3, CH
3	3.46, d (1.8)	51.1, CH
4		165.1, C
5	8.65, d (8.2)	
6	3.73, m	59.1, CH
7		17.7, C
8	0.52, m; 0.69, m	11.6, CH_2
9	0.32, m; 0.41, m	12.0, CH_2
10	1.04, s	19.2, CH_3
11		171.6, C

NMR spectrum (500 MHz) for ^{1}H, NMR spectrum (125 MHz) for ^{13}C, DMSO-d_6, "m" means overlapped or multiple with other signals. Chemical shifts are reported in ppm.

HRMS (ESI, M+H$^+$) calculated for $C_{10}H_{14}NO_6^+$ 244.0816; found 244.0810.

$[\alpha]_D^{24.1}$ + 132° (*c* 0.1, MeOH).

Supplementary Table 35. Spectroscopic data of (2*S*,3*S*)-*t*-ES-a13

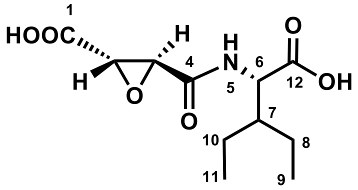

(2*S*,3*S*)-*t*-ES-a13

	(2*S*,3*S*)-*t*-ES-a13 in DMSO-d_6 (500MHz)	
Position	δ_H (*J* in Hz)	δ_C, type
1		168.8, C
2	3.81, d (1.8)	53.4, CH
3	3.46, d (1.8)	52.3, CH
4		165.5, C
5	8.58, d (8.2)	
6	4.44, m	51.2, CH
7	1.67, m	43.1, CH
8	1.18, m; 1.32, m	22.0, CH_2
9	0.85, t (7.3)	11.4, CH_3
10	1.32, m	22.2, CH_2
11	0.85, t (7.3)	11.5, CH_3
12		173.0, C

NMR spectrum (500 MHz) for ^1H, NMR spectrum (125 MHz) for ^{13}C, DMSO-d_6, "m" means overlapped or multiple with other signals. Chemical shifts are reported in ppm.

HRMS (ESI, M+H$^+$) calculated for $C_{11}H_{18}NO_6^+$ 260.1129; found 260.1105.

$[\alpha]_D^{24.1}$ + 138° (*c* 0.1, MeOH).

Supplementary Table 36. Spectroscopic data of (2*S*,3*S*)-*t*-ES-a14

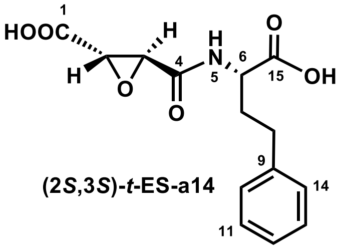

(2*S*,3*S*)-*t*-ES-a14

	(2*S*,3*S*)-*t*-ES-a14 in DMSO-d_6 (500MHz)	
Position	δ_H (*J* in Hz)	δ_C, type
1		169.2, C
2	3.65, d (1.9)	53.0, CH
3	3.45, m	52.0, CH
4		166.2, C
5	8.73, d (7.9)	
6	4.20, m	52.2, CH
7	1.93, m; 2.01, m	33.1, CH_2
8	2.61, m	31.8, CH_2
9		141.3, C
10	7.28, m	128.8, CH
11	7.19, m	128.8, CH
12	7.19, m	126.4, CH
13	7.28, m	128.8, CH
14	7.19, m	128.8, CH
15		173.4, C

NMR spectrum (500 MHz) for ^{1}H, NMR spectrum (125 MHz) for ^{13}C, DMSO-d_6, "m" means overlapped or multiple with other signals. Chemical shifts are reported in ppm.

HRMS (ESI, M+H$^+$) calculated for $C_{14}H_{16}NO_6^+$ 294.0972; found 294.0957.

$[\alpha]_D^{24.1}$ + 84° (*c* 0.1, MeOH).

Supplementary Table 37. Spectroscopic data of (2*S*,3*S*)-*t*-ES-a15

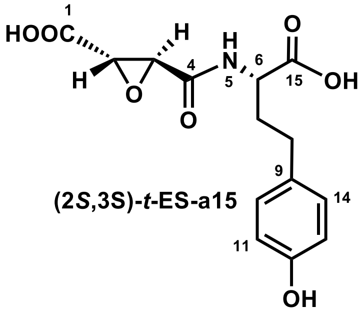

(2*S*,3S)-*t*-ES-a15

Position	(2*S*,3*S*)-*t*-ES-a15 in DMSO-d_6 (500MHz) δ_H (*J* in Hz)	δ_C, type
1		168.7, C
2	3.67, d (1.9)	52.6, CH
3	3.48, m	51.4, CH
4		165.5, C
5	8.74, d (7.9)	
6	4.17, m	51.4, CH
7	1.83, m; 1.95, m	33.0, CH_2
8	2.44, m	30.5, CH_2
9		130.8, C
10	6.97, m	129.2, CH
11	6.66, m	115.1, CH
12		155.5, C
13	6.66, m	115.1, CH
14	6.97, m	129.2, CH
15		173.0, C

NMR spectrum (500 MHz) for ^{1}H, NMR spectrum (125 MHz) for ^{13}C, DMSO-d_6, "m" means overlapped or multiple with other signals. Chemical shifts are reported in ppm.

HRMS (ESI, M+H$^+$) calculated for $C_{14}H_{16}NO_7^+$ 310.0921; found 310.0924.

$[\alpha]_D^{24.1}$ + 56° (*c* 0.1, MeOH)

Supplementary Table 38. Spectroscopic data of (2*S*,3*S*)-*t*-ES-a16

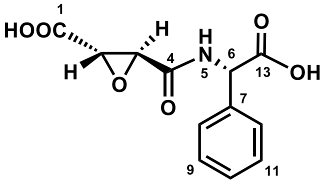

(2*S*,3*S*)-*t*-ES-a16

	(2*S*,3*S*)-*t*-ES-a16 in DMSO-d_6 (500MHz)	
Position	δ_H (*J* in Hz)	δ_C, type
1		168.7, C
2	3.79, d (1.9)	52.3, CH
3	3.50, d (1.9)	51.3, CH
4		165.0, C
5	9.26, d (7.3)	
6	5.37, d (7.3)	56.5, CH
7		136.7, C
8	7.39, m	127.6, CH
9	7.39, m	128.7, CH
10	7.35, m	128.2, CH
11	7.39, m	128.7, CH
12	7.39, m	127.6, CH
13		171.3, C

NMR spectrum (500 MHz) for ^1H, NMR spectrum (125 MHz) for ^{13}C, DMSO-d_6, "m" means overlapped or multiple with other signals. Chemical shifts are reported in ppm.

HRMS (ESI, M+H$^+$) calculated for $C_{12}H_{12}NO_6^+$ 266.0659; found 266.0658.

$[\alpha]_D^{24.1}$ + 114° (*c* 0.1, MeOH)

Supplementary Table 39. Spectroscopic data of (2*S*,3*S*)-*t*-ES-a17

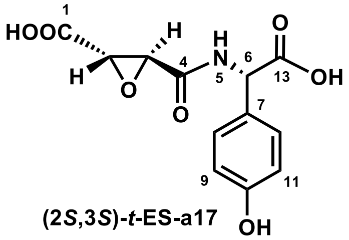

(2*S*,3*S*)-*t*-ES-a17

	(2*S*,3*S*)-*t*-ES-a17 in DMSO-d_6 (500MHz)	
Position	δ_H (*J* in Hz)	δ_C, type
1		168.8, C
2	3.77, d (1.9)	52.3, CH
3	3.48, d (1.9)	51.2, CH
4		164.8, C
5	9.13, d (7.3)	
6	5.21, d (7.3)	56.0, CH
7		126.6, C
8	7.19, m	128.9, CH
9	6.76, m	115.4, CH
10		157.4, C
11	6.76, m	115.4, CH
12	7.19, m	128.9, CH
13		171.7, C

NMR spectrum (500 MHz) for ^{1}H, NMR spectrum (125 MHz) for ^{13}C, DMSO-d_6, "m" means overlapped or multiple with other signals. Chemical shifts are reported in ppm.

HRMS (ESI, M+H$^+$) calculated for $C_{12}H_{12}NO_7^+$ 282.0608; found 282.0605.

$[\alpha]_D^{24.1}$ + 97° (*c* 0.1, MeOH)

Supplementary Table 40. Spectroscopic data of (2*S*,3*S*)-*t*-ES-a18

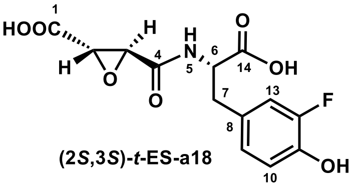

Position	(2*S*,3*S*)-*t*-ES-a18 in DMSO-d_6 (500MHz) δ_H (*J* in Hz)	δ_C, type
1		168.6, C
2	3.58, d (1.9)	52.4, CH
3	3.30, d (1.9)	51.5, CH
4		165.3, C
5	8.60, d (7.3)	
6	4.42, m	53.4, CH
7	2.80, dd (9.5, 13.9); 2.98, dd (4.9, 13.9)	35.5, CH_2
8		128.5 d (6.07), C
9	6.98, dd (1.8, 12.4)	116.7 d (18.14), CH
10	6.84, m	117.4 d (3.22), CH
11		143.4 d (12.17), C
12		150.6 d (240.17), C
13	6.84, m	125.2 d (2.99), CH
14		172.3, C

NMR spectrum (500 MHz) for ^{1}H, NMR spectrum (125 MHz) for ^{13}C, DMSO-d_6, "m" means overlapped or multiple with other signals. Chemical shifts are reported in ppm.

HRMS (ESI, M+H$^+$) calculated for $C_{13}H_{13}NO_7F^+$ 314.0671; found 314.0668.

$[\alpha]_D^{24.1}$ + 138° (*c* 0.1, MeOH)

Supplementary Table 41. Spectroscopic data of (2*S*,3*S*)-*t*-ES-a19

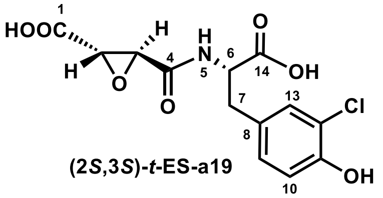

Position	(2*S*,3*S*)-*t*-ES-a19 in DMSO-d_6 (500MHz) δ_H (*J* in Hz)	δ_C, type
1		169.0, C
2	3.59, d (1.9)	52.9, CH
3	3.32, d (1.9)	51.9, CH
4		165.7, C
5	8.62, d (8.3)	
6	4.41, m	53.9, CH
7	2.80, dd (9.4, 13.9); 2.98, dd (4.9, 13.9)	35.7, CH_2
8		129.4, C
9	6.97, dd (2.1, 8.3)	129.2, CH
10	6.86, d (8.2)	116.8, CH
11		152.1, C
12		119.6, C
13	7.18, d (2.1)	130.8, CH
14		172.7, C

NMR spectrum (125 MHz) for ^{13}C, DMSO-d_6, "m" means overlapped or multiple with other signals. Chemical shifts are reported in ppm.

HRMS (ESI, M+H$^+$) calculated for $C_{13}H_{13}NO_7Cl^+$ 330.0375; found 330.0376.

$[\alpha]_D^{24.1}$ + 130° (*c* 0.1, MeOH)

Supplementary Table 42. Spectroscopic data of (2*S*,3*S*)-*t*-ES-a20

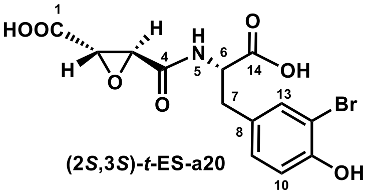

(2*S*,3*S*)-*t*-ES-a20

	(2*S*,3*S*)-*t*-ES-a20 in DMSO-d_6 (500MHz)	
Position	δ_H (*J* in Hz)	δ_C, type
1		168.6, C
2	3.59, d (1.8)	52.5, CH
3	3.31, d (1.8)	51.5, CH
4		165.3, C
5	8.61, d (8.3)	
6	4.41, m	53.5, CH
7	2.80, dd (9.4, 13.9); 2.98, dd (4.9, 13.9)	35.2, CH_2
8		129.4, C
9	7.02, dd (2.1, 8.2)	129.4, CH
10	6.85, d (8.2)	116.1, CH
11		152.7, C
12		108.9, C
13	7.33, d (2.1)	133.3, CH
14		172.2, C

NMR spectrum (500 MHz) for ^{1}H, NMR spectrum (125 MHz) for ^{13}C, DMSO-d_6, "m" means overlapped or multiple with other signals. Chemical shifts are reported in ppm.

HRMS (ESI, M+H$^+$) calculated for $C_{13}H_{13}NO_7Br^+$ 373.9870; found 373.9874.

$[\alpha]_D^{24.1}$ + 132 (*c* 0.1, MeOH)

Supplementary Table 43. Spectroscopic data of (2*S*,3*S*)-*t*-ES-a21

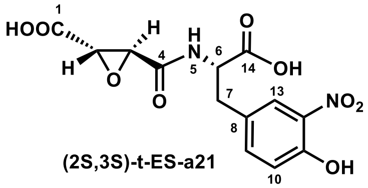

(2S,3S)-t-ES-a21

Position	(2*S*,3*S*)-*t*-ES-a21 in DMSO-d_6 (500MHz) δ_H (*J* in Hz)	δ_C, type
1		168.5, C
2	3.58, d (1.9)	52.5, CH
3	3.32, d (1.9)	51.4, CH
4		165.4, C
5	8.66, d (8.4)	
6	4.48, m	53.1, CH
7	2.89, dd (9.4, 13.9); 3.08, dd (4.9, 13.9)	35.1, CH_2
8		128.5, C
9	7.41, dd (2.2, 8.6)	136.2, CH
10	7.06, d (8.5)	119.0, CH
11		150.9, C
12		136.3, C
13	7.76, d (2.2)	125.5, CH
14		172.0, C

NMR spectrum (500 MHz) for ^1H, NMR spectrum (125 MHz) for ^{13}C, DMSO-d_6, "m" means overlapped or multiple with other signals. Chemical shifts are reported in ppm.

HRMS (ESI, M+H$^+$) calculated for $C_{13}H_{13}N_2O_9^+$ 341.0616; found 341.0619.

$[\alpha]_D^{24.1}$ + 138 (*c* 0.1, MeOH)

Supplementary Table 44. Spectroscopic data of (2*S*,3*S*)-*t*-ES-a22

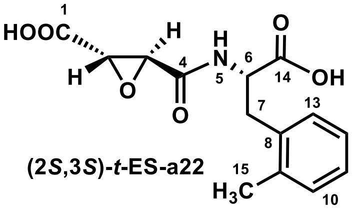

Position	(2*S*,3*S*)-*t*-ES-a22 in DMSO-d_6 (500MHz)	
	δ_H (*J* in Hz)	δ_C, type
1		168.5, C
2	3.57, d (1.8)	52.5, CH
3	3.30, d (1.8)	51.3, CH
4		165.3, C
5	8.75, d (8.4)	
6	4.48, m	52.0, CH
7	2.88, dd (9.9, 14.1); 3.14, dd (4.9, 14.2)	34.2, CH_2
8		136.1, C
9		135.5, C
10	7.11, m	130.1, CH
11	7.11, m	126.7, CH
12	7.11, m	125.6, CH
13	7.12, m	129.7, CH
14		172.5, C
15	2.29, s	18.9, CH_3

NMR spectrum (500 MHz) for ^1H, NMR spectrum (125 MHz) for ^{13}C, DMSO-d_6, "m" means overlapped or multiple with other signals. Chemical shifts are reported in ppm.

HRMS (ESI, M+H$^+$) calculated for $C_{14}H_{16}NO_6^+$ 294.0972; found 294.09812.

$[\alpha]_D^{24.1}$ + 90º (*c* 0.1, MeOH).

Supplementary Table 45. Spectroscopic data of (2*S*,3*S*)-*t*-ES-a23

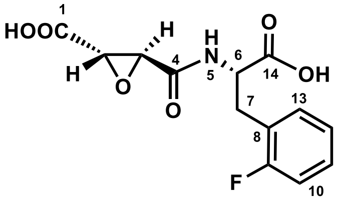

(2*S*,3*S*)-*t*-ES-a23

	(2*S*,3*S*)-*t*-ES-a23 in DMSO-d_6 (500MHz)	
Position	δ_H (*J* in Hz)	δ_C, type
1		168.5, C
2	3.58, d (1.8)	52.5, CH
3	3.30, d (1.8)	51.3, CH
4		165.3, C
5	8.70, d (8.4)	
6	4.53, m	51.9, CH
7	2.92, dd (9.5, 13.9); 3.19, m	30.2, CH_2
8		124.0, C, d (15.4)
9		160.8, C, d (244.2)
10	7.14, m	115.1, CH, d (21.6)
11	7.29, m	128.9, CH, d (8.1)
12	7.13, m	124.2, CH, d (3.3)
13	7.29, m	131.8, CH, d (4.5)
14		172.0, C

NMR spectrum (500 MHz) for ^{1}H, NMR spectrum (125 MHz) for ^{13}C, DMSO-d_6, "m" means overlapped or multiple with other signals. Chemical shifts are reported in ppm.

HRMS (ESI, M+H$^+$) calculated for $C_{13}H_{13}NO_6F^+$ 298.0721; found 298.0723.

$[\alpha]_D^{24.1}$ + 74º (*c* 0.1, MeOH).

Supplementary Table 46. Spectroscopic data of (2*S*,3*S*)-*t*-ES-a24

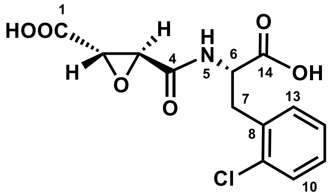

(2*S*,3*S*)-*t*-ES-a24

	(2*S*,3*S*)-*t*-ES-a24 in DMSO-d_6 (500MHz)	
Position	δ_H (*J* in Hz)	δ_C, type
1		168.5, C
2	3.54, d (1.8)	52.5, CH
3	3.29, m	51.3, CH
4		165.3, C
5	8.73, d (8.6)	
6	4.59, m	51.4, CH
7	2.98, dd (10.3, 13.9); 3.32, m	34.5, CH_2
8		134.9, C
9		133.3, C
10	7.27, m	127.0, CH
11	7.27, m	128.7, CH
12	7.42, m	129.3, CH
13	7.33, m	131.8, CH
14		172.1, C

NMR spectrum (500 MHz) for ^{1}H, NMR spectrum (125 MHz) for ^{13}C, DMSO-d_6, "m" means overlapped or multiple with other signals. Chemical shifts are reported in ppm.

HRMS (ESI, M+H$^+$) calculated for $C_{13}H_{13}NO_6Cl^+$ 314.0426; found 314.0434.

$[\alpha]_D^{24.1}$ + 64 (*c* 0.1, MeOH).

Supplementary Table 47. Spectroscopic data of (2*S*,3*S*)-*t*-ES-a25

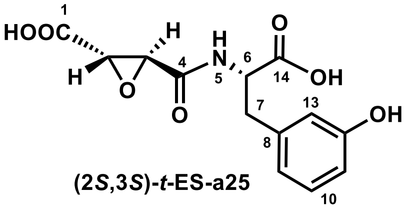

Position	(2*S*,3*S*)-*t*-ES-a25 in DMSO-d_6 (500MHz) δ_H (*J* in Hz)	δ_C, type
1		168.6, C
2	3.61, d (1.8)	52.5, CH
3	3.32, d (1.8)	51.3, CH
4		165.2, C
5	8.65, d (8.2)	
6	4.44, m	53.4, CH
7	2.83, dd (9.4, 13.9); 3.00, dd (4.8, 13.9)	36.5, CH_2
8		138.6, C
9	7.00, m	116.0, CH
10	6.66, m	129.2, CH
11		113.6, CH
12	6.66, m	157.2, C
13	7.00, m	119.7, CH
14		172.3, C

NMR spectrum (500 MHz) for ^{1}H, NMR spectrum (125 MHz) for ^{13}C, DMSO-d_6, "m" means overlapped or multiple with other signals. Chemical shifts are reported in ppm.

HRMS (ESI, M+H$^+$) calculated for $C_{13}H_{14}NO_7^+$ 296.0765; found 296.0767.

$[\alpha]_D^{24.1}$ + 144° (*c* 0.1, MeOH).

Supplementary Table 48. Spectroscopic data of (2*S*,3*S*)-*t*-ES-a26

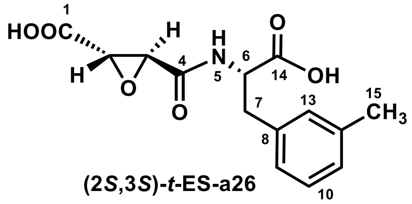

(2*S*,3*S*)-*t*-ES-a26

Position	(2*S*,3*S*)-*t*-ES-a26 in DMSO-d_6 (500MHz) δ_H (*J* in Hz)	δ_C, type
1		168.6, C
2	3.60, d (1.8)	52.5, CH
3	3.31, d (1.8)	51.3, CH
4		165.2, C
5	8.64, d (8.2)	
6	4.46, m	53.4, CH
7	2.87, dd (9.5, 13.7); 3.06, dd (4.8, 13.8)	36.4, CH_2
8		137.2, C
9	7.02, m	126.2, CH
10	7.17, t (7.7)	128.2, CH
11	7.02, m	127.2, CH
12		137.2, C
13	7.02, m	129.8, CH
14		172.3, C
15	2.27, s	21.0, CH_3

NMR spectrum (500 MHz) for ^1H, NMR spectrum (125 MHz) for ^{13}C, DMSO-d_6, "m" means overlapped or multiple with other signals. Chemical shifts are reported in ppm.

HRMS (ESI, M+H$^+$) calculated for $C_{14}H_{16}NO_6^+$ 294.0972; found 294.0977.

$[\alpha]_D^{24.1}$ + 90° (*c* 0.1, MeOH).

Supplementary Table 49. Spectroscopic data of (2*S*,3*S*)-*t*-ES-a27

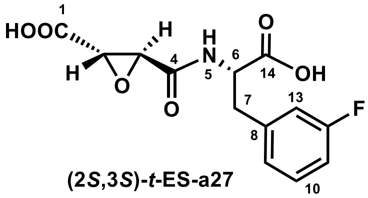

(2*S*,3*S*)-*t*-ES-a27

	(2*S*,3*S*)-*t*-ES-a27 in DMSO-d_6 (500MHz)	
Position	δ_H (*J* in Hz)	δ_C, type
1		168.5, C
2	3.59, d (1.8)	52.4, CH
3	3.30, d (1.8)	51.3, CH
4		165.2, C
5	8.69, d (8.3)	
6	4.51, m	53.0, CH
7	2.93, dd (9.7, 13.8); 3.13, dd (4.9, 13.8)	36.1, CH_2
8		140.2, C
9	7.03, m	125.3, CH
10	7.32, m	130.1, CH
11	7.04, m	113.4, CH
12		161.2, C
13	7.03, m	115.9, CH
14		172.1, C

NMR spectrum (500 MHz) for ^{1}H, NMR spectrum (125 MHz) for ^{13}C, DMSO-d_6, "m" means overlapped or multiple with other signals. Chemical shifts are reported in ppm.

HRMS (ESI, M+H$^+$) calculated for $C_{13}H_{13}NO_6F^+$ 298.0721; found 298.0723.

$[\alpha]_D^{24.1}$ + 92° (*c* 0.1, MeOH).

Supplementary Table 50. Spectroscopic data of (2*S*,3*S*)-*t*-ES-a28

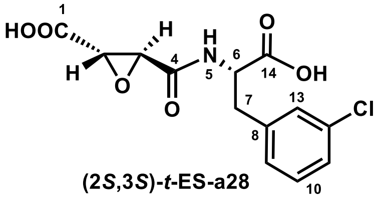

(2*S*,3*S*)-*t*-ES-a28

	(2*S*,3*S*)-*t*-ES-a28 in DMSO-d_6 (500MHz)	
Position	δ_H (*J* in Hz)	δ_C, type
1		168.5, C
2	3.59, d (1.8)	52.5, CH
3	3.30, d (1.8)	51.4, CH
4		165.3, C
5	8.68, d (8.2)	
6	4.46, m	53.0, CH
7	2.92, m; 3.11, m	36.0, CH_2
8		139.9, C
9	7.30, m	129.1, CH
10	7.32, m	130.1, CH
11	7.29, m	126.6, CH
12		132.8, C
13	7.19, m	128.0, CH
14		172.1, C

NMR spectrum (500 MHz) for ^1H, NMR spectrum (125 MHz) for ^{13}C, DMSO-d_6, "m" means overlapped or multiple with other signals. Chemical shifts are reported in ppm.

HRMS (ESI, M+H$^+$) calculated for $C_{13}H_{13}NO_6Cl^+$ 314.0426; found 314.0434.

$[\alpha]_D^{24.1}$ + 70° (*c* 0.1, MeOH).

Supplementary Table 51. Spectroscopic data of (2*S*,3*S*)-*t*-ES-a29

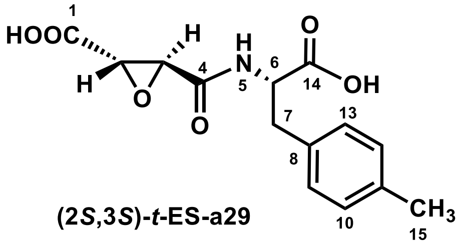

(2*S*,3*S*)-*t*-ES-a29

	(2*S*,3*S*)-*t*-ES-a29 in DMSO-d_6	
Position	δ_H (*J* in Hz)	δ_C, type
1		168.6, C
2	3.60, d (1.9)	52.5, CH
3	3.30, d (1.9)	51.3, CH
4		165.2, C
5	8.64, d (8.2)	
6	4.46, m	53.5, CH
7	2.87, m; 3.05, m	36.1, CH_2
8		134, C
9	7.10, m	129, CH
10	7.10, m	129, CH
11		134.4, C
12	7.10, m	128.9, CH
13	7.10, m	128.9, CH
14		172.4, C
15	2.26, s	20.7, CH_3

NMR spectrum (500 MHz) for ^{1}H, NMR spectrum (125 MHz) for ^{13}C, DMSO-d_6, "m" means overlapped or multiple with other signals. Chemical shifts are reported in ppm.

HRMS (ESI, M+H$^+$) calculated for $C_{14}H_{16}NO_6^+$ 294.0972; found 294.0981.

$[\alpha]_D^{24.1}$ + 109° (*c* 0.1, MeOH)

Supplementary Table 52. Spectroscopic data of (2*S*,3*S*)-*t*-ES-a30

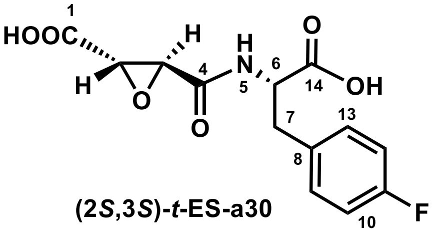

(2*S*,3*S*)-*t*-ES-a30

	(2*S*,3*S*)-*t*-ES-a30 in DMSO-d_6 (500MHz)	
Position	δ_H (J in Hz)	δ_C, type
1		168.5, C
2	3.59, d (1.8)	52.5, CH
3	3.30, d (1.8)	51.3, CH
4		165.2, C
5	8.67, d (8.3)	
6	4.48, m	53.7, CH
7	2.90, dd (9.6, 13.8); 3.09, dd (4.9, 13.9)	35.8, CH_2
8		133.5, C
9	7.26, m	131.0 d (8.06), CH
10	7.11, m	115.0 d (21.06), CH
11		161.1 d (242.13), C
12	7.11, m	115.0 d (21.06), CH
13	7.26, m	131.0 d (8.06), CH
14		172.2, C

NMR spectrum (500 MHz) for ^{1}H, NMR spectrum (125 MHz) for ^{13}C, DMSO-d_6, “m” means overlapped or multiple with other signals. Chemical shifts are reported in ppm.

HRMS (ESI, M+H$^+$) calculated for $C_{13}H_{13}NO_6F^+$ 298.0721; found 298.0723.

$[\alpha]_D^{24.1}$ + 112° (c 0.1, MeOH).

Supplementary Table 53. Spectroscopic data of (2*S*,3*S*)-*t*-ES-a31

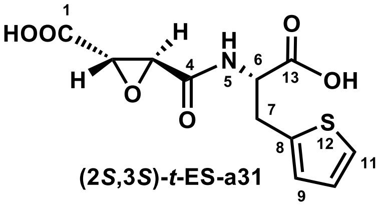

(2*S*,3*S*)-*t*-ES-a31

Position	(2*S*,3*S*)-*t*-ES-a31 in DMSO-d_6 (500MHz) δ_H (*J* in Hz)	δ_C, type
1		168.6, C
2	3.66, d (1.9)	52.5, CH
3	3.38, d (1.9)	51.7, CH
4		165.6, C
5	8.70, d (8.1)	
6	4.48, dd (4.6, 8.5)	53.4, CH
7	3.19, m; 3.32, m	30.8, CH_2
8		139.0, C
9	6.90, d (3.4)	126.5, CH
10	6.95, dd (3.4, 5.2)	126.8, CH
11	7.36, d (5.1)	124.9, CH
13		171.8, C

NMR spectrum (500 MHz) for ^{1}H, NMR spectrum (125 MHz) for ^{13}C, DMSO-d_6, "m" means overlapped or multiple with other signals. Chemical shifts are reported in ppm.

HRMS (ESI, M+H$^+$) calculated for $C_{11}H_{12}NO_6S^+$ 286.0380; found 286.0382.

$[\alpha]_D^{24.1}$ + 50° (*c* 0.1, MeOH).

Supplementary Table 54. Spectroscopic data of (2*S*,3*S*)-*t*-ES-a32

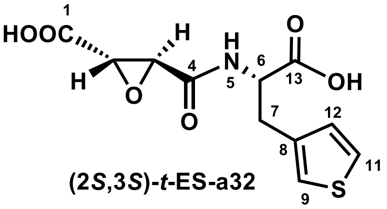

Position	(2*S*,3*S*)-*t*-ES-a32 in DMSO-d_6 (500MHz) δ_H (*J* in Hz)	δ_C, type
1		168.6, C
2	3.62, d (1.8)	52.5, CH
3	3.32, d (1.8)	51.4, CH
4		165.3, C
5	8.65, d (8.1)	
6	4.48, dd (4.7, 8.1, 9.1)	52.9, CH
7	2.98, dd (4.7, 14.5); 3.10, dd (9.3, 14.4)	31.1, CH_2
8		137.4, C
9	7.23, dd (1.2, 3.0)	122.6, CH
11	7.45, dd (3.0, 4.9)	125.9, CH
12	6.99, dd (1.3, 4.9)	128.6, CH
13		172.3, C

NMR spectrum (500 MHz) for ^{1}H, NMR spectrum (125 MHz) for ^{13}C, DMSO-d_6, "m" means overlapped or multiple with other signals. Chemical shifts are reported in ppm.

HRMS (ESI, M+H$^+$) calculated for $C_{11}H_{12}NO_6S^+$ 286.0380; found 286.0378.

$[\alpha]_D^{24.1}$ + 54° (*c* 0.1, MeOH).

Supplementary Table 55. Spectroscopic data of **(2*S*,3*S*)-*t*-ES-Phe-b15**

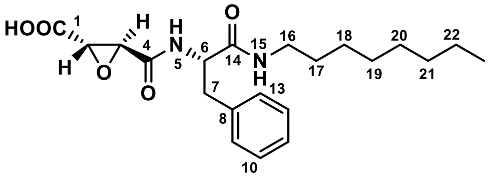

***t*-ES-Phe-b15**

Position	***t*-ES-Phe-b15** in DMSO-d_6 (500MHz) δ_H (J in Hz)	δ_C, type
1		168.7, C
2	3.59, d (1.8)	52.6, CH
3	3.30, d (1.8)	51.2, CH
4		164.9, C
5	8.61, d (8.6)	
6	4.50, td (5.4, 9.0)	54.1, CH
7	2.79, dd (9.3, 13.6); 3.05, m	37.9, CH_2
8		137.5, C
9	7.22, m	129.1, CH
10	7.27, m	128.1, CH
11	7.19, m	126.4, CH
12	7.27, m	128.1, CH
13	7.22, m	129.1, CH
14		170.0, C
15	8.06, t (5.6)	
16	2.97, m	38.5, CH_2
17	1.33, m	28.9, CH_2
18	1.18, m	26.3, CH_2
19	1.23, m	28.7, CH_2
20	1.23, m	28.6, CH_2
21	1.23, m	31.2, CH_2
22	1.24, m	22.1, CH_2
23	0.86, t (6.9)	14.0, CH_3

NMR spectrum (500 MHz) for ^{1}H, NMR spectrum (125 MHz) for ^{13}C, DMSO-d_6, "m" means overlapped or multiple with other signals. Chemical shifts are reported in ppm.

HRMS (ESI, M+H$^+$) calculated for $C_{21}H_{31}N_2O_5^+$ 391.2227; found 391.2221.

$[\alpha]_D^{24.1}$ + 35° (c 0.1, MeOH).

Supplementary Table 56. Spectroscopic data of **(2*S*,3*S*)-*t*-ES-Phe-b18**

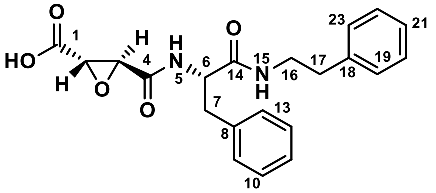

***t*-ES-Phe-b18**

Position	**(2*S*,3*S*)-*t*-ES-Phe-b18** in DMSO-d_6 (500MHz) δ_H (*J* in Hz)	δ_C, type
1		168.7, C
2	3.58, d (1.8)	52.6, CH
3	3.29, d (1.8)	51.3, CH
4		164.9, C
5	8.60, d (8.6)	
6	4.50, td (4.9, 9.2)	54.1, CH
7	2.76, dd (9.7, 13.6); 2.94, m	37.8, CH_2
8		137.6, C
9	7.22, m	129.2, CH
10	7.27, m	128.1, CH
11	7.19, m	126.4, CH
12	7.27, m	128.1, CH
13	7.22, m	129.2, CH
14		170.2, C
15	8.21, t (5.6)	
16	3.25, m	40.2, CH_2
17	2.67, m	35.0, CH_2
18		139.3, C
19	7.22, m	128.7, CH
20	7.27, m	128.3, CH
21	7.19, m	126.1, CH
22	7.27, m	128.3, CH
23	7.22, m	128.7, CH

NMR spectrum (500 MHz) for ^1H, NMR spectrum (125 MHz) for ^{13}C, DMSO-d_6, "m" means overlapped or multiple with other signals. Chemical shifts are reported in ppm.

HRMS (ESI, M+H$^+$) calculated for $C_{21}H_{23}N_2O_5^+$ 383.1601; found 383.1596.

$[\alpha]_D^{24.1}$ + 30° (*c* 0.1, MeOH).

Supplementary Table 57. Spectroscopic data of **(2*S*,3*S*)-*t*-ES-Phe-b19**

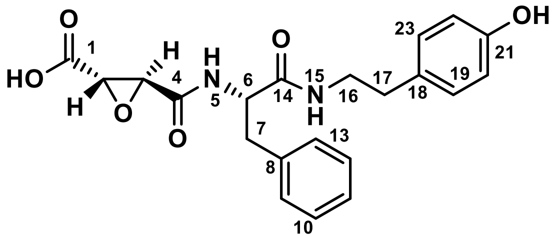

***t*-ES-Phe-b19**

Position	**(2*S*,3*S*)-*t*-ES-Phe-b19** in DMSO-d_6 (500MHz) δ_H (*J* in Hz)	δ_C, type
1		168.7, C
2	3.58, d (1.8)	52.6, CH
3	3.29, d (1.8)	51.3, CH
4		164.9, C
5	8.59, d (8.6)	
6	4.49, td (4.9, 9.1)	54.1, CH
7	2.75, m; 2.95, m	37.8, CH_2
8		137.6, C
9	7.20, m	129.2, CH
10	7.27, m	128.1, CH
11	7.20, m	126.4, CH
12	7.27, m	128.1, CH
13	7.20, m	129.2, CH
14		170.2, C
15	8.17, t (5.7)	
16	3.17, m; 3.23, m	40.6, CH_2
17	2.55, m	34.2, CH_2
18		129.3, C
19	6.96, m	129.5, CH
20	6.66, m	115.1, CH
21		155.7, C
22	6.66, m	115.1, CH
23	6.97, m	129.5, CH

NMR spectrum (500 MHz) for ^1H, NMR spectrum (125 MHz) for ^{13}C, DMSO-d_6, "m" means overlapped or multiple with other signals. Chemical shifts are reported in ppm.

HRMS (ESI, M+H$^+$) calculated for $C_{21}H_{23}N_2O_6^+$ 399.1551; found 399.1550.

$[\alpha]_D^{24.1}$ + 32° (*c* 0.1, MeOH).

Supplementary Table 58. Spectroscopic data of **(2*S*,3*S*)-*t*-ES-Phe-b20**

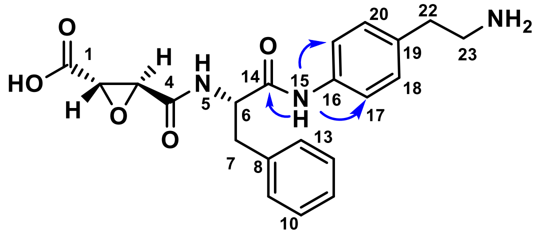

***t*-ES-Phe-b20**

Position	**(2*S*,3*S*)-*t*-ES-Phe-b20** in DMSO-d_6 (500MHz) δ_H (J in Hz)	δ_C, type
1		168.6, C
2	3.63, d (1.8)	52.5, CH
3	3.33, d (1.8)	51.3, CH
4		165.2, C
5	8.82, d (8.2)	
6	4.72, m	54.8, CH
7	2.91, m; 3.08, m	37.7, CH_2
8		137.2, C
9	7.20, m	129.2, CH
10	7.29, m	128.2, CH
11	7.20, m	126.6, CH
12	7.29, m	128.2, CH
13	7.20, m	129.2, CH
14		169.3, C
15	10.21, s	
16		137.7, C
17	7.53, m	119.7, CH
18	7.83, m	129.0, CH
19		132.3, C
20	7.83, m	129.0, CH
21	7.53, m	119.7, CH
22	2.81, m	32.9, CH_2
23	3.01, m	40.4, CH_2

NMR spectrum (500 MHz) for ^{1}H, NMR spectrum (125 MHz) for ^{13}C, DMSO-d_6, "m" means overlapped or multiple with other signals. Chemical shifts are reported in ppm.

HRMS (ESI, M+H$^+$) calculated for $C_{21}H_{24}N_3O_5^+$ 398.1710; found 398.1709.

$[\alpha]_D^{24.1}$ + 38° (c 0.1, MeOH).

Supplementary Table 59. Spectroscopic data of **(2*S*,3*S*)-*t*-ES-Phe-b24**

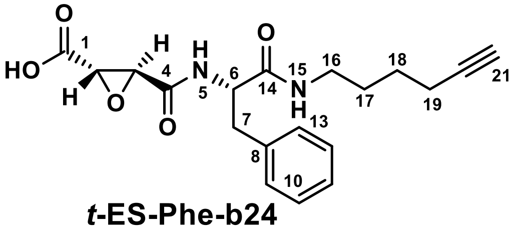

***t*-ES-Phe-b24**

Position	**(2*S*,3*S*)-*t*-ES-Phe-b24** in DMSO-d_6 (500MHz) δ_H (*J* in Hz)	δ_C, type
1		168.7, C
2	3.59, d (1.8)	52.6, CH
3	3.33, d (1.8)	51.3, CH
4		164.9, C
5	8.61, d (8.5)	
6	4.49, m	54.1, CH
7	2.80, m; 3.03, m	37.9, CH_2
8		137.5, C
9	7.20, m	129.2, CH
10	7.29, m	128.1, CH
11	7.20, m	126.4, CH
12	7.29, m	128.1, CH
13	7.20, m	129.2, CH
14		170.1, C
15	8.10, t (5.7)	
16	2.97, m	38.0, CH_2
17	1.43, m	28.1, CH_2
18	1.39, m	25.3, CH_2
19	2.13, m	17.4, CH_2
20		84.4, C
21	2.74, m	71.3, CH

NMR spectrum (500 MHz) for ^{1}H, NMR spectrum (125 MHz) for ^{13}C, DMSO-d_6, "m" means overlapped or multiple with other signals. Chemical shifts are reported in ppm.

HRMS (ESI, M+H$^+$) calculated for $C_{19}H_{23}N_2O_5^+$ 359.1601; found 359.1610.

$[\alpha]_D^{24.1}$ + 22° (*c* 0.1, MeOH).

Supplementary Table 60. Spectroscopic data of **(2*S*,3*S*)-*t*-ES-Phe-b27**

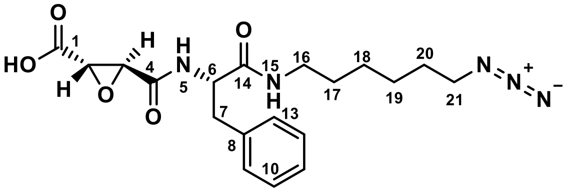

***t*-ES-Phe-b27**

	**(2*S*,3*S*)-*t*-ES-Phe-b27** in DMSO-d_6 (500MHz)	
Position	δ_H (*J* in Hz)	δ_C, type
1		168.7, C
2	3.59, d (1.8)	52.6, CH
3	3.32, d (1.8)	51.3, CH
4		164.9, C
5	8.61, d (8.5)	
6	4.50, m	54.1, CH
7	2.80, m; 2.96, m	37.9, CH_2
8		137.5, C
9	7.20, m	129.2, CH
10	7.27, m	128.1, CH
11	7.20, m	126.4, CH
12	7.27, m	128.1, CH
13	7.20, m	129.2, CH
14		170.0, C
15	8.07, t (5.7)	
16	2.99, m	38.4, CH_2
17	1.31, m	28.8, CH_2
18	1.20, m	25.8, CH_2
19	1.31, m	25.8, CH_2
20	1.50, m	28.2, CH_2
21	3.31, m	50.6, CH_2

NMR spectrum (500 MHz) for ^{1}H, NMR spectrum (125 MHz) for ^{13}C, DMSO-d_6, “m” means overlapped or multiple with other signals. Chemical shifts are reported in ppm.

HRMS (ESI, M+H$^+$) calculated for $C_{19}H_{26}N_5O_5^+$ 404.1928; found 404.1927.

$[\alpha]_D^{24.1}$ + 28° (*c* 0.1, MeOH).

Supplementary Table 61. Spectroscopic data of **(2*S*,3*S*)-*t*-ES-Phe-b29**

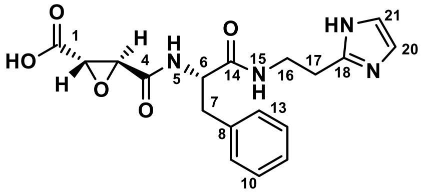

***t*-ES-Phe-b29**

Position	**(2*S*,3*S*)-*t*-ES-Phe-b29** in DMSO-d_6 (500MHz) δ_H (*J* in Hz)	δ_C, type
1		168.7, C
2	3.56, d (1.8)	52.6, CH
3	3.27, d (1.8)	51.3, CH
4		165.1, C
5	8.59, d (8.4)	
6	4.44, m	54.0, CH
7	2.74, m; 2.96, m	37.5, CH_2
8		137.5, C
9	7.20, m	129.1, CH
10	7.27, m	128.1, CH
11	7.20, m	126.5, CH
12	7.27, m	128.1, CH
13	7.20, m	129.1, CH
14		170.8, C
15	8.40, t (5.8)	
16	3.46, m	36.5, CH_2
17	3.02, m	25.9, CH_2
18		145.1, C
20	7.55, m	119.0, CH
21	7.55, m	119.0, CH

NMR spectrum (500 MHz) for ^1H, NMR spectrum (125 MHz) for ^{13}C, DMSO-d_6, "m" means overlapped or multiple with other signals. Chemical shifts are reported in ppm.

HRMS (ESI, M+H$^+$) calculated for $C_{18}H_{21}N_4O_5^+$ 373.1506; found 373.1505.

$[\alpha]_D^{24.1}$ + 18° (*c* 0.1, MeOH).

Supplementary Table 62. Spectroscopic data of **(2*S*,3*S*)-*t*-ES-Phe-b30**

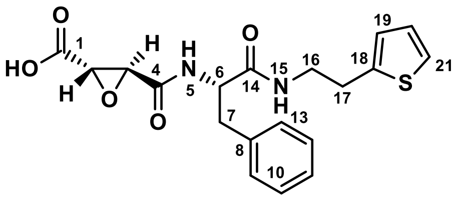

***t*-ES-Phe-b30**

	**(2*S*,3*S*)-*t*-ES-Phe-b30** in DMSO-d_6 (500MHz)	
Position	δ_H (J in Hz)	δ_C, type
1		168.7, C
2	3.58, d (1.8)	52.6, CH
3	3.32, d (1.8)	51.3, CH
4		164.9, C
5	8.63, d (8.5)	
6	4.51, m	54.1, CH
7	2.78, m; 2.99, m	37.7, CH_2
8		137.6, C
9	7.21, m	129.2, CH
10	7.27, m	128.1, CH
11	7.21, m	126.4, CH
12	7.27, m	128.1, CH
13	7.21, m	129.2, CH
14		170.4, C
15	8.29, t (5.7)	
16	3.26, m	40.4, CH_2
17	2.89, m	29.1, CH_2
18		141.3, C
19	6.86, d (3.4)	125.3, CH
20	6.94, m	127.0, CH
21	7.33, d (5.1)	124.0, CH

NMR spectrum (500 MHz) for ^1H, NMR spectrum (125 MHz) for ^{13}C, DMSO-d_6, “m” means overlapped or multiple with other signals. Chemical shifts are reported in ppm.

HRMS (ESI, M+H$^+$) calculated for $C_{19}H_{21}N_2O_5S^+$ 389.1116; found 389.1138.

$[\alpha]_D^{24.1}$ + 36° (c 0.1, MeOH).

Supplementary Table 63. Spectroscopic data of **(2*S*,3*S*)-*t*-ES-Phe-b31**

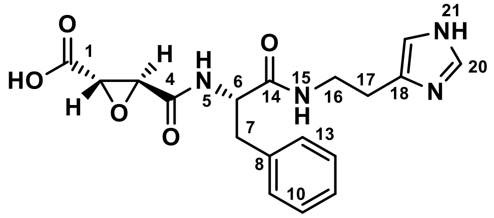

***t*-ES-Phe-b31**

Position	**(2*S*,3*S*)-*t*-ES-Phe-b31** in DMSO-d_6 (500MHz) δ_H (J in Hz)	δ_C, type
1		168.7, C
2	3.57, d (1.8)	52.6, CH
3	3.37, d (1.8)	51.3, CH
4		165.1, C
5	8.63, d (8.4)	
6	4.44, m	54.2, CH
7	2.76, m; 2.95, m	37.6, CH_2
8		137.5, C
9	7.20, m	129.1, CH
10	7.26, m	128.1, CH
11	7.20, m	126.5, CH
12	7.26, m	128.1, CH
13	7.20, m	129.1, CH
14		170.6, C
15	8.28, t (5.8)	
16	3.35, m	37.3, CH_2
17	2.75, m	24.3, CH_2
18		130.7, C
20	8.98, d (1.3)	133.8, CH
22	7.35, s	116.7, CH

NMR spectrum (500 MHz) for ^1H, NMR spectrum (125 MHz) for ^{13}C, DMSO-d_6, "m" means overlapped or multiple with other signals. Chemical shifts are reported in ppm.

HRMS (ESI, M+H$^+$) calculated for $C_{18}H_{21}N_4O_5^+$ 373.1506; found 373.1510.

$[\alpha]_D^{24.1}$ + 24° (c 0.1, MeOH).

Supplementary Table 64. Spectroscopic data of **(2*S*,3*S*)-*t*-ES-Phe-b32**

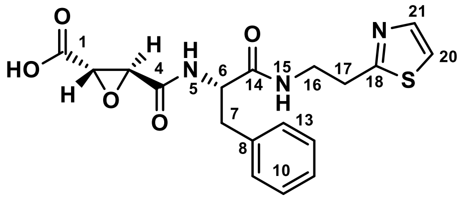

***t*-ES-Phe-b32**

Position	**(2*S*,3*S*)-*t*-ES-Phe-b32** in DMSO-d_6 (500MHz) δ_H (*J* in Hz)	δ_C, type
1		168.7, C
2	3.58, d (1.9)	52.6, CH
3	3.29, d (1.8)	51.3, CH
4		164.9, C
5	8.63, d (8.6)	
6	4.50, m	54.0, CH
7	2.78, m; 2.99, m	37.7, CH_2
8		137.6, C
9	7.21, m	129.2, CH
10	7.27, m	128.1, CH
11	7.21, m	126.4, CH
12	7.27, m	128.1, CH
13	7.21, m	129.2, CH
14		170.5, C
15	8.33, t (5.7)	
16	3.43, m	38.6, CH_2
17	3.09, m	32.3, CH_2
18		167.2, C
20	7.59, d (3.4)	119.7, CH
21	7.73, d (3.3)	142.1, CH

NMR spectrum (500 MHz) for ^{1}H, NMR spectrum (125 MHz) for ^{13}C, DMSO-d_6, "m" means overlapped or multiple with other signals. Chemical shifts are reported in ppm.

HRMS (ESI, M+H$^+$) calculated for $C_{18}H_{20}N_3O_5S^+$ 390.1118; found 390.1119.

$[\alpha]_D^{24.1}$ + 20° (*c* 0.1, MeOH).

Supplementary Table 65. Spectroscopic data of **(2*S*,3*S*)-*t*-ES-Phe-b33**

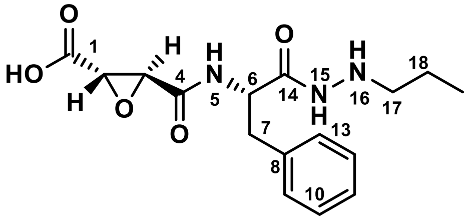

***t*-ES-Phe-b33**

	**(2*S*,3*S*)-*t*-ES-Phe-b33** in DMSO-d_6 (500MHz)	
Position	δ_H (*J* in Hz)	δ_C, type
1		168.6, C
2	3.60, d (1.8)	52.5, CH
3	3.31, d (1.8)	51.3, CH
4		165.3, C
5	8.84, d (8.0)	
6	4.54, m	52.8, CH
7	2.90, m; 3.00, m	37.2, CH_2
8		136.8, C
9	7.24, m	129.2, CH
10	7.29, m	128.2, CH
11	7.24, m	126.7, CH
12	7.29, m	128.2, CH
13	7.24, m	129.2, CH
14		169.2, C
17	2.76, m	51.8, CH_2
18	1.37, m	18.6, CH_2
19	0.84, t (7.4)	11.1, CH_3

NMR spectrum (500 MHz) for ^{1}H, NMR spectrum (125 MHz) for ^{13}C, DMSO-d_6, “m” means overlapped or multiple with other signals. Chemical shifts are reported in ppm.

HRMS (ESI, M+H$^+$) calculated for $C_{16}H_{22}N_3O_5^+$ 336.1554; found 336.1552.

$[\alpha]_D^{24.1}$ + 12° (*c* 0.1, MeOH).

Supplementary Table 66. Spectroscopic data of **(2*S*,3*S*)-*t*-ES-Phe-b37**

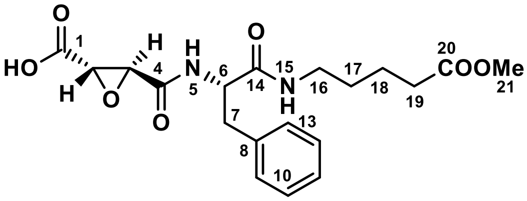

***t*-ES-Phe-b37**

	**(2*S*,3*S*)-*t*-ES-Phe-b37** in DMSO-d_6 (500MHz)	
Position	δ_H (*J* in Hz)	δ_C, type
1		168.7, C
2	3.58, d (1.5)	52.6, CH
3	3.29, d (1.9)	51.2, CH
4		164.9, C
5	8.61, d (8.5)	
6	4.49, m	54.1, CH
7	2.79, m; 2.98, m	37.8, CH_2
8		137.5, C
9	7.19, m	129.1, CH
10	7.26, m	128.1, CH
11	7.19, m	126.4, CH
12	7.26, m	128.1, CH
13	7.19, m	129.1, CH
14		170.1, C
15	8.09, t (5.7)	
16	3.02, m	38.1, CH_2
17	1.34, m	28.3, CH_2
18	1.44, m	21.8, CH_2
19	2.28, m	32.9, CH_2
20		173.2, C
21	3.58, s	51.2, CH_3

NMR spectrum (500 MHz) for ^{1}H, NMR spectrum (125 MHz) for ^{13}C, DMSO-d_6, “m” means overlapped or multiple with other signals. Chemical shifts are reported in ppm.

HRMS (ESI, M+H$^+$) calculated for $C_{19}H_{25}N_2O_7^+$ 393.1656; found 393.1663.

$[\alpha]_D^{24.1}$ + 18° (*c* 0.1, MeOH).

Supplementary Table 67. Spectroscopic data of **(2*S*,3*S*)-*t*-ES-Phe-b38**

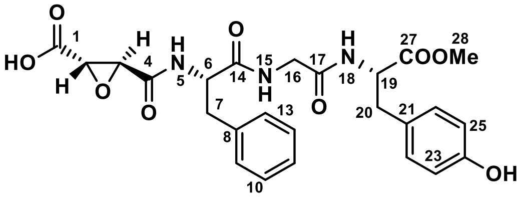

***t*-ES-Phe-b38**

Position	**(2*S*,3*S*)-*t*-ES-Phe-b38** in DMSO-d_6 (500MHz) δ_H (J in Hz)	δ_C, type
1		168.6, C
2	3.56, d (1.8)	52.5, CH
3	3.27, d (1.8)	51.3, CH
4		165.2, C
5	8.62, d (8.4)	
6	4.57, m	54.0, CH
7	2.79, m; 3.06, m	37.5, CH_2
8		137.6, C
9	7.23, m	129.2, CH
10	7.25, m	128.1, CH
11	7.20, m	126.4, CH
12	7.25, m	128.1, CH
13	7.23, m	129.2, CH
14		168.6, C
15	8.40, t (5.8)	
16	3.72, m	41.6, CH_2
17		170.7, C
18	8.25, d (7.7)	
19	4.39, m	53.9, CH
20	2.80, m; 2.88, m	36.1, CH_2
21		126.9, C
22	6.99, m	130.2, CH
23	6.66, m	115.1, CH
24		156.0, C
25	6.66, m	115.1, CH
26	6.99, m	130.2, CH
27		172.0, C
28	3.59, s	51.8, CH_3

NMR spectrum (500 MHz) for ^{1}H, NMR spectrum (125 MHz) for ^{13}C, DMSO-d_6, "m" means overlapped or multiple with other signals. Chemical shifts are reported in ppm.

HRMS (ESI, M+H$^+$) calculated for $C_{25}H_{28}N_3O_9^+$ 514.1820; found 514.1825.

$[\alpha]_D^{24.1}$ + 14° (c 0.1, MeOH).

Supplementary Table 68. Spectroscopic data of **(2*S*,3*S*)-*t*-ES-Phe-b41**

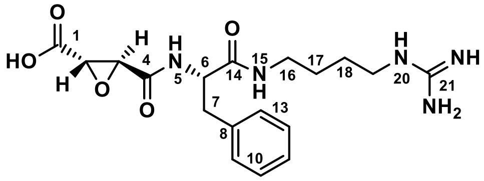

***t*-ES-Phe-b41**

	**(2*S*,3*S*)-*t*-ES-Phe-b41** in DMSO-d_6 (500MHz)	
Position	δ_H (*J* in Hz)	δ_C, type
1		168.7, C
2	3.59, d (1.8)	52.6, CH
3	3.29, d (1.8)	51.3, CH
4		165.0, C
5	8.62, d (8.4)	
6	4.49, td (5.4, 9.0)	54.1, CH
7	2.81, dd (9.3, 13.6); 3.05, m	37.8, CH_2
8		137.5, C
9	7.21, m	129.2, CH
10	7.27, m	128.1, CH
11	7.21, m	126.4, CH
12	7.27, m	128.1, CH
13	7.21, m	129.2, CH
14		170.3, C
15	8.14, t (5.7)	
16	3.08, m	38.1, CH_2
17	1.37, m	26.2, CH_2
18	1.37, m	25.9, CH_2
19	3.08, m	40.4, CH_2
20	7.55, m	
21		156.7, C

NMR spectrum (500 MHz) for ^{1}H, NMR spectrum (125 MHz) for ^{13}C, DMSO-d_6, "m" means overlapped or multiple with other signals. Chemical shifts are reported in ppm.

HRMS (ESI, M+H$^+$) calculated for $C_{18}H_{26}N_5O_5^+$ 392.1920; found 392.1931.

$[\alpha]_D^{24.1}$ + 24° (*c* 0.1, MeOH).

Supplementary Table 69. Spectroscopic data of **(2*S*,3*S*)-*t*-ES-a9-b7**

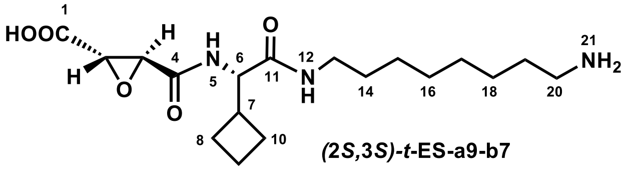

Position	**(2*S*,3*S*)-*t*-ES-a9-b7** in DMSO-d_6 (500MHz) δ_H (J in Hz)	δ_C, type
1		168.8, C
2	3.73, d (1.8)	52.5, CH
3	3.44, d (1.8)	51.3, CH
4		165.1, C
5	8.53, d (8.5)	
6	4.27, t (8.5)	56.8, CH
7	2.53, m	37.4, CH
8	1.73, m; 1.88, m	24.7, CH_2
9	1.75, m	17.5, CH_2
10	1.82, m	24.3, CH_2
11		169.5, C
12	8.04, t (5.7)	
13	2.96, m; 3.08, m	38.3, CH_2
14	1.35, m	29.0, CH_2
15	1.23, m	26.1, CH_2
16	1.23, m	28.5, CH_2
17	1.23, m	25.7, CH_2
18	1.23, m	28.5, CH_2
19	1.49, m	27.0, CH_2
20	2.75, m	38.8, CH_2

NMR spectrum (500 MHz) for ^{1}H, NMR spectrum (125 MHz) for ^{13}C, DMSO-d_6, "m" means overlapped or multiple with other signals. Chemical shifts are reported in ppm.

HRMS (ESI, M+H$^+$) calculated for $C_{18}H_{32}N_3O_5^+$ 370.2336; found 370.2332.

$[\alpha]_D^{24.1}$ + 36° (*c* 0.1, MeOH).

Supplementary Table 70. Spectroscopic data of **(2S,3S)-*t*-ES-a9-b13**

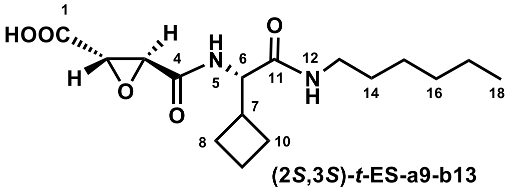

(2*S*,3*S*)-*t*-ES-a9-b13

Position	**(2*S*,3*S*)-*t*-ES-a9-b13** in DMSO-d_6 (500MHz) δ_H (J in Hz)	δ_C, type
1		168.8, C
2	3.74, d (1.8)	52.5, CH
3	3.45, d (1.8)	51.2, CH
4		165.0, C
5	8.53, d (8.4)	
6	4.28, t (8.5)	56.7, CH
7	2.54, m	37.5, CH
8	1.75, m; 1.83, m	24.7, CH_2
9	1.73, m	17.4, CH_2
10	1.82, m	24.3, CH_2
11		169.5, C
12	8.04, t (5.7)	
13	2.95, m; 3.09, m	38.3, CH_2
14	1.35, m	29.0, CH_2
15	1.23, m	25.9, CH_2
16	1.23, m	30.9, CH_2
17	1.23, m	22.1, CH_2
18	0.85, t (6.8)	13.9, CH_3

NMR spectrum (500 MHz) for ^{1}H, NMR spectrum (125 MHz) for ^{13}C, DMSO-d_6, "m" means overlapped or multiple with other signals. Chemical shifts are reported in ppm.

HRMS (ESI, M+H$^+$) calculated for $C_{16}H_{27}N_2O_5^+$ 327.1914; found 327.1912.

$[\alpha]_D^{24.1}$ + 36° (c 0.1, MeOH).

Supplementary Table 71. Spectroscopic data of **(2*S*,3*S*)-*t*-ES-a10-b9**

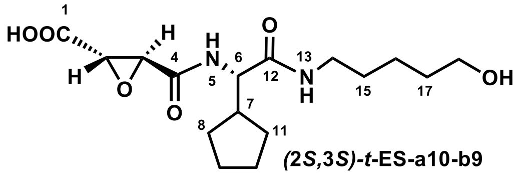

Position	**(2*S*,3*S*)-*t*-ES-a10-b9** in DMSO-d_6 (500MHz) δ_H (*J* in Hz)	δ_C, type
1		168.8, C
2	3.70, d (1.8)	52.1, CH
3	3.42, d (1.8)	51.2, CH
4		164.9, C
6	4.16, t (8.7)	56.1, CH
7	2.12, m	42.2, CH
8	1.26, m	28.6, CH_2
9	1.54, m	24.8, CH_2
10	1.45, m	24.5, CH_2
11	1.51, m; 1.61, m	28.6, CH_2
12		170.3, C
14	2.95, dt (6.9, 13.5); 3.09, dt (6.9, 13.5)	38.3, CH_2
15	1.36, m	28.8, CH_2
16	1.23, m	22.9, CH_2
17	1.39, m	32.1, CH_2
18	1.39, dd (8.7, 15.2)	60.6, CH_2

NMR spectrum (500 MHz) for ^{1}H, NMR spectrum (125 MHz) for ^{13}C, DMSO-d_6, “m” means overlapped or multiple with other signals. Chemical shifts are reported in ppm.

HRMS (ESI, M+H$^+$) calculated for $C_{16}H_{27}N_2O_6^+$ 343.1864; found 343.1807.

$[\alpha]_D^{24.1}$ + 56° (*c* 0.1, MeOH).

Supplementary Table 72. Spectroscopic data of **(2*S*,3*S*)-*t*-ES-a10-b14**

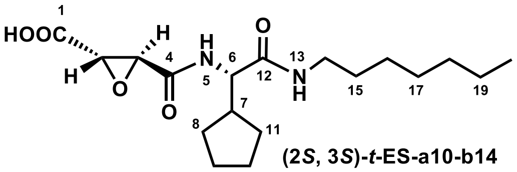

(2*S*, 3*S*)-*t*-ES-a10-b14

Position	**(2*S*,3*S*)-*t*-ES-a10-b14** in acetone-d_6 (500MHz) δ_H (*J* in Hz)	δ_C, type
1		168.8, C
2	3.69, d (1.8)	54.2, CH
3	3.59, d (1.8)	52.6, CH
4		166.0, C
5	7.47, d (8.7)	
6	4.30, t (8.5)	57.2, CH
7	2.25, m	43.9, CH
8	1.37, m	29.9, CH_2
9	1.50, m	25.6, CH_2
10	1.60, m	25.8 CH_2
11	1.63, m	30.1, CH_2
12		171.4, C
13	7.37, t (5.8)	
14	3.13, dt (6.4, 13.0); 3.25, dt (6.6, 13.2)	39.8, CH_2
15	1.49, m	30.2, CH_2
16	1.27, m	32.5, CH_2
17	1.70, m	29.3, CH_2
18	1.30, m	27.5, CH_2
19	1.28, m	23.2, CH_2
20	0.89, m	14.3, CH_3

NMR spectrum (500 MHz) for ^1H, NMR spectrum (125 MHz) for ^{13}C, acetone-d_6, "m" means overlapped or multiple with other signals. Chemical shifts are reported in ppm.

HRMS (ESI, M+H$^+$) calculated for $C_{18}H_{31}N_2O_5^+$ 355.2227; found 355.2226.

$[\alpha]_D^{24.1}$ + 56° (*c* 0.1, MeOH).

Supplementary Table 73. Spectroscopic data of **(2*S*,3*S*)-*t*-ES-a10-b26**

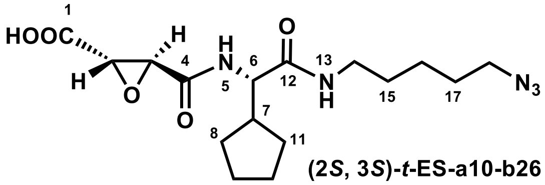

	**(2*S*,3*S*)-*t*-ES-a10-b26** in acetone-d_6 (500MHz)	
Position	δ_H (*J* in Hz)	δ_C, type
1		168.7, C
2	3.69, d (1.8)	54.2, CH
3	3.59, d (1.8)	52.8, CH
4		166.1, C
5	7.45, m	
6	4.29, t (8.5)	57.2, CH
7	2.26, m	43.8, CH
8	1.32, m; 1.52, m	30.2, CH_2
9	1.49, m	25.6, CH_2
10	1.56, m	25.6, CH_2
11	1.69, m	29.3, CH_2
12		171.4, C
13	7.45, m	
14	3.16, m; 3.26, m	39.5, CH_2
15	1.51, m	25.9, CH_2
16	1.40, m	24.7, CH_2
17	1.51, m; 1.60, m	29.1, CH_2
18	3.33, m	51.9, CH_2

NMR spectrum (500 MHz) for ^{1}H, NMR spectrum (125 MHz) for ^{13}C, acetone-d_6, "m" means overlapped or multiple with other signals. Chemical shifts are reported in ppm.

HRMS (ESI, M+H$^+$) calculated for $C_{16}H_{26}N_5O_5^+$ 368.1928; found 368.1929.

$[\alpha]_D^{24.1}$ + 42° (*c* 0.1, MeOH).

Supplementary Table 74. Spectroscopic data of **(2*S*,3*S*)-*t*-ES-Leu-b43**

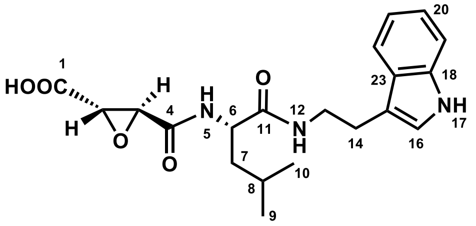

(2*S*,3*S*)-*t*-ES-Leu-b43

	**(2*S*,3*S*)-*t*-ES-Leu-b43** in DMSO-d_6 (500MHz)	
Position	δ_H (J in Hz)	δ_C, type
1		168.8, C
2	3.67, d (1.8)	52.7, CH
3	3.48, d (1.8)	51.2, CH
4		164.9, C
5	8.58, d (8.4)	
6	4.32, m	51.2, CH
7	1.44, m	41.1, CH_2
8	1.52, m	24.2, CH
9	0.83, d (6.5)	21.6, CH_3
10	0.87, d (6.5)	22.9, CH_3
11		171.1, C
12	8.20, t (5.7)	
13	3.31, m	40.1, CH_2
14	2.81, m	25.0, CH_2
15		111.6, C
16	7.13, d (2.3)	122.7, CH
17	10.81, m	
18		136.2, C
19	7.32, d (8.1)	111.3, CH
20	7.05, t (7.5)	120.9, CH
21	6.97, t (7.4)	118.2, CH
22	7.53, d (7.8)	118.2, CH
23		127.2, C

NMR spectrum (500 MHz) for ^{1}H, NMR spectrum (125 MHz) for ^{13}C, DMSO-d_6, "m" means overlapped or multiple with other signals. Chemical shifts are reported in ppm.

HRMS (ESI, M+H$^+$) calculated for $C_{20}H_{26}N_3O_5^+$ 388.1867; found 388.1864.

$[\alpha]_D^{24.1}$ + 48° (c 0.1, MeOH).

Supplementary Table 75. Spectroscopic data of **E-64c-Hydrazide**

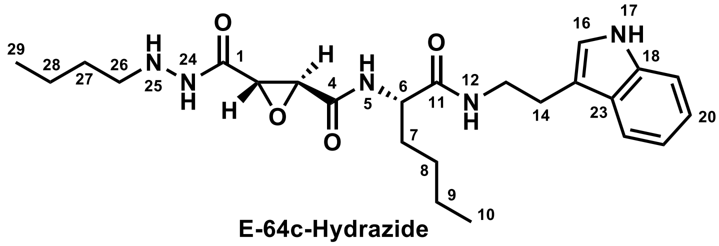

Position	**E-64c-Hydrazide**[10] in DMSO-d_6 (500MHz) δ_H (*J* in Hz)	δ_C, type
1		164.7, C
2	3.67, d (1.8)	52.6, CH
3	3.48, d (1.8)	51.7, CH
4		165.1, C
5	8.58, d (8.4)	
6	4.32, m	52.7, CH
7	1.52, m; 1.61, m	32.0, CH_2
8	1.22, m	27.4, CH_2
9	1.24, m	21.7, CH_2
10	0.86, m	13.7, CH_3
11		170.8, C
12	8.19, t (5.7)	
13	3.30, m	40.2, CH_2
14	2.83, m	25.1, CH_2
15		111.6, C
16	7.13, d (2.3)	122.7, CH
17	10.81, m	
18		136.2, C
19	7.32, d (8.1)	111.6, CH
20	7.05, t (7.5)	121.3, CH
21	6.97, t (7.4)	118.2, CH
22	7.53, d (7.8)	118.2, CH
23		127.2, C
26	2.83, m	50.2, CH_2
27	1.44, m	28.1, CH_2
28	1.31, m	19.5, CH_2
29	0.86, m	13.9, CH_3

NMR spectrum (500 MHz) for ^1H, NMR spectrum (125 MHz) for ^{13}C, DMSO-d_6, "m" means overlapped or multiple with other signals. Chemical shifts are reported in ppm.

HRMS (ESI, M+H$^+$) calculated for $C_{24}H_{36}N_5O_4^+$ 458.2762; found 458.2755.

$[\alpha]_D^{24.1}$ + 18° (*c* 0.1, MeOH).

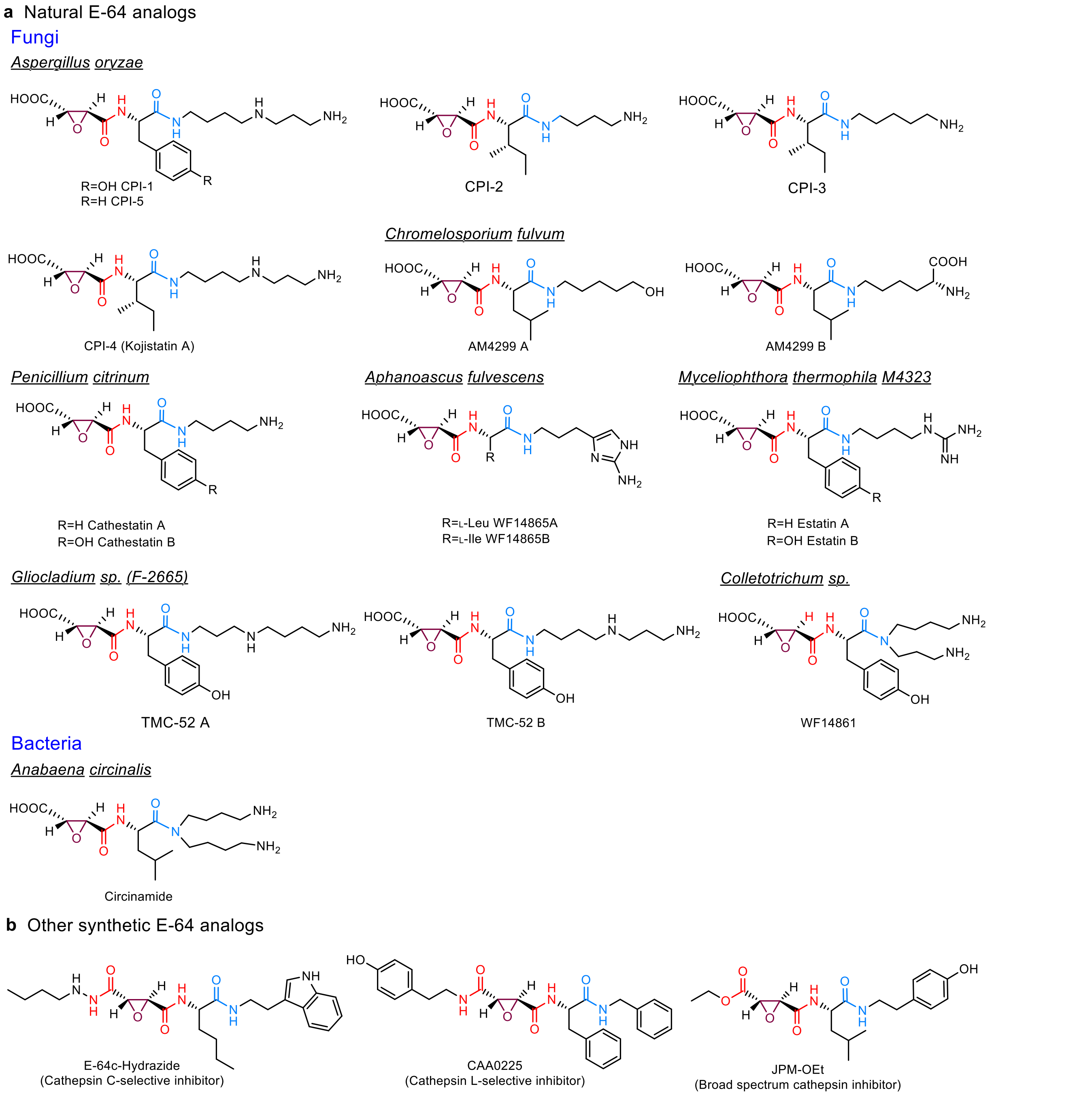

Supplementary Fig. 1 | Natural and synthetic E-64 analogs. **a**, Natural E-64 analogs, including CP1-1 to CPI-5[9], AM4299A and B[11], cathestatin A and B[12], estatin A and B[13], WF14865A and B[14], TMC-52 A and B[15], were isolated, from different fungal species, such as *Aspergillus*, *Penicillium*, *Aphanoascus*, *Myceliophthora*, *Gliocladium*, *Colletotrichum spp*. E-64 analog circinamide[16] was isolated from cyanobacteria *Anabaena circinalis*. Most of E-64 analogs contain putrescine and cadaverine or its derivatives at C-terminus. **b**, Other synthetic cysteine protease inhibitors derived from E-64[17].

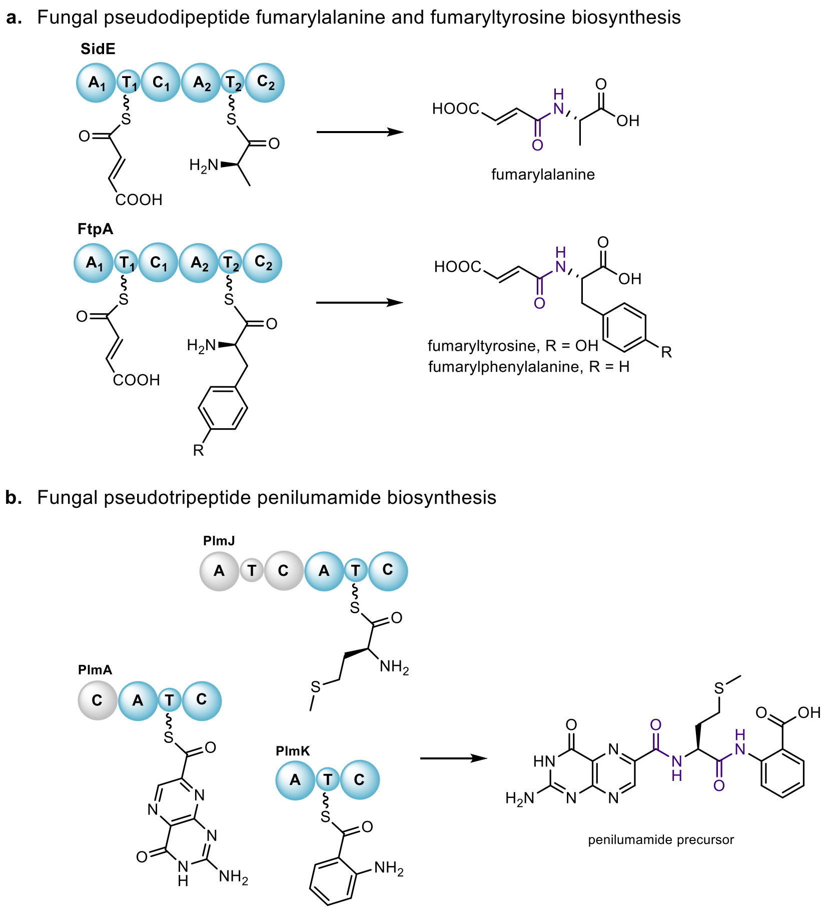

Supplementary Fig. 2 | Amide bond formation in fumaryl dipeptides[18,19] and penilumamide[20] by NRPS. a, Dimodule NRPS, SidE and FtpA were responsible for biosynthesis of fumaryl pseudodipeptide fumarylalanine and fumaryltyrosine, respectively. **b**, In penilumamide precursor biosynthesis, NRPS, PlmA and PlmJ and PlmK were required for penilumamide biosynthesis. Domain highlighted in grey was proposed to be either skipped or inactive.

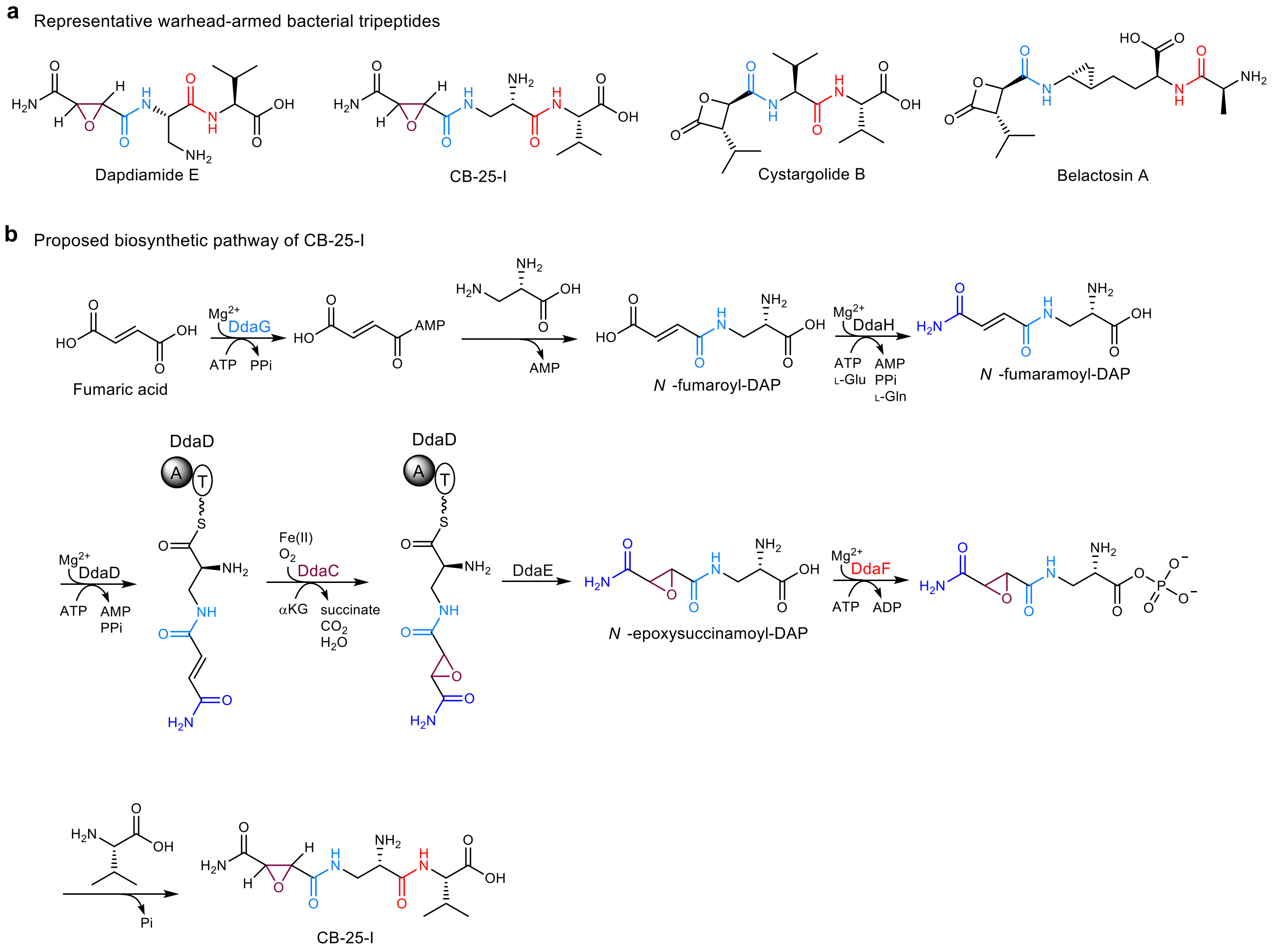

Supplementary Fig. 3 | Structure and biosynthesis of warhead-armed bacterial pseudo tripeptides. **a**, The structures of the representative warhead-armed bacterial tripeptides[21–24]. **b**, The proposed biosynthesis of the dapdiamide analog, CB-25-I[21,22]. Briefly, a ABS DdaG catalyzes the condensation of fumaric acid and 2,3-diaminopropanoate (DAP) to form N_β-fumaroyl-DAP. It should be noted that N_β-fumaroyl-DAP cannot be the substrate for a ATP-grasp enzyme DdaF to catalyze the second amide bond formation. Next immediate enzyme is a putative amidotransferase DdaH that catalyzes the amidation of N_β-fumaroyl-DAP to N_β-fumaramoyl-DAP. The resultant N_β-fumaramoyl-DAP is adenylated by DdaD (adenylation-thiolation didomain) and transferred to the thiolation domain of DdaD. Then, αKG/Fe(II)-dependent oxygenase DdaC catalyzes the epoxidation on the DdaD-tethered N_β-fumaramoyl-DAP to form the DdaD-tethered N_β-epoxysuccinamoyl-DAP. Next, N_β-epoxysuccinamoyl-DAP is released from DdaD by hydrolysis proposed to be catalyzed by a thioesterase DdaE. Lastly, DdaF catalyzes the condensation of N_β-epoxysuccinamoyl-DAP and L-valine to give CB-25-I. Note that the proposed functions of DdaH and DdaE have not been biochemically assessed. Nonetheless, given the fact that 1)DdaF requires epoxysuccinamoyl or fumaramoyl moiety for the substrate recognition and 2) the formation of N_β-epoxysuccinamoyl-DAP requires three enzyme cascade (DdaD, DdaC, and DdaE), the biocatalytic applications of the Dda enzyme system for library synthesis are inherently challenging, which requires the multiple enzymes with substrate promiscuity in addition to two amide-forming enzymes DdaG and DdaF.

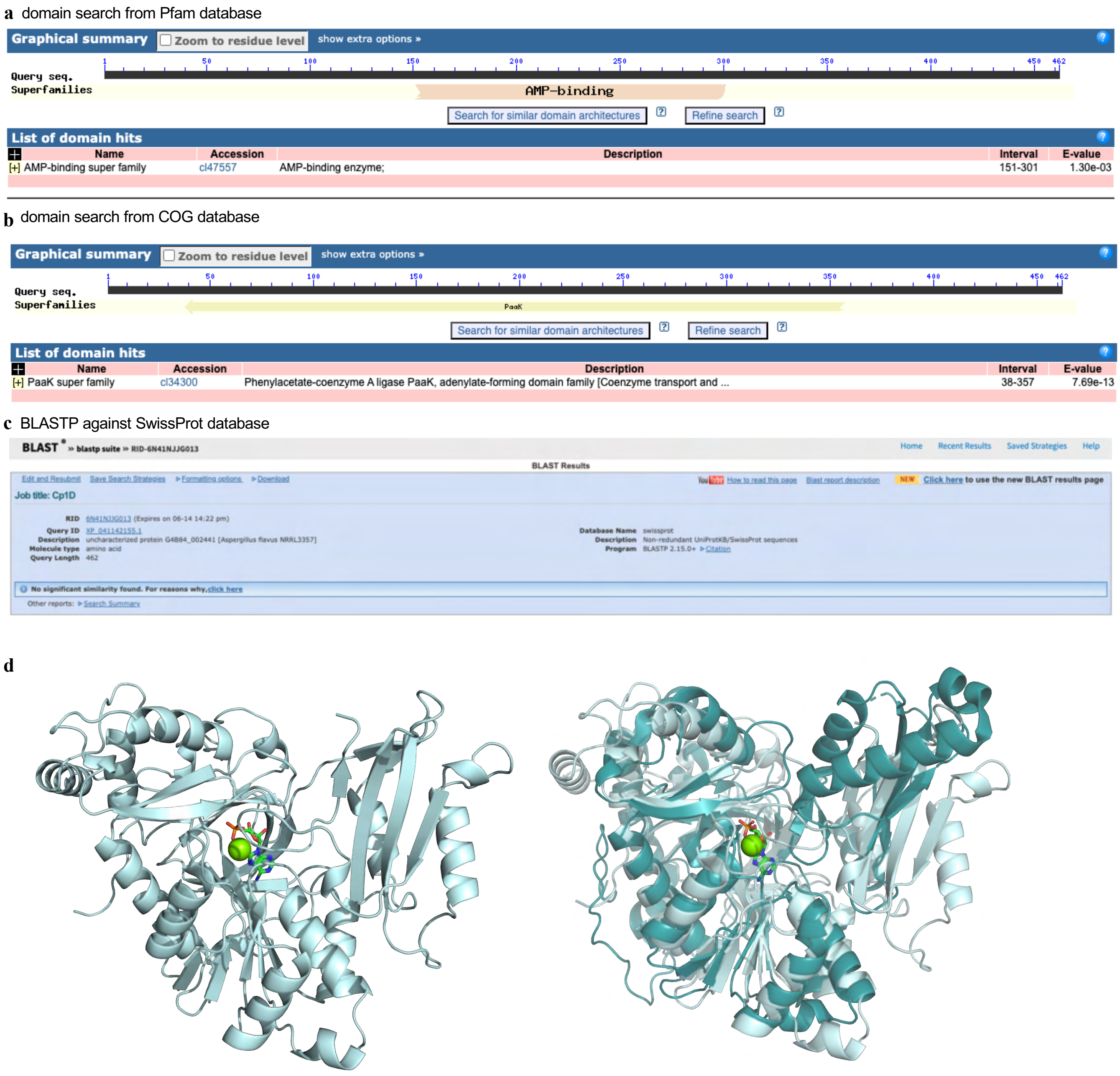

Supplementary Fig. 4 | Bioinformatic analysis of Cp1D. a. Domain search of Cp1D from Pfam database. Cp1D has the weak conservation of AMP-binding superfamily domain. **b**. Domain search of Cp1D from COG database. Cp1D belongs to the family of phenylacetate-CoA ligase, suggesting Cp1D is also an ANL-family enzyme. **c**. BLASTP search using Cp1D as the query against SwissProt database. No characterized homolog of Cp1D was identified from the database. **d**. Left, AF3[25] predicted Cp1D with AMP and Mg^{2+}. Right, overlay of Cp1D and PDB 6he0[26], shown in cartoon representation, RMSD = 5.367, (2096 to 2096 atoms by using "super" alignment method in pymol

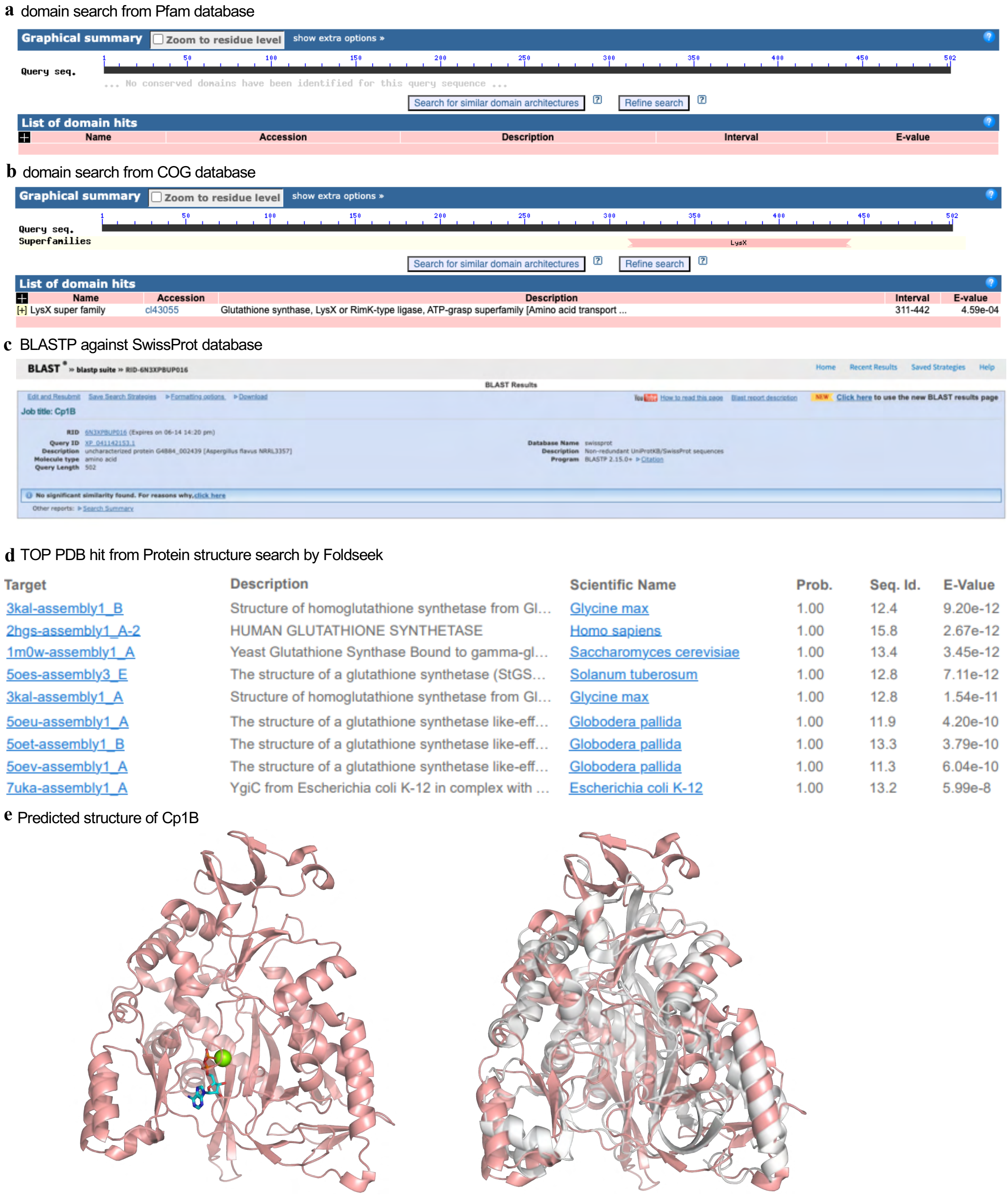

Supplementary Fig. 5 | Bioinformatic analysis of Cp1B. a, Pfam domain search for Cp1B on NCBI website. **b**, COG domain search for Cp1B on NCBI website. The small portion of Cp1B has a LysX superfamily domain with high E-value (4.59×10^{-4}). **c**, BLASTP search using Cp1B as the query against SwissProt database. No characterized homolog of Cp1B was identified from the database. **d**, Structural analysis of Cp1B/Cp2B by Foldseek[27] identified homoglutathione synthase (3KAL, PDB number)[28] from *Glycine max* as closet structure homolog. **e**. Left, AF3[25] predicted Cp1B with ADP and Mg^{2+}. Right, overlay of Cp1B and 3KAL, shown in cartoon representation, RMSD = 5.697 (2147 to 2147 atoms by using “super” align method in pymol).

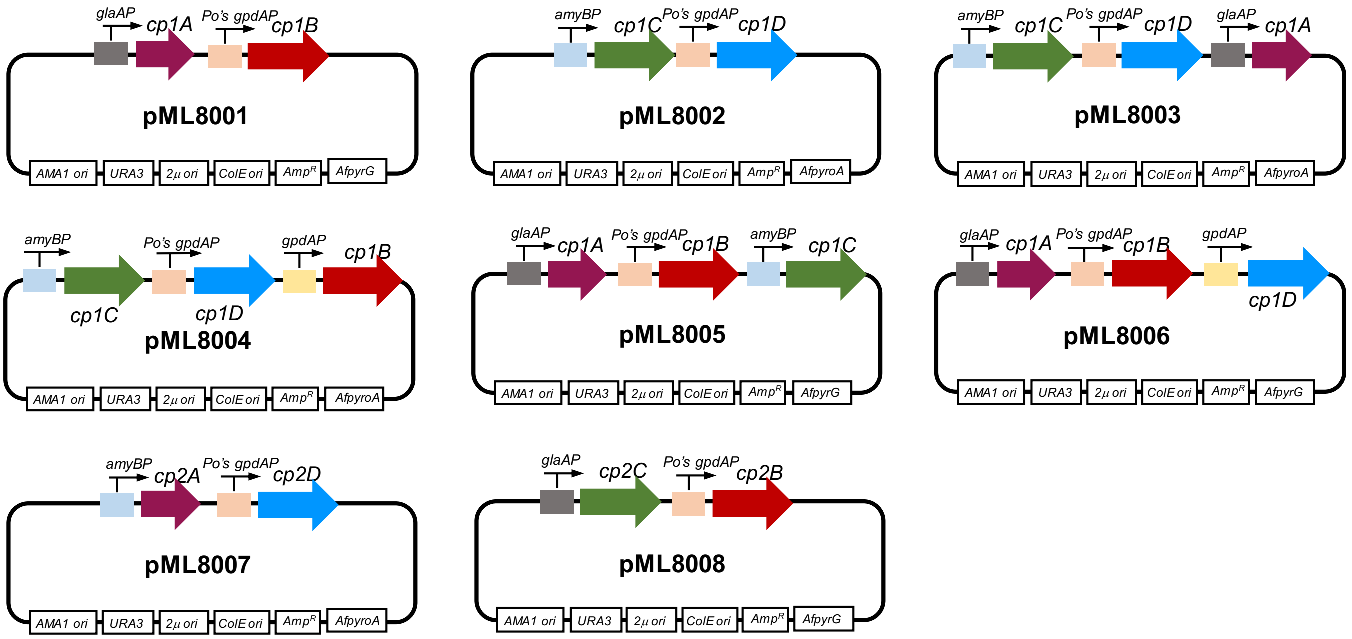

Supplementary Fig. 6 | Plasmids used for heterologous expression of *cp1* and *cp2* genes in *A. nidulans* heterologous host.

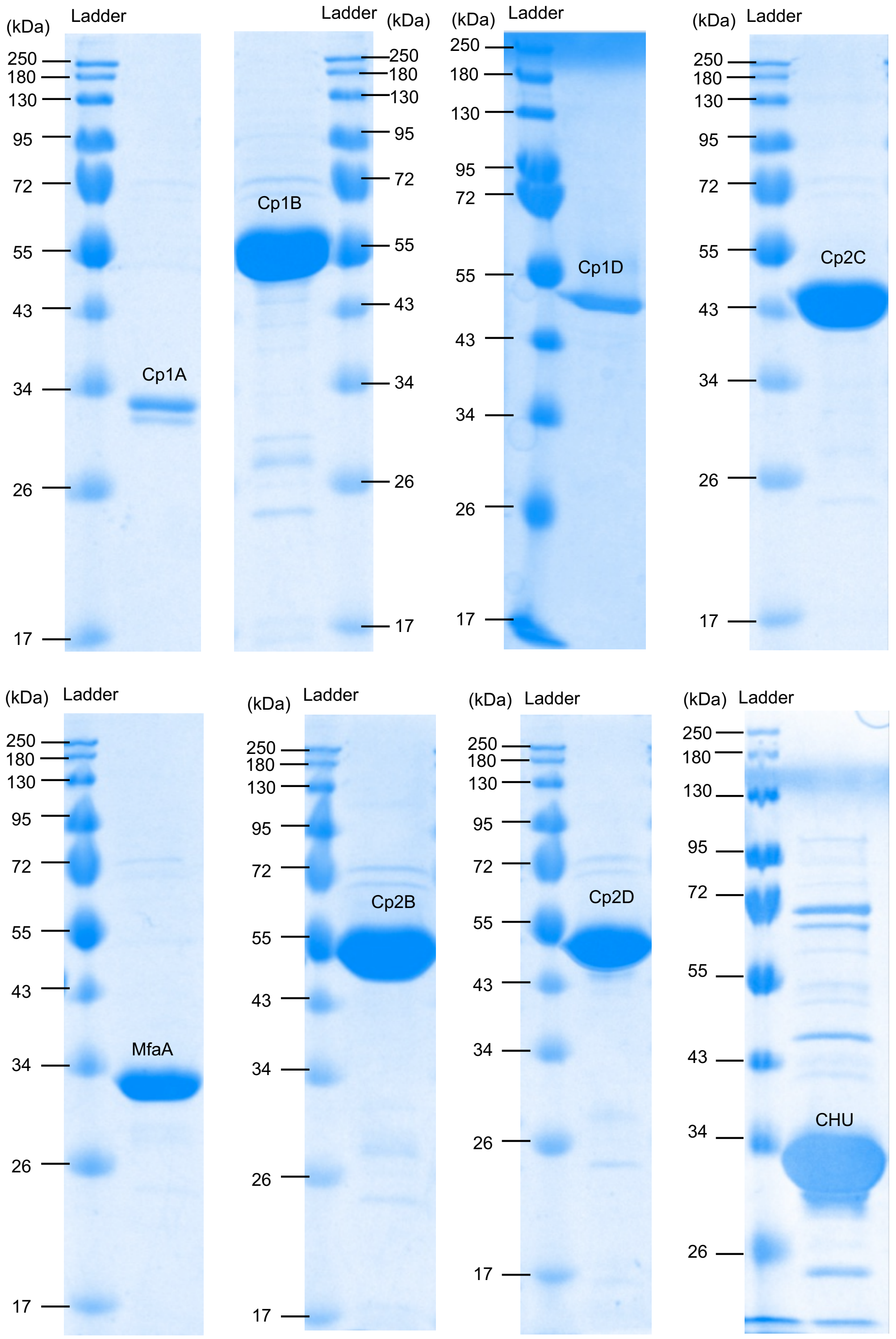

Supplementary Fig. 7 | SDS-PAGE gels of purified proteins used in this study. Expected molecular weights of Cp1A (oxygenase), Cp1B (HP), Cp1D (ABS), MfaA (oxygenase), Cp2C (decarboxylase), Cp2B (HP), Cp2D (ABS), CHU (Polyphosphate kinase) are 33 kDa, 57 kDa, 52 kDa, 32 kDa, 45 kDa, 57 kDa, 52 kDa and 36 kDa, respectively. These experiments were repeated three times independently and representative results are shown.

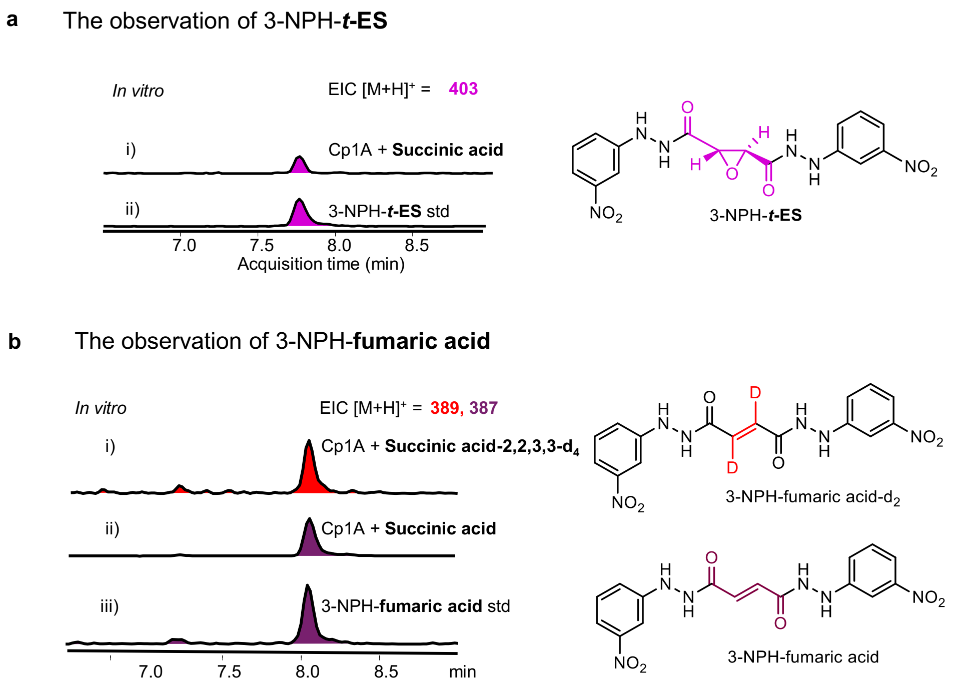

Supplementary Fig. 8 | Succinic acid can also be the substrate for Cp1A. 3-NPH-*t*-ES (Supplementary Fig. **8a**) and 3-NPH-fumaric acid (Supplementary Fig. **8b**) was observed when succinic acid as the substrate for Cp1A. Deuterium labeled 3-NPH-fumaric acid was also observed when succinic acid-2,2,3,3-d_4 as substrate. 100 μL reactions were performed at 30 °C for 3 h, in 50 mM sodium phosphate buffer (pH 8.0) containing 0.2 mM $FeSO_4$, 2 mM αKG, 2 mM ascorbate, 1 mM of substrate, and 10 μM of Cp1A. After overnight incubation at 30 °C, the product was derivatized with 3-NPH. Selected ion monitoring of 3-NPH-***t*-ES** ($[M + H]^+ = 403$), 3-NPH-**fumaric acid** ($[M + H]^+ = 387$), and 3-NPH-**fumaric acid-**d_2 ($[M + H]^+ = 389$) is shown. Y-axis represents ion counts and the chromatograms are presented on the same scale.

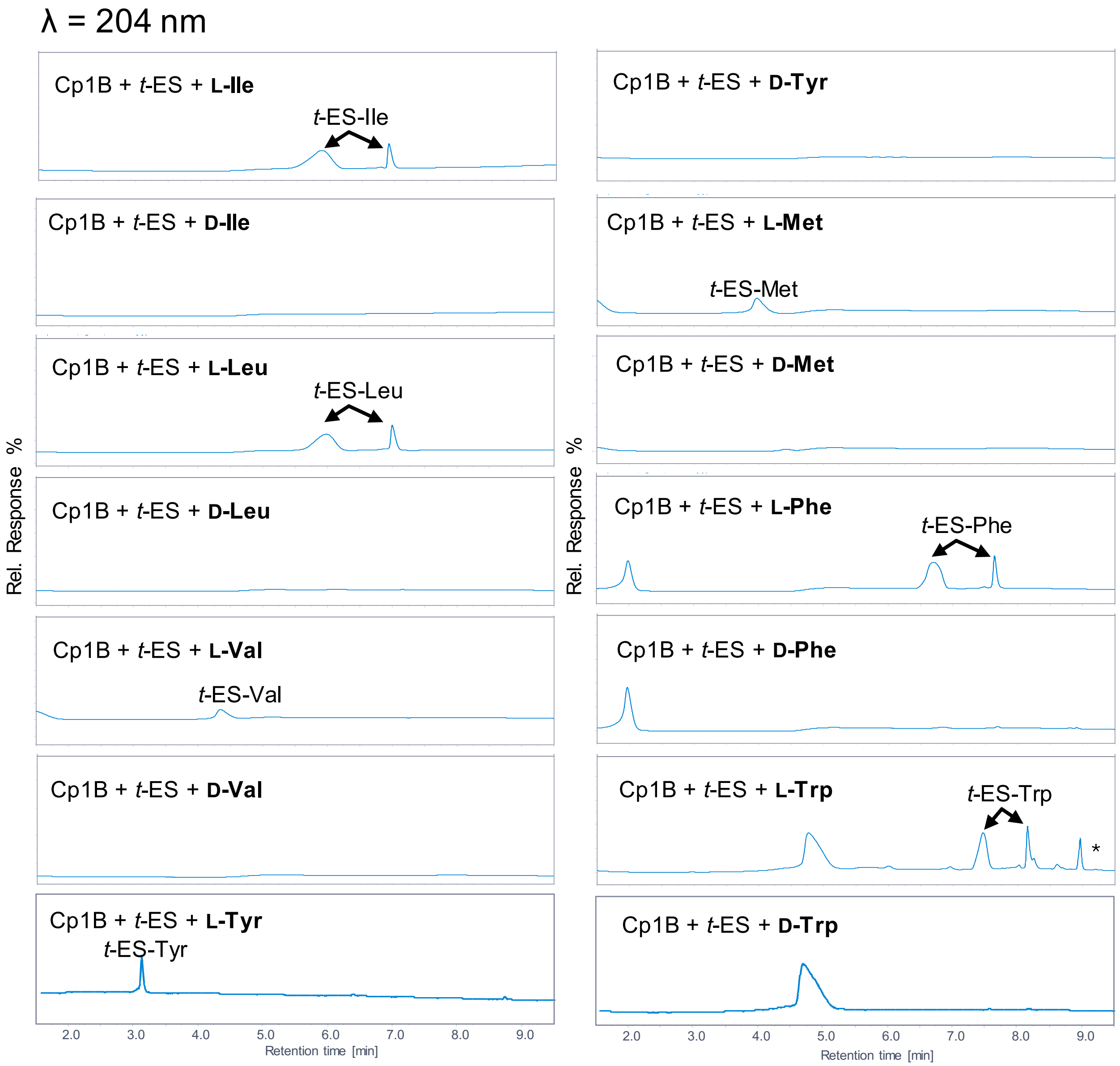

Supplementary Fig. 9 | D-amino acids are not substrate for Cp1B. LC/MS analysis of *in vitro* assay of Cp1B with hydrophobic D-amino acids. The corresponding products were not detected when D-amino acid was used. Assays were carried out with enzymes (25 µM), (±)-*trans*-epoxy-succinate (5 mM) and amino acid (2.5 mM), 10 mM $MgCl_2$, 10 mM ATP, 50 mM sodium phosphate at 30 °C for 16 h. Note: Epoxy-succinyl L-amino acids were present as two split peaks which result from different charge state (0.1% formic acid was used as the additive for LC/MS solvents). The LC-MS elution gradients method: 0-0.25 min, 1% eluent B; 0.25-13.0 min, 1-99% eluent B; 13.0-16.0 min, 99% eluent B; 16.0-18.0 min, 1% eluent B using Agilent LC/MSD iQ (AgilentTM InfinityLab Poroshell 120 Aq-C18, 2.7 µm, 100 Å, 2.1 × 100 mm).

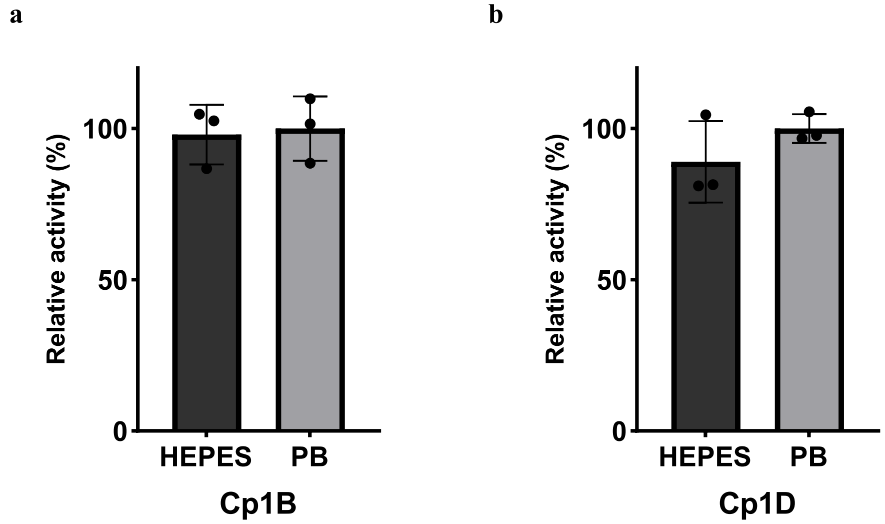

Supplementary Fig. 10 | Effect of in vitro enzymatic activity toward Cp1B or Cp1D in different buffers. a. Assays were carried out for 5 min at 30 °C with 2.5 μM Cp1B, 5 mM (±)-***t*-ES**, 2.5 mM L-Phe, 10 mM $MgCl_2$, and 10 mM ATP in 100 μL of 50 mM sodium phosphate buffer (pH 8.0) or 50 mM HEPES buffer (pH 8.0). **b.** Assays were carried out for 5 min at 30 °C with 2.5 μM Cp1D, 2 mM **15**, 5 mM octylamine, 10 mM $MgCl_2$, and 10 mM ATP in 100 μL of 50 mM sodium phosphate buffer (pH 8.0) or 50 mM HEPES buffer (pH 8.0). Y-axis represents relative activity of Cp1B (set the activity in sodium phosphate buffer (PB) as 100%) derived from the peak area of the corresponding products at λ=204 nm. Values and error bars represent the average and s.d. of three independent replicates (black filled circles), respectively ($n = 3$).

a

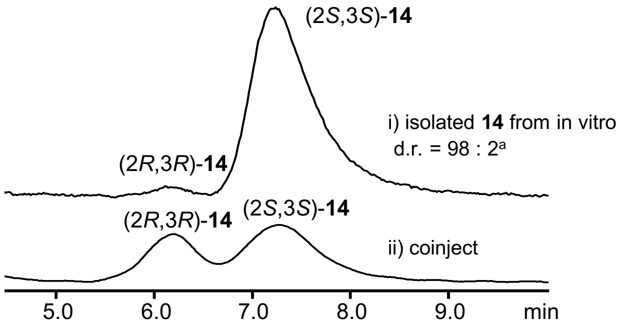

b

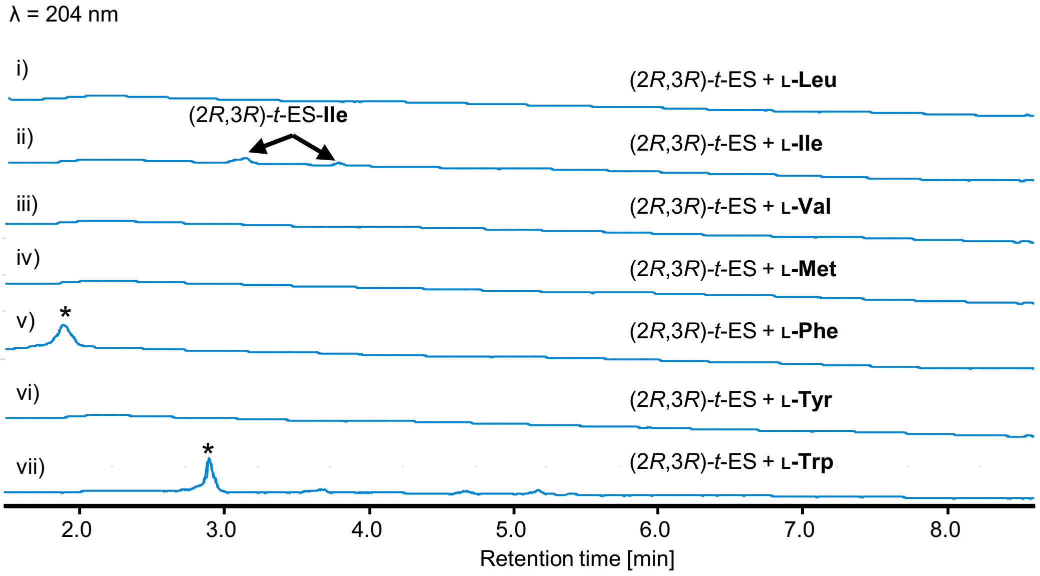

Supplementary Fig. 11 | Examination of the kinetic resolution potential for Cp1B. a. i) The HPLC chromatogram for ***t*-ES**-L-Ile (**14**) that was enzymatically prepared from racemic ***t*-ES** with L-Ile. Diasteromeric ratio (d.r.) was calculated from HPLC peak area ratio at 204 nm using chiral analytical HPLC with a CHIRALPAK® IA-3 (150 x 4.6 mm, 3 µm) at room temperature (flow rate 1 mL/min, 40% MeCN–H_2O with 0.1% trifluoroacetic acid). ii) the coinjection of (2*S*,3*S*)-***t*-ES**-L-Ile (**14**) and (2*R*,3*R*)-**14**. **b**. (2*R*,3*R*)-***t*-ES** test with proteinogenic amino acids. Assays were carried out with 25 µM enzymes, 2.5 mM (2*R*,3*R*)-***t*-ES** and 2.5 mM amino acid, 10 mM $MgCl_2$, 10 mM ATP in 100 µL of 50 mM sodium phosphate at 30 °C for 16 h. While only in vitro assay with L-Ile gave trace amount pseudopeptides, no product can be observed when tested with other L-amino acids. The LC-MS elution gradients method: 0-0.25 min, 1% eluent B; 0.25-13.0 min, 1-99% eluent B; 13.0-16.0 min, 99% eluent B; 16.0-18.0 min, 1% eluent B using Agilent LC/MSD iQ (AgilentTM InfinityLab Poroshell 120 Aq-C18, 2.7 µm, 100 Å, 2.1 × 100 mm). *: amino acid substrate.

a. chiral resolution by L-arginine (Kanaoka, Hesse)

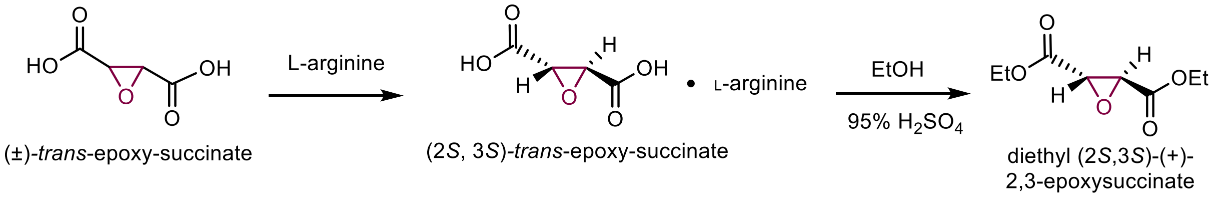

b. stereoselective synthesis (Bogyo)

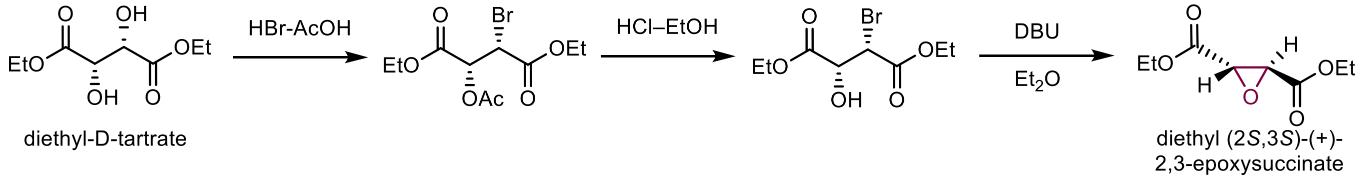

Supplementary Fig. 12 | Previous work for the preparation of (2*S*,3*S*)-*trans*-epoxy-succinic acid[29–31]. **a**. The reported preparation of diethyl (2*S*,3*S*)-(+)-2,3-epoxysuccinate through chiral resolution is shown. **b**. The reported stereoselective synthesis of diethyl (2*S*,3*S*)-(+)-2,3-epoxysuccinate is shown.

a Proposed binding sites for the substrate

b Cp1B overlay with Cp2B

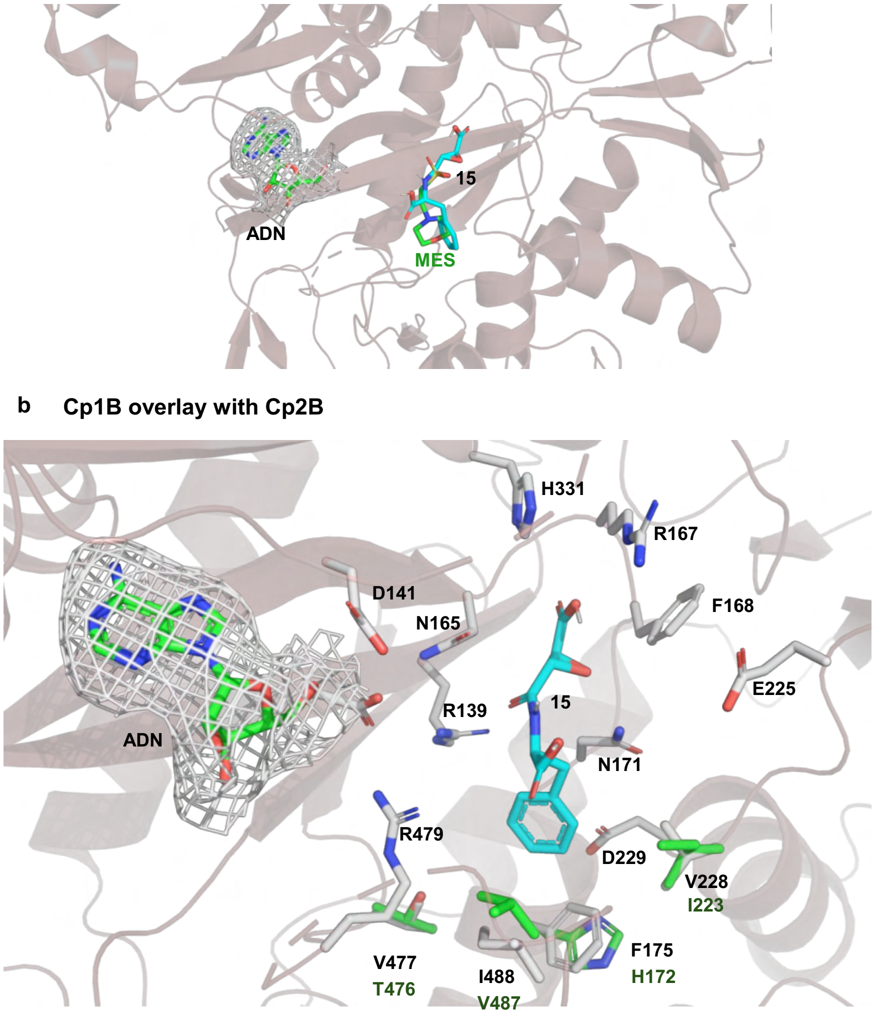

Supplementary Fig. 13 | Docking simulation of 15 with Cp1B and comparison of residues forming hydrophobic pocket between Cp1B and Cp2B. **a.** Docking simulation of **15** with the Cp1B active site was performed using Autodock Vina[32]. One of possible binding modes for **15** based on the MES binding is shown. **15** is colored in cyan. MES is colored in green. **b.** The surrounding residues of **15** are colored in white and green for Cp1B and Cp2B, respectively. Based on the docking simulation, the side chain of L-Phe can also be housed in the same hydrophobic pocket formed by F175, V228, V477, and I488, suggesting the hydrophobic pocket accommodates the side chain of L-amino acid moiety. R167 likely anchors **15** through the hydrogen bonding with the carboxylate of ***t*-ES** in **15** (The distance between oxygen atom of the carboxylate and guanidino nitrogen is 3 to 4 Å), which could be supported by the result where the mutation of R167 to alanine decreases the enzymatic activity (see **Extended Data Fig. 6d**). The epoxide moiety of ***t*-ES** in **15** forms hydrogen bonding with the amide side chain of N171, which is consistent with the mutagenesis results where N171A mutant significantly attenuates the enzymatic activity of Cp1B. Notable difference of the residues between Cp1B and Cp2B can be found in the residues forming the hydrophobic pocket. The corresponding residues of Cp2B to F172, V228, V477, and I488 in Cp1B are

H172, I223, T476, V487. Given the clear preference of L-Tyr for Cp2B as the amino acid donor over Cp1B, H172 might have a critical role in recognizing the phenolic alcohol in L-Tyr through hydrogen bonding.

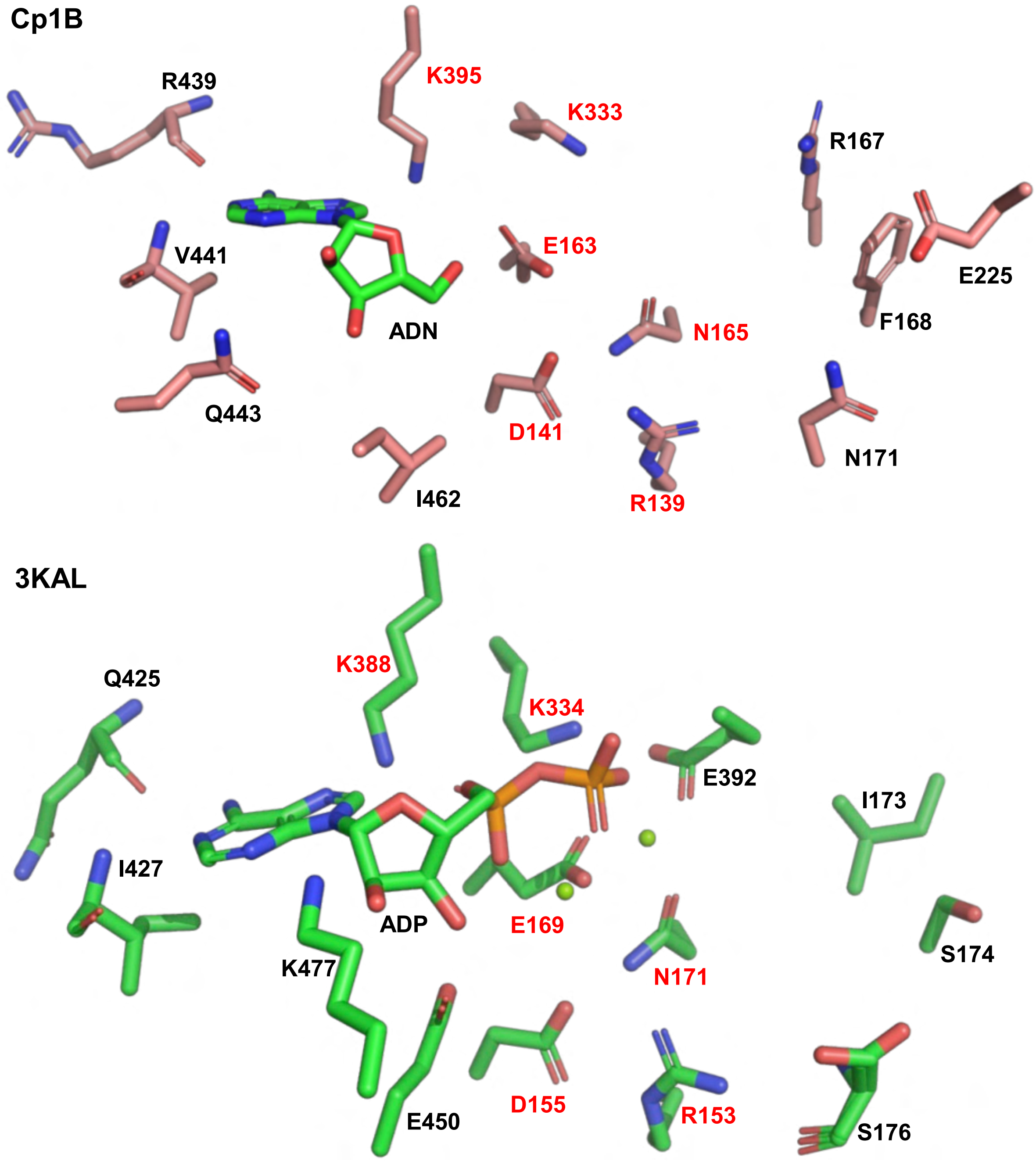

Supplementary Fig. 14 | The comparison of nucleotide binding mode and surrounding residues between Cp1B and homoglutathione synthetase (PDB:3KAL)[28]. Adenosine (ADN) and ADP in Cp1B and 3KAL are both surrounded by the conserved residues R139 (3KAL:R153), D141 (D155), E163 (E169), and N165 (N171) which are likely involved in the phosphorylation of the carboxylate in substrate. K333 (K334) and K395 (K388) are also conserved in 3KAL and Cp1B.

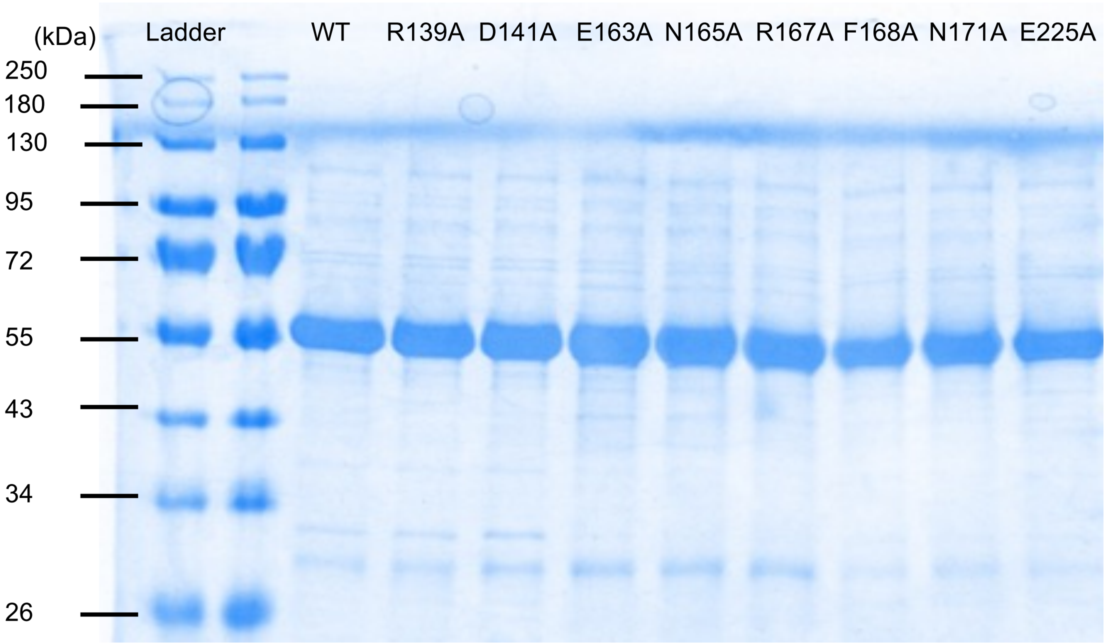

Supplementary Fig. 15 | SDS-PAGE gels of Cp1B mutants.

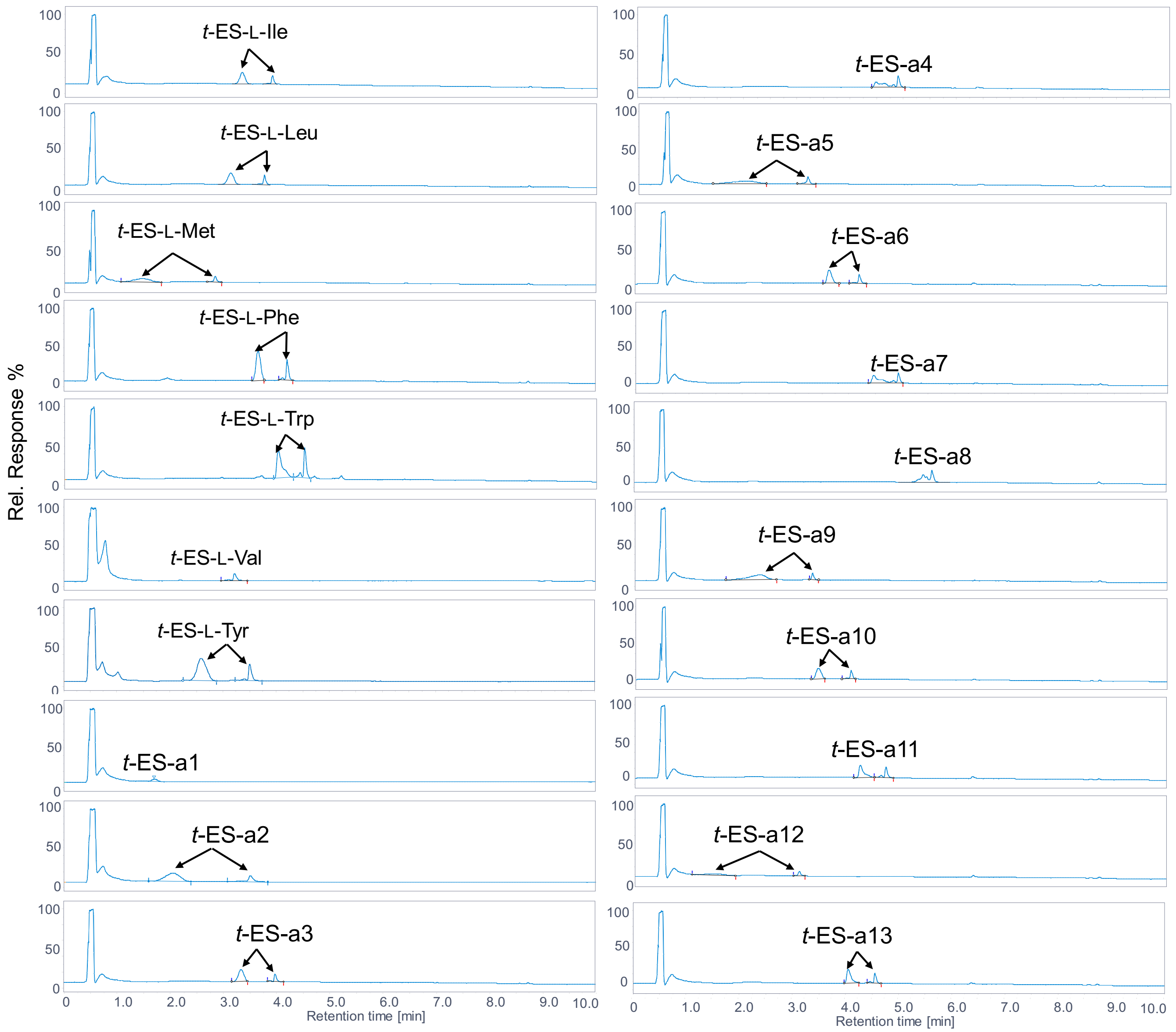
t-ES-L-Ile
t-ES-L-Leu
t-ES-L-Met
t-ES-L-Phe
t-ES-L-Trp
t-ES-L-Val
t-ES-L-Tyr
t-ES-a1
t-ES-a2
t-ES-a3
t-ES-a4
t-ES-a5
t-ES-a6
t-ES-a7
t-ES-a8
t-ES-a9
t-ES-a10
t-ES-a11
t-ES-a12
t-ES-a13
Rel. Response %
Retention time [min]

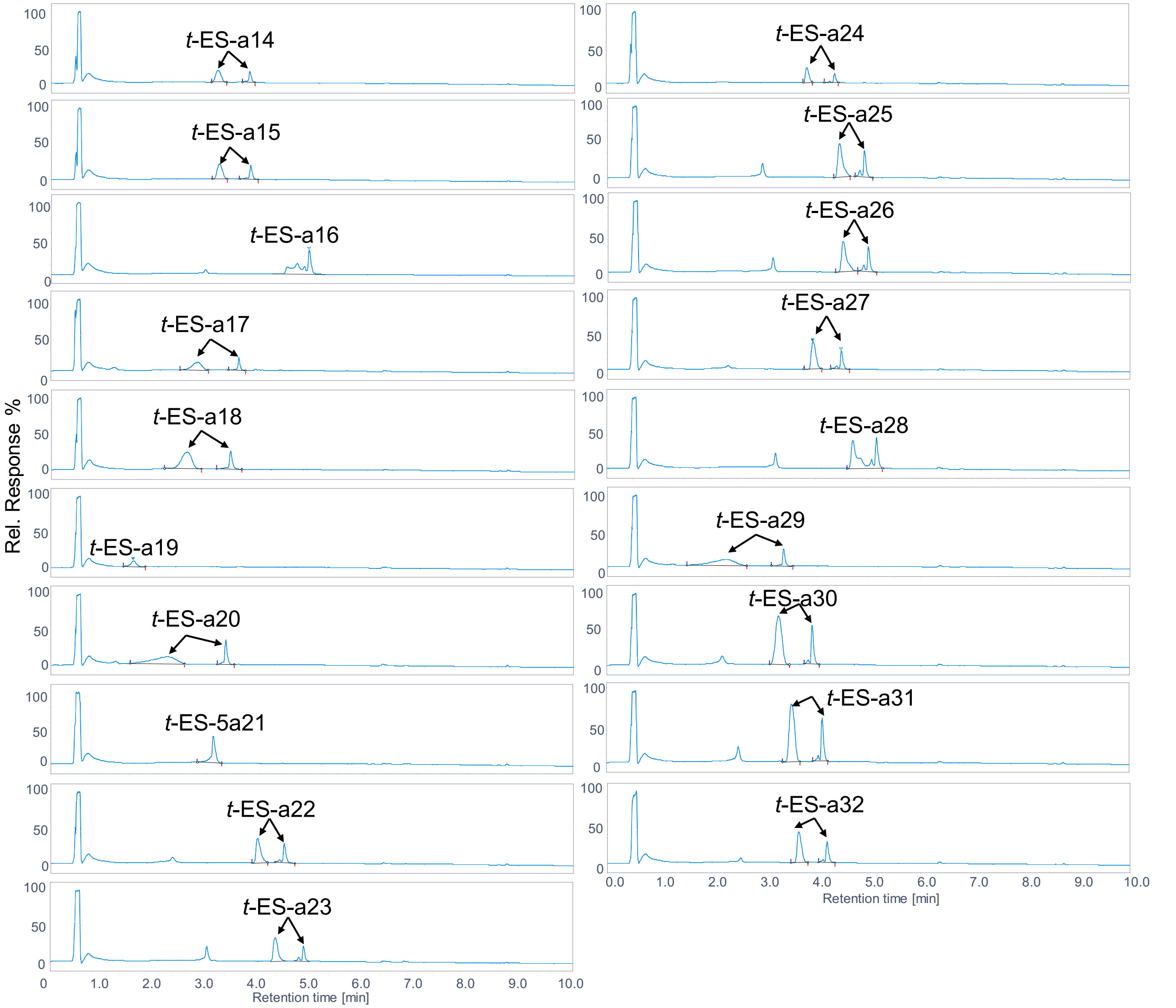

Supplementary Fig. 16 | HPLC traces of (2*S*,3*S*)-*t*-ES-amino acids formed by the Cp1B reaction. Substrate scope test for Cp1B against coupling of (±)-***t*-ES** with different proteinogenic and non-proteinogenic amino acids. Cp1B was found to accept hydrophobic L-amino acids (I, L, M, F, W, V, Y, a1-a32). Assays were carried out with 25 μM enzymes, 5 mM (±)-***t*-ES** and 2.5 mM amino acid, 10 mM $MgCl_2$, 10 mM ATP in 100 μL of 50 mM sodium phosphate buffer (pH 8.0) at 30 °C for 16 h. Note: the corresponding products were present as two split peaks which result from different charge state of the product (0.1% formic acid was used as the additive for LC/MS solvents). Only one peak was observed during compound isolation by HPLC as 0.1% trifluoroacetic acid was added to eluents. Analytical % yields were estimated based on the standard curve of **14** or the enzymatically synthesized standard at λ 204 nm. The LC/MS elution gradients method: 0-0.25 min, 1% eluent B; 0.25-13.0 min, 1-99% eluent B; 13.0-16.0 min, 99% eluent B; 16.0-18.0 min, 1% eluent B using Agilent LC/MSD iQ (AgilentTM InfinityLab Poroshell 120 Aq-C18, 2.7 μm, 100 Å, 2.1 × 100 mm).

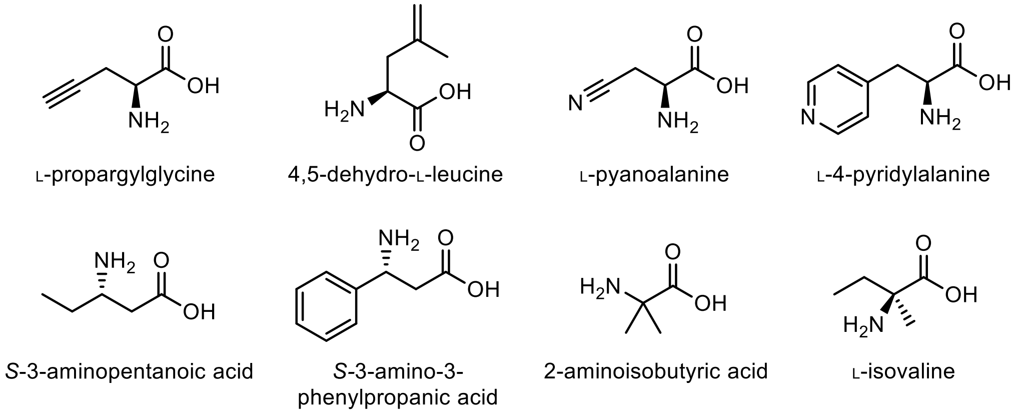

Supplementary Fig. 17 | Structures of non-proteinogenic amino acids tested but could not be accepted by Cp1B/Cp2B. Amino acids with double bond, triple bond, nitrile, pyridine are disfavored. α,α-disubstituted amino acid, *N*-methylated amino acid was also not preferred.

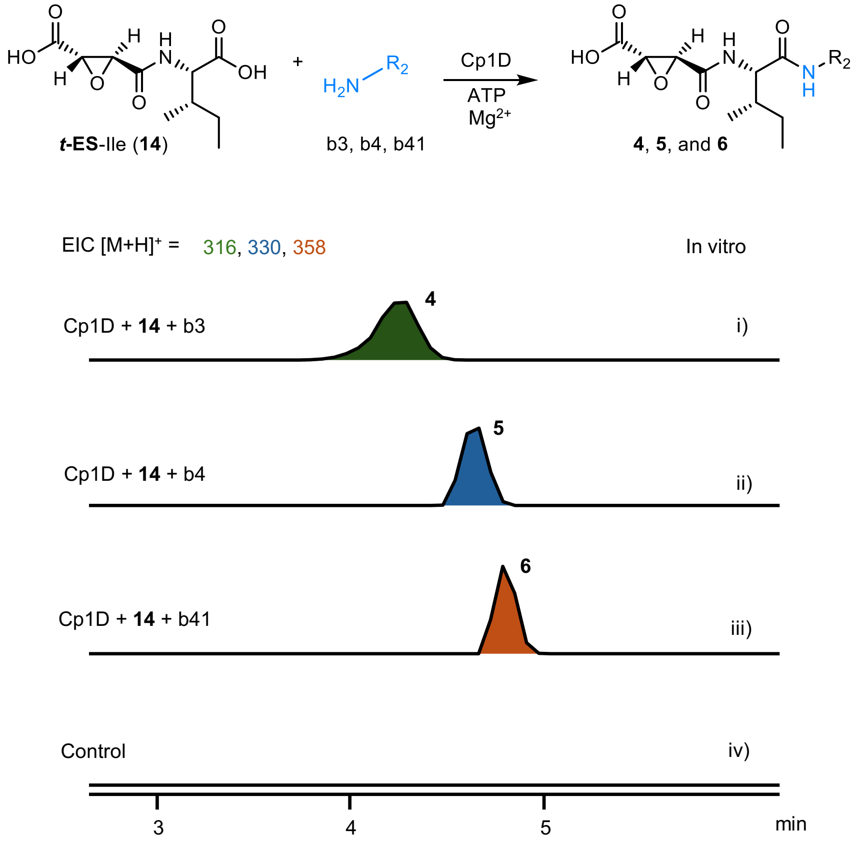

Supplementary Fig. 18 | In vitro reaction of Cp1D with 14 and amines. LC/MS analysis of in vitro assay of Cp1D with **14** and putrescine (b3, i), cadaverine (b4, ii), and agmatine (b41, iii). The exclusive formation of **4** ($[M+H]^+$ = 316), **5** ($[M+H]^+$ = 330) and **6** ($[M+H]^+$ = 358) was detected. The assays were carried out with 10 µM Cp1D, 2.5 mM **14**, 10 mM ATP, 10 mM $MgCl_2$, 2.5 mM amine donor (b3, b4, and b41) in 100 µL of 50 mM phosphate buffer (pH 8.0) at 30 °C for 16 h. Y-axis represents ion counts and the chromatograms are presented on the same scale.

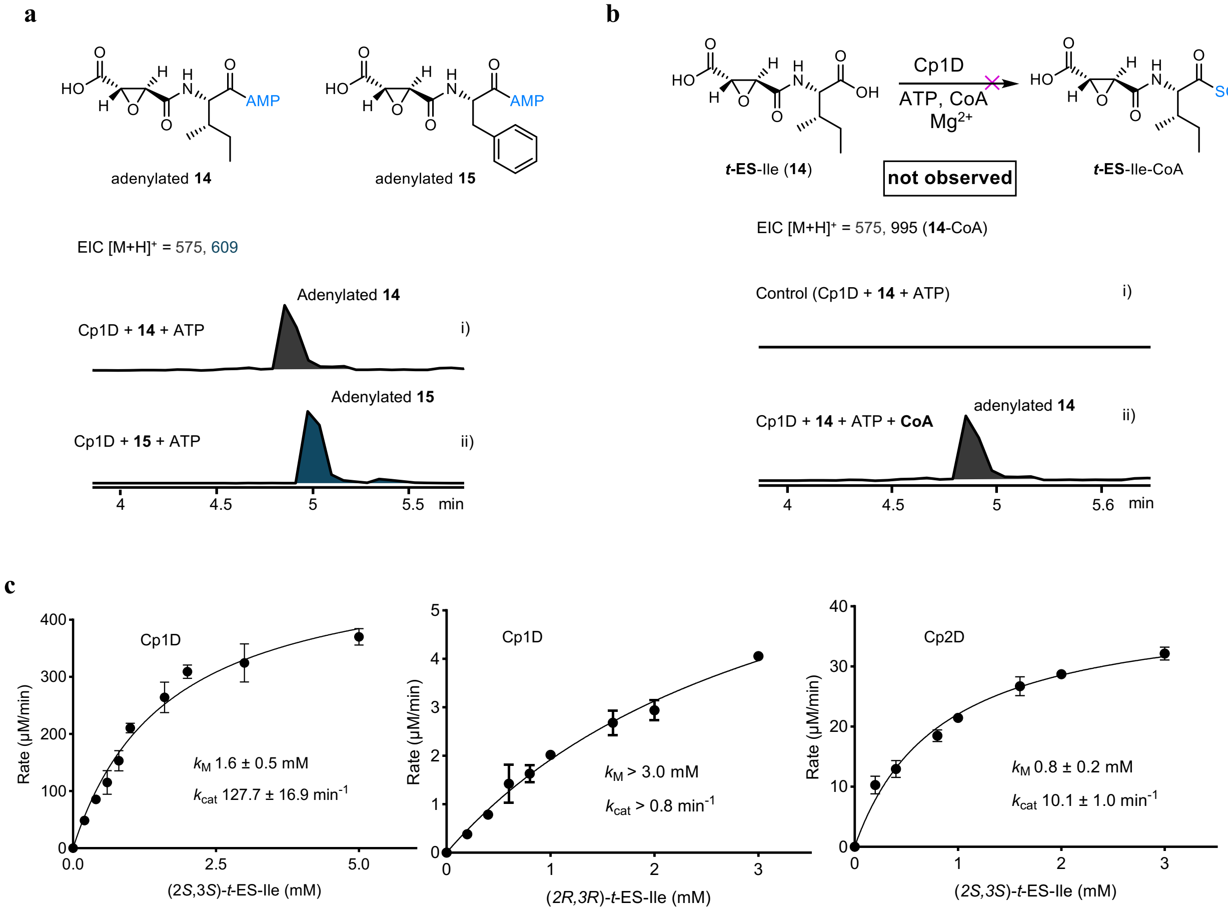

Supplementary Fig. 19 | Cp1D is a new family of diamide-forming amide bond synthetase not CoA ligase. a. Cp1D activity assay with (2*S*,3*S*)-***t*-ES**-Ile (**14**), and (2*S*,3*S*)-***t*-ES**-Phe (**15**) as substrate, the MS peak of adenylated **14** (*m/z* 575 for $[M+H]^+$) and adenylated **15** (*m/z* 609 for $[M+H]^+$) was observed. Assays were carried out with 25 μM enzymes, 0.4 mM **14** or **15**, 10 mM $MgCl_2$, 10 mM ATP in 100 μL of 50 mM sodium phosphate (pH 8.0). Reactions were analyzed by UPLC-MS after incubation at 30 °C for 10 min. Y-axis represents ion counts and the chromatograms are presented on the same scale. **b**. Assays were carried out with 25 μM enzymes, 0.4 mM **14**, 1 mM CoA-SH, 10 mM $MgCl_2$, 10 mM ATP in 100 μL of 50 mM sodium phosphate (pH 8.0). Reactions were analyzed by UPLC-MS after incubation at 30 °C for 1 h. Selected ion chromatography of ***t*-ES**-Ile-CoA (m/z 995 for $[M+H]^+$) and adenylated **14** (m/z 575 for $[M+H]^+$) is shown. The MS peak of ***t*-ES**-Ile-CoA was not observed. Y-axis represents ion counts and the chromatograms are presented on the same scale. **c**. Apparent Michaelis-Menten plots for the Cp1D and Cp2D catalyzed amidation with single diastereomer (2*S*,3*S*)-***t*-ES**-Ile or (2*R*,3*R*)-***t*-ES**-Ile. Briefly, 100 μL of reaction mixture, 50 mM sodium phosphate (pH 8.0), 10 mM $MgCl_2$, 10 mM ATP, 5 mM agmatine, 4 μM Cp1D, various concentrations of the substrate, were prepared. The reaction was incubated at 30 °C for 5 min and quenched with 100 μL of MeCN. The kinetic constants are derived from the formation of the corresponding product and shown as an apparent value. The values represent means ± s.d., and error bars indicate s.d. of three independent replicates (*n* = 3).

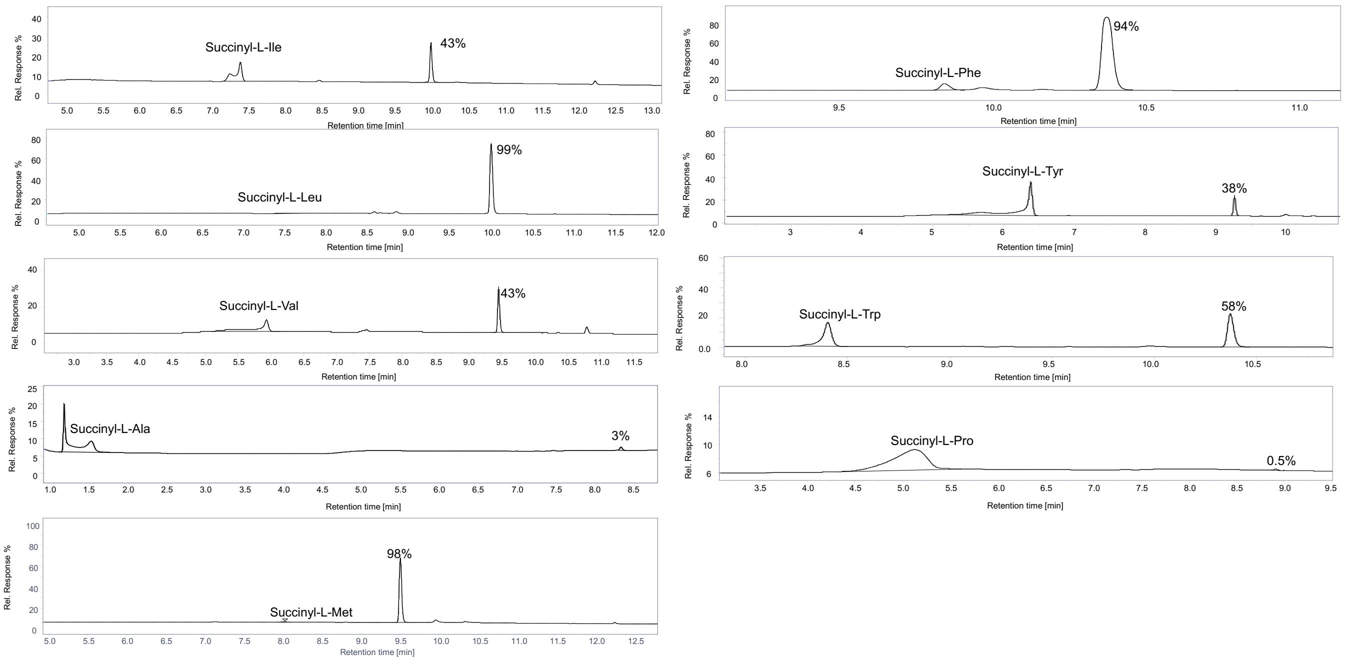

Supplementary Fig. 20 | Substrate scope test for Cp1D against coupling of isopentylamine with 20 synthesized *N*-succinyl proteinogenic amino acids. Cp1D was found to accept *N*-succinyl proteinogenic amino acids with hydrophobic amino acids. Assays were carried out with 25 µM Cp1D, 2 mM *N*-succinyl proteinogenic amino acid and 5 mM isopentylamine, 10 mM $MgCl_2$, 10 mM ATP in 100 µL of 50 mM sodium phosphate buffer (pH 8.0). Reactions were analyzed by LC/MS after incubation at 30 °C for 16 h. Analytical % conversion of *N*-succinyl proteinogenic amino acid to the amide products was calculated using HPLC peak area ratios at λ 204 nm of product and substrate (% Conversion = (peak area of product / (peak area of substrate + peak area of product)) × 100%). The LC-MS elution gradients method: 0-0.25 min, 1% eluent B; 0.25-13.0 min, 1-99% eluent B; 13.0-16.0 min, 99% eluent B; 16.0-18.0 min, 1% eluent B using Agilent LC/MSD iQ (AgilentTM InfinityLab Poroshell 120 Aq-C18, 2.7 µm, 100 Å, 2.1 × 100 mm).

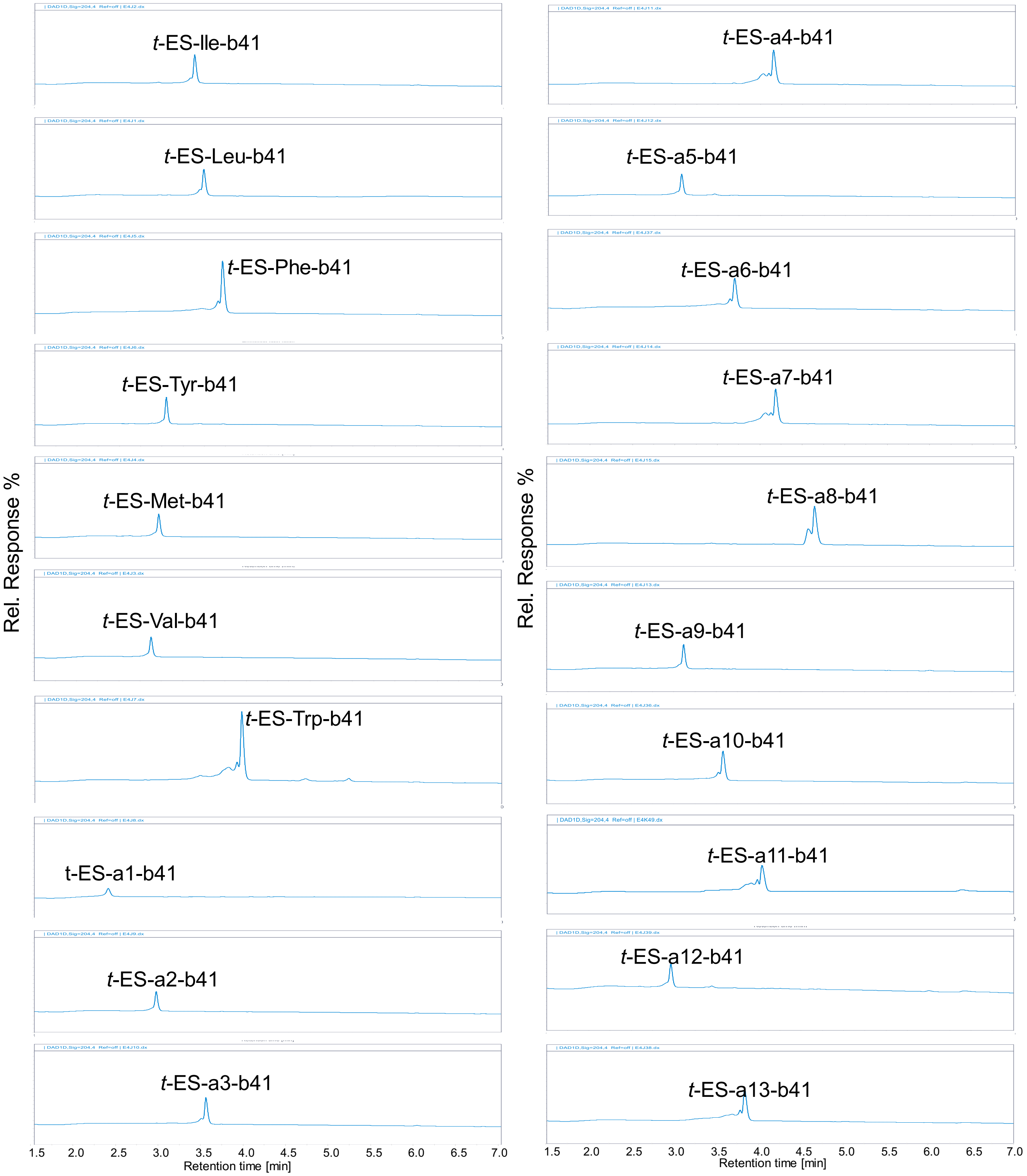
t-ES-Ile-b41
t-ES-Leu-b41
t-ES-Phe-b41
t-ES-Tyr-b41
t-ES-Met-b41
t-ES-Val-b41
t-ES-Trp-b41
t-ES-a1-b41
t-ES-a2-b41
t-ES-a3-b41
t-ES-a4-b41
t-ES-a5-b41
t-ES-a6-b41
t-ES-a7-b41
t-ES-a8-b41
t-ES-a9-b41
t-ES-a10-b41
t-ES-a11-b41
t-ES-a12-b41
t-ES-a13-b41
Rel. Response %
Rel. Response %
1.5 2.0 2.5 3.0 3.5 4.0 4.5 5.0 5.5 6.0 6.5 7.0
Retention time [min]
1.5 2.0 2.5 3.0 3.5 4.0 4.5 5.0 5.5 6.0 6.5 7.0
Retention time [min]

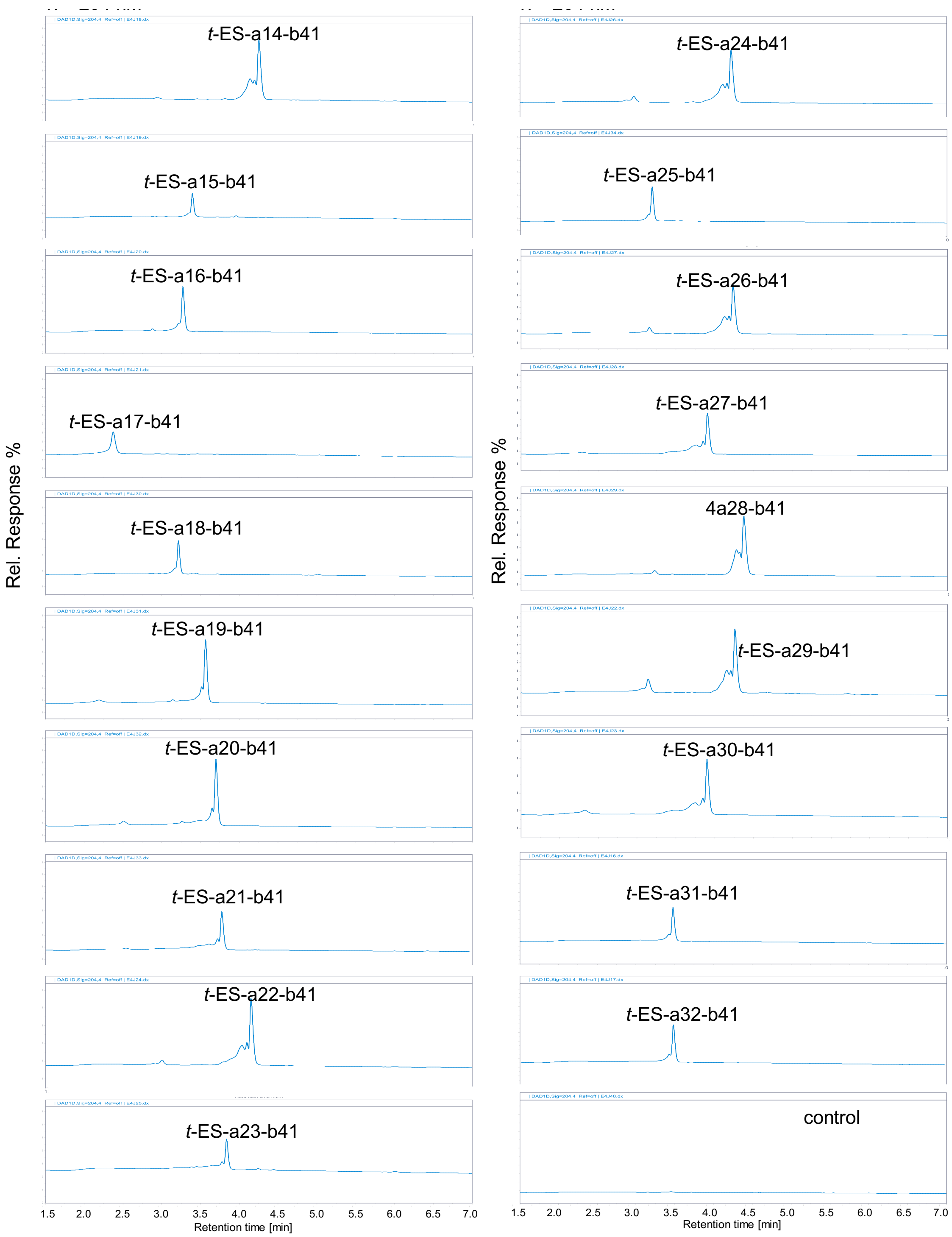

Supplementary Fig. 21 | HPLC traces of combinatorial biocatalysis with agmatine and different L-amino acids. Assays were carried out with 25 μM Cp1B, 25 μM Cp1D, 2 mM (±)-***t*-ES**, 1 mM amino acid, 1 mM agmatine (b41) in 100 μL of 50 mM sodium phosphate buffer (pH 8.0) at 30 °C for 16 h. The LC/MS elution gradients method: 0-0.25 min, 1% eluent B; 0.25-13.0 min, 1-99% eluent B; 13.0-16.0 min, 99% eluent B; 16.0-18.0 min, 1% eluent B using Agilent LC/MSD iQ (AgilentTM InfinityLab Poroshell 120 Aq-C18, 2.7 μm, 100 Å, 2.1 × 100 mm).

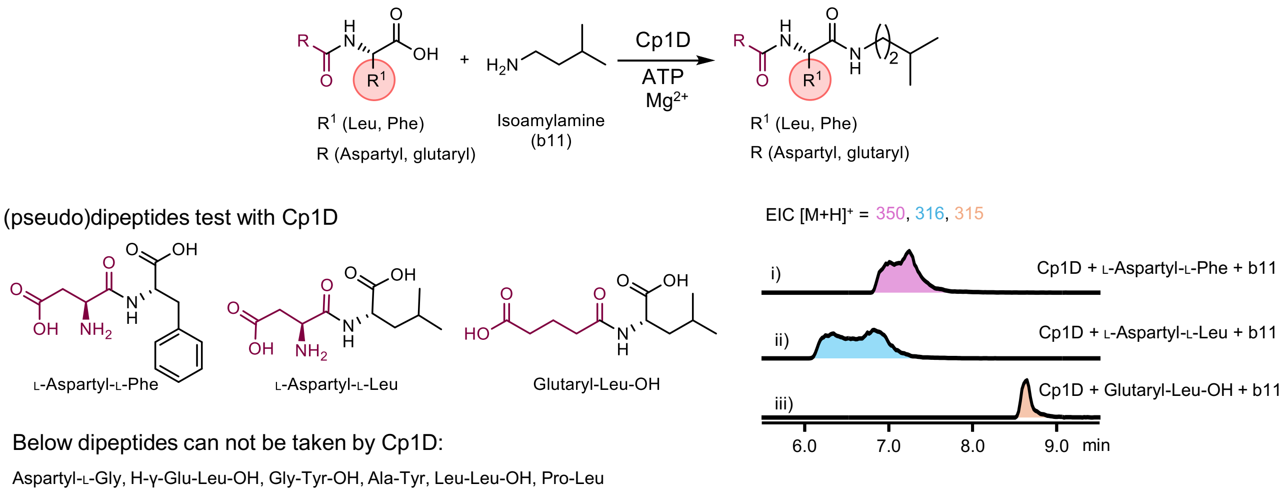

Supplementary Fig. 22 | Structures of (pseudo)dipeptides tested for the acid acceptor of Cp1D. Assays were carried out with 25 µM Cp1D, 2 mM dipeptide and 5 mM isopentylamine, 10 mM $MgCl_2$, 10 mM ATP in 100 µL of 50 mM sodium phosphate buffer (pH 8.0) at 30 ºC for 16 h.. LC/MS analysis of in vitro reaction of Cp1D with the (pseudo)dipeptides and isoamylamine (isopentylamine). EICs for L-Aspartyl-L-Phe, L-Aspartyl-L-Leu, and Glutaryl-L-Leu are $[M+H]^+ = 350$, $[M+H]^+ = 316$, and $[M+H]^+ = 315$, respectively. Y-axis represents ion counts and the chromatograms are presented on the same scale.

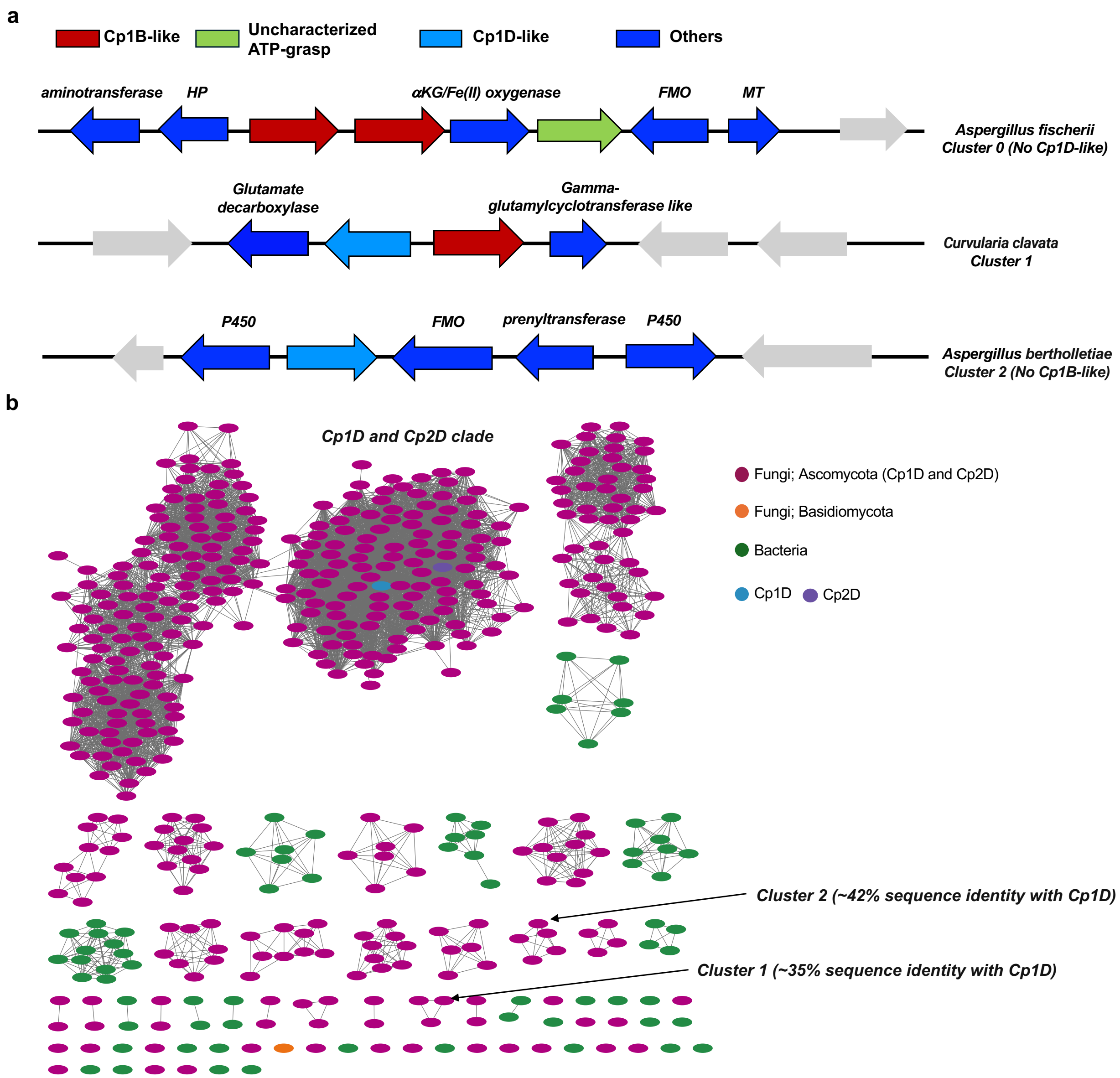

Supplementary Fig. 23 | Non-E-64 biosynthetic gene clusters containing homologs of Cp1B and Cp1D. a, The BGCs with Cp1B-like and/or Cp1D-like enzymes could be found in fungi such as *Aspergillus fischerii* (Cluster 0), *Curvularia clavata* (Cluster 1), *Aspergillus bertholletiae* (Cluster 2). These cryptic clusters such as Cluster 0 encode one or multiple ATP-grasp enzyme homologous to Cp1B, and the presence of multiple amide bond forming enzyme or oxidation enzyme implies that these clusters may code new amide-containing compounds with oxidative modifications. **b**, Sequence similarity network (SSN)[33] of Cp1D and homologues. Protein sequences (maximum 1000) were obtained by performing a BLASTp with query e-value of 5 on the EFI-EST website. The SSN of Cp1D was generated with alignment score threshold of 160. The analysis of SSN led to identify new BGCs such as Cluster 1 and Cluster 2 which potentially code new amide-containing natural products. As similar to the SSN of Cp1B (**Extended Data Fig. 2c**), Cp1D-like enzymes could also be found in the bacterial genomes.

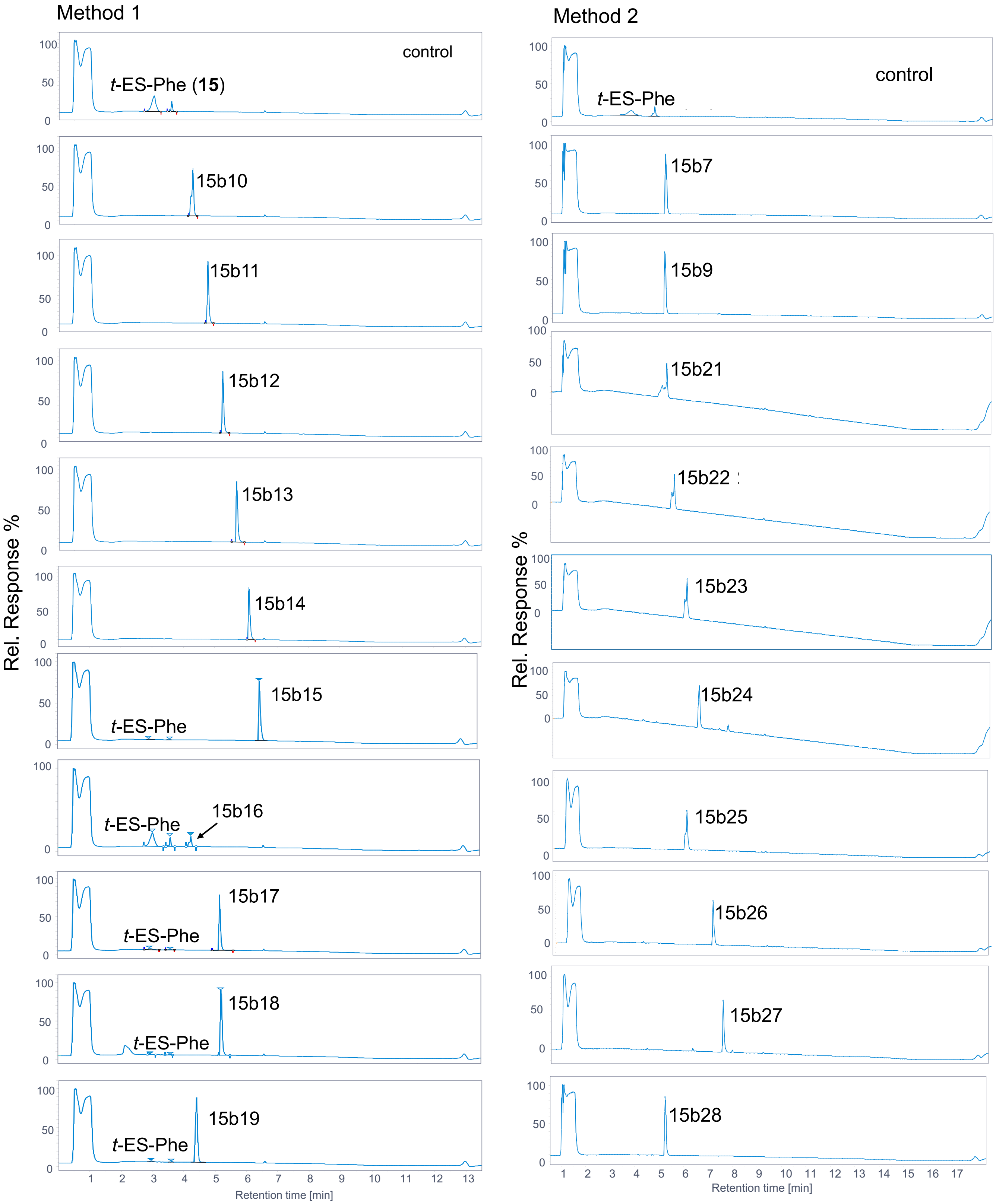

Method 1
Method 2
control
t-ES-Phe (15)
t-ES-Phe
15b10
15b11
15b12
15b13
15b14
15b15
15b16
15b17
15b18
15b19
15b7
15b9
15b21
15b22
15b23
15b24
15b25
15b26
15b27
15b28
Rel. Response %
Retention time [min]

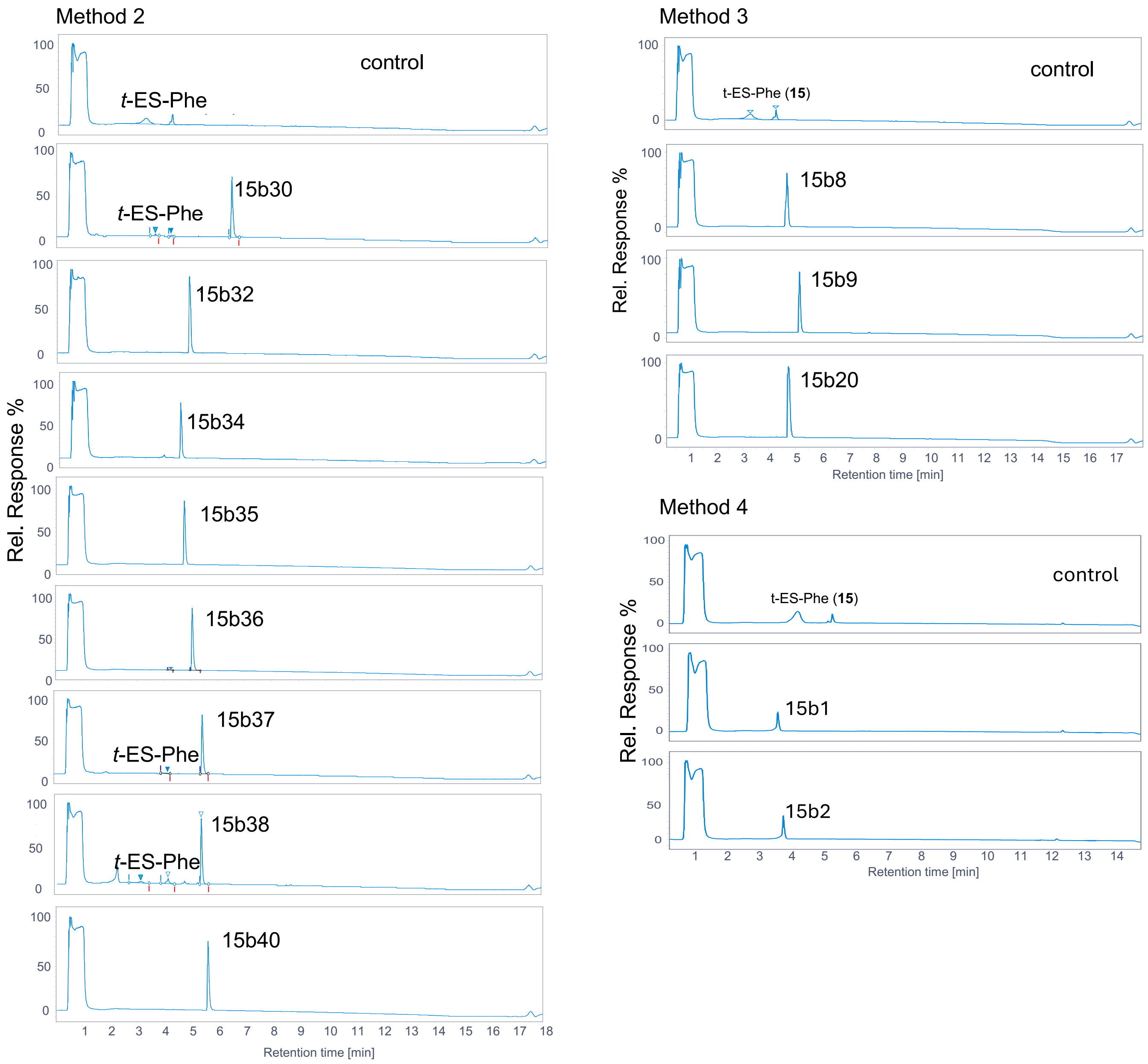

Method 2
control
t-ES-Phe
15b30
15b32
15b34
15b35
15b36
15b37
15b38
15b40
Rel. Response %
Retention time [min]
Method 3
control
t-ES-Phe (15)
15b8
15b9
15b20
Rel. Response %
Retention time [min]
Method 4
control
t-ES-Phe (15)
15b1
15b2
Rel. Response %
Retention time [min]

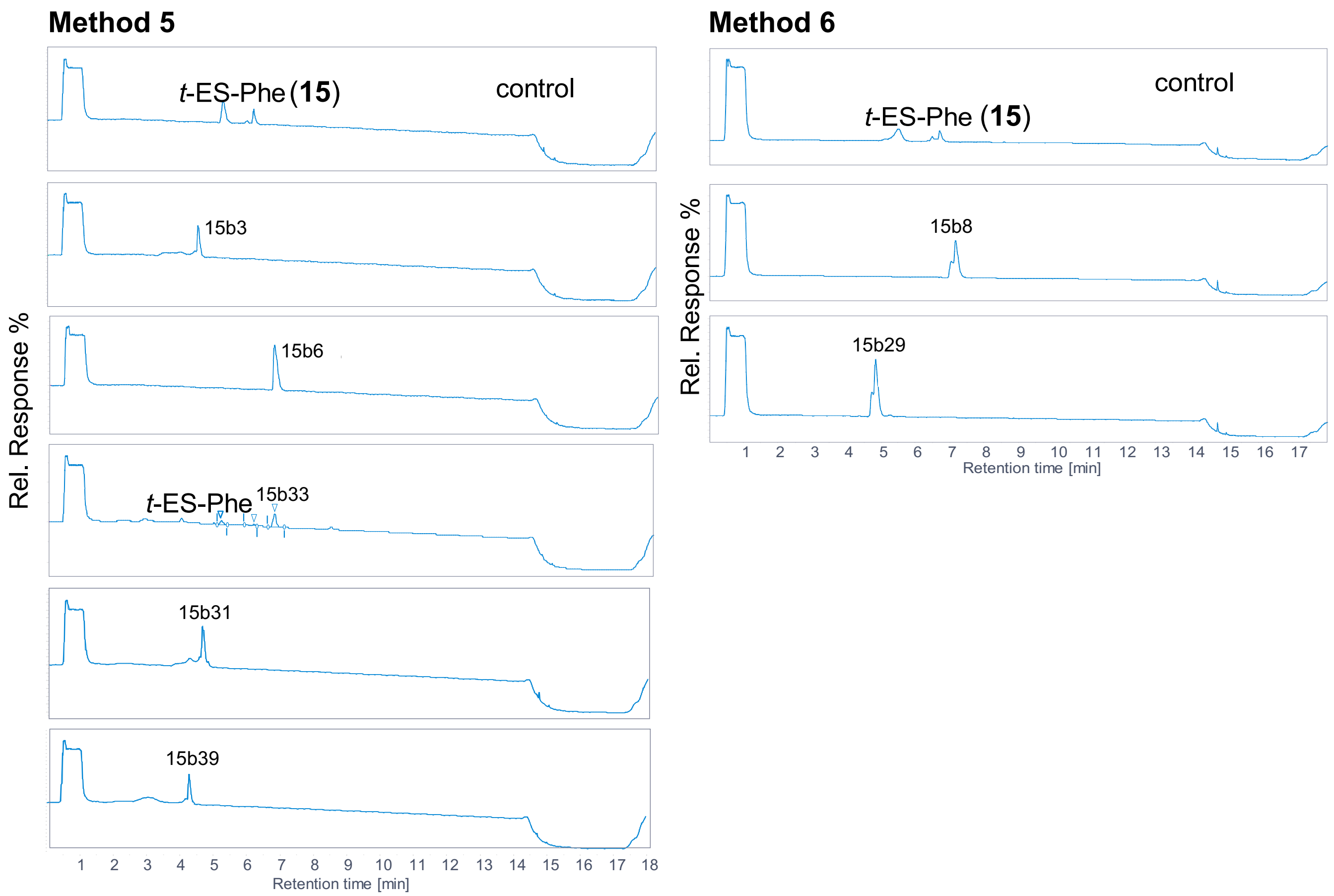

Supplementary Fig. 24 | HPLC traces from the in vitro reaction of Cp1D with (2*S*,3*S*)-*t*-ES-L-Phe (15) and different amines. Assays were carried out with 25 μM enzyme of Cp1D, 2 mM (2*S*,3*S*)-***t*-ES**-L-Phe (**15**), 5 mM amine, 10 mM ATP, and 10 mM $MgCl_2$ in 100 μL of 50 mM sodium phosphate buffer (pH 8.0) at 30 °C for 16 h. The traces were recorded at λ 204 nm. Cp1D was found to accept a variety of primary amines. The LC/MS analysis using Agilent LC/MSD iQ (AgilentTM InfinityLab Poroshell 120 Aq-C18, 2.7 μm, 100 Å, 2.1 × 100 mm) with elution gradients method, **Method 1**: 0-0.25 min, 1% eluent B; 0.25-8.5 min, 1-99% eluent B; 8.5-11.5 min, 99% eluent B; 11.5-13.5 min, 1% eluent B. **Method 2**: 0-0.25 min, 1% eluent B; 0.25-13.0 min, 1-99% eluent B; 13.0-16.0 min, 99% eluent B; 16.0-18.0 min, 1% eluent B. **Method 3**: 0-0.25 min, 1% eluent B; 0.25-13.0 min, 1-80% eluent B; 13.0-16.0 min, 99% eluent B; 16.0-18.0 min, 1% eluent B. **Method 4**: 0-0.25 min, 1% eluent B; 0.25-13.0 min, 1-60% eluent B; 13.0-16.0 min, 99% eluent B; 16.0-18.0 min, 1% eluent B. **Method 5**: 0-0.25 min, 1% eluent B; 0.25-13.0 min, 1-40% eluent B; 13.0-16.0 min, 99% eluent B; 16.0-18.0 min, 1% eluent B. **Method 6**: 0-0.25 min, 1% eluent B; 0.25-13.0 min, 1-30% eluent B; 13.0-16.0 min, 99% eluent B; 16.0-18.0 min, 1% eluent B. The analytical % yields shown in Fig. 4 were estimated from the standard curves generated at λ=204 nm of purified **15-b15** (for **15-b1−b17**, **15-b28**, **15-b39** and **15-b40**), **15-b24** (for **15-b21−b23**), **15-b27** (for 15-b25−b26), or **15-b37** (for **15-b34−b36**).

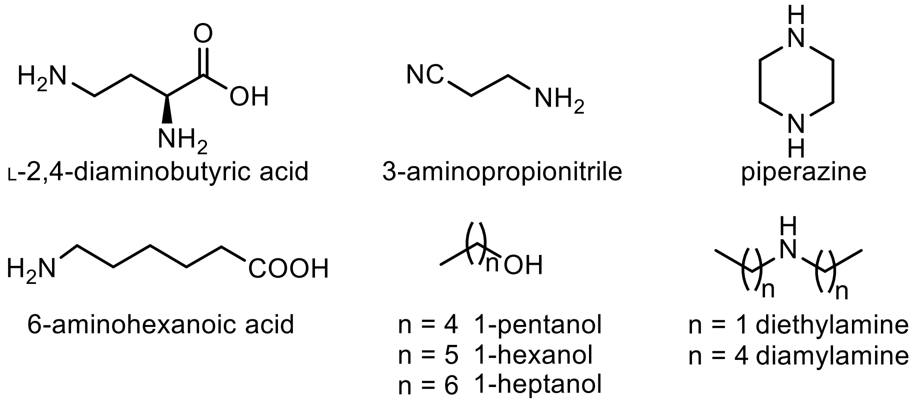

Supplementary Fig. 25 | Structures of amine nucleophiles which could not be accepted by Cp1D or Cp2D. Amines with negative charge functional groups were disfavored, such as carboxylic acid. On the other hand, amines with carboxylate ester can be used as amine nucleophiles. Secondary amines were also not preferred except for dimethylamine.

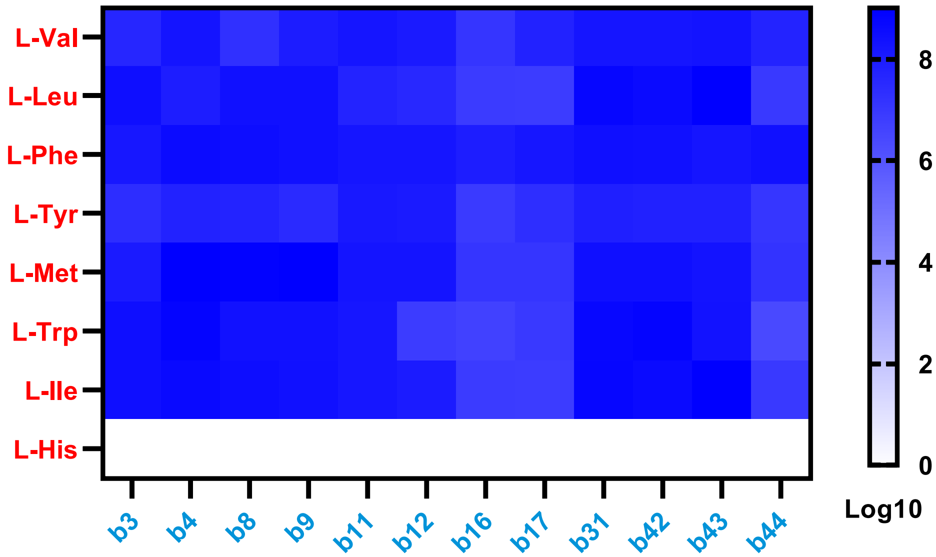

Supplementary Fig. 26 | LC/MS analysis of one-pot synthesis E-64 analogs by Cp1B and Cp1D in 96-well plate. The heat map for the corresponding products from ligation of proteinogenic amino acids (Y-axis: V, L, F, Y, M, W, I) with amine donors (X-axis: b3-4, b8-b9, b11-b12, b16-17, b31, b42 (spermidine), b43 (4-(2-aminoethyl)-pyridine), and b44 (tryptamine)) is shown as the heat map. Assays were carried out with enzymes 25 µM Cp1B and 25 µM Cp1D, 2 mM (±)-***t*-ES**, 1 mM L-amino acid, 1 mM amine, 10 mM ATP, and 10 mM $MgCl_2$ in 100 µL of 50 mM sodium phosphate buffer (pH 8.0) at 30 °C. The production of the corresponding products was determined by LC/MS analysis following 16 h incubation. Color scale is shown as ion counts of the corresponding products.

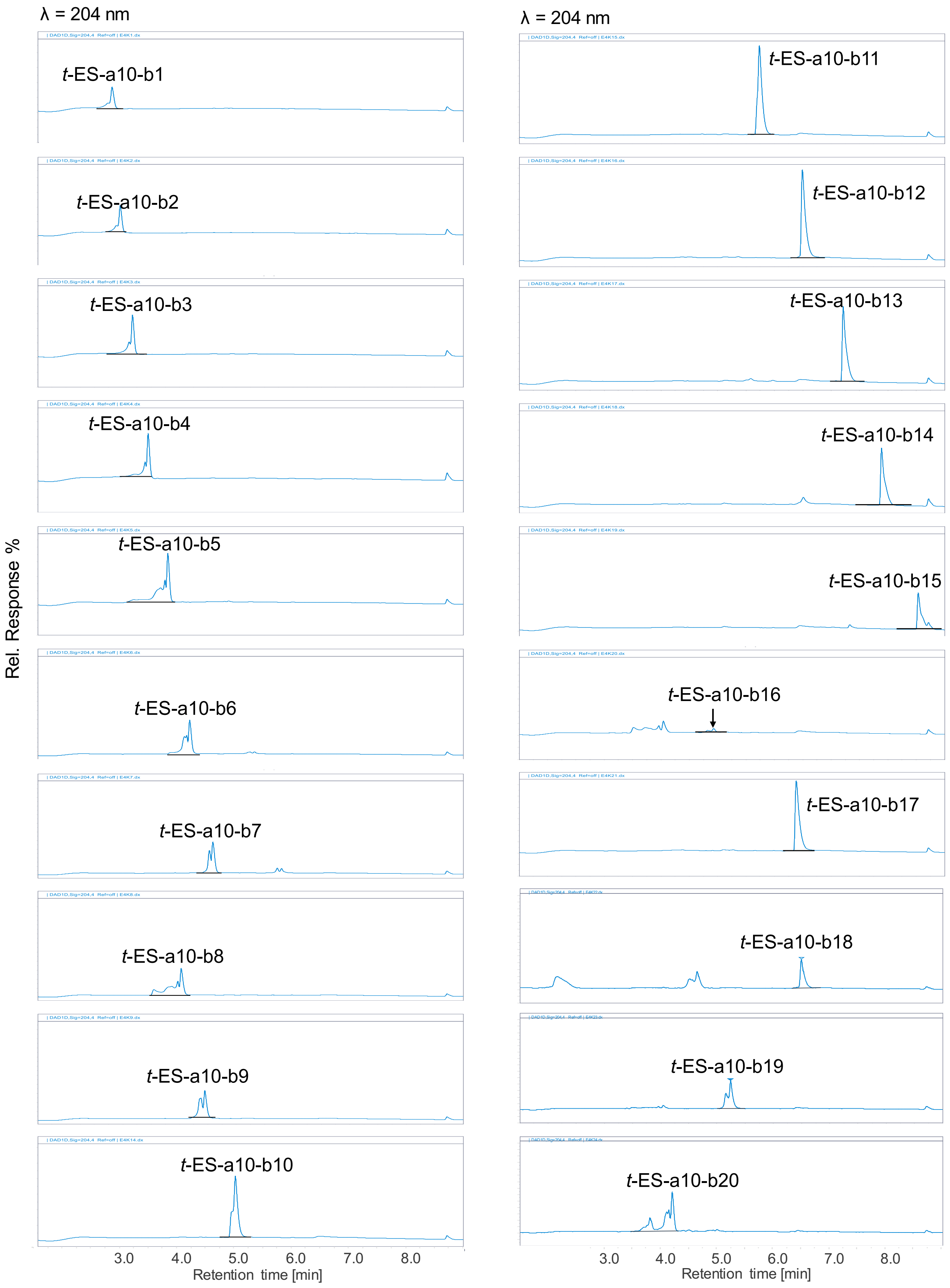

λ = 204 nm
t-ES-a10-b1
t-ES-a10-b2
t-ES-a10-b3
t-ES-a10-b4
t-ES-a10-b5
t-ES-a10-b6
t-ES-a10-b7
t-ES-a10-b8
t-ES-a10-b9
t-ES-a10-b10
Rel. Response %
3.0
4.0
5.0
6.0
7.0
8.0
Retention time [min]
λ = 204 nm
t-ES-a10-b11
t-ES-a10-b12
t-ES-a10-b13
t-ES-a10-b14
t-ES-a10-b15
t-ES-a10-b16
t-ES-a10-b17
t-ES-a10-b18
t-ES-a10-b19
t-ES-a10-b20
3.0
4.0
5.0
6.0
7.0
8.0
Retention time [min]

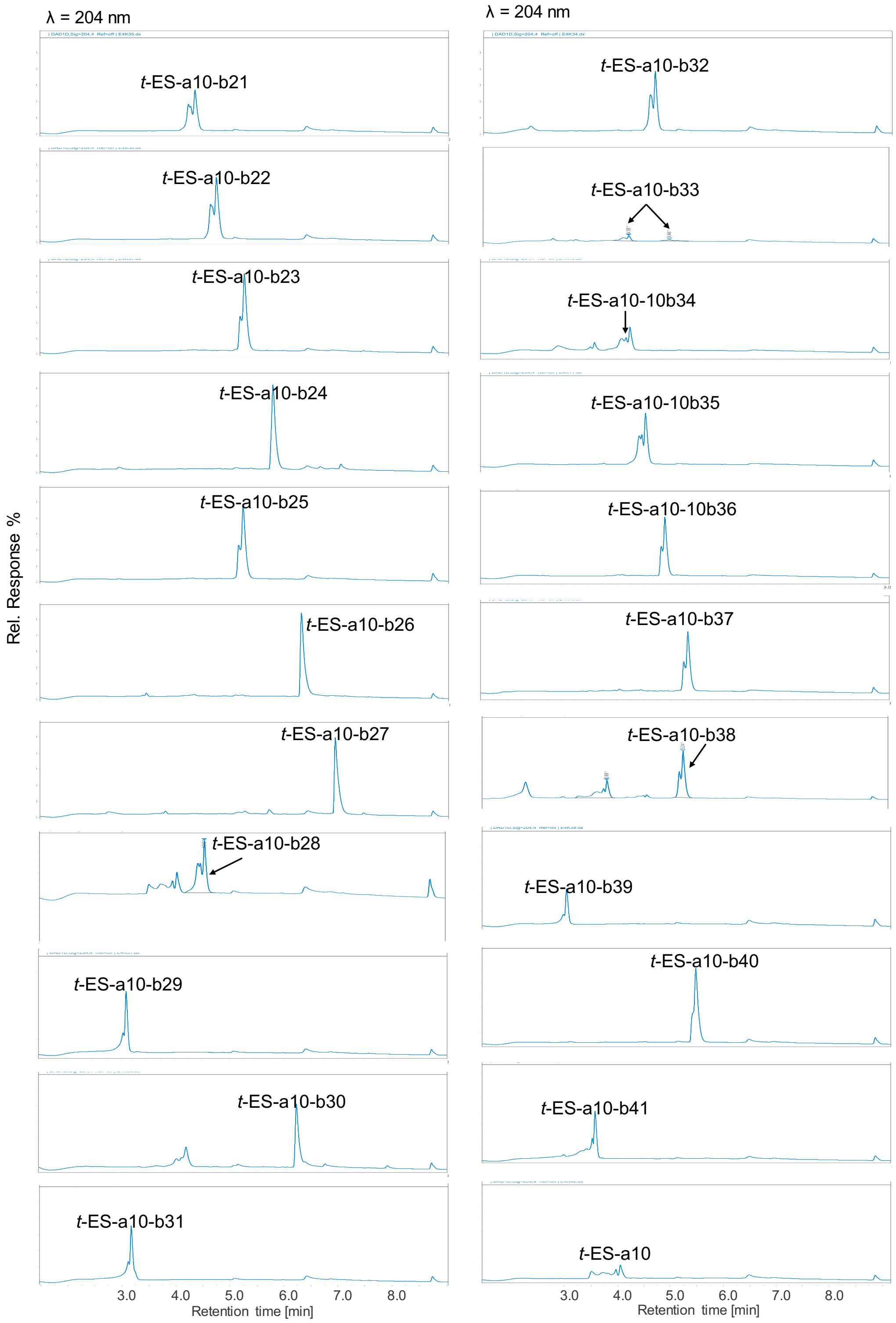

Supplementary Fig. 27 | HPLC traces of combinatorial biocatalysis with a10 and different amines. Assays were carried out with enzymes 25 µM Cp1B and 25 µM Cp1D, 2 mM (±)-***t*-ES**, 1 mM **a10**, 1 mM amine, 10 mM ATP, 10 mM $MgCl_2$ in 100 µL of 50 mM sodium phosphate buffer (pH 8.0) at 30 ºC for 16 hours. The LC/MS elution gradients method: 0-0.25 min, 1% eluent B; 0.25-13.0 min, 1-99% eluent B; 13.0-16.0 min, 99% eluent B;

16.0-18.0 min, 1% eluent B using Agilent LC/MSD iQ (AgilentTM InfinityLab Poroshell 120 Aq-C18, 2.7 µm, 100 Å, 2.1 × 100 mm).

a) E-64 synthesis

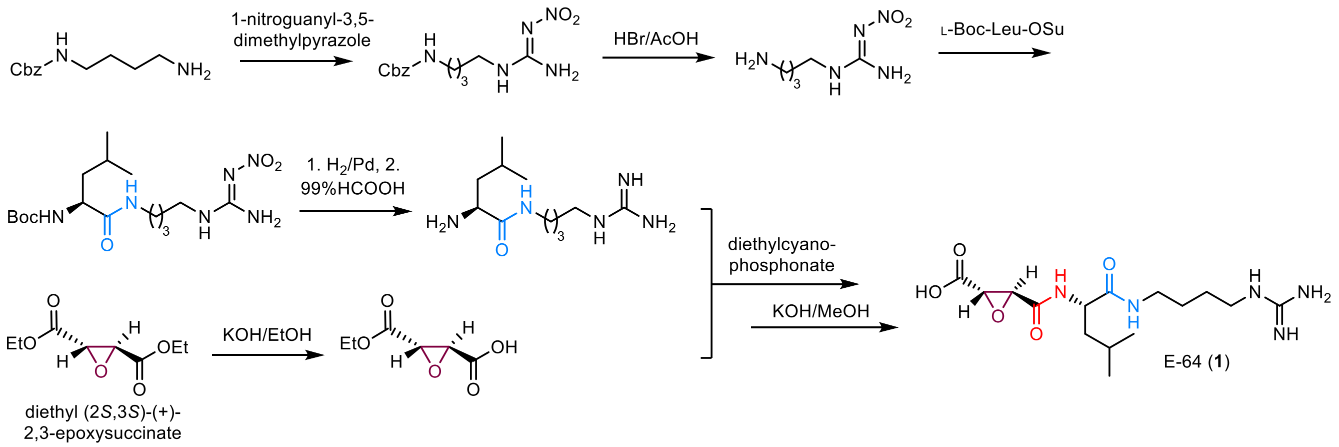

b) CLIK-148 synthesis

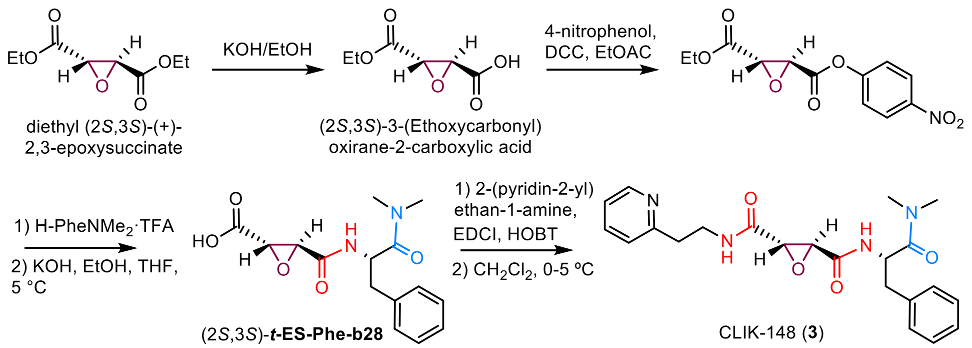

Supplementary Fig. 28 | Reported chemical synthesis for E-64[34] and CLIK-148[8]. a. General scheme for chemical synthesis of E-64. **b**. Recently reported synthetic scheme for CLIK-148. Both synthetic schemes require the preparation of diethyl (2*S*,3*S*)-(+)-2,3-epoxysuccinate and multiple synthetic steps including protection-deprotection steps.

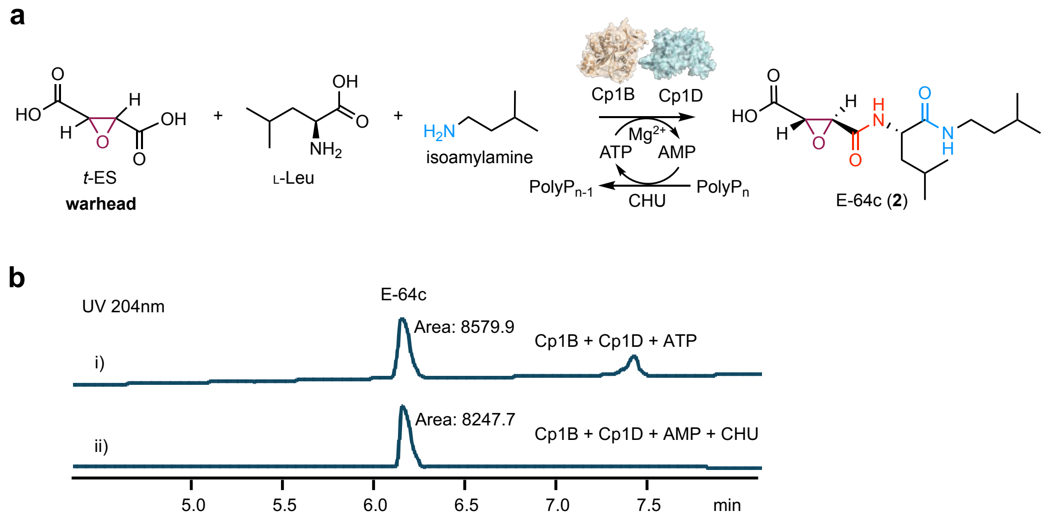

Supplementary Fig. 29. Preparative-scale synthesis of E-64c using ATP regeneration system. **a**, Scheme for E-64c (**2**) from (±)-*t*-ES, L-Leu, and isoamylamine (isopentylamine) catalyzed by Cp1B and Cp1D, with recycling the ATP using the Polyphosphate kinase (CHU)[35] and polyphosphate ($PolyP_n$). **b**, Preparative-scale synthesis of E-64c with addition of 10 mM ATP (i) or using ATP Regeneration system (ii). Reaction conditions for i): 5 mM (±)-***t*-ES**, 2.5 mM L-Leu, 5 mM isoamylamine, 10 mM ATP, 10 mM $MgCl_2$, 2.5 μM Cp1B, and 2.5 μM Cp1D in 20 mL of 50 mM sodium phosphate buffer (pH 8.0). Reaction conditions for ii): 5 mM (±)-***t*-ES**, 2.5 mM L-Leu, 5 mM isoamylamine, 10 mM AMP, 10 mM $MgCl_2$, 20 mg mL^{-1} $PolyP_n$, 2.5 μM Cp1B, 2.5 μM Cp1D, and 50 μM CHU in 20 mL of 50 mM sodium phosphate buffer (pH 8.0). Incubation at 30°C for 48 h. A comparable production of E-64c formation was observed.

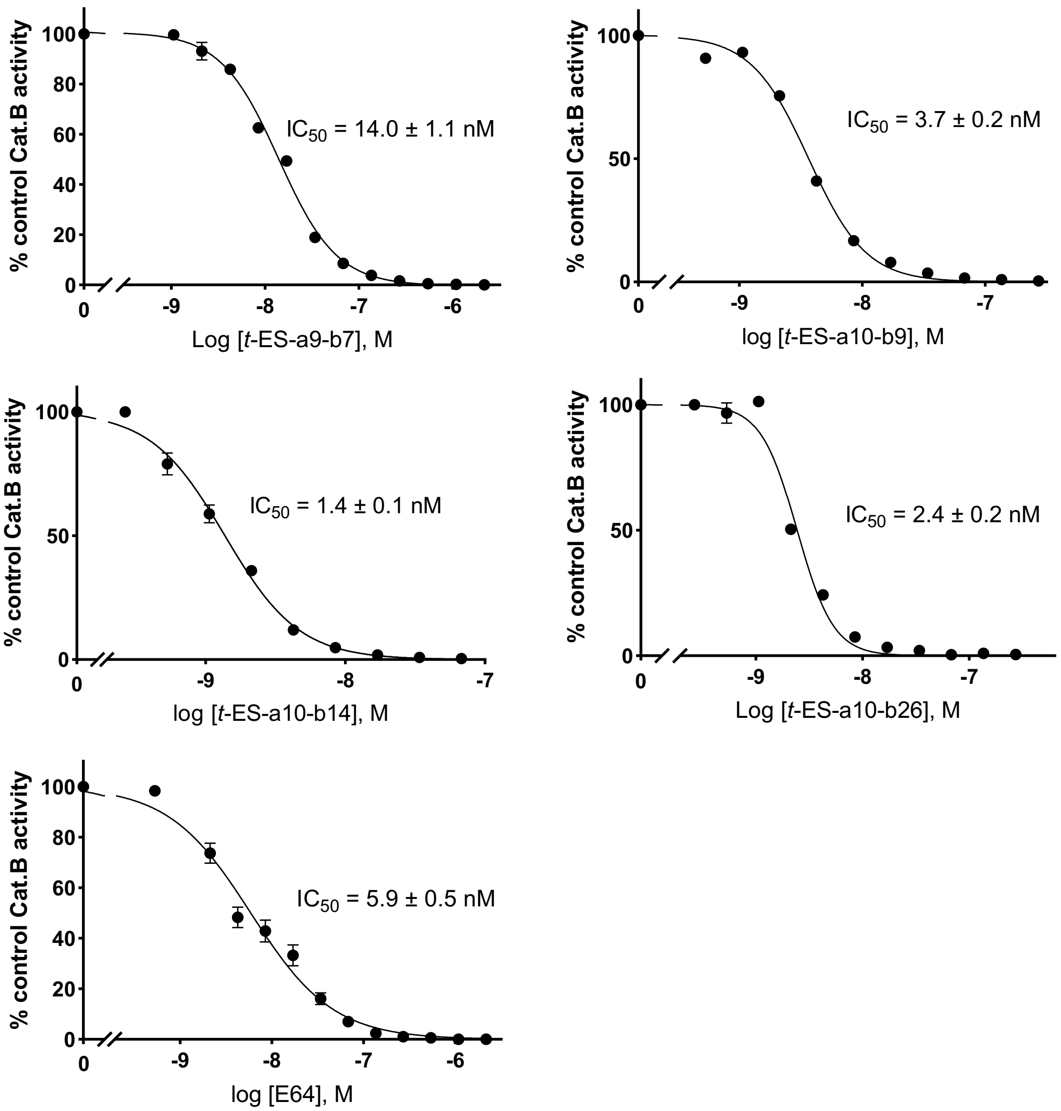

Supplementary Fig. 30 | In vitro cathepsin B inhibition assay of enzymatically synthesized inhibitors. The values represent means ± s.d., and error bars indicate s.d. of three independent replicates (n = 3).

a

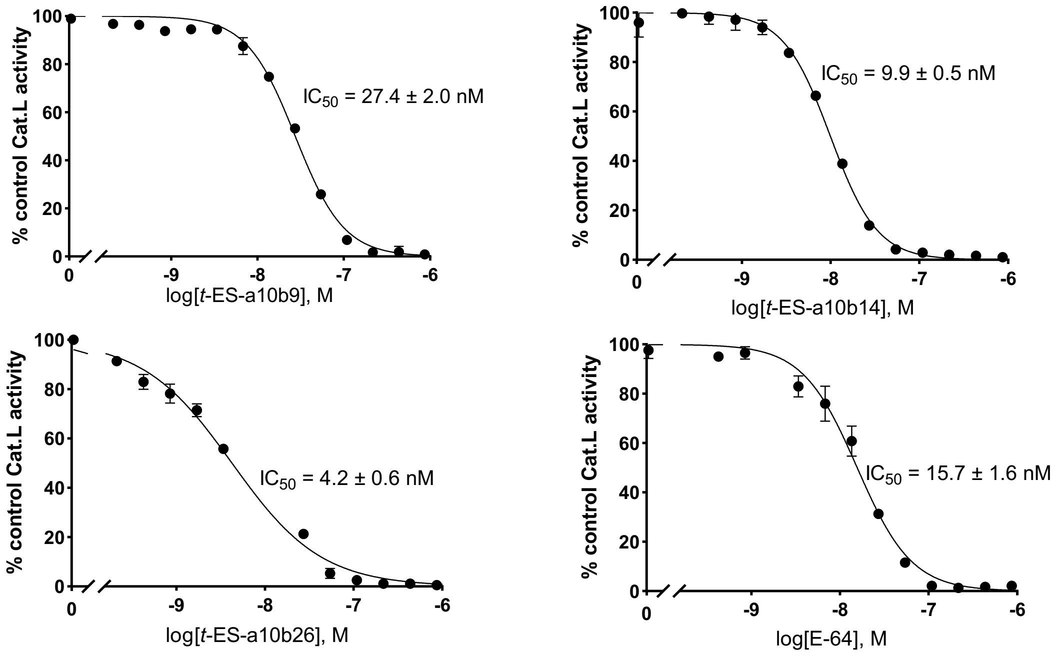

b

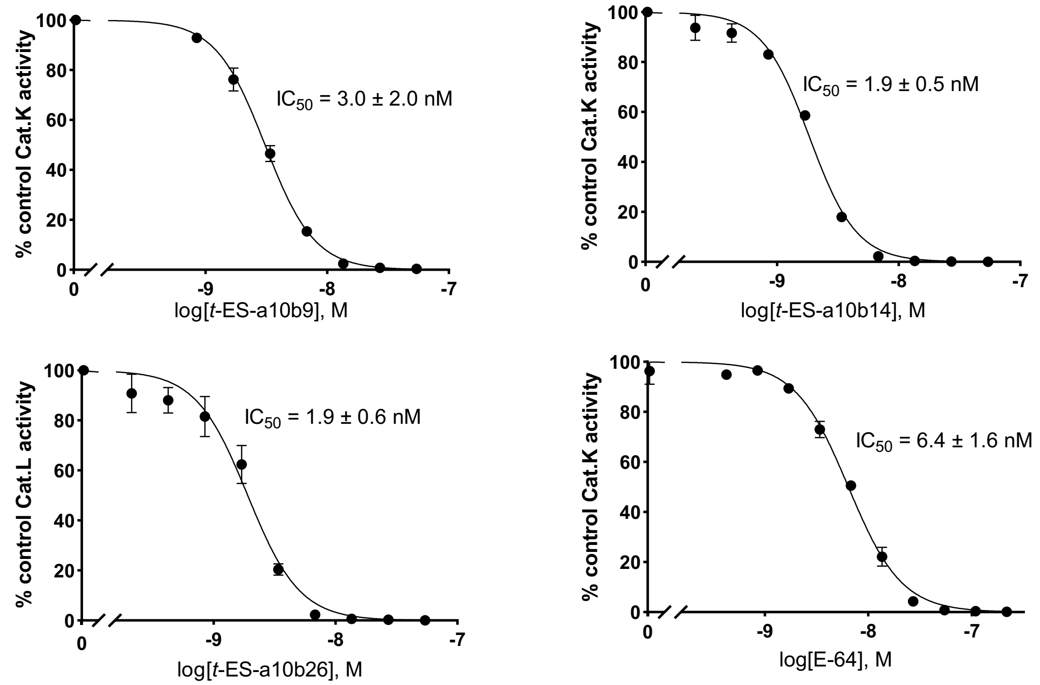

Supplementary Fig. 31 | Inhibitory activity toward other cathepsin proteases. a. Inhibitory activity of the compounds toward cathepsin L. **b**. Inhibitory activity of the compounds toward cathepsin K. The values represent means ± s.d., and error bars indicate s.d. of three independent replicates (n = 3).

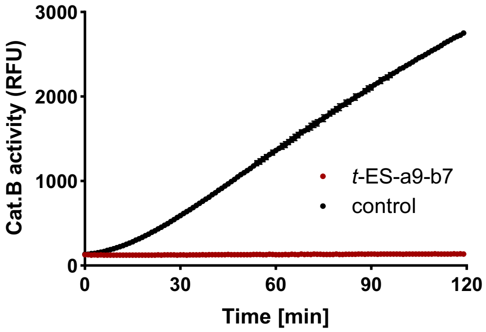

Supplementary Fig. 32 | Irreversible inhibition of cathepsin B by (2*S*,3*S*)-*t*-ES-a9-b7. Inhibition mode of (2*S*,3*S*)-***t*-ES-a9-b7** toward cathepsin B (irreversible or reversible inhibition) was assessed by the standard dilution experiments. The lack of cathepsin B activity after dilution agrees with the irreversible inhibitory mechanism. Briefly, mature cathepsin B was incubated with ***t*-ES-a9-b7** at 10 times the IC_{50} concentration (corresponding to 140 nM) for 30 min. Then, the mixture was diluted to 1/10 the IC_{50} concentration by activation buffer (20 mM Na-acetate pH 5.5, 1 mM EDTA, 5 mM DTT, and 100 mM NaCl). The diluted sample was assayed with 40 μM *Z*-Phe-Arg-AMC in reaction buffer (40 mM citrate phosphate pH 5.5, 1 mM EDTA, 100 mM NaCl, 5 mM DTT, and 0.01% Brij). Cleavage of *Z*-Phe-Arg-AMC to generate fluorescent AMC was monitored at relative fluorescence unit (RFU) (excitation 360 nm, emission 460 nm) were recorded over a period of 30 min in infinite M200 PRO multimode microplate reader (Tecan) at 37 °C. The lack of cathepsin B activity after dilution demonstrates the irreversible inhibitory mechanism of ***t*-ES-a9-b7**. Control: Cathepsin B was incubated without an inhibitor and displayed the formation of AMC in the assay.

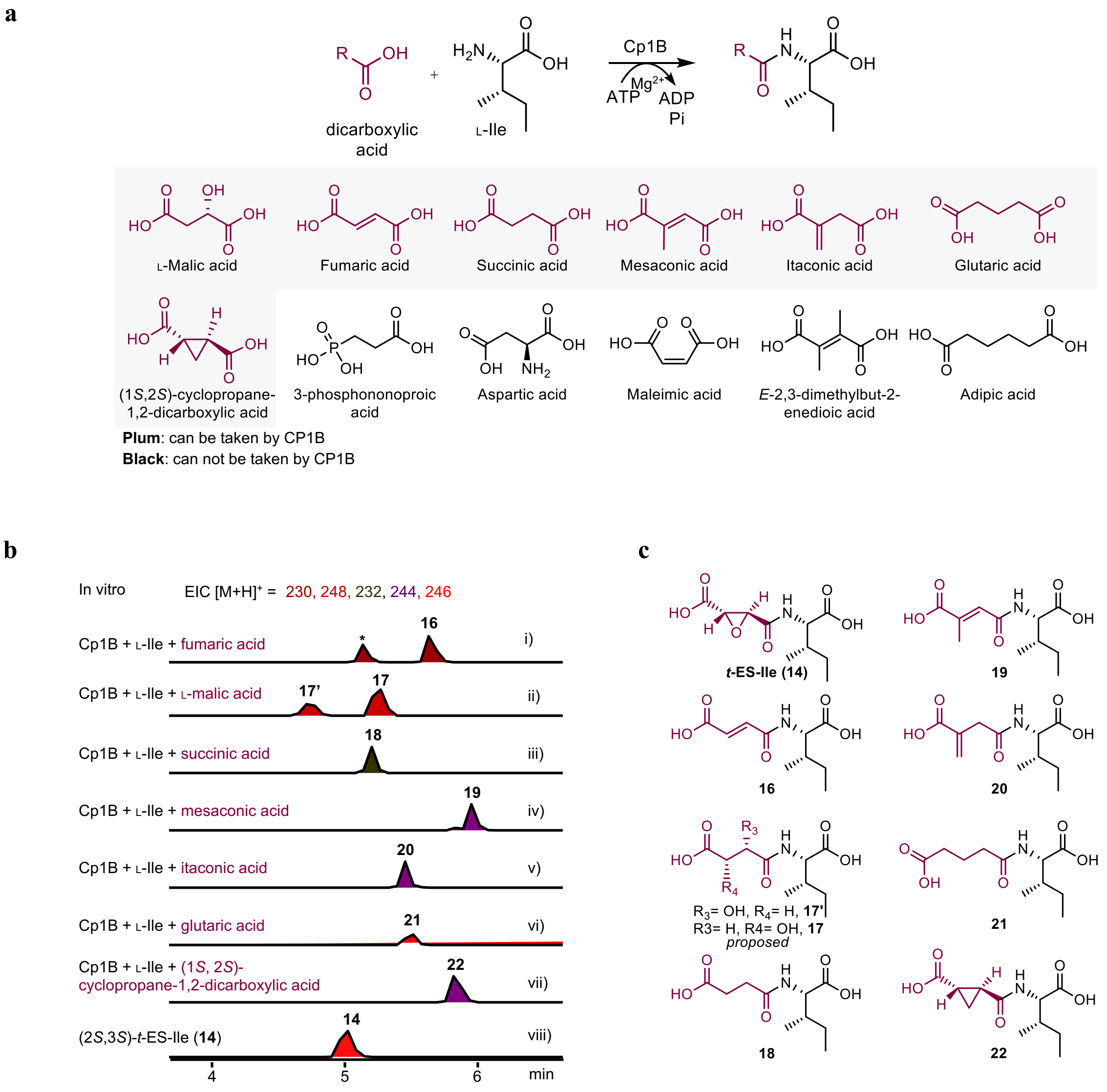

Supplementary Fig. 33 | Dicarboxylic acid scope for Cp1B. a. Structure of dicarboxylic acids tested with Cp1B. Note: dicarboxylic acids which can be taken by Cp1B are highlighted in plum while dicarboxylic acids which cannot be taken by Cp1B are colored in black. **b**. LC/QTOF analysis of the predicted product from the ligation of various dicarboxylic acids with L-Ile. The products were detected when L-malic acid, succinic acid, fumaric acid, mesaconic acid, itaconic acid, (1*S*,2*S*)-*trans*-cyclopropane-1,2-dicarboxylic acid, and glutaric acid were used as the acid acceptor. *the parent MS is different with **16**. Y-axis represents ion counts and the chromatograms are presented on the same scale. **c**. Predicted structures of corresponding products. For *in vitro* assay of Cp1B, assays were carried out in 100 µL of 50 mM sodium phosphate buffer (pH 8.0), containing 25 µM Cp1B, 5 mM *trans*-epoxy-succinic acid or 2.5 mM analog, 2.5 mM amino acid, 10 mM ATP, 10 mM $MgCl_2$ at 30 °C for 16 h. The reaction was then quenched with 120 µL MeCN and centrifuged at 17,000 x *g* for 5 min. The supernatant was subjected to LC/QTOF analysis.

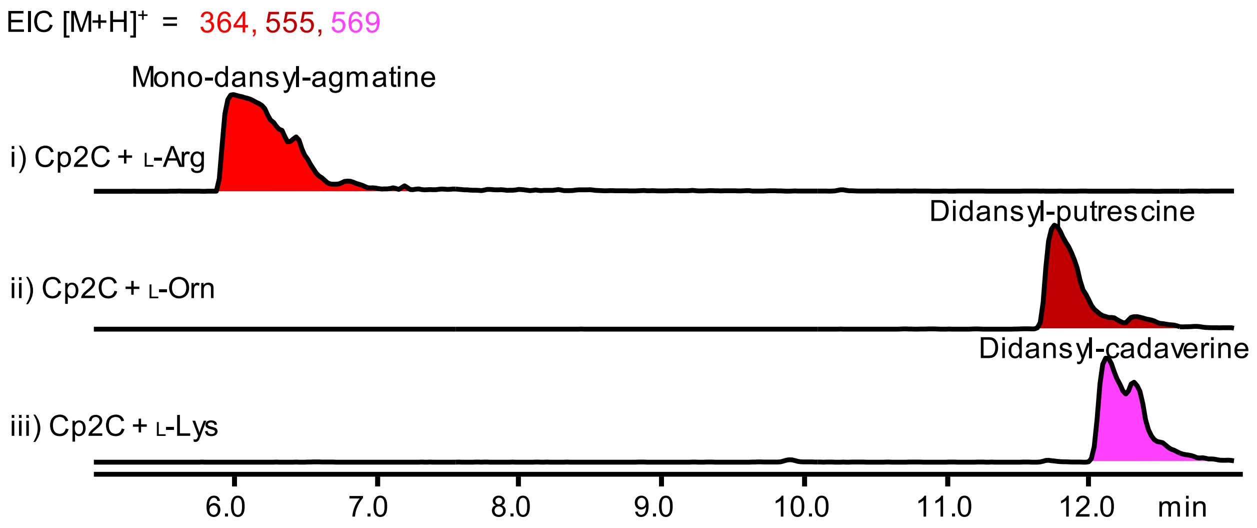

Supplementary Fig. 34 | In vitro assay of Cp2C with L-Ornithine, L-Arginine and L-Lysine. The formation of corresponding decarboxylated product agmatine (mono-dansyl-agmatine, $[M+H]^+$ = 364), putrescine (didansyl-putrescine, $[M+H]^+$ = 555), and cadaverine (didansyl-cadaverine, $[M+H]^+$ = 569) was observed. *In vitro* assays of Cp2C were performed in 50 µL of 50 mM sodium phosphate buffer (pH 8.0), containing 50 µM of Cp2C, 100 µM PLP, 2 mM L-amino acid such as L-ornithine, L-lysine, and L-arginine at 30 °C. After 1 hours at 30 °C, all reactions were quenched with 50 µL MeCN, centrifuged at 17,000 x *g* for 5 min, which was subjected to LC-MS analysis after dansyl derivatization with dansyl chloride (TCI). As for dansyl derivatization to detect decarboxylated product, 1 M borate buffer (pH 8.0) and dansyl chloride solution (10 mM final concentration) were added to the mixture. After incubating at 30 °C for 1 hour, the resultant reaction mixture was centrifuged at 17,000 x *g* for 5 min. The resultant supernatant was subjected to QTOF analysis.

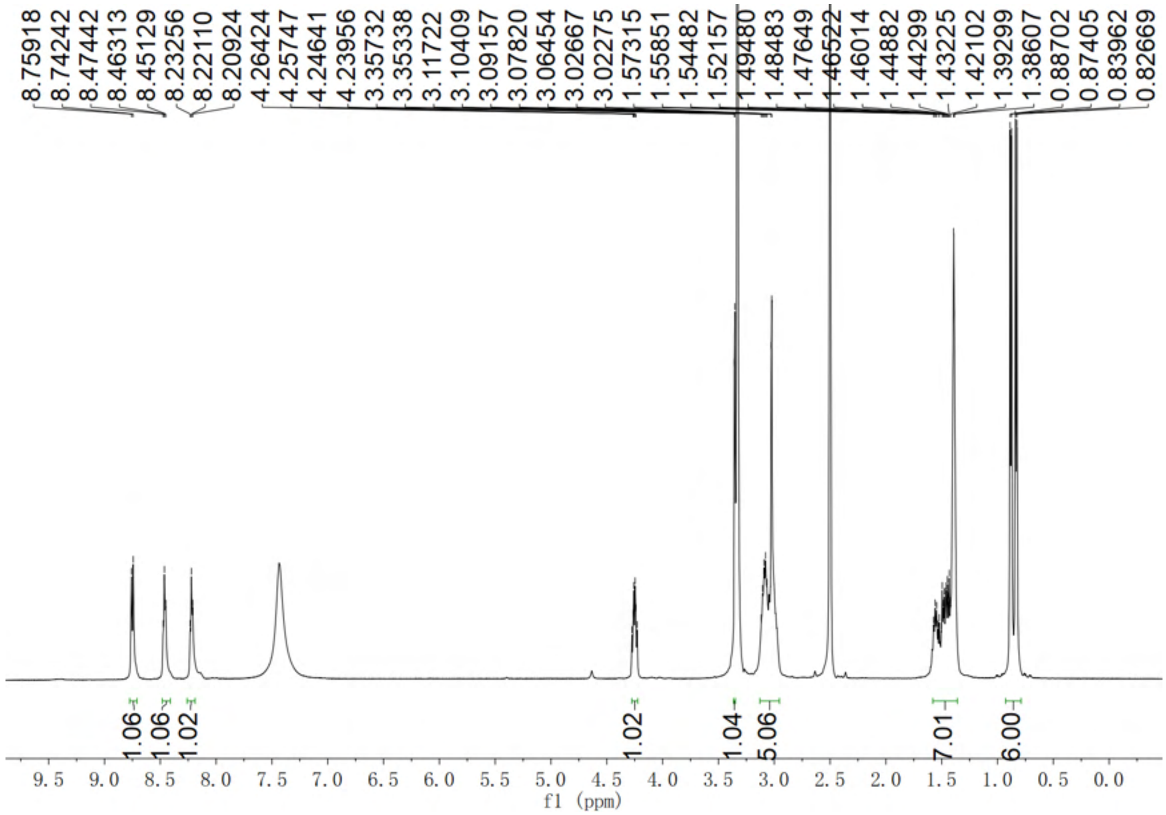

Supplementary Fig. 35. ^{1}H NMR spectrum of E-64 (1) in DMSO-d_6

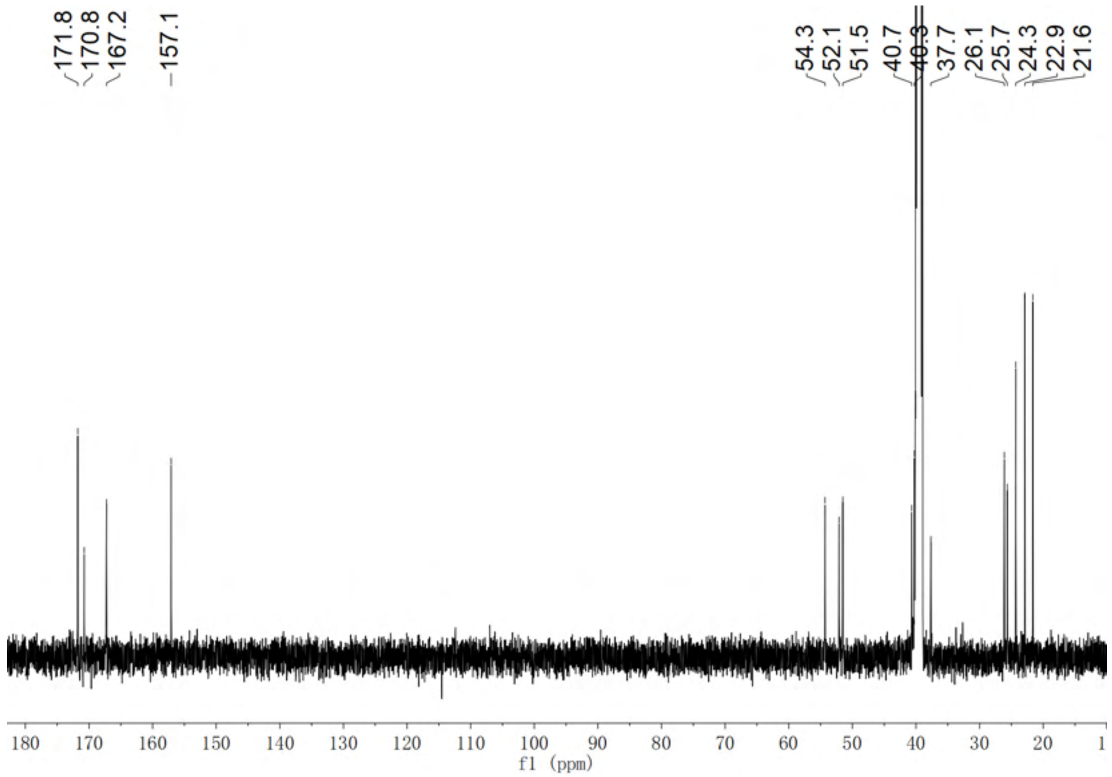

Supplementary Fig. 36. ^{13}C NMR spectrum of E-64 (1) in DMSO-d_6

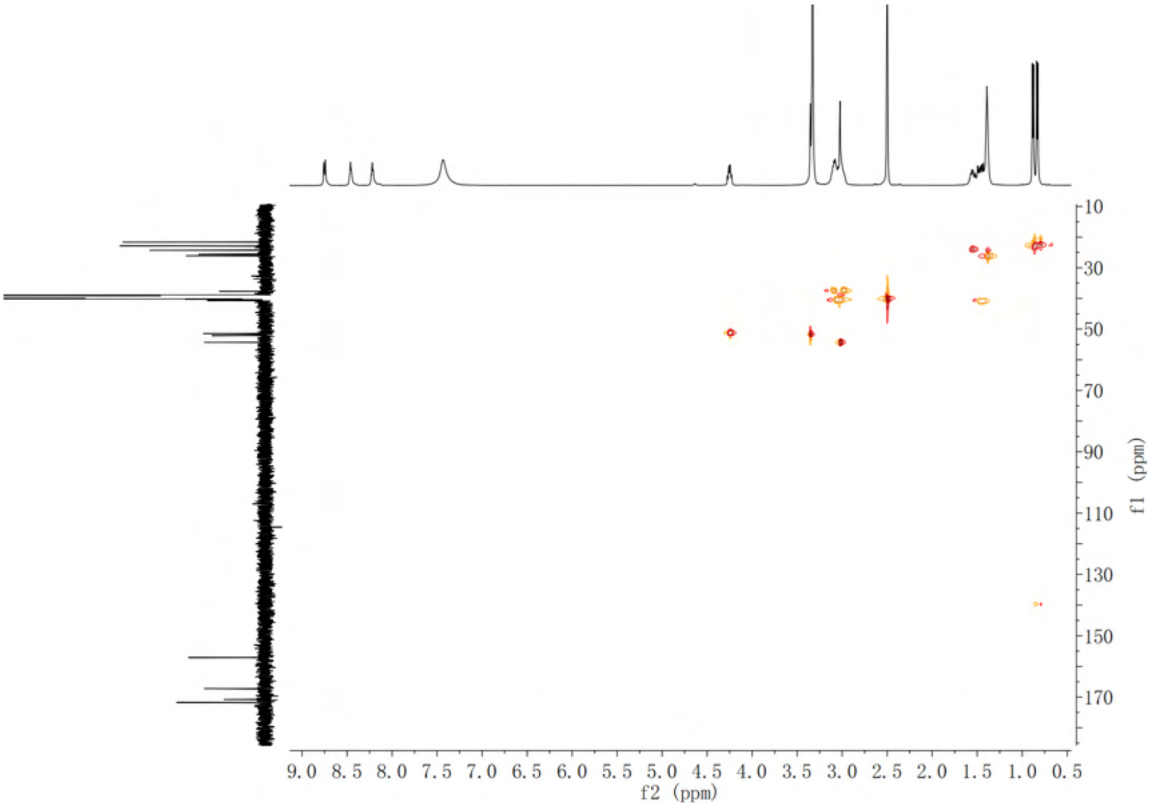

Supplementary Fig. 37. HSQC spectrum of E-64 (1) in DMSO-d_6

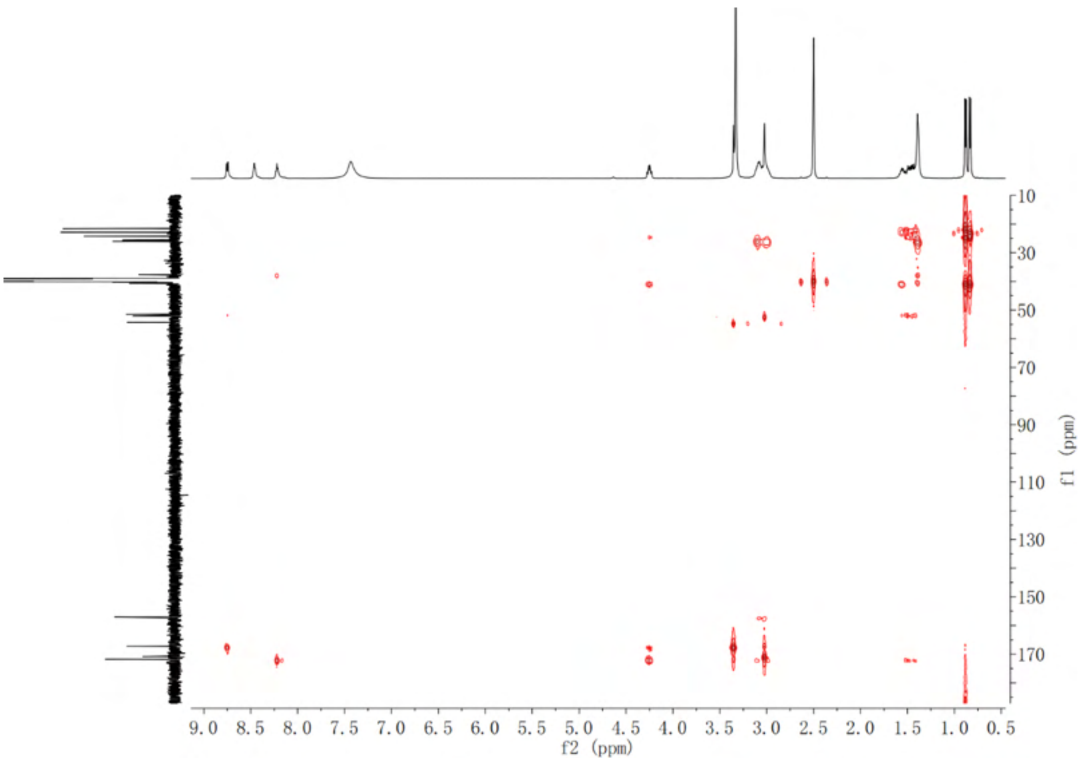

Supplementary Fig. 38. HMBC spectrum of E-64 (1) in DMSO-d_6

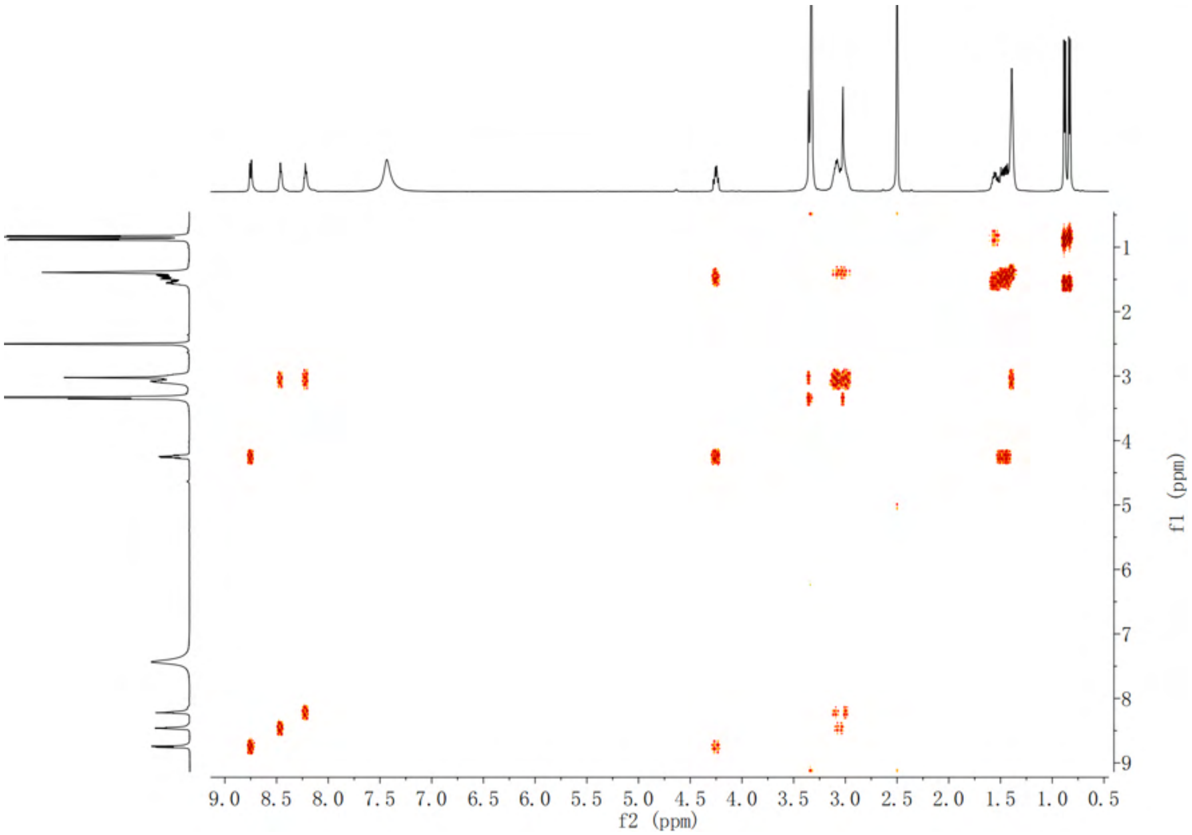

Supplementary Fig. 39. ^{1}**H-**^{1}**H COSY spectrum of E-64 (1) in DMSO-*****d***$_6$

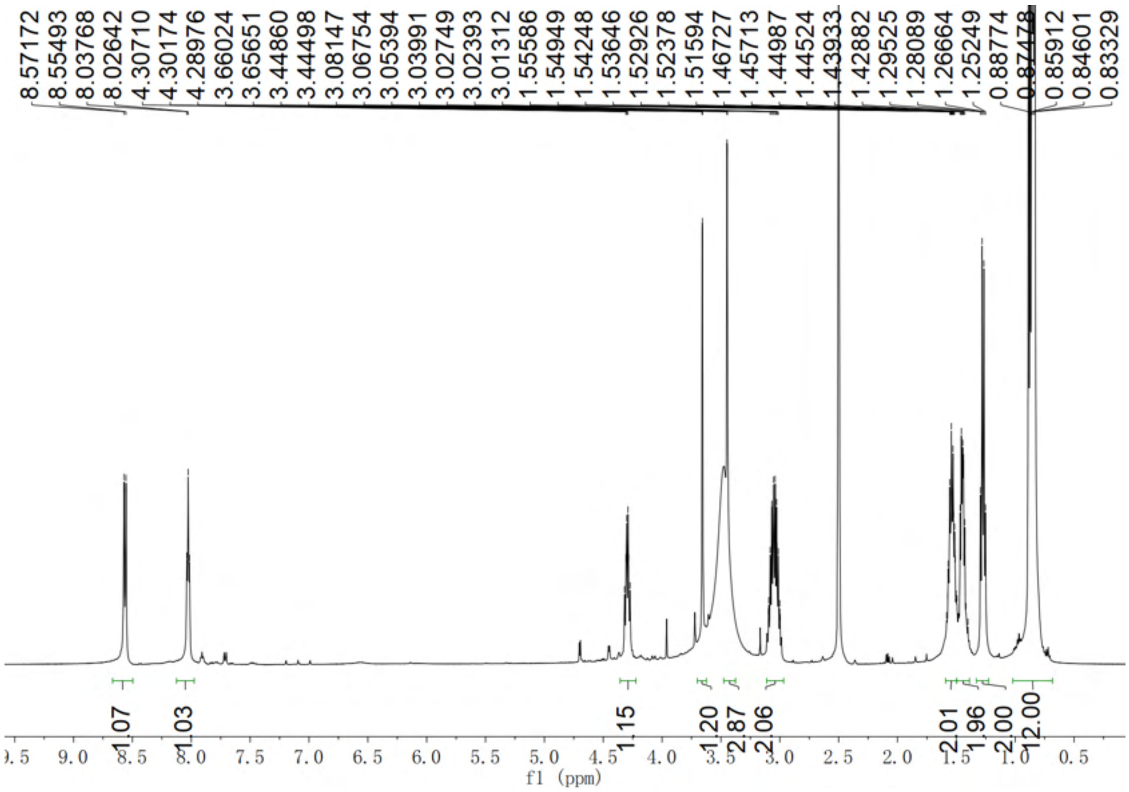

Supplementary Fig. 40. ^{1}**H NMR spectrum of E-64c (2) in DMSO-*****d***$_6$

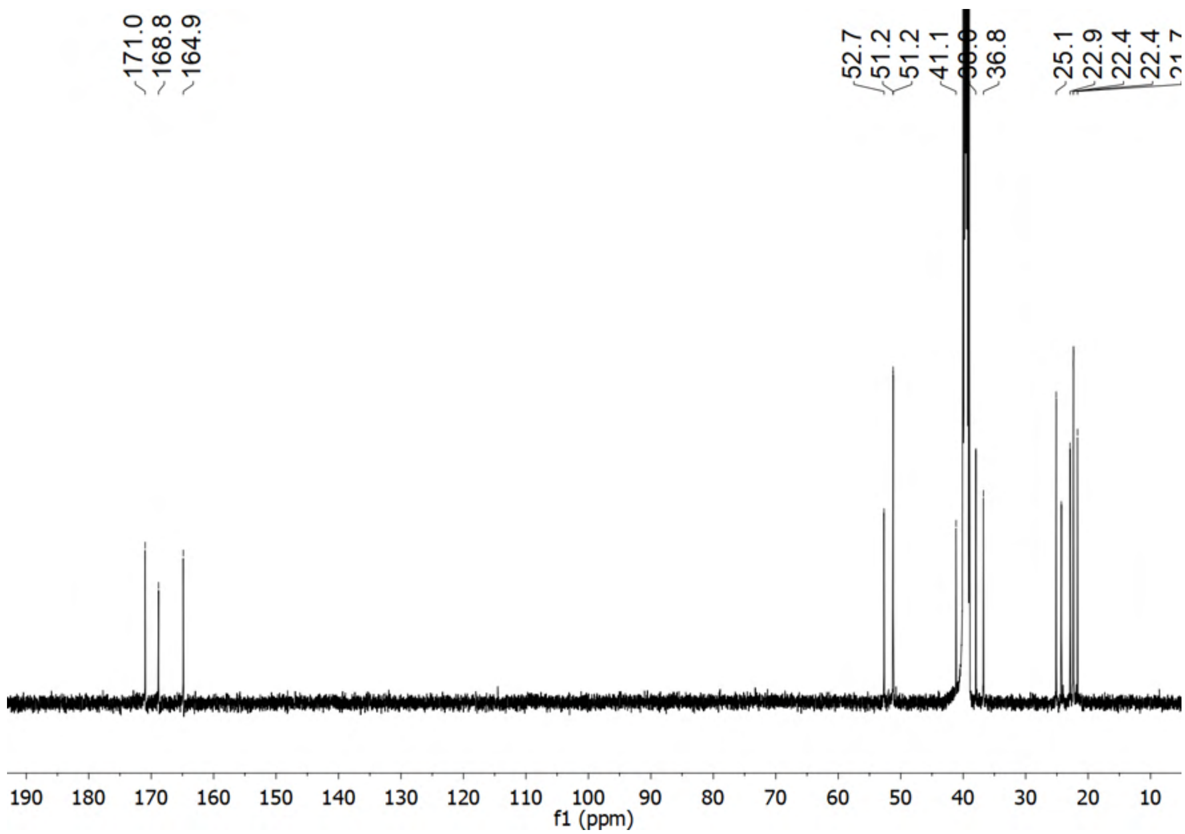

Supplementary Fig. 41. ^{13}C NMR spectrum of E-64c (2) in DMSO-d_6

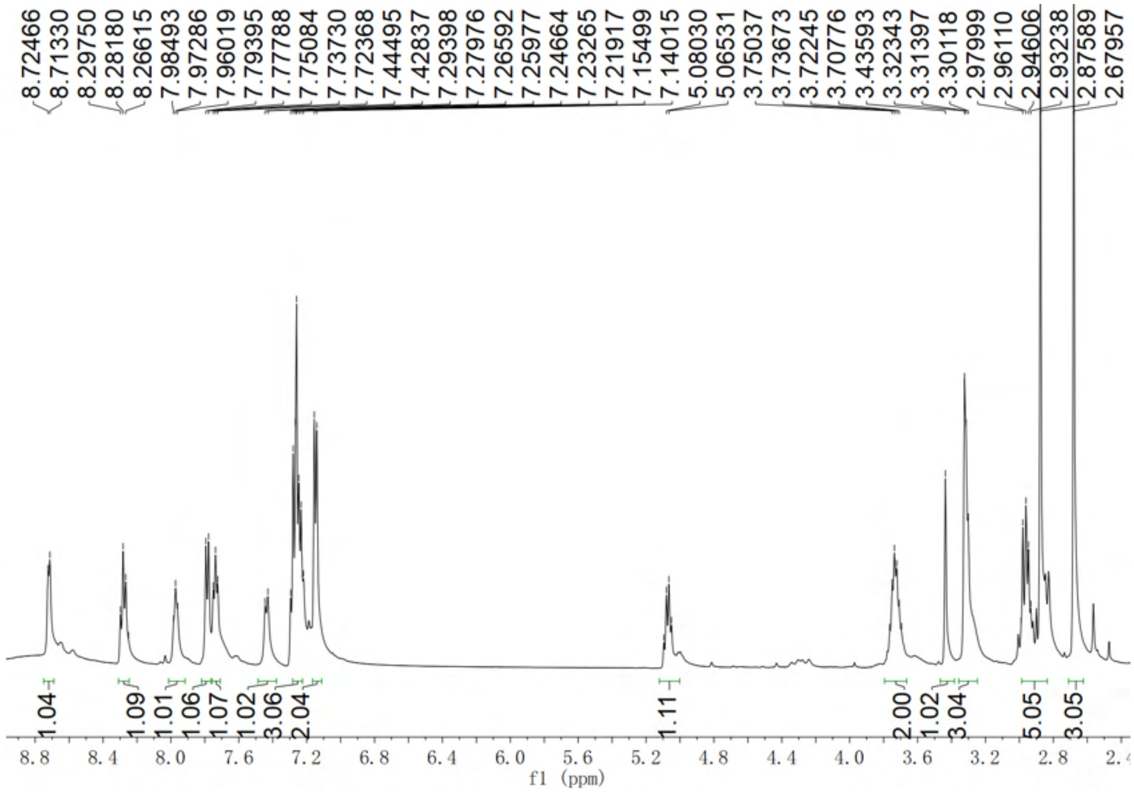

Supplementary Fig. 42. ^{1}H NMR spectrum of CLIK148 (3) in $CDCl_3$

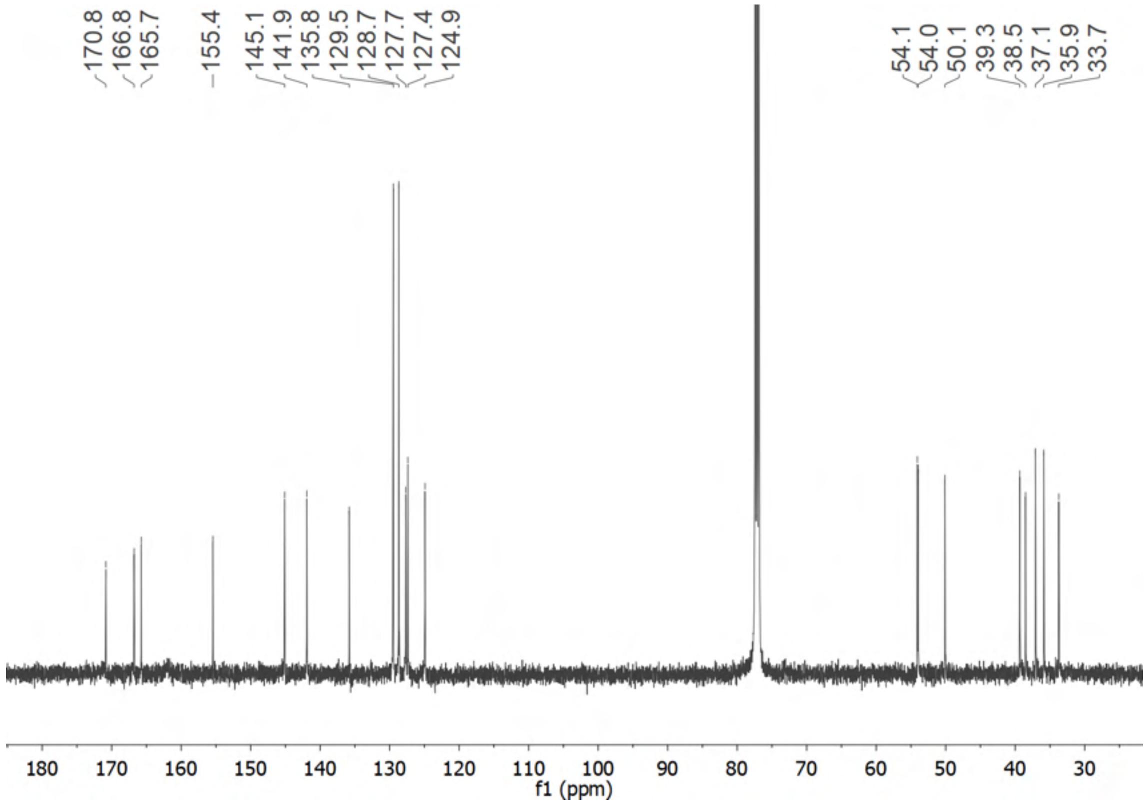

Supplementary Fig. 43. ^{13}**C NMR spectrum of CLIK148 (3) in CDCl**$_3$

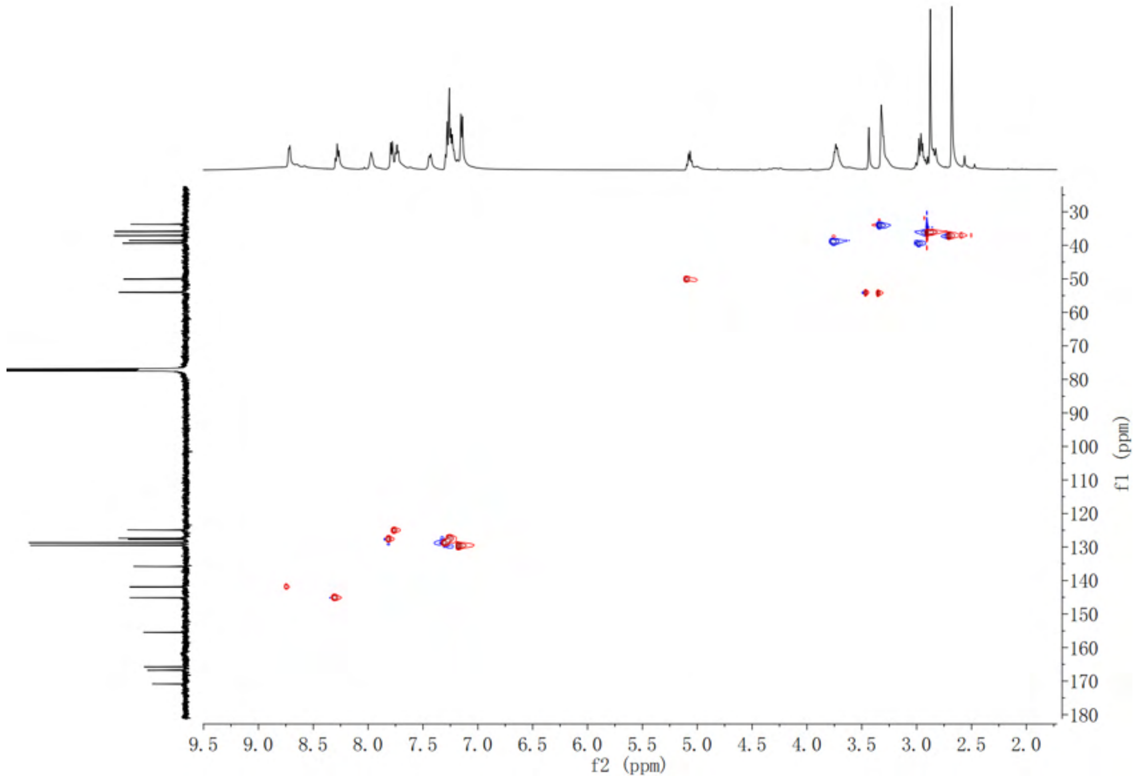

Supplementary Fig. 44. HSQC spectrum of CLIK148 (3) in CDCl$_3$

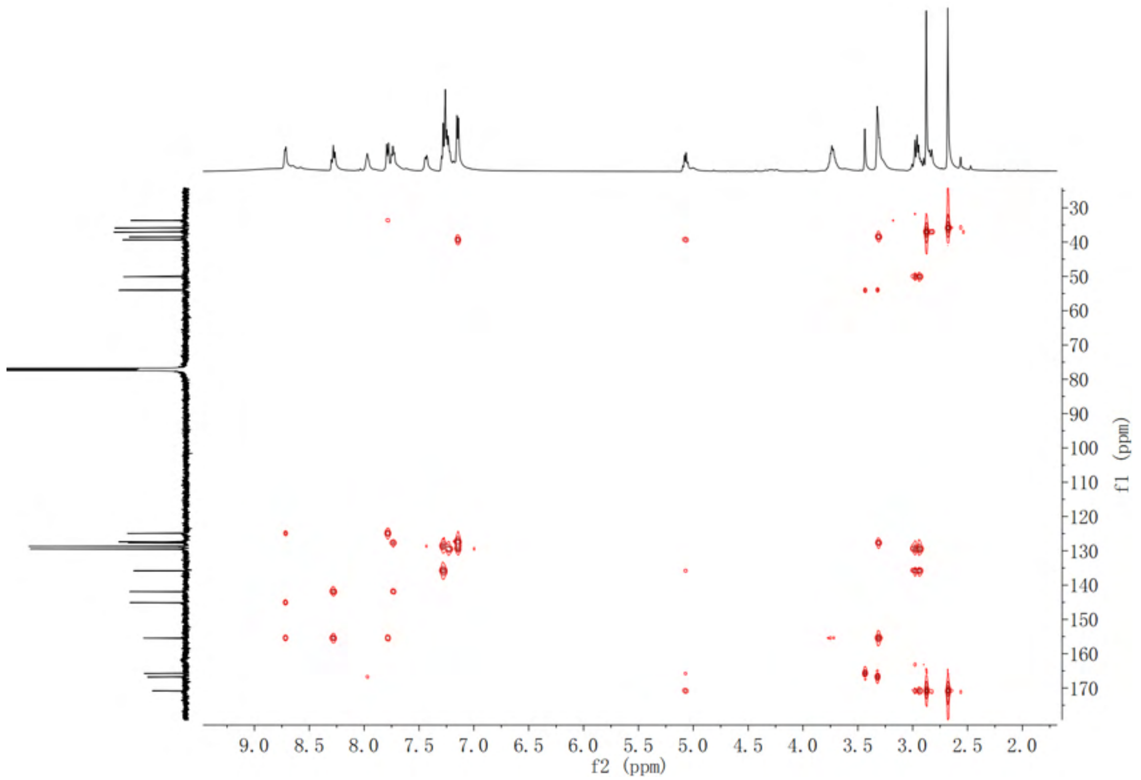

Supplementary Fig. 45. HMBC spectrum of CLIK148 (3) in $CDCl_3$

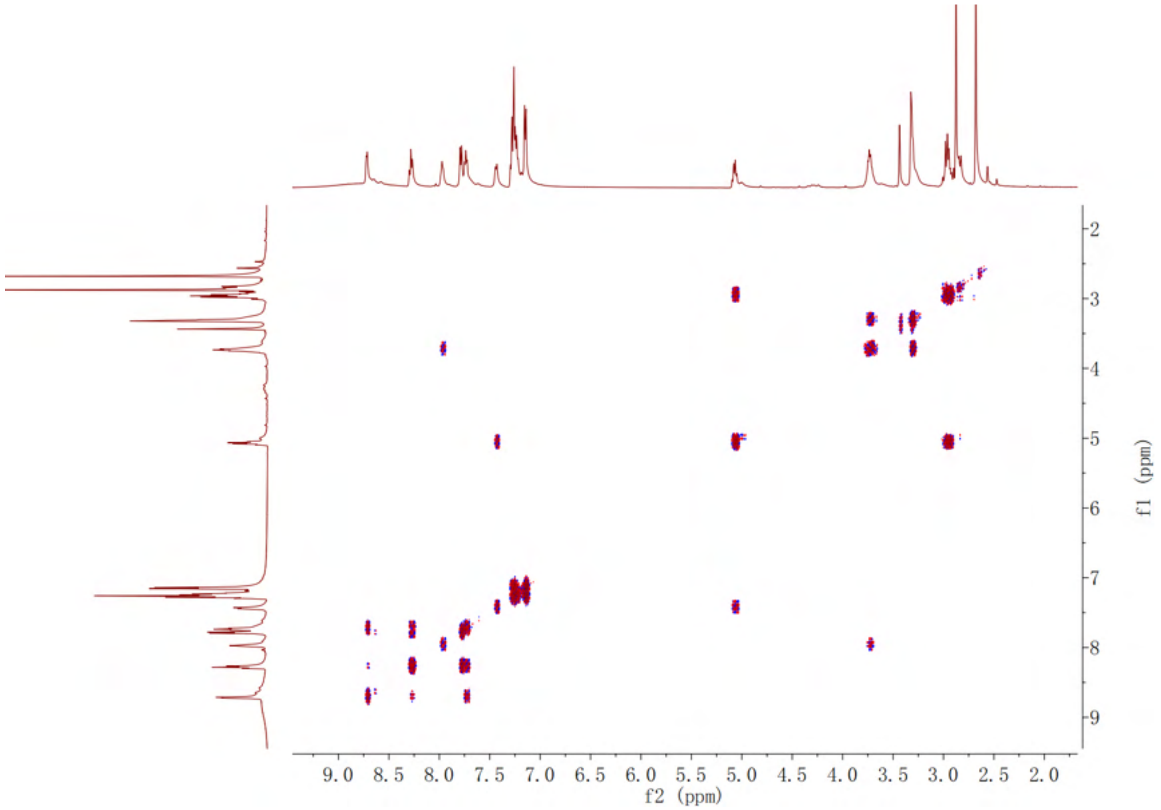

Supplementary Fig. 46. ^1H-^1H COSY spectrum of CLIK148 (3) in $CDCl_3$

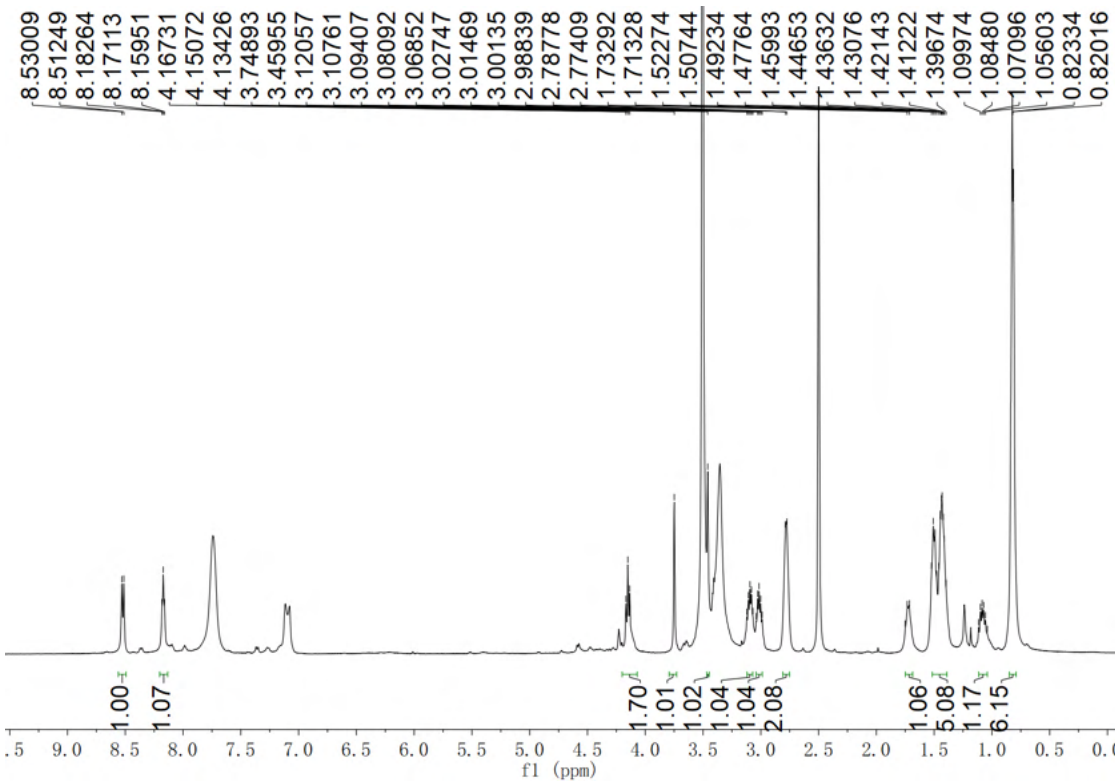

Supplementary Fig. 47. ^{1}H NMR spectrum of CPI-2 (4) in DMSO-d_6

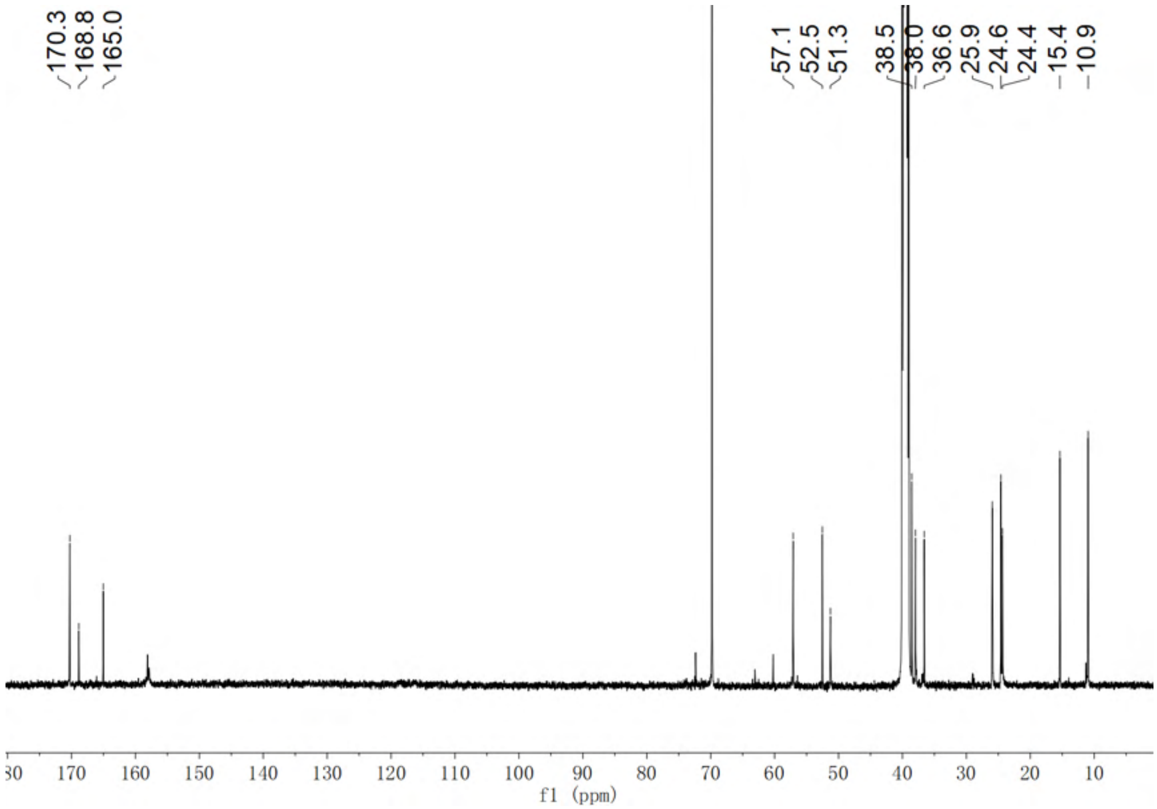

Supplementary Fig. 48. ^{13}C NMR spectrum of CPI-2 (4) in DMSO-d_6

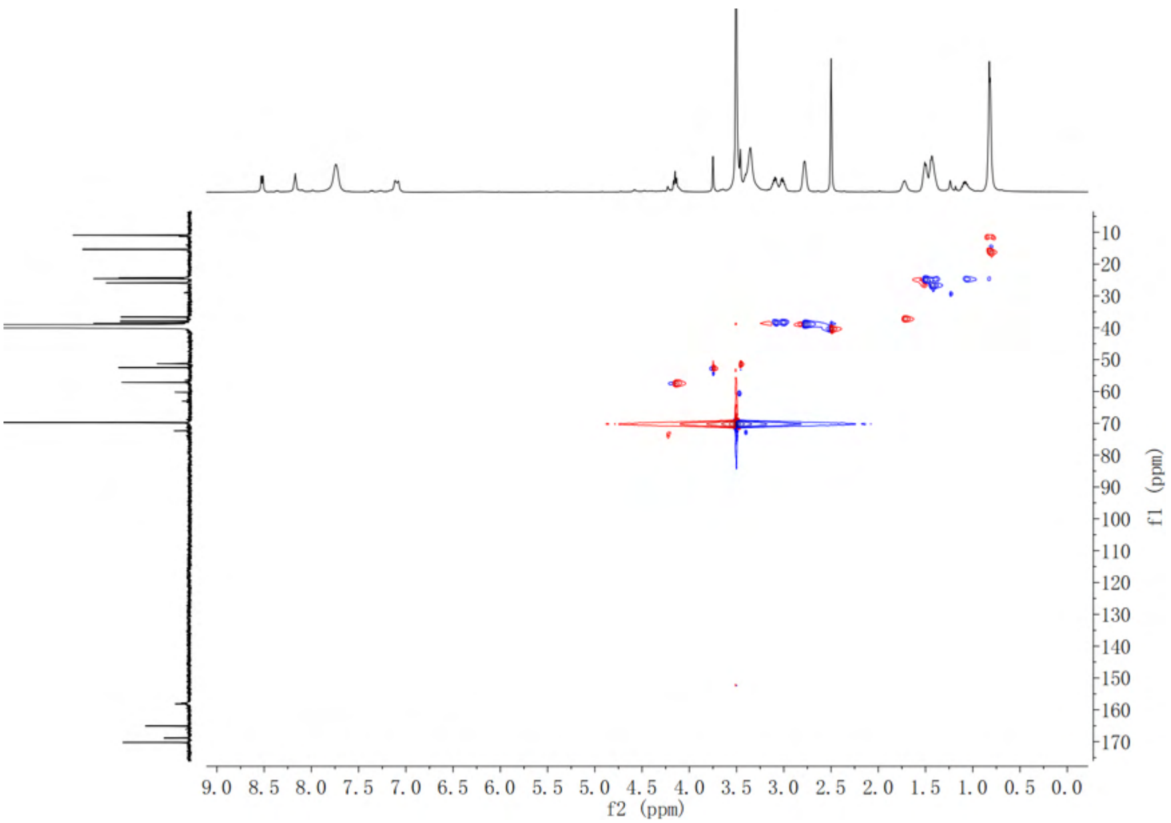

Supplementary Fig. 49. HSQC spectrum of CPI-2 (4) in DMSO-d_6

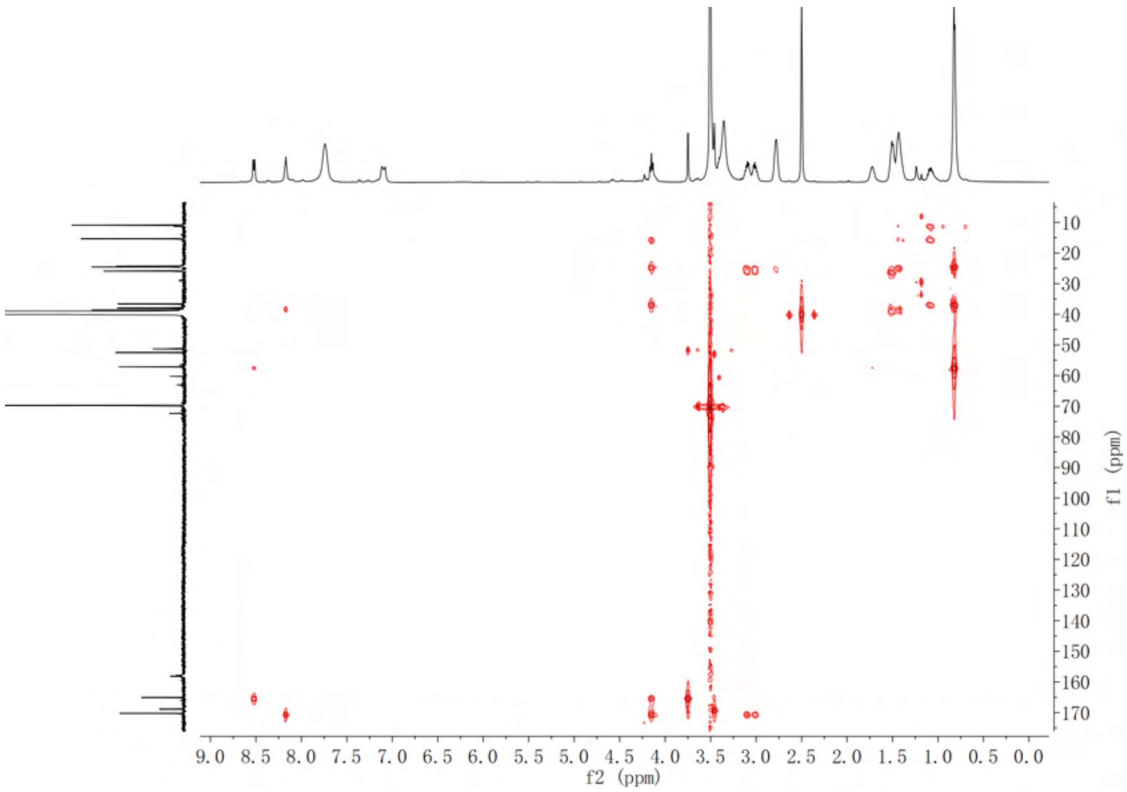

Supplementary Fig. 50. HMBC spectrum of CPI-2 (4) in DMSO-d_6

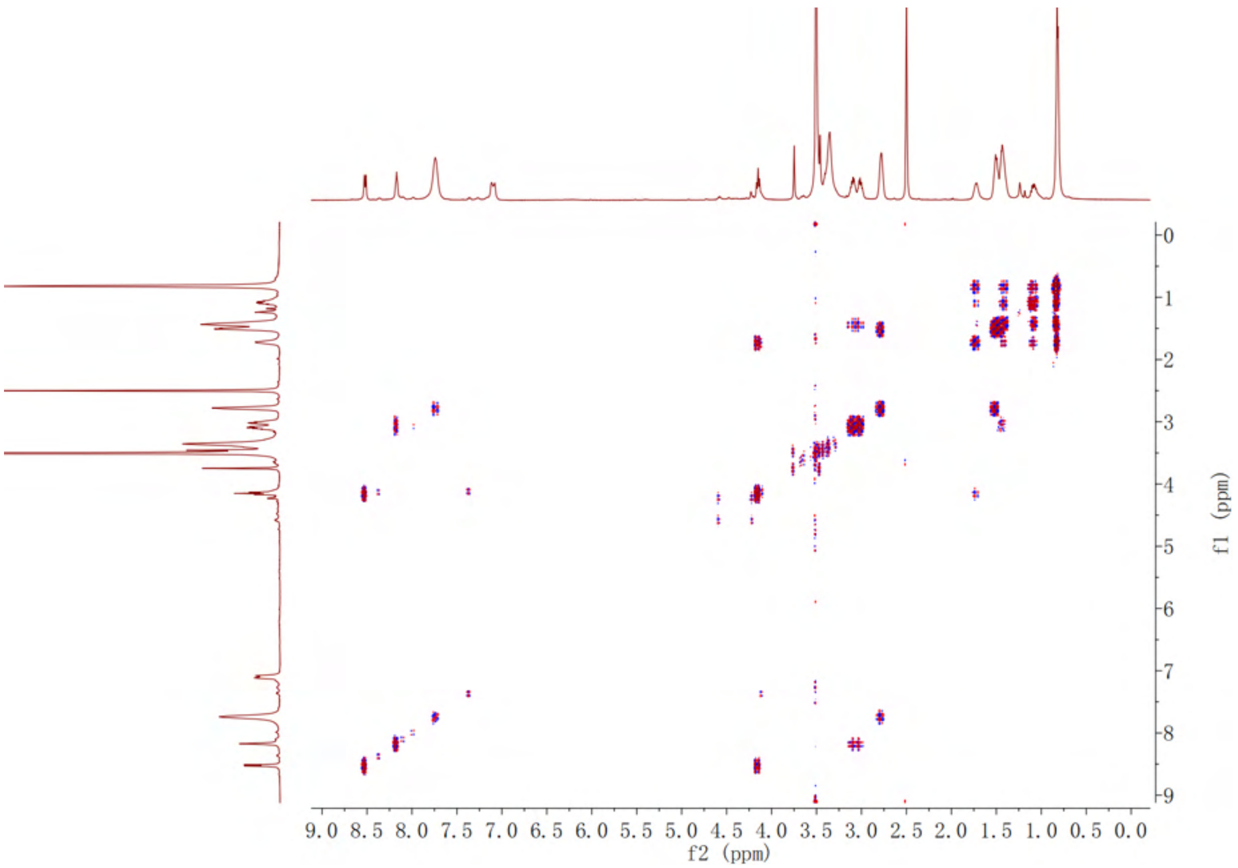

Supplementary Fig. 51. ^{1}H-^{1}H COSY spectrum of CPI-2 (4) in DMSO-d_6

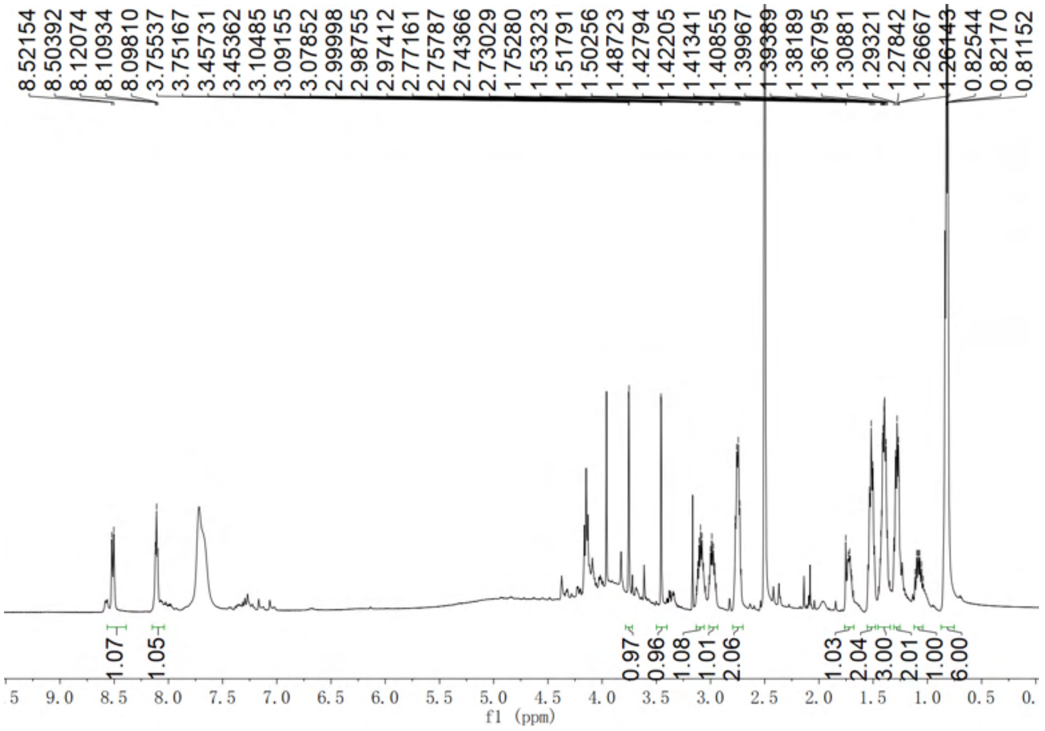

Supplementary Fig. 52. ^{1}H NMR spectrum of CPI-3 (5) in DMSO-d_6

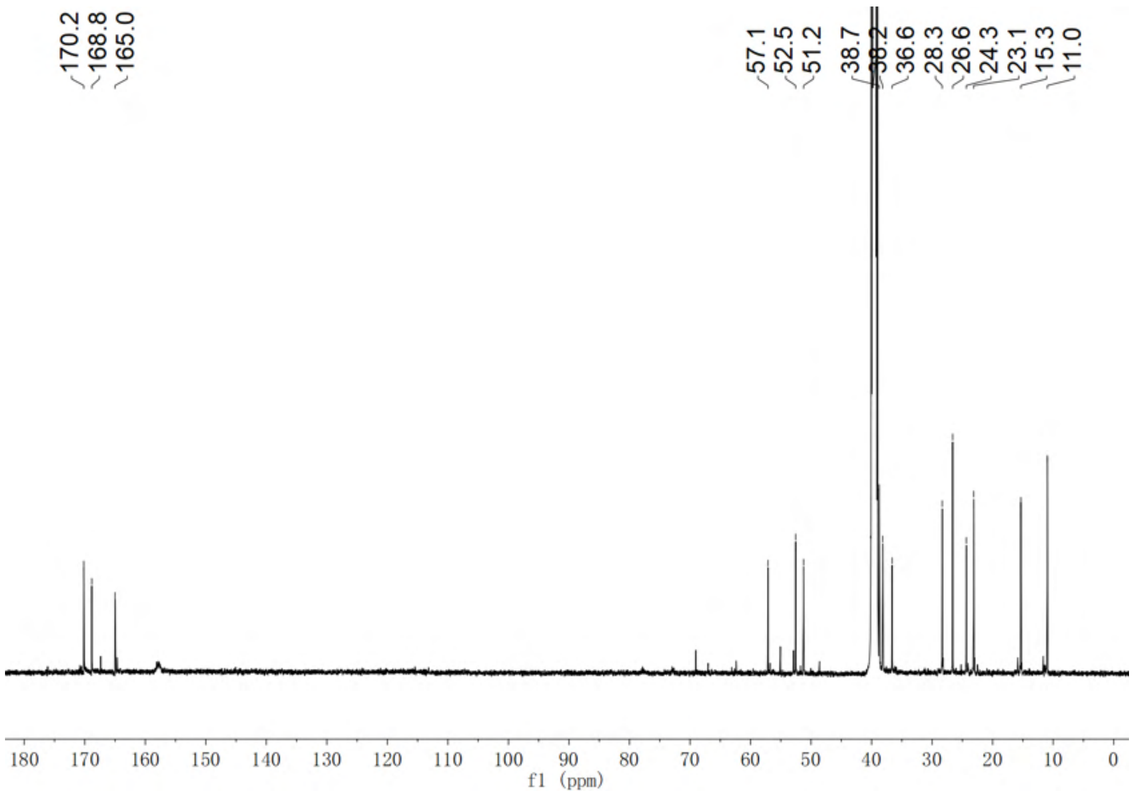

Supplementary Fig. 53. ^{13}C NMR spectrum of CPI-3 (5) in DMSO-d_6

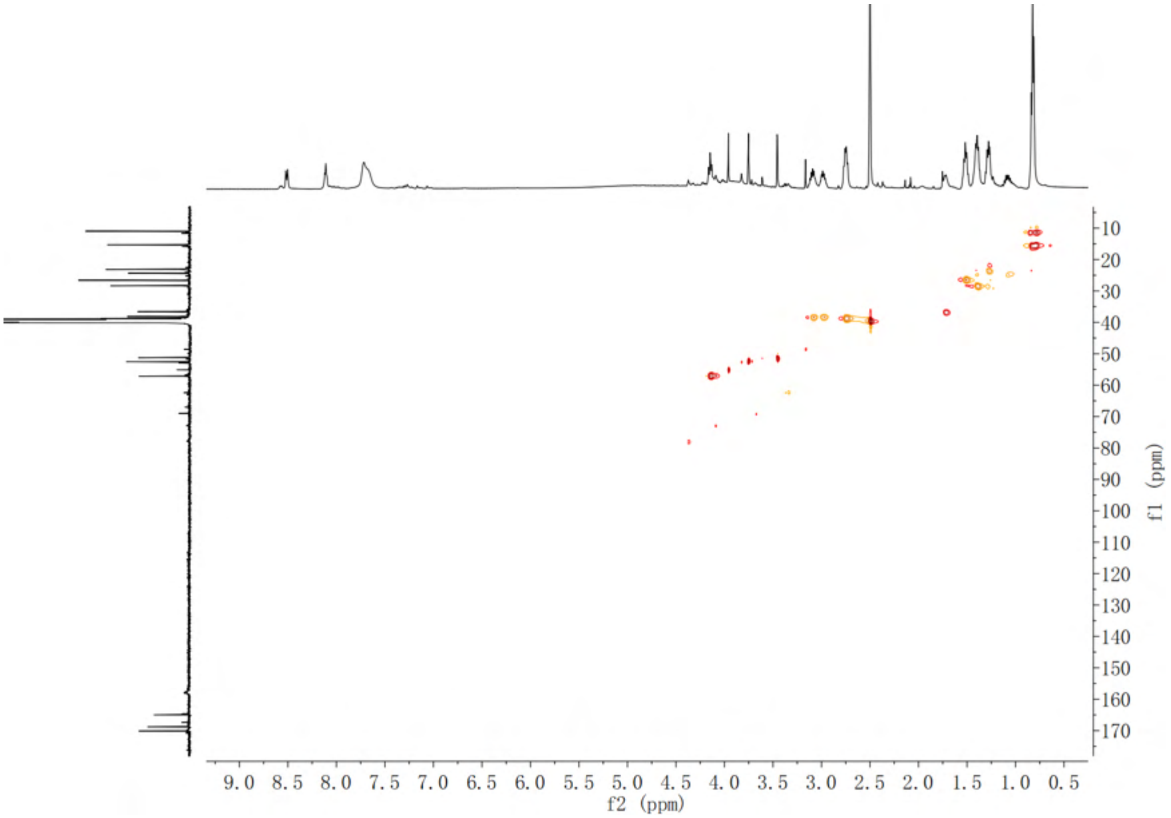

Supplementary Fig. 54. HSQC spectrum of CPI-3 (5) in DMSO-d_6

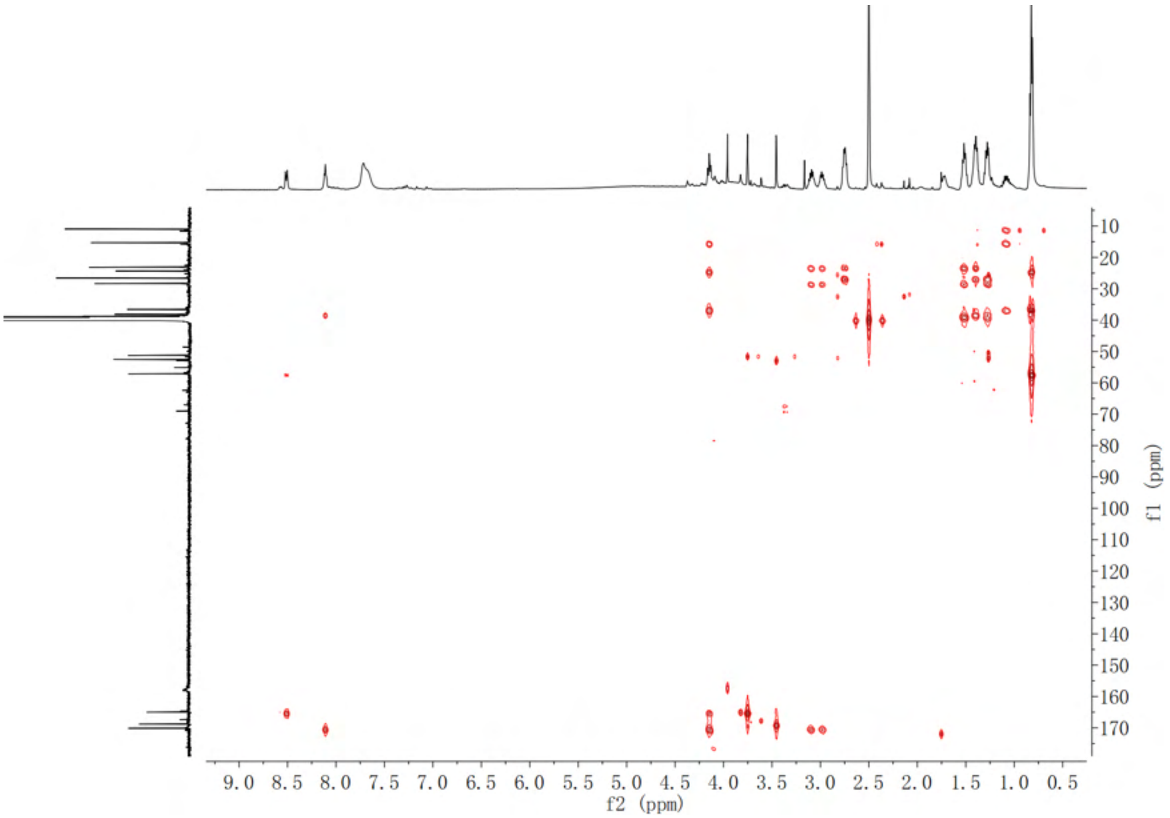

Supplementary Fig. 55. HMBC spectrum of CPI-3 (5) in DMSO-*d*$_6$

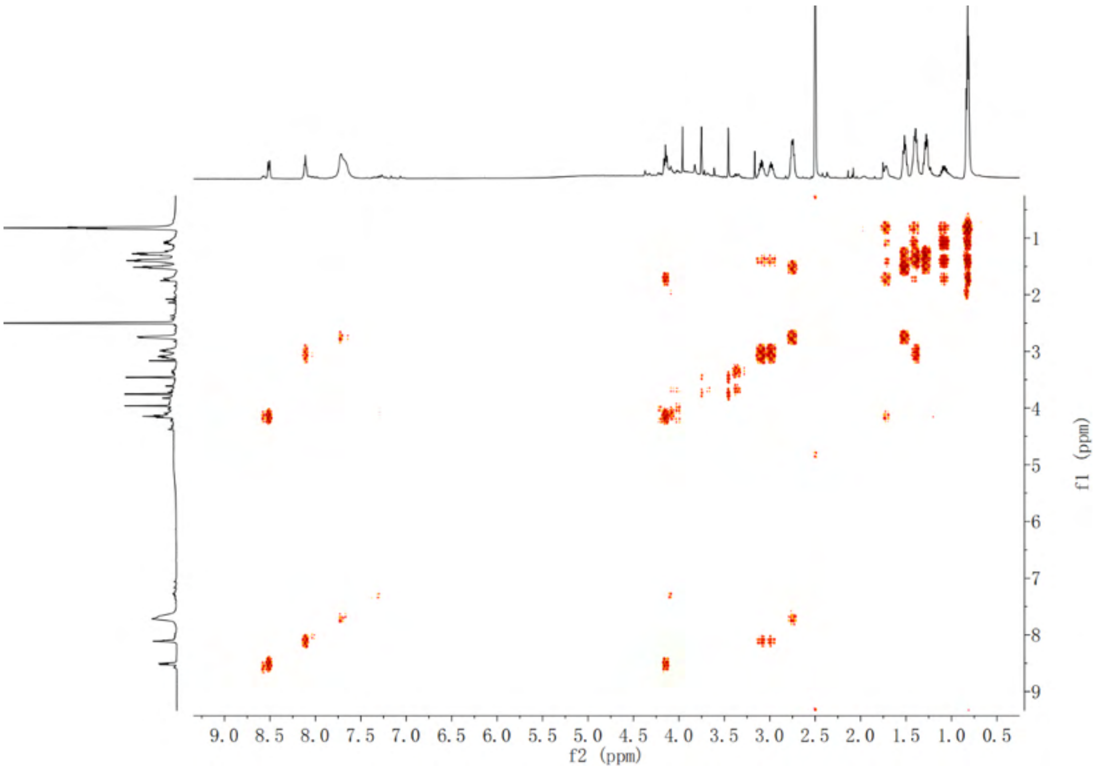

Supplementary Fig. 56. ^{1}H-^{1}H COSY spectrum of CPI-3 (5) in DMSO-*d*$_6$

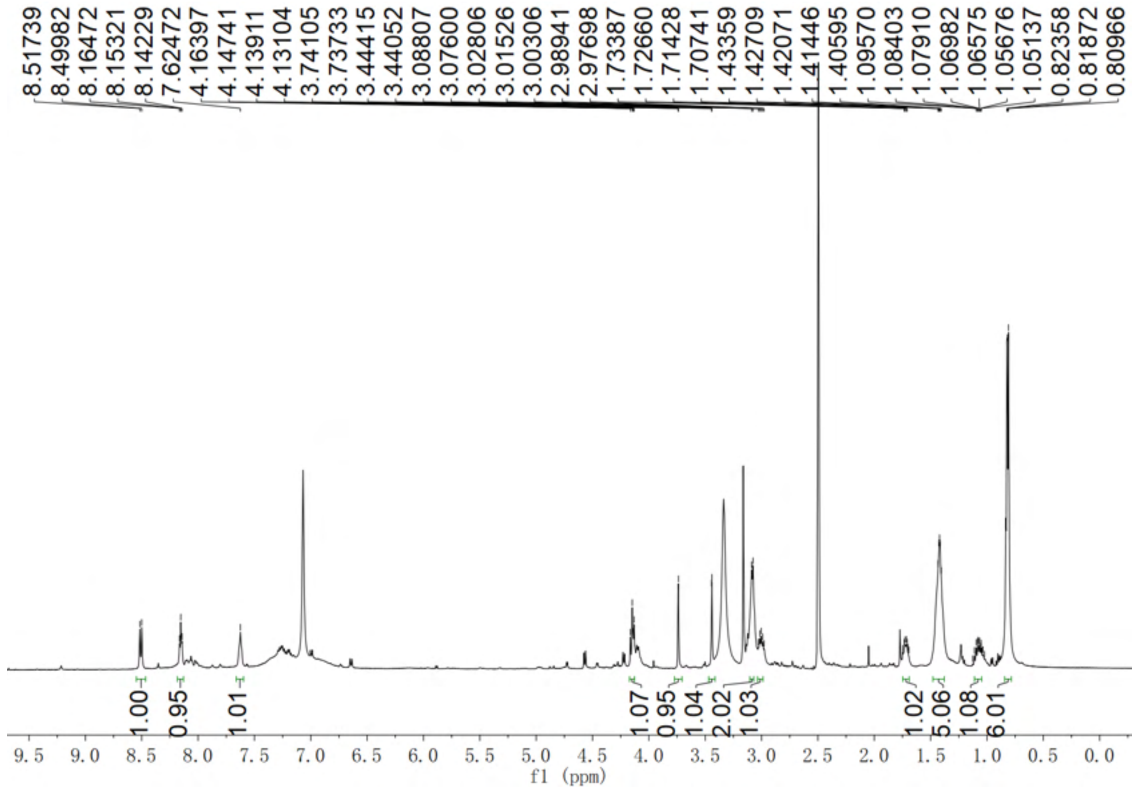

Supplementary Fig. 57. ^{1}H NMR spectrum of compound 6 in DMSO-d_6

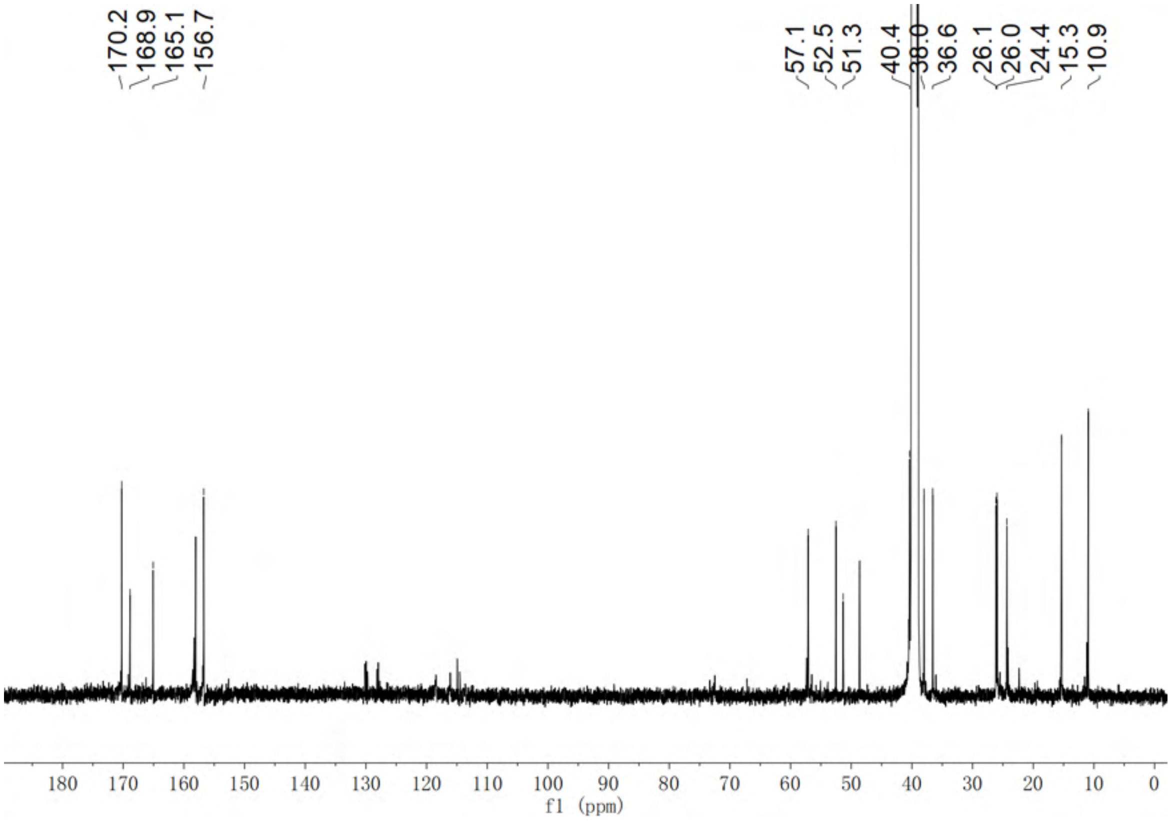

Supplementary Fig. 58. ^{13}C NMR spectrum of compound 6 in DMSO-d_6

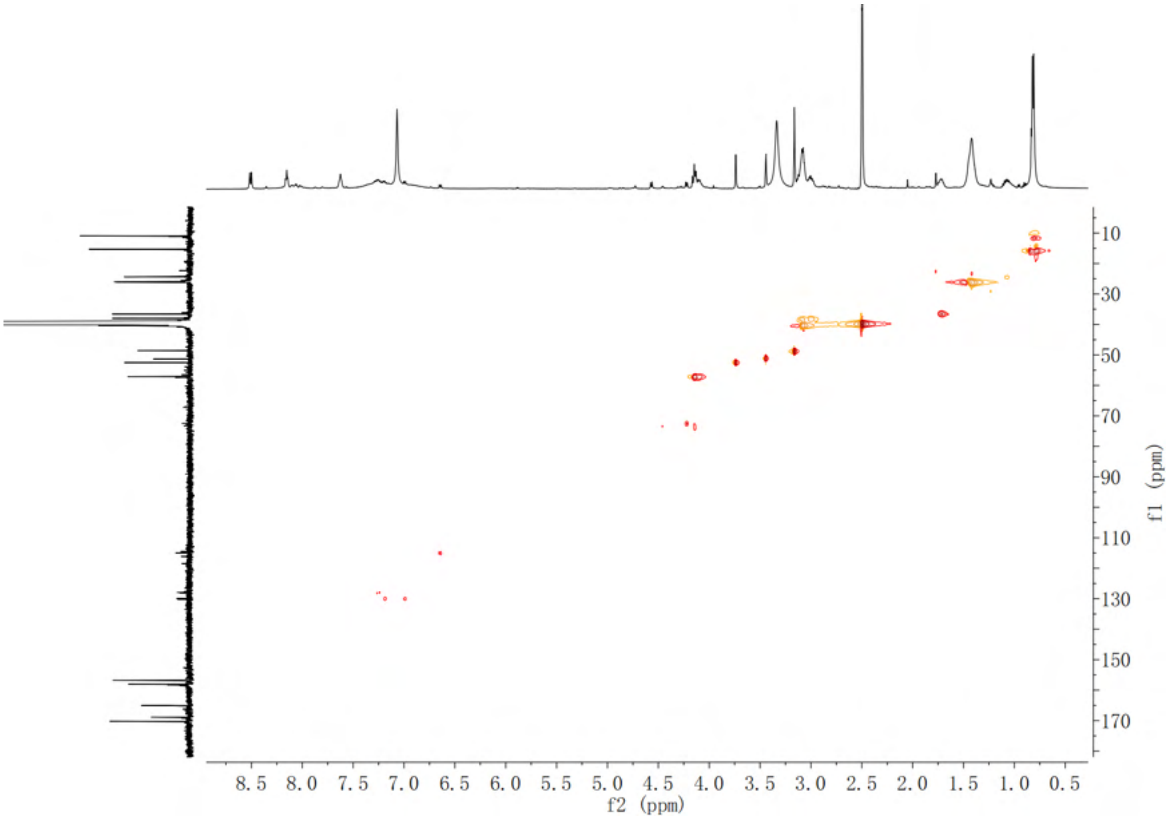

Supplementary Fig. 59. HSQC spectrum of compound 6 in DMSO-d_6

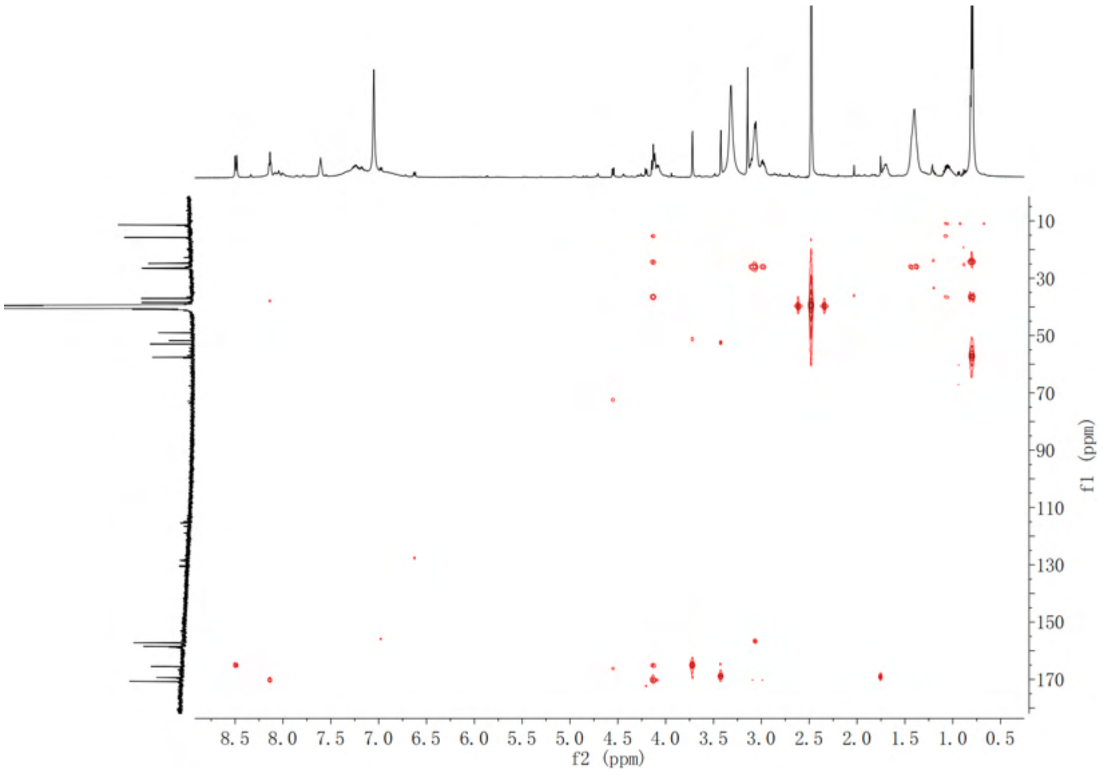

Supplementary Fig. 60. HMBC spectrum of compound 6 in DMSO-d_6

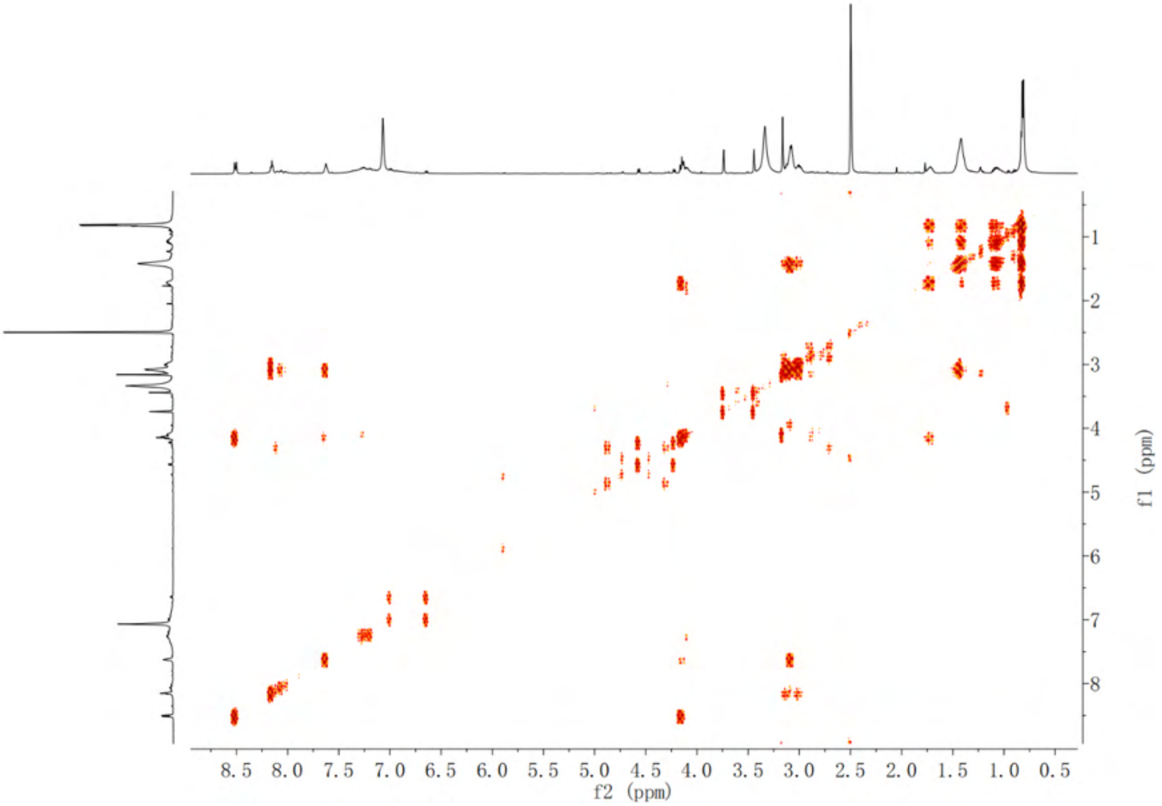

Supplementary Fig. 61. ^{1}H-^{1}H COSY spectrum of compound 6 in DMSO-d_6

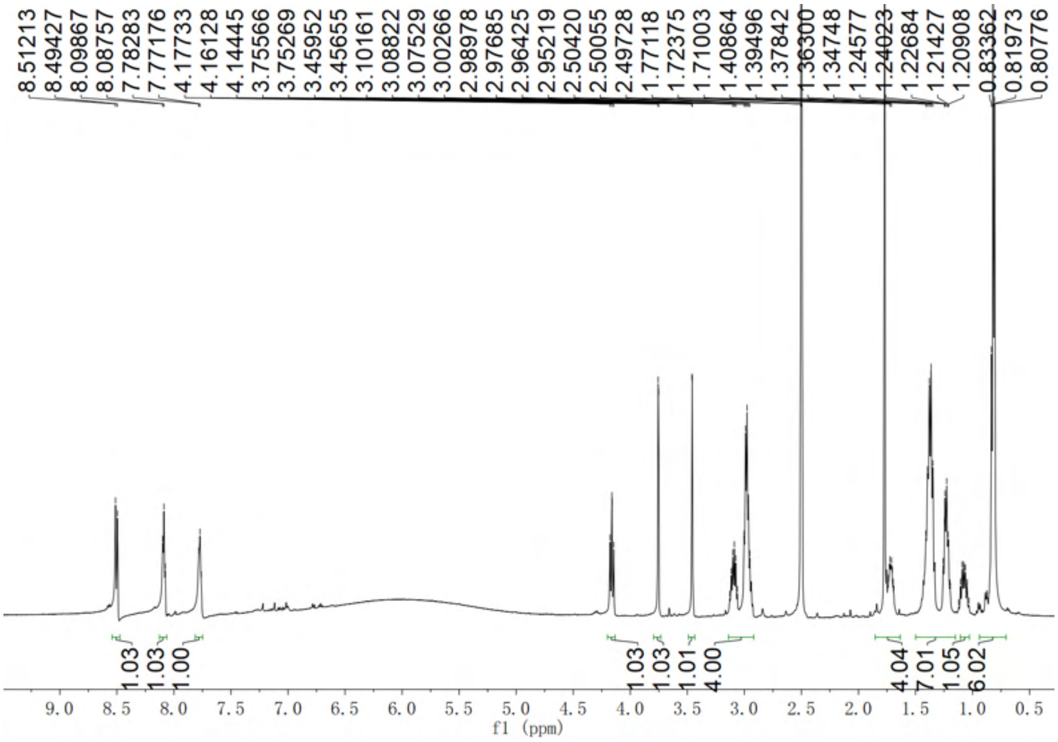

Supplementary Fig. 62. ^{1}H NMR spectrum of compound 7 in DMSO-d_6

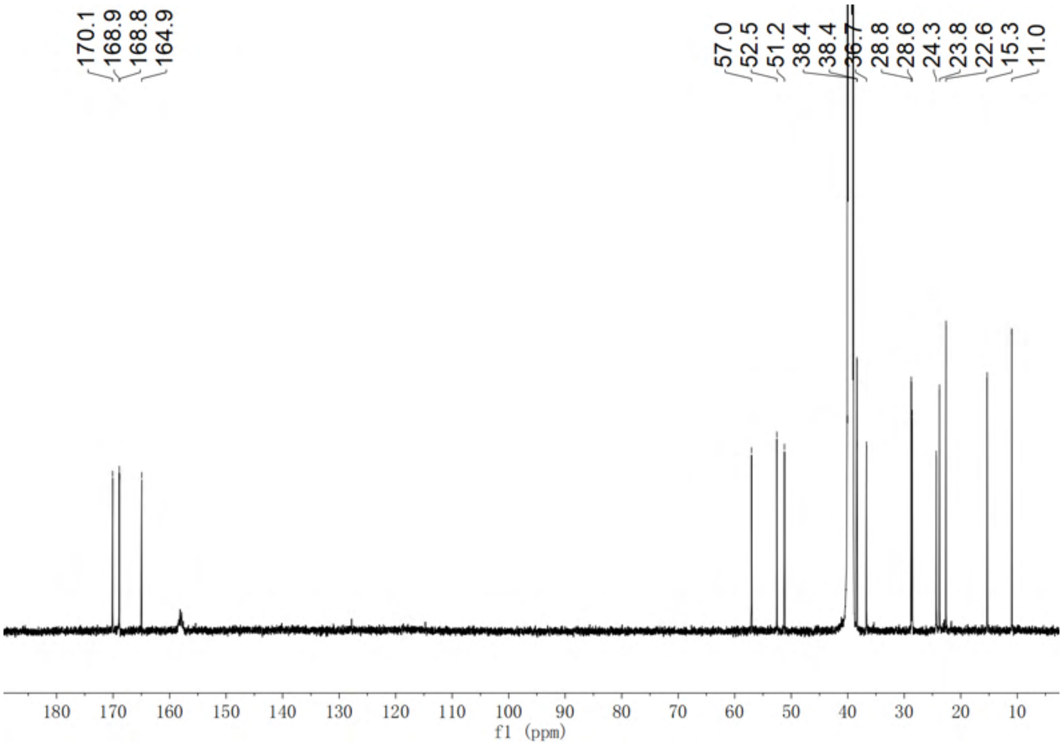

Supplementary Fig. 63. ^{13}C NMR spectrum of compound 7 in DMSO-d_6

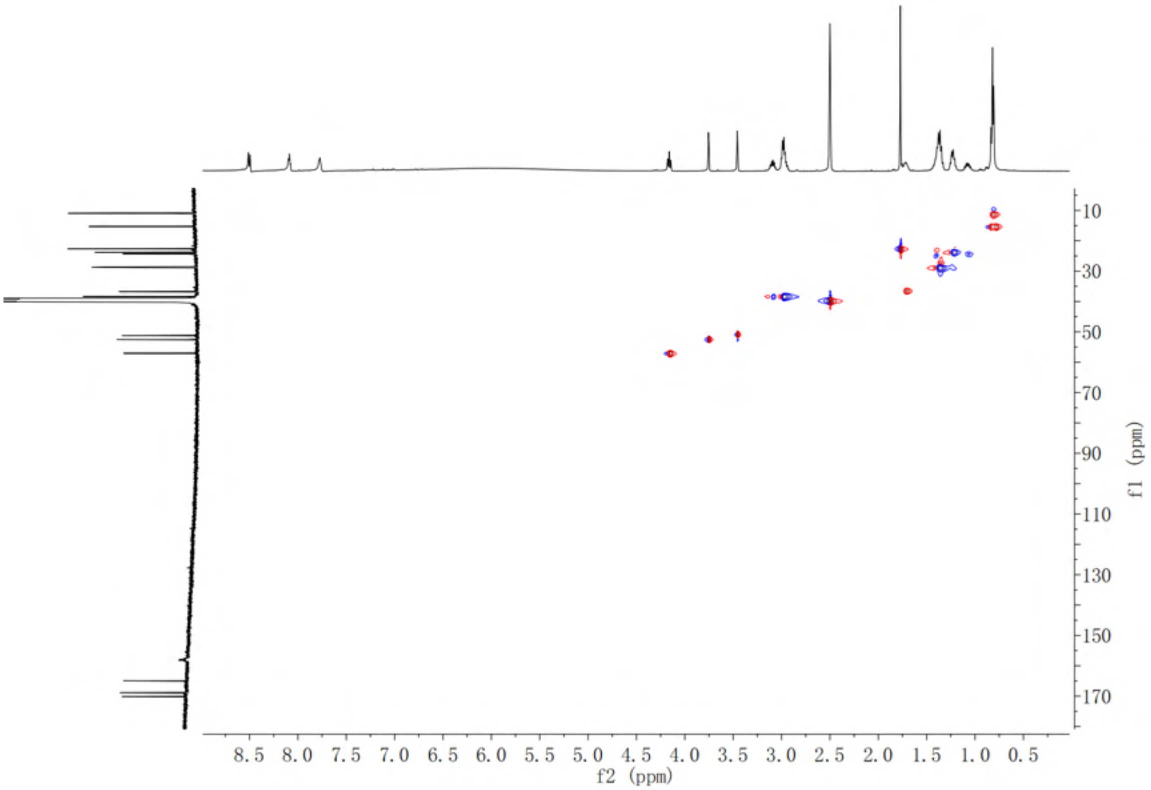

Supplementary Fig. 64. HSQC spectrum of compound 7 in DMSO-d_6

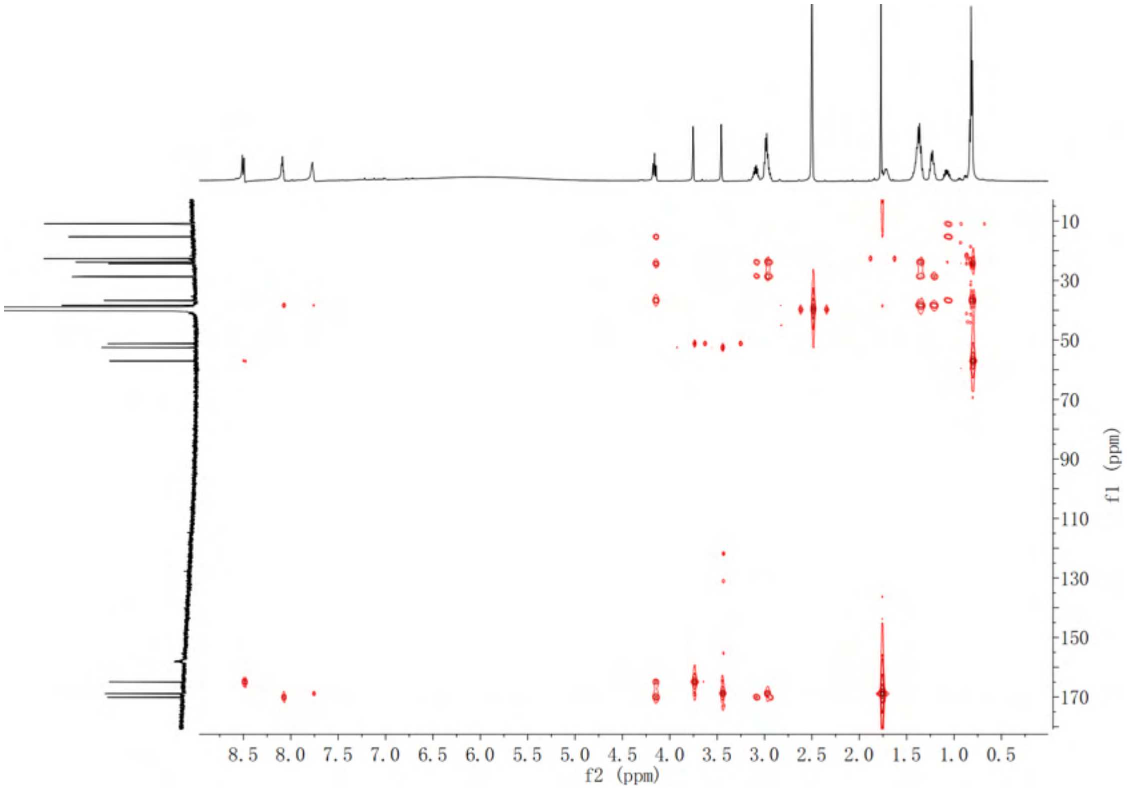

Supplementary Fig. 65. HMBC spectrum of compound 7 in DMSO-d_6

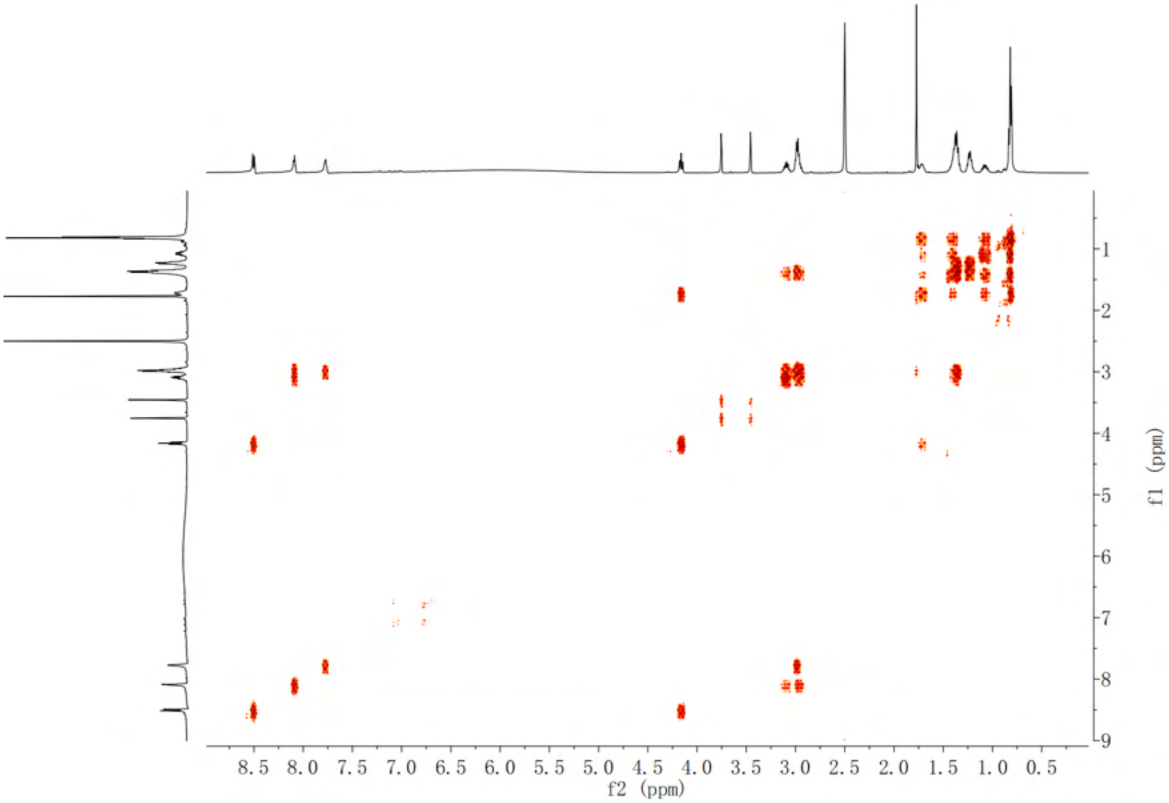

Supplementary Fig. 66. ^{1}H-^{1}H COSY spectrum of compound 7 in DMSO-d_6

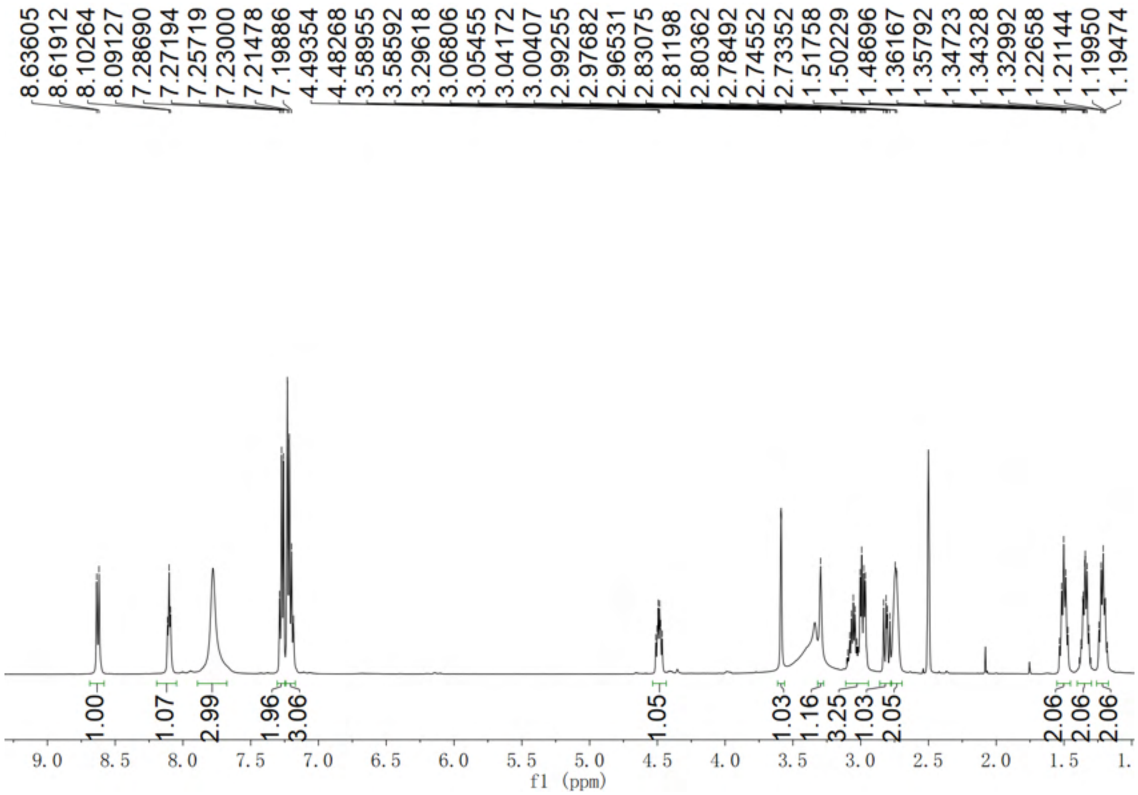

Supplementary Fig. 67. ^{1}**H NMR spectrum of compound 8 in DMSO-**d_6

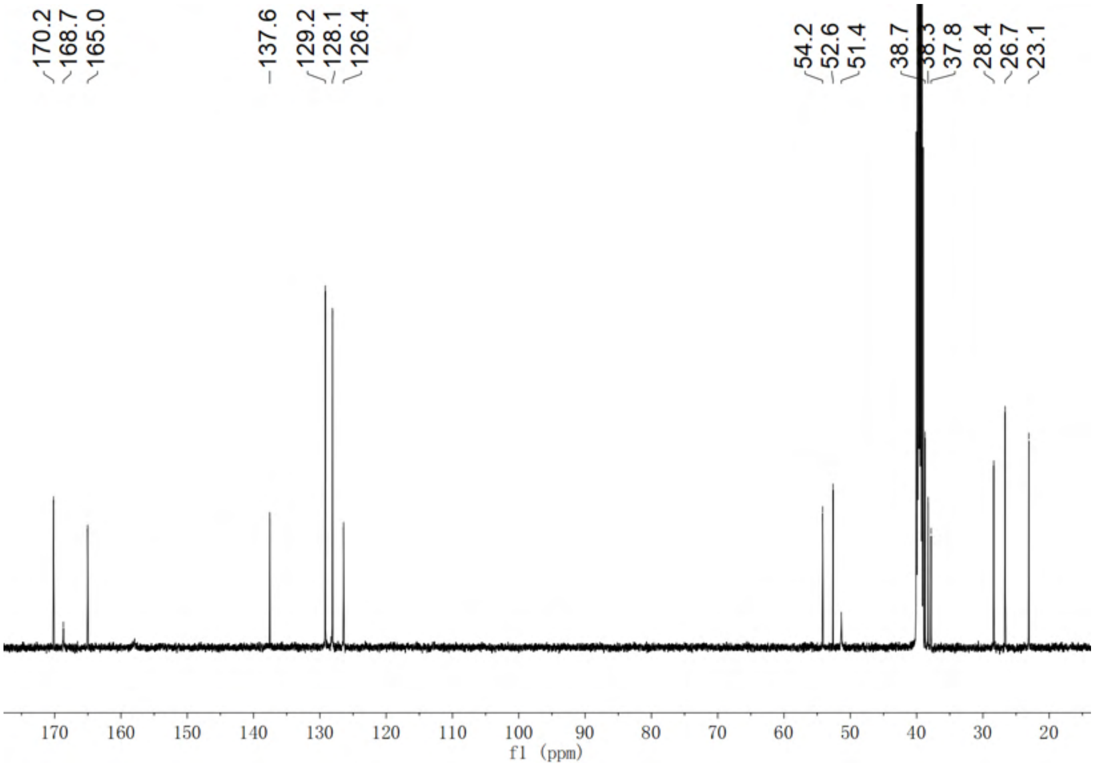

Supplementary Fig. 68. ^{13}**C NMR spectrum of compound 8 in DMSO-**d_6

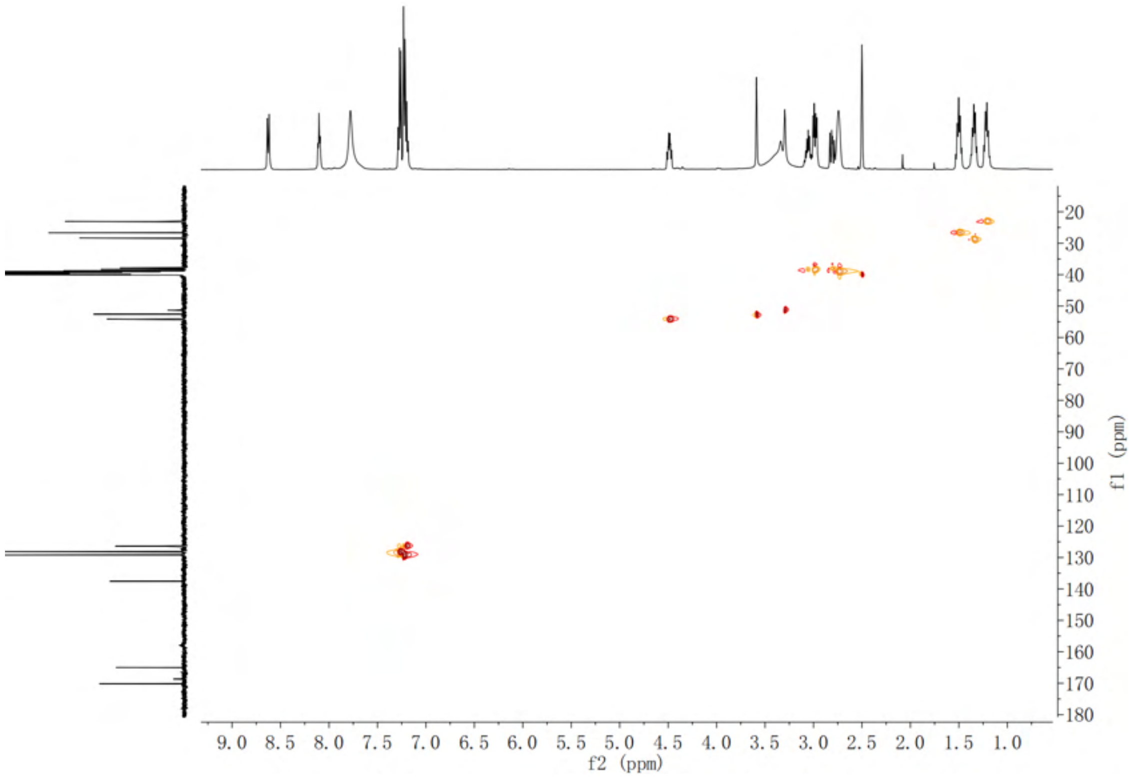

Supplementary Fig. 69. HSQC spectrum of compound 8 in DMSO-d_6

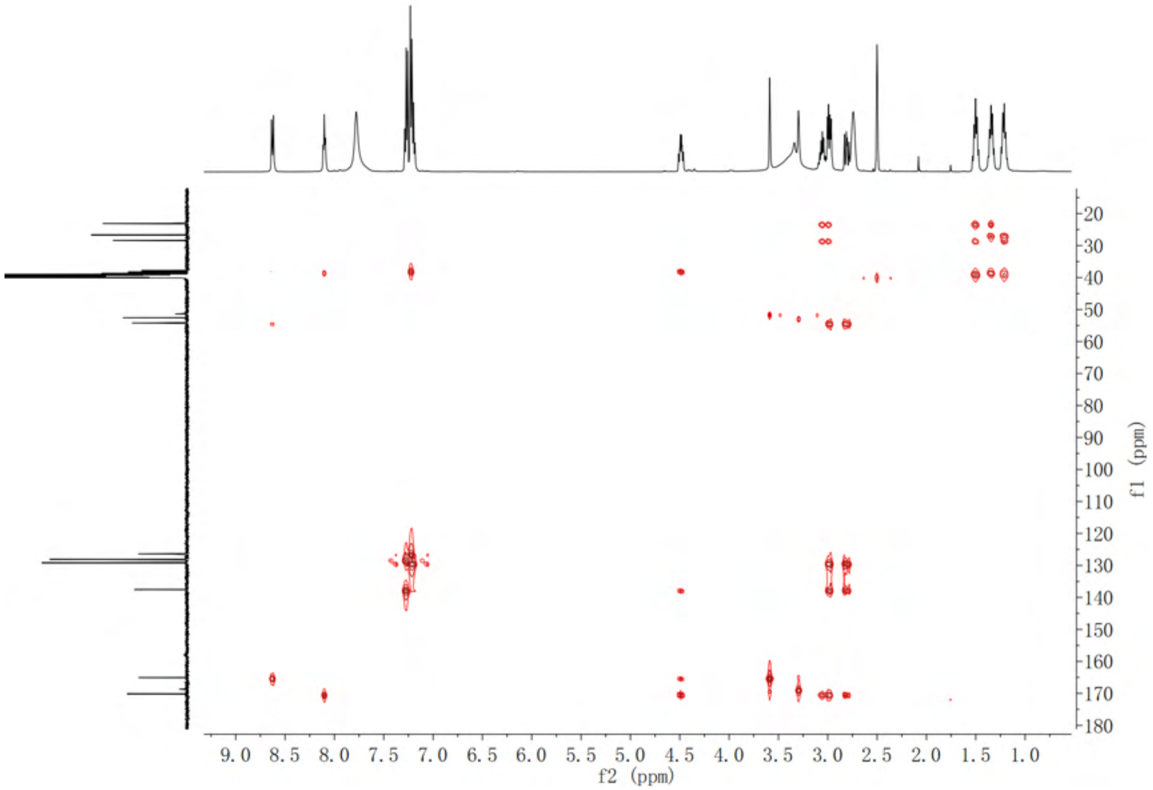

Supplementary Fig. 70. HMBC spectrum of compound 8 in DMSO-d_6

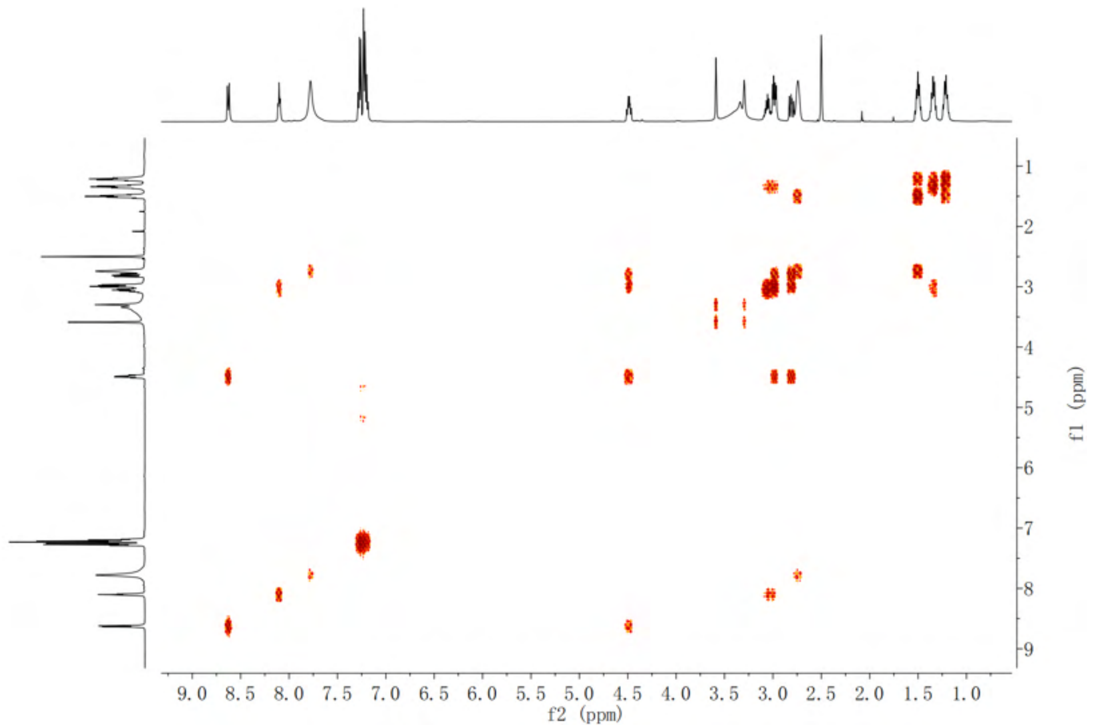

Supplementary Fig. 71. ^{1}H-^{1}H COSY spectrum of compound 8 in DMSO-*d*$_6$

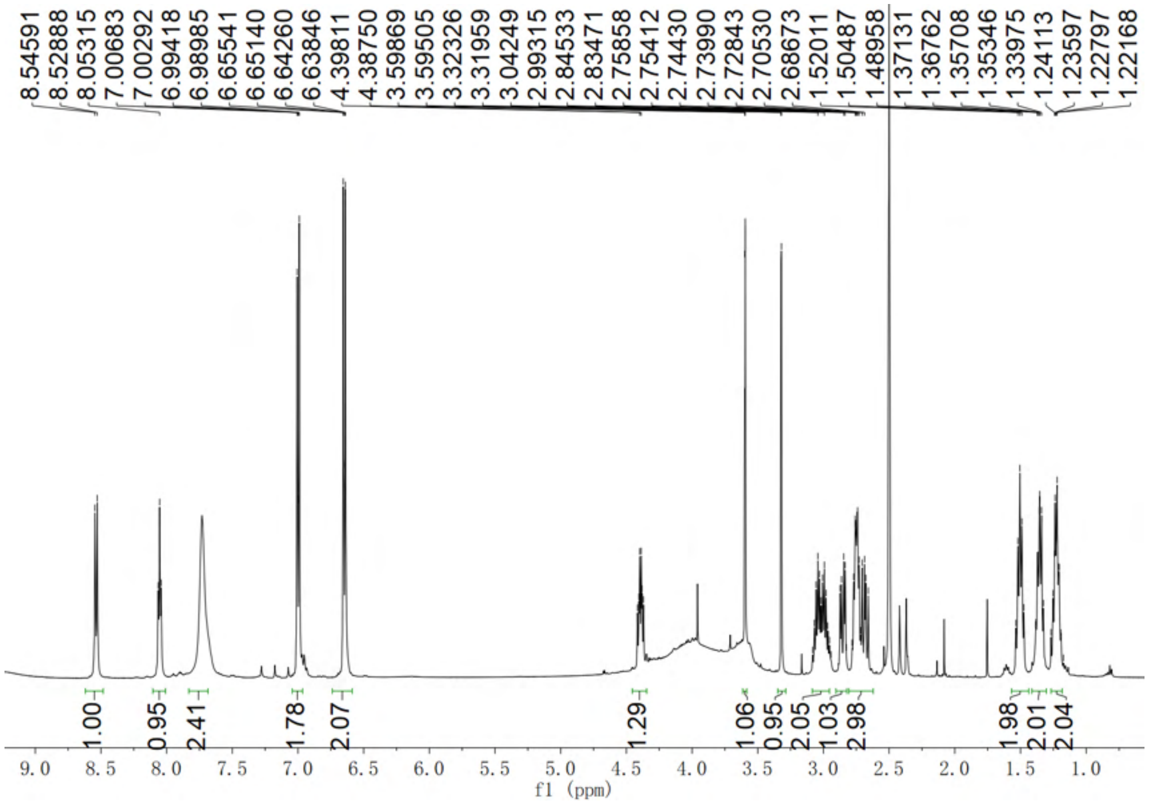

Supplementary Fig. 72. ^{1}H NMR spectrum of compound 9 in DMSO-*d*$_6$

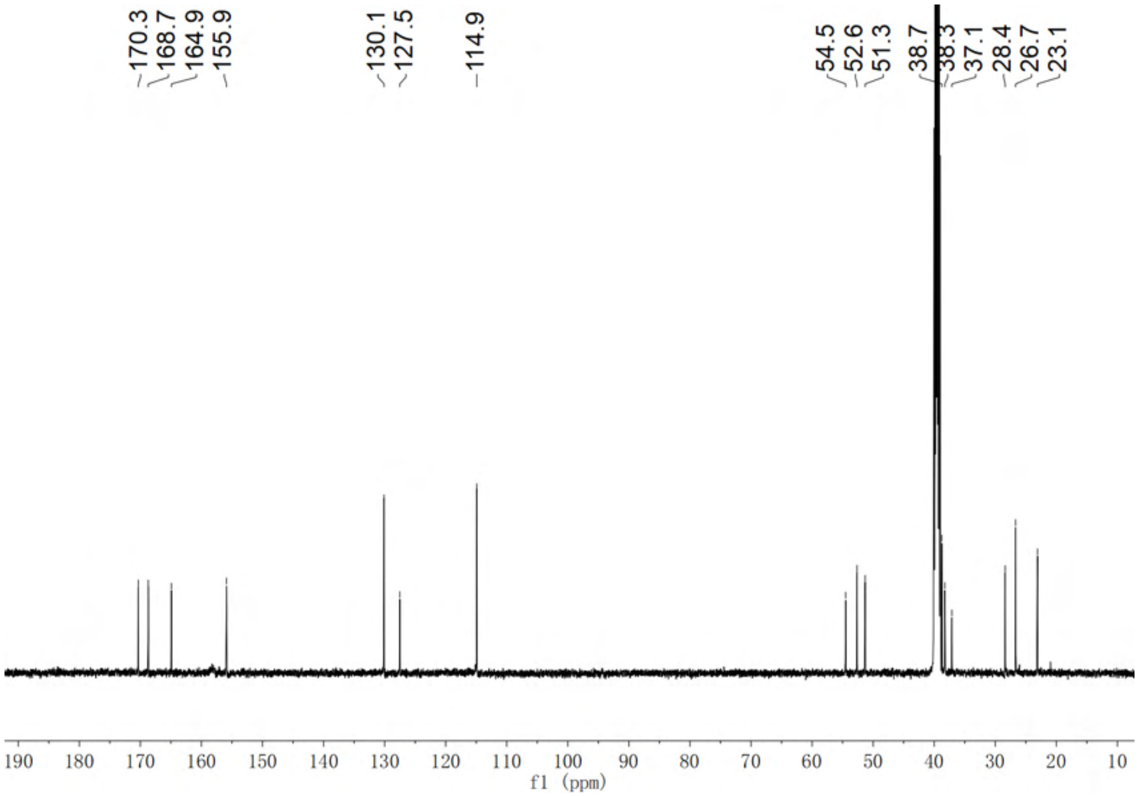

Supplementary Fig. 73. ^{13}C NMR spectrum of compound 9 in DMSO-d_6

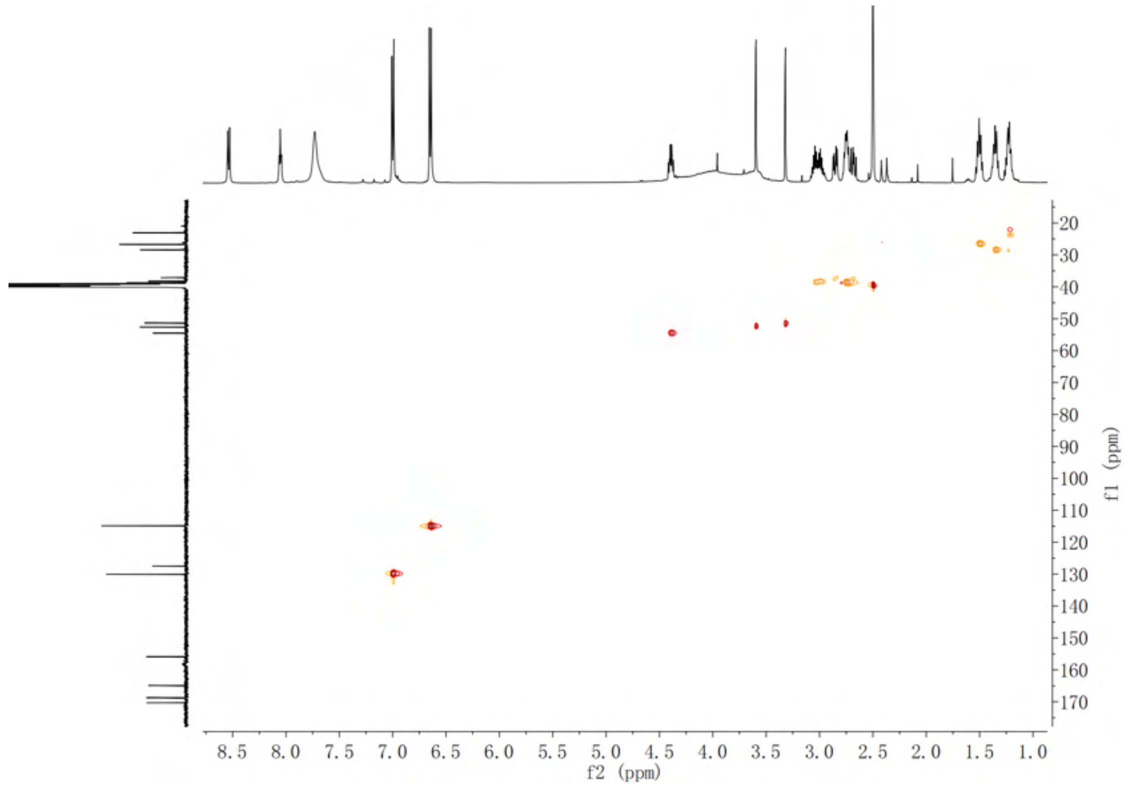

Supplementary Fig. 74. HSQC spectrum of compound 9 in DMSO-d_6

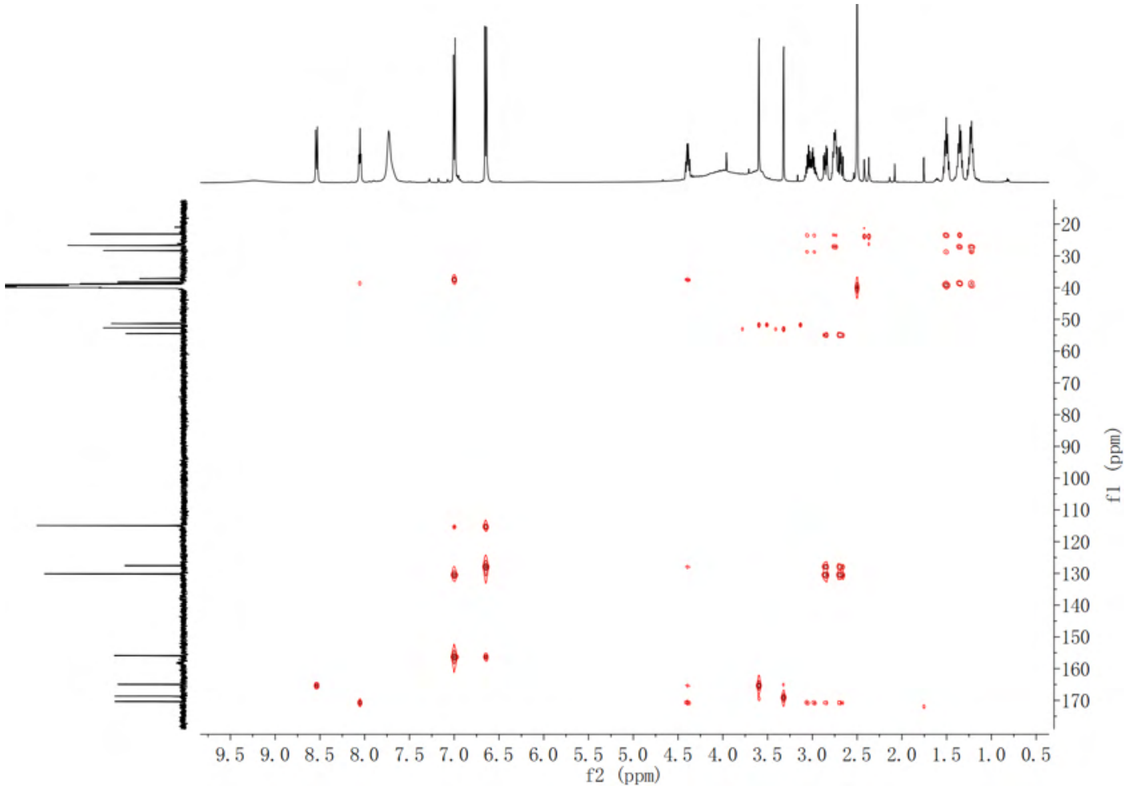

Supplementary Fig. 75. HMBC spectrum of compound 9 in DMSO-d_6

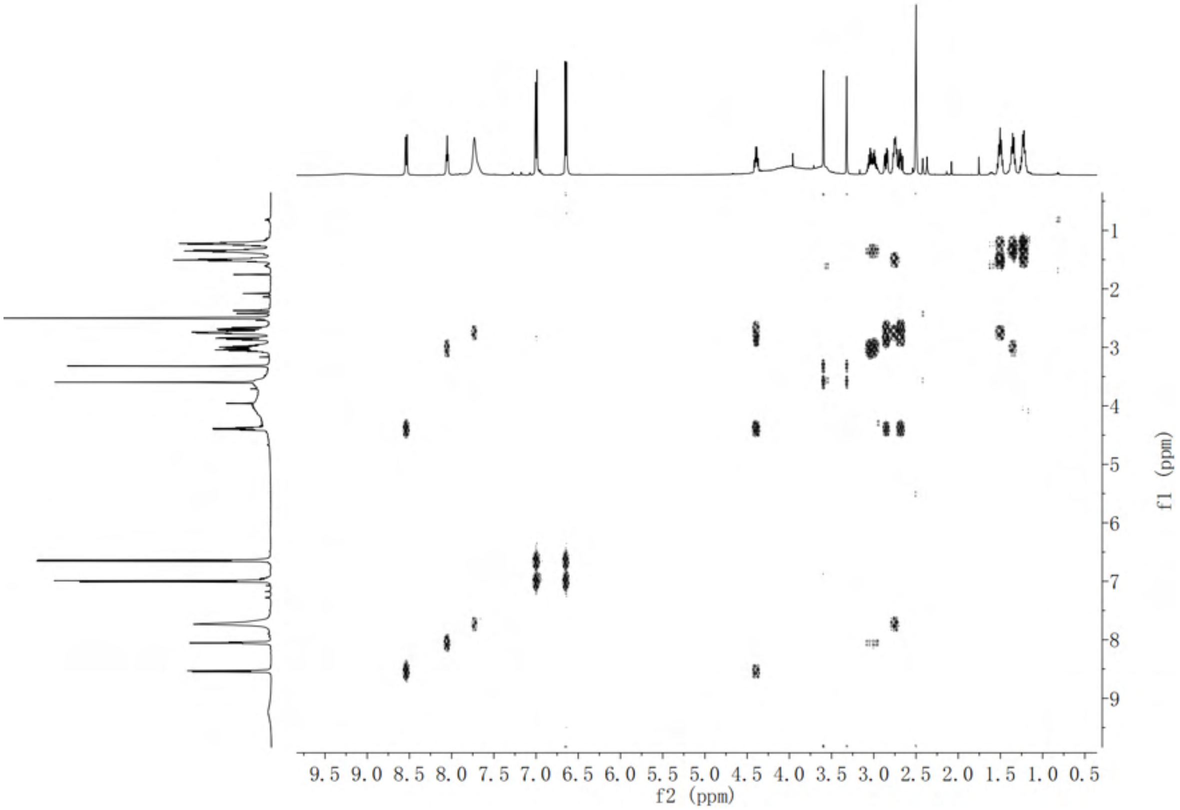

Supplementary Fig. 76. ^{1}H-^{1}H COSY spectrum of compound 9 in DMSO-d_6

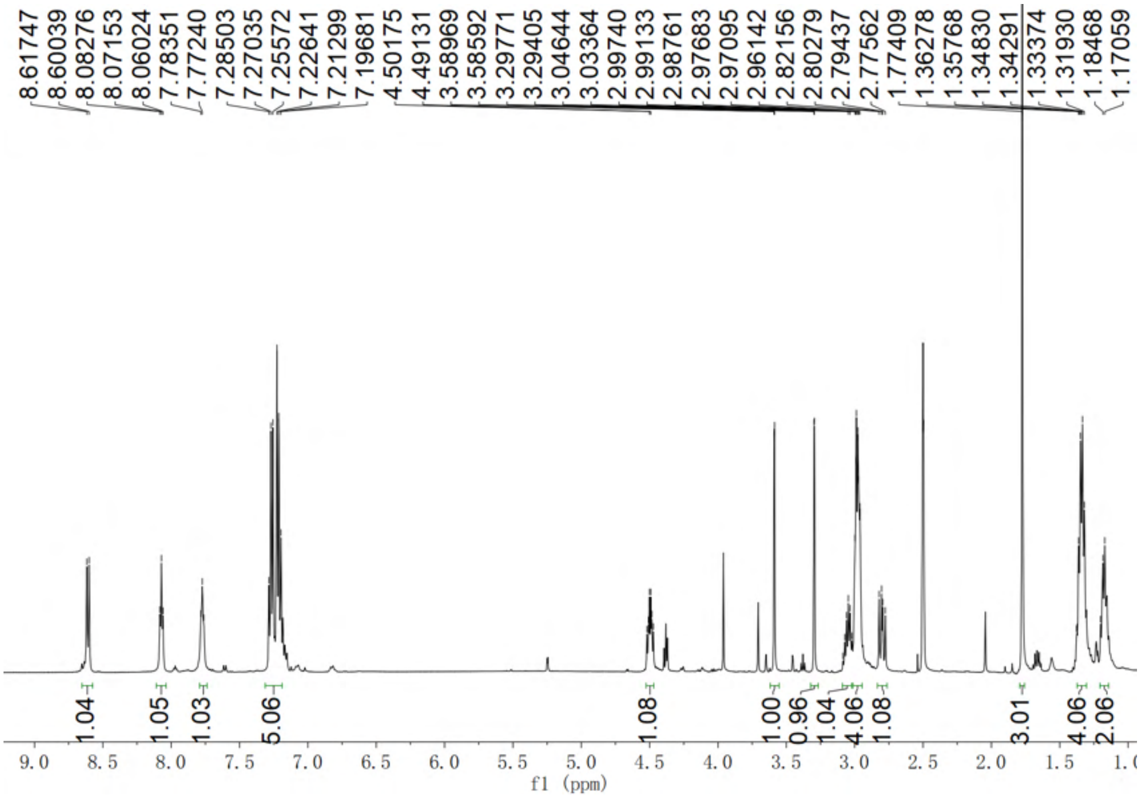

Supplementary Fig. 77. ^{1}H NMR spectrum of compound 10 in DMSO-d_6

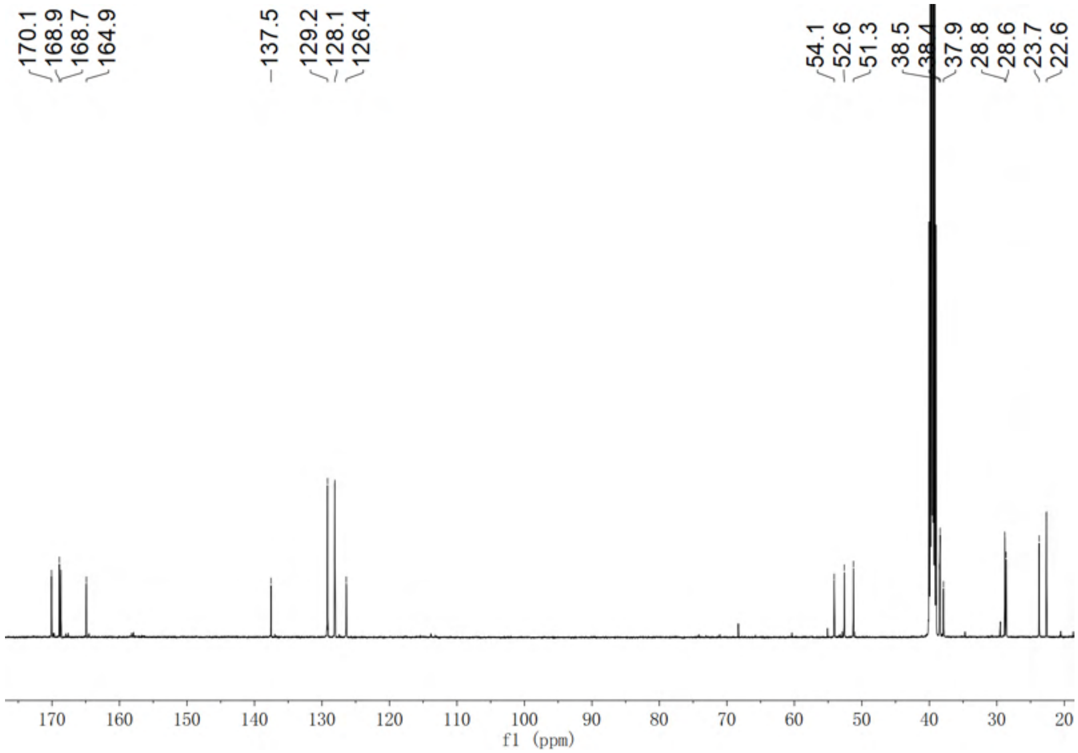

Supplementary Fig. 78. ^{13}C NMR spectrum of compound 10 in DMSO-d_6

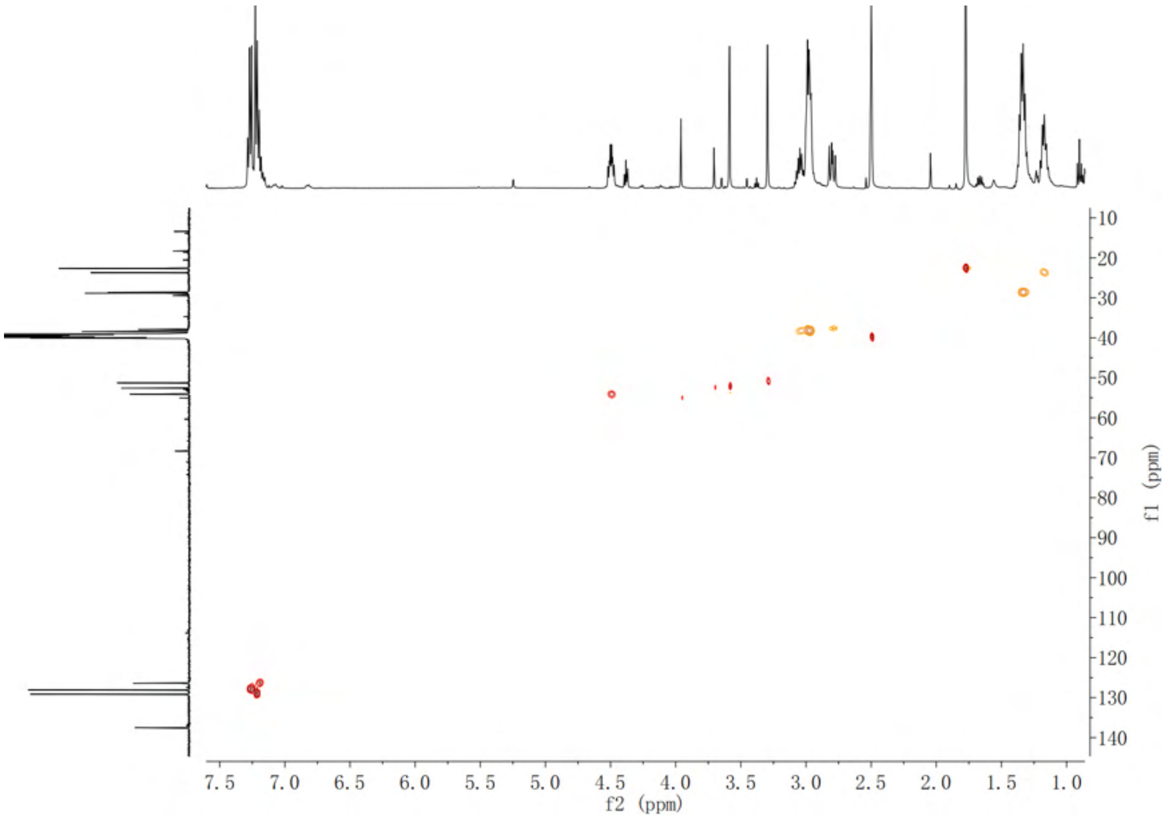

Supplementary Fig. 79. HSQC spectrum of compound 10 in DMSO-d_6

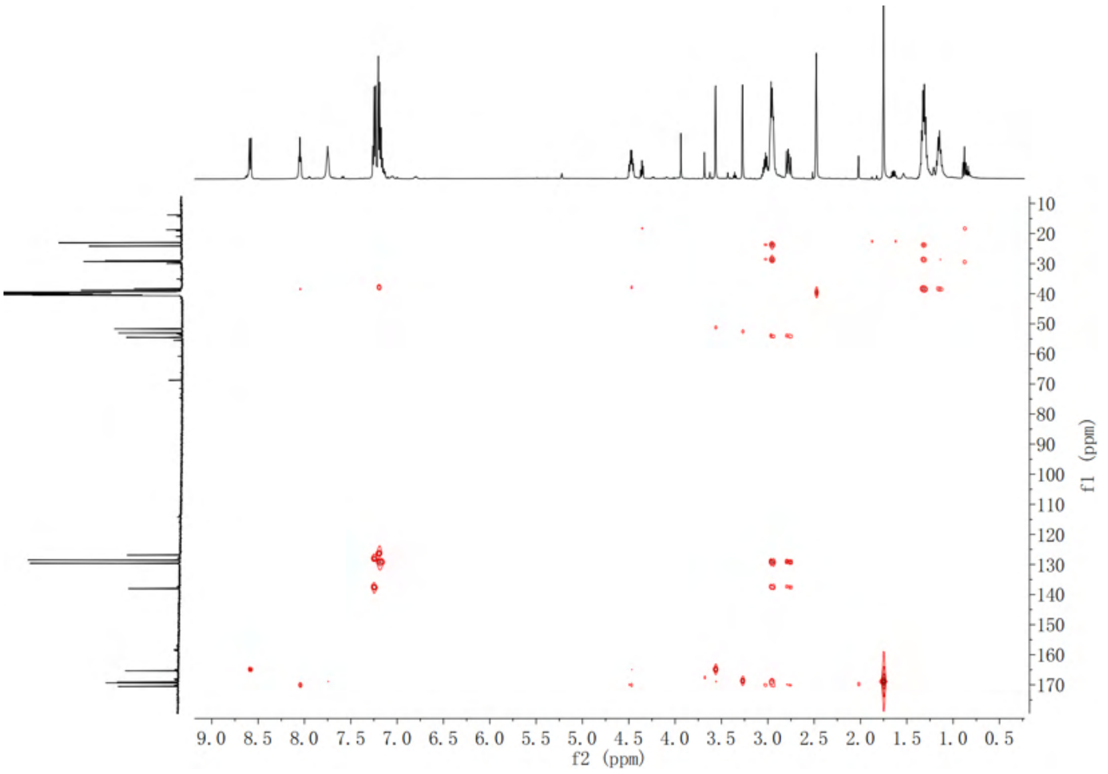

Supplementary Fig. 80. HMBC spectrum of compound 10 in DMSO-d_6

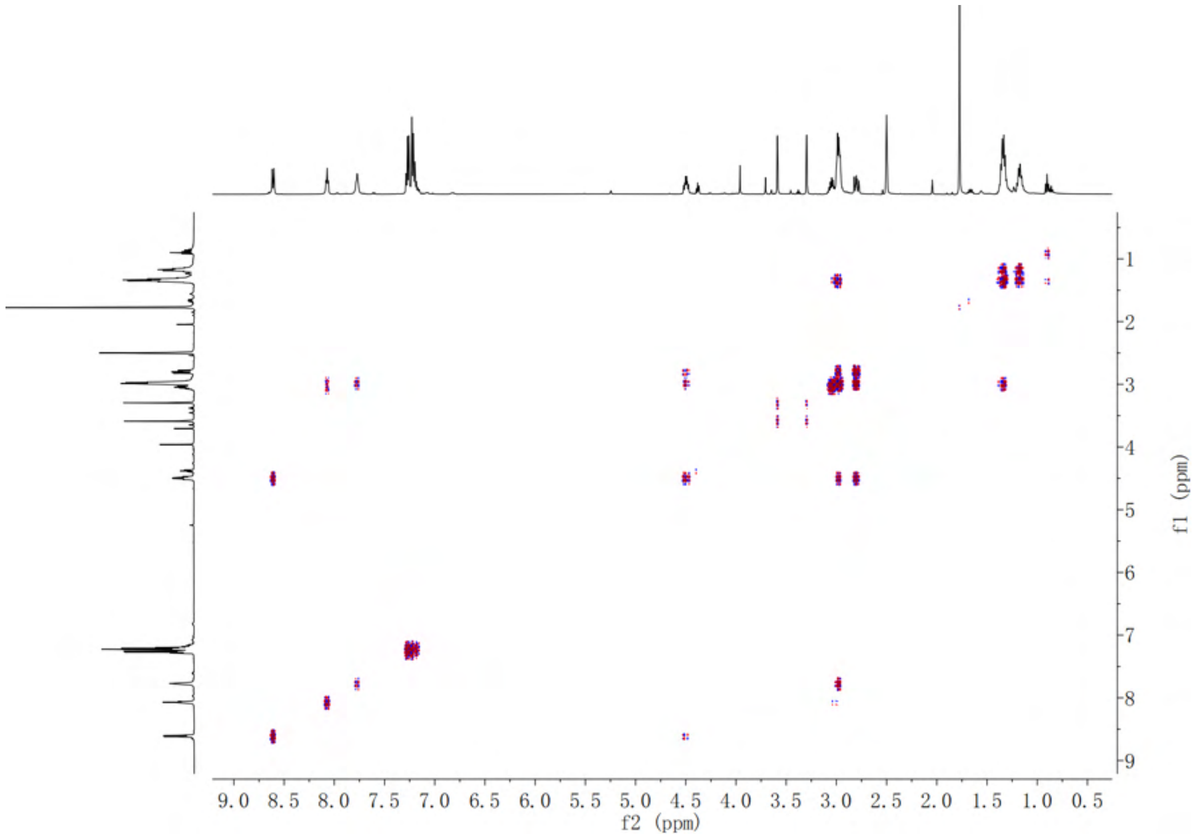

Supplementary Fig. 81. ^{1}H-^{1}H COSY spectrum of compound 10 in DMSO-d_6

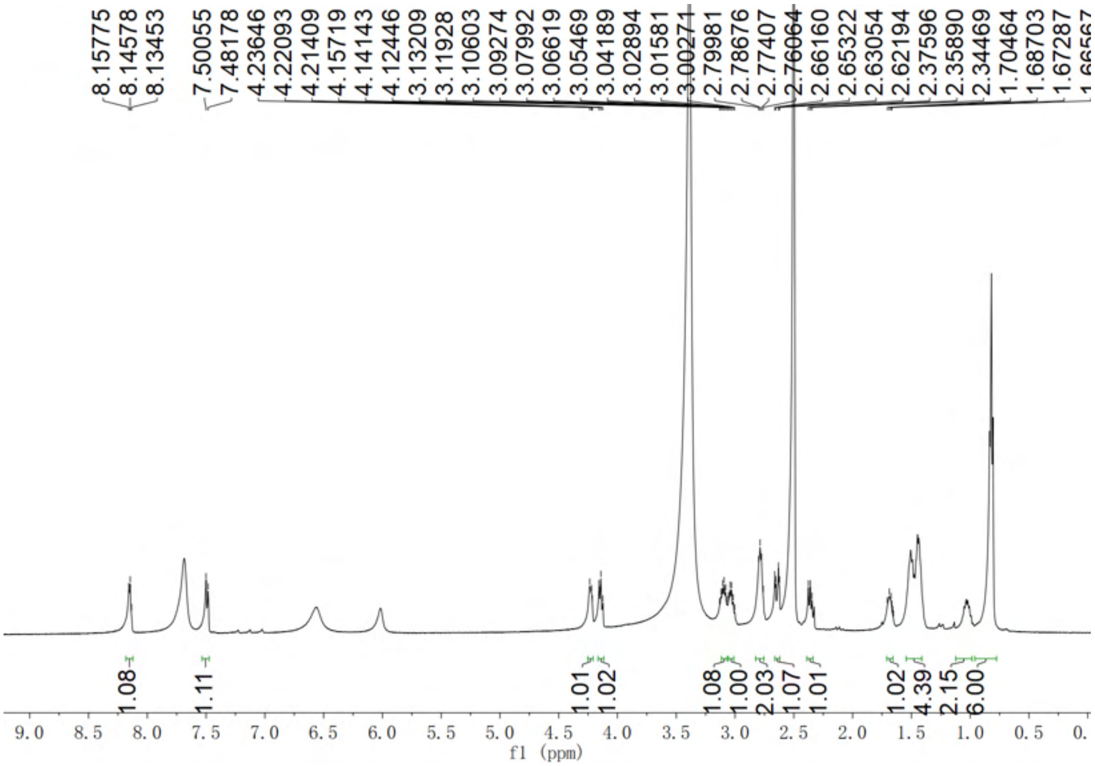

Supplementary Fig. 82. ^{1}H NMR spectrum of compound 12 in DMSO-d_6

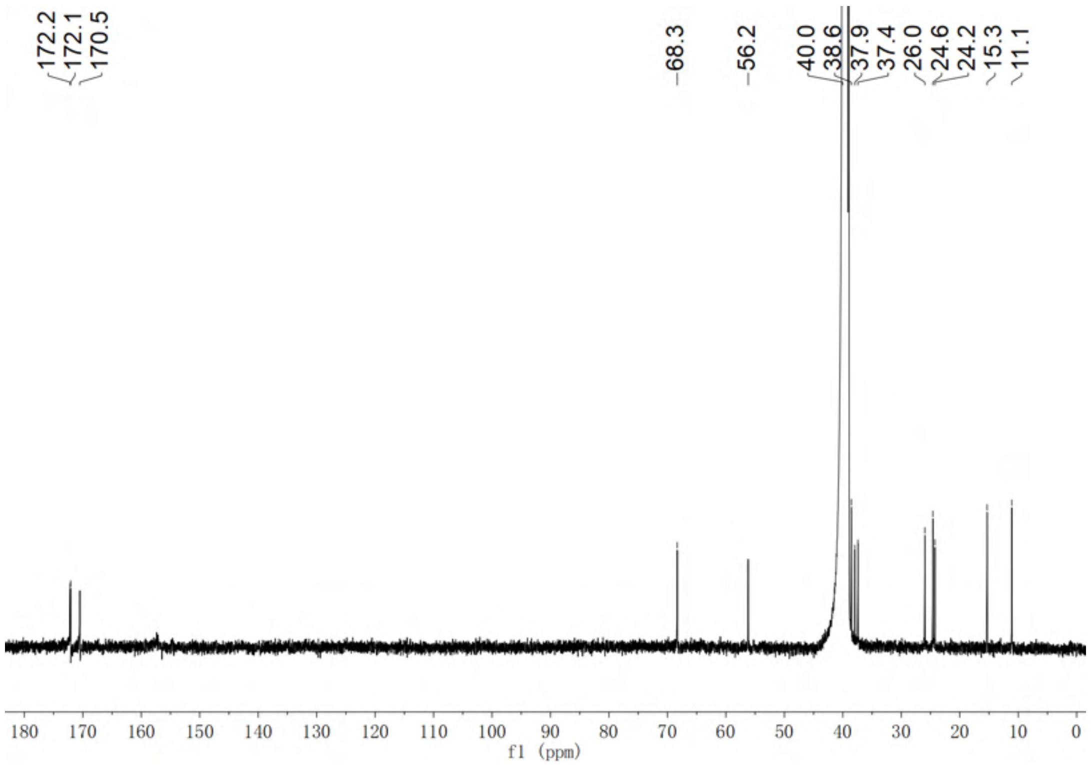

Supplementary Fig. 83. ^{13}C NMR spectrum of compound 12 in DMSO-d_6

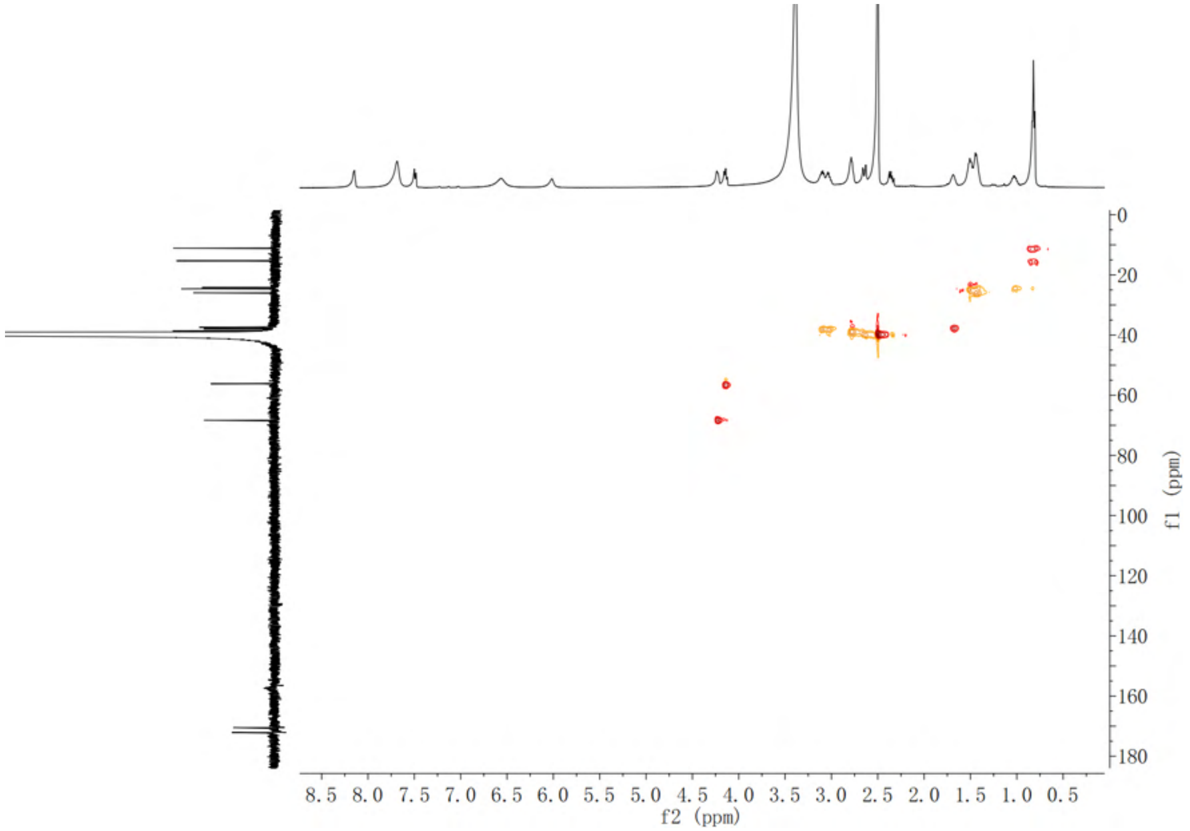

Supplementary Fig. 84. HSQC spectrum of compound 12 in DMSO-d_6

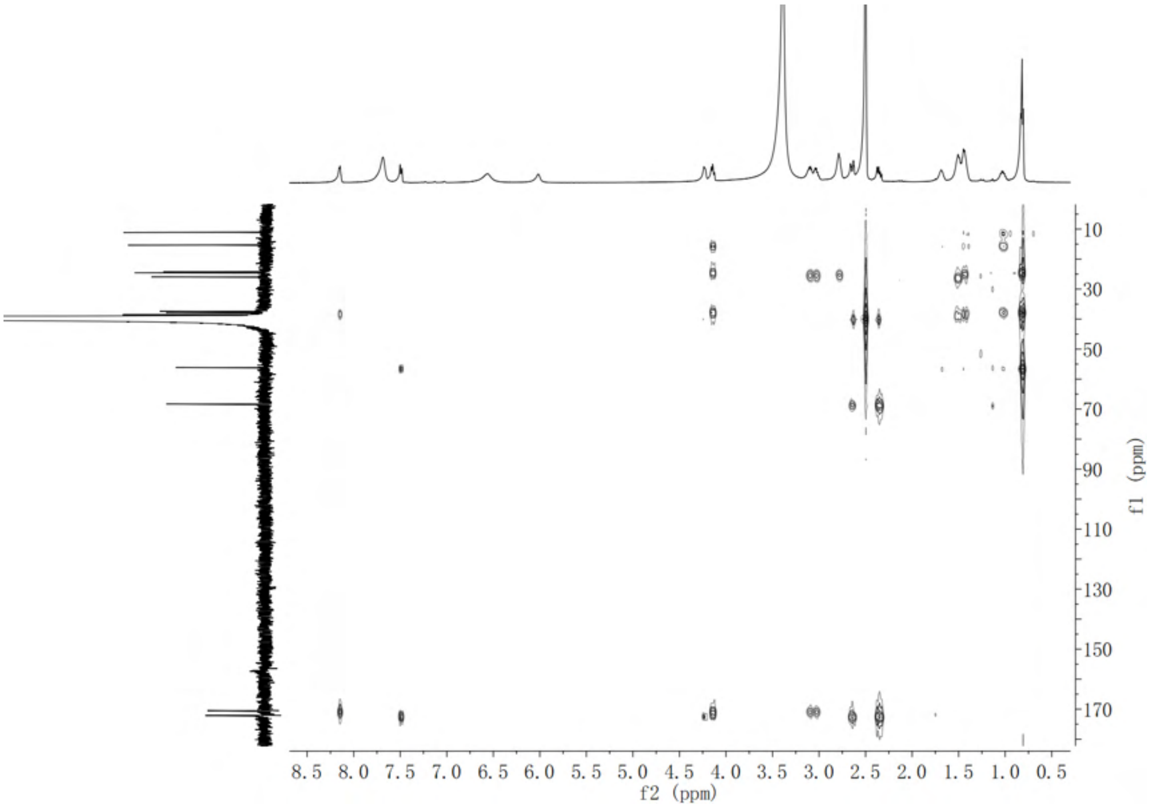

Supplementary Fig. 85. HMBC spectrum of compound 12 in DMSO-*d*$_6$

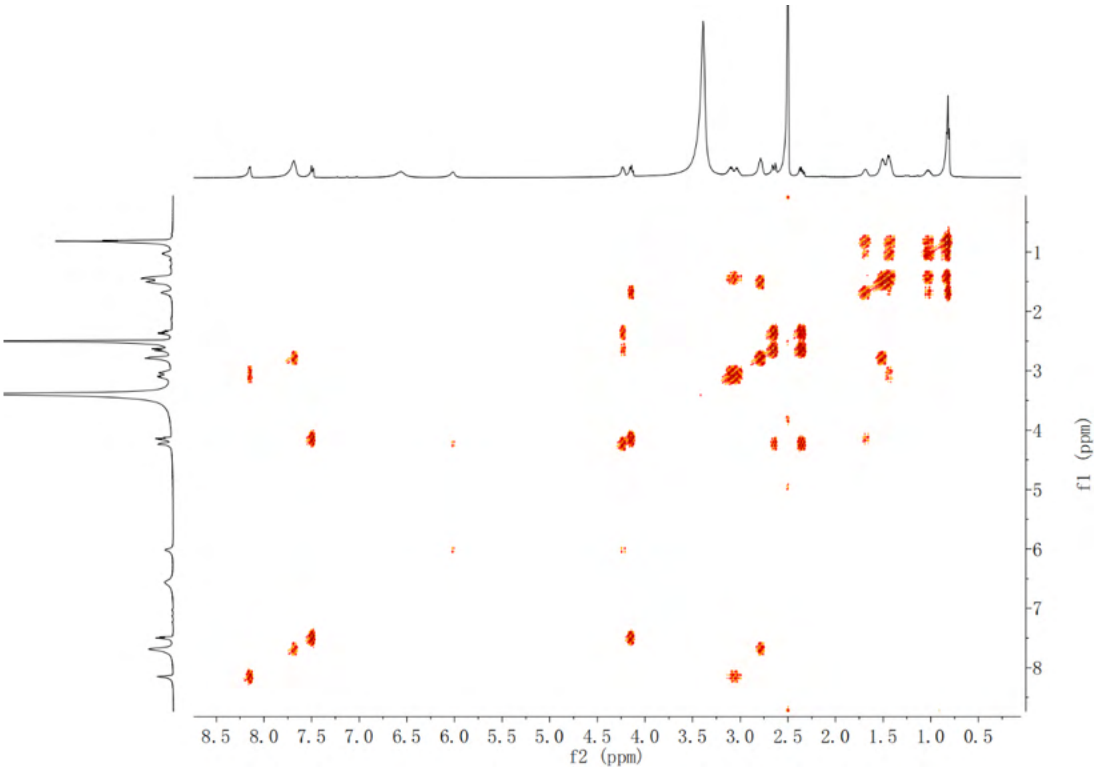

Supplementary Fig. 86. ^{1}H-^{1}H COSY spectrum of compound 12 in DMSO-*d*$_6$

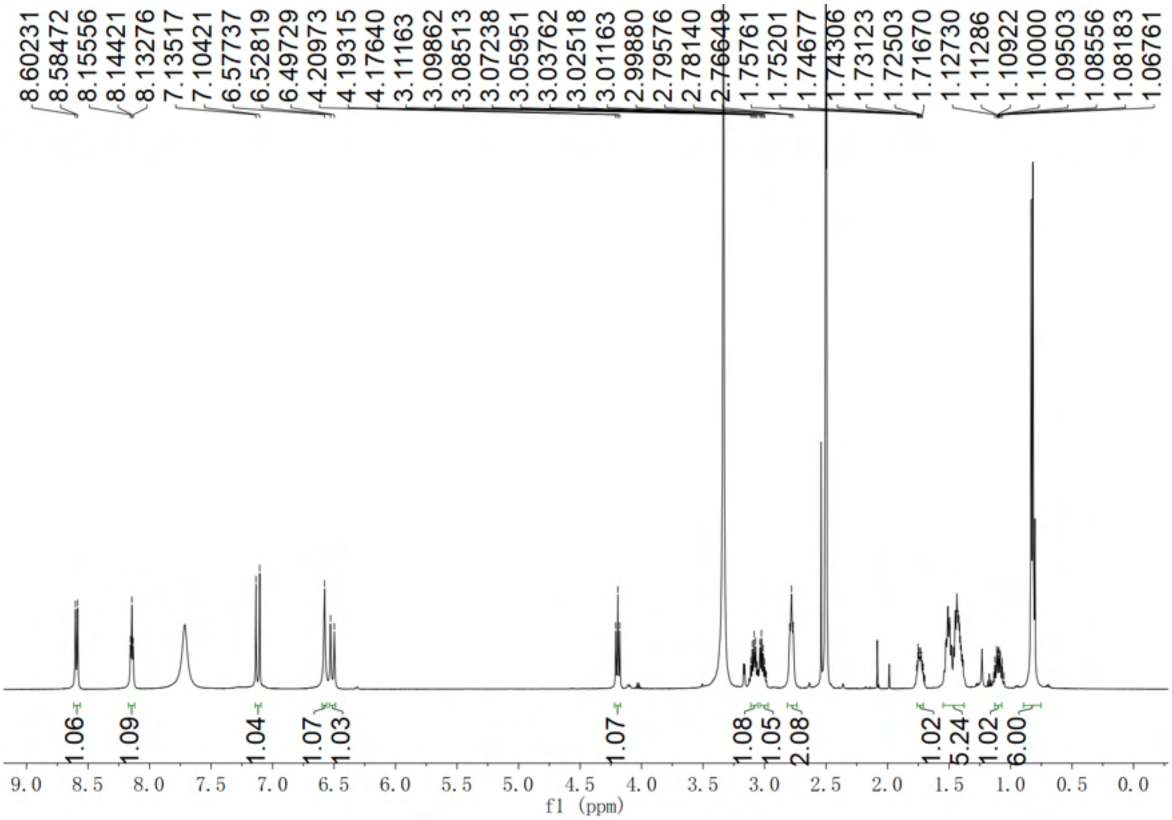

Supplementary Fig. 87. ^{1}H NMR spectrum of compound 13 in DMSO-d_6

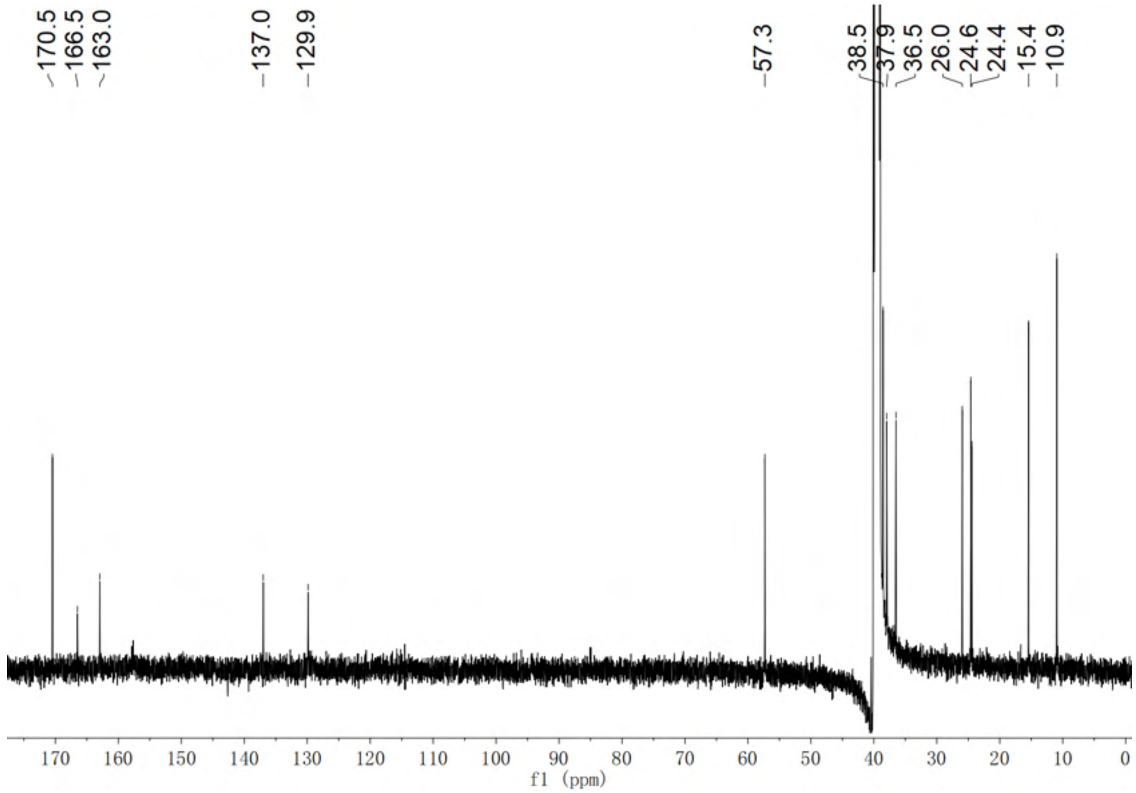

Supplementary Fig. 88. ^{13}C NMR spectrum of compound 13 in DMSO-d_6

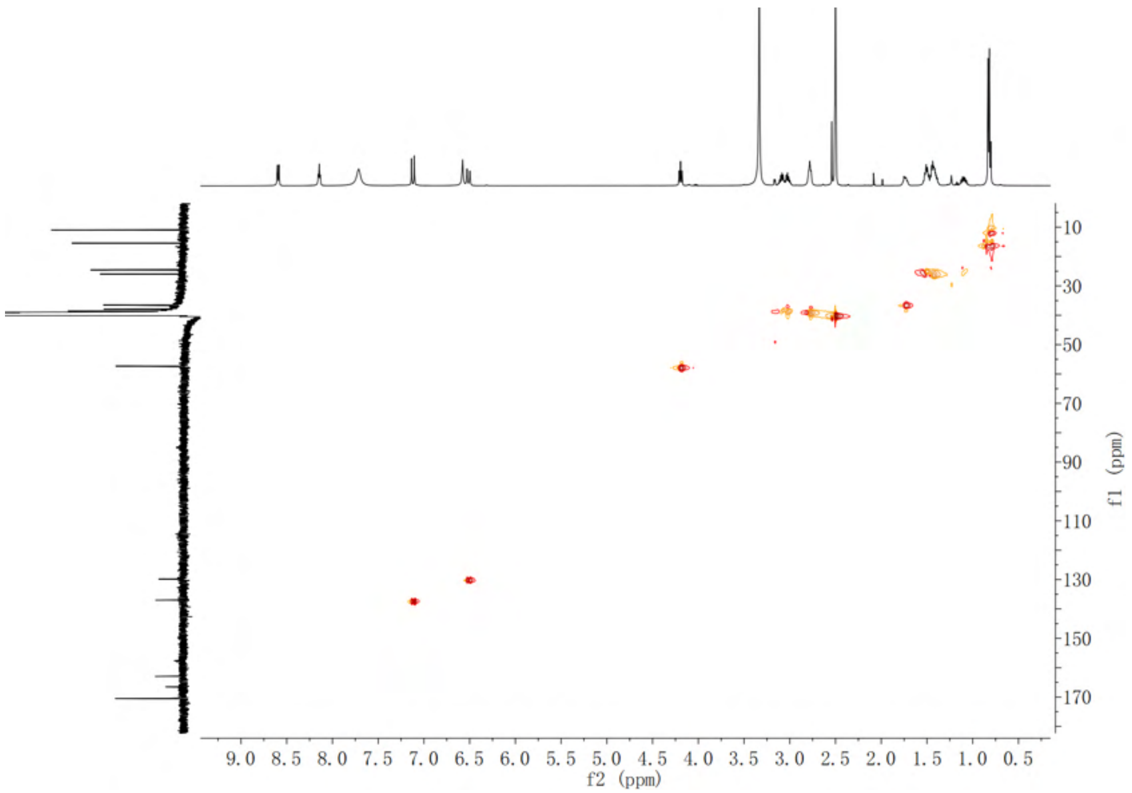

Supplementary Fig. 89. HSQC spectrum of compound 13 in DMSO-*d*₆

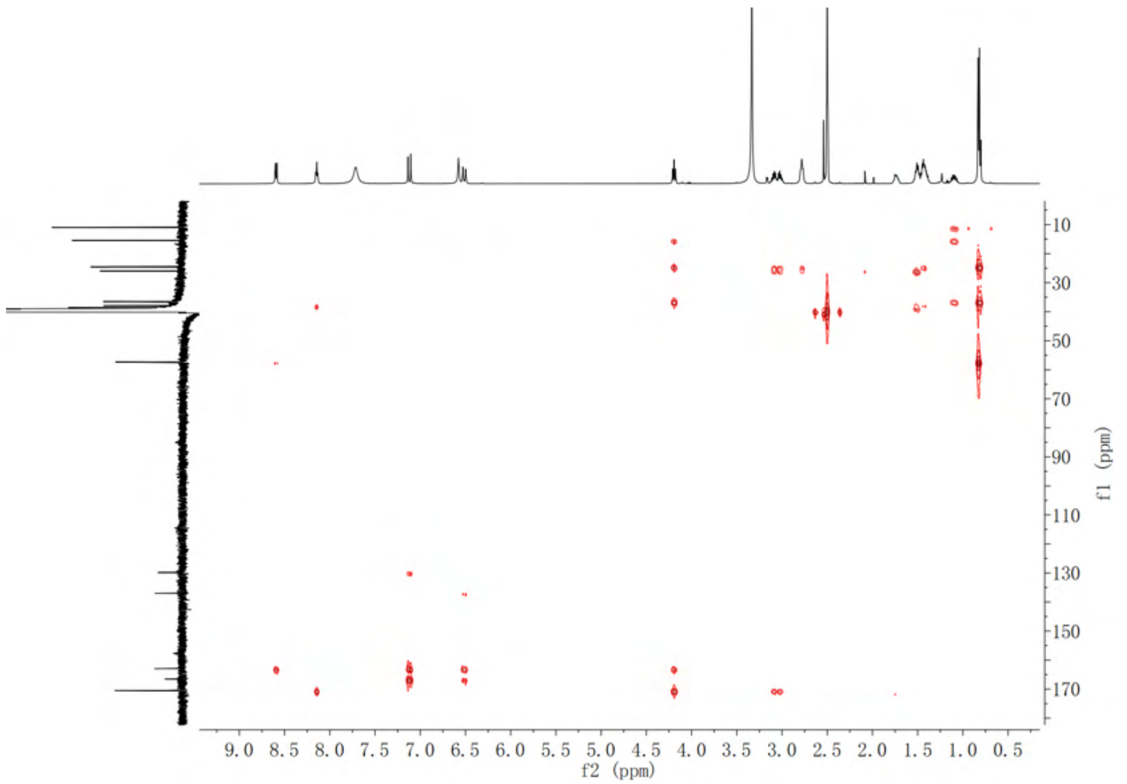

Supplementary Fig. 90. HMBC spectrum of compound 13 in DMSO-*d*₆

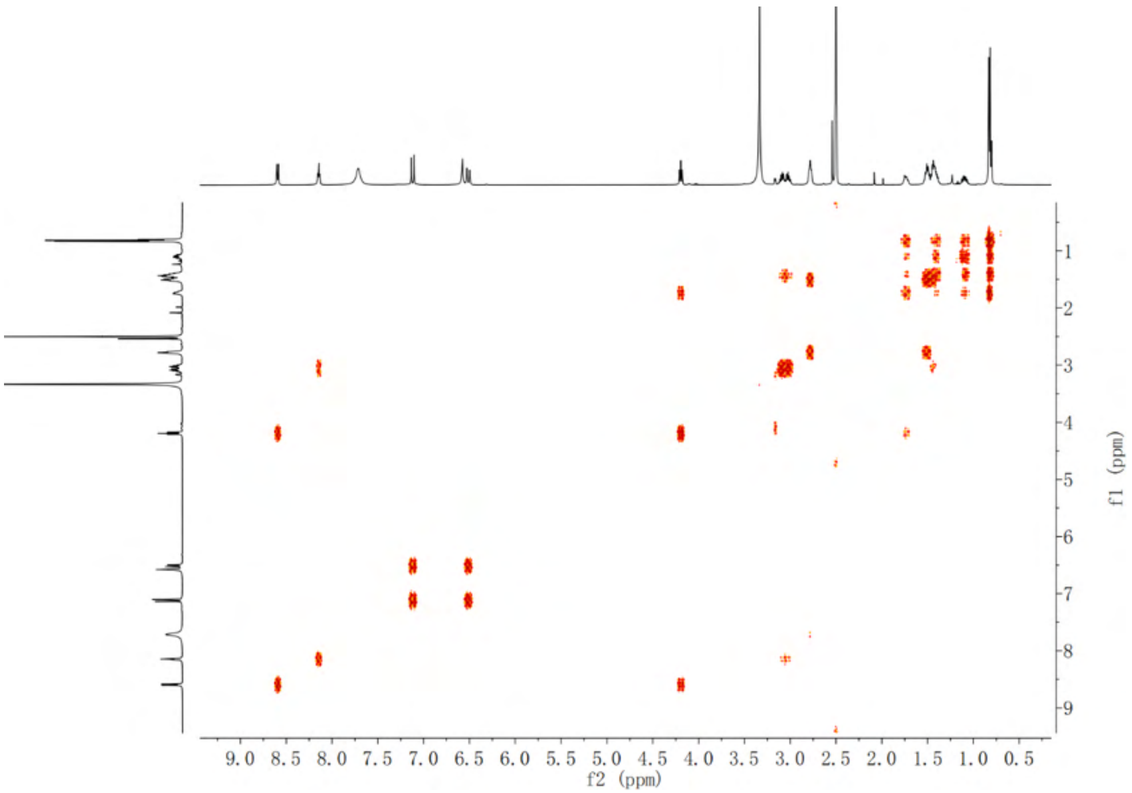

Supplementary Fig. 91. ^{1}H-^{1}H COSY spectrum of compound 13 in DMSO-d_6

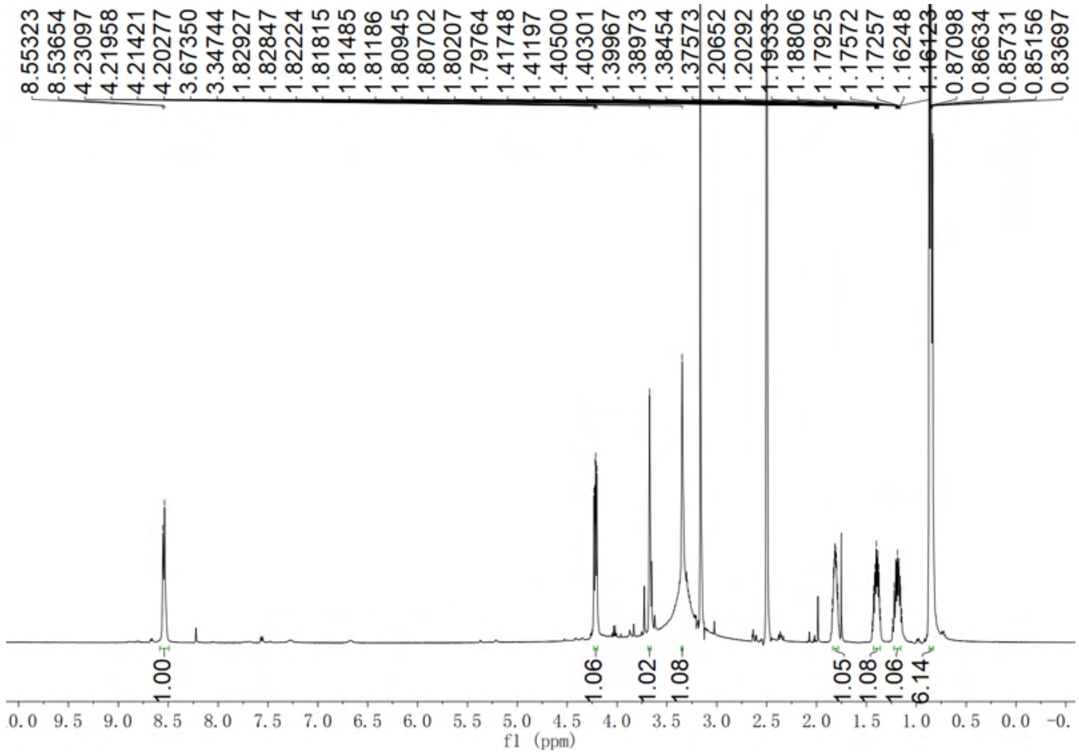

Supplementary Fig. 92. ^{1}H NMR spectrum of compound 14 in DMSO-d_6

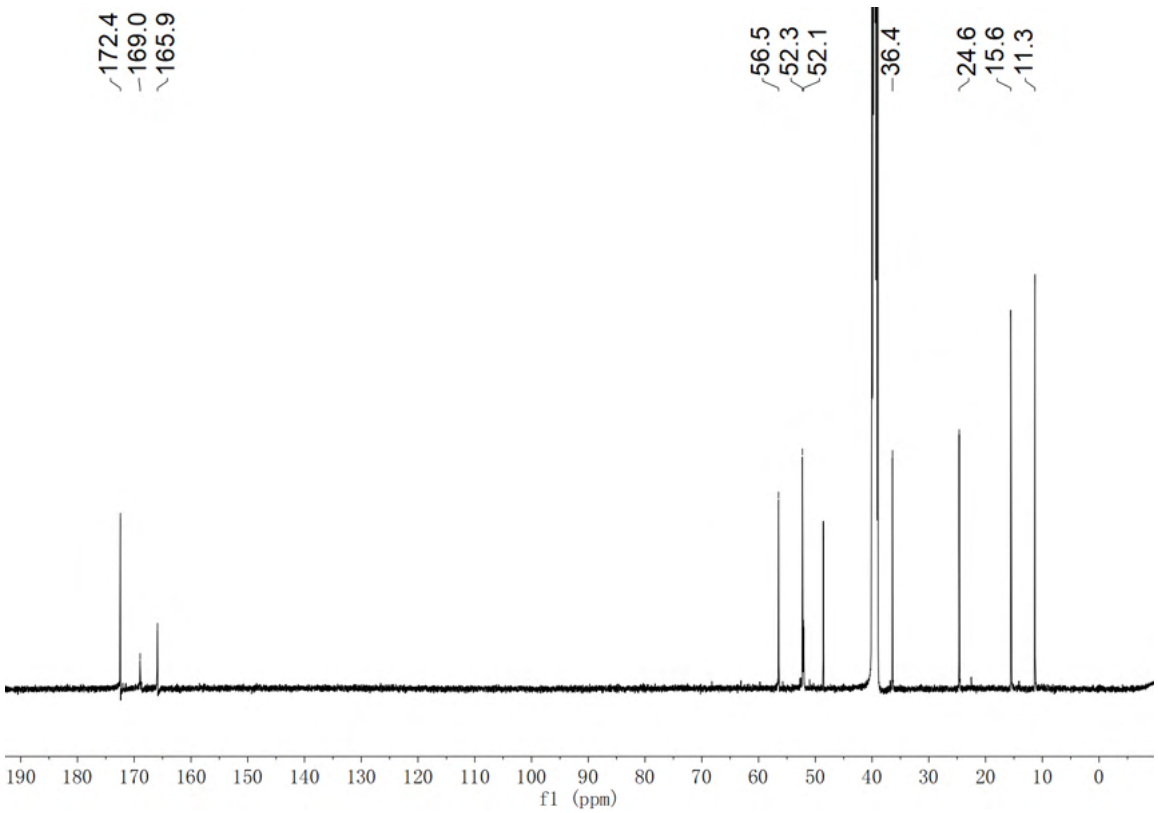

Supplementary Fig. 93. ^{13}C NMR spectrum of compound 14 in DMSO-*d*$_6$

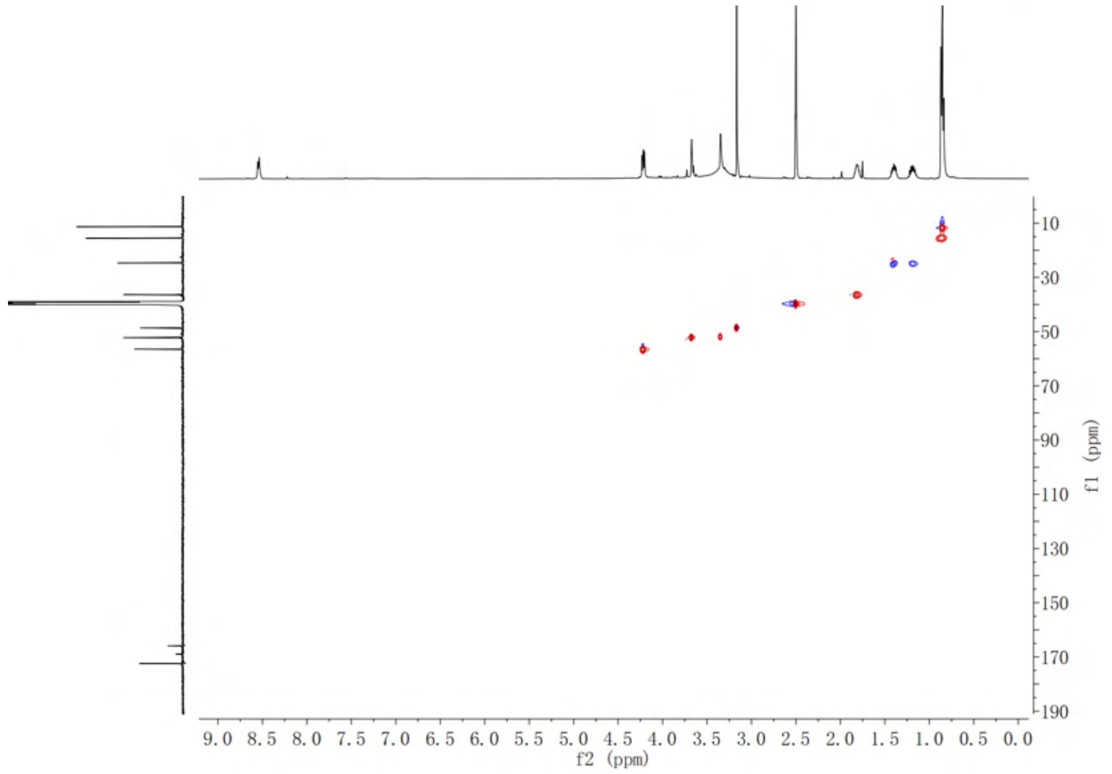

Supplementary Fig. 94. HSQC spectrum of compound 14 in DMSO-*d*$_6$

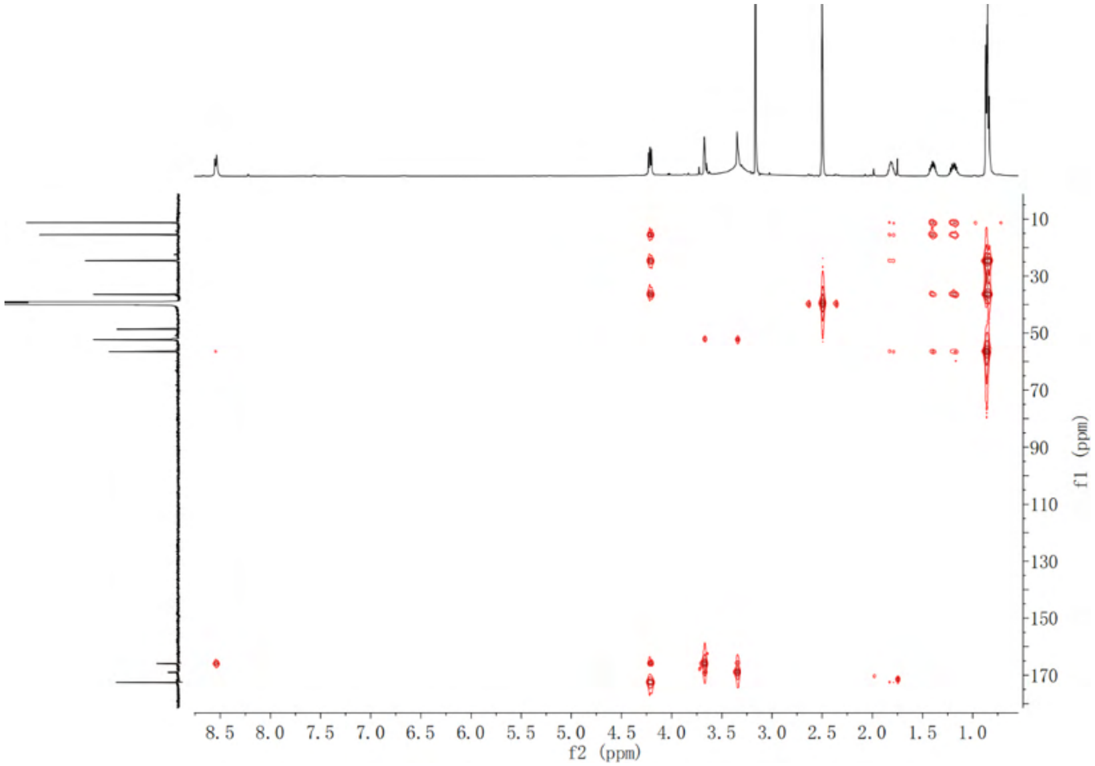

Supplementary Fig. 95. HMBC spectrum of compound 14 in DMSO-d_6

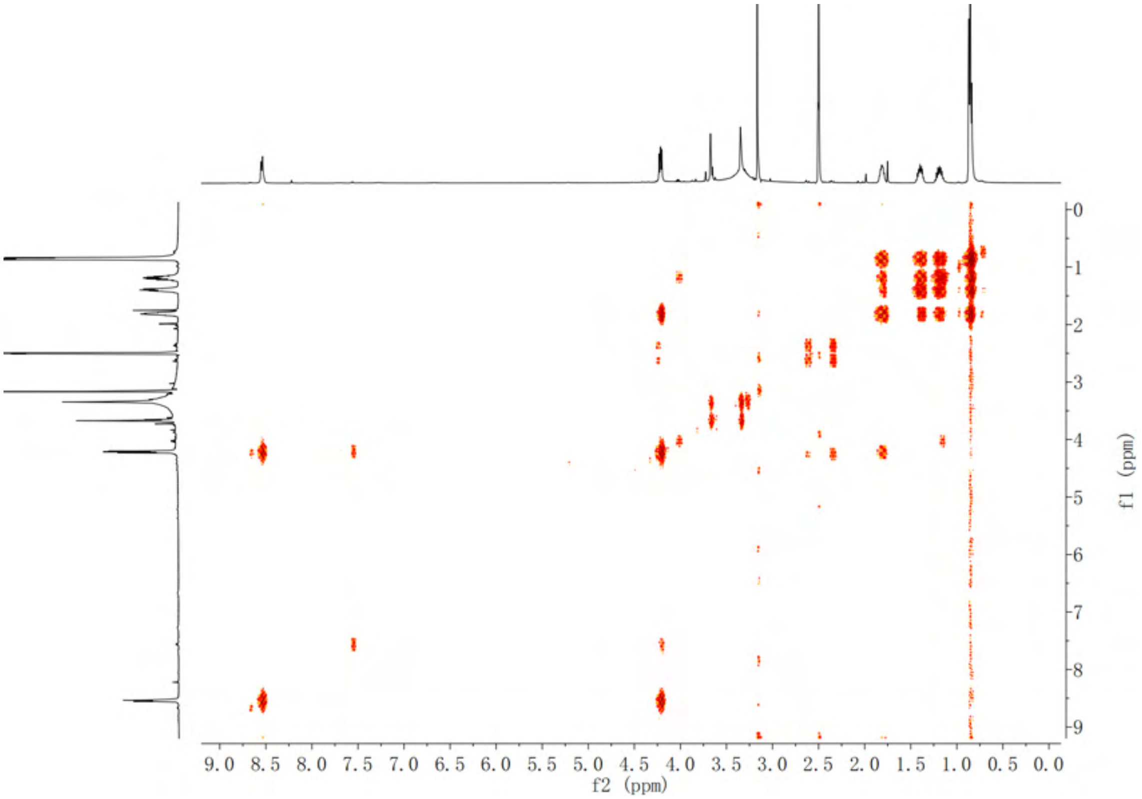

Supplementary Fig. 96. ^{1}H-^{1}H COSY spectrum of compound 14 in DMSO-d_6

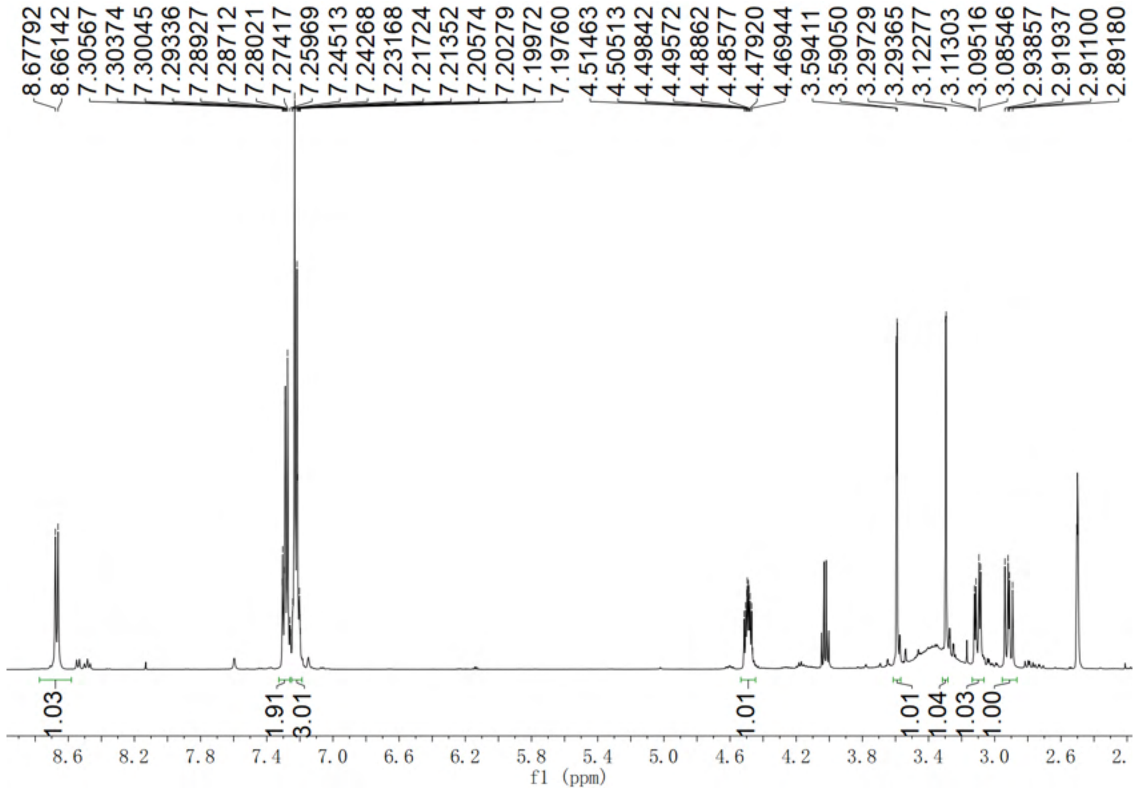

Supplementary Fig. 97. ^{1}H NMR spectrum of compound 15 in DMSO-d_6

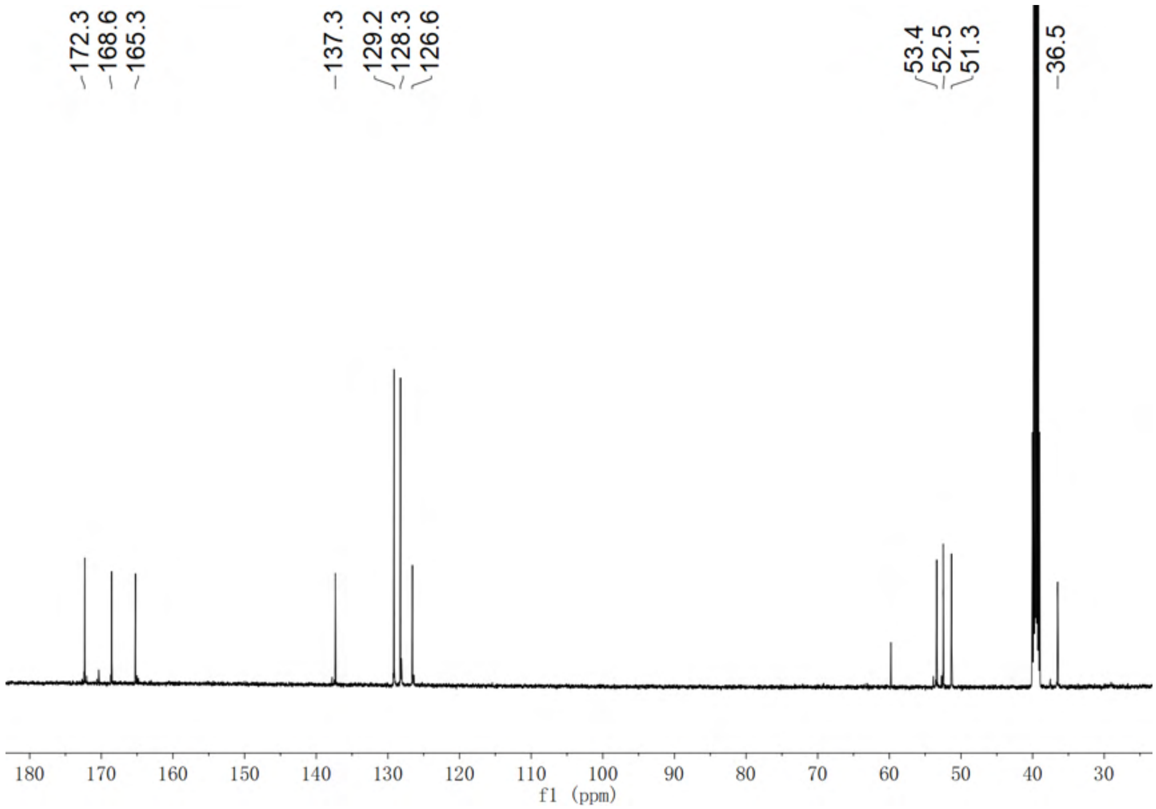

Supplementary Fig. 98. ^{13}C NMR spectrum of compound 15 in DMSO-d_6

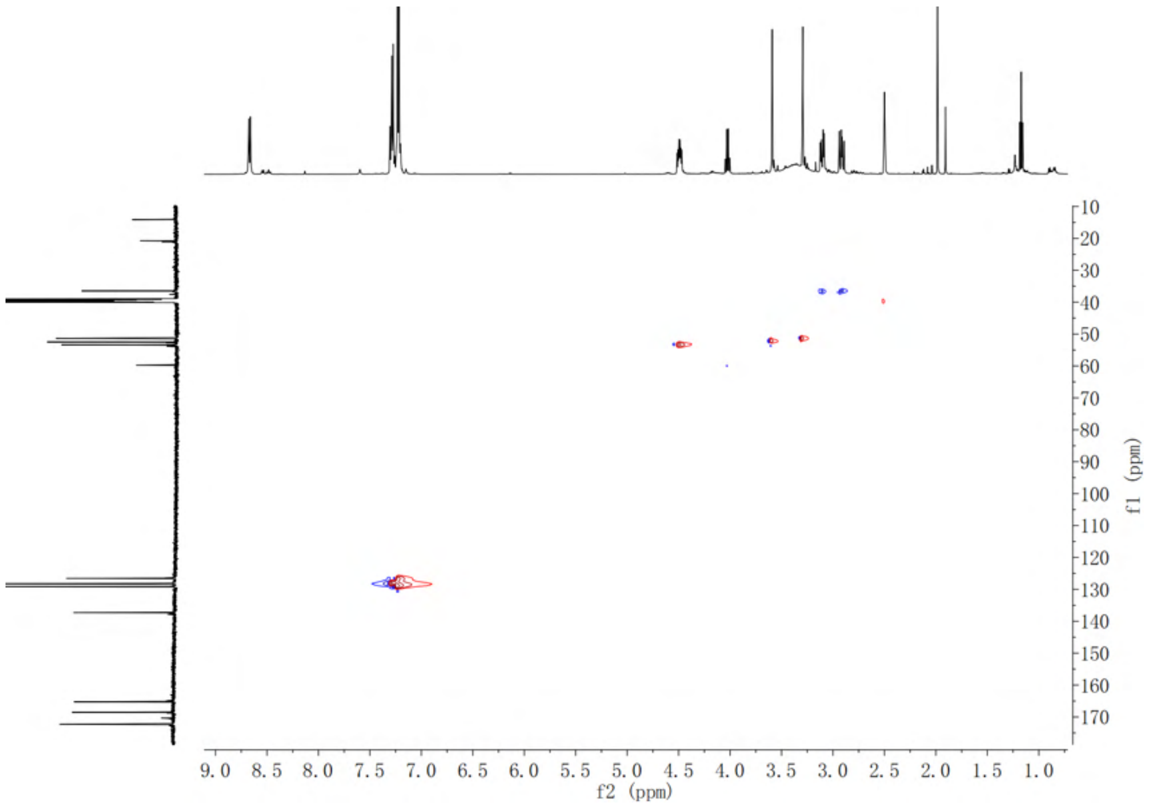

Supplementary Fig. 99. HSQC spectrum of compound 15 in DMSO-d_6

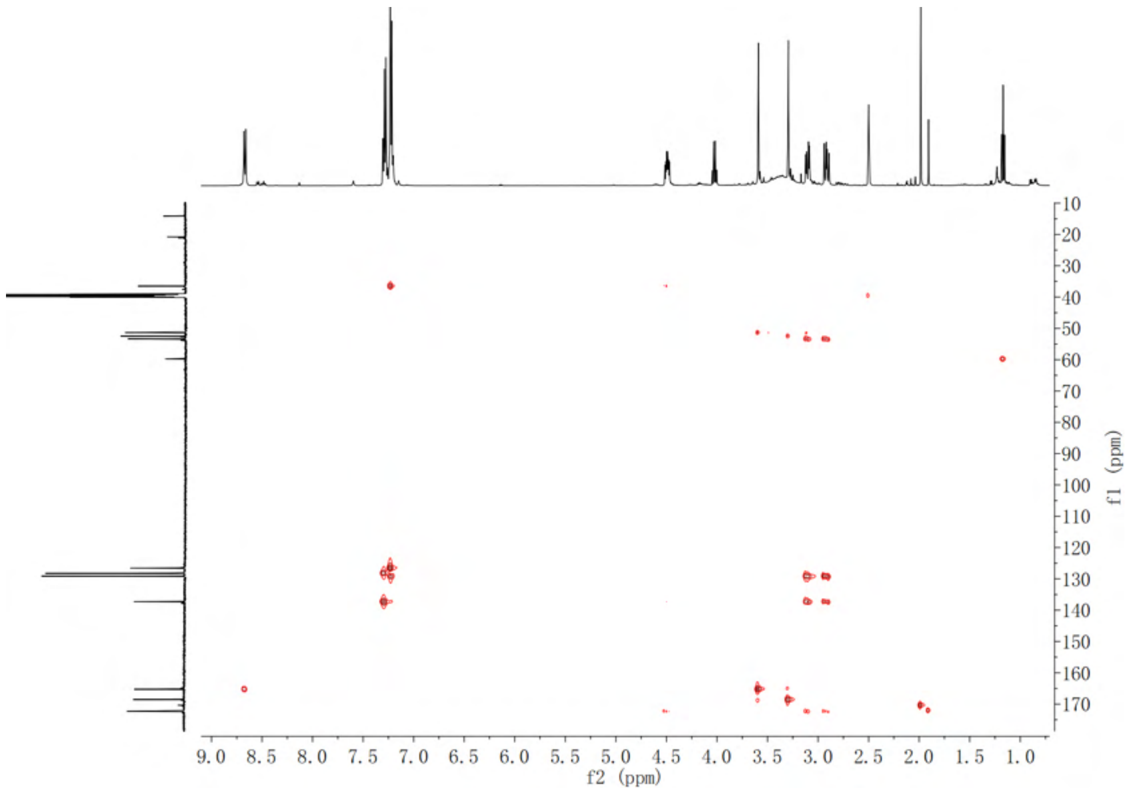

Supplementary Fig. 100. HMBC spectrum of compound 15 in DMSO-d_6

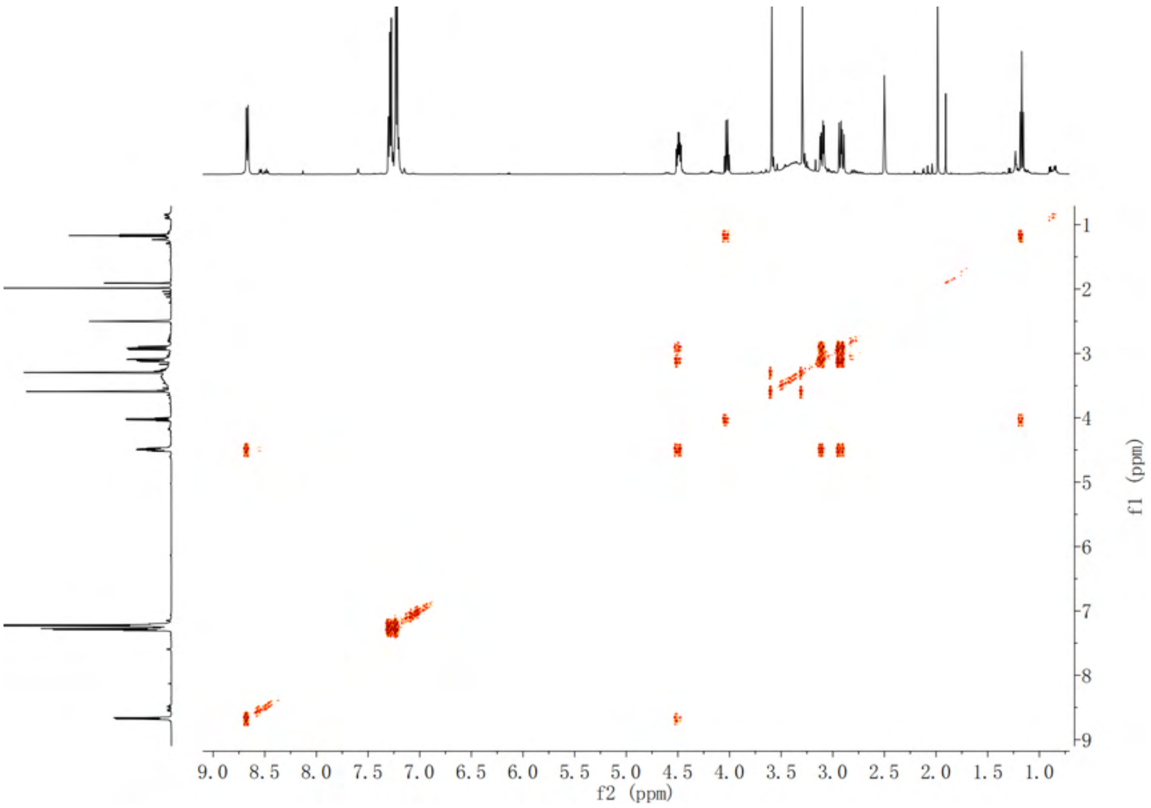

Supplementary Fig. 101. **^{1}H-^{1}H COSY spectrum of compound 15 in DMSO-d_6**

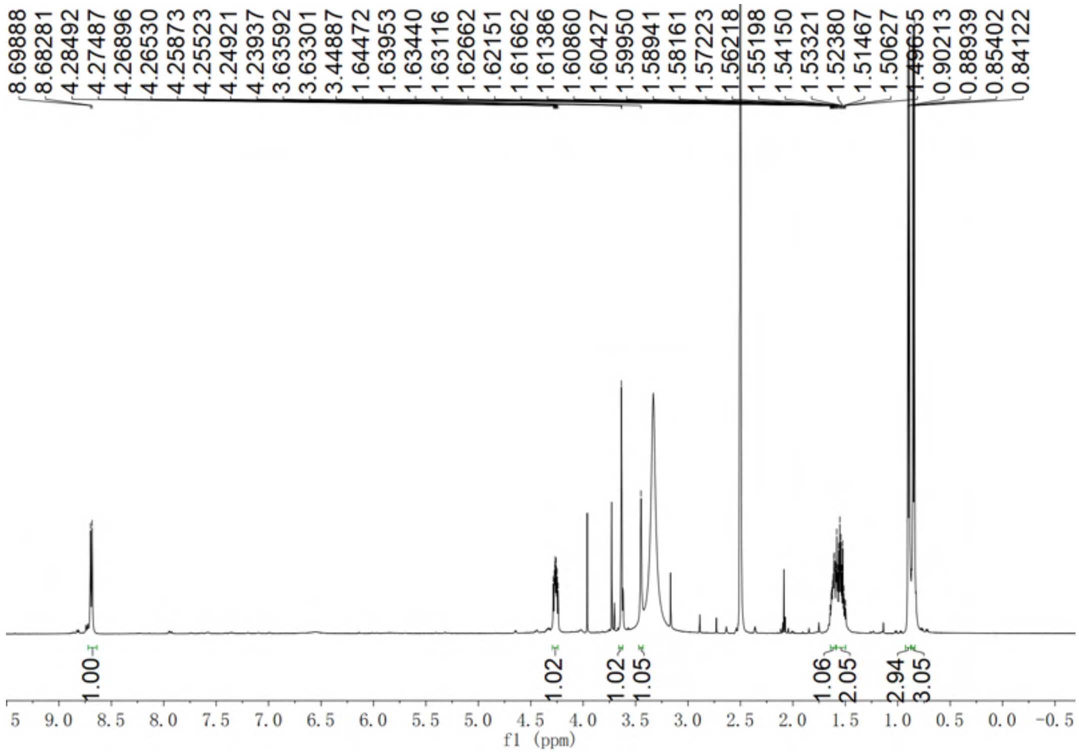

Supplementary Fig. 102. **^{1}H NMR spectrum of compound (2*S*,3*S*)-*t*-ES-Leu in DMSO-d_6**

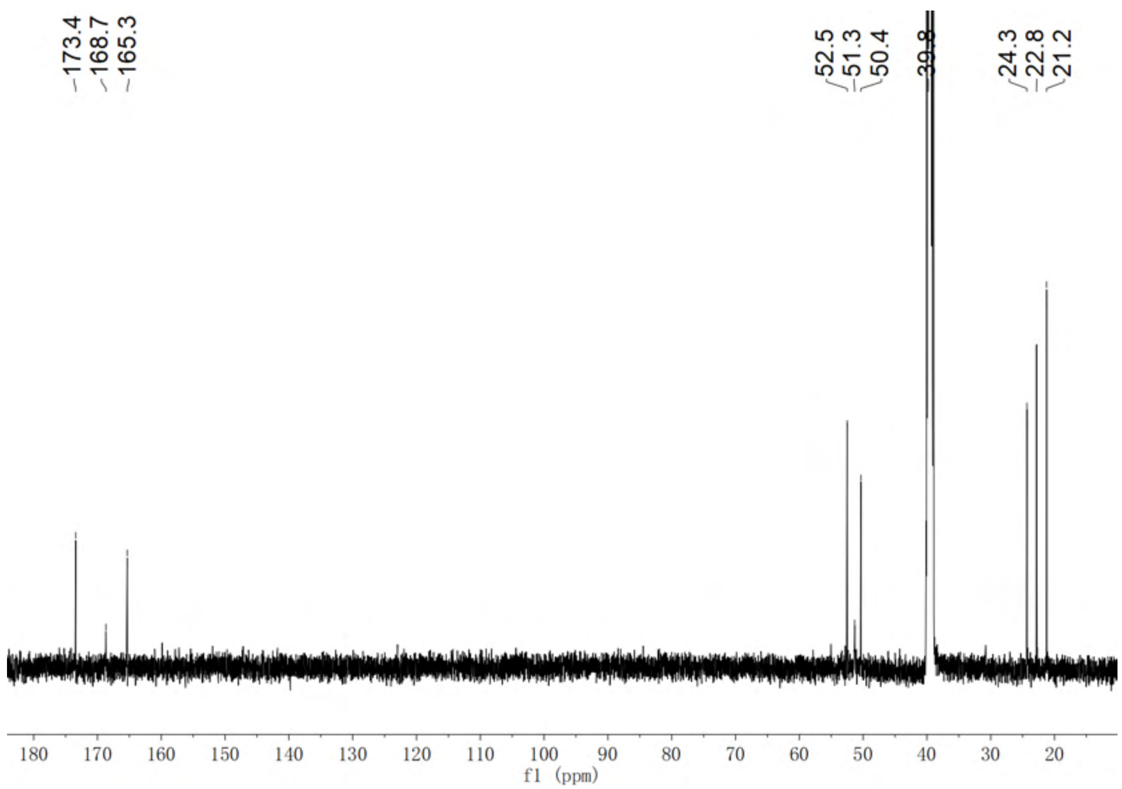

Supplementary Fig. 103. ^{13}C NMR spectrum of compound (2*S*,3*S*)-*t*-ES-Leu in DMSO-d_6

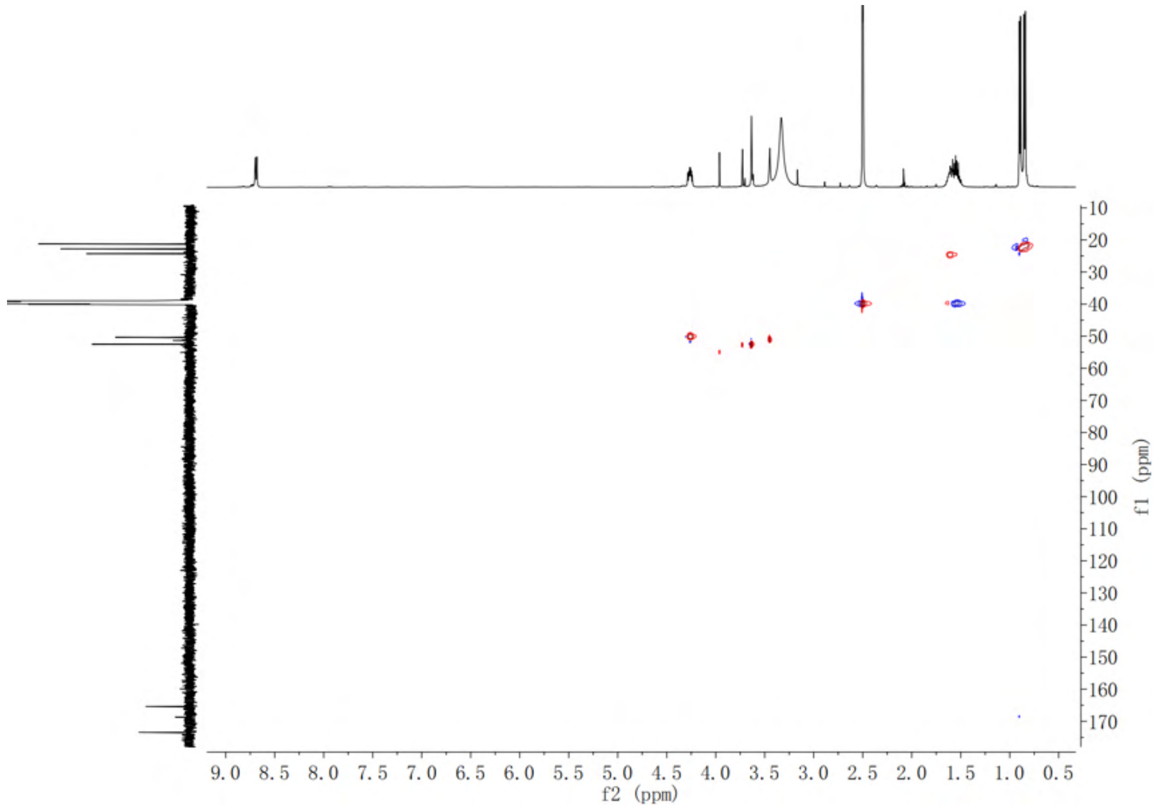

Supplementary Fig. 104. HSQC spectrum of compound (2*S*,3*S*)-*t*-ES-Leu in DMSO-d_6

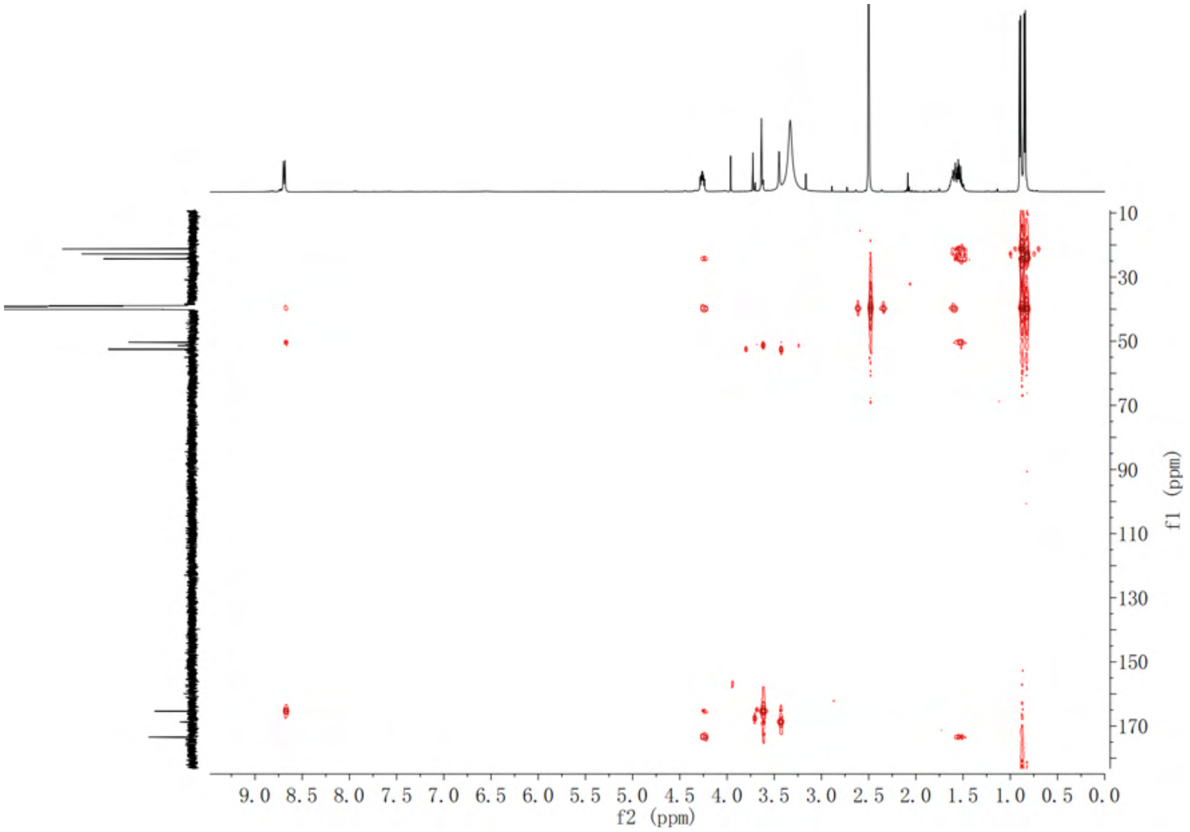

Supplementary Fig. 105. HMBC spectrum of compound (2*S*,3*S*)-*t*-ES-Leu in DMSO-*d*$_6$

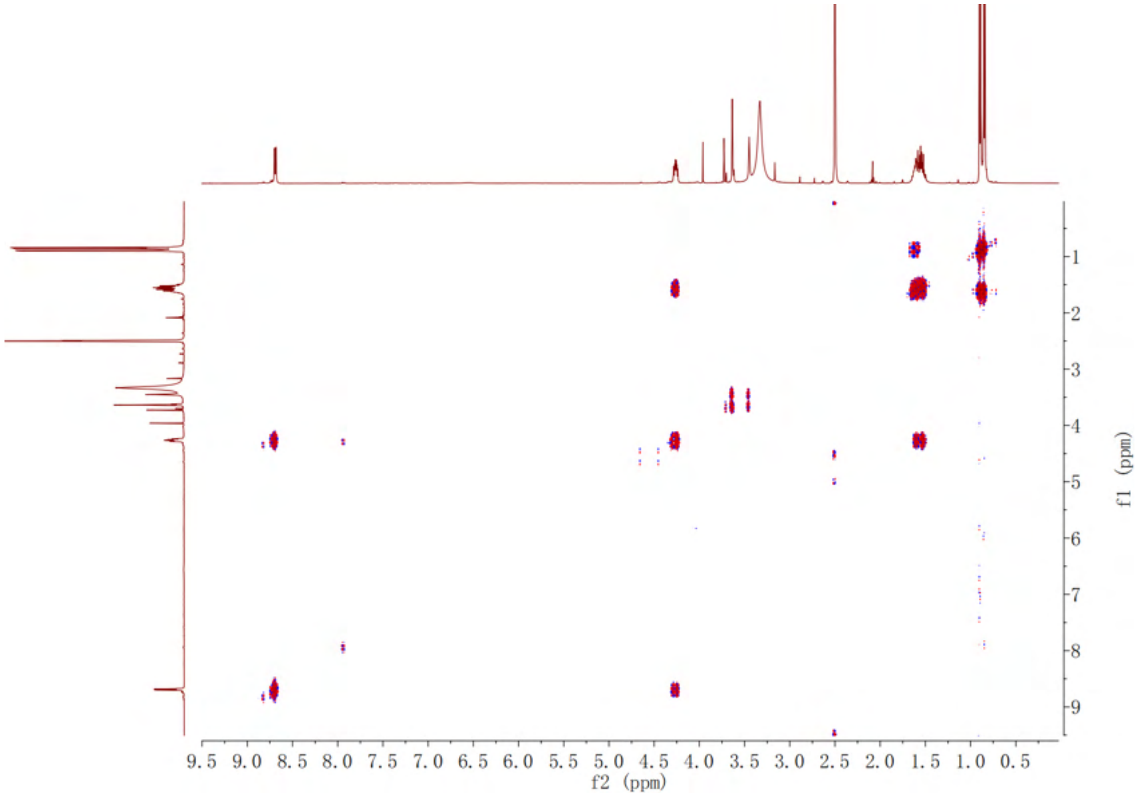

Supplementary Fig. 106. ^{1}H-^{1}H COSY spectrum of compound (2*S*,3*S*)-*t*-ES-Leu in DMSO-*d*$_6$

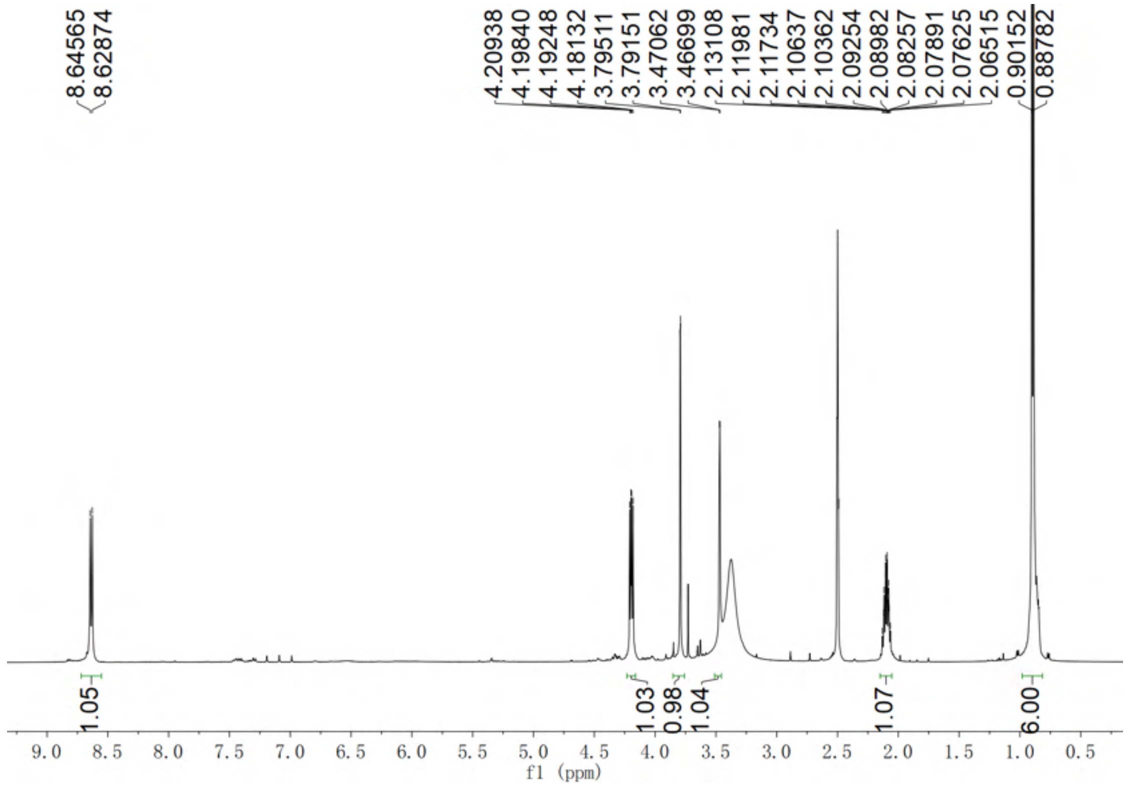

Supplementary Fig. 107. ^{1}H NMR spectrum of compound (2*S*,3*S*)-*t*-ES-Val in DMSO-d_6

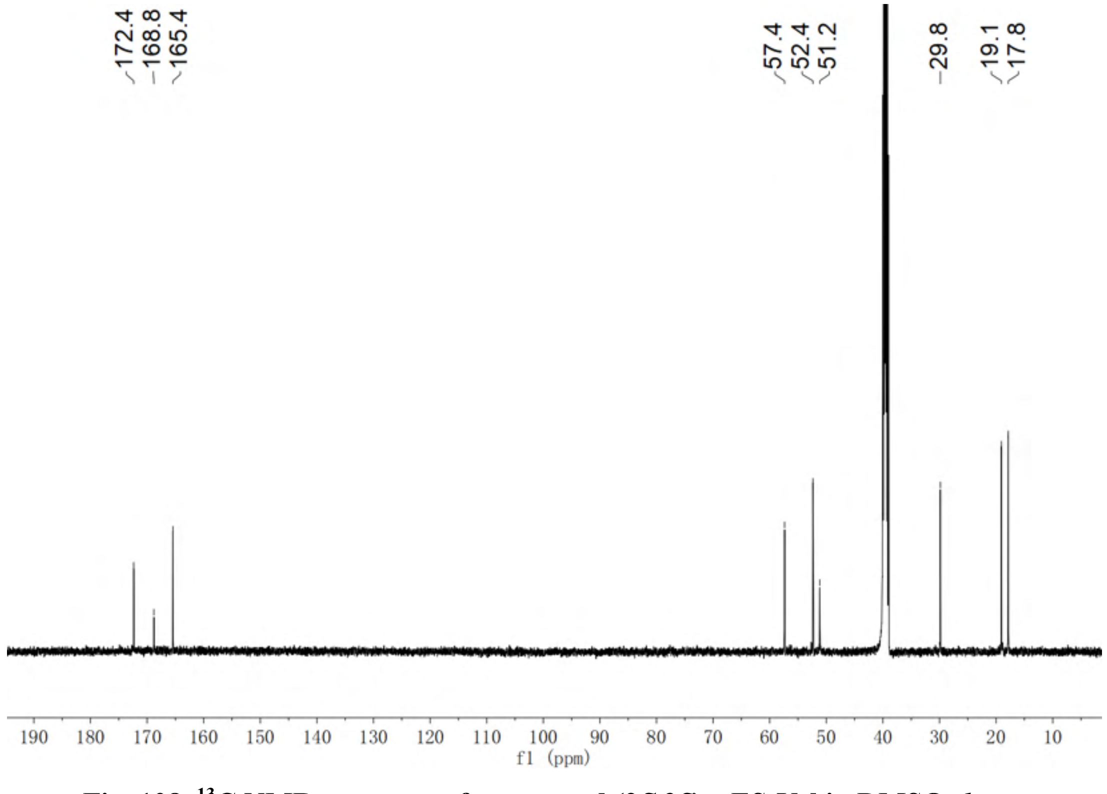

Supplementary Fig. 108. ^{13}C NMR spectrum of compound (2*S*,3*S*)-*t*-ES-Val in DMSO-d_6

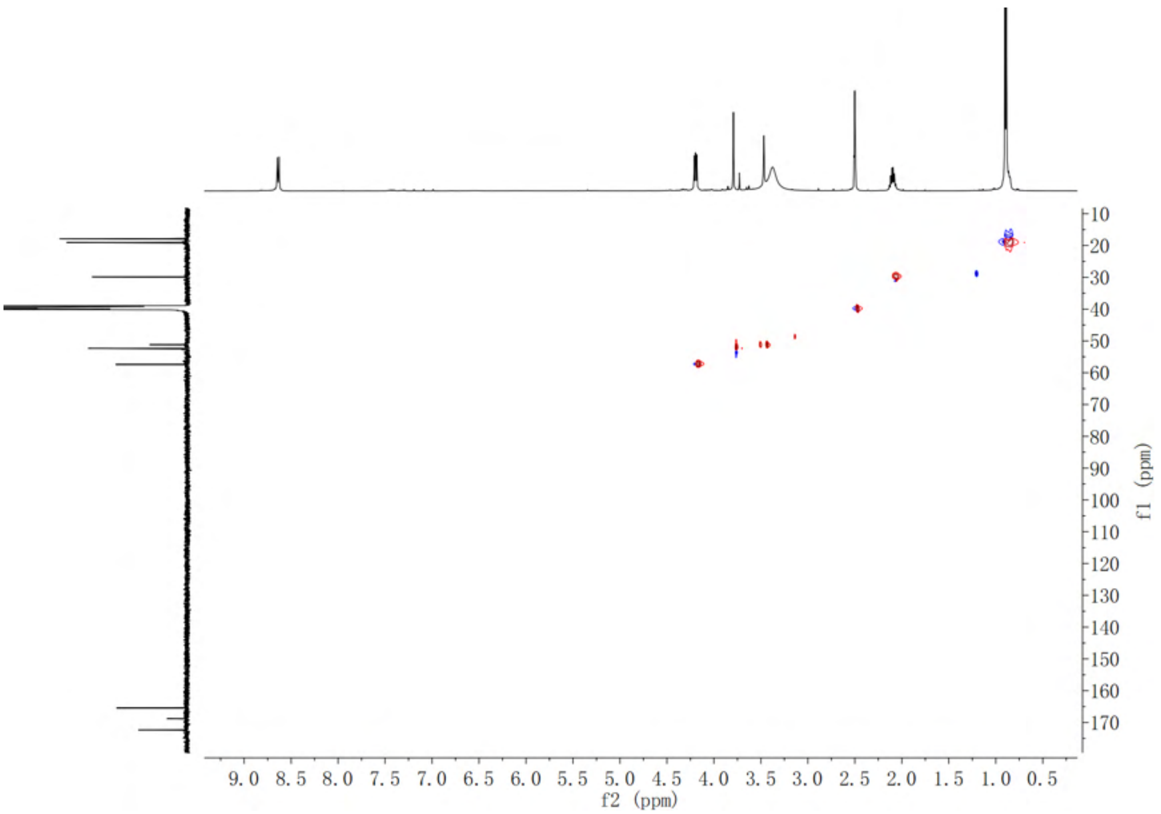

Supplementary Fig. 109. HSQC spectrum of compound (2*S*,3*S*)-*t*-ES-Val in DMSO-d_6

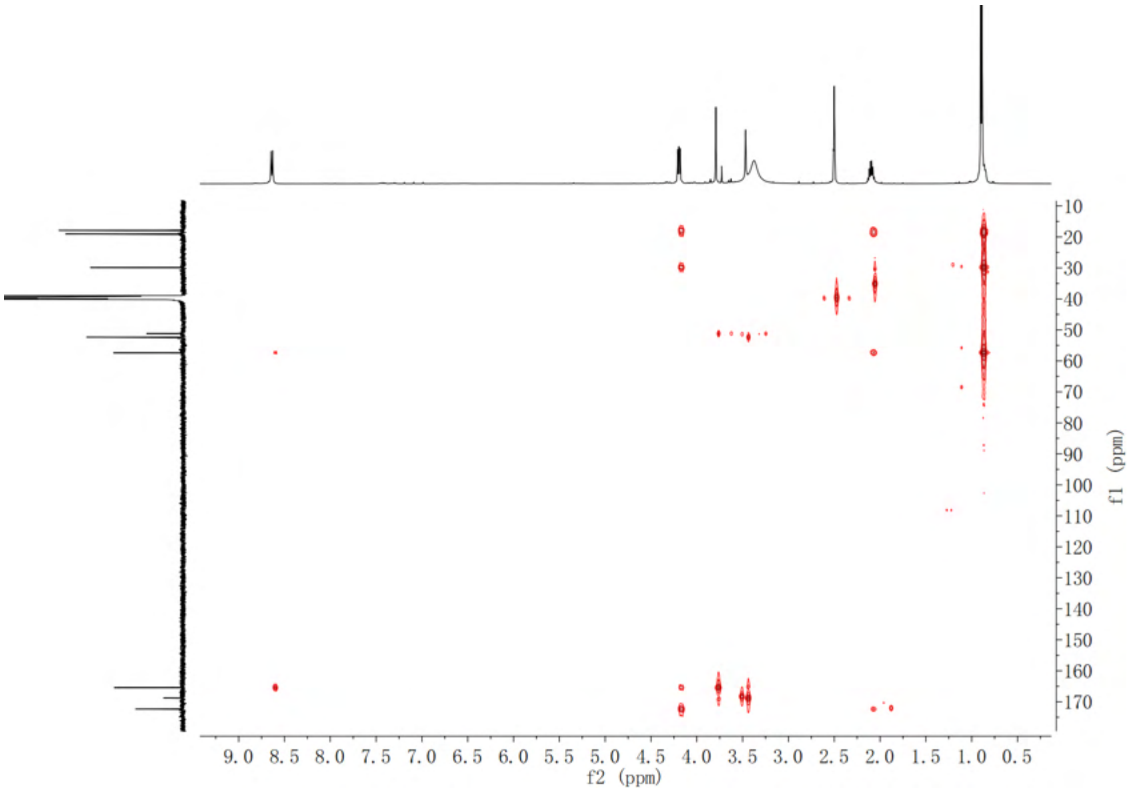

Supplementary Fig. 110. HMBC spectrum of compound (2*S*,3*S*)-*t*-ES-Val in DMSO-d_6

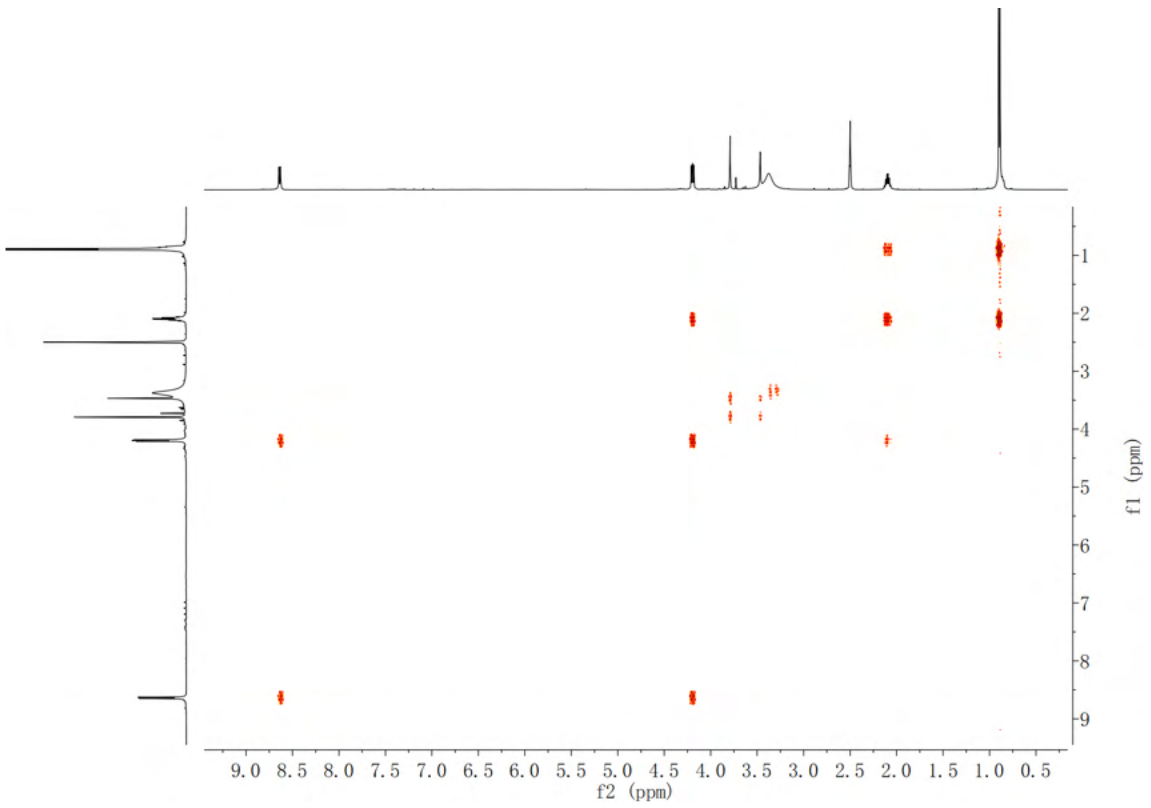

Supplementary Fig. 111. ^{1}H-^{1}H COSY spectrum of compound (2*S*,3*S*)-*t*-ES-Val in DMSO-*d*$_6$

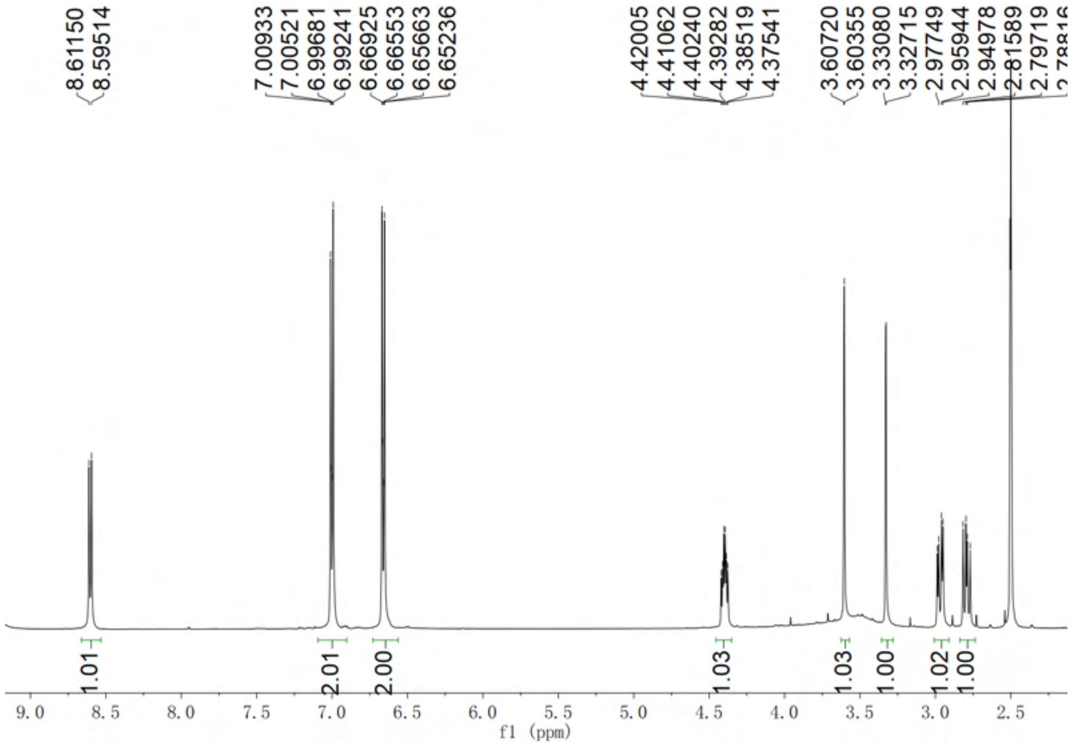

Supplementary Fig. 112. ^{1}H NMR spectrum of compound (2*S*,3*S*)-*t*-ES-Tyr in DMSO-*d*$_6$

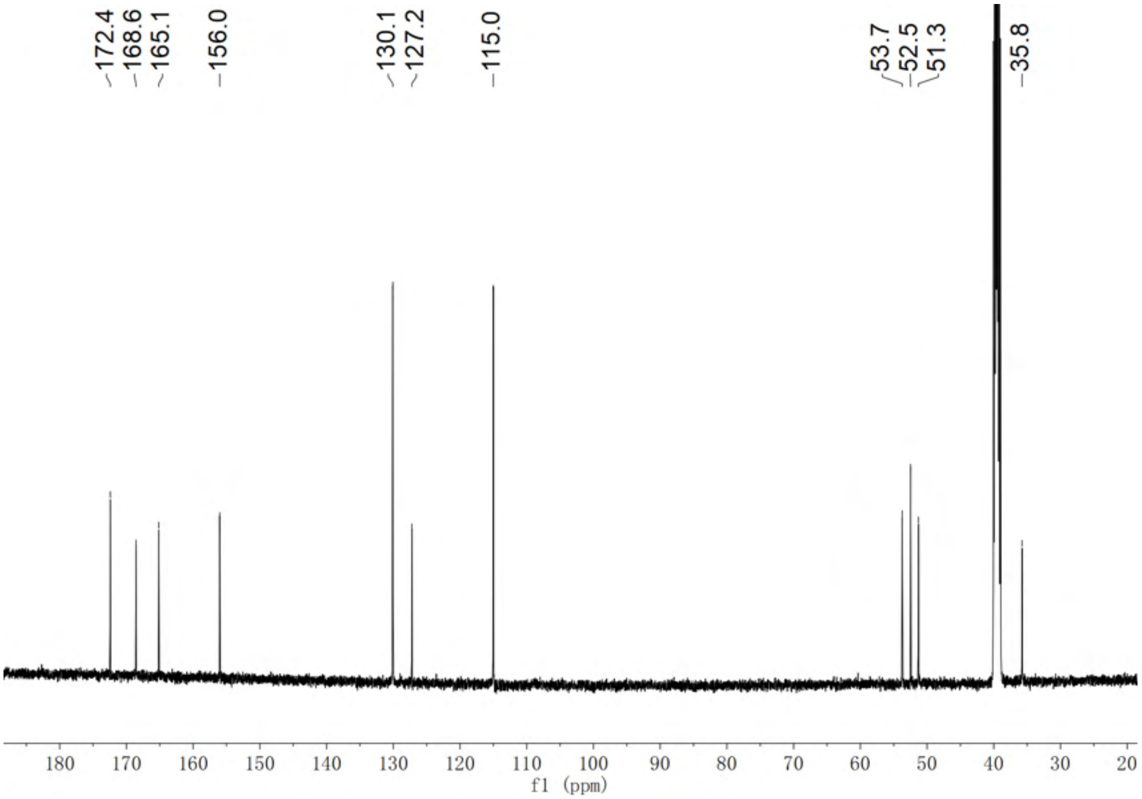

Supplementary Fig. 113. ^{13}**C NMR spectrum of compound (2*S*,3*S*)-*t*-ES-Tyr in DMSO-*d*$_6$**

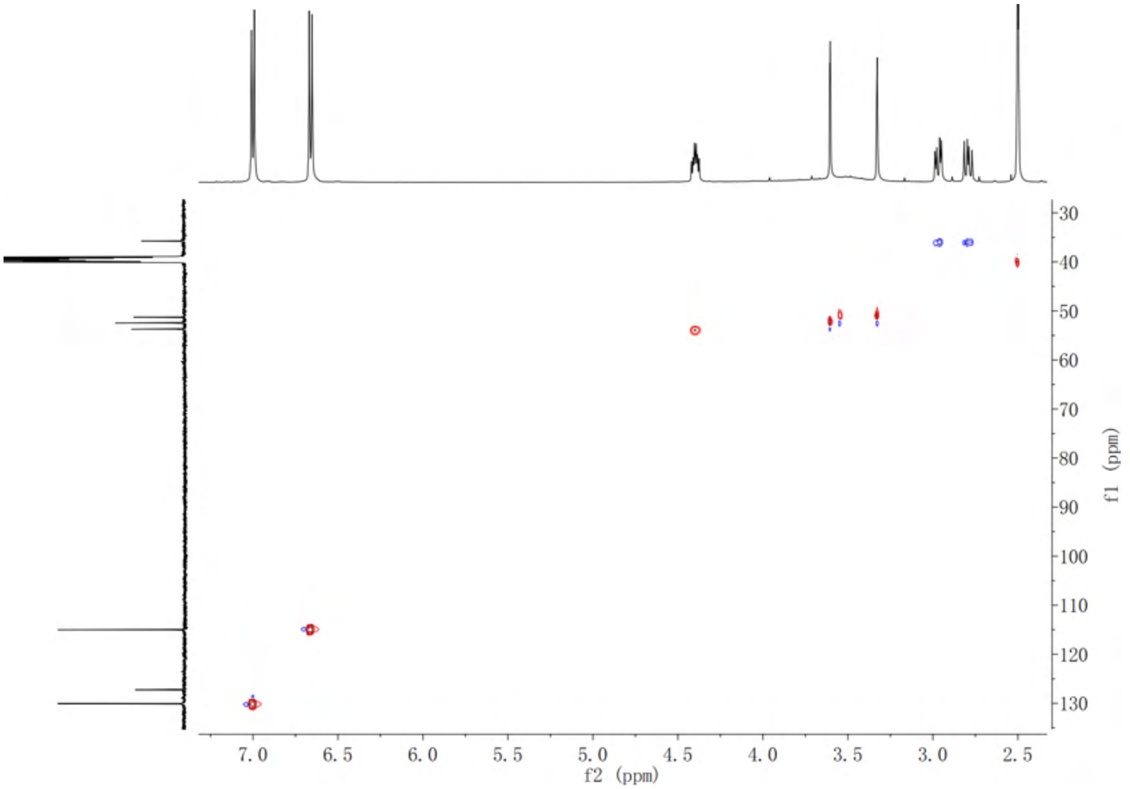

Supplementary Fig. 114. HSQC spectrum of compound (2*S*,3*S*)-*t*-ES-Tyr in DMSO-*d*$_6$

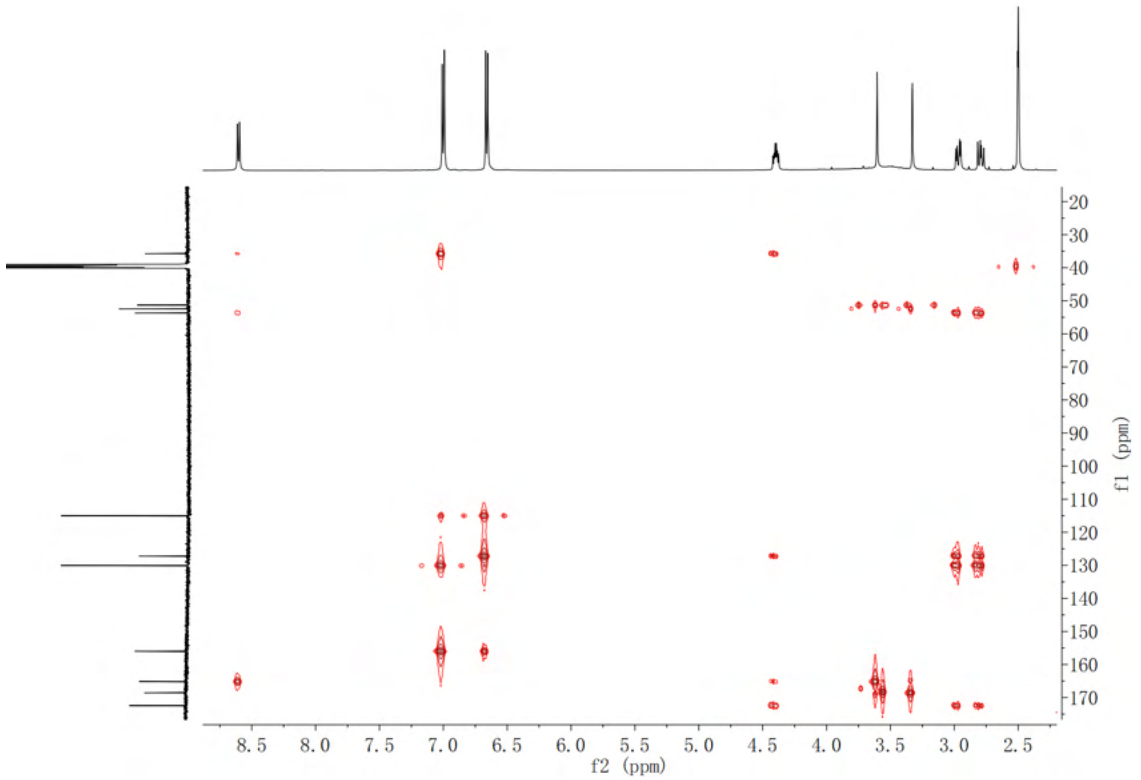

Supplementary Fig. 115. HMBC spectrum of compound (2*S*,3*S*)-*t*-ES-Tyr in DMSO-d_6

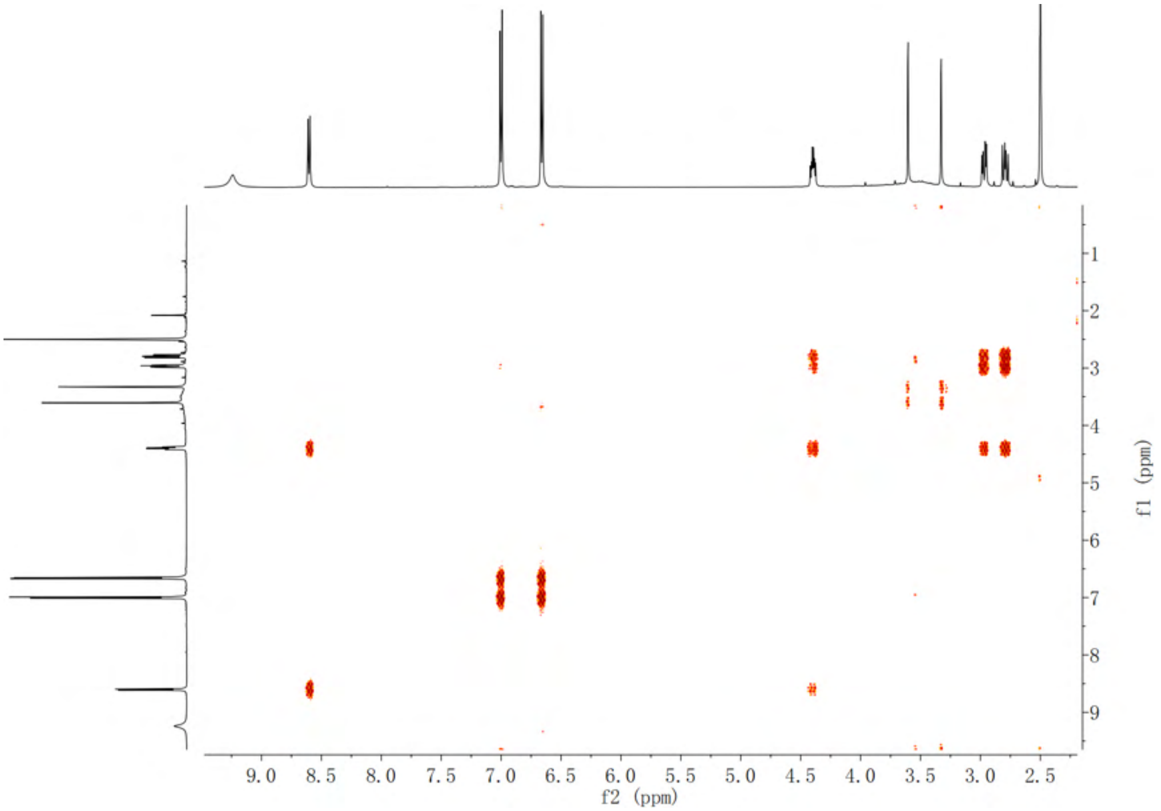

Supplementary Fig. 116. ^{1}H-^{1}H COSY spectrum of compound (2*S*,3*S*)-*t*-ES-Tyr in DMSO-d_6

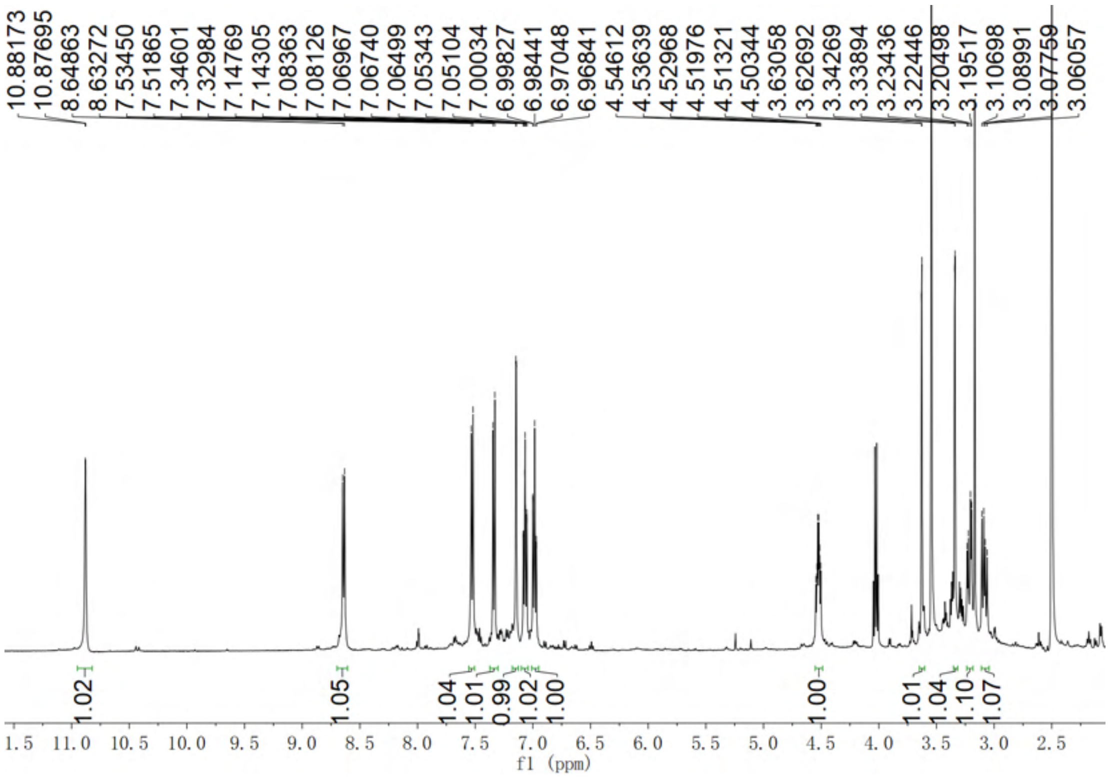

Supplementary Fig. 117. ^{1}H NMR spectrum of compound (2*S*,3*S*)-*t*-ES-Trp in DMSO-*d*$_6$

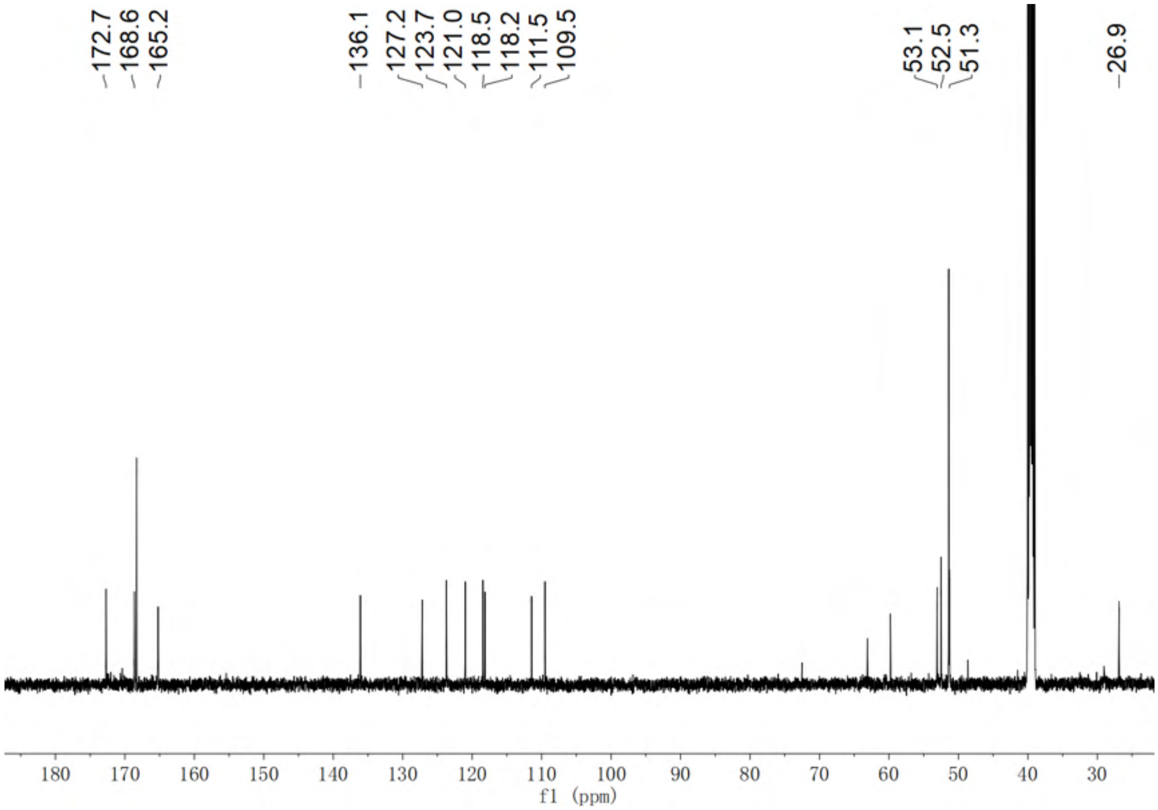

Supplementary Fig. 118. ^{13}C NMR spectrum of compound (2*S*,3*S*)-*t*-ES-Trp in DMSO-*d*$_6$

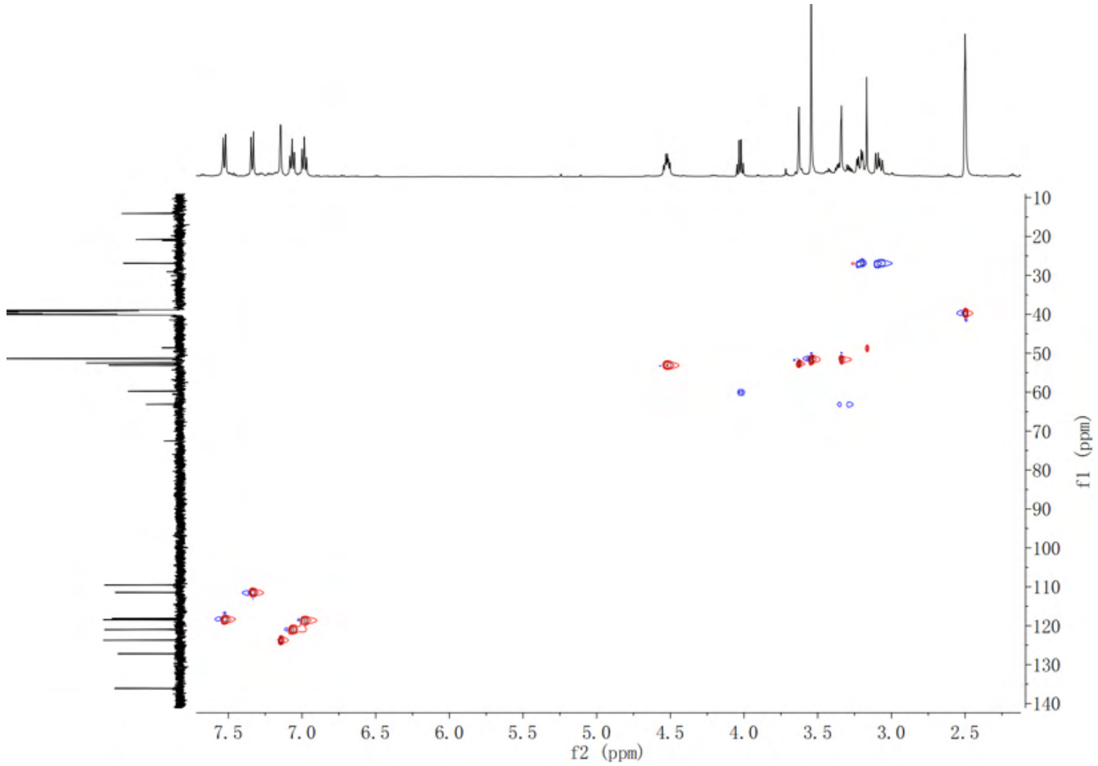

Supplementary Fig. 119. HSQC spectrum of compound (2*S*,3*S*)-*t*-ES-Trp in DMSO-*d*$_6$

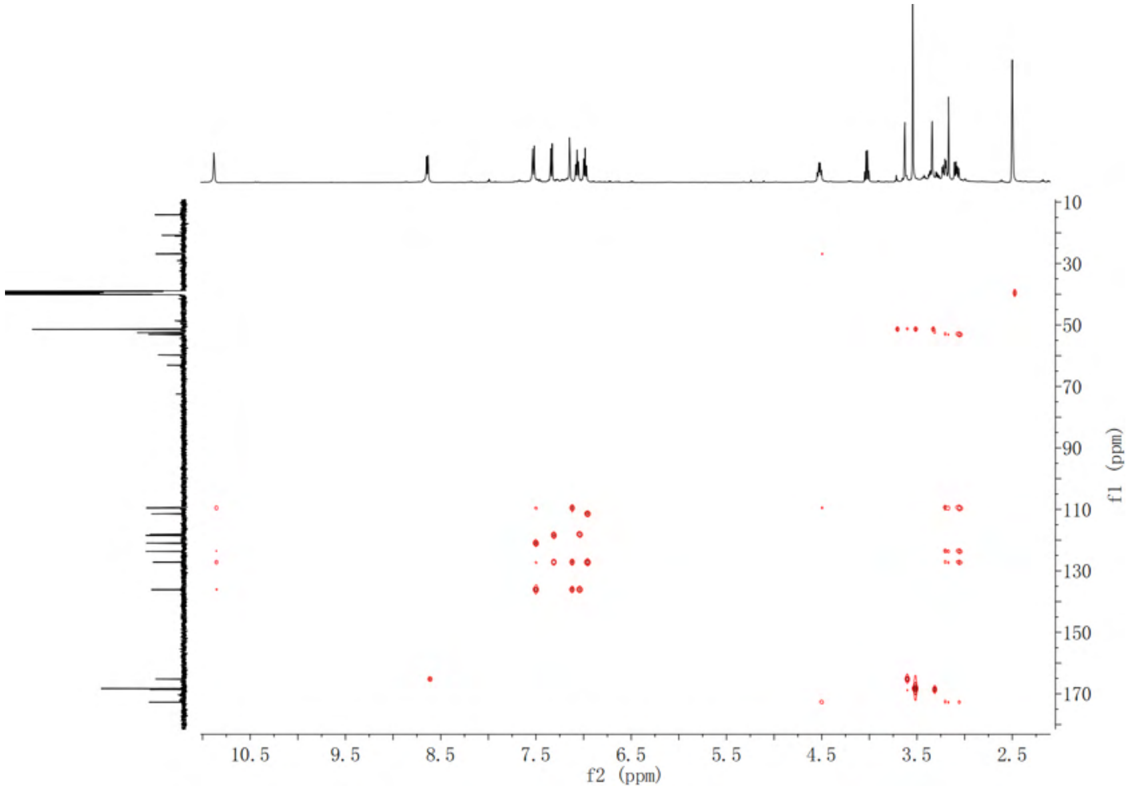

Supplementary Fig. 120. HMBC spectrum of compound (2*S*,3*S*)-*t*-ES-Trp in DMSO-*d*$_6$

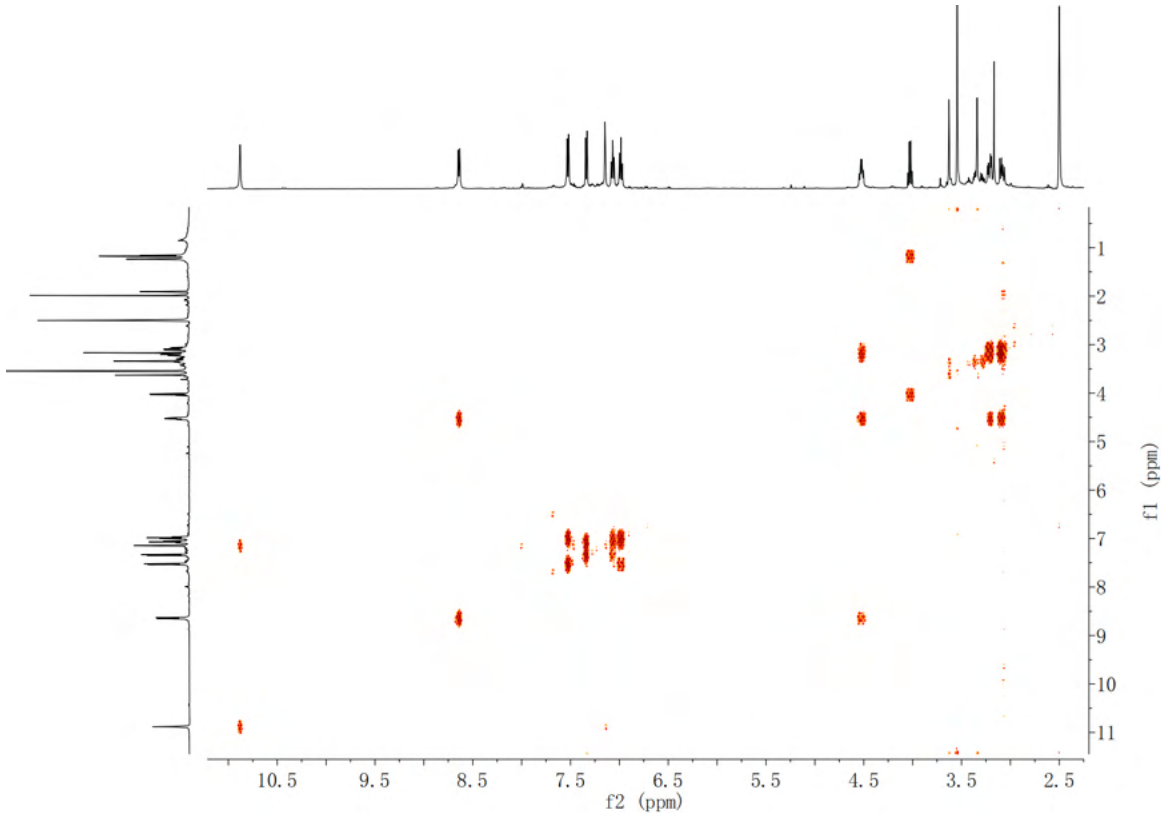

Supplementary Fig. 121. ^{1}H-^{1}H COSY spectrum of compound (2*S*,3*S*)-*t*-ES-Trp in DMSO-d_6

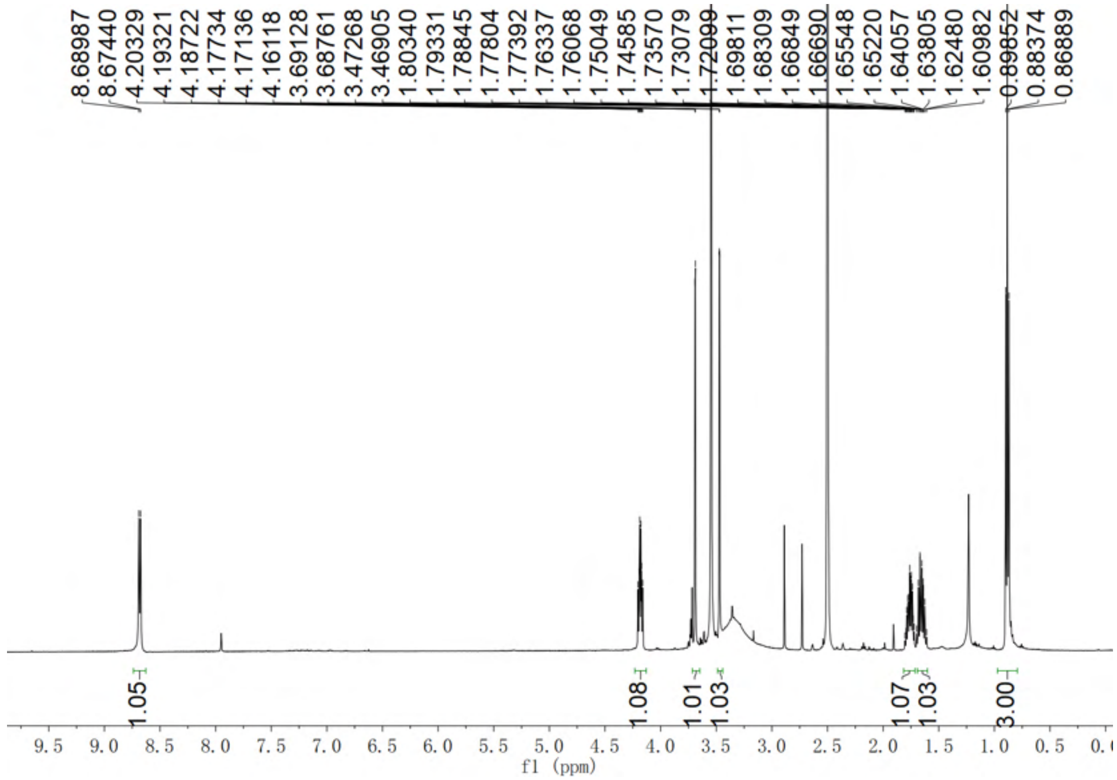

Supplementary Fig. 122. ^{1}H NMR spectrum of compound (2*S*,3*S*)-*t*-ES-a1 in DMSO-d_6

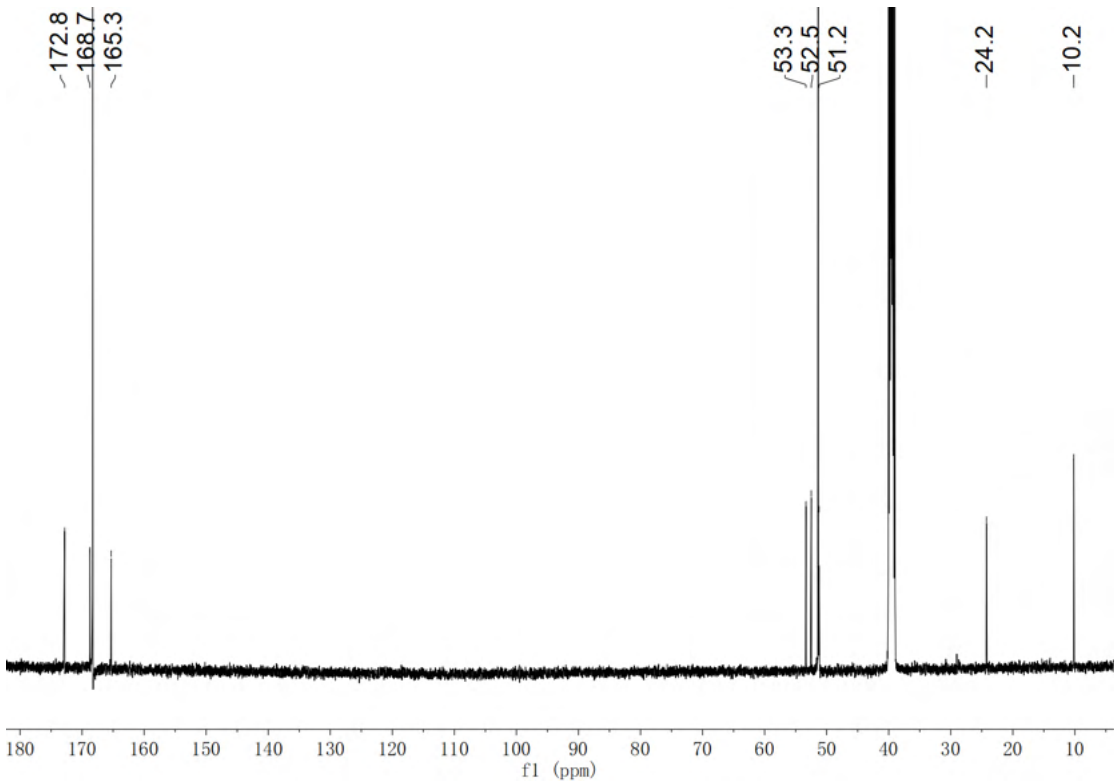

Supplementary Fig. 123. ^{13}C NMR spectrum of compound (2*S*,3*S*)-*t*-ES-a1 in DMSO-*d*$_6$

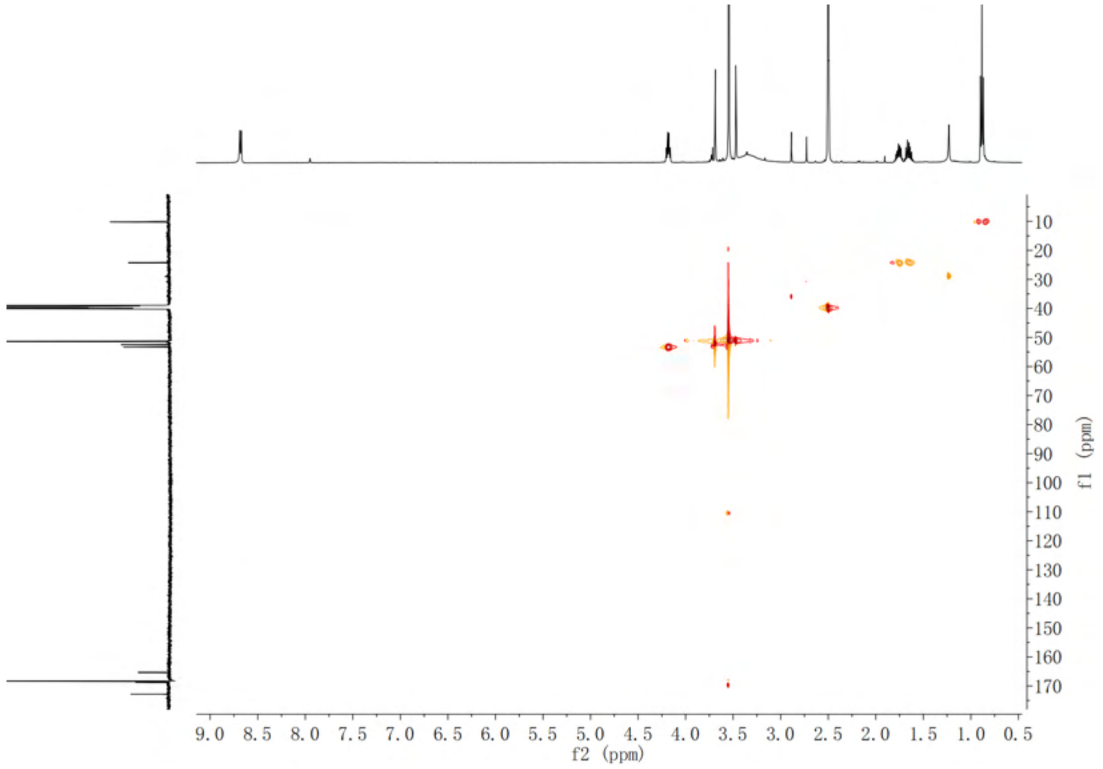

Supplementary Fig. 124. HSQC spectrum of compound (2*S*,3*S*)-*t*-ES-a1 in DMSO-*d*$_6$

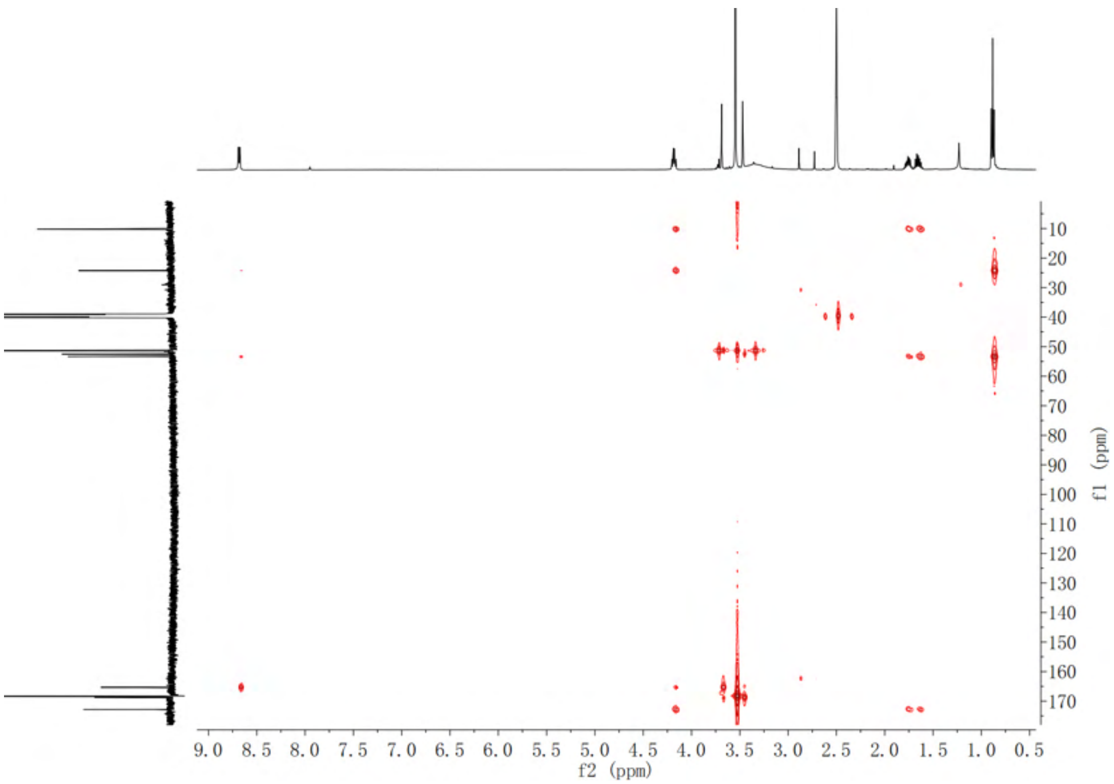

Supplementary Fig. 125. HMBC spectrum of compound (2*S*,3*S*)-*t*-ES-a1 in DMSO-d_6

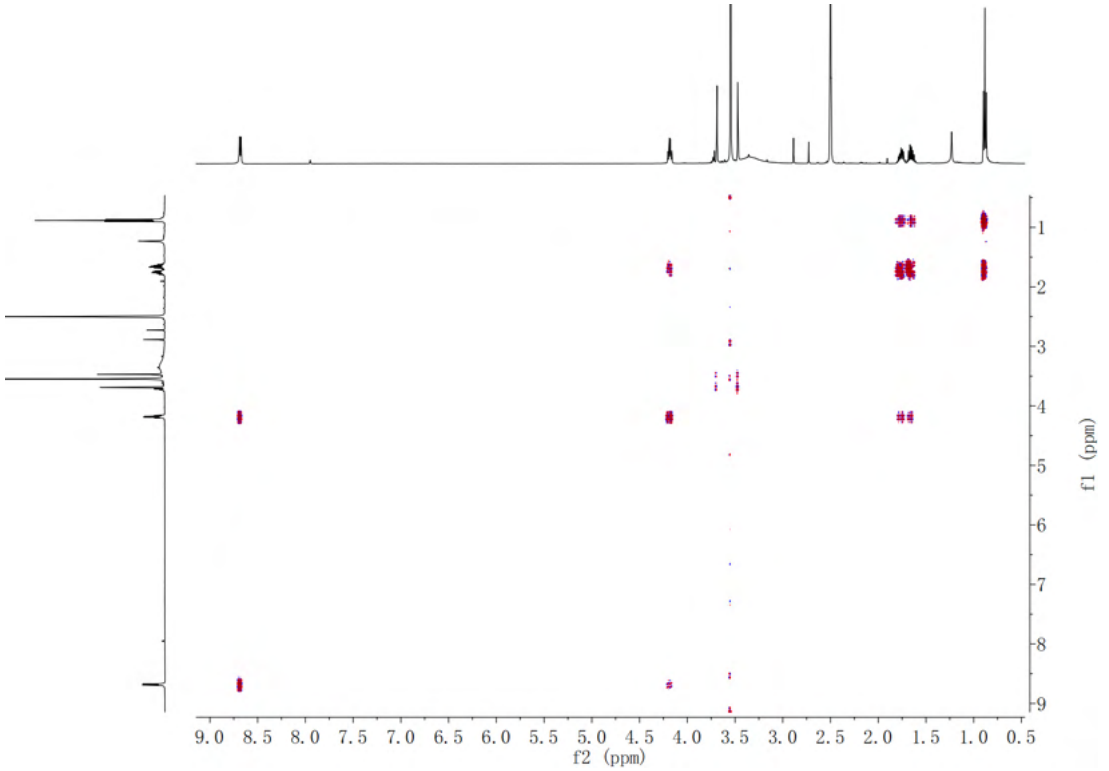

Supplementary Fig. 126. ^{1}H-^{1}H COSY spectrum of compound (2*S*,3*S*)-*t*-ES-a1 in DMSO-d_6

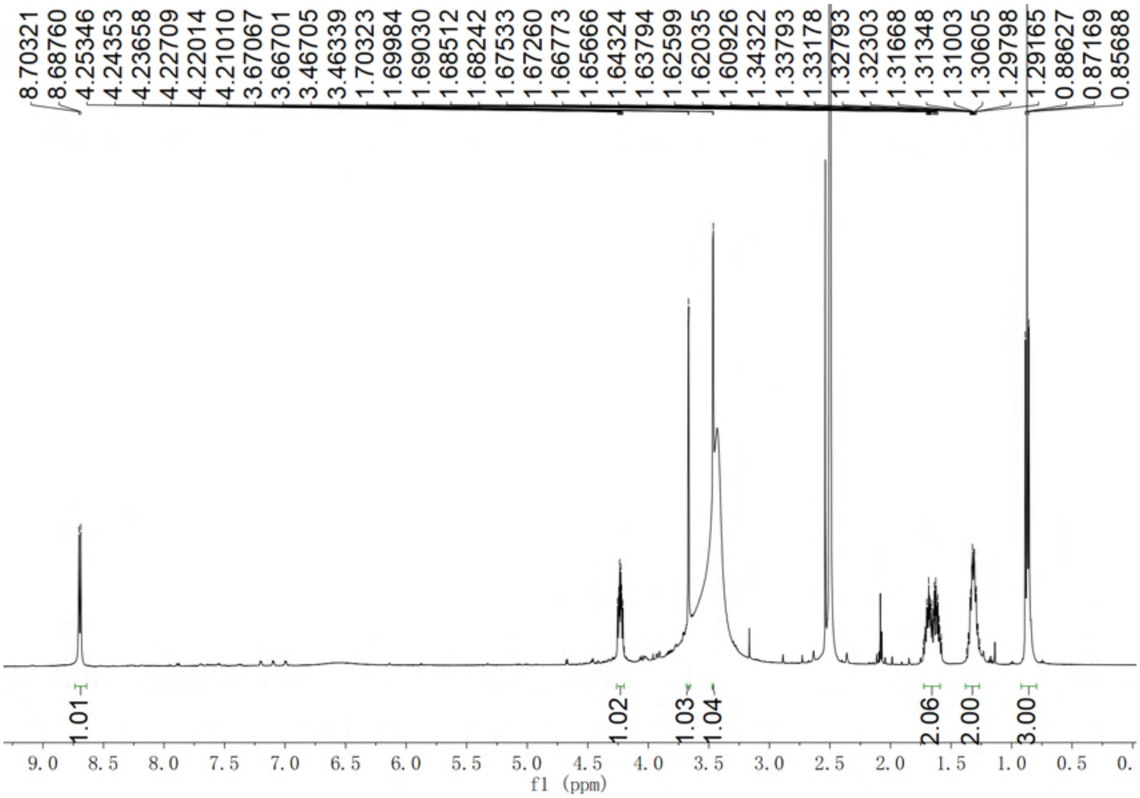

Supplementary Fig. 127. ^{1}H NMR spectrum of compound (2*S*,3*S*)-*t*-ES-a2 in DMSO-d_6

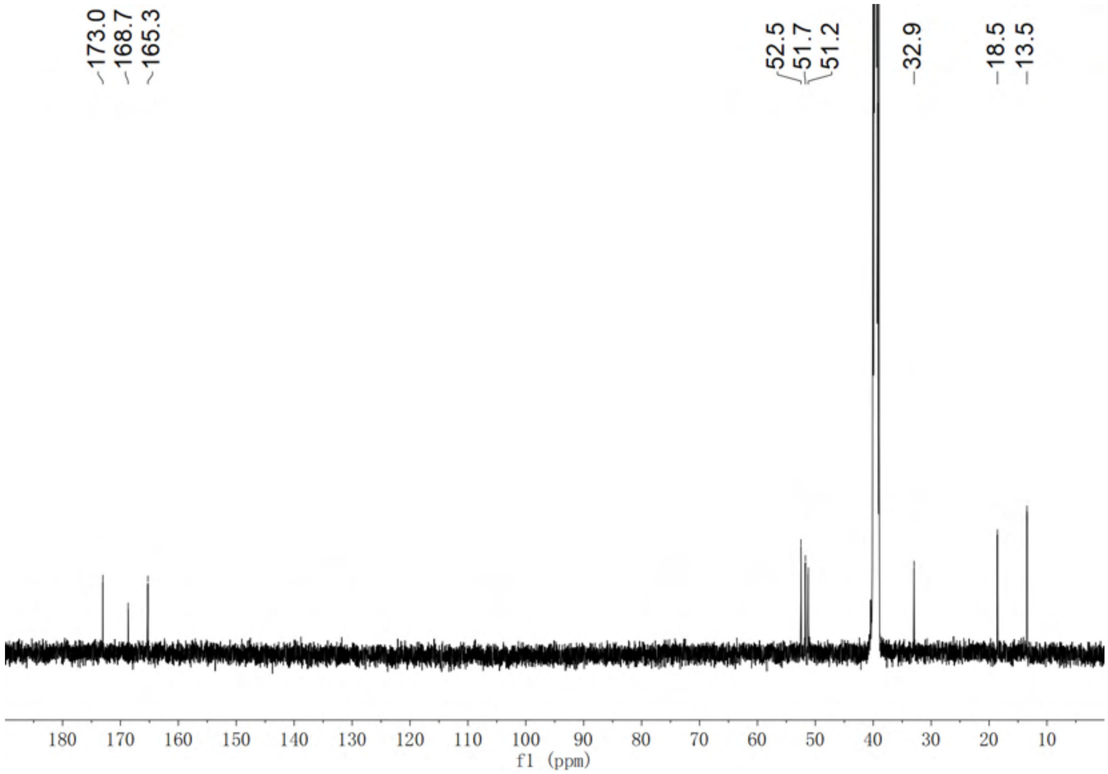

Supplementary Fig. 128. ^{13}C NMR spectrum of compound (2*S*,3*S*)-*t*-ES-a2 in DMSO-d_6

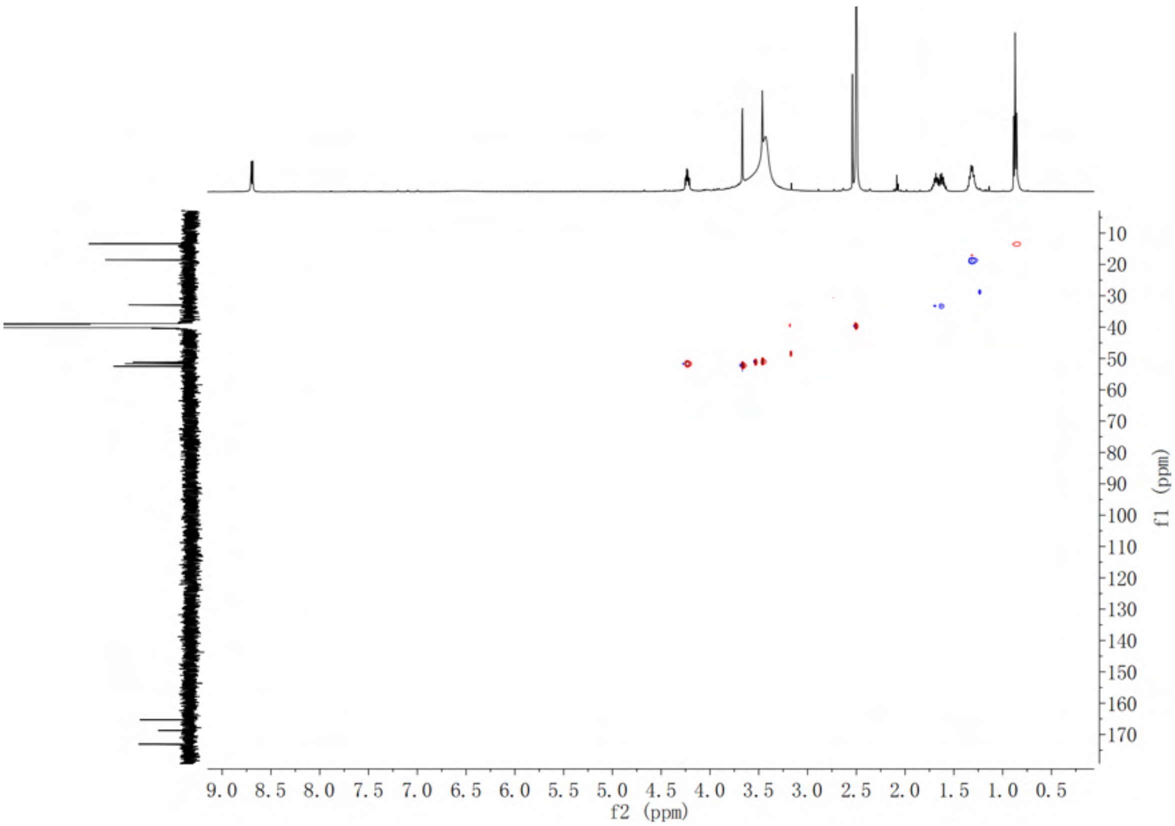

Supplementary Fig. 129. HSQC spectrum of compound (2*S*,3*S*)-*t*-ES-a2 in DMSO-d_6

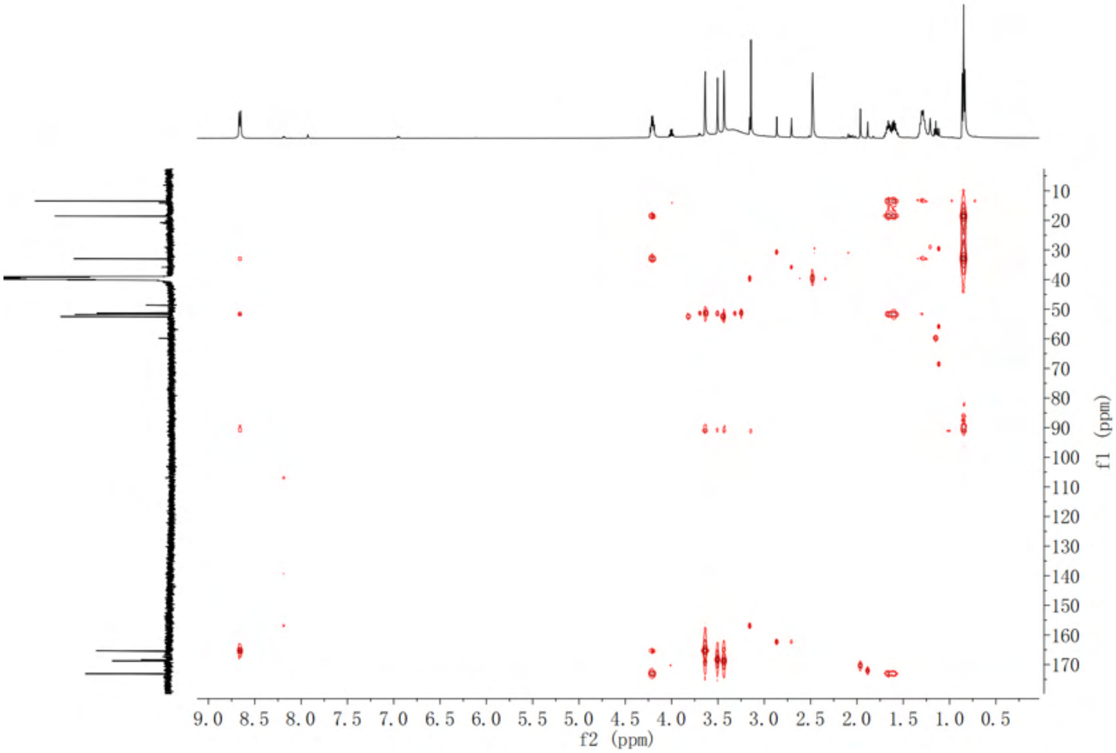

Supplementary Fig. 130. HMBC spectrum of compound (2*S*,3*S*)-*t*-ES-a2 in DMSO-d_6

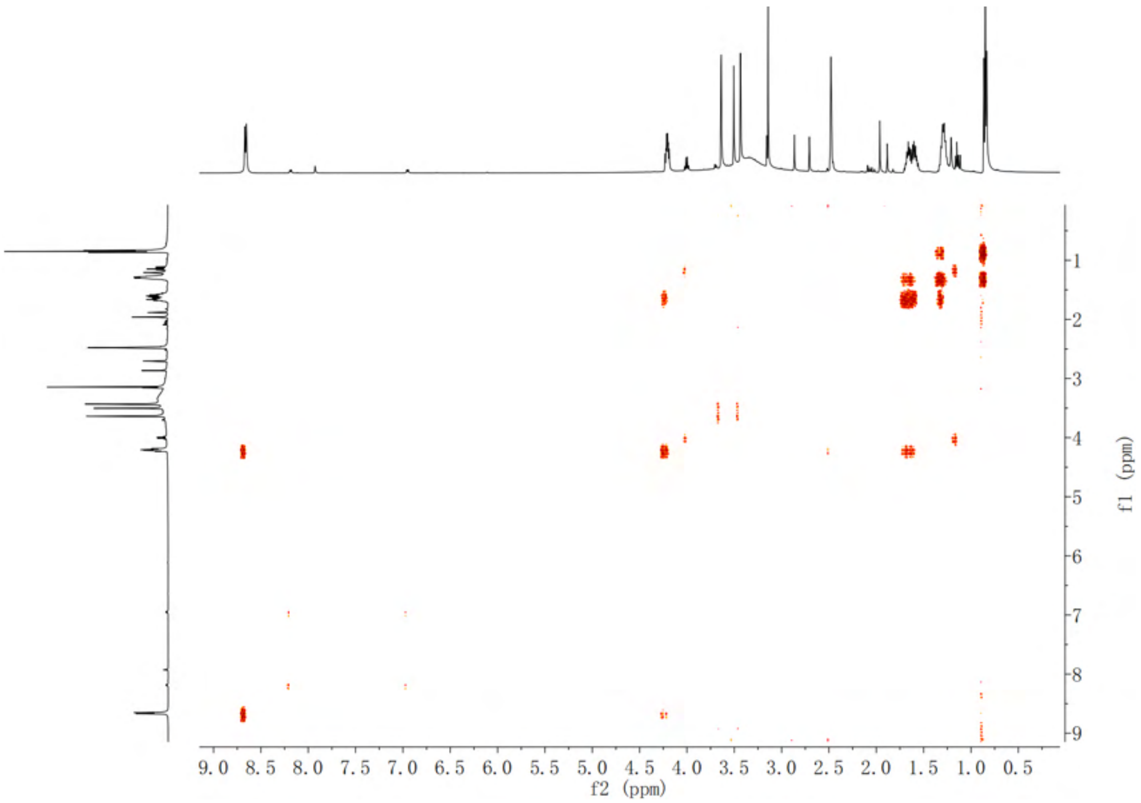

Supplementary Fig. 131. ^{1}**H-**^{1}**H COSY spectrum of compound (2*S*,3*S*)-*t*-ES-a2 in DMSO-*d*$_6$**

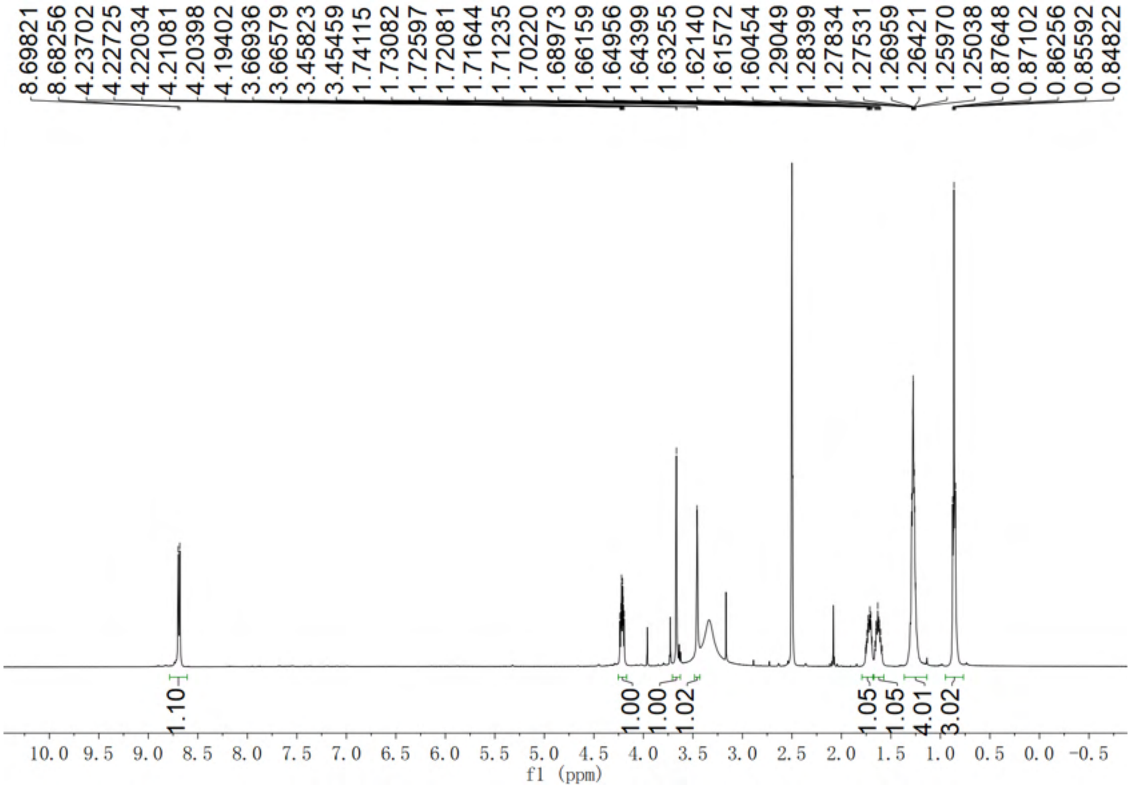

Supplementary Fig. 132. ^{1}**H NMR spectrum of compound (2*S*,3*S*)-*t*-ES-a3 in DMSO-*d*$_6$**

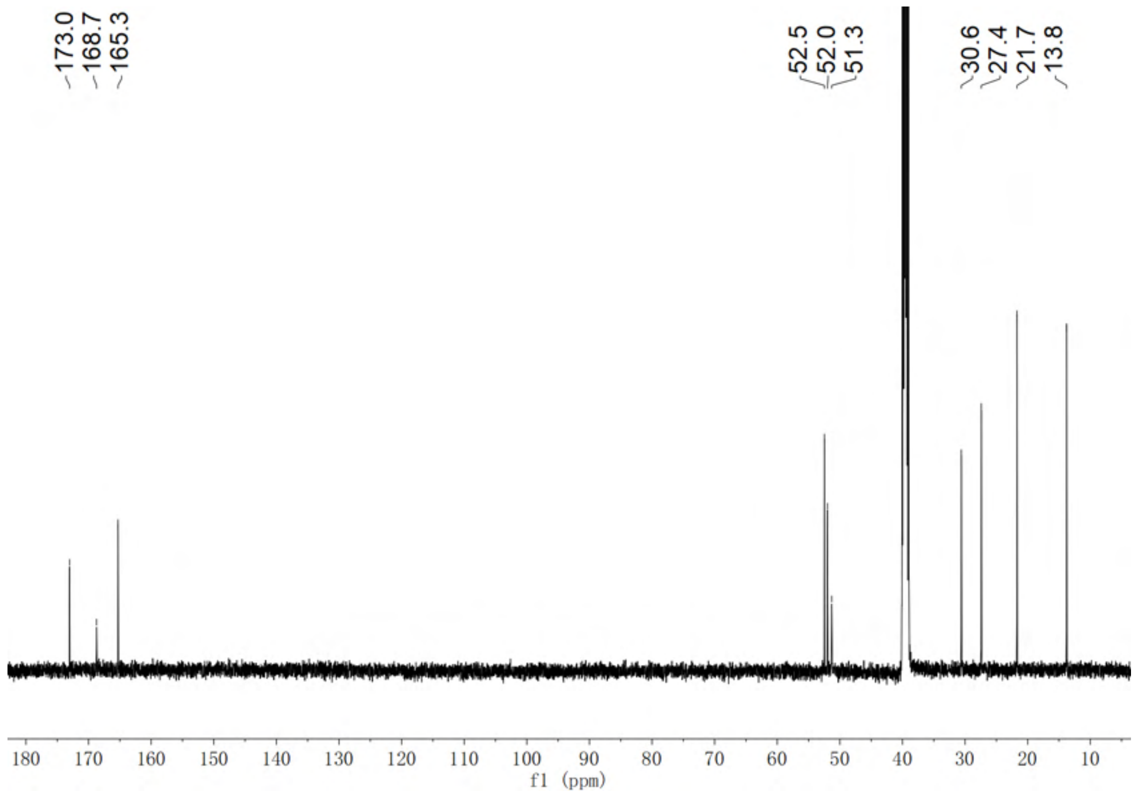

Supplementary Fig. 133. ^{13}C NMR spectrum of compound (2*S*,3*S*)-*t*-ES-a3 in DMSO-d_6

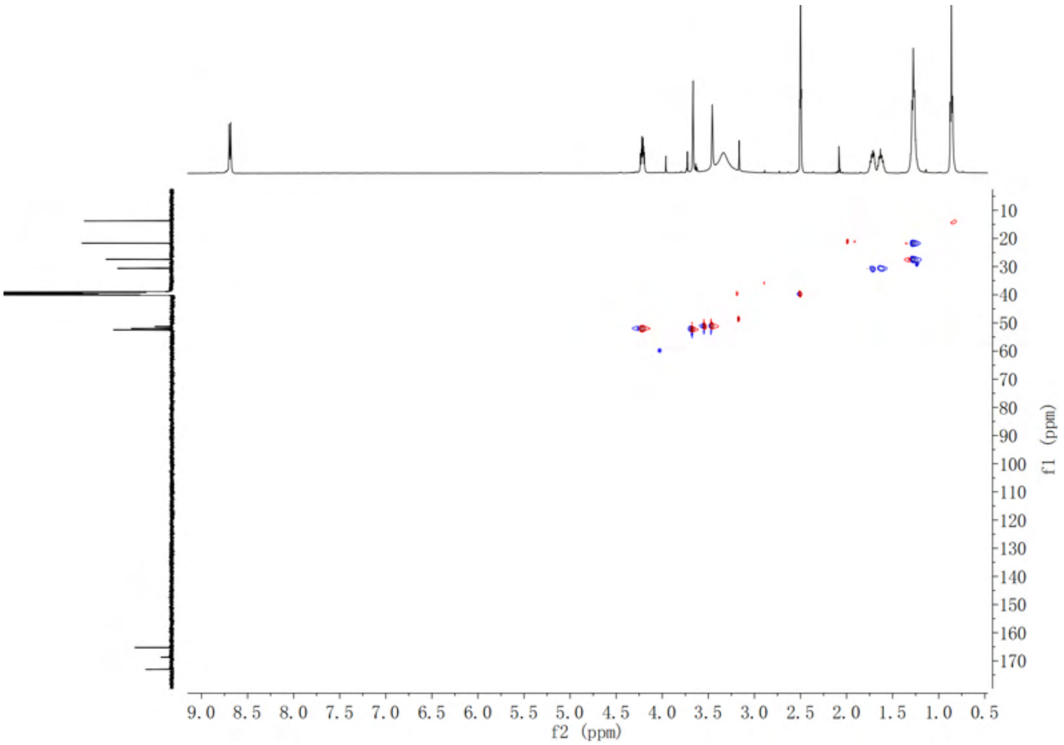

Supplementary Fig. 134. HSQC spectrum of compound (2*S*,3*S*)-*t*-ES-a3 in DMSO-d_6

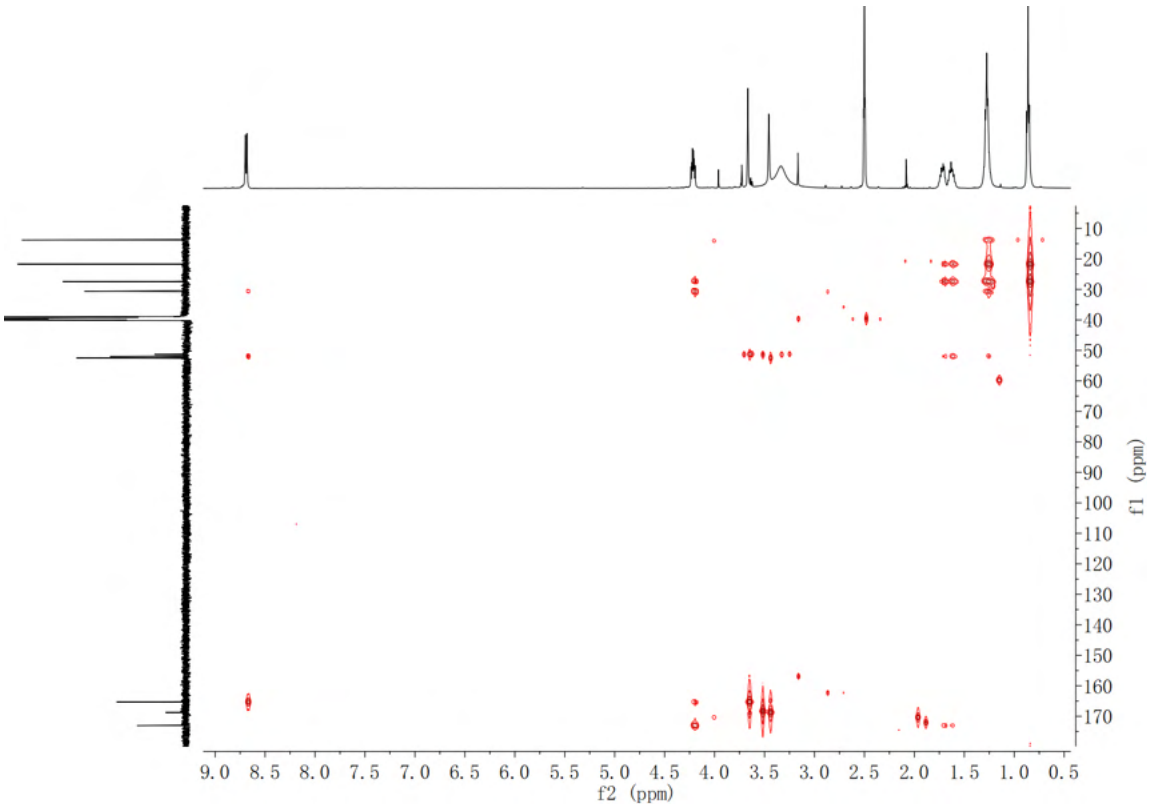

Supplementary Fig. 135. HMBC spectrum of compound (2*S*,3*S*)-*t*-ES-a3 in DMSO-d_6

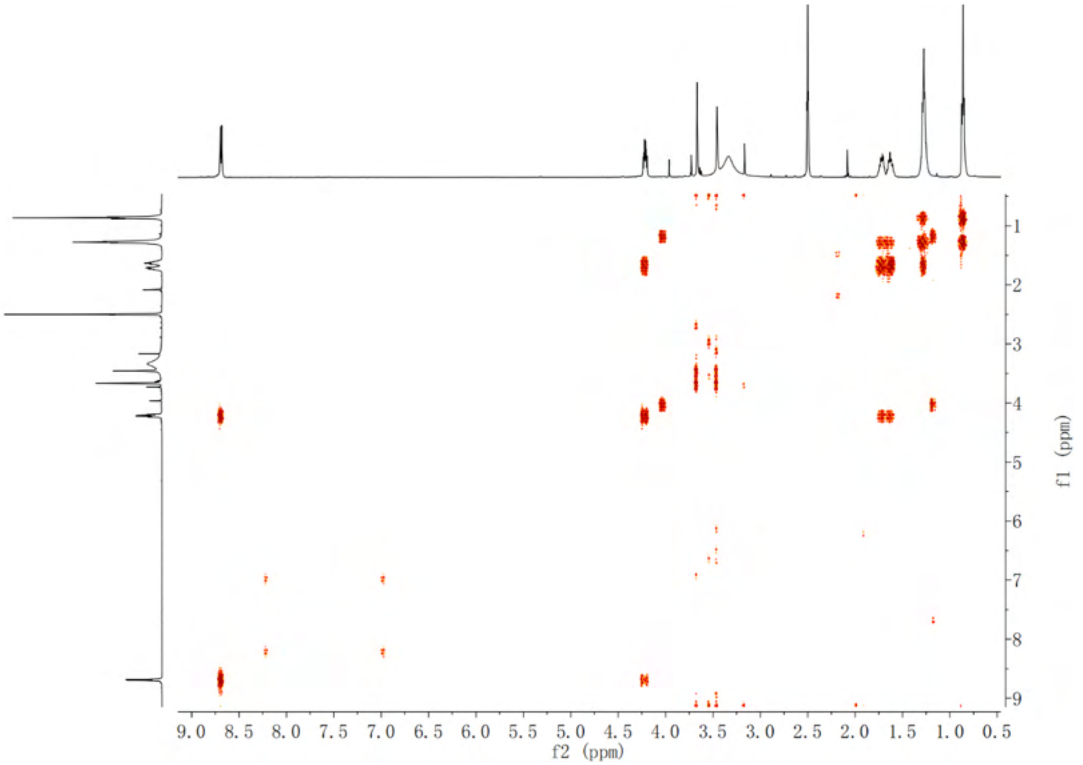

Supplementary Fig. 136. ^{1}H-^{1}H COSY spectrum of compound (2*S*,3*S*)-*t*-ES-a3 in DMSO-d_6

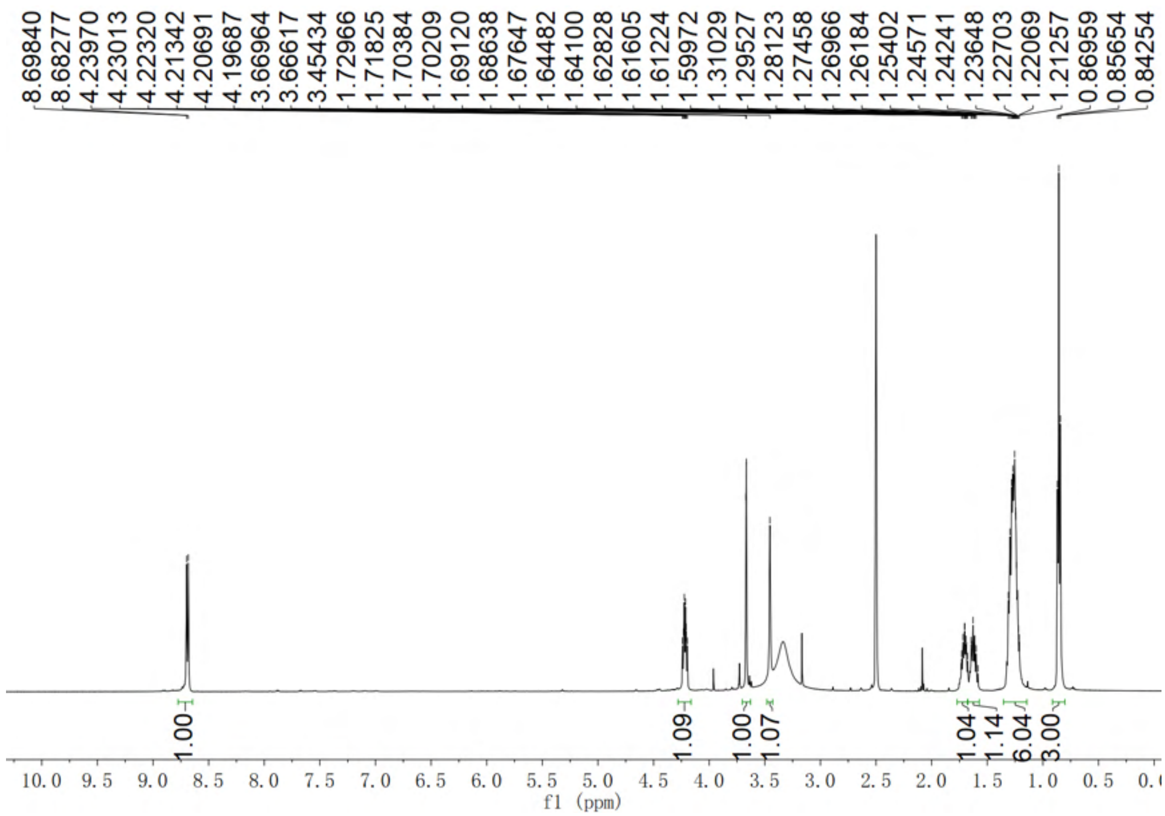

Supplementary Fig. 137. **^{1}H NMR spectrum of compound (2*S*,3*S*)-*t*-ES-a4 in DMSO-*d*$_6$**

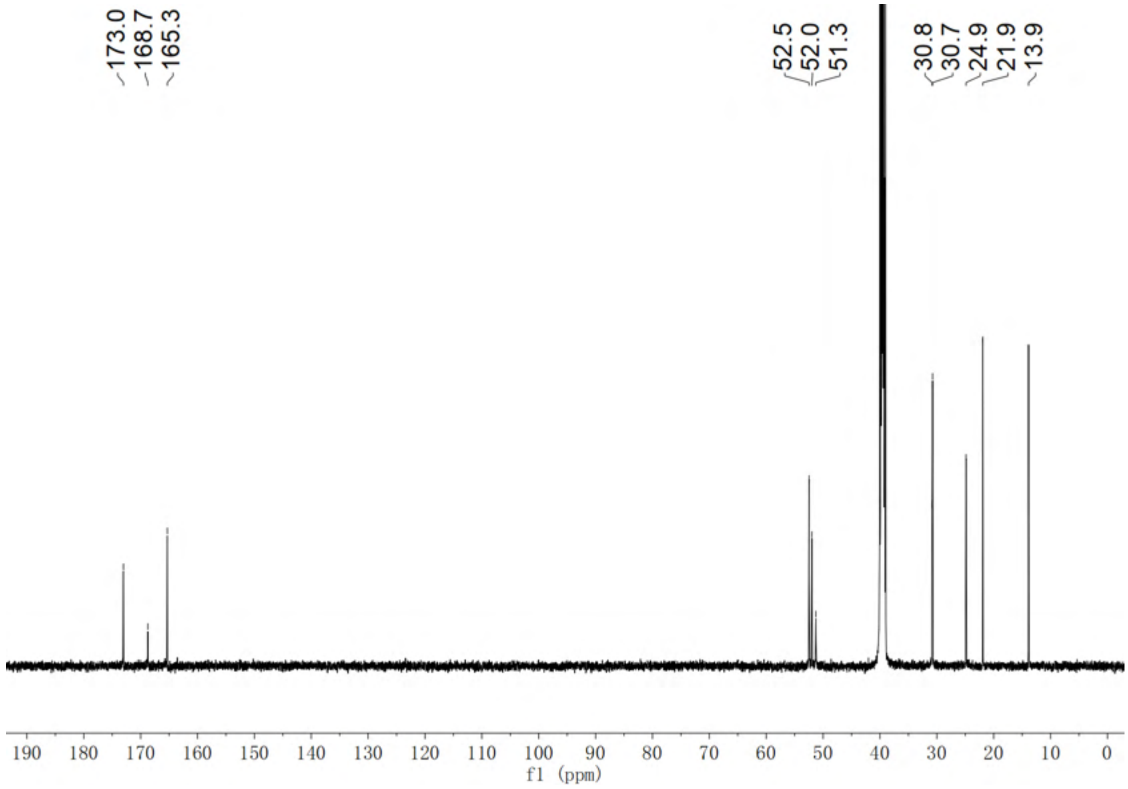

Supplementary Fig. 138. **^{13}C NMR spectrum of compound (2*S*,3*S*)-*t*-ES-a4 in DMSO-*d*$_6$**

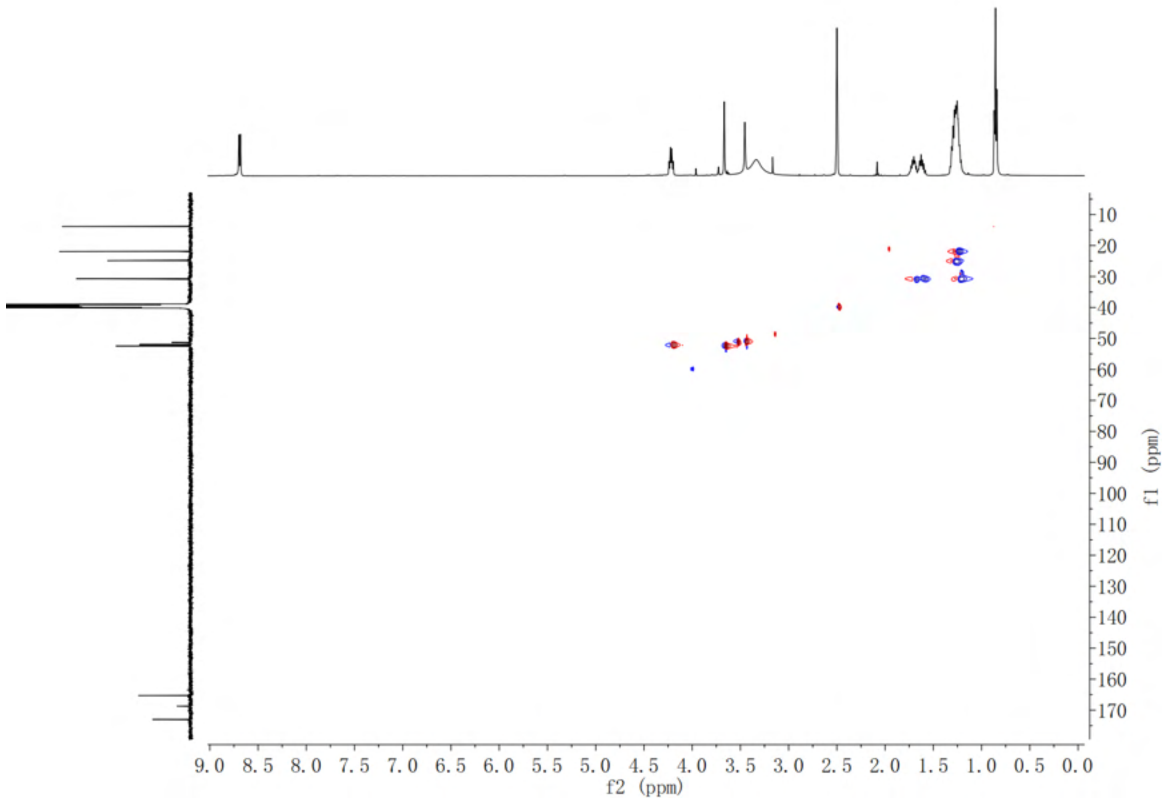

Supplementary Fig. 139. HSQC spectrum of compound (2*S*,3*S*)-*t*-ES-a4 in DMSO-d_6

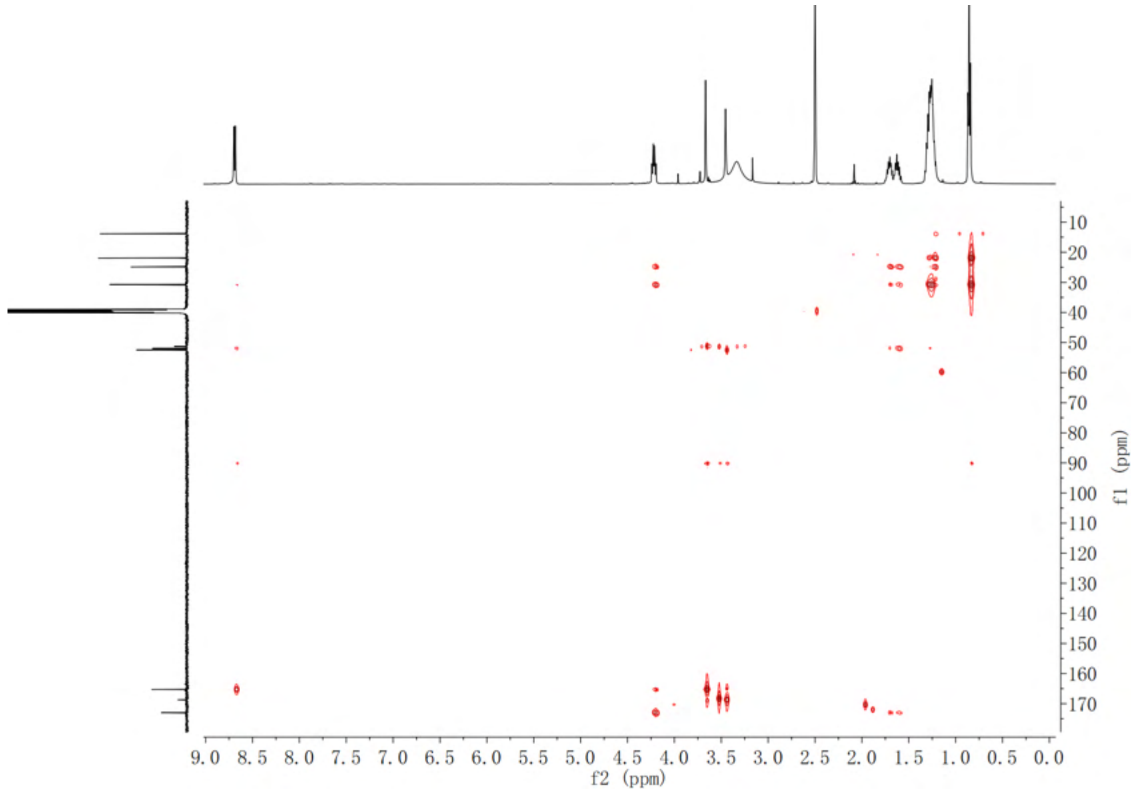

Supplementary Fig. 140. HMBC spectrum of compound (2*S*,3*S*)-*t*-ES-a4 in DMSO-d_6

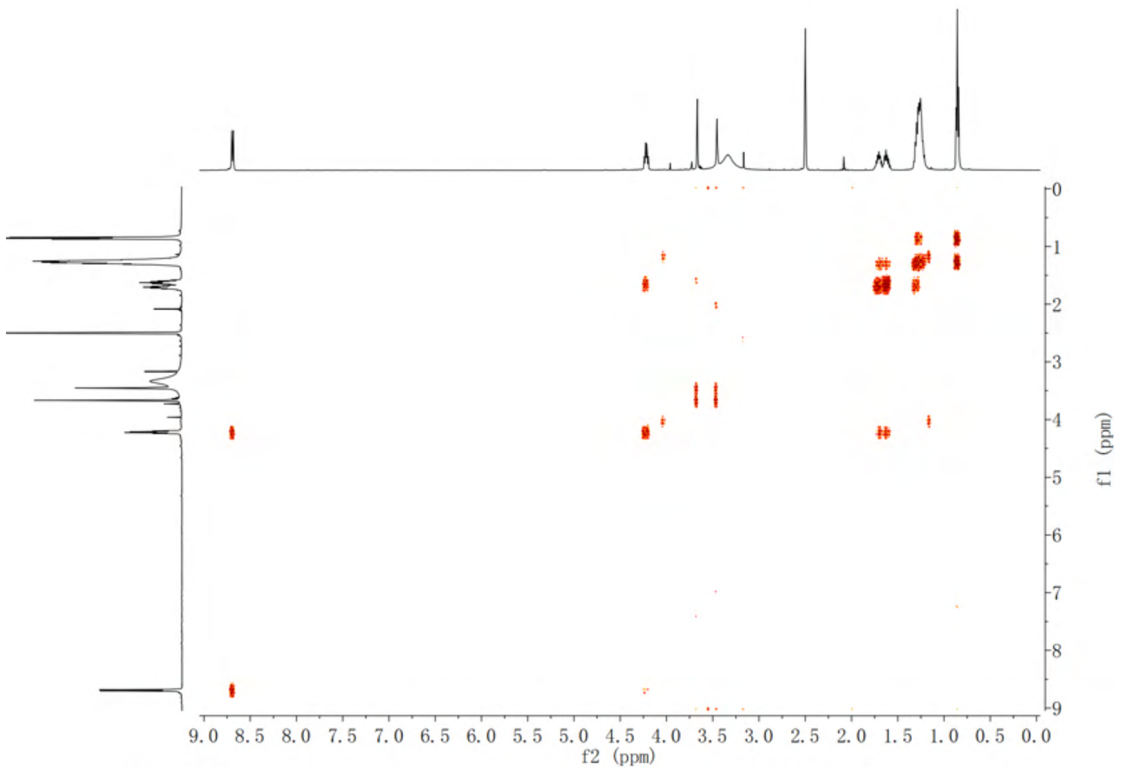

Supplementary Fig. 141. ^{1}H-^{1}H COSY spectrum of compound (2*S*,3*S*)-*t*-ES-a4 in DMSO-d_6

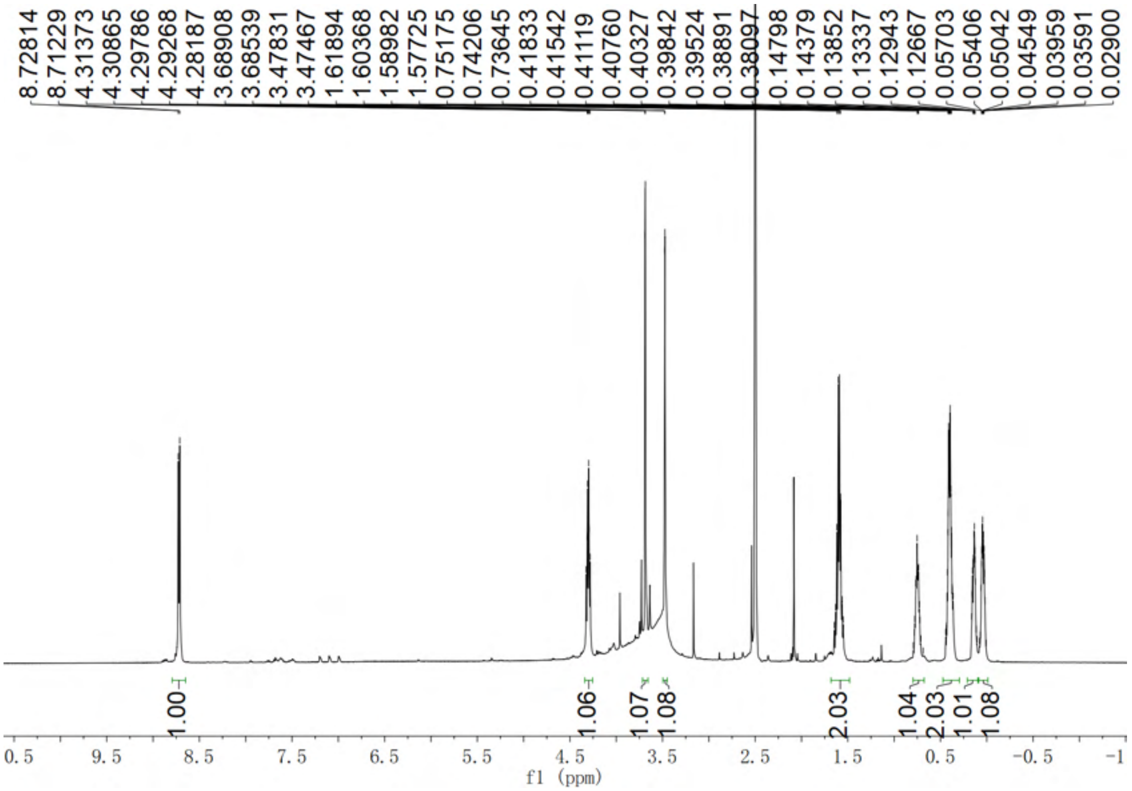

Supplementary Fig. 142. ^{1}H NMR spectrum of compound (2*S*,3*S*)-*t*-ES-a5 in DMSO-d_6

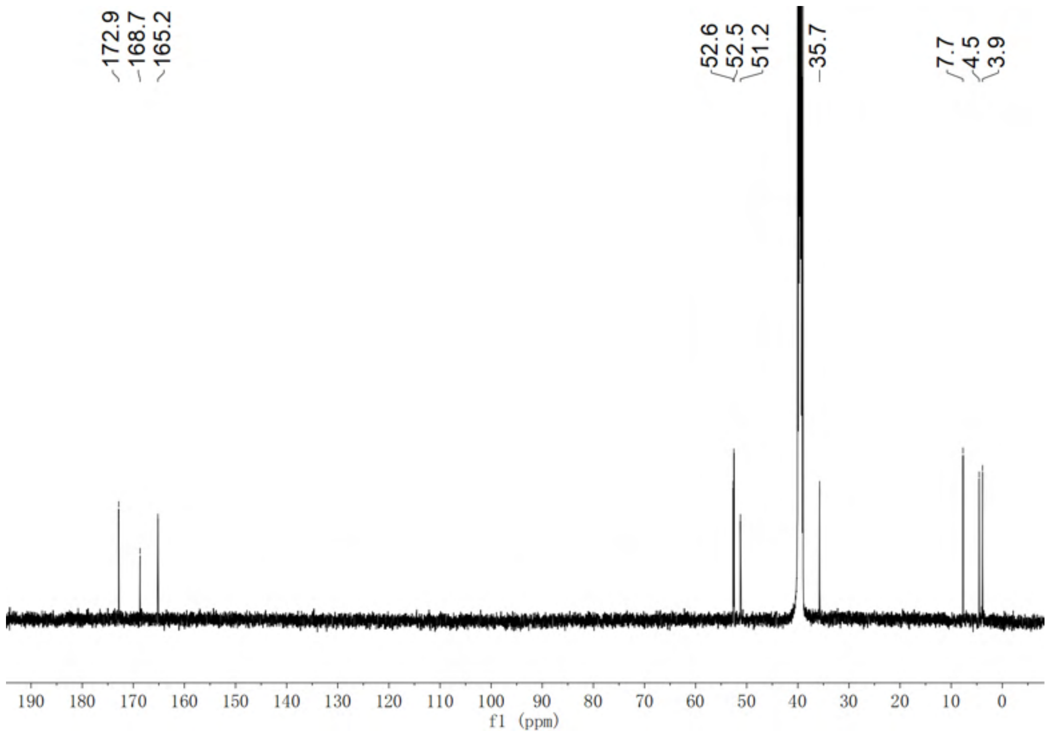

Supplementary Fig. 143. ^{13}C NMR spectrum of compound (2*S*,3*S*)-*t*-ES-a5 in DMSO-d_6

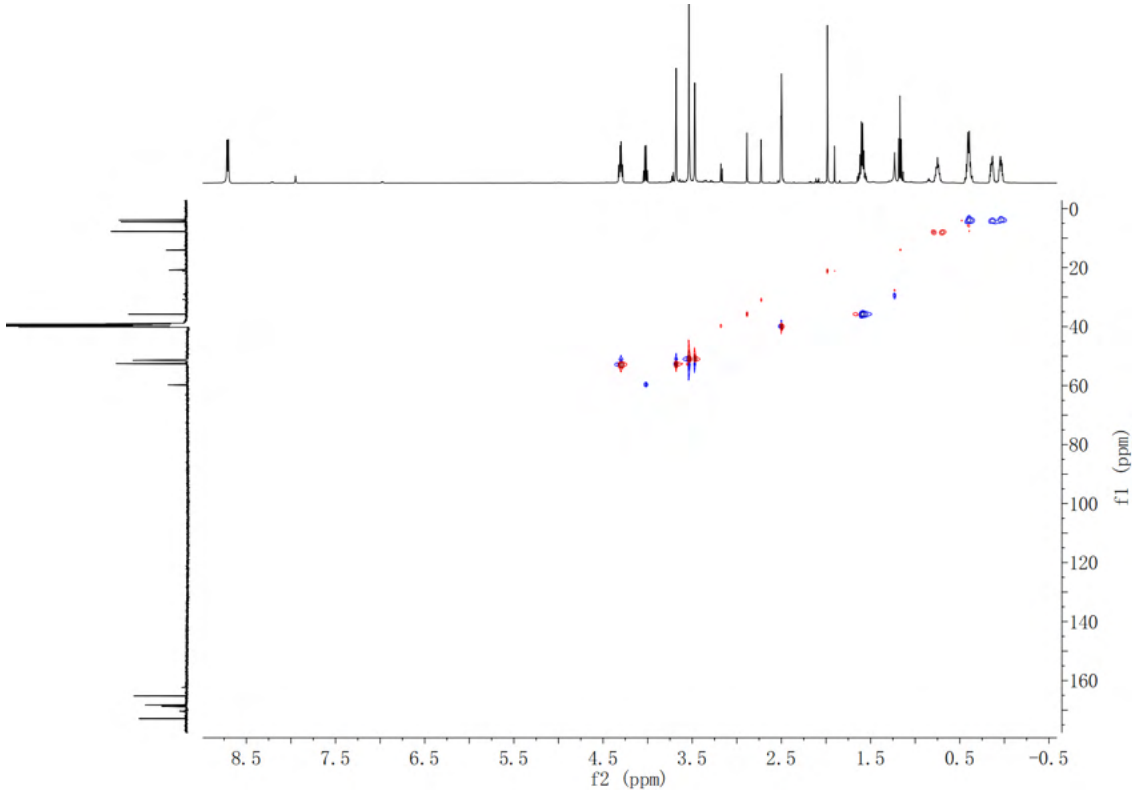

Supplementary Fig. 144. HSQC spectrum of compound (2*S*,3*S*)-*t*-ES-a5 in DMSO-d_6

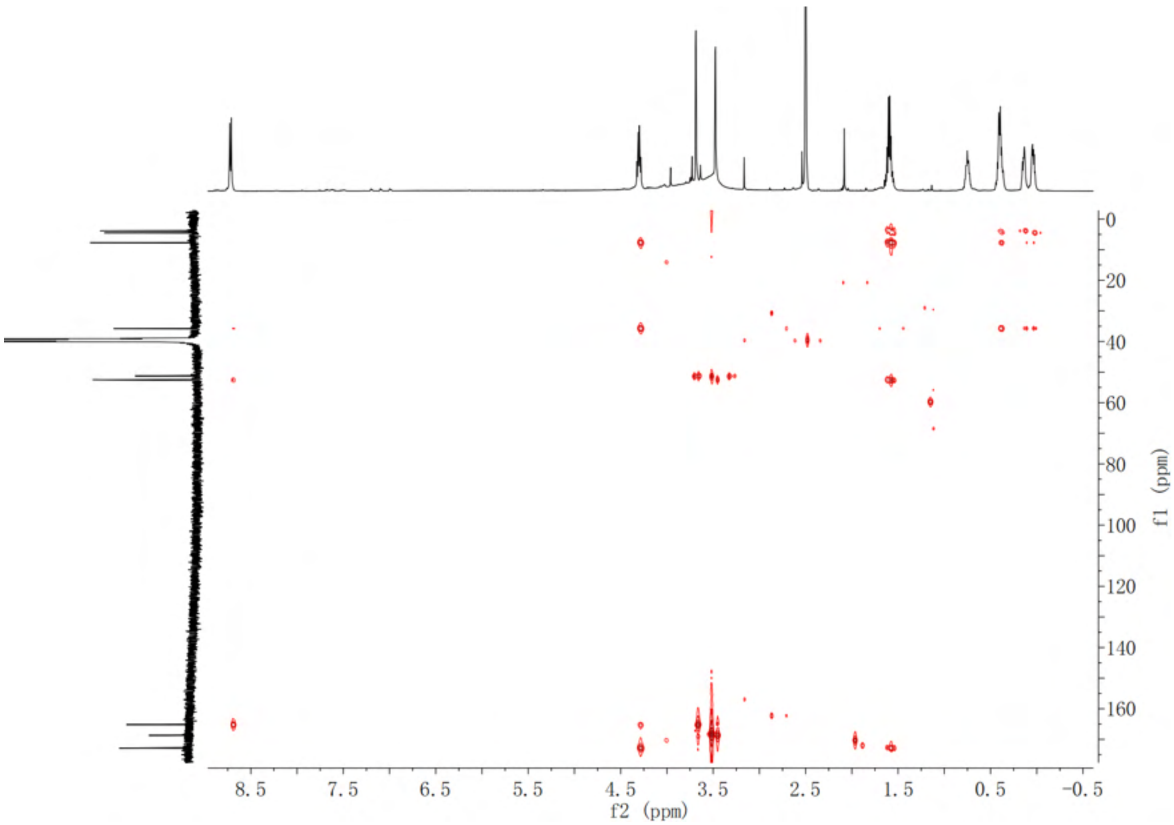

Supplementary Fig. 145. HMBC spectrum of compound (2*S*,3*S*)-*t*-ES-a5 in DMSO-d_6

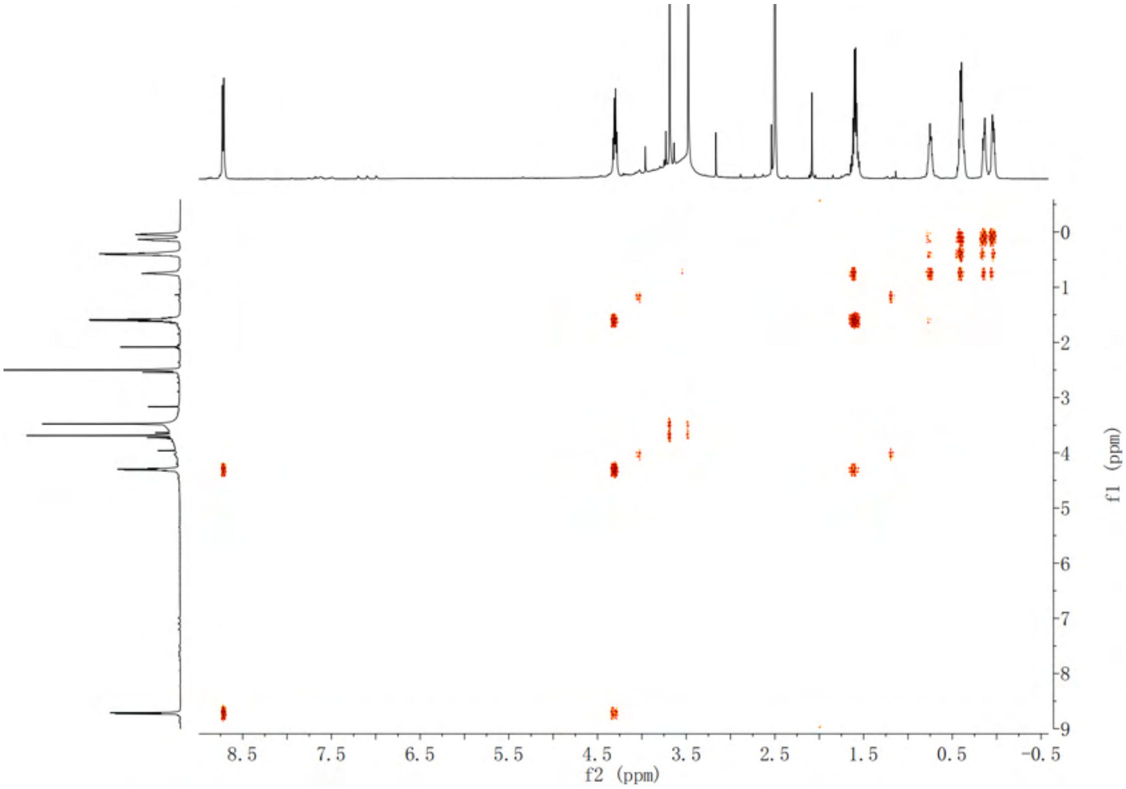

Supplementary Fig. 146. ^{1}H-^{1}H COSY spectrum of compound (2*S*,3*S*)-*t*-ES-a5 in DMSO-d_6

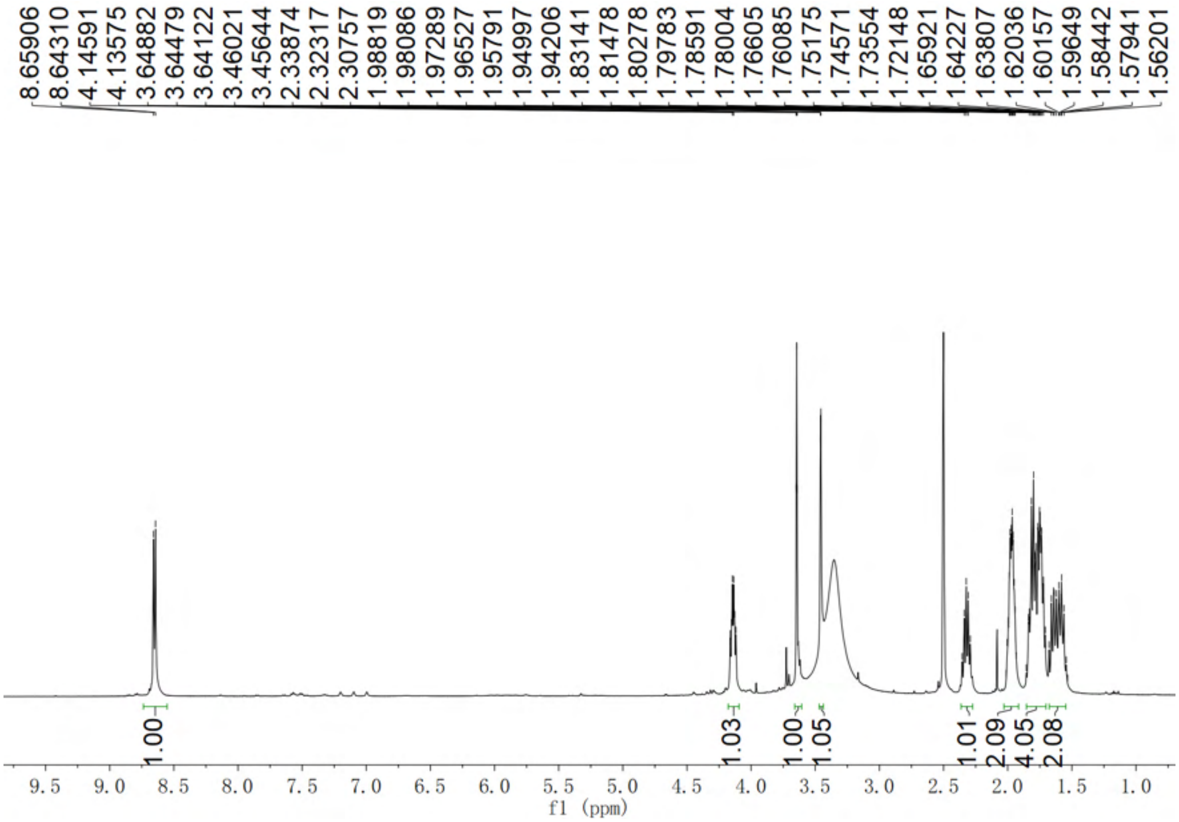

Supplementary Fig. 147. ^{1}H NMR spectrum of compound (2*S*,3*S*)-*t*-ES-a6 in DMSO-d_6

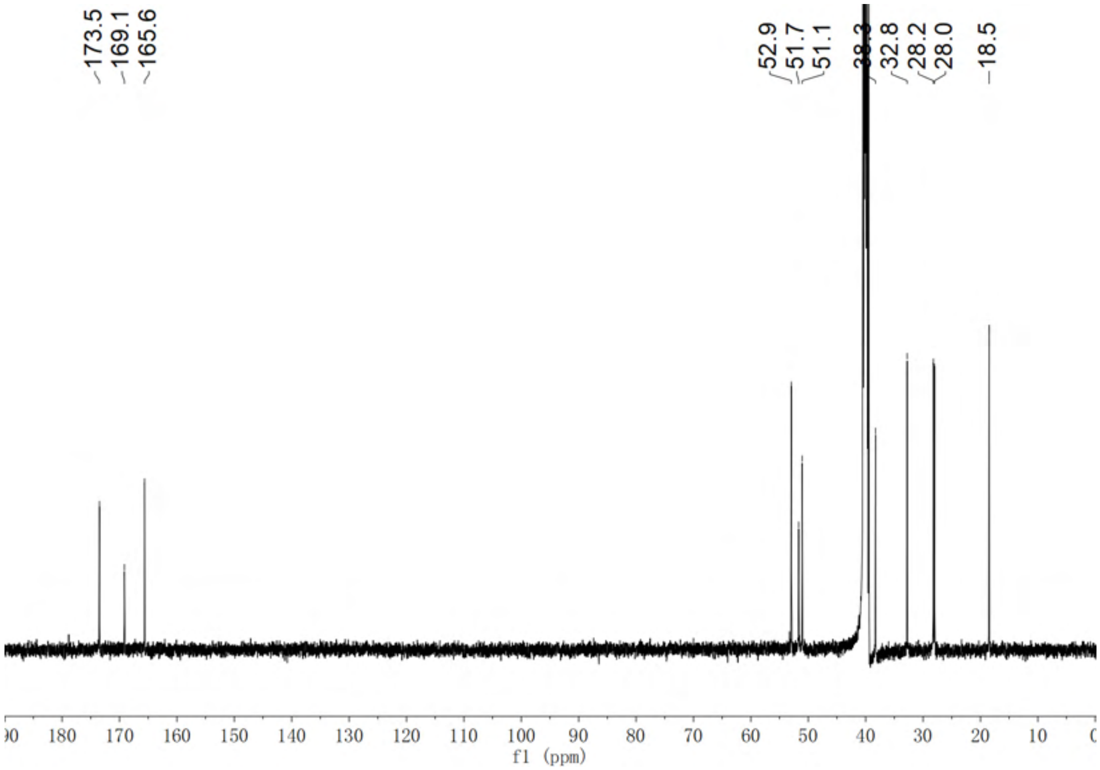

Supplementary Fig. 148. ^{13}C NMR spectrum of compound (2*S*,3*S*)-*t*-ES-a6 in DMSO-d_6

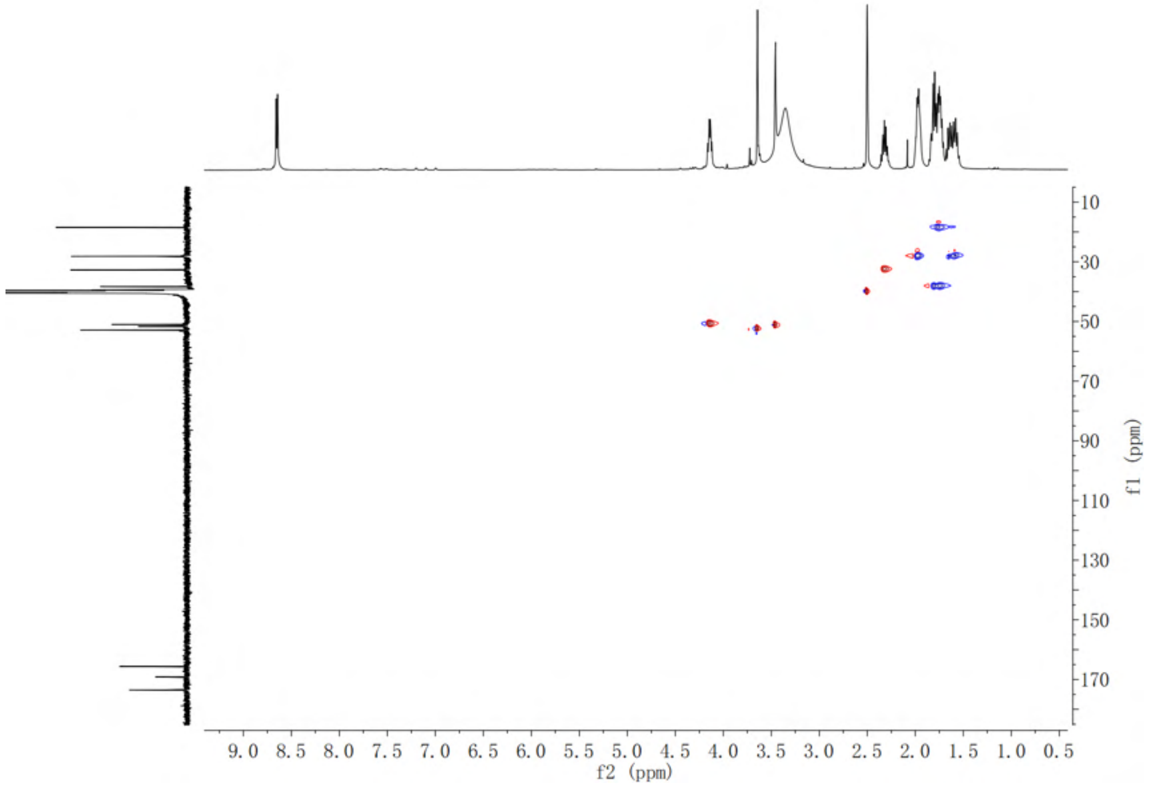

Supplementary Fig. 149. HSQC spectrum of compound (2*S*,3*S*)-*t*-ES-a6 in DMSO-d_6

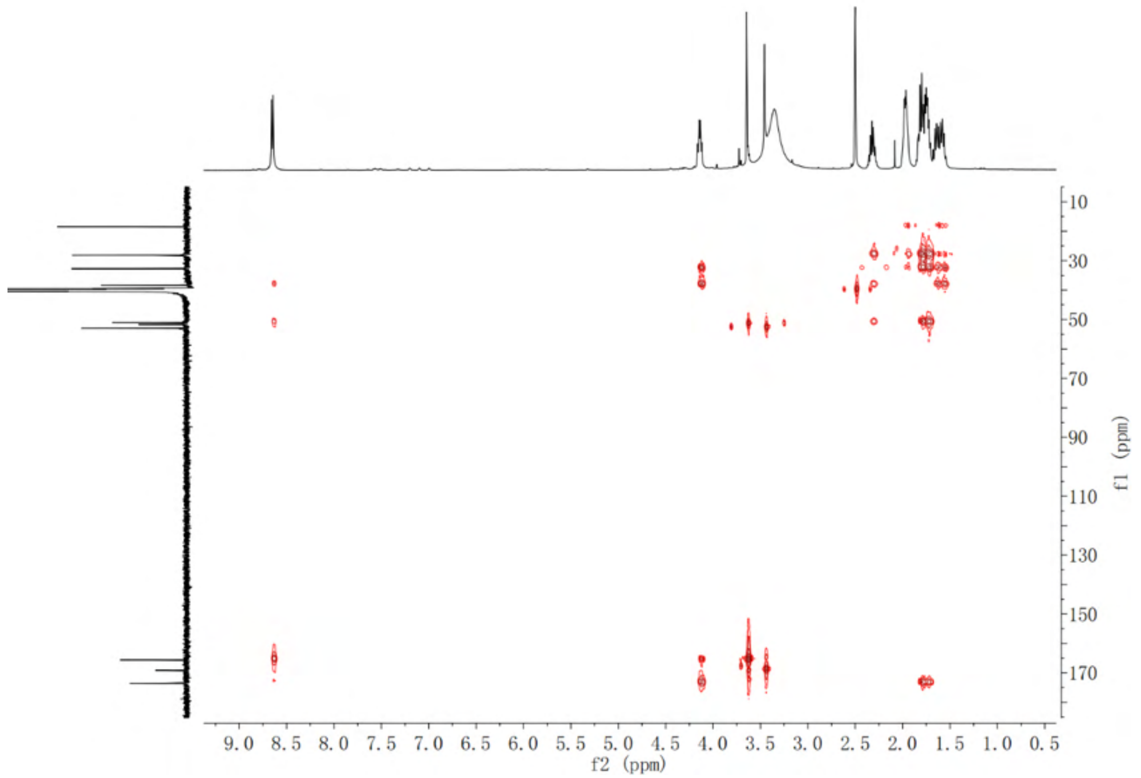

Supplementary Fig. 150. HMBC spectrum of compound (2*S*,3*S*)-*t*-ES-a6 in DMSO-d_6

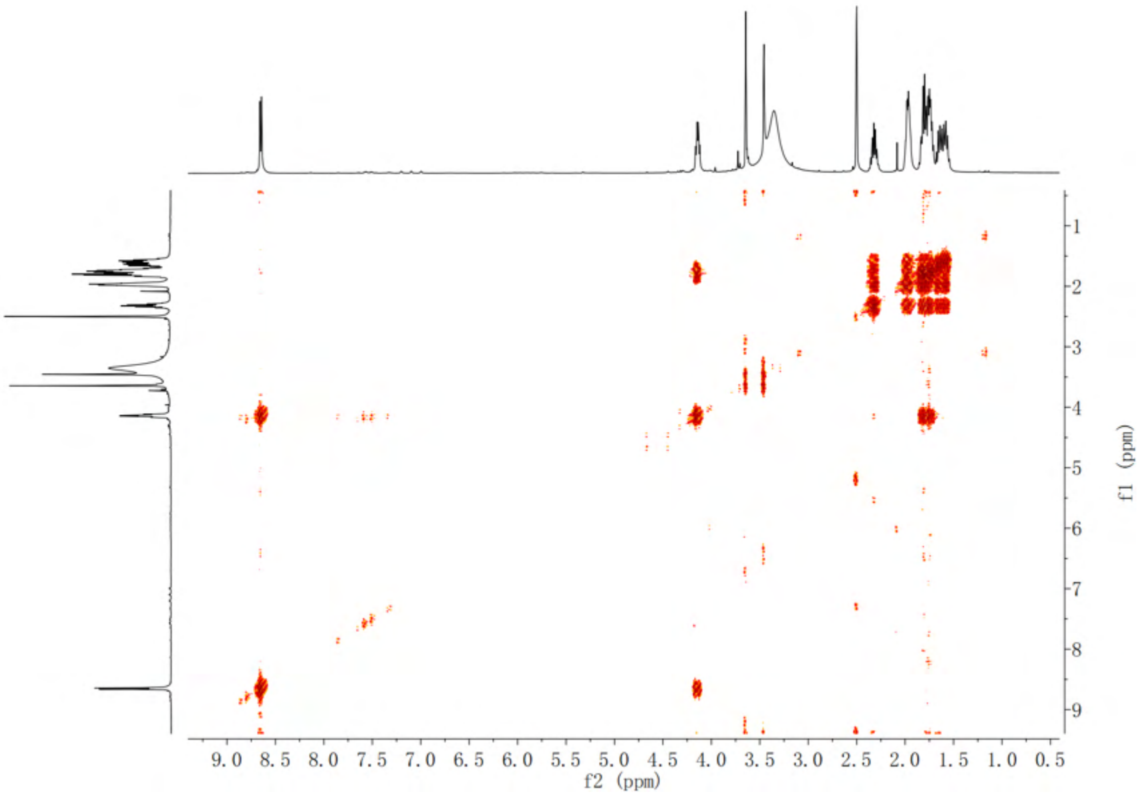

Supplementary Fig. 151. **^{1}H-^{1}H COSY spectrum of compound (2*S*,3*S*)-*t*-ES-a6 in DMSO-d_6**

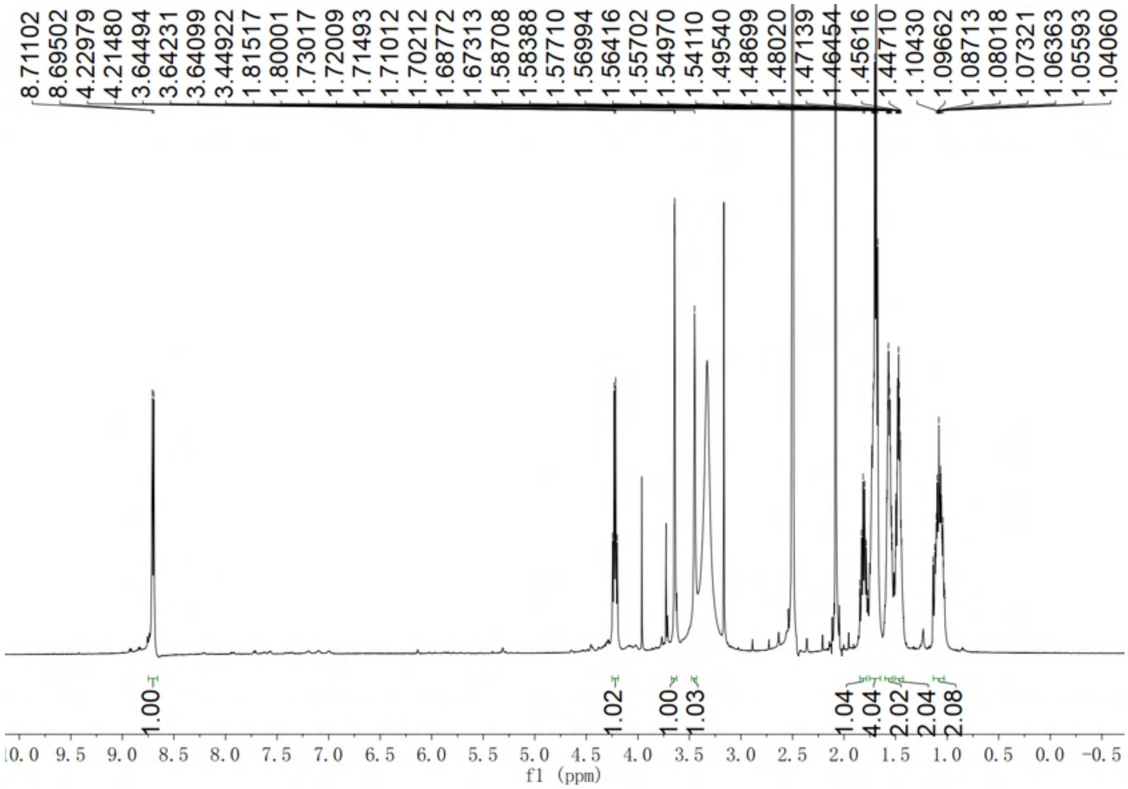

Supplementary Fig. 152. **^{1}H NMR spectrum of compound (2*S*,3*S*)-*t*-ES-a7 in DMSO-d_6**

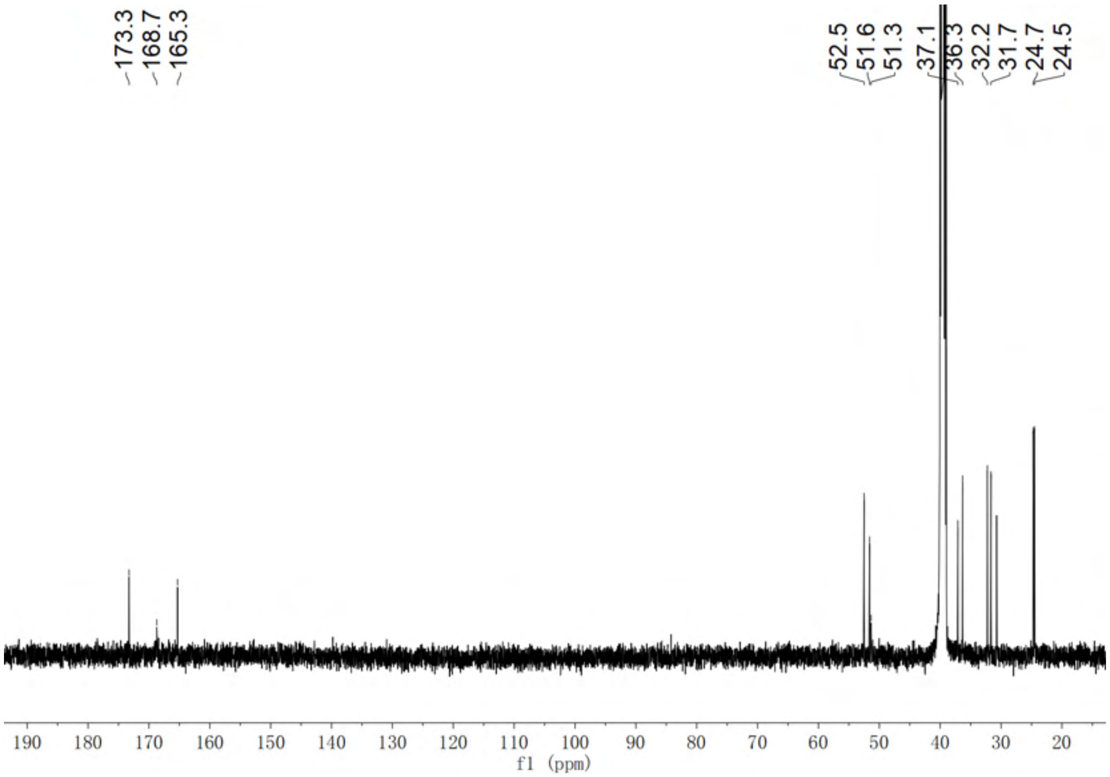

Supplementary Fig. 153. ^{13}C NMR spectrum of compound (2*S*,3*S*)-*t*-ES-a7 in DMSO-d_6

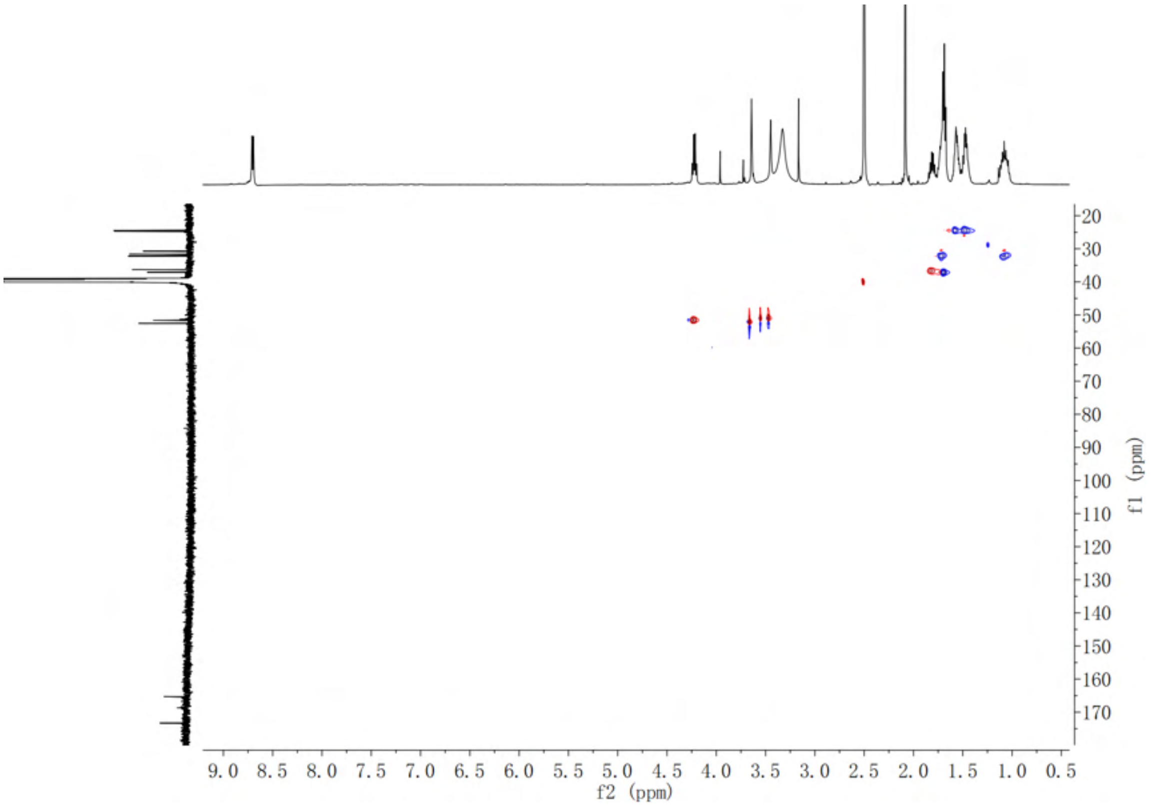

Supplementary Fig. 154. HSQC spectrum of compound (2*S*,3*S*)-*t*-ES-a7 in DMSO-d_6

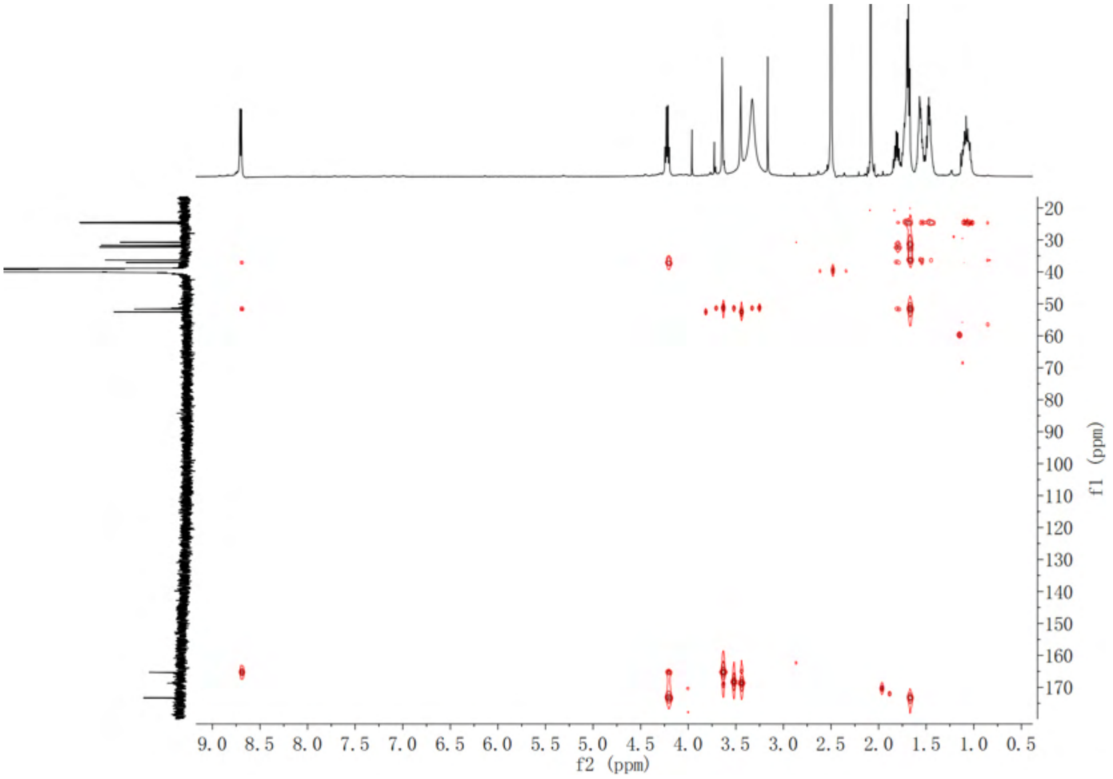

Supplementary Fig. 155. HMBC spectrum of compound (2*S*,3*S*)-*t*-ES-a7 in DMSO-*d*$_6$

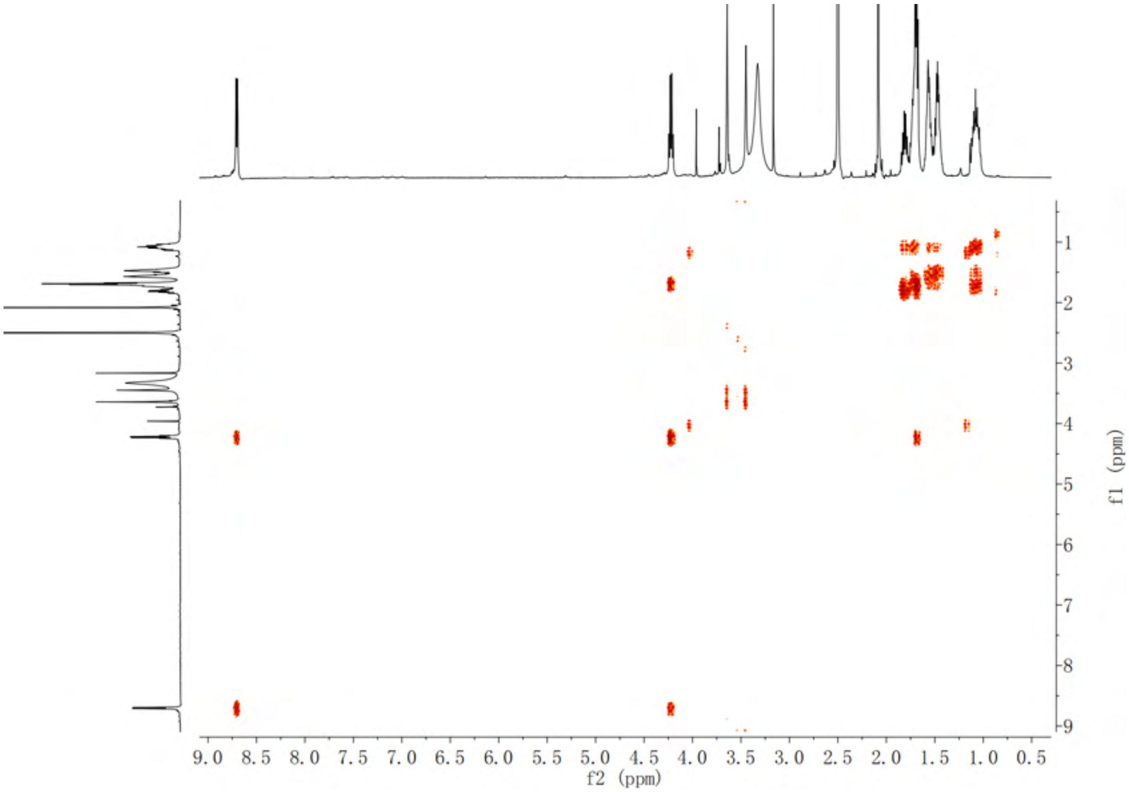

Supplementary Fig. 156. ^{1}H-^{1}H COSY spectrum of compound (2*S*,3*S*)-*t*-ES-a7 in DMSO-*d*$_6$

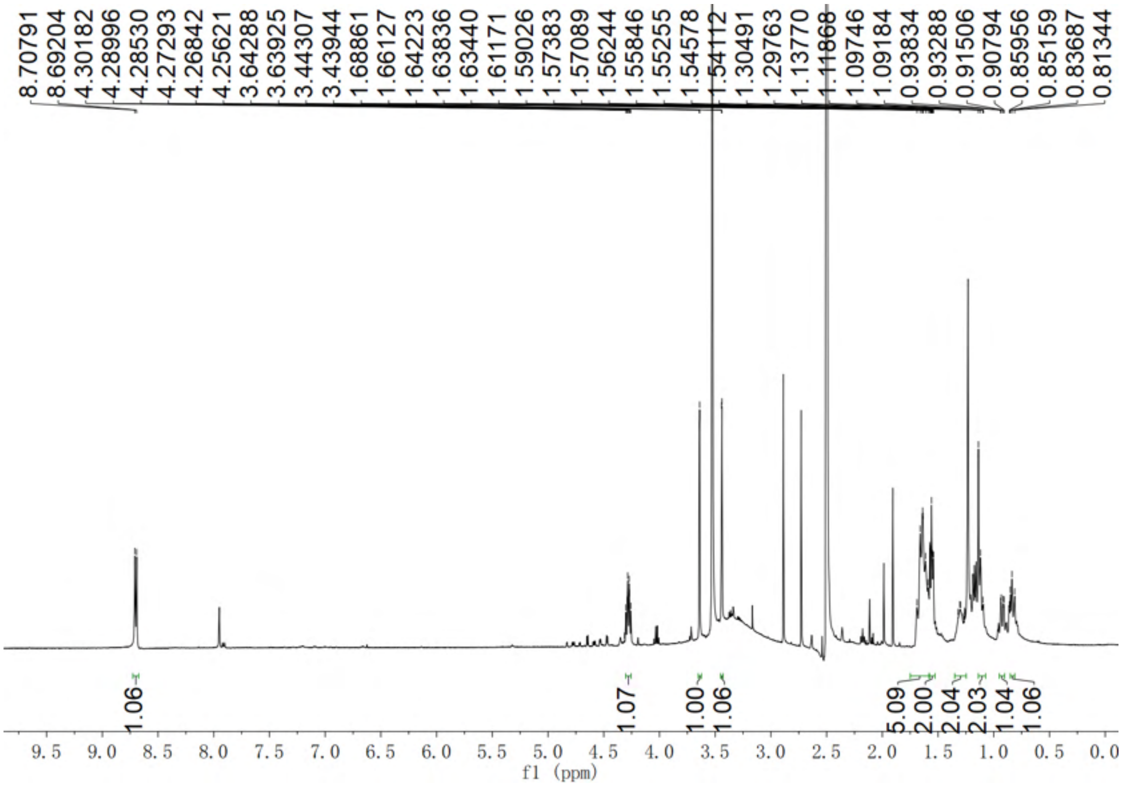

Supplementary Fig. 157. **^{1}H NMR spectrum of compound (2*S*,3*S*)-*t*-ES-a8 in DMSO-d_6**

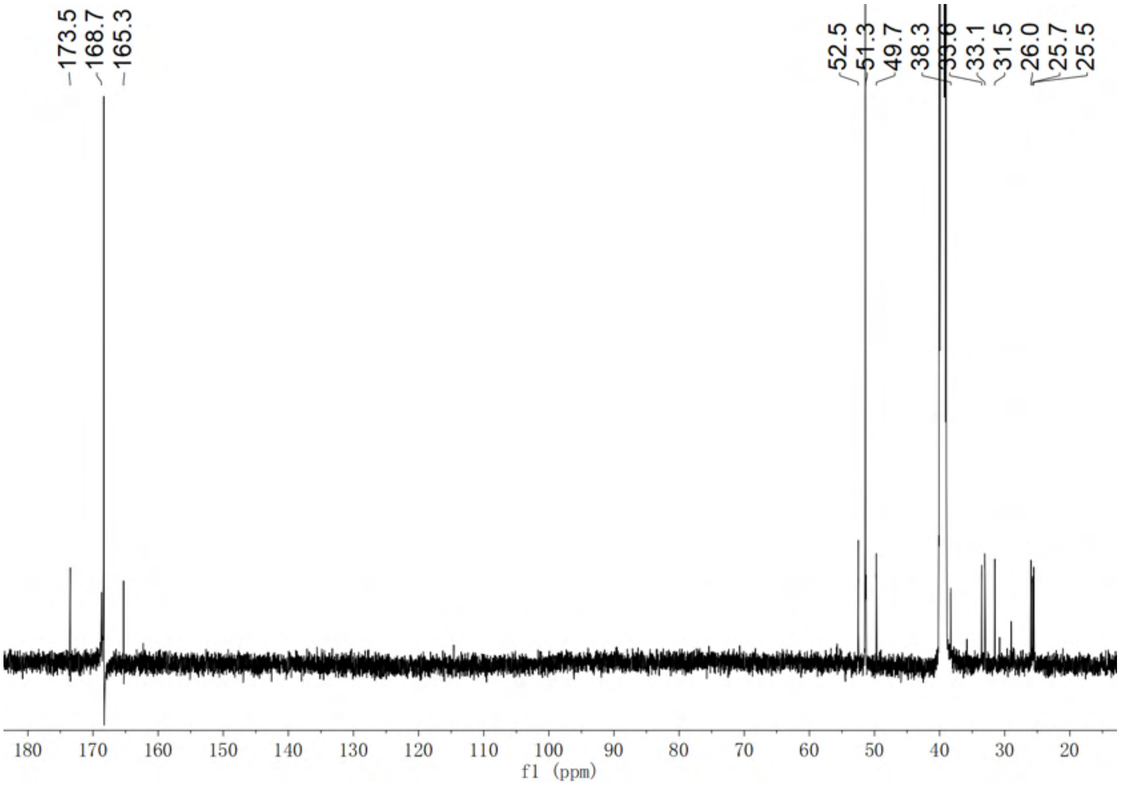

Supplementary Fig. 158. **^{13}C NMR spectrum of compound (2*S*,3*S*)-*t*-ES-a8 in DMSO-d_6**

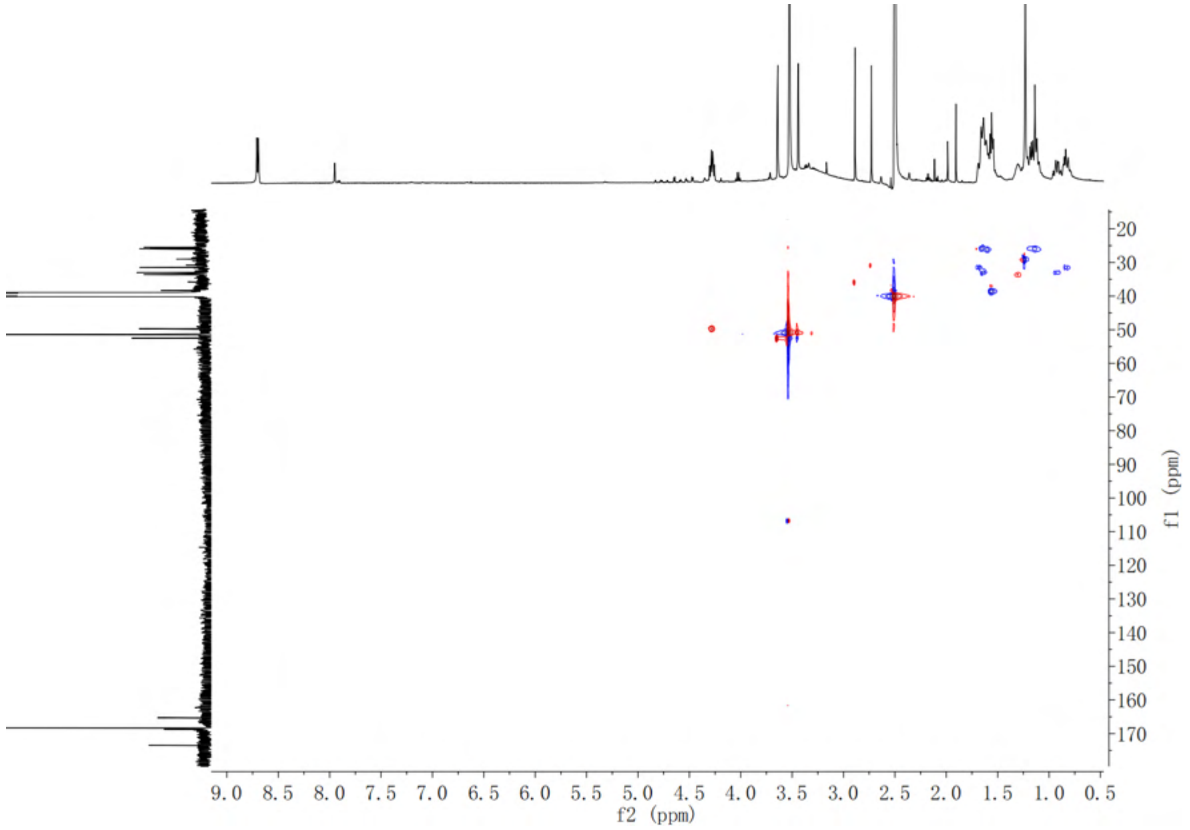

Supplementary Fig. 159. HSQC spectrum of compound (2*S*,3*S*)-*t*-ES-a8 in DMSO-d_6

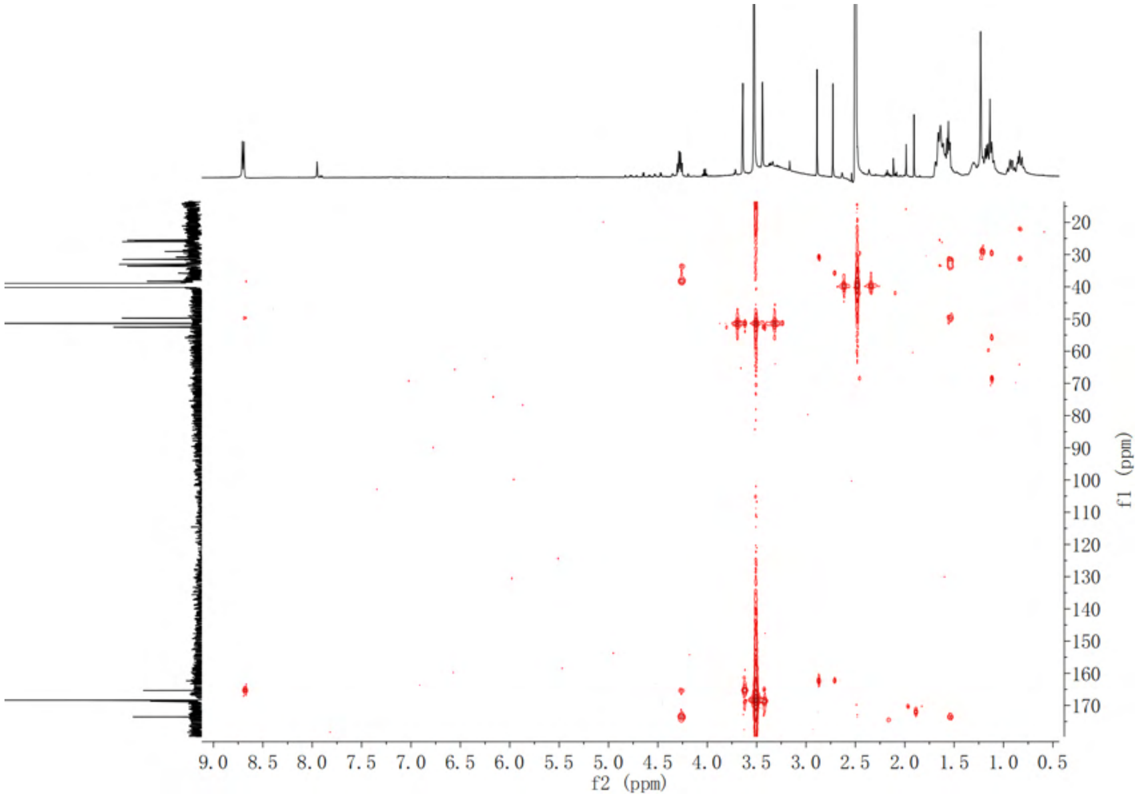

Supplementary Fig. 160. HMBC spectrum of compound (2*S*,3*S*)-*t*-ES-a8 in DMSO-d_6

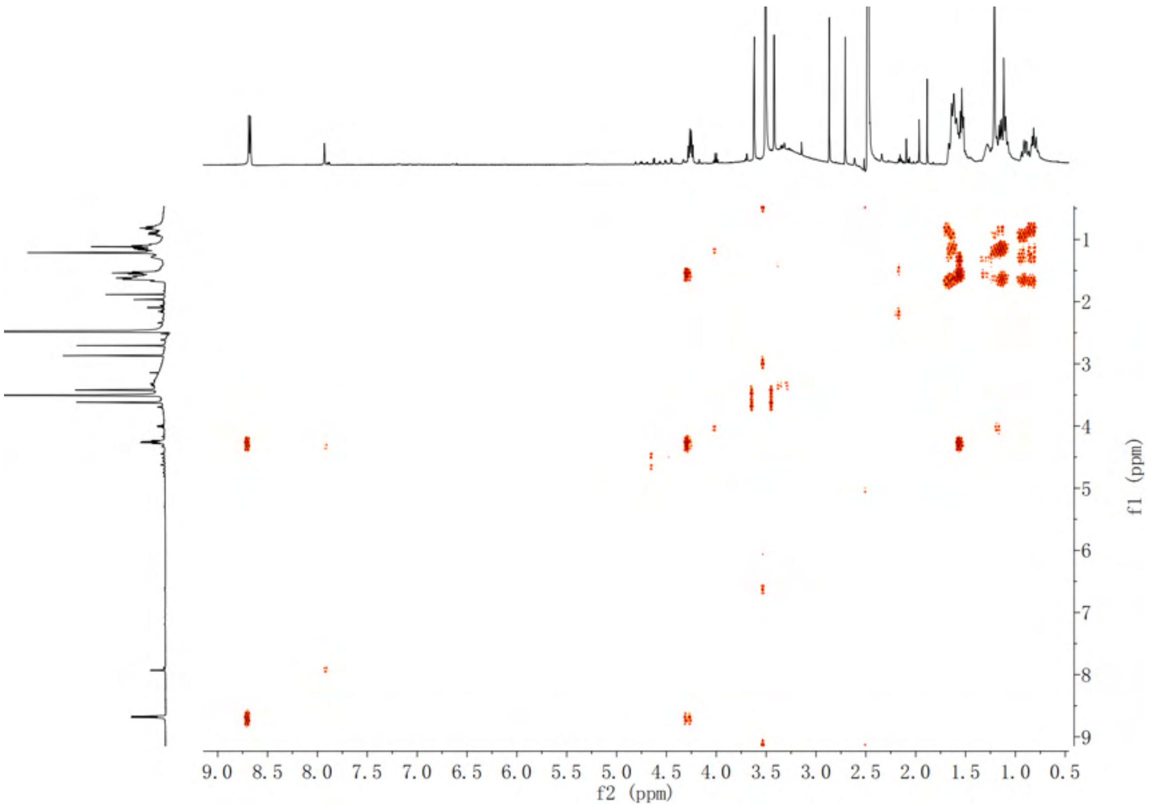

Supplementary Fig. 161. ^{1}**H-**^{1}**H COSY spectrum of compound (2*S*,3*S*)-*t*-ES-a8 in DMSO-*d*$_6$**

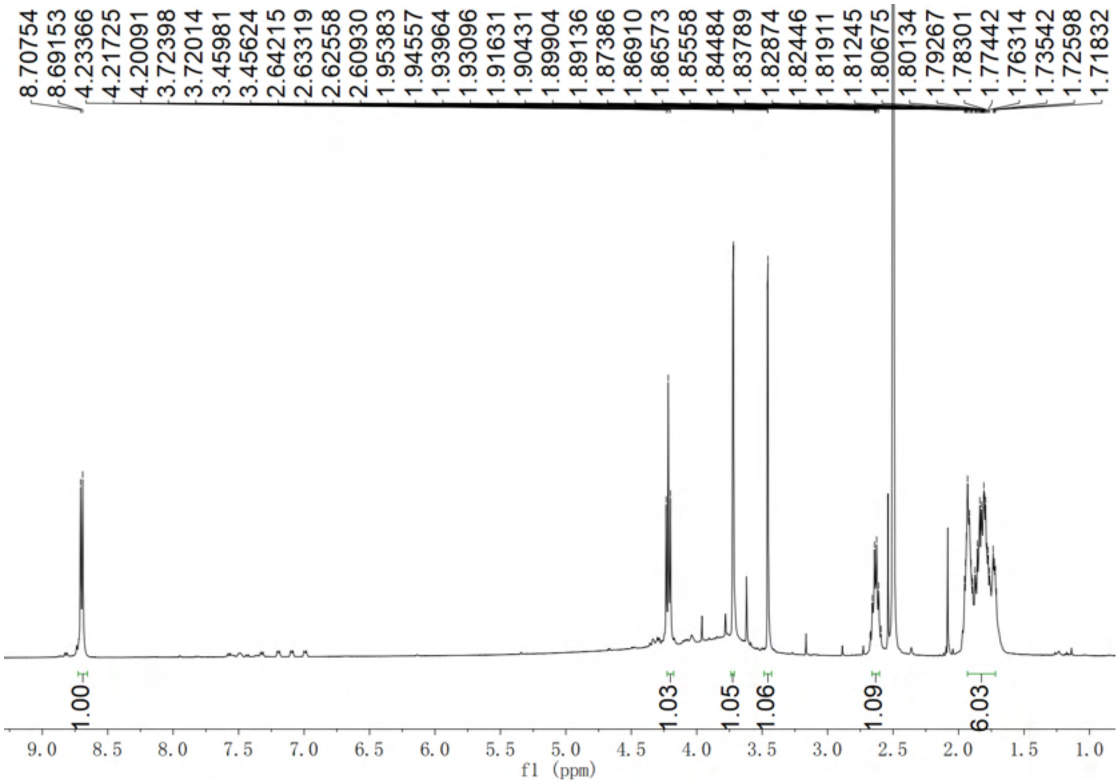

Supplementary Fig. 162. ^{1}**H NMR spectrum of compound (2*S*,3*S*)-*t*-ES-a9 in DMSO-*d*$_6$**

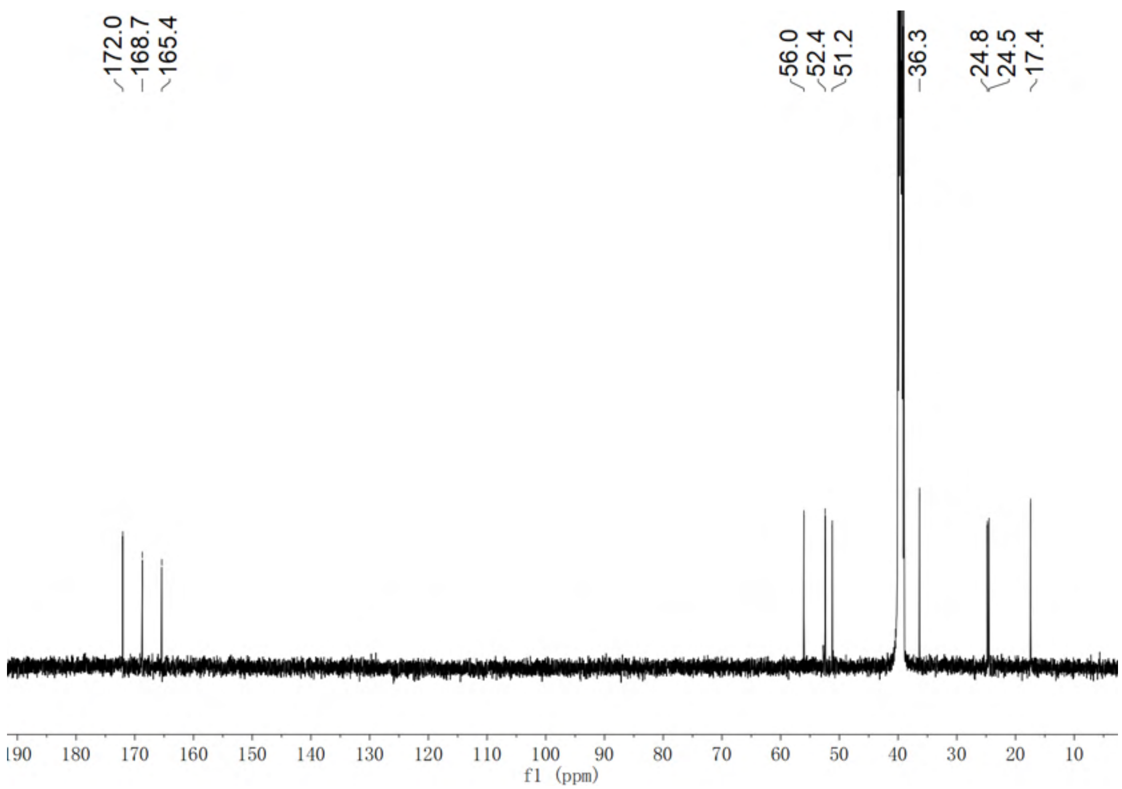

Supplementary Fig. 163. ^{13}C NMR spectrum of compound (2*S*,3*S*)-*t*-ES-a9 in DMSO-d_6

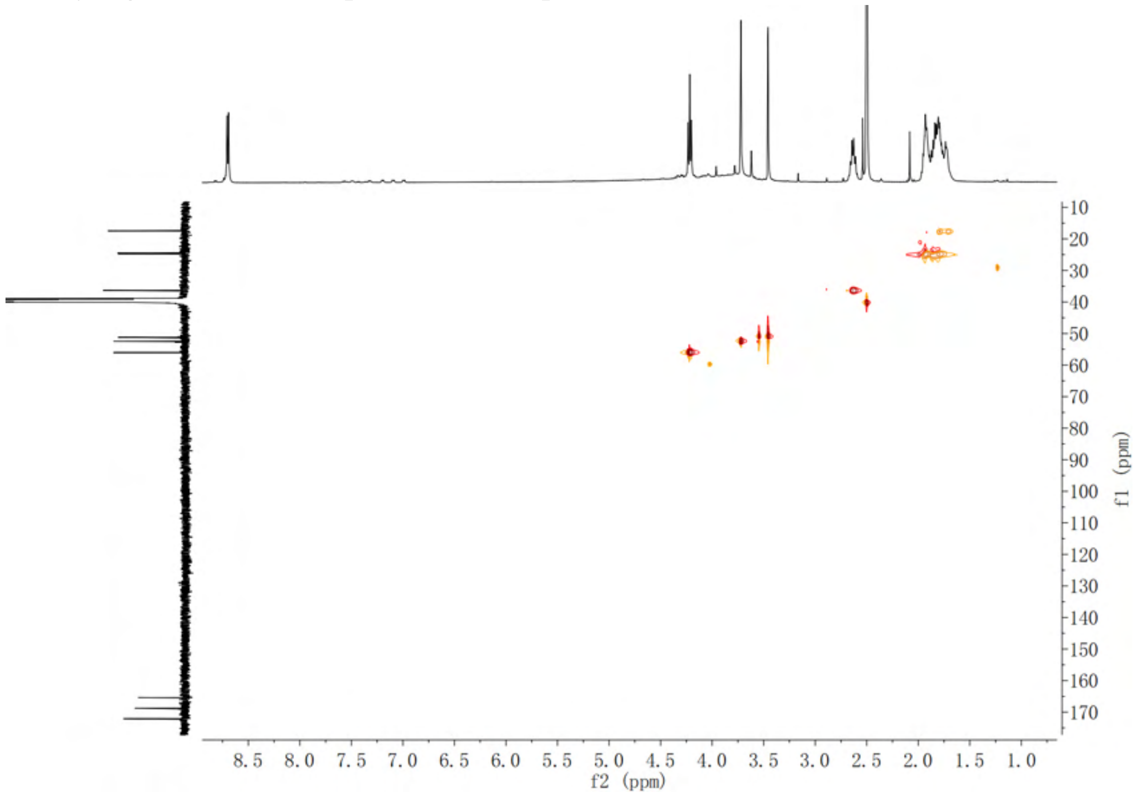

Supplementary Fig. 164. HSQC spectrum of compound (2*S*,3*S*)-*t*-ES-a9 in DMSO-d_6

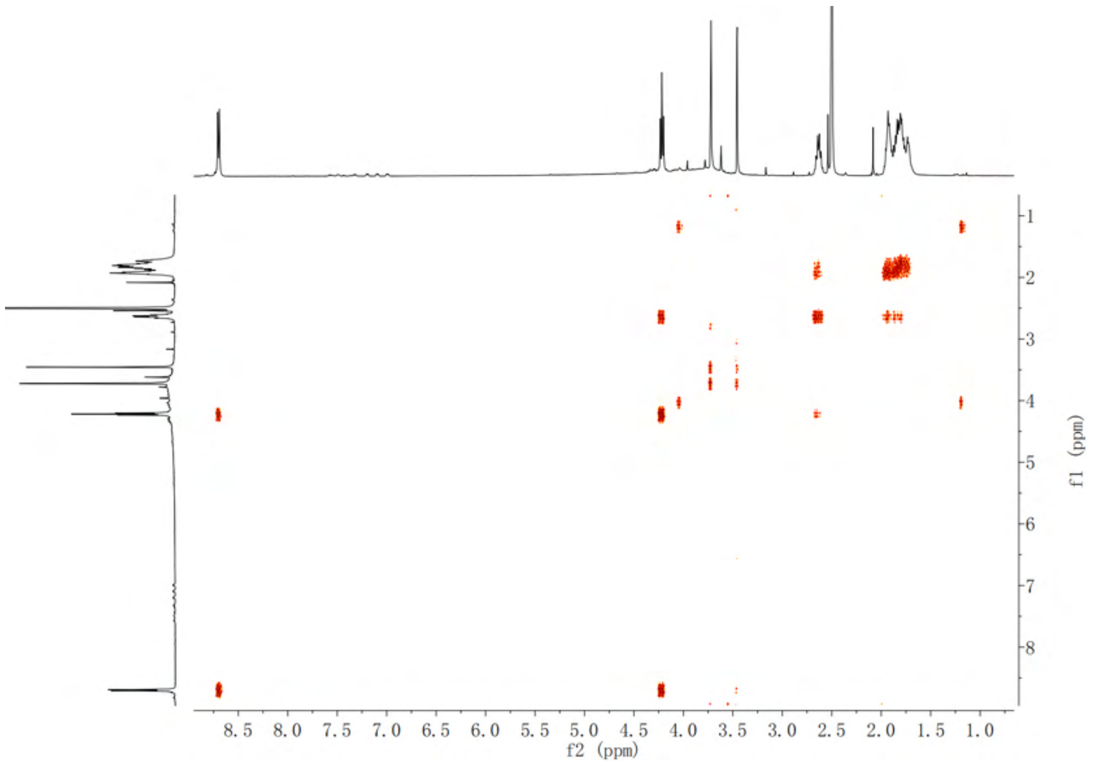

Supplementary Fig. 165. HMBC spectrum of compound (2*S*,3*S*)-*t*-ES-a9 in DMSO-d_6

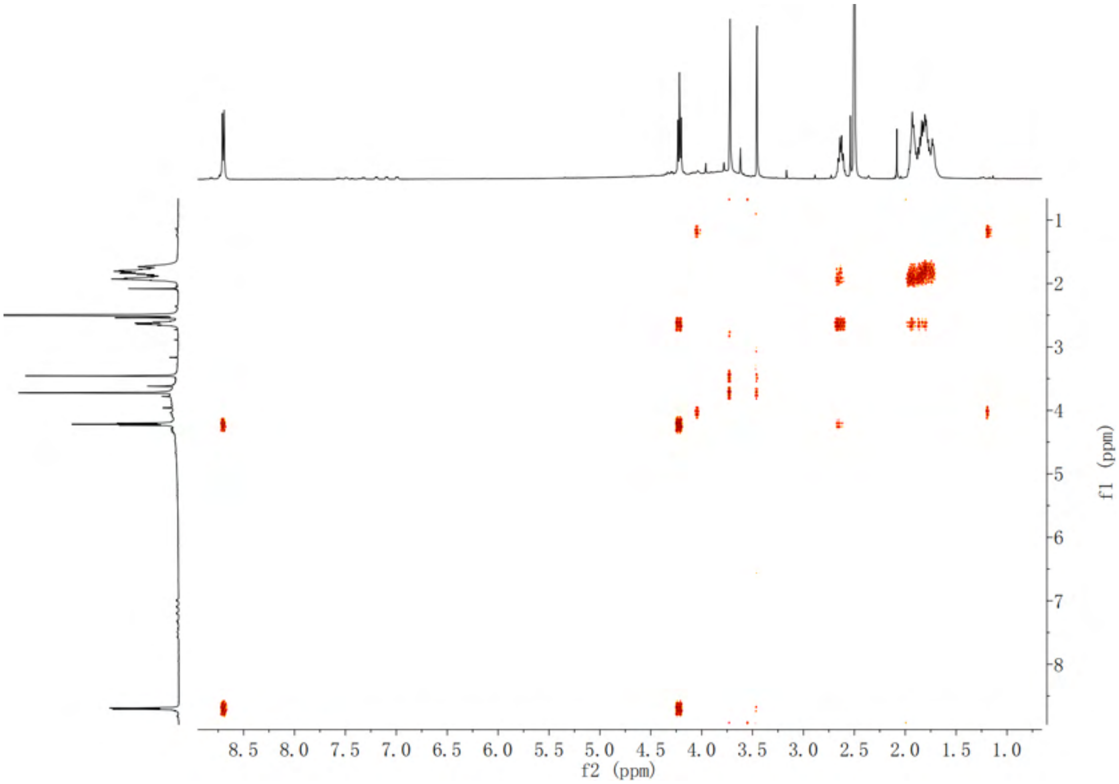

Supplementary Fig. 166. ^{1}H-^{1}H COSY spectrum of compound (2*S*,3*S*)-*t*-ES-a9 in DMSO-d_6

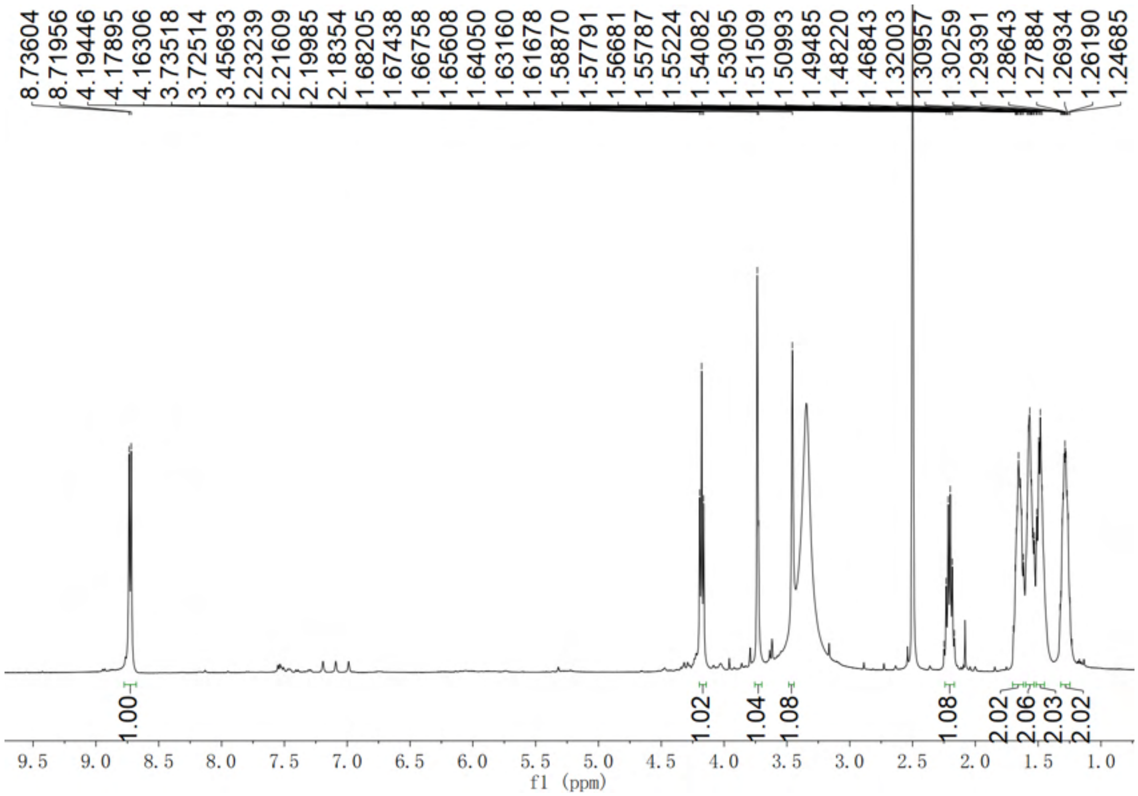

Supplementary Fig. 167. **^{1}H NMR spectrum of compound (2*S*,3*S*)-*t*-ES-a10 in DMSO-d_6**

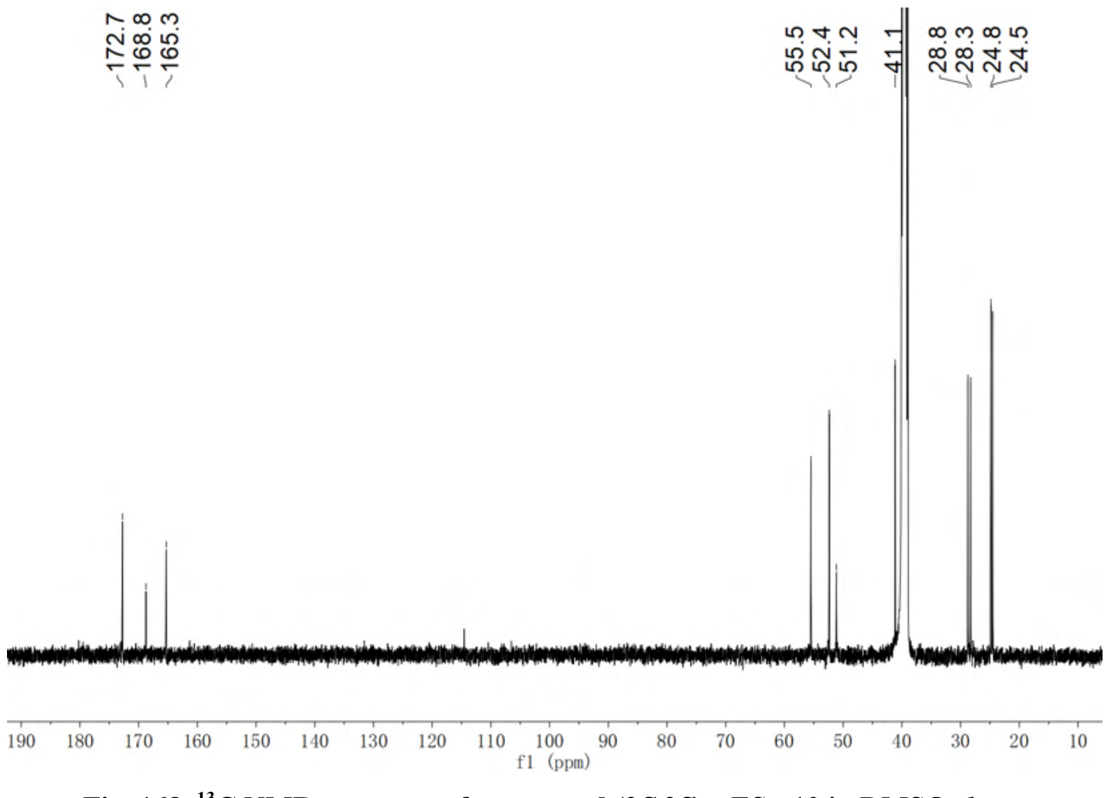

Supplementary Fig. 168. **^{13}C NMR spectrum of compound (2*S*,3*S*)-*t*-ES-a10 in DMSO-d_6**

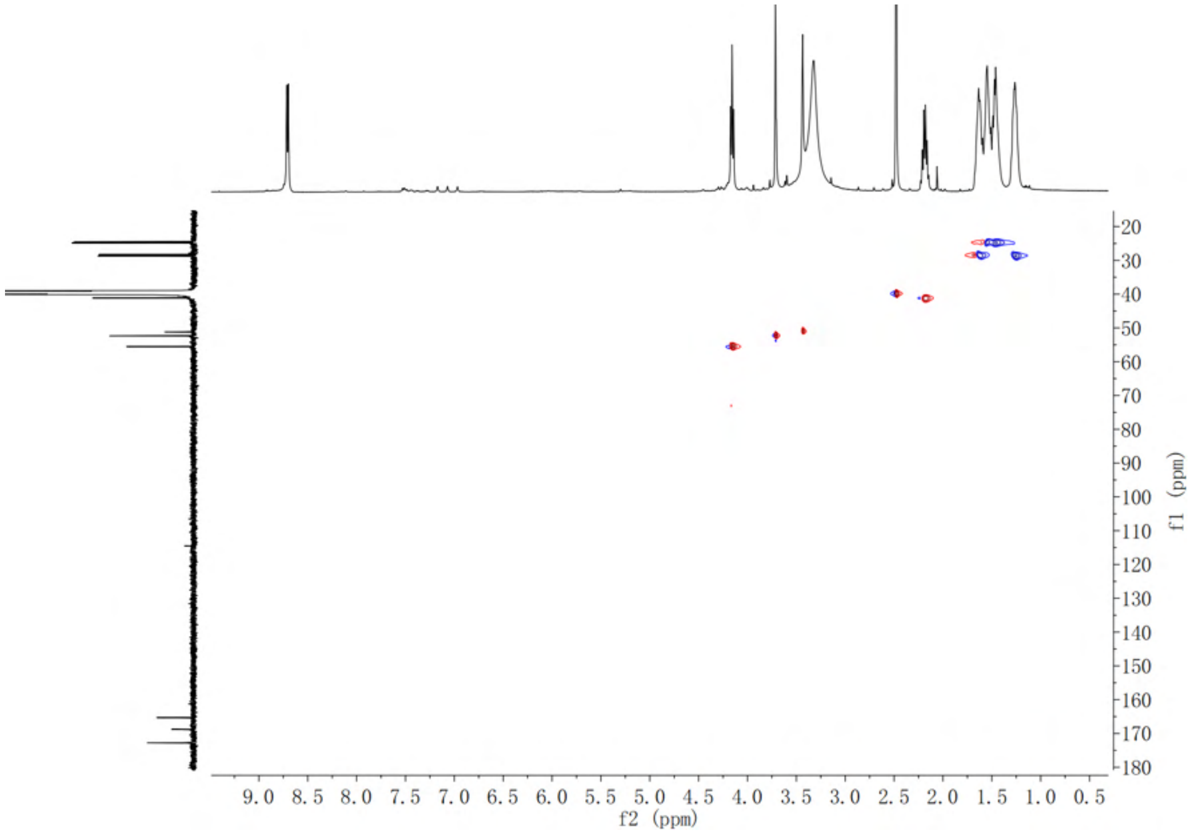

Supplementary Fig. 169. HSQC spectrum of compound (2*S*,3*S*)-*t*-ES-a10 in DMSO-*d*$_6$

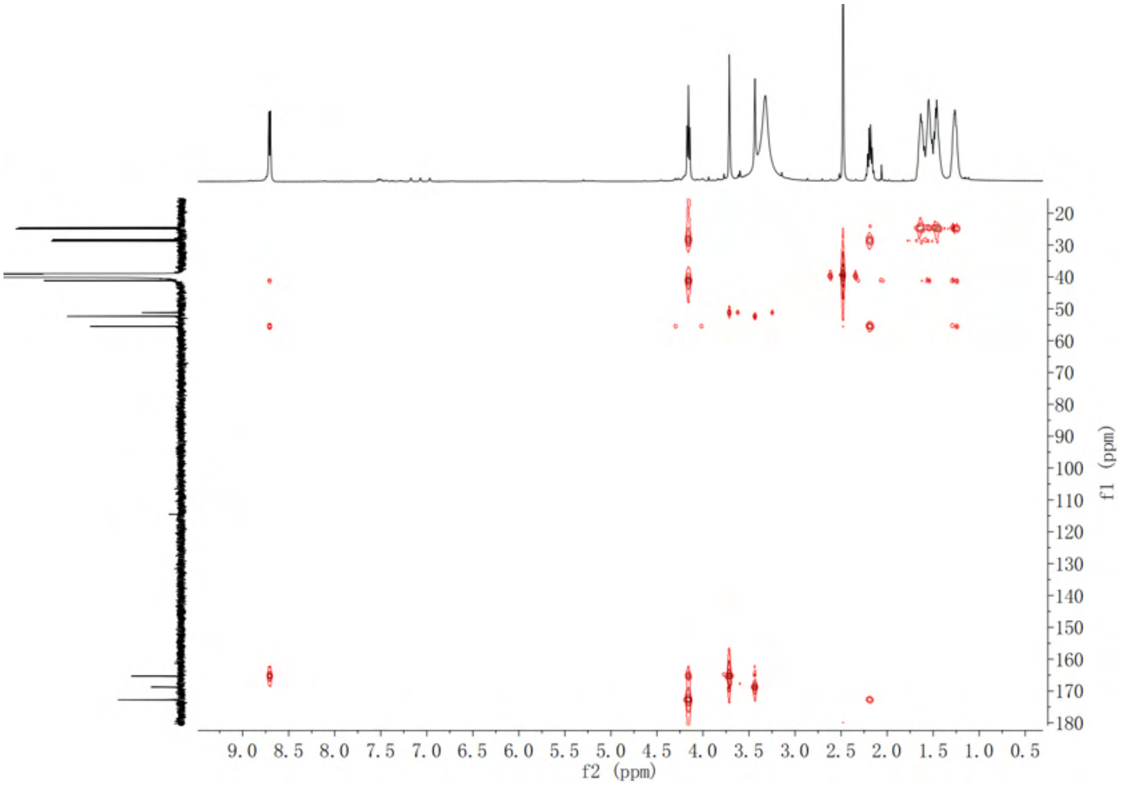

Supplementary Fig. 170. HMBC spectrum of compound (2*S*,3*S*)-*t*-ES-a10 in DMSO-*d*$_6$

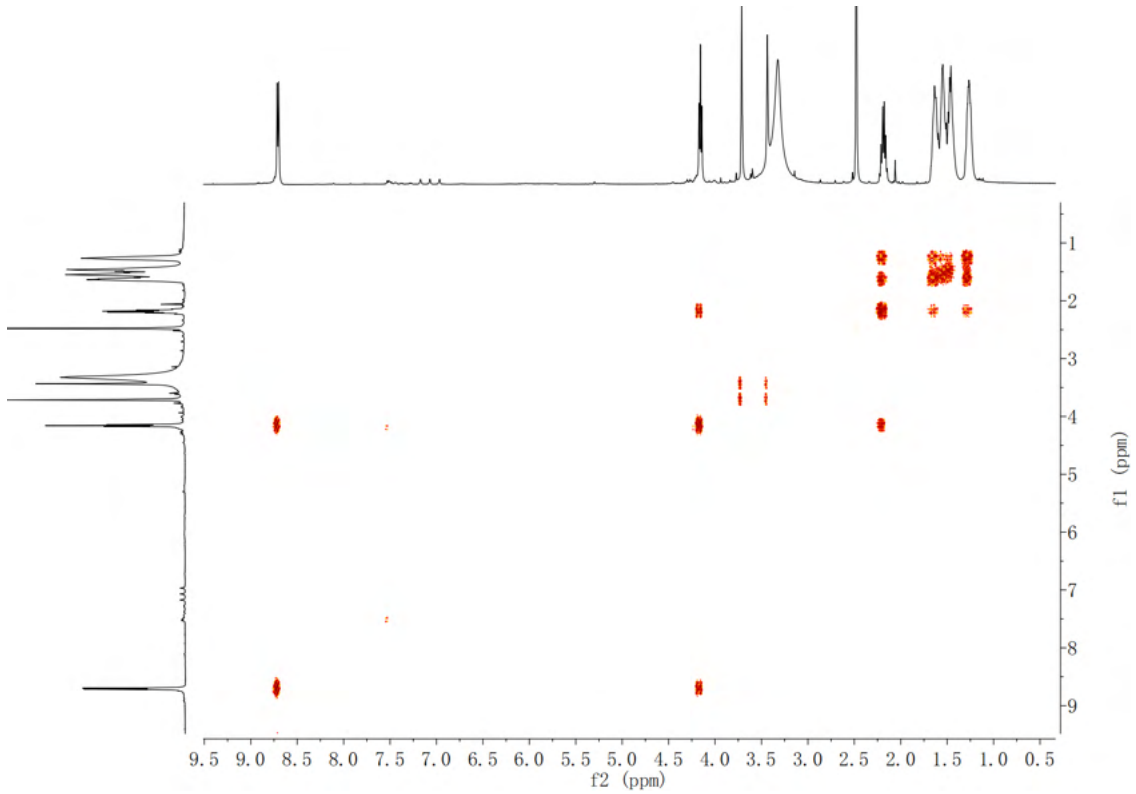

Supplementary Fig. 171. ^{1}H-^{1}H COSY spectrum of compound (2*S*,3*S*)-*t*-ES-a10 in DMSO-d_6

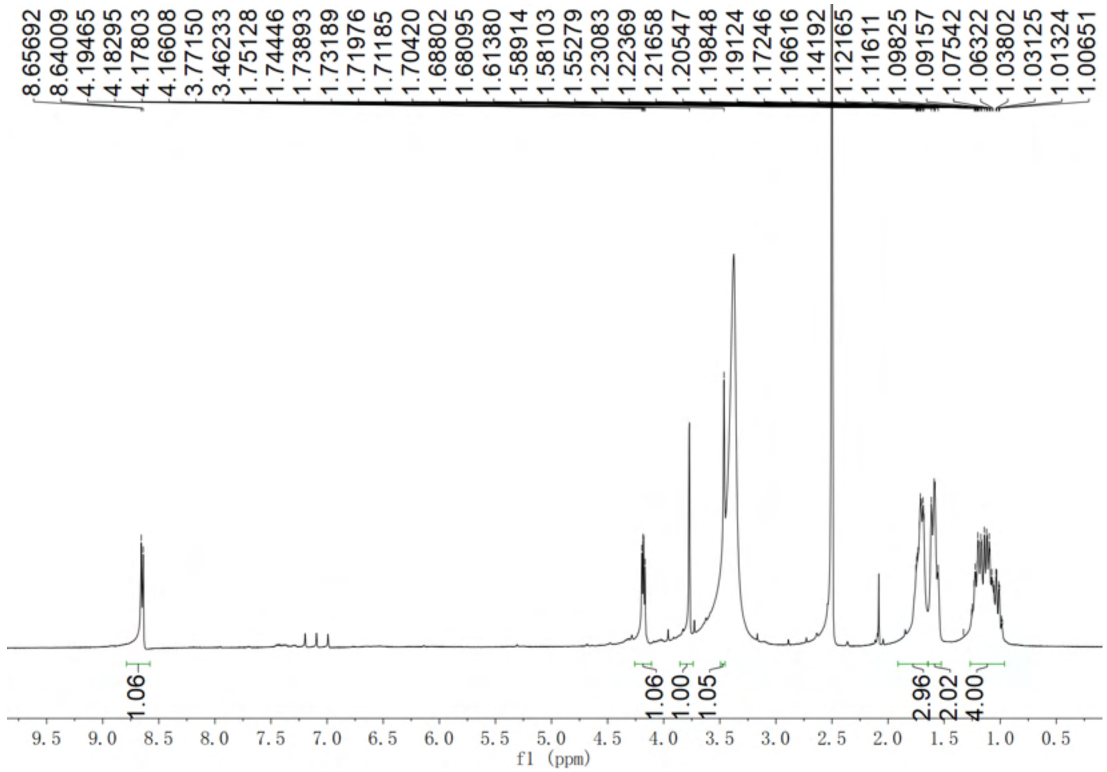

Supplementary Fig. 172. ^{1}H NMR spectrum of compound (2*S*,3*S*)-*t*-ES-a11 in DMSO-d_6

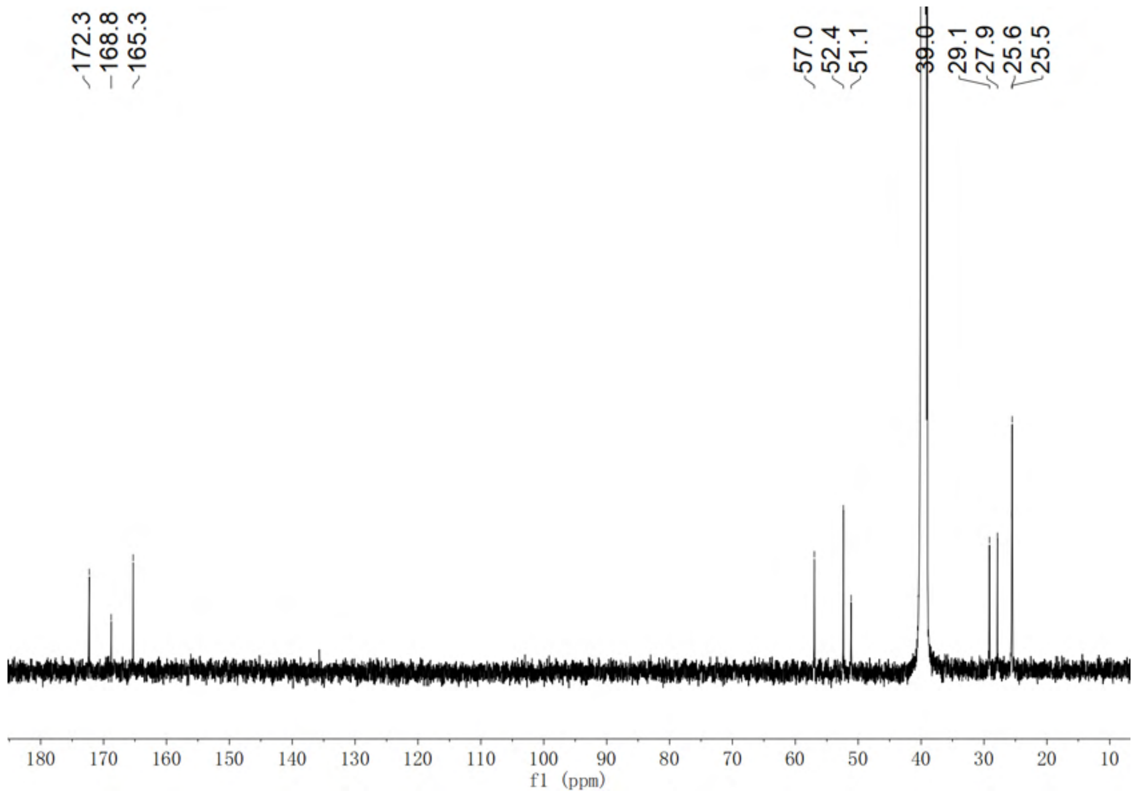

Supplementary Fig. 173. ^{13}**C NMR spectrum of compound (2*S*,3*S*)-*t*-ES-a11 in DMSO-*d*$_6$**

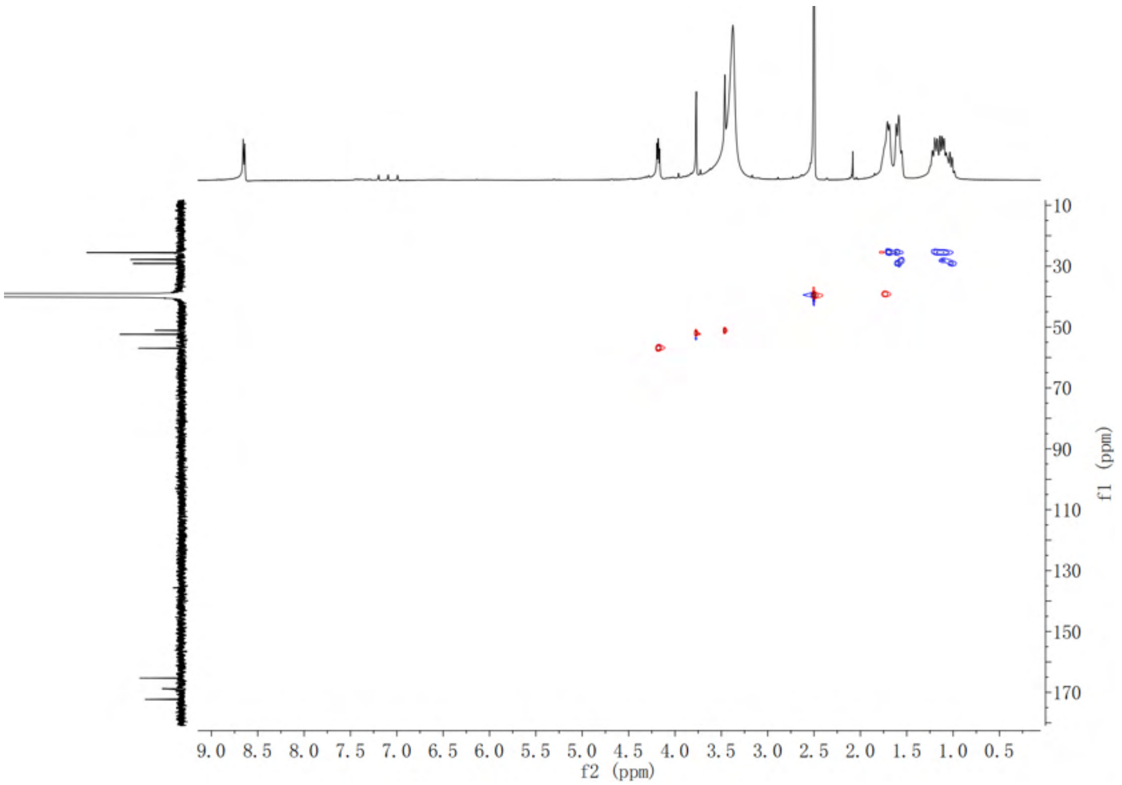

Supplementary Fig. 174. HSQC spectrum of compound (2*S*,3*S*)-*t*-ES-a11 in DMSO-*d*$_6$

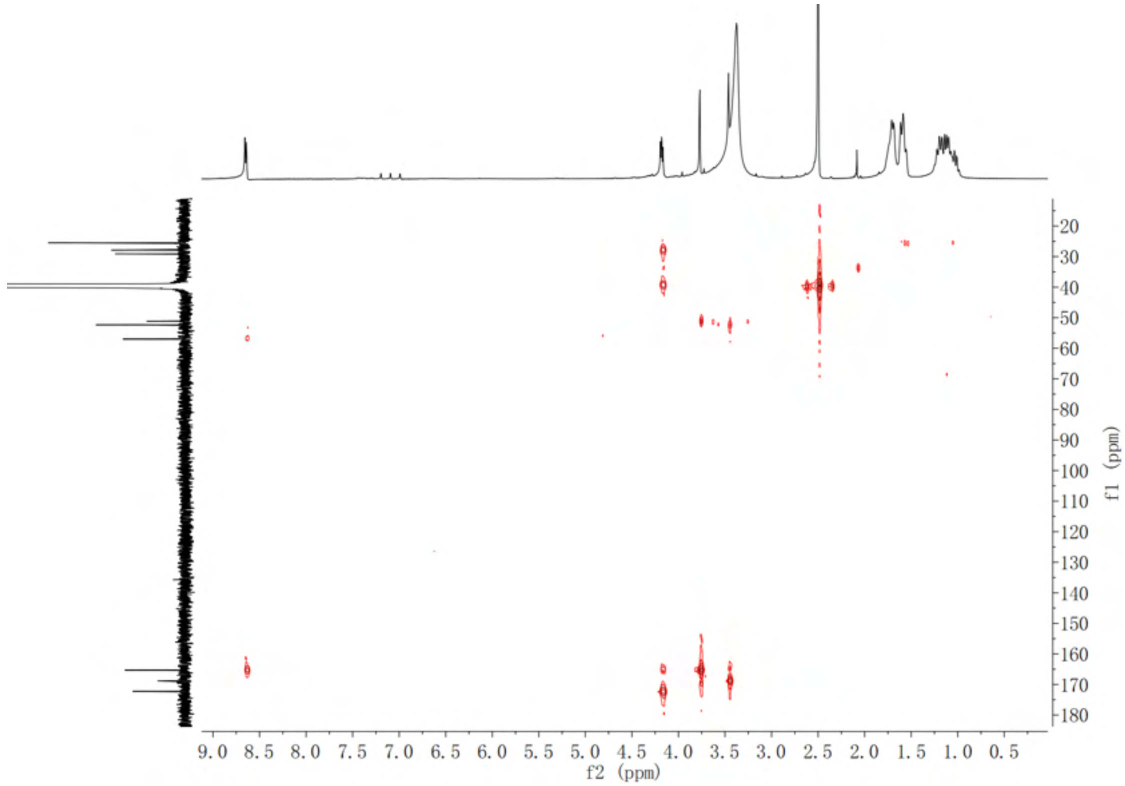

Supplementary Fig. 175. HMBC spectrum of compound (2*S*,3*S*)-*t*-ES-a11 in DMSO-d_6

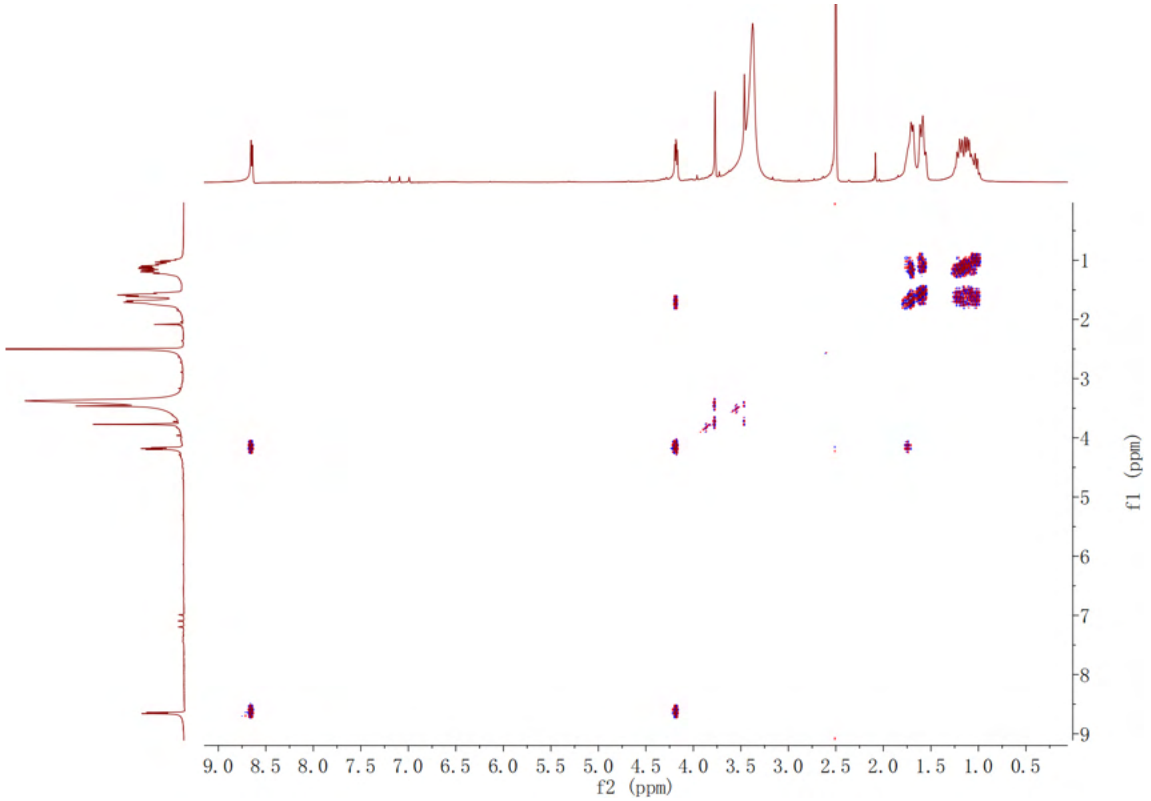

Supplementary Fig. 176. ^{1}H-^{1}H COSY spectrum of compound (2*S*,3*S*)-*t*-ES-a11 in DMSO-d_6

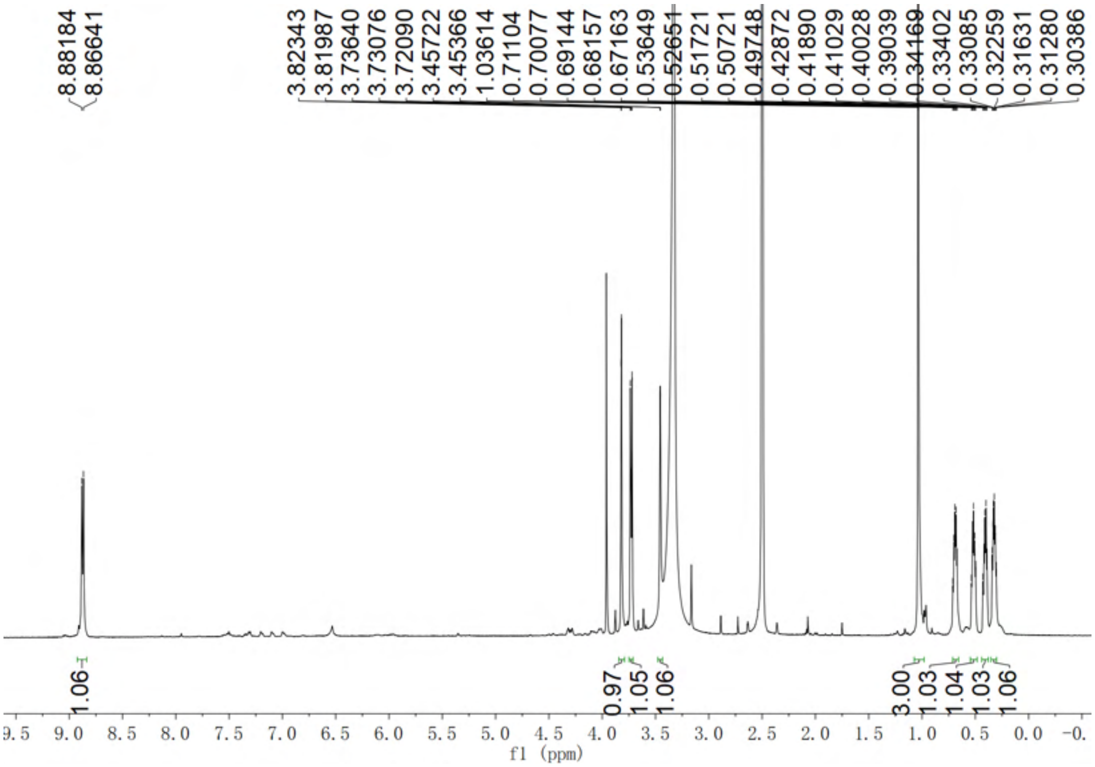

Supplementary Fig. 177. **^{1}H NMR spectrum of compound (2*S*,3*S*)-*t*-ES-a12 in DMSO-*d*$_6$**

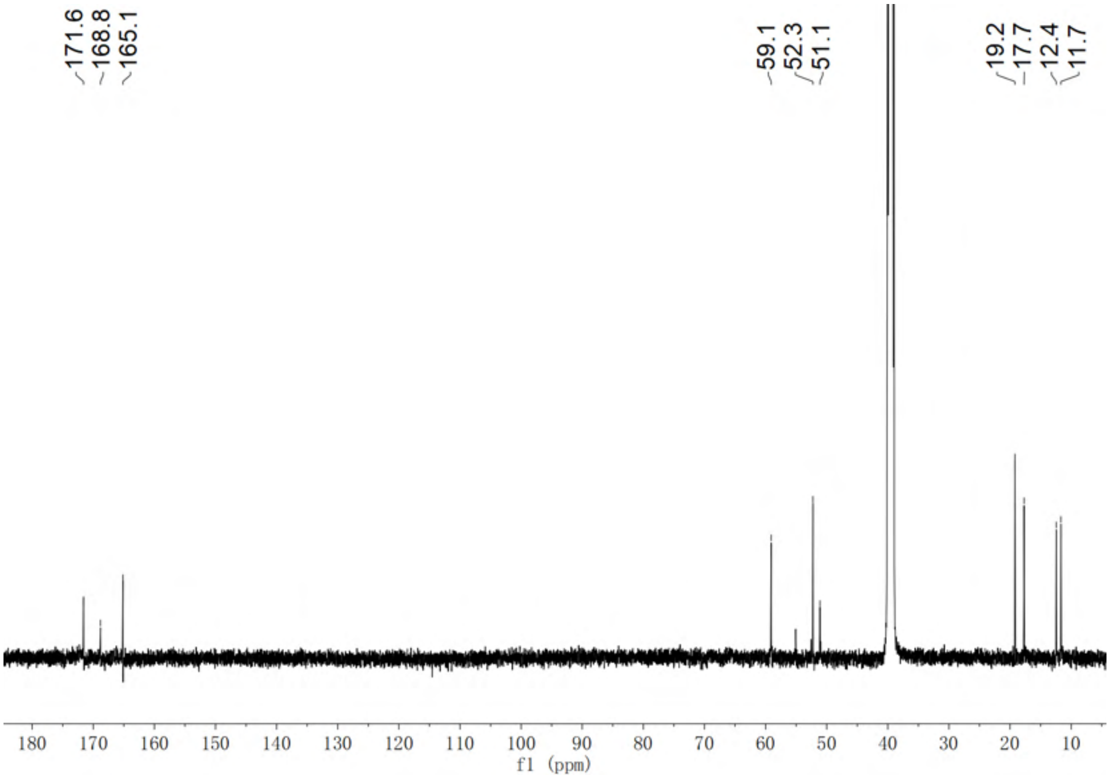

Supplementary Fig. 178. **^{13}C NMR spectrum of compound (2*S*,3*S*)-*t*-ES-a12 in DMSO-*d*$_6$**

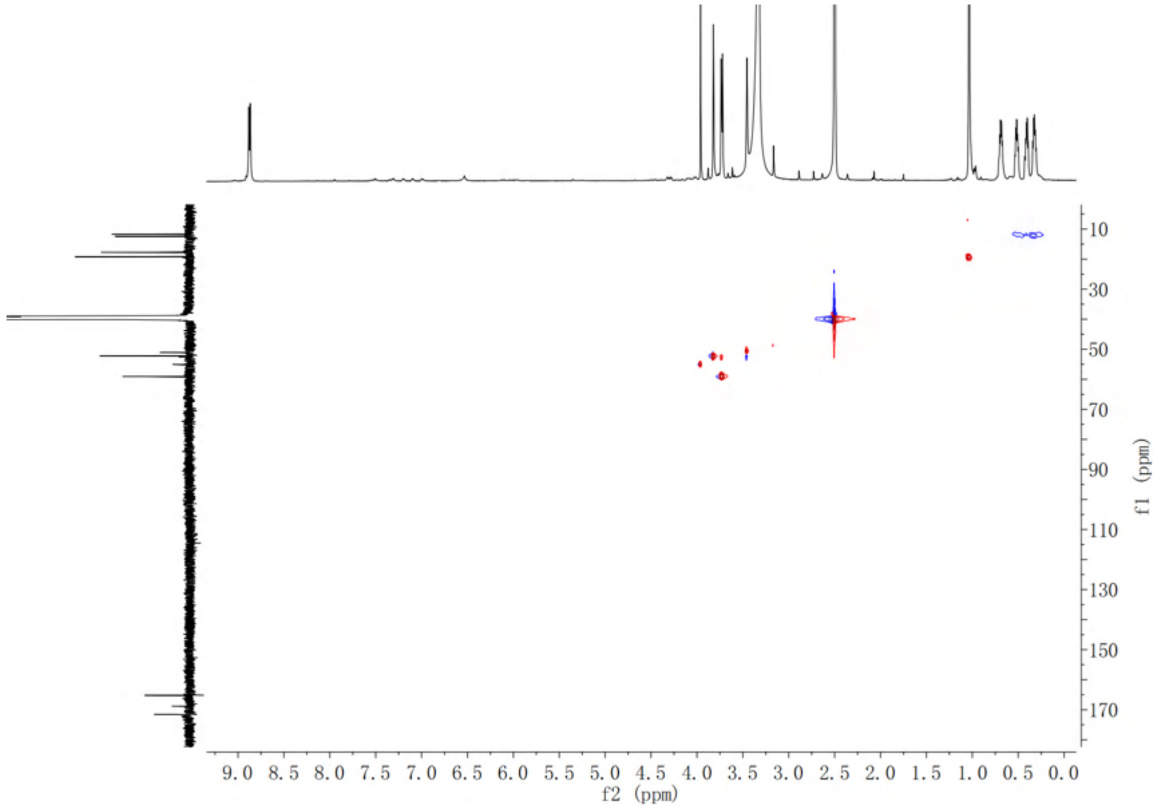

Supplementary Fig. 179. HSQC spectrum of compound (2*S*,3*S*)-*t*-ES-a12 in DMSO-d_6

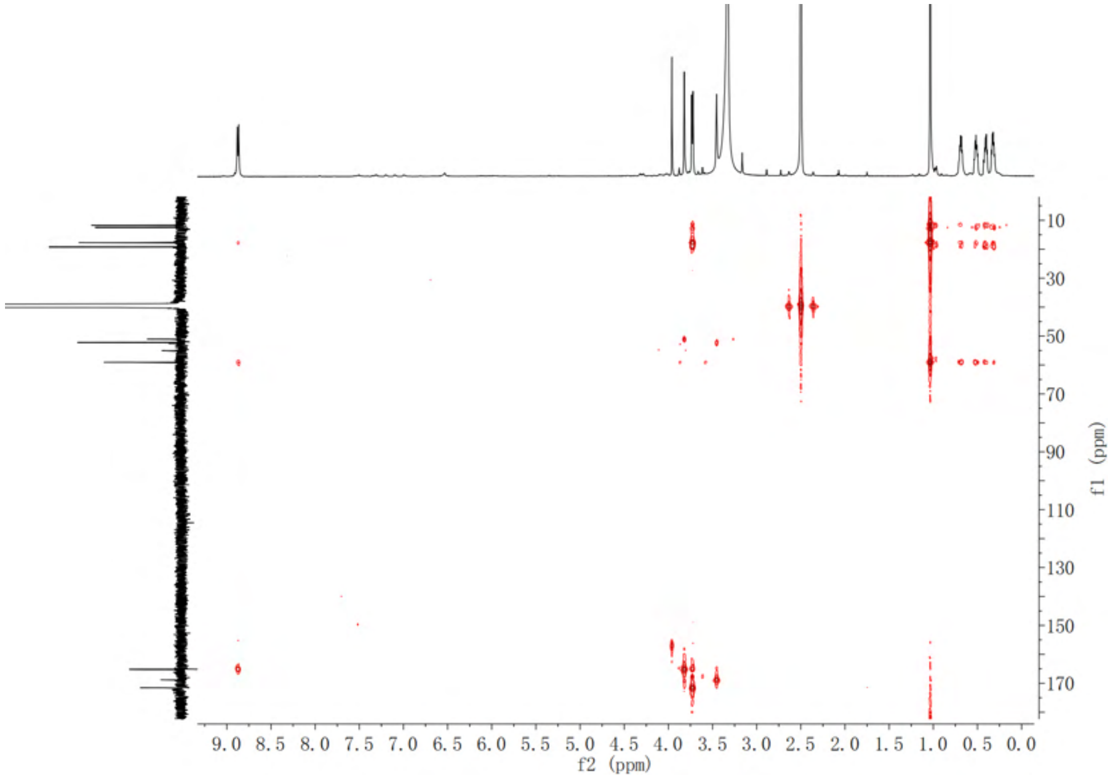

Supplementary Fig. 180. HMBC spectrum of compound (2*S*,3*S*)-*t*-ES-a12 in DMSO-d_6

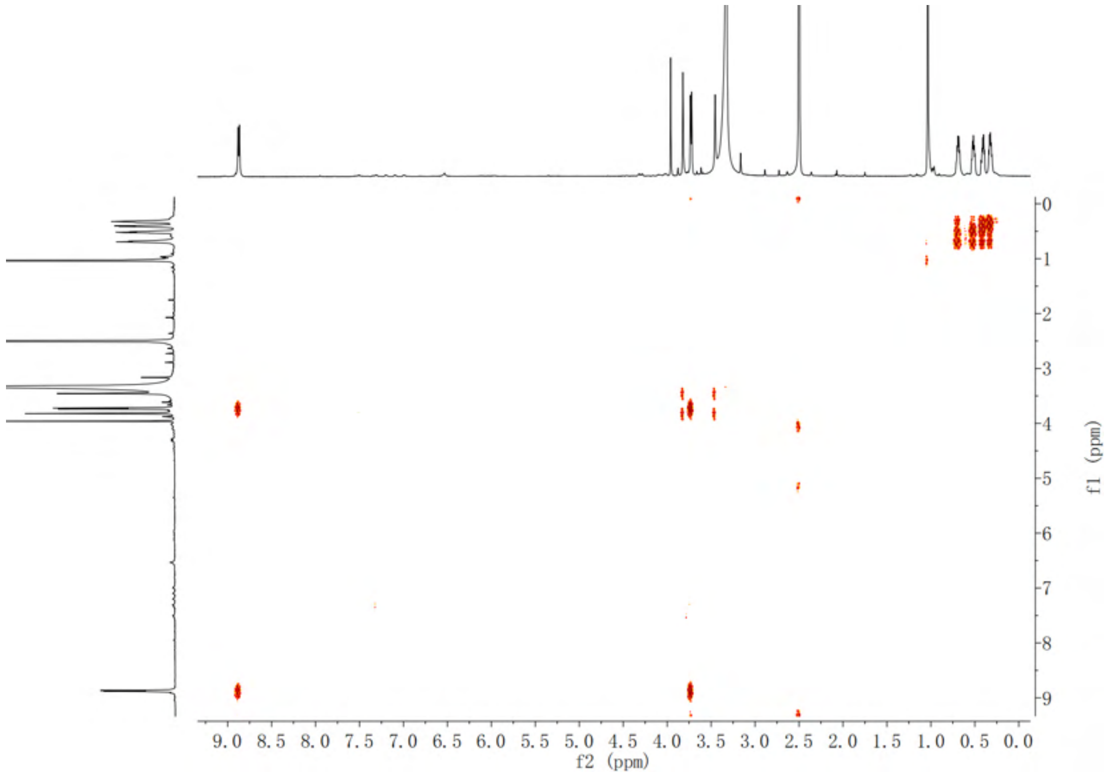

Supplementary Fig. 181. ^{1}H-^{1}H COSY spectrum of compound (2*S*,3*S*)-*t*-ES-a12 in DMSO-d_6

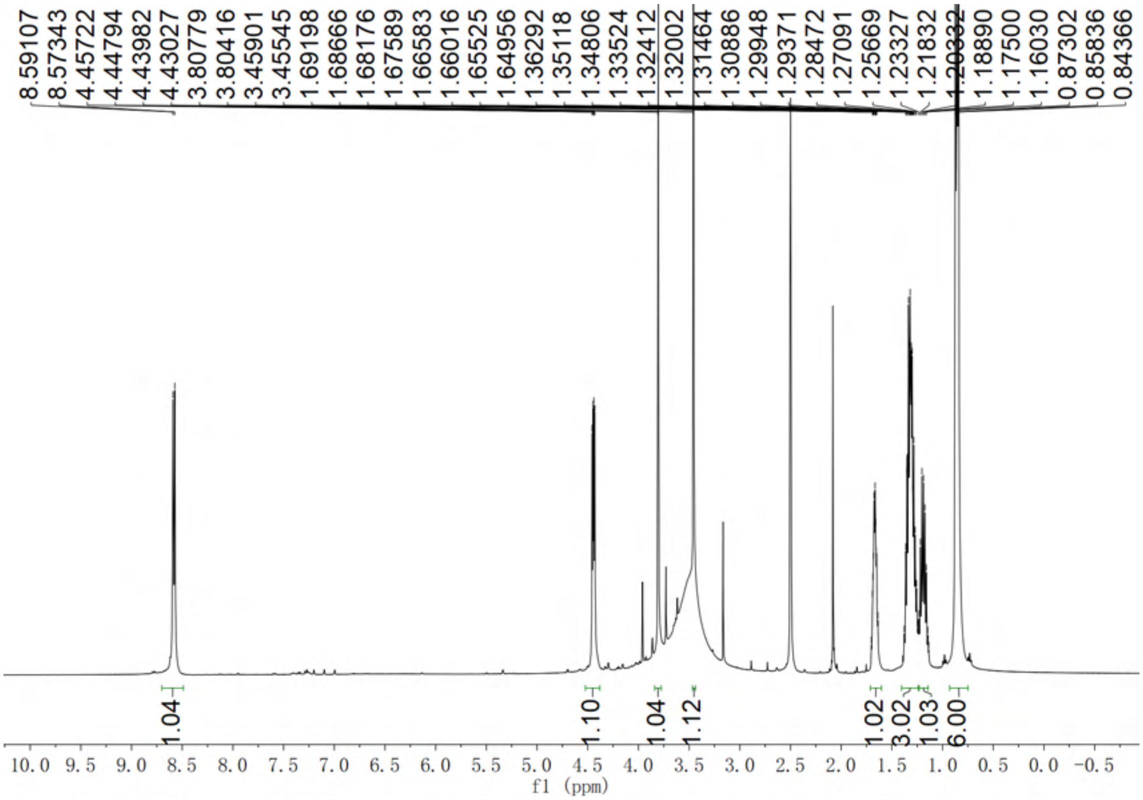

Supplementary Fig. 182. ^{1}H NMR spectrum of compound (2*S*,3*S*)-*t*-ES-a13 in DMSO-d_6

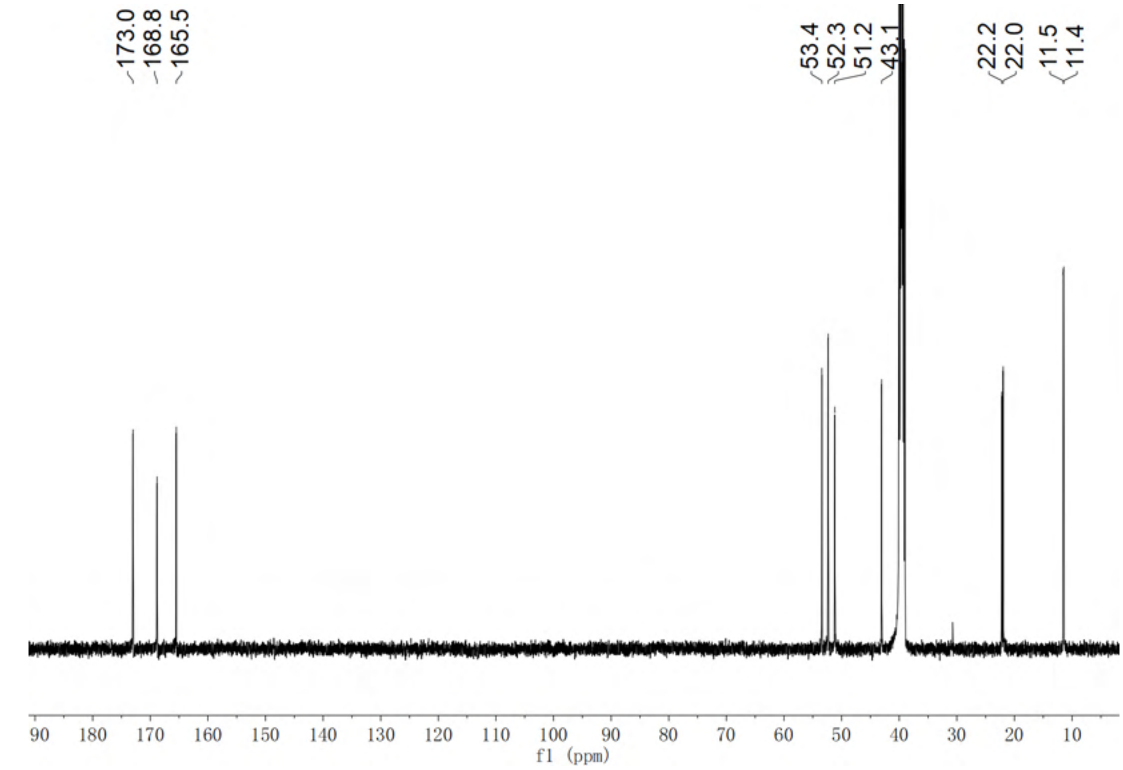

Supplementary Fig. 183. ^{13}C NMR spectrum of compound (2*S*,3*S*)-*t*-ES-a13 in DMSO-d_6

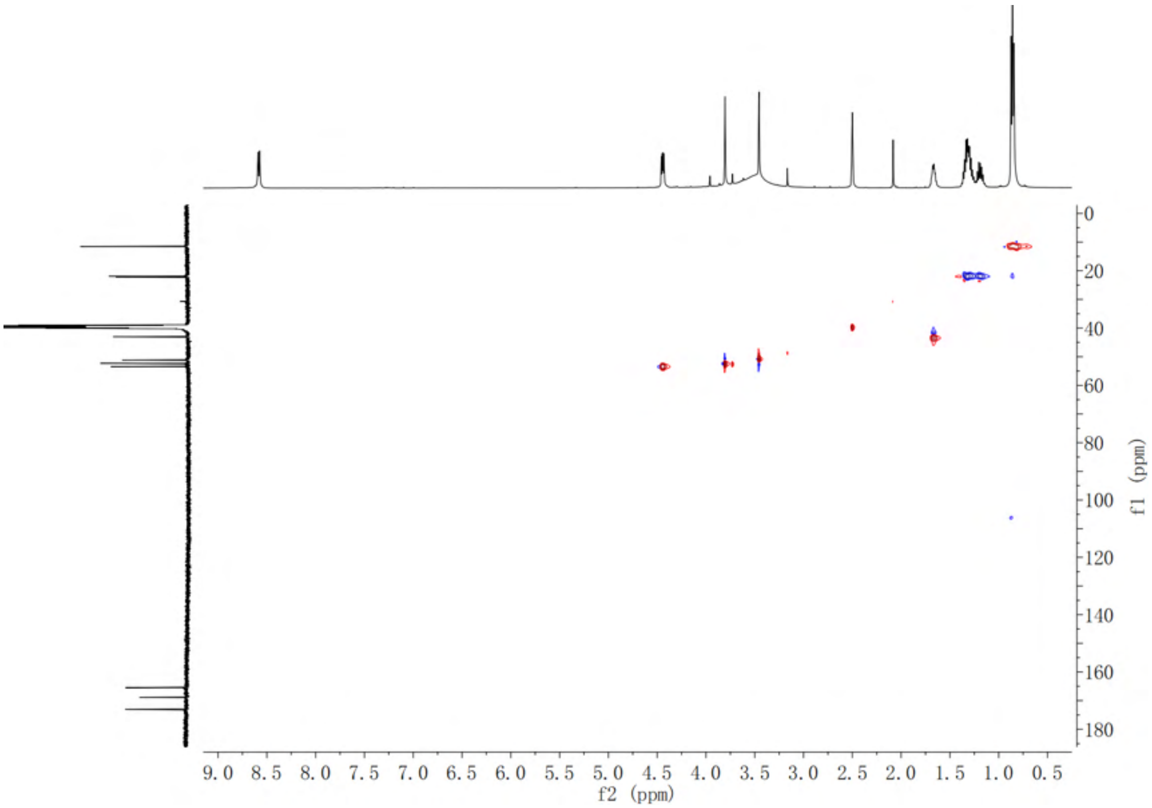

Supplementary Fig. 184. HSQC spectrum of compound (2*S*,3*S*)-*t*-ES-a13 in DMSO-d_6

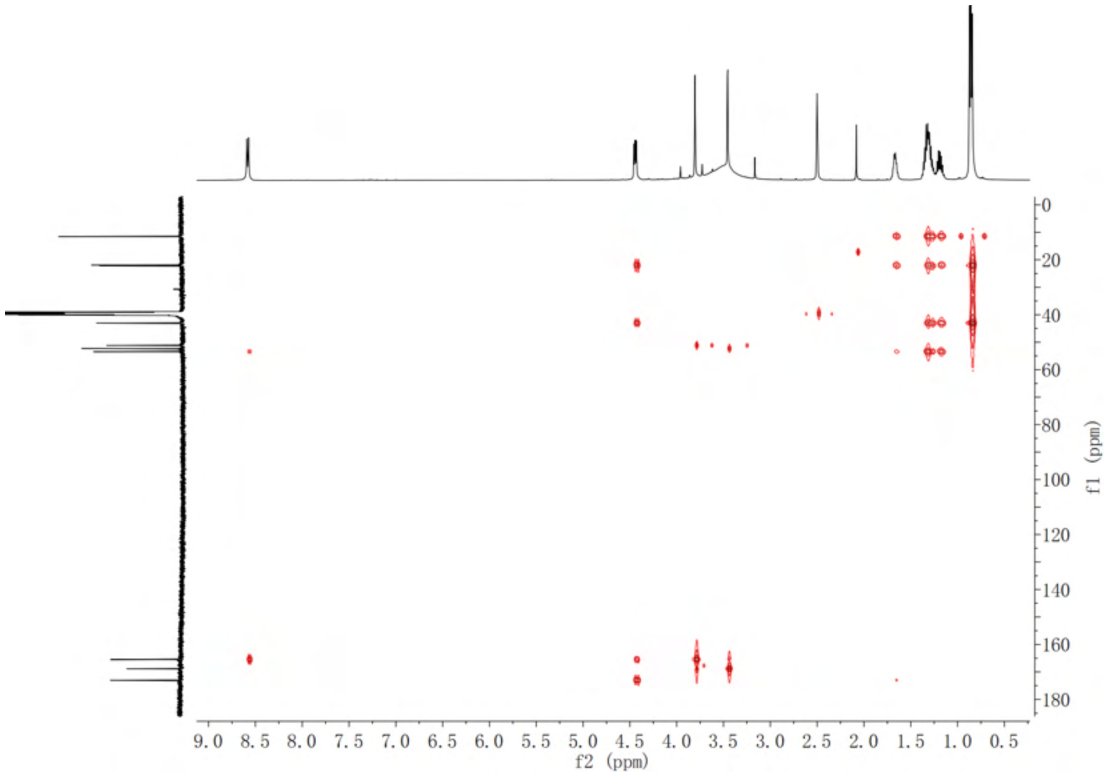

Supplementary Fig. 185. HMBC spectrum of compound (2*S*,3*S*)-*t*-ES-a13 in DMSO-*d*$_6$

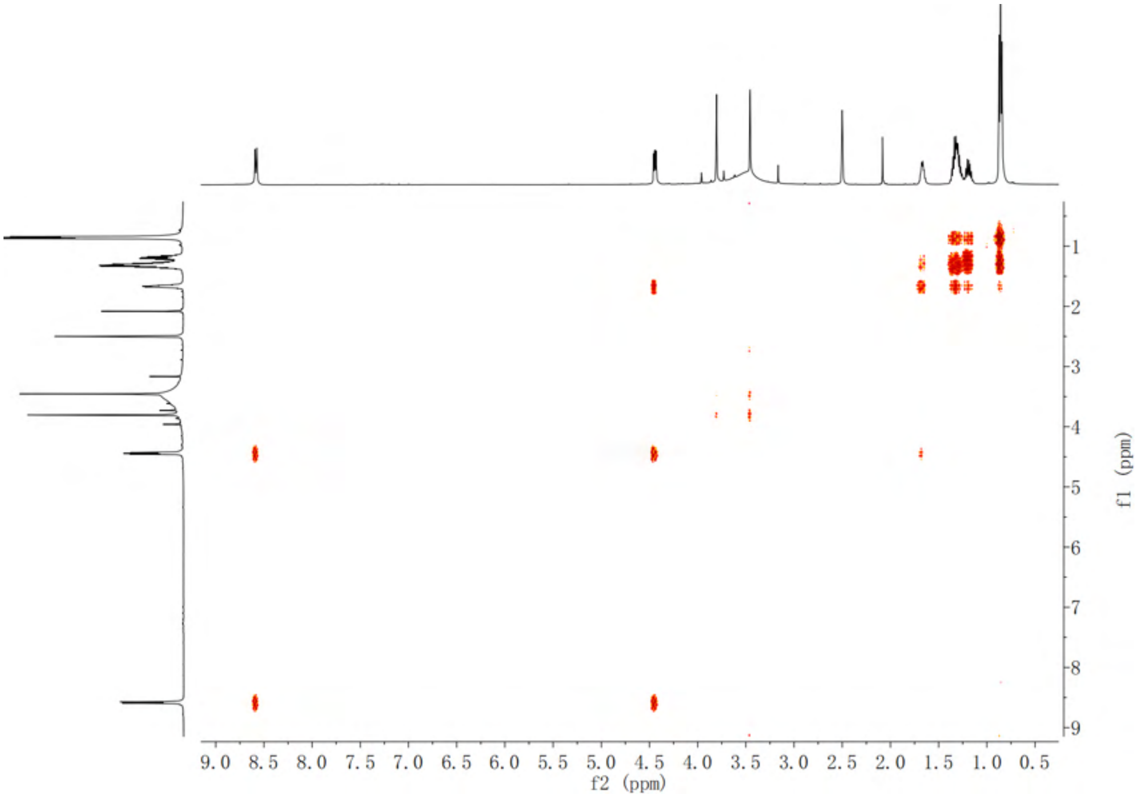

Supplementary Fig. 186. ^{1}H-^{1}H COSY spectrum of compound (2*S*,3*S*)-*t*-ES-a13 in DMSO-*d*$_6$

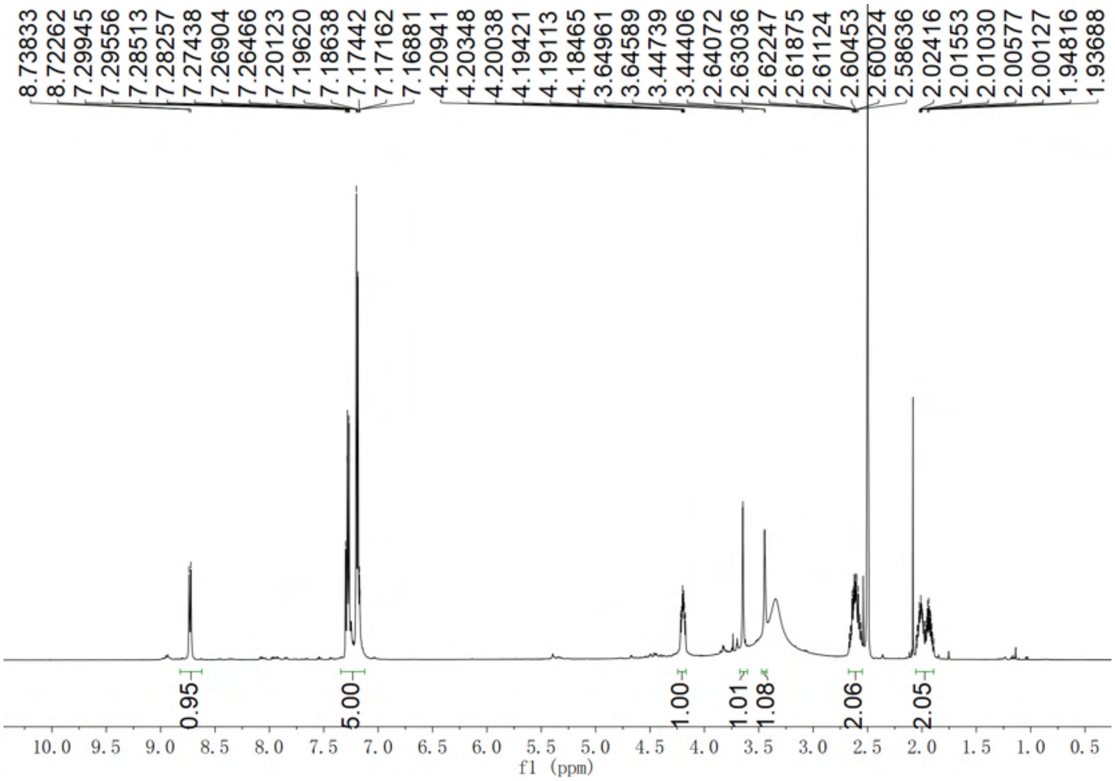

Supplementary Fig. 187. ^{1}H NMR spectrum of compound (2*S*,3*S*)-*t*-ES-a14 in DMSO-d_6

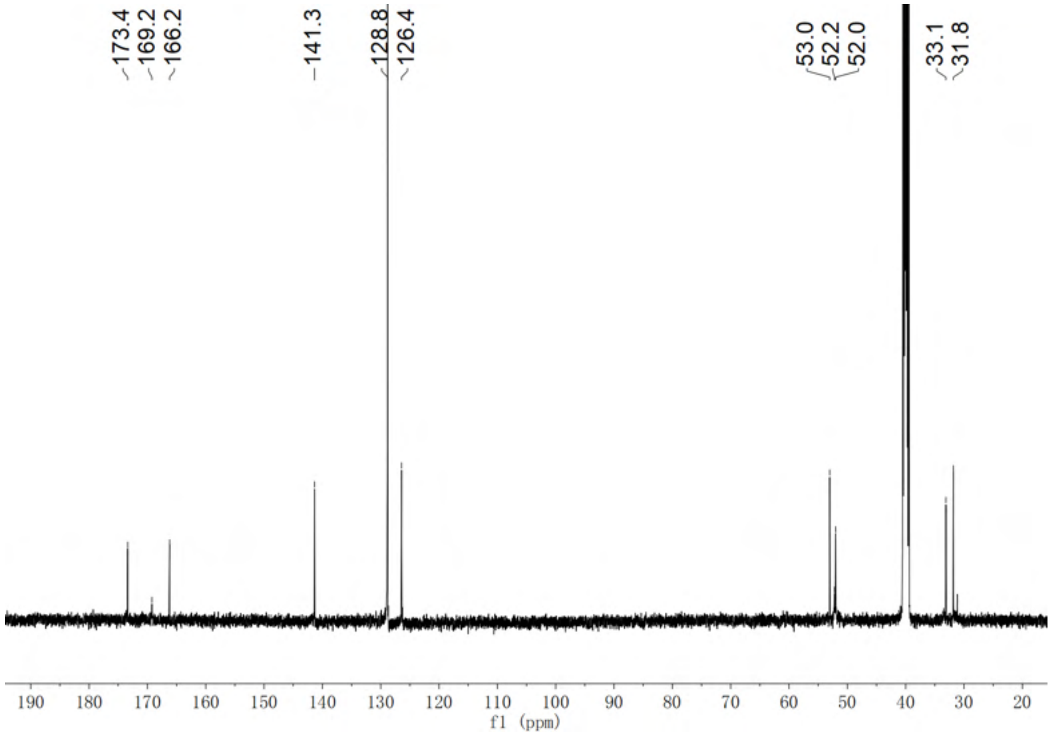

Supplementary Fig. 188. ^{13}C NMR spectrum of compound (2*S*,3*S*)-*t*-ES-a14 in DMSO-d_6

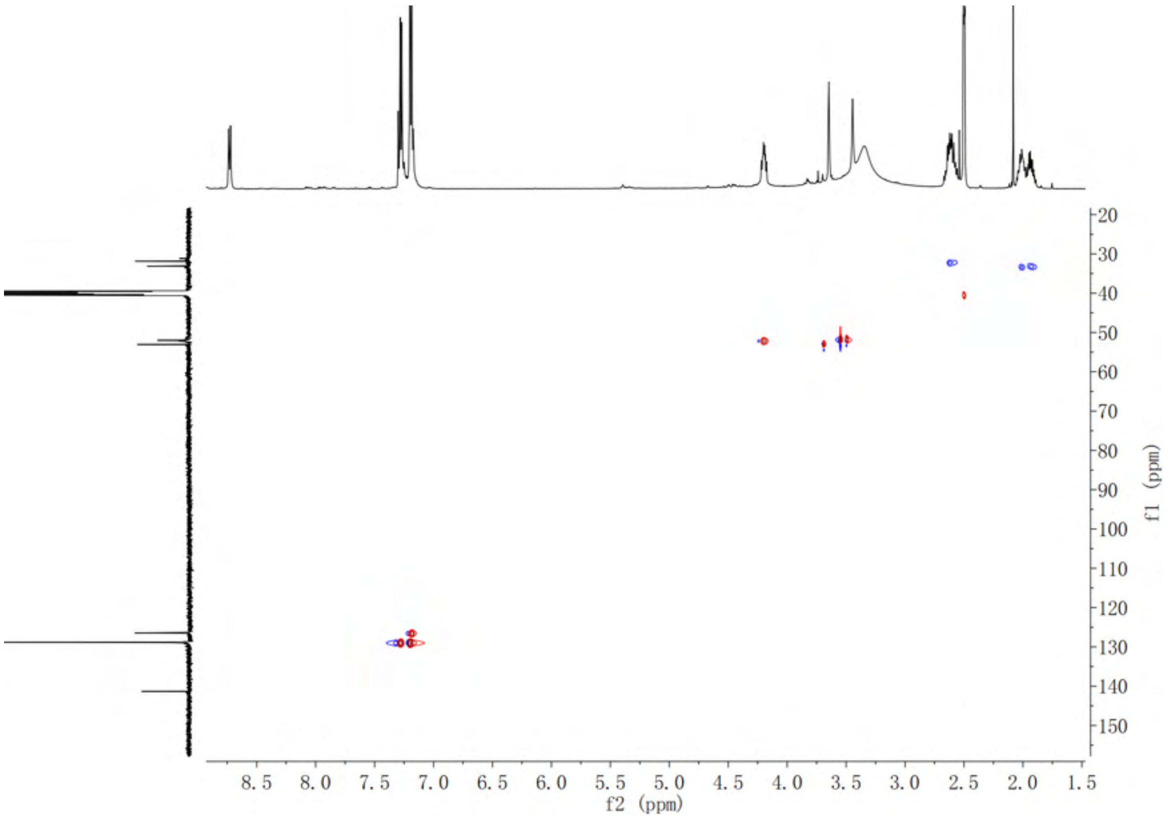

Supplementary Fig. 189. HSQC spectrum of compound (2*S*,3*S*)-*t*-ES-a14 in DMSO-d_6

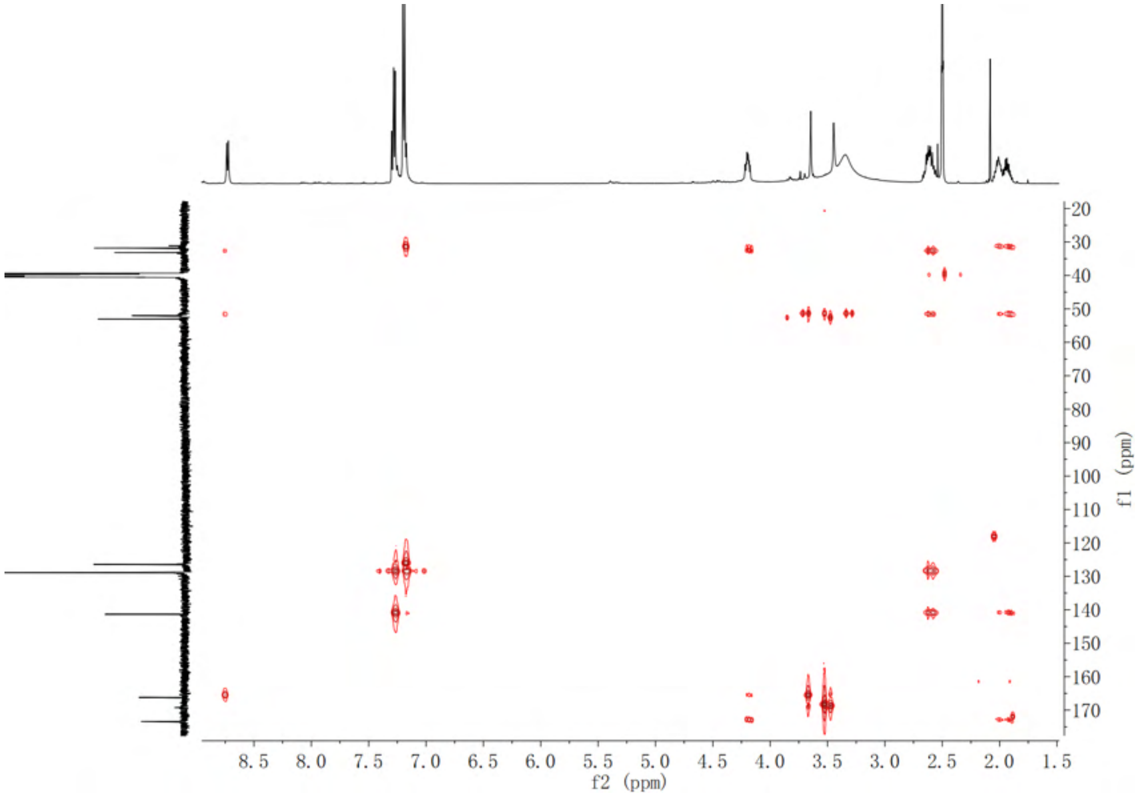

Supplementary Fig. 190. HMBC spectrum of compound (2*S*,3*S*)-*t*-ES-a14 in DMSO-d_6

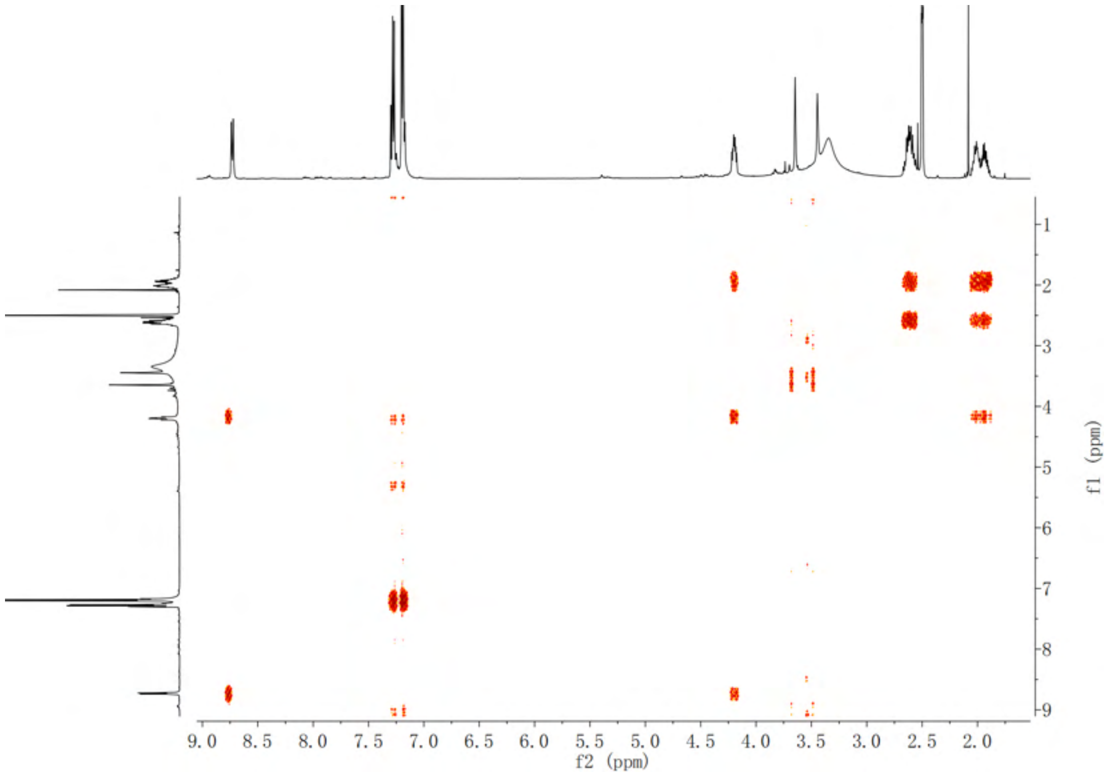

Supplementary Fig. 191. ^{1}**H-**^{1}**H COSY spectrum of compound (2*S*,3*S*)-*t*-ES-a14 in DMSO-*d*$_6$**

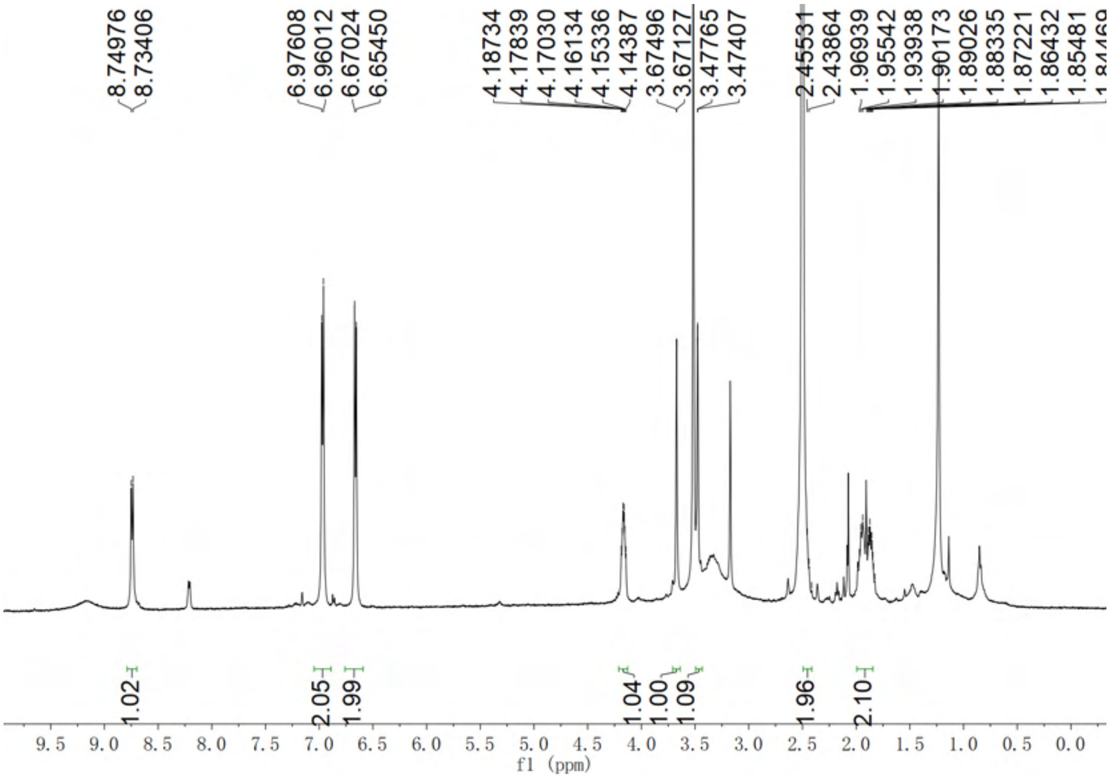

Supplementary Fig. 192. ^{1}**H NMR spectrum of compound (2*S*,3*S*)-*t*-ES-a15 in DMSO-*d*$_6$**

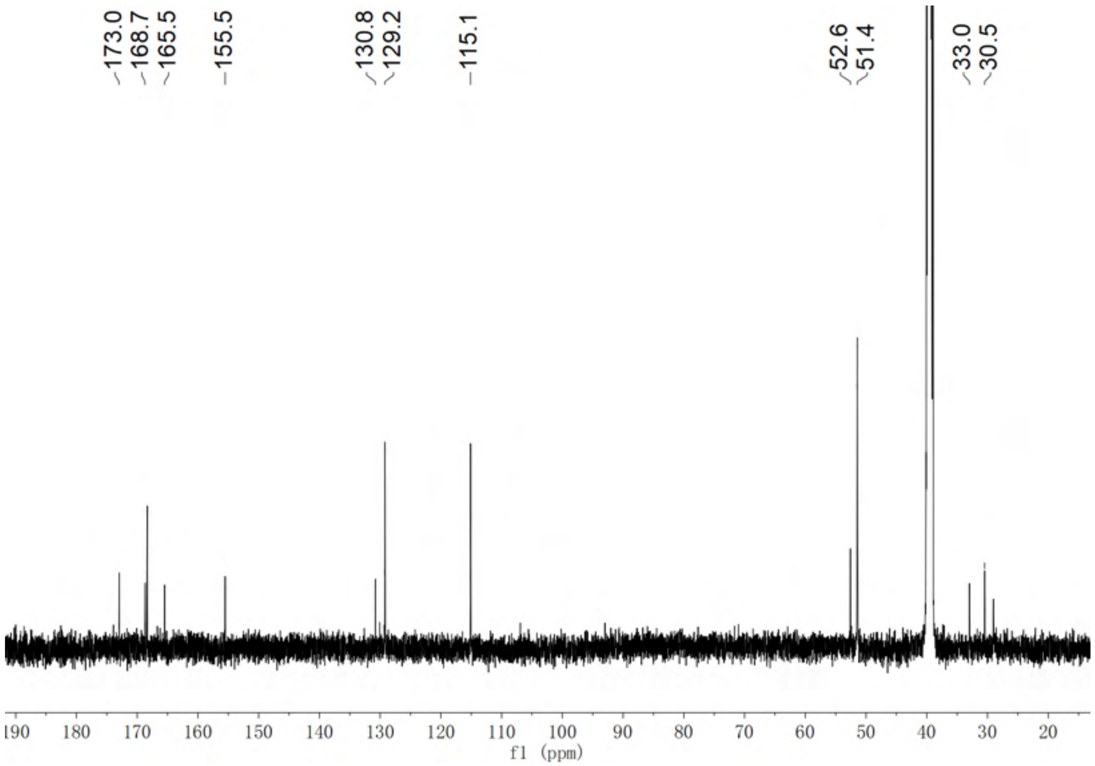

Supplementary Fig. 193. ^{13}C NMR spectrum of compound (2*S*,3*S*)-*t*-ES-a15 in DMSO-d_6

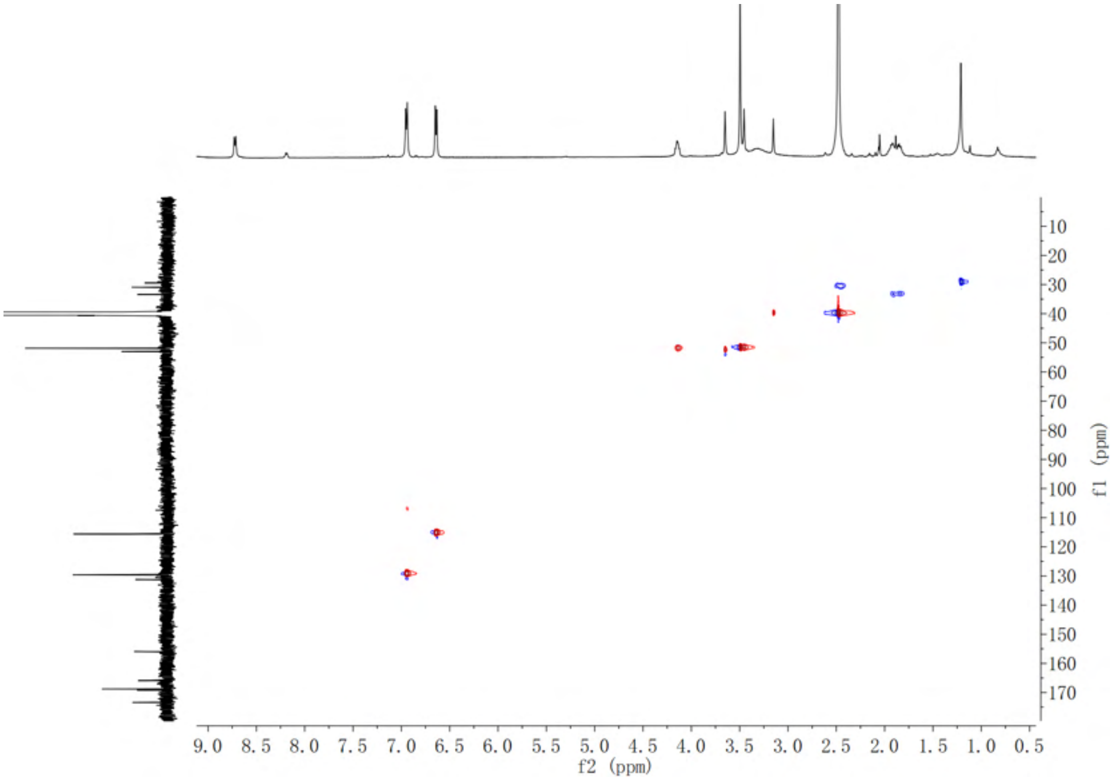

Supplementary Fig. 194. HSQC spectrum of compound (2*S*,3*S*)-*t*-ES-a15 in DMSO-d_6

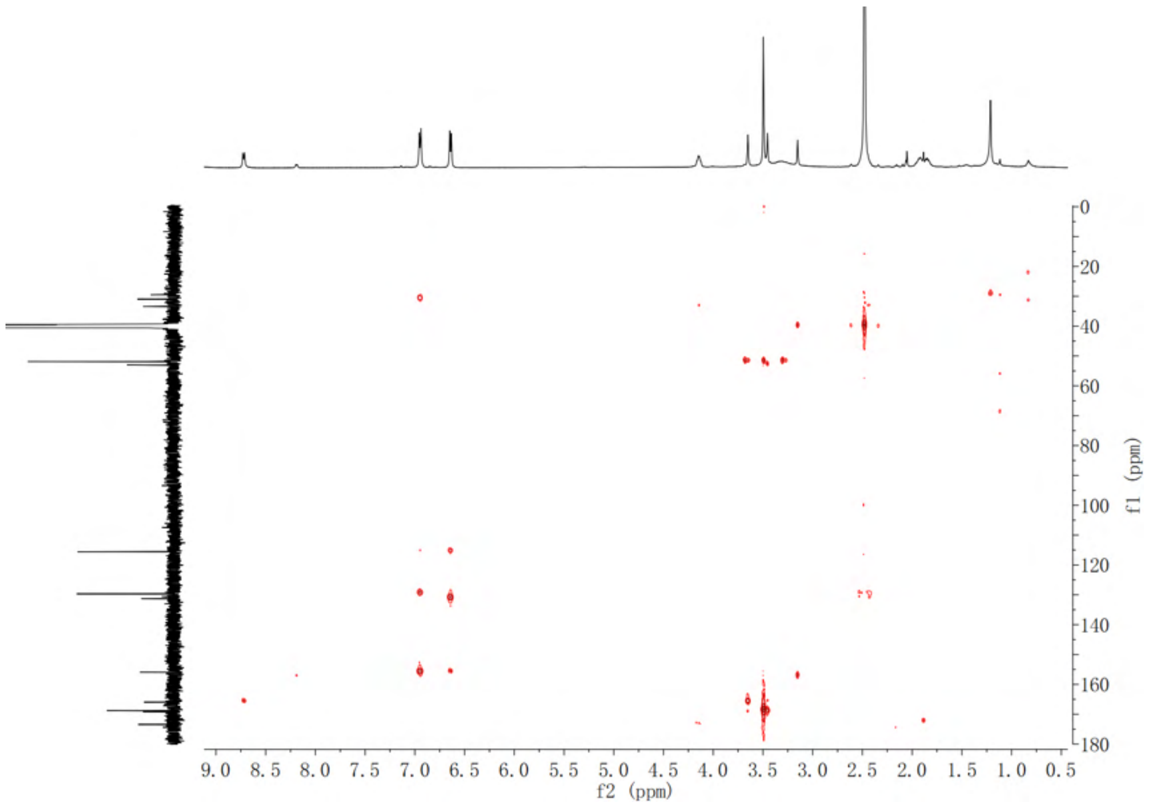

Supplementary Fig. 195. HMBC spectrum of compound (2*S*,3*S*)-*t*-ES-a15 in DMSO-d_6

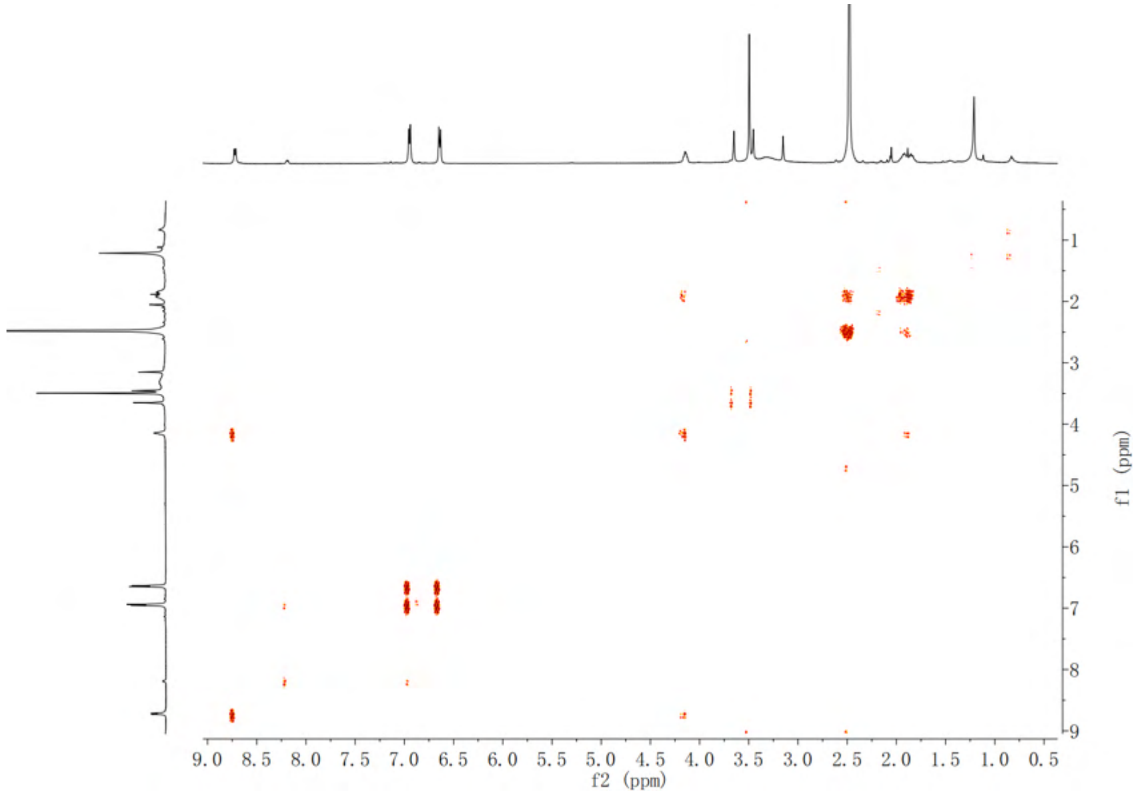

Supplementary Fig. 196. ^{1}H-^{1}H COSY spectrum of compound (2*S*,3*S*)-*t*-ES-a15 in DMSO-d_6

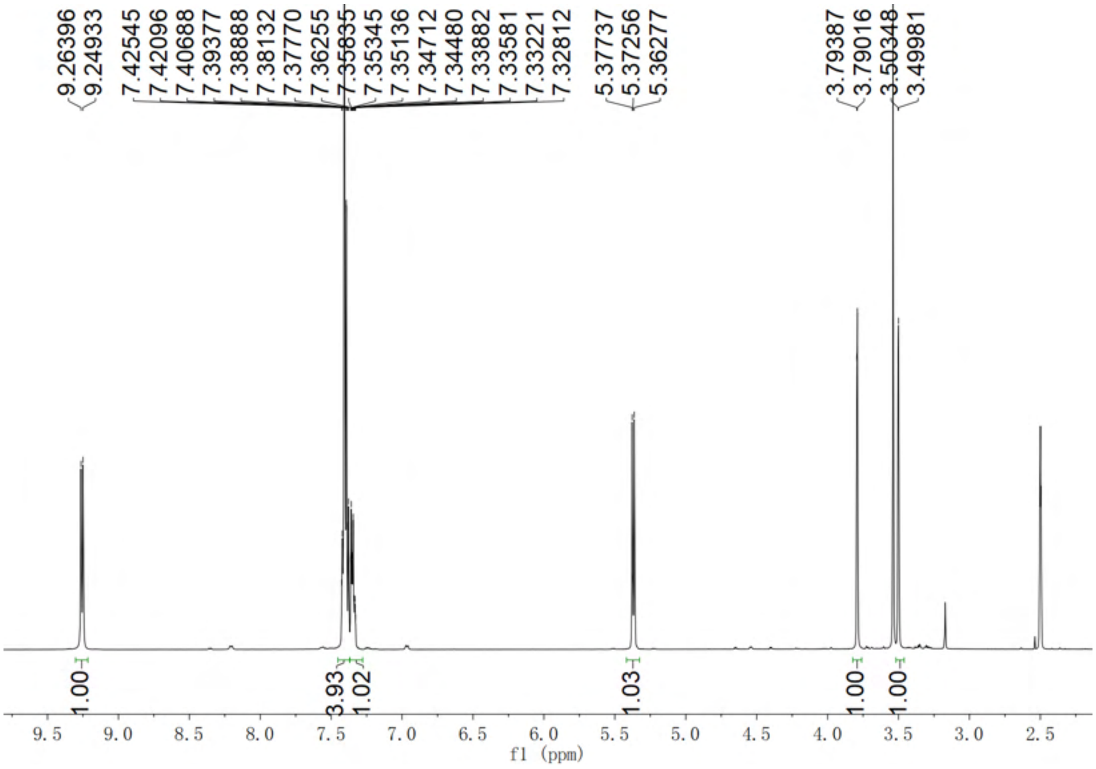

Supplementary Fig. 197. ¹H NMR spectrum of compound (2*S*,3*S*)-*t*-ES-a16 in DMSO-d_6

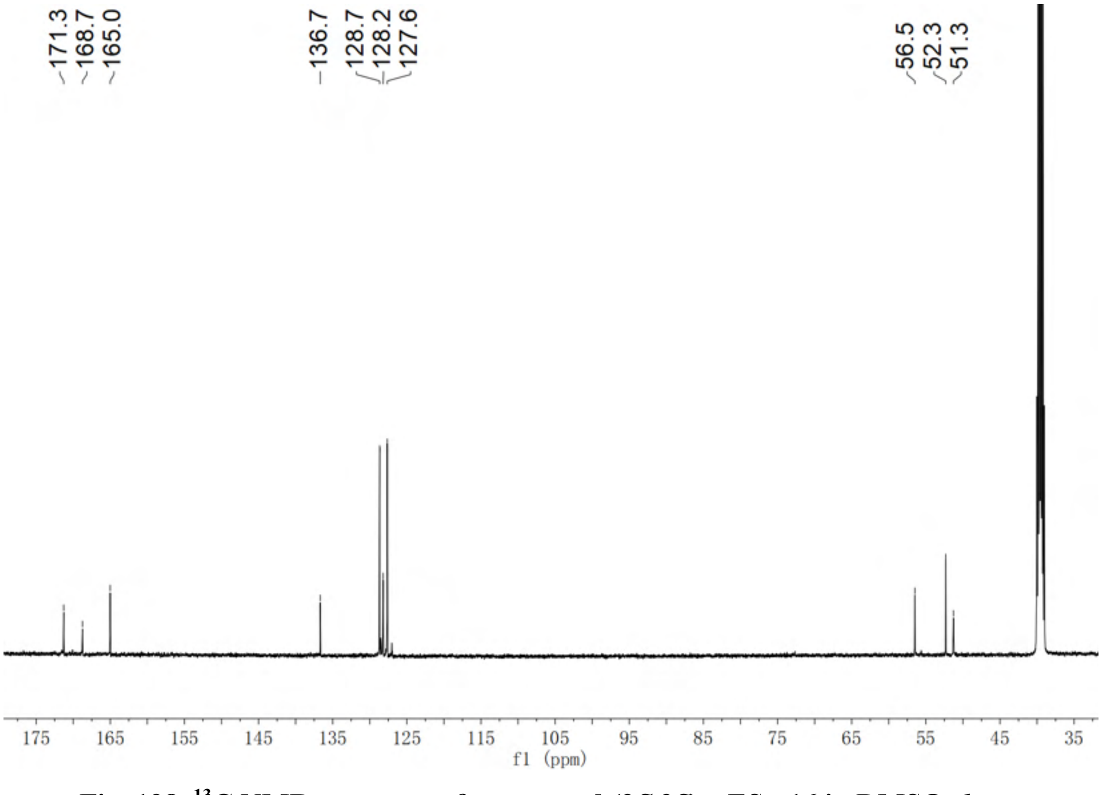

Supplementary Fig. 198. ¹³C NMR spectrum of compound (2*S*,3*S*)-*t*-ES-a16 in DMSO-d_6

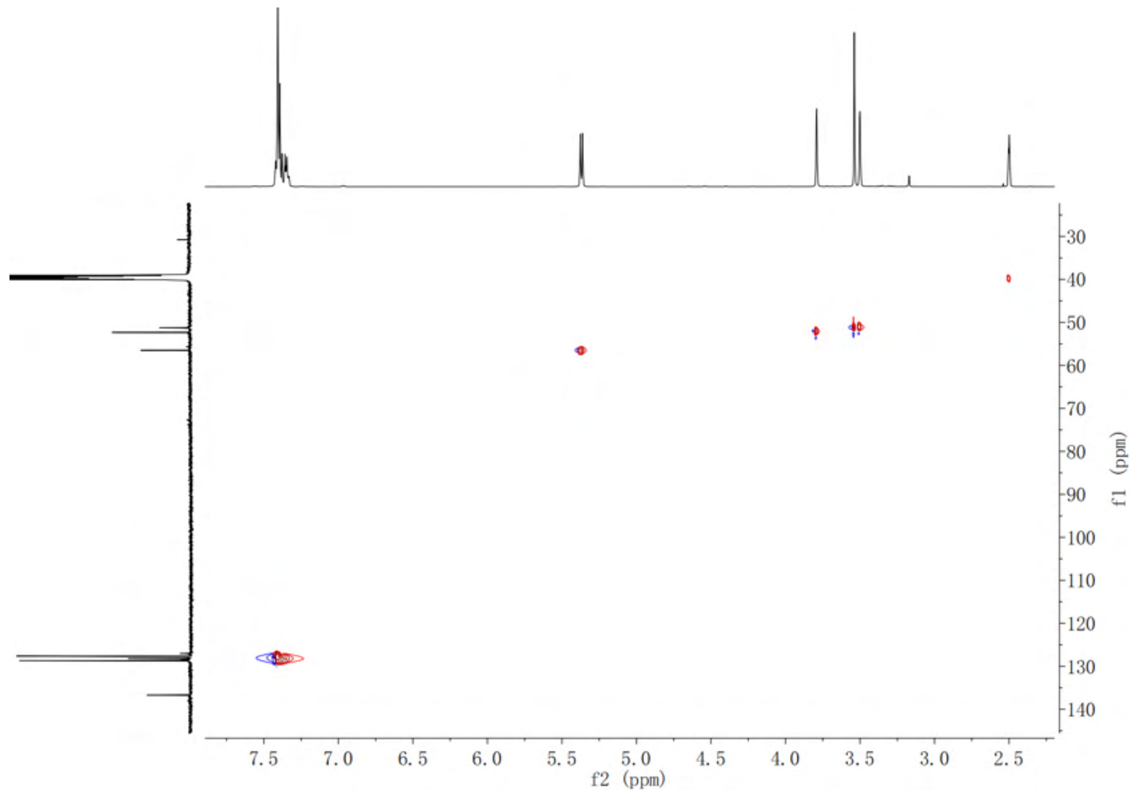

Supplementary Fig. 199. HSQC spectrum of compound (2*S*,3*S*)-*t*-ES-a16 in DMSO-d_6

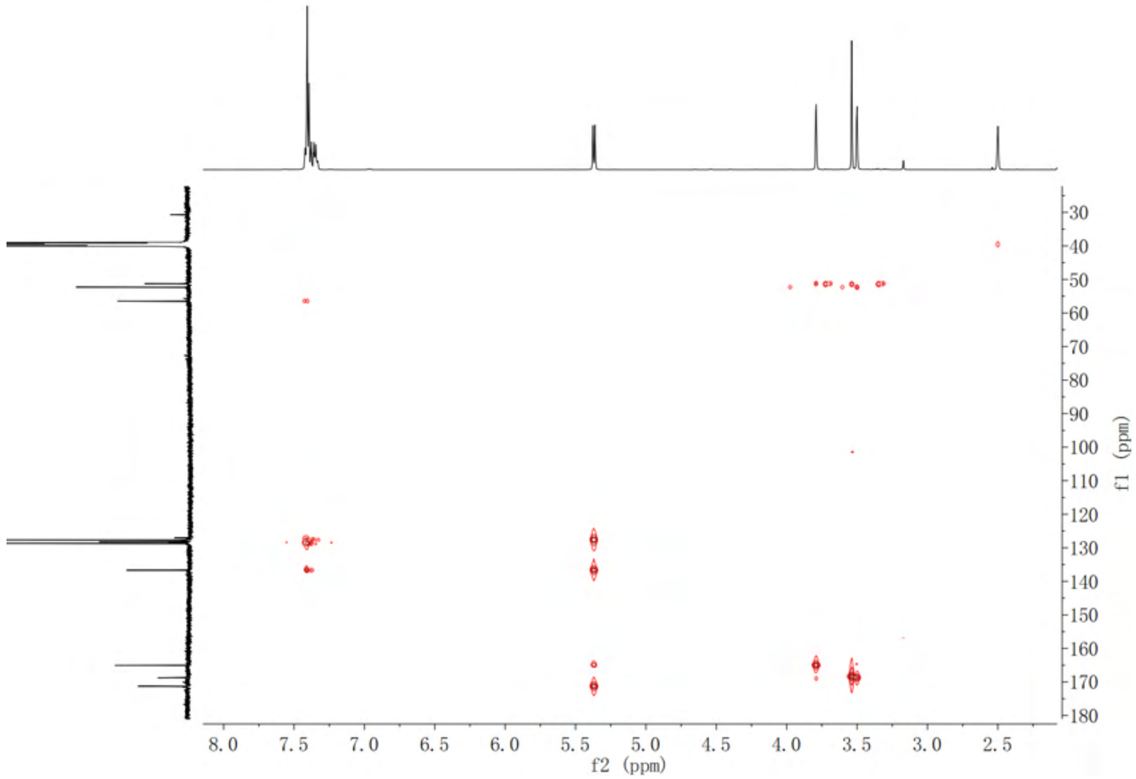

Supplementary Fig. 200. HMBC spectrum of compound (2*S*,3*S*)-*t*-ES-a16 in DMSO-d_6

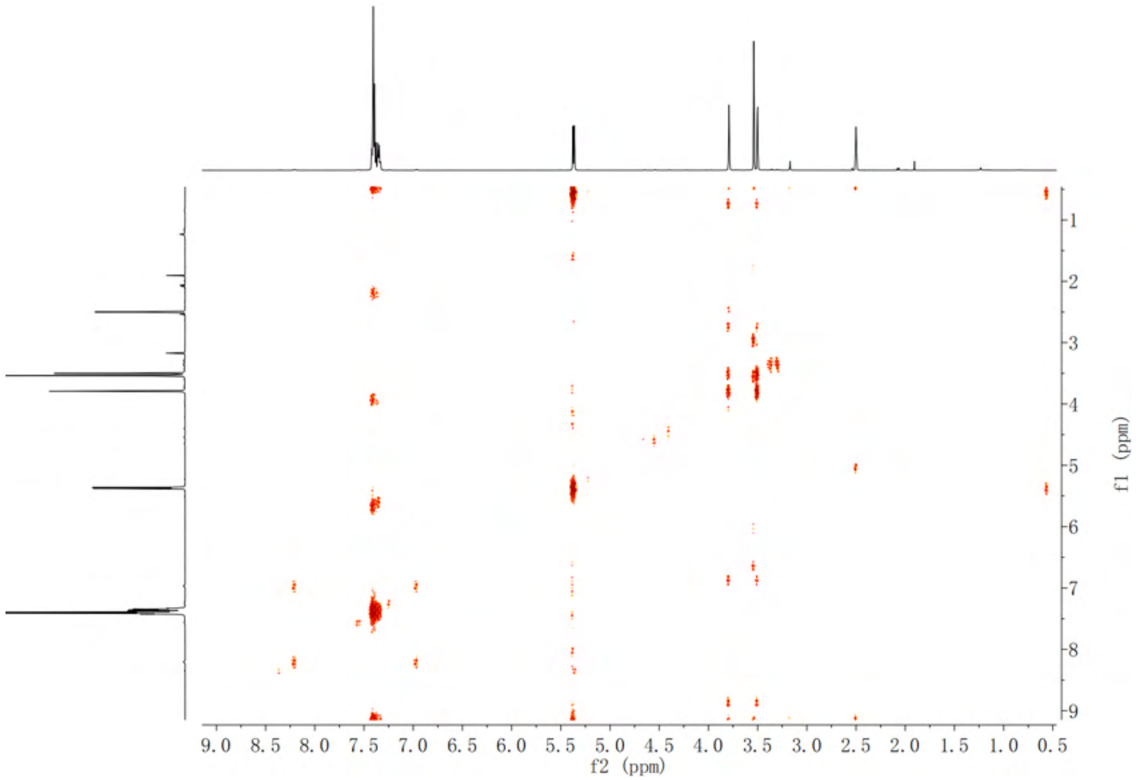

Supplementary Fig. 201. ^{1}**H-**^{1}**H COSY spectrum of compound (2*S*,3*S*)-*t*-ES-a16 in DMSO-*d*$_6$**

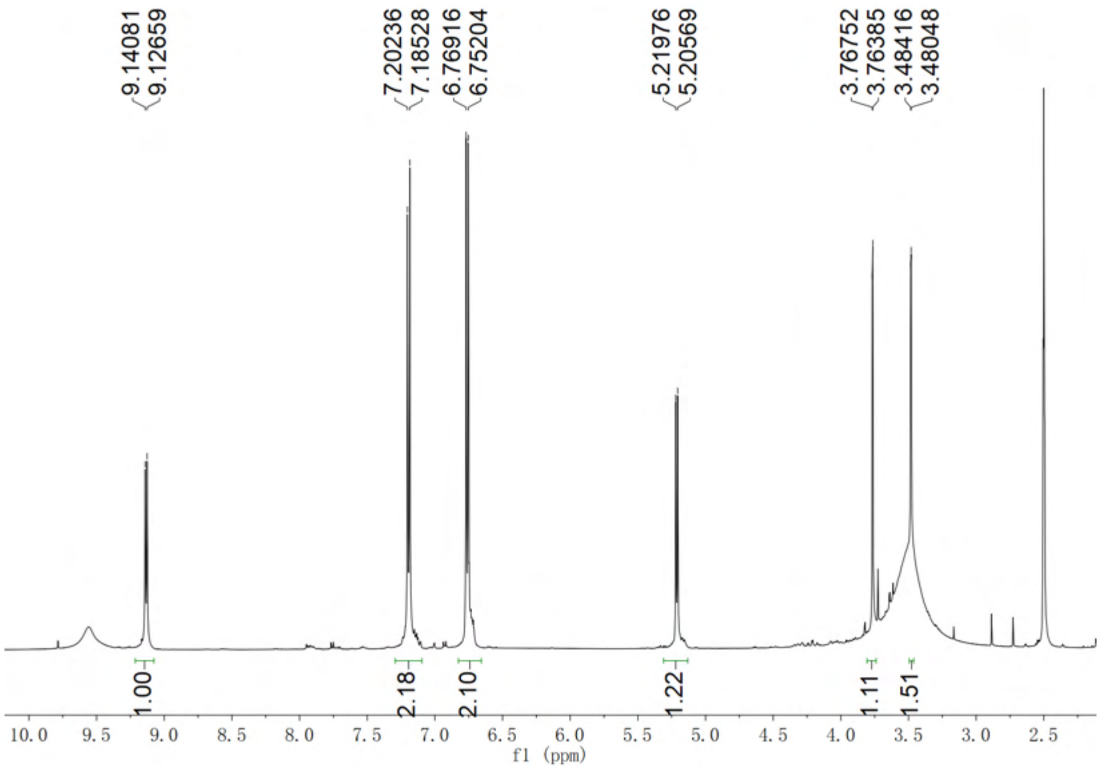

Supplementary Fig. 202. ^{1}**H NMR spectrum of compound (2*S*,3*S*)-*t*-ES-a17 in DMSO-*d*$_6$**

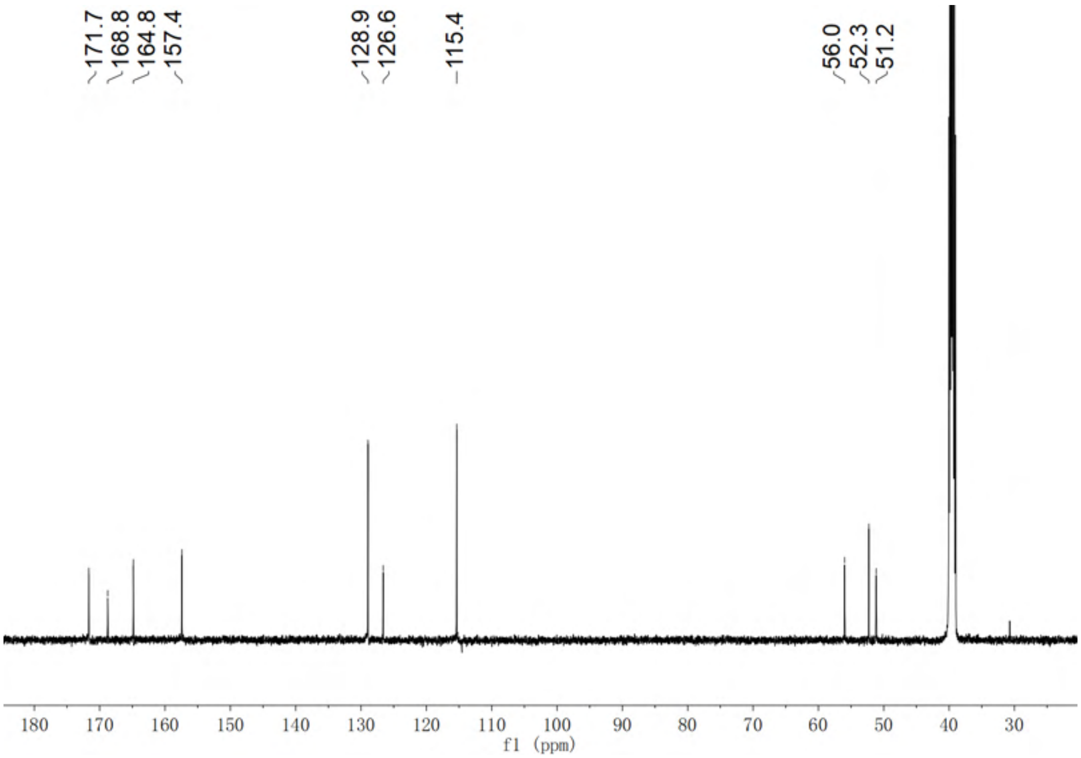

Supplementary Fig. 203. ^{13}**C NMR spectrum of compound (2*S*,3*S*)-*t*-ES-a17 in DMSO-*d*$_6$**

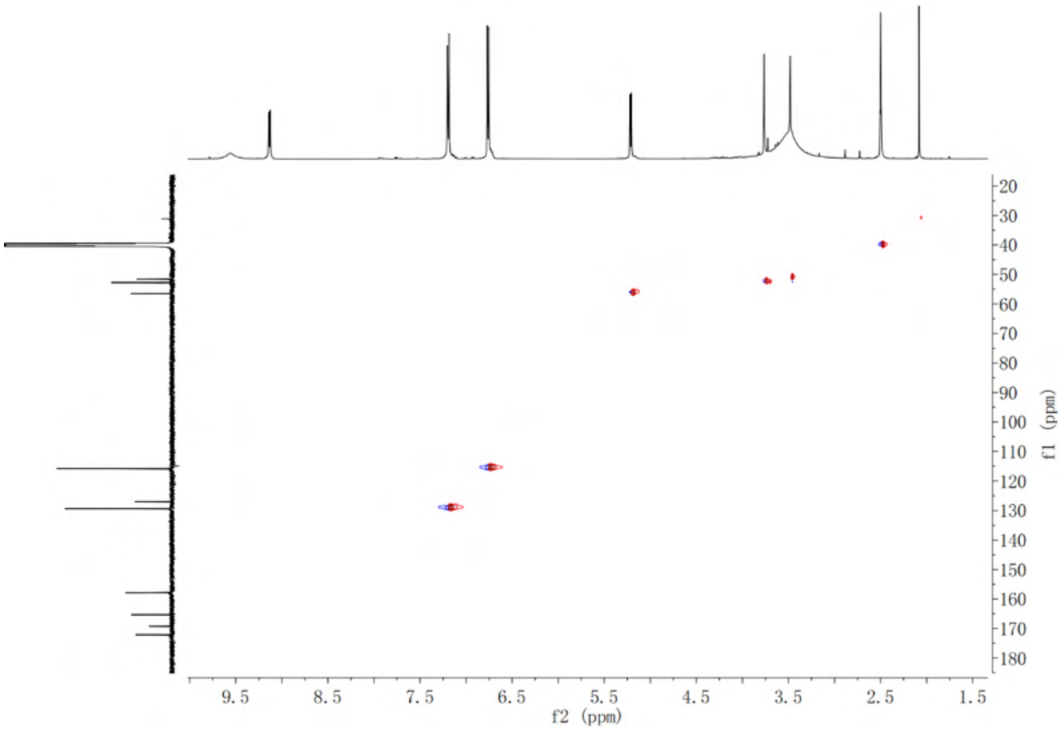

Supplementary Fig. 204. HSQC spectrum of compound (2*S*,3*S*)-*t*-ES-a17 in DMSO-*d*$_6$

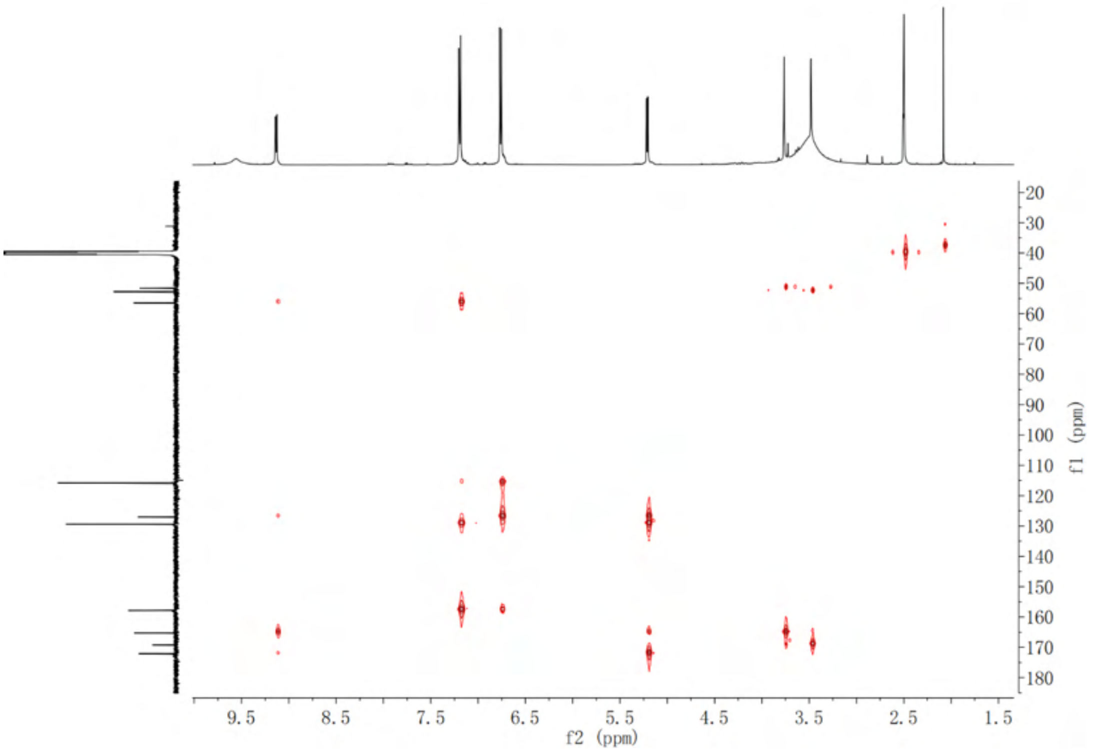

Supplementary Fig. 205. HMBC spectrum of compound (2*S*,3*S*)-*t*-ES-a17 in DMSO-d_6

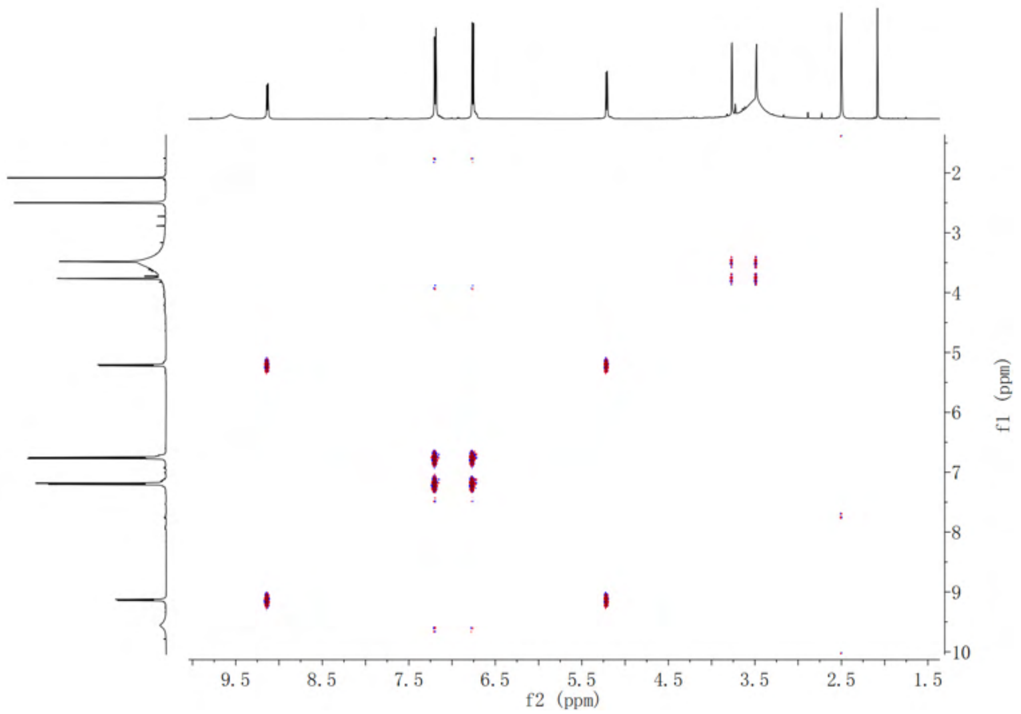

Supplementary Fig. 206. ^{1}H-^{1}H COSY spectrum of compound (2*S*,3*S*)-*t*-ES-a17 in DMSO-d_6

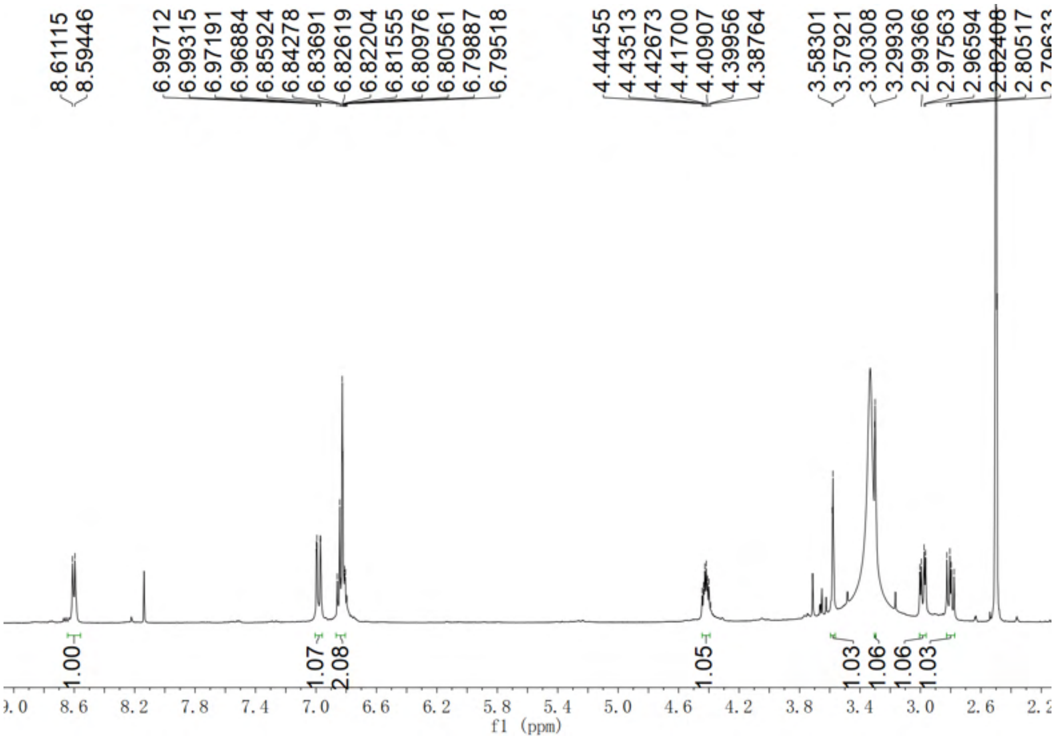

Supplementary Fig. 207. ^{1}H NMR spectrum of compound (2*S*,3*S*)-*t*-ES-a18 in DMSO-d_6

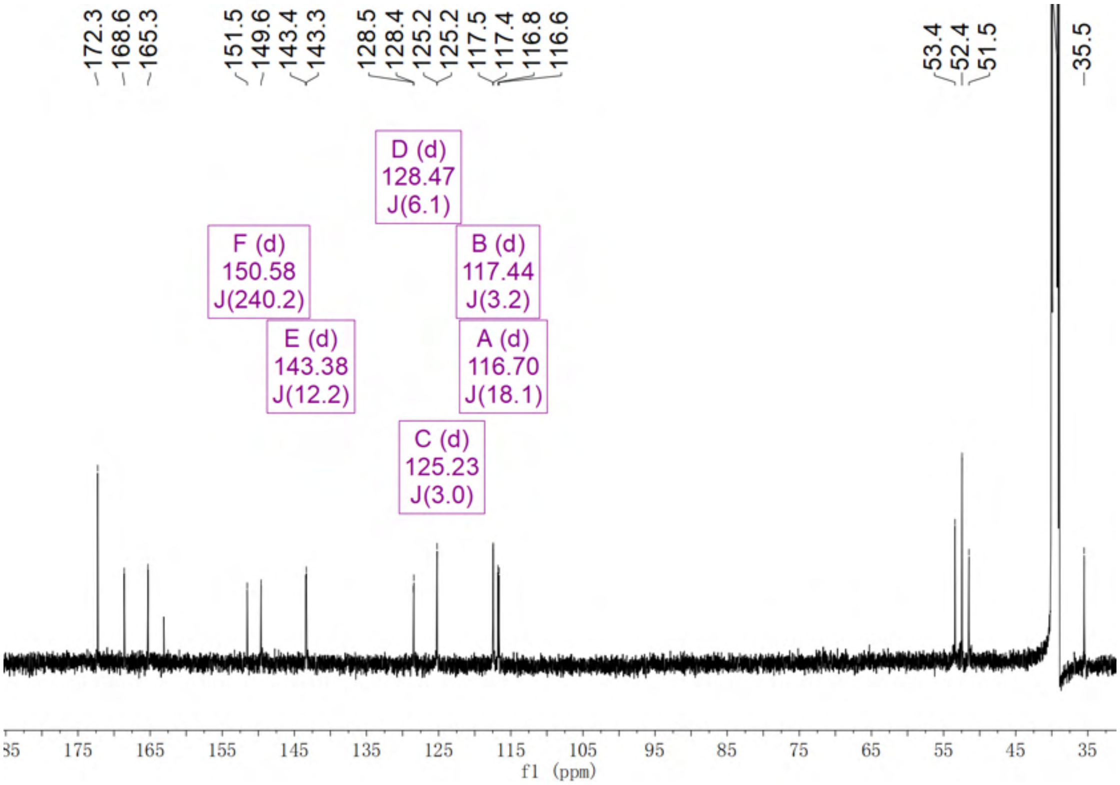

Supplementary Fig. 208. ^{13}C NMR spectrum of compound (2*S*,3*S*)-*t*-ES-a18 in DMSO-d_6

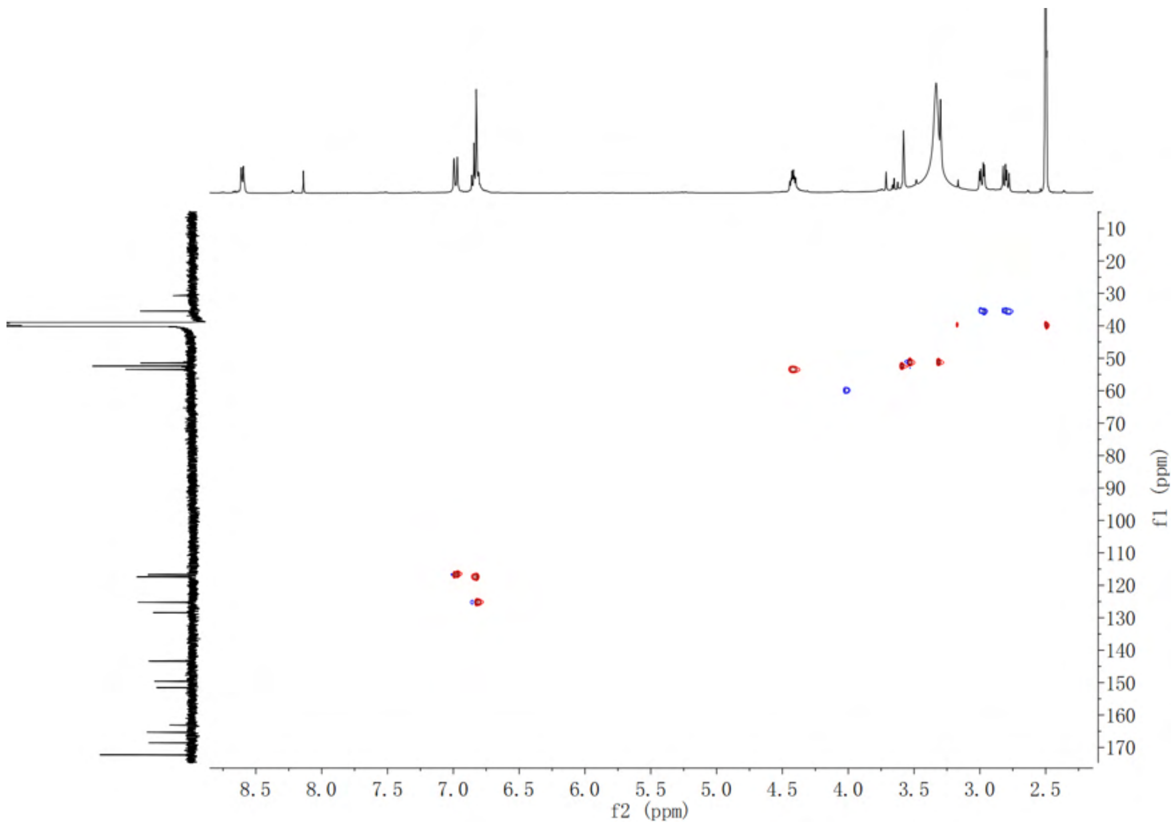

Supplementary Fig. 209. HSQC spectrum of compound (2*S*,3*S*)-*t*-ES-a18 in DMSO-d_6

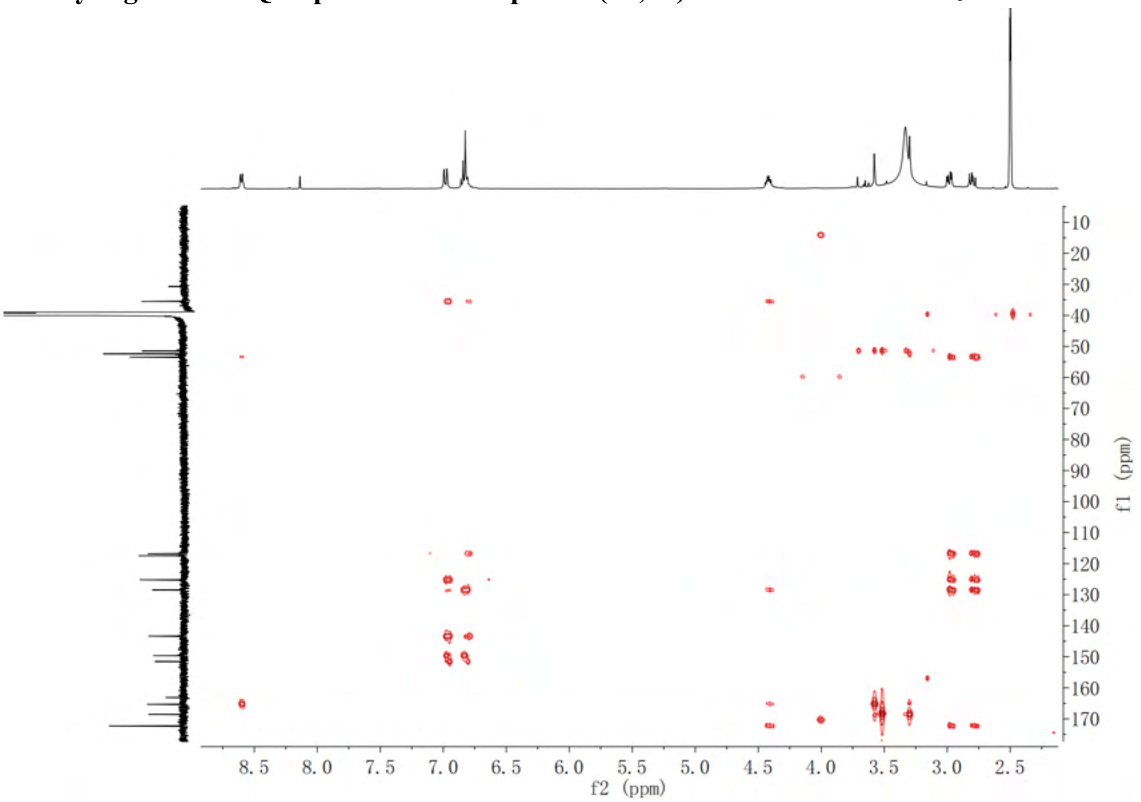

Supplementary Fig. 210. HMBC spectrum of compound (2*S*,3*S*)-*t*-ES-a18 in DMSO-d_6

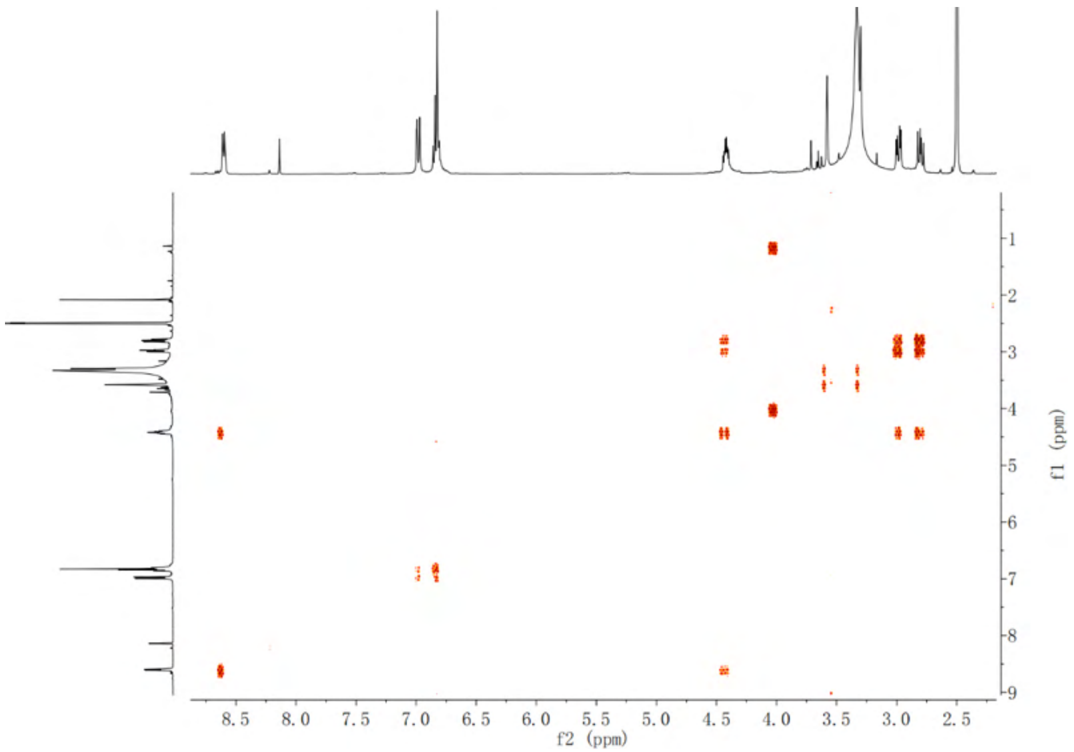

Supplementary Fig. 211. ^{1}**H-**^{1}**H COSY spectrum of compound (2*S*,3*S*)-*t*-ES-a18 in DMSO-*d*$_6$**

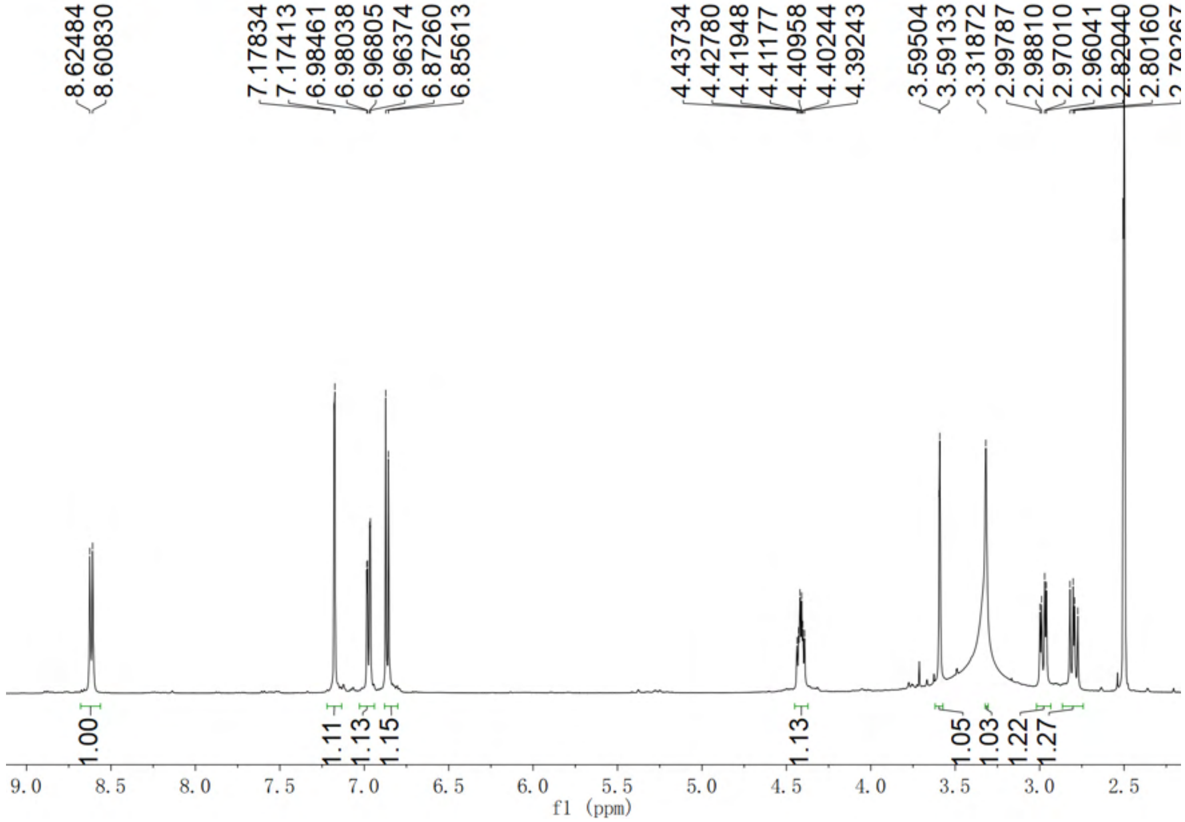

Supplementary Fig. 212. ^{1}**H NMR spectrum of compound (2*S*,3*S*)-*t*-ES-a19 in DMSO-*d*$_6$**

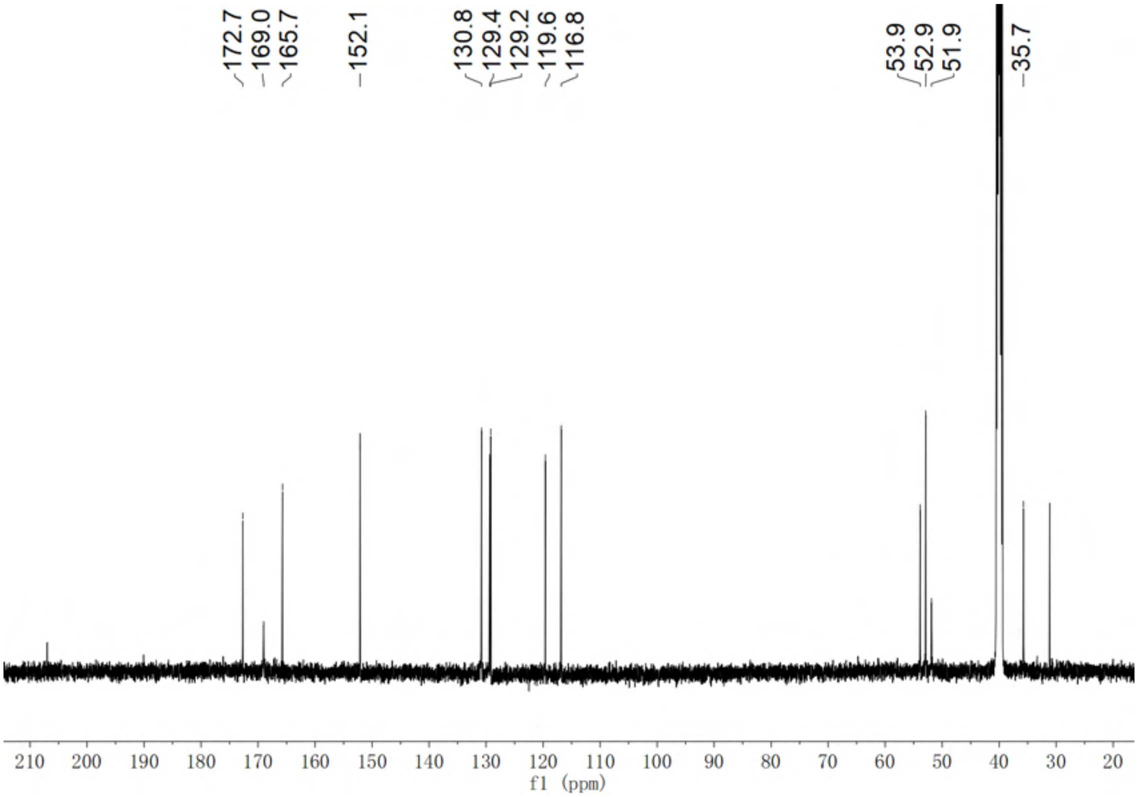

Supplementary Fig. 213. ^{13}C NMR spectrum of compound (2*S*,3*S*)-*t*-ES-a19 in DMSO-d_6

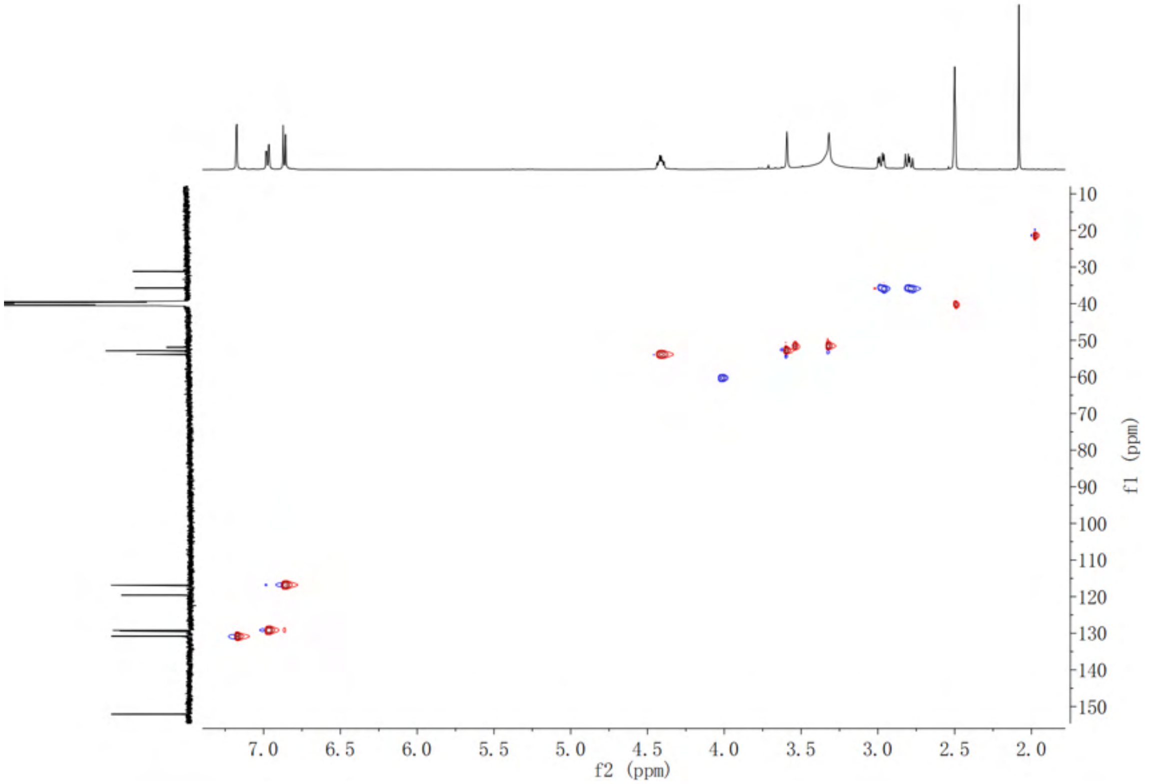

Supplementary Fig. 214. HSQC spectrum of compound (2*S*,3*S*)-*t*-ES-a19 in DMSO-d_6

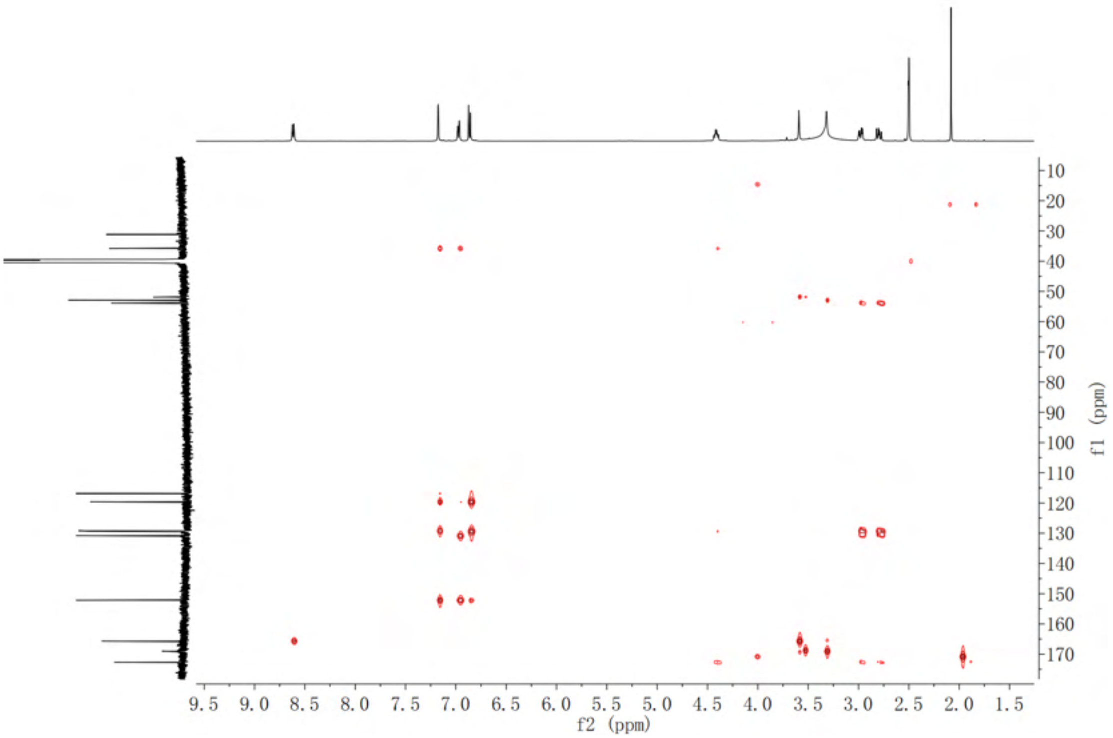

Supplementary Fig. 215. HMBC spectrum of compound (2*S*,3*S*)-*t*-ES-a19 in DMSO-d_6

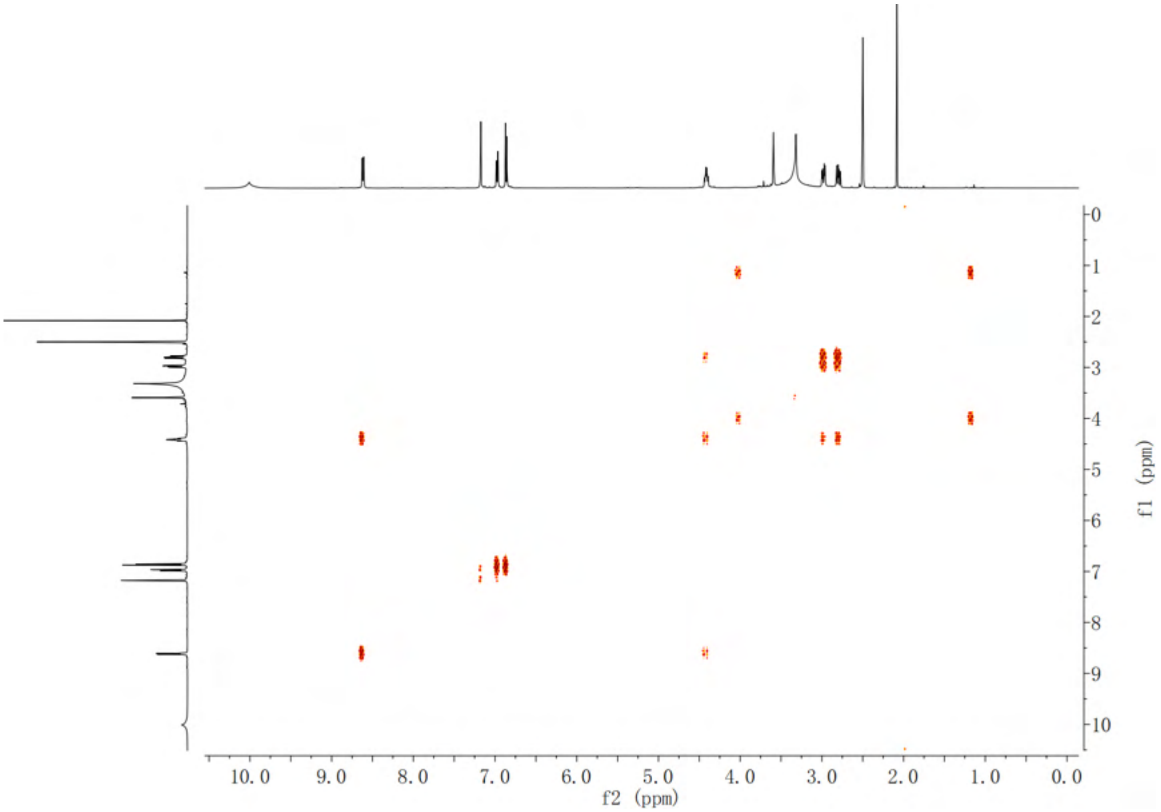

Supplementary Fig. 216. ^{1}H-^{1}H COSY spectrum of compound (2*S*,3*S*)-*t*-ES-a19 in DMSO-d_6

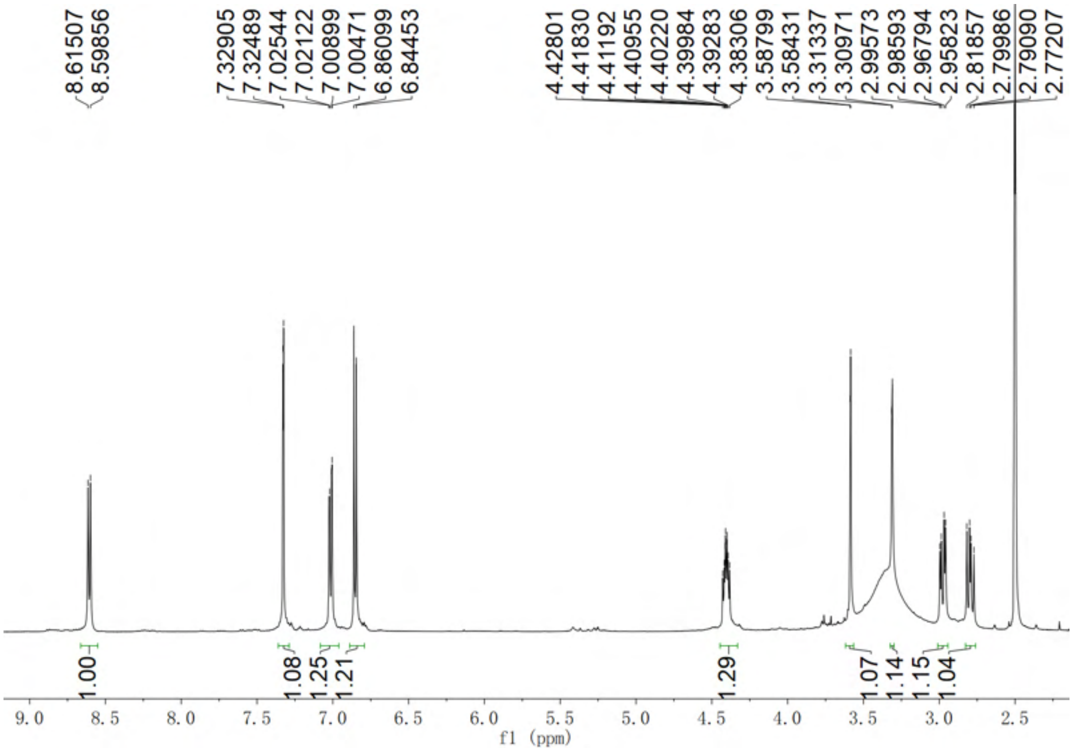

Supplementary Fig. 217. ^{1}**H NMR spectrum of compound (2*S*,3*S*)-*t*-ES-a20 in DMSO-*d*$_6$**

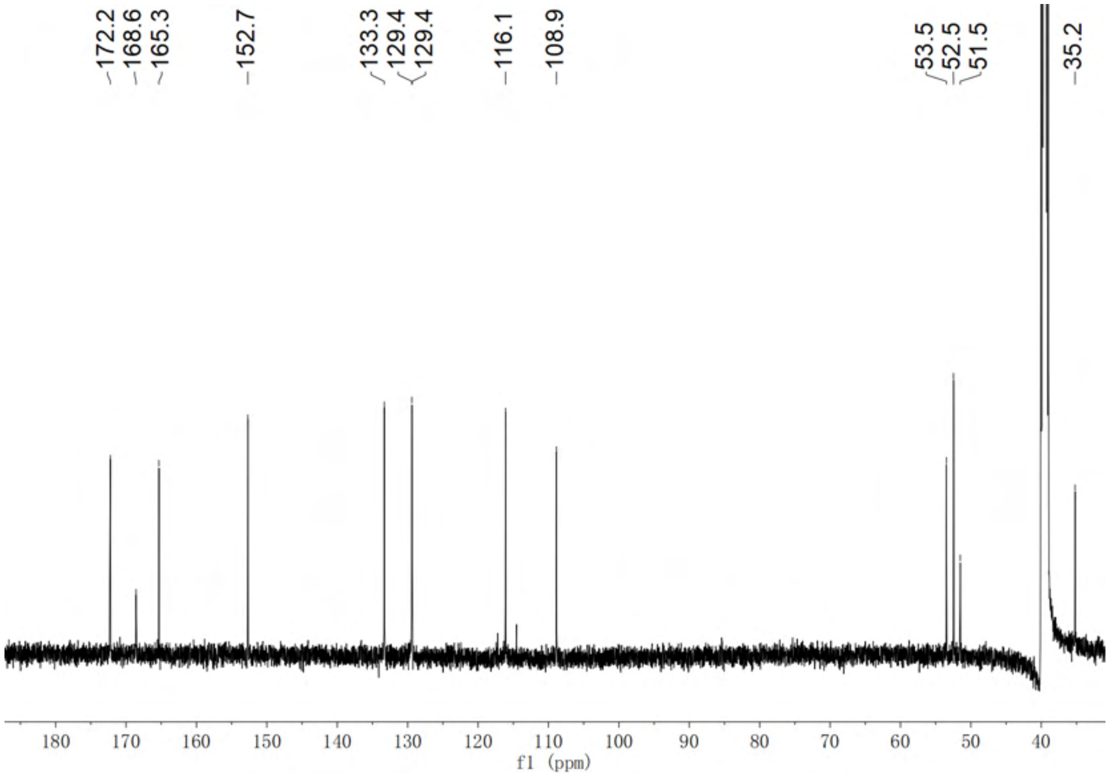

Supplementary Fig. 218. ^{13}**C NMR spectrum of compound (2*S*,3*S*)-*t*-ES-a20 in DMSO-*d*$_6$**

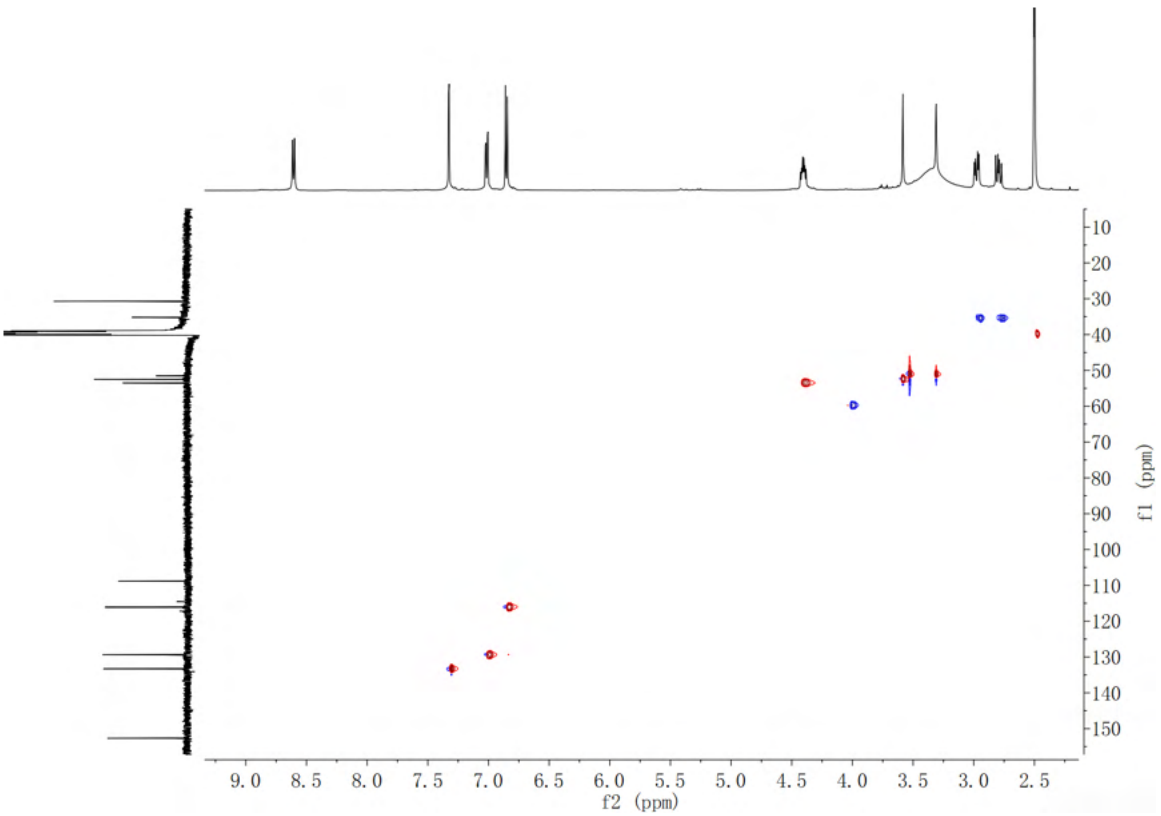

Supplementary Fig. 219. HSQC spectrum of compound (2*S*,3*S*)-*t*-ES-a20 in DMSO-*d*$_6$

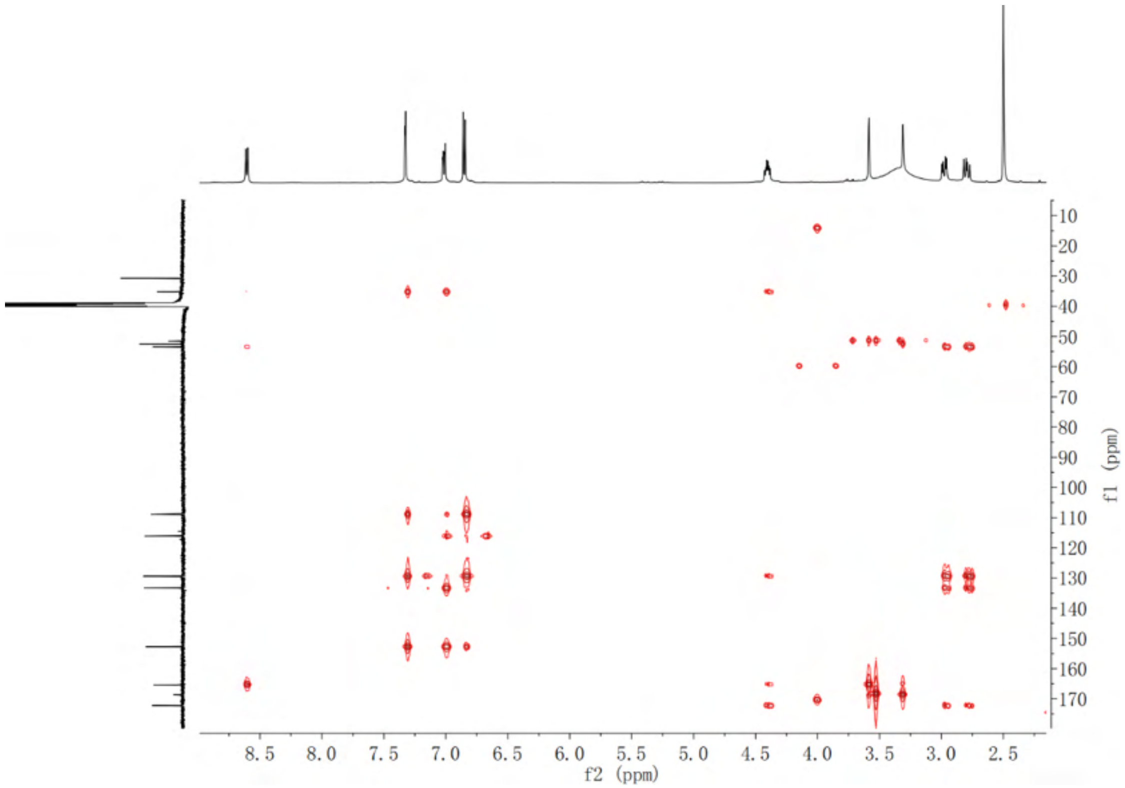

Supplementary Fig. 220. HMBC spectrum of compound (2*S*,3*S*)-*t*-ES-a20 in DMSO-*d*$_6$

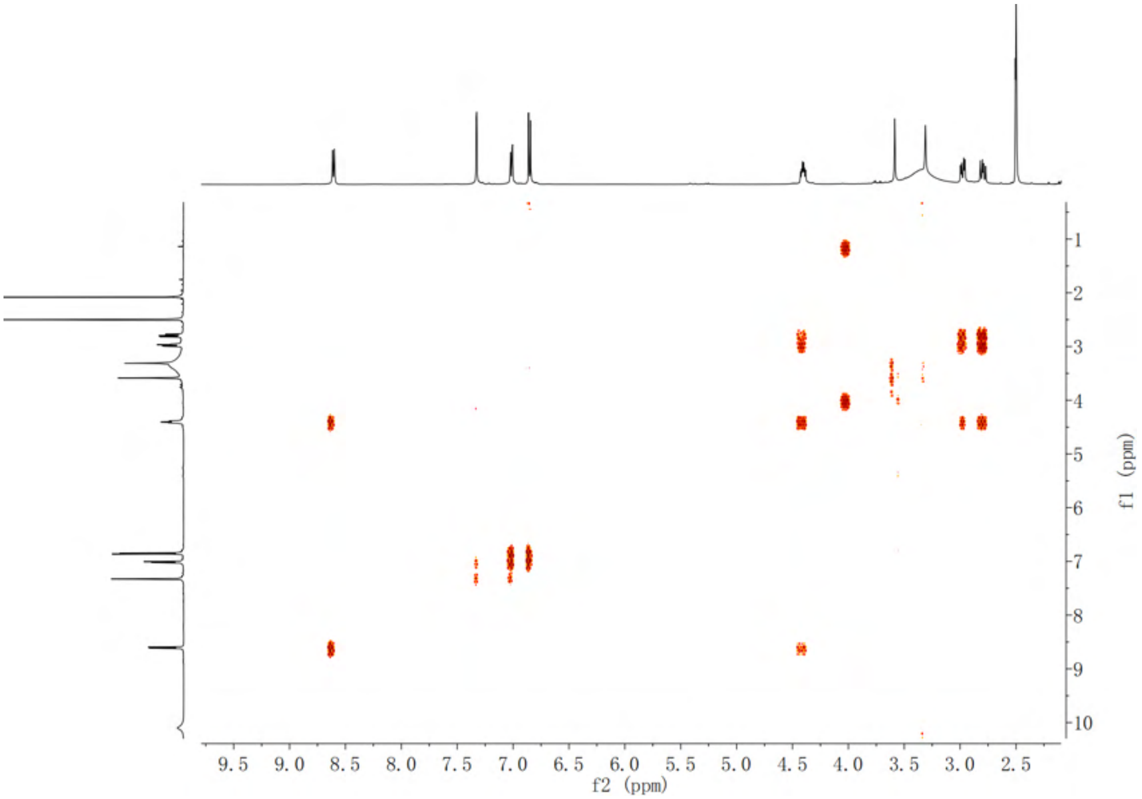

Supplementary Fig. 221. ^{1}**H-**^{1}**H COSY spectrum of compound (2*S*,3*S*)-*t*-ES-a20 in DMSO-*d*$_6$**

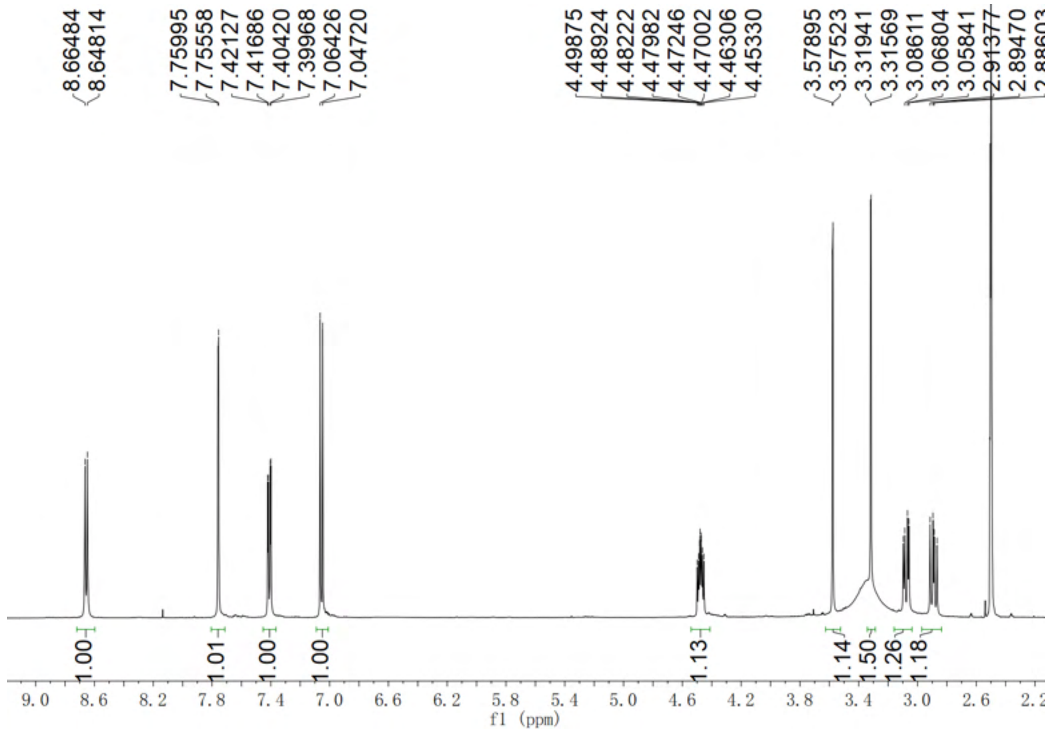

Supplementary Fig. 222. ^{1}**H NMR spectrum of compound (2*S*,3*S*)-*t*-ES-a21 in DMSO-*d*$_6$**

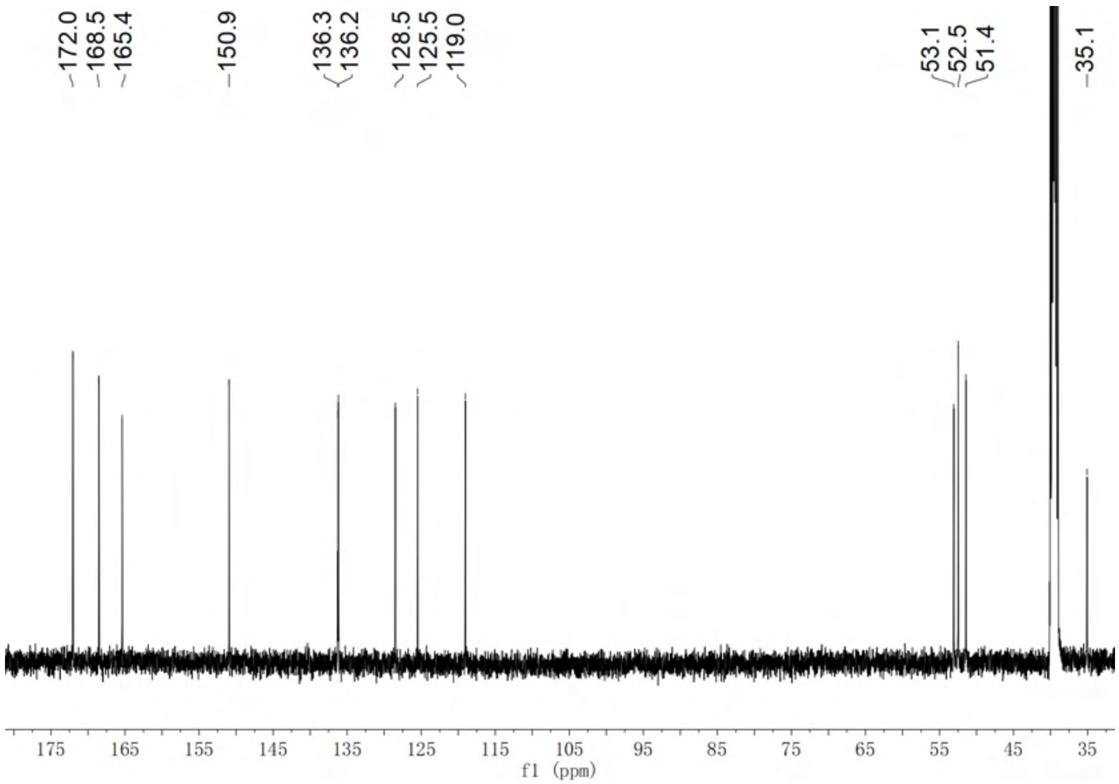

Supplementary Fig. 223. ^{13}C NMR spectrum of compound (2*S*,3*S*)-*t*-ES-a21 in DMSO-d_6

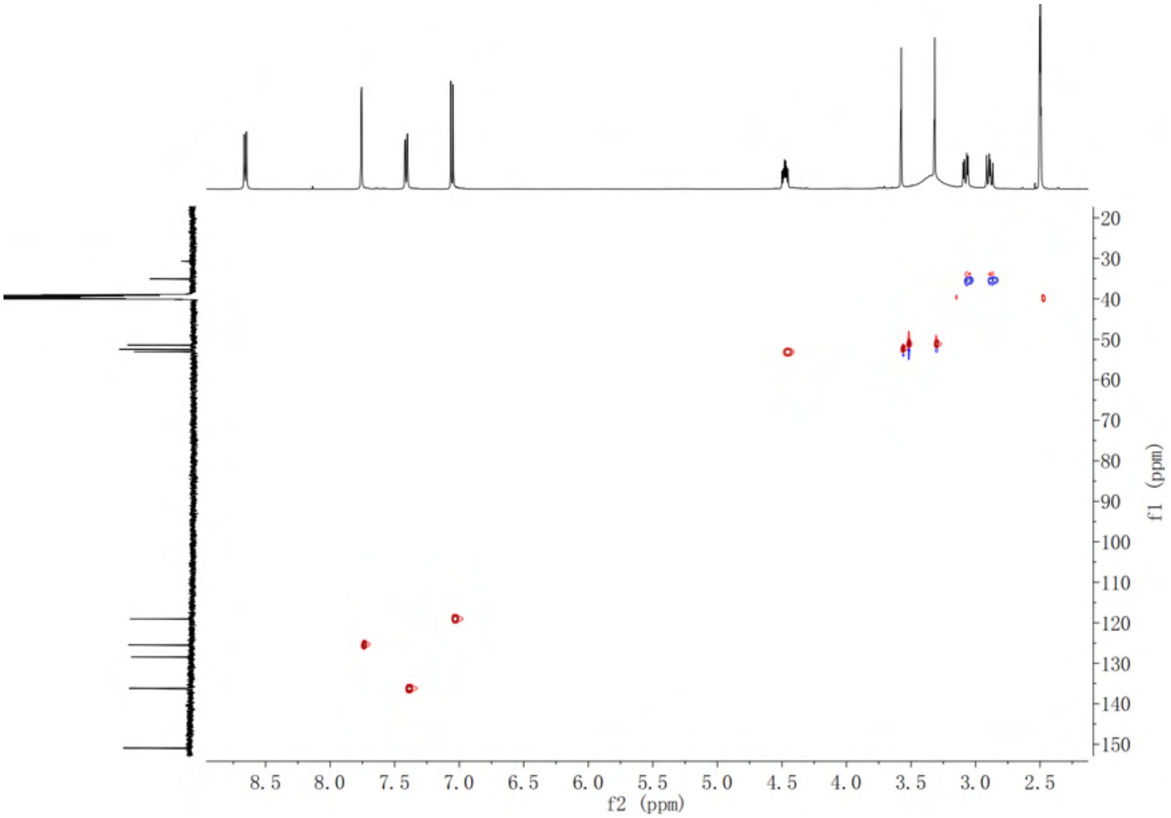

Supplementary Fig. 224. HSQC spectrum of compound (2*S*,3*S*)-*t*-ES-a21 in DMSO-d_6

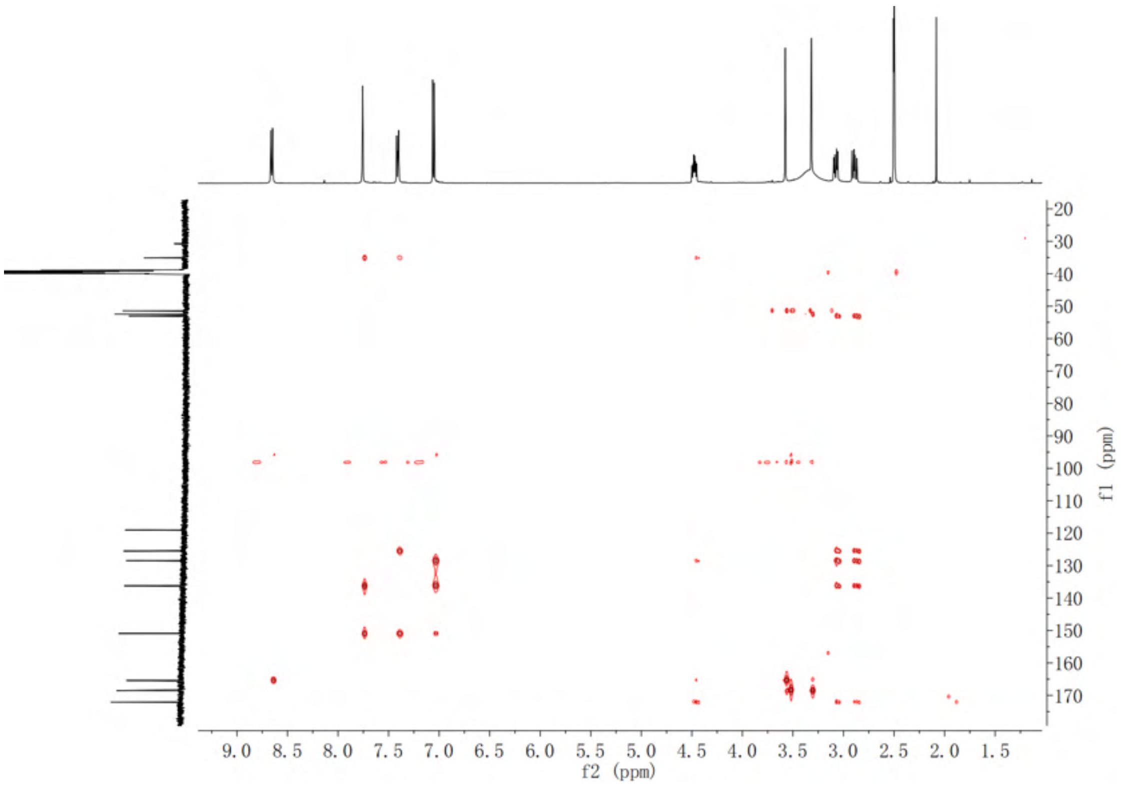

Supplementary Fig. 225. HMBC spectrum of compound (2*S*,3*S*)-*t*-ES-a21 in DMSO-d_6

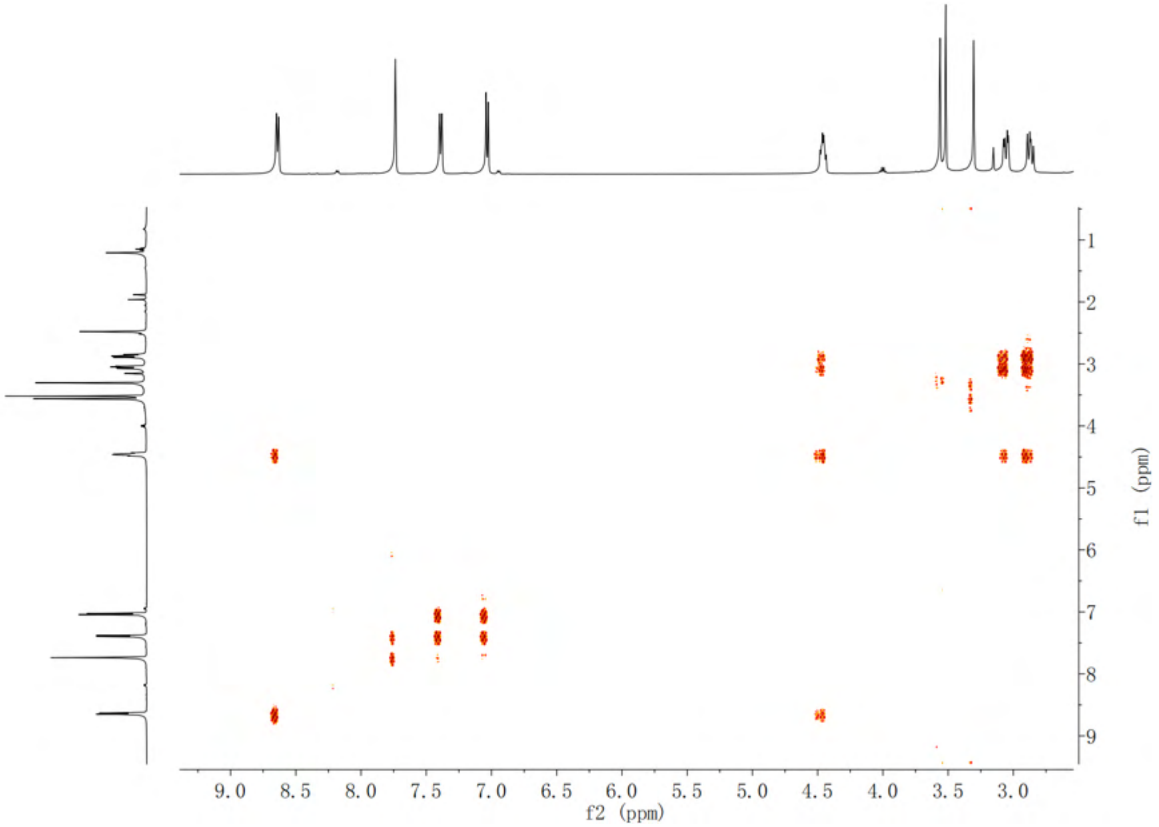

Supplementary Fig. 226. ^{1}H-^{1}H COSY spectrum of compound (2*S*,3*S*)-*t*-ES-a21 in DMSO-d_6

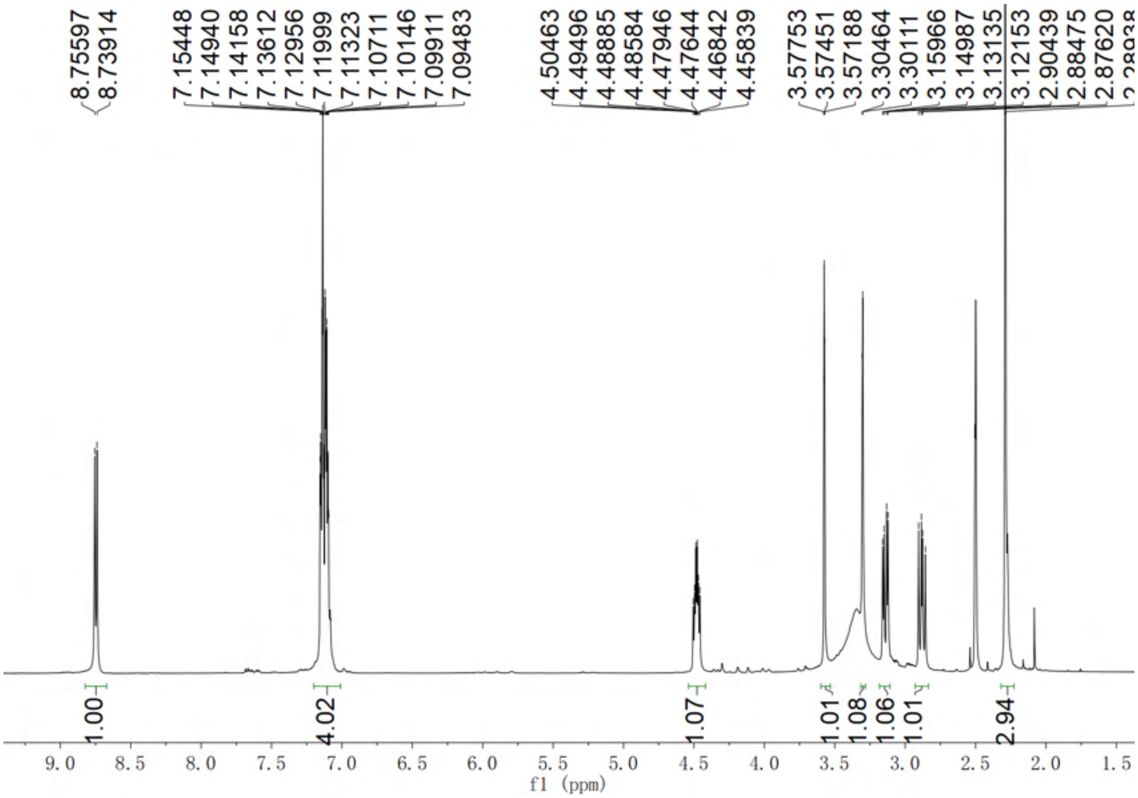

Supplementary Fig. 227. **^{1}H NMR spectrum of compound (2*S*,3*S*)-*t*-ES-a22 in DMSO-d_6**

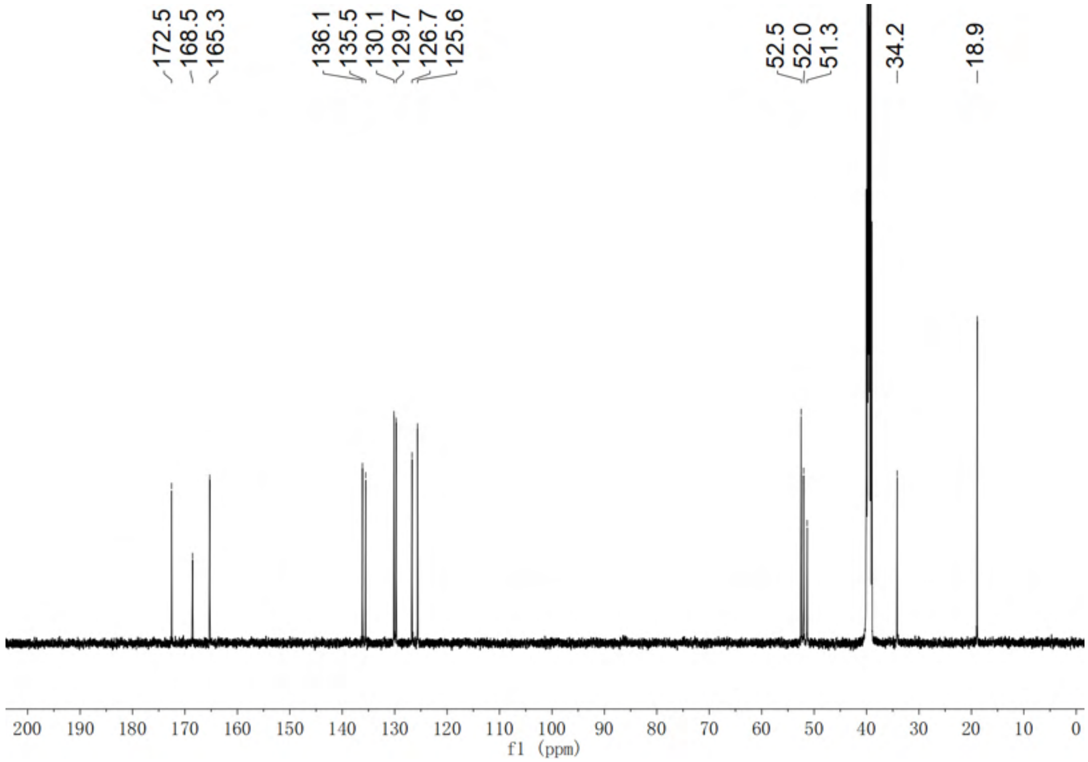

Supplementary Fig. 228. **^{13}C NMR spectrum of compound (2*S*,3*S*)-*t*-ES-a22 in DMSO-d_6**

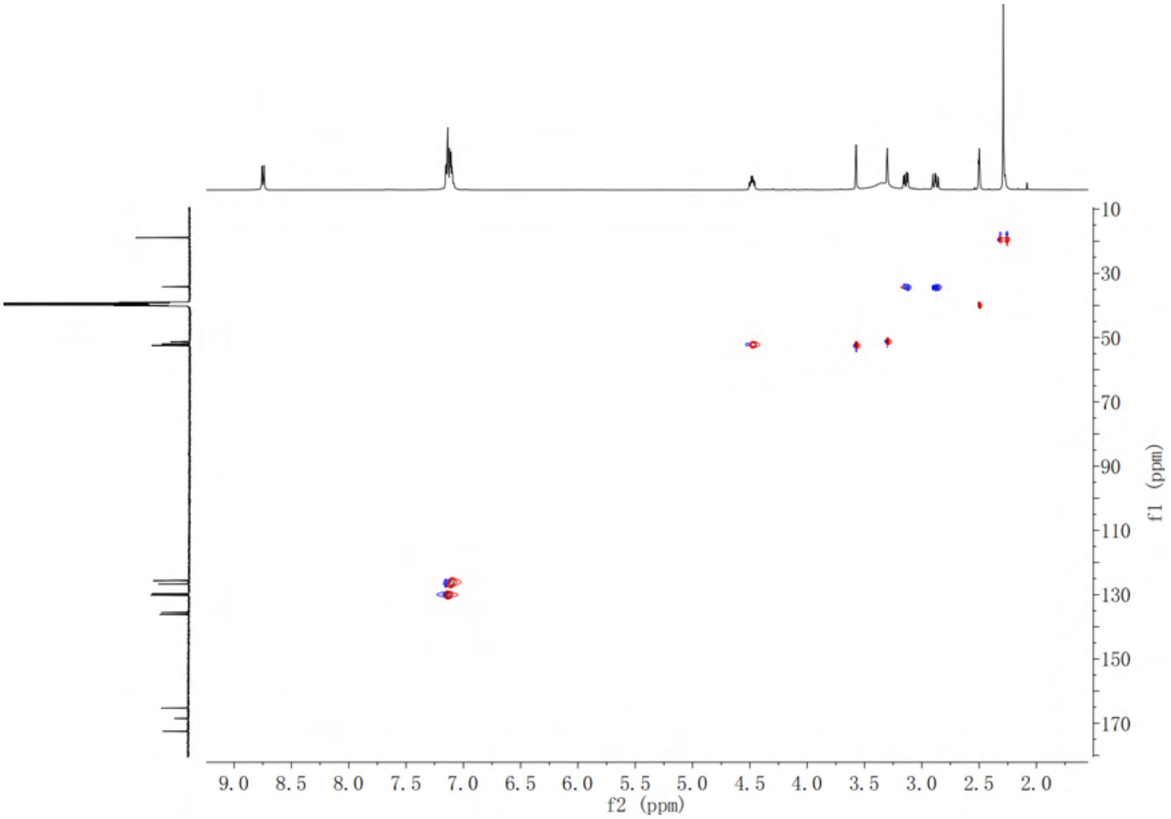

Supplementary Fig. 229. HSQC spectrum of compound (2*S*,3*S*)-*t*-ES-a22 in DMSO-*d*$_6$

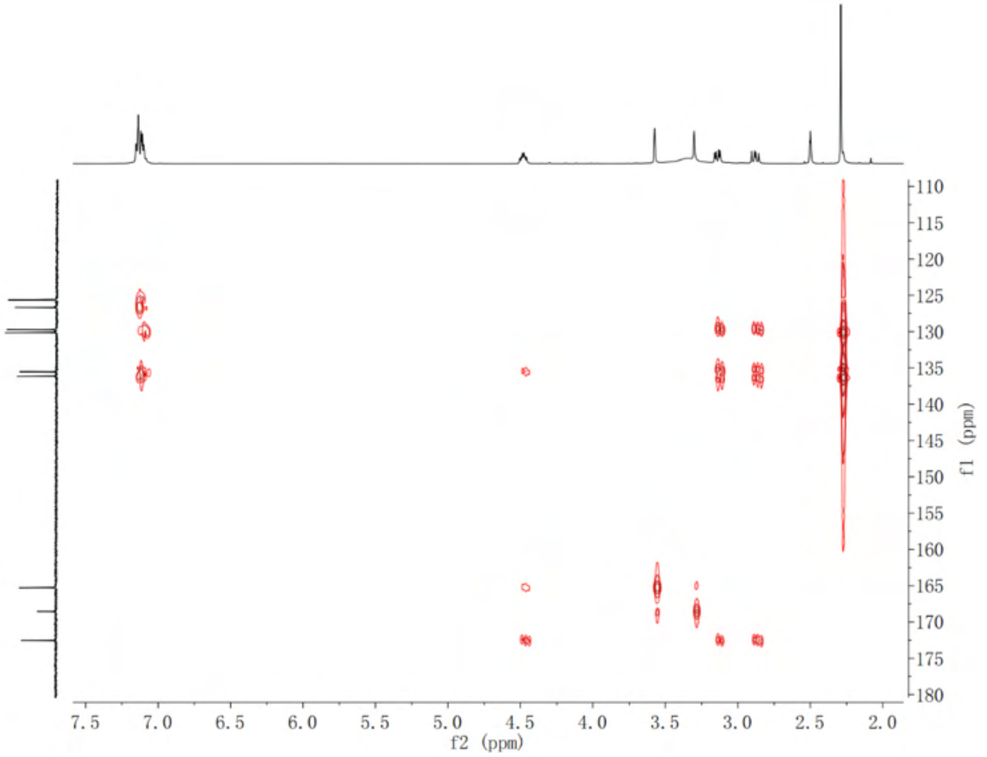

Supplementary Fig. 230. HMBC spectrum of compound (2*S*,3*S*)-*t*-ES-a22 in DMSO-*d*$_6$

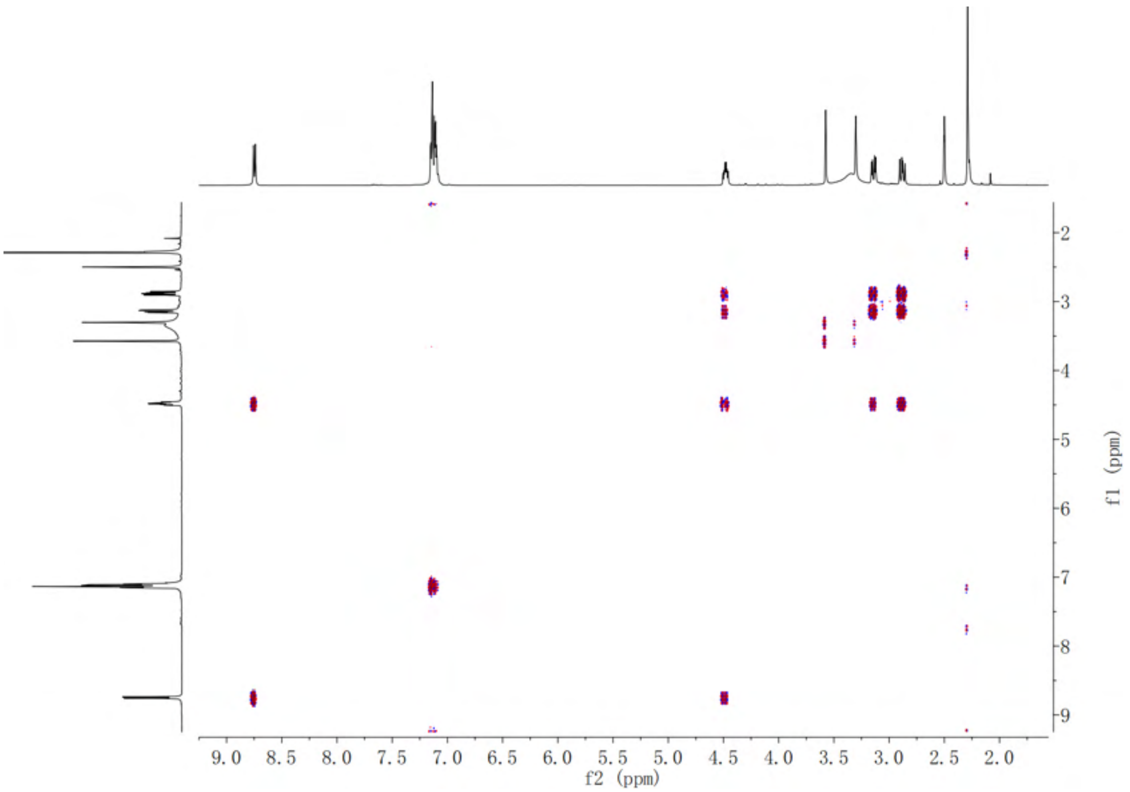

Supplementary Fig. 231. **^{1}H-^{1}H COSY spectrum of compound (2*S*,3*S*)-*t*-ES-a22 in DMSO-*d*$_6$**

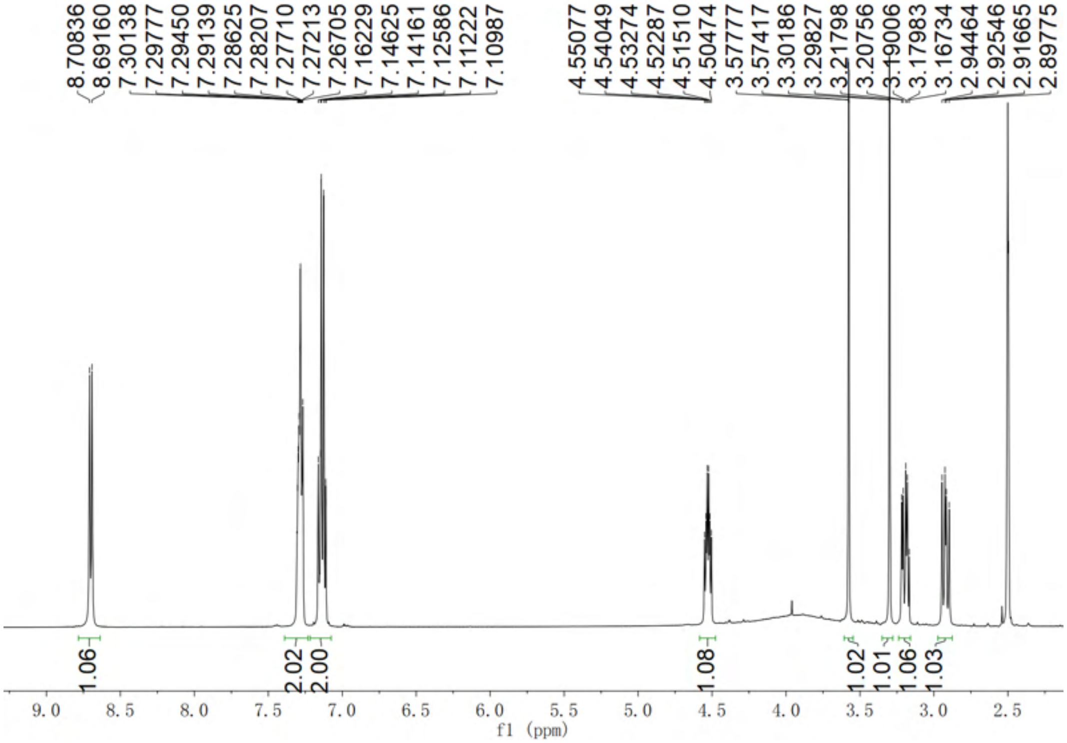

Supplementary Fig. 232. **^{1}H NMR spectrum of compound (2*S*,3*S*)-*t*-ES-a23 in DMSO-*d*$_6$**

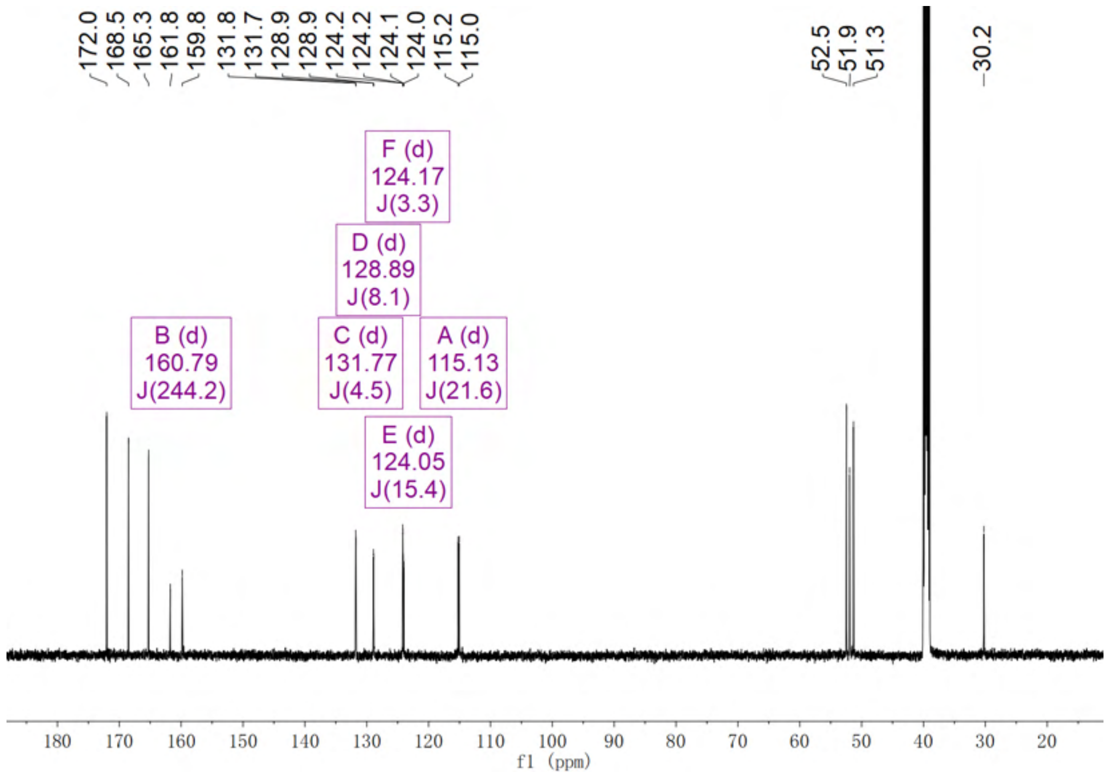

Supplementary Fig. 233. ^{13}**C NMR spectrum of compound (2*S*,3*S*)-*t*-ES-a23 in DMSO-*d*$_6$**

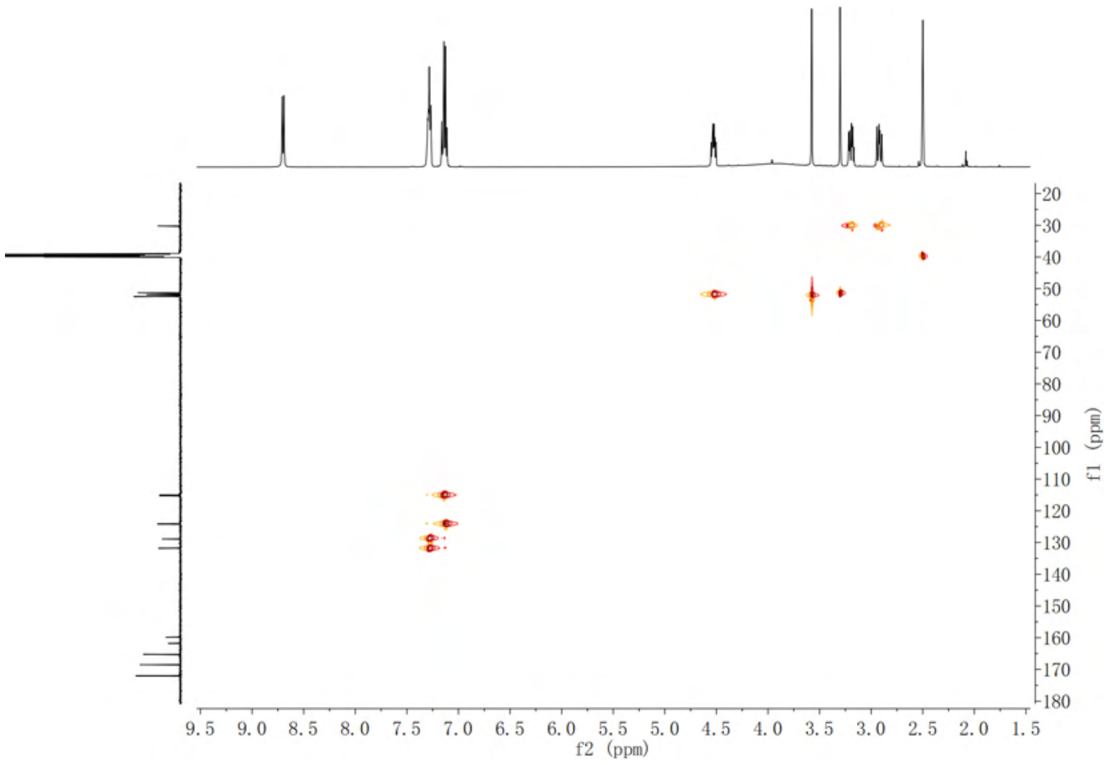

Supplementary Fig. 234. HSQC spectrum of compound (2*S*,3*S*)-*t*-ES-a23 in DMSO-*d*$_6$

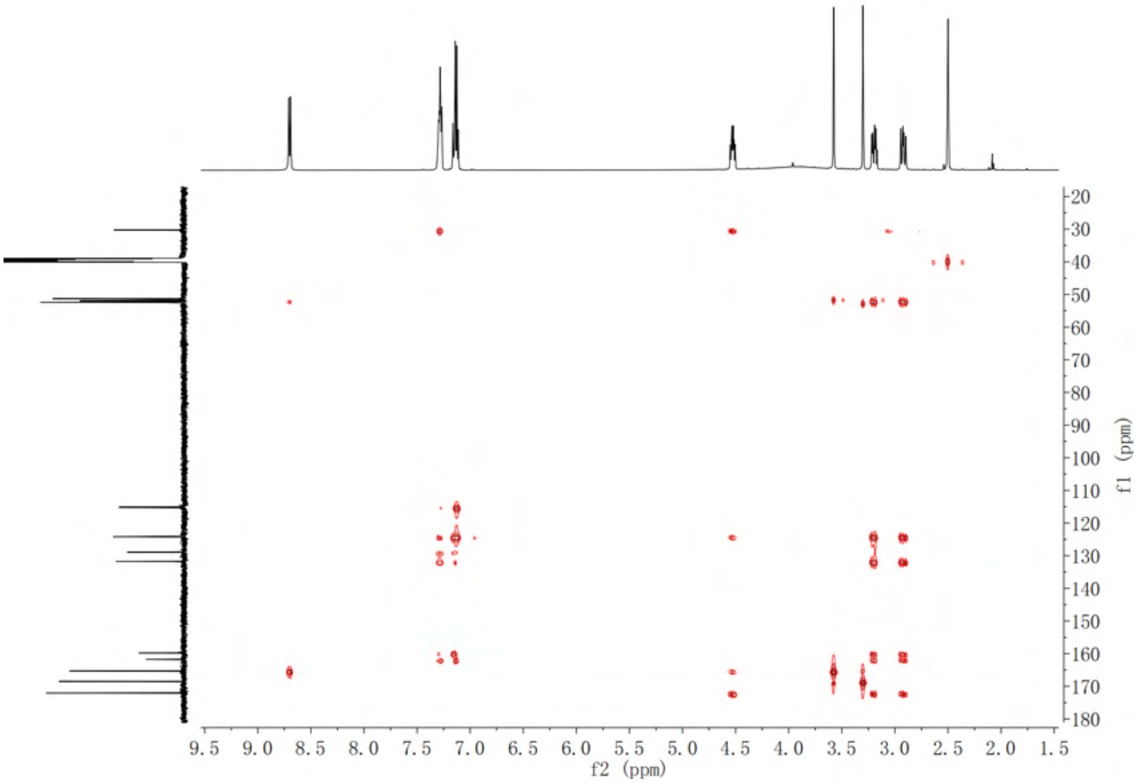

Supplementary Fig. 235. HMBC spectrum of compound (2*S*,3*S*)-*t*-ES-a23 in DMSO-d_6

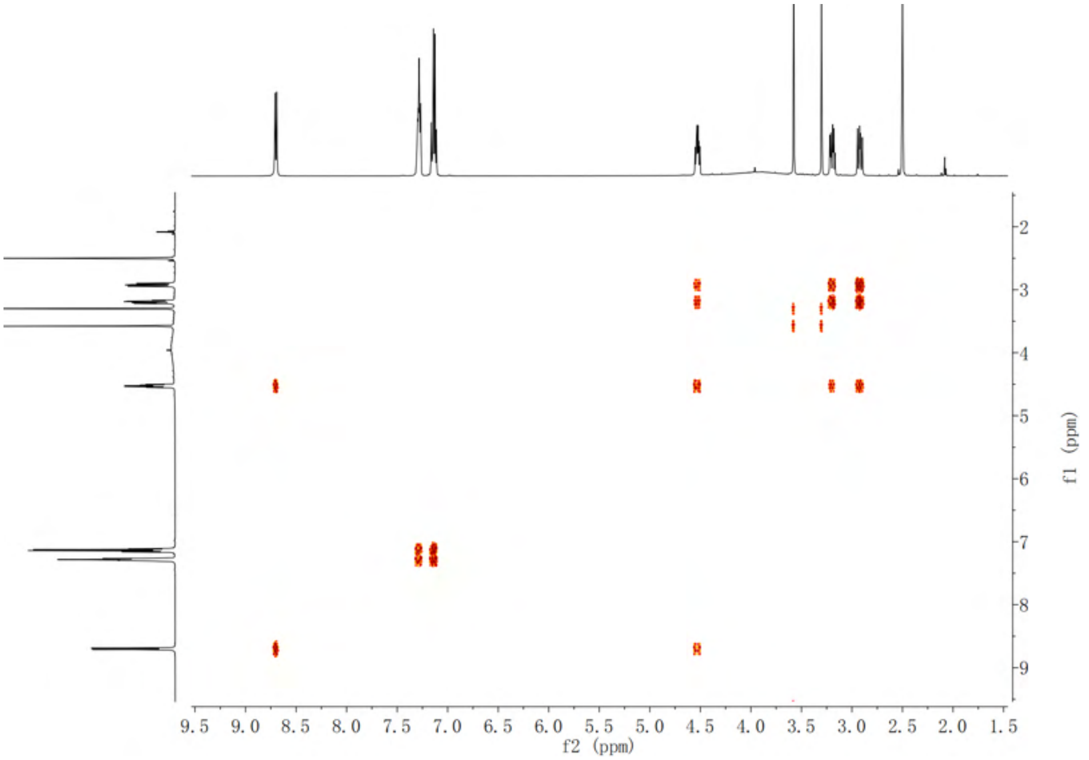

Supplementary Fig. 236. ^{1}H-^{1}H COSY spectrum of compound (2*S*,3*S*)-*t*-ES-a23 in DMSO-d_6

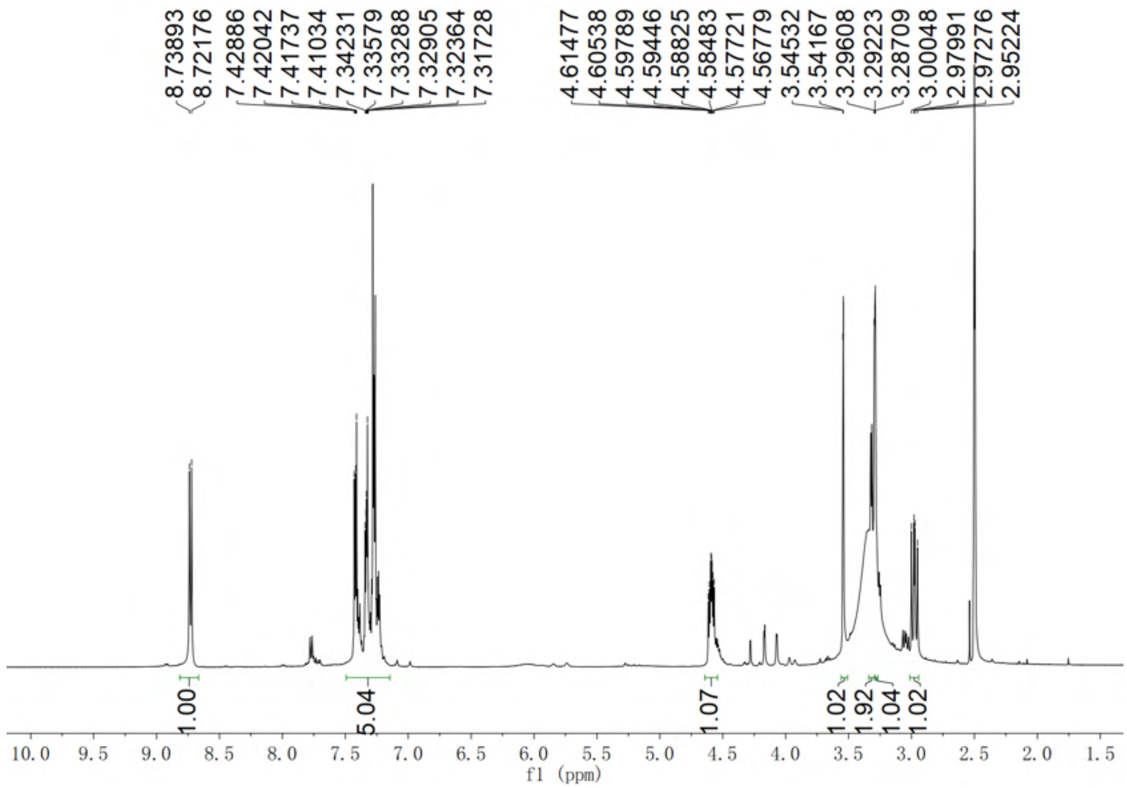

Supplementary Fig. 237. [1]**H NMR spectrum of compound (2*S*,3*S*)-*t*-ES-a24 in DMSO-*d*$_6$**

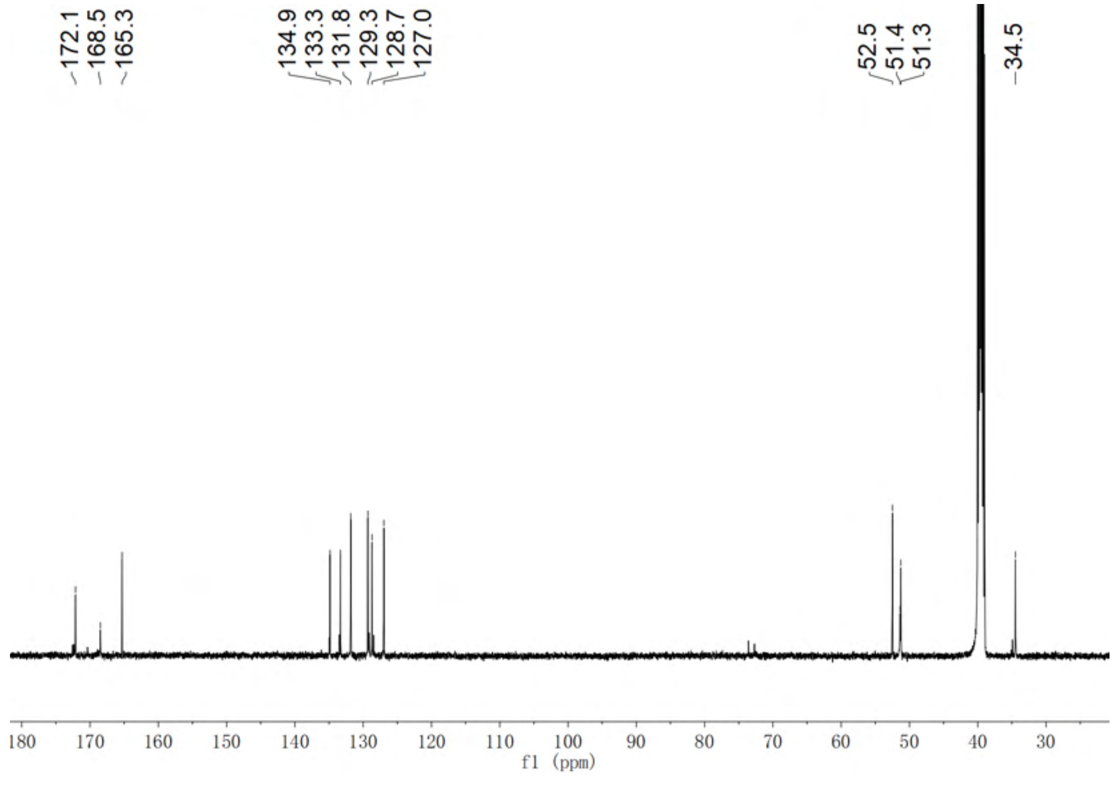

Supplementary Fig. 238. [13]**C NMR spectrum of compound (2*S*,3*S*)-*t*-ES-a24 in DMSO-*d*$_6$**

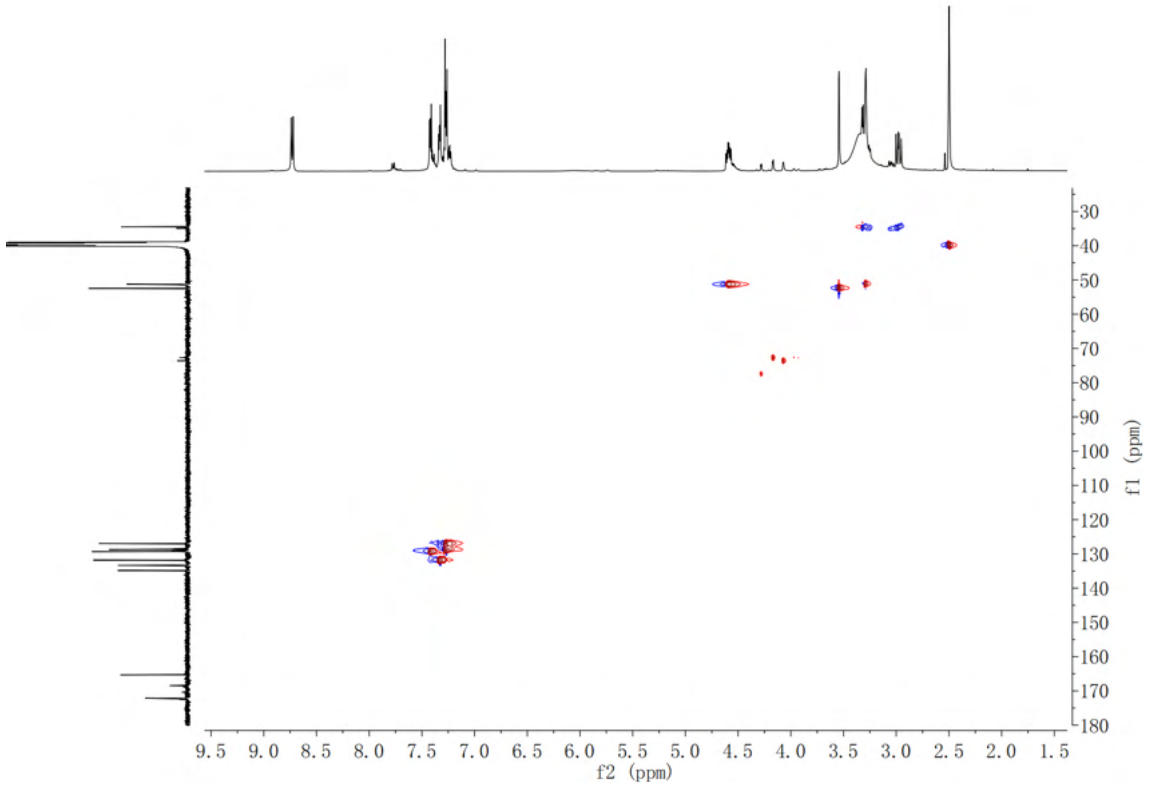

Supplementary Fig. 239. HSQC spectrum of compound (2*S*,3*S*)-*t*-ES-a24 in DMSO-*d*$_6$

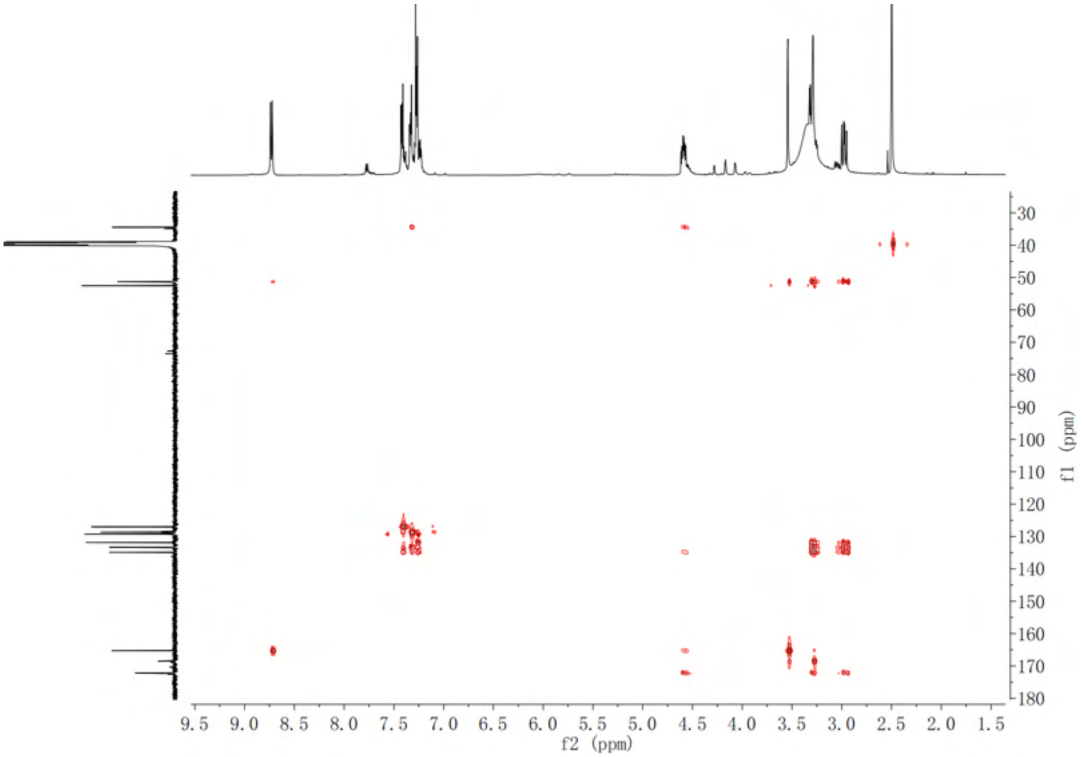

Supplementary Fig. 240. HMBC spectrum of compound (2*S*,3*S*)-*t*-ES-a24 in DMSO-*d*$_6$

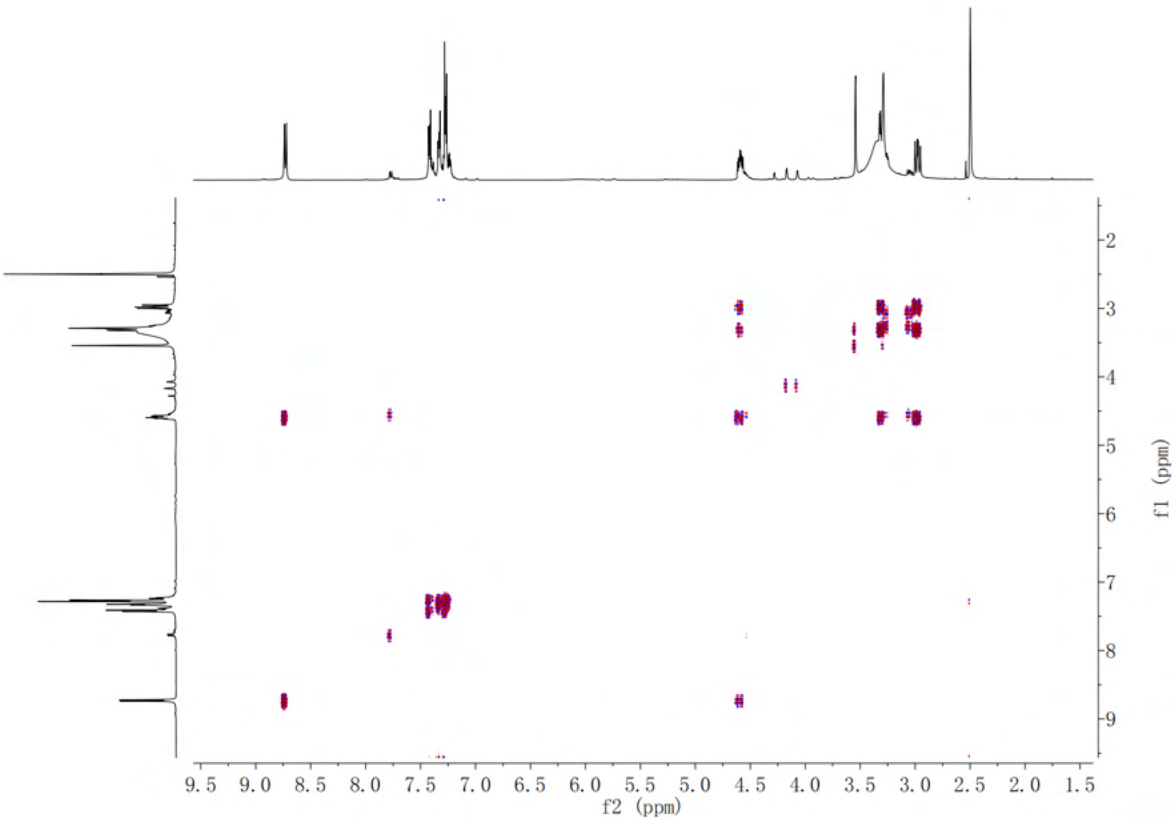

Supplementary Fig. 241. ^{1}H-^{1}H COSY spectrum of compound (2*S*,3*S*)-*t*-ES-a24 in DMSO-d_6

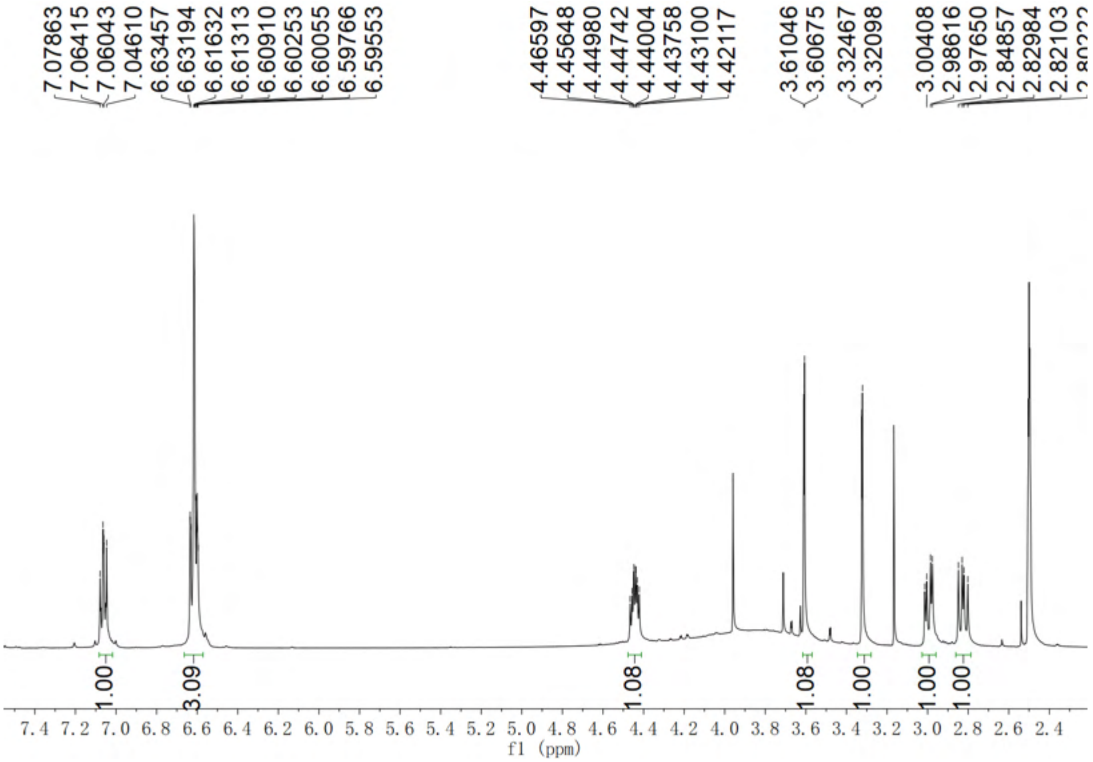

Supplementary Fig. 242. ^{1}H NMR spectrum of compound (2*S*,3*S*)-*t*-ES-a25 in DMSO-d_6

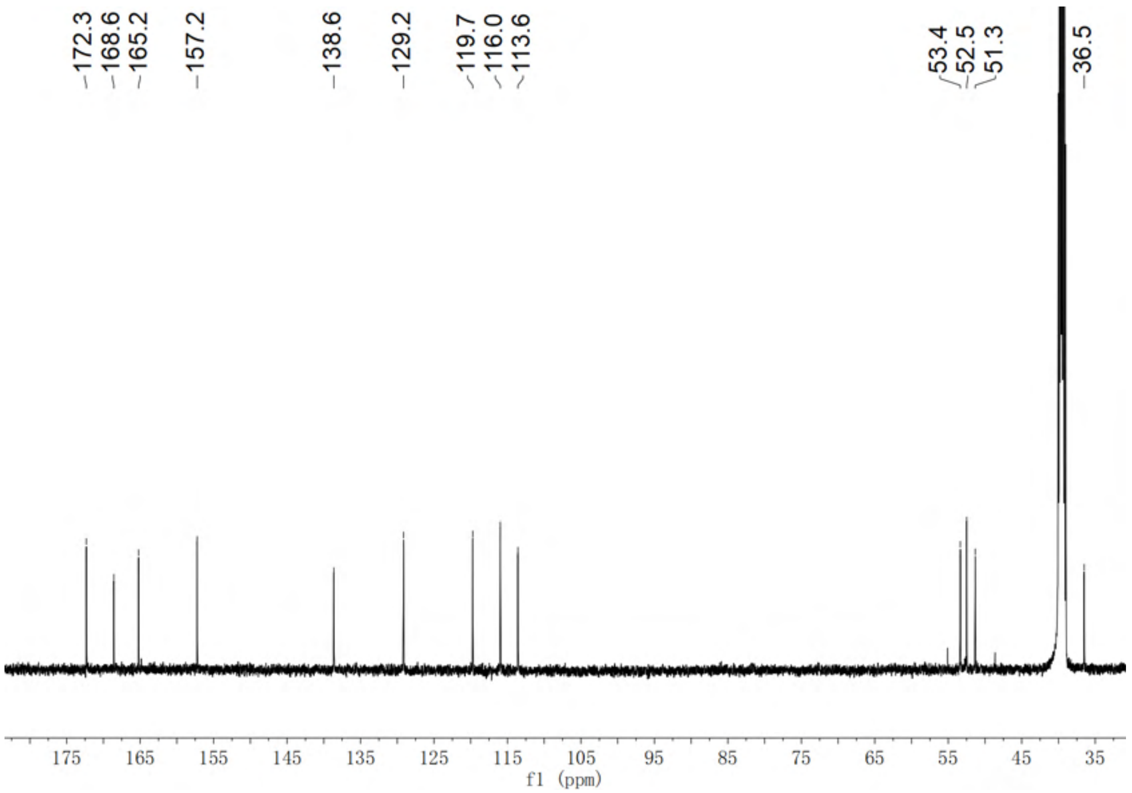

Supplementary Fig. 243. ^{13}C NMR spectrum of compound (2*S*,3*S*)-*t*-ES-a25 in DMSO-*d*$_6$

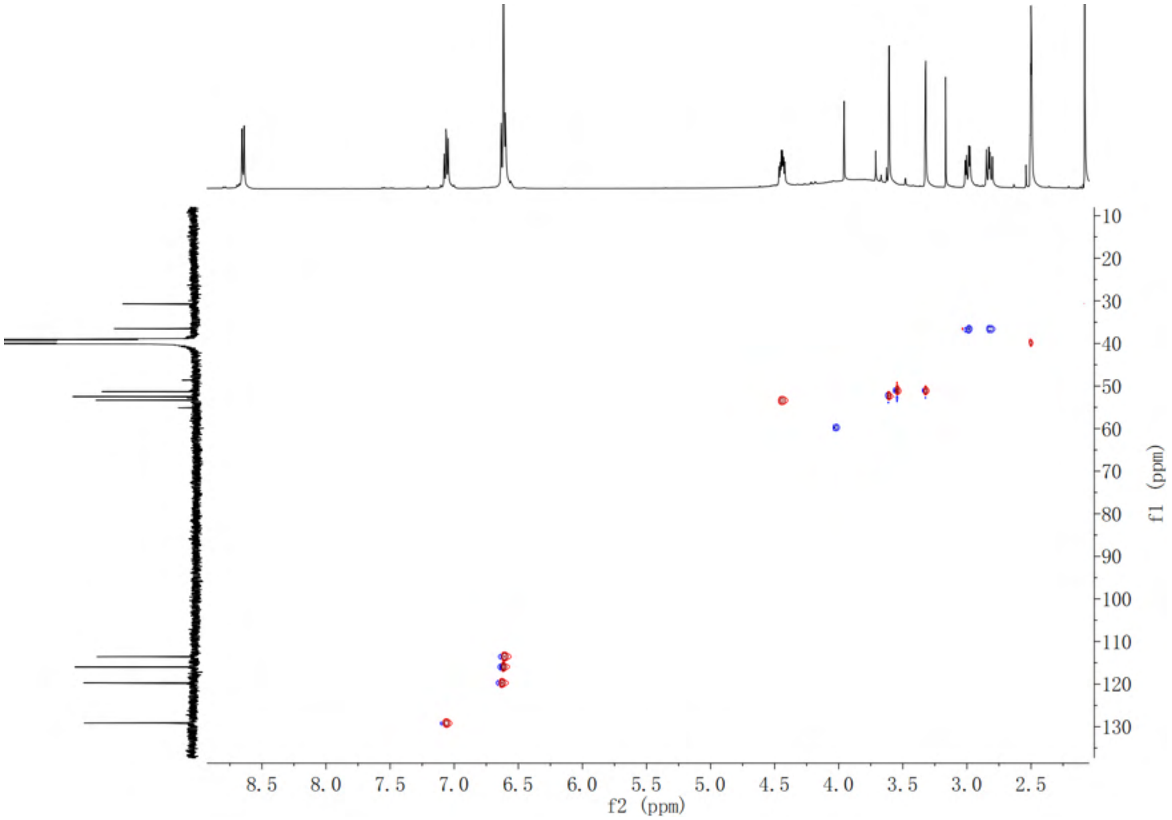

Supplementary Fig. 244. HSQC spectrum of compound (2*S*,3*S*)-*t*-ES-a25 in DMSO-*d*$_6$

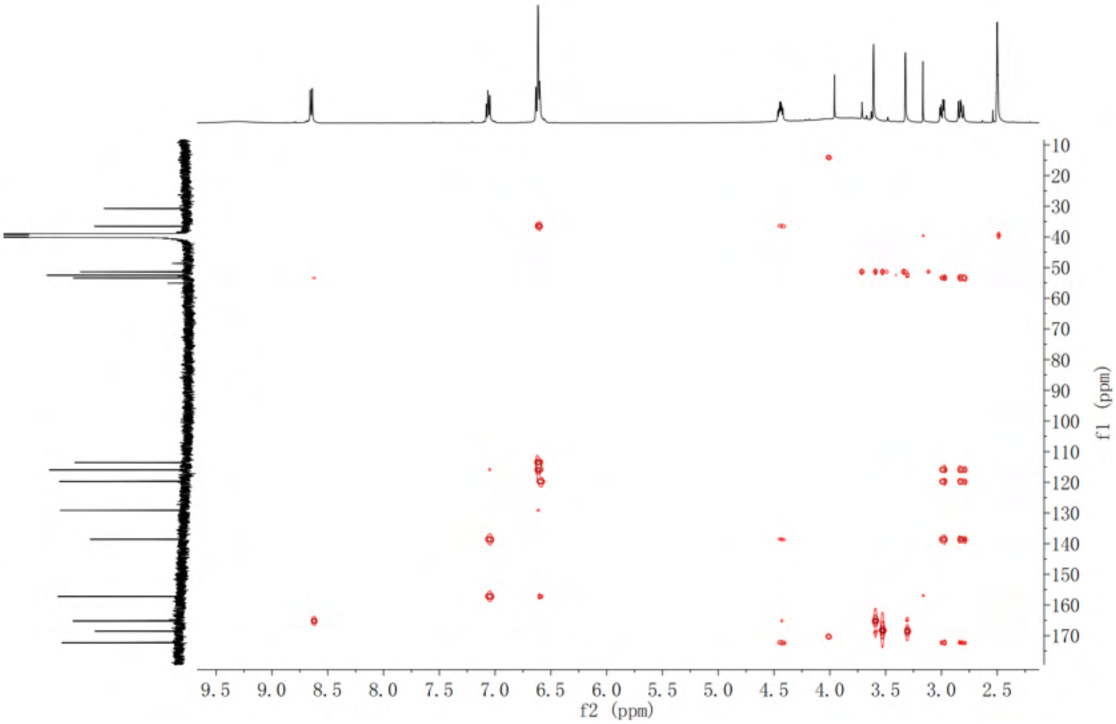

Supplementary Fig. 245. HMBC spectrum of compound (2*S*,3*S*)-*t*-ES-a25 in DMSO-d_6

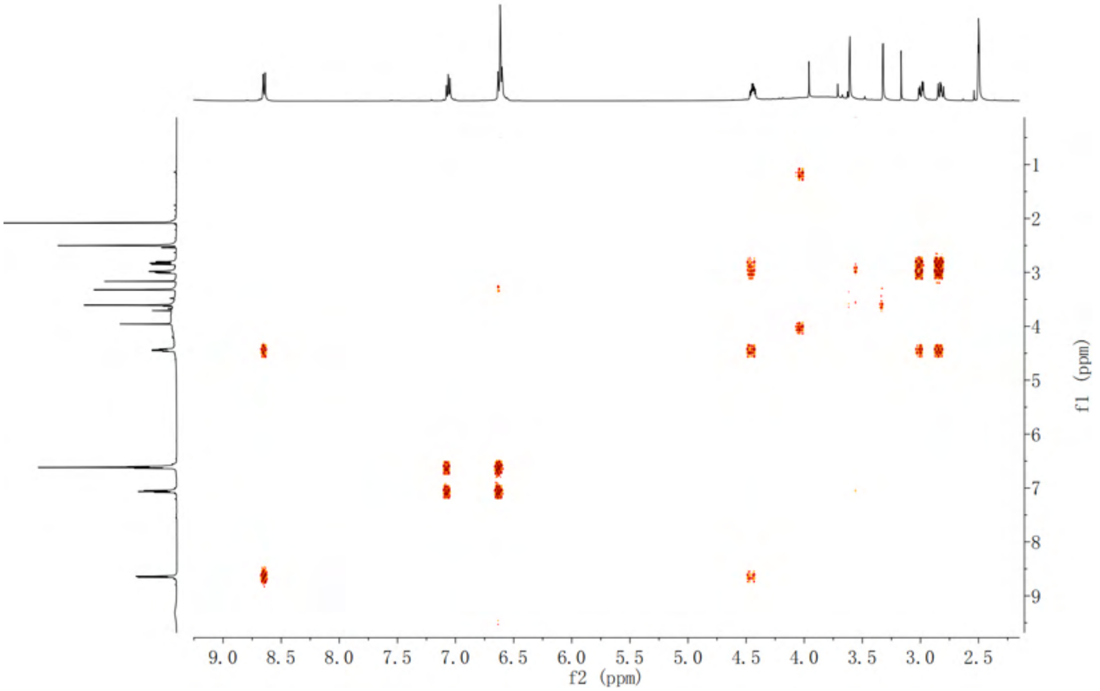

Supplementary Fig. 246. ^{1}H-^{1}H COSY spectrum of compound (2*S*,3*S*)-*t*-ES-a25 in DMSO-d_6

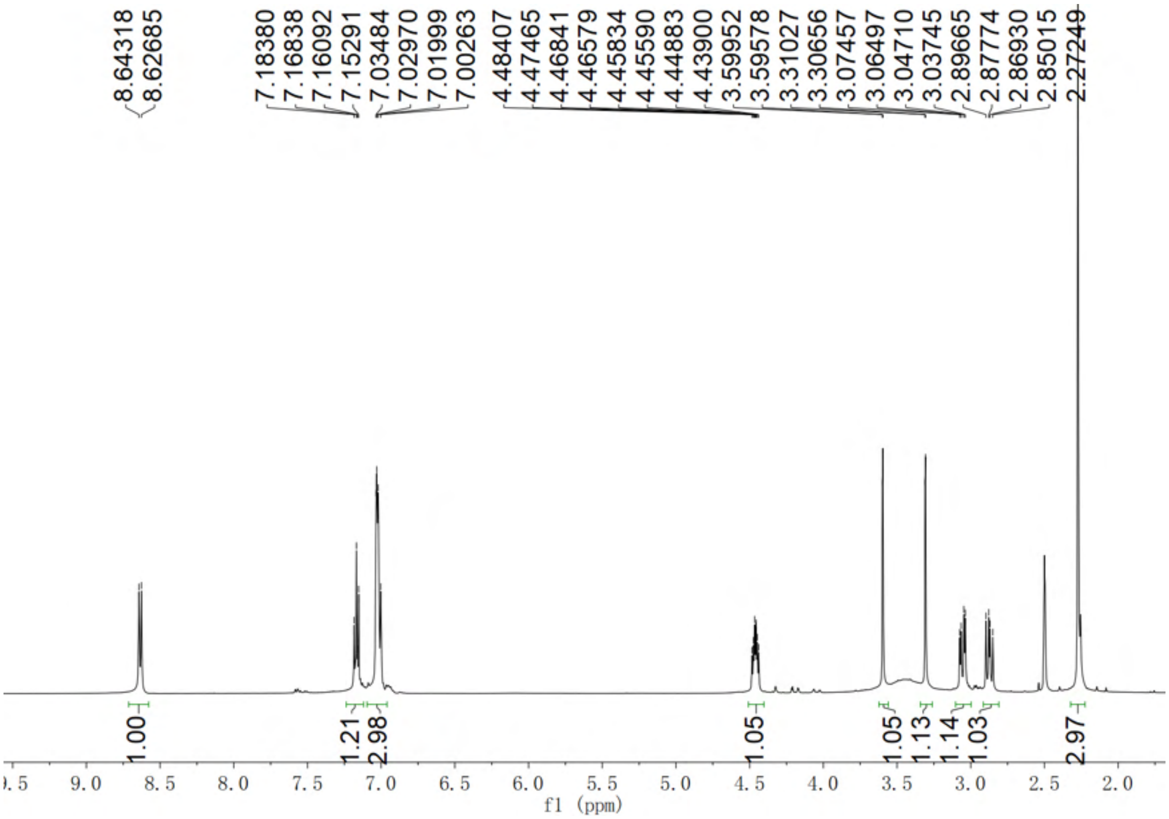

Supplementary Fig. 247. ^{1}H NMR spectrum of compound (2*S*,3*S*)-*t*-ES-a26 in DMSO-d_6

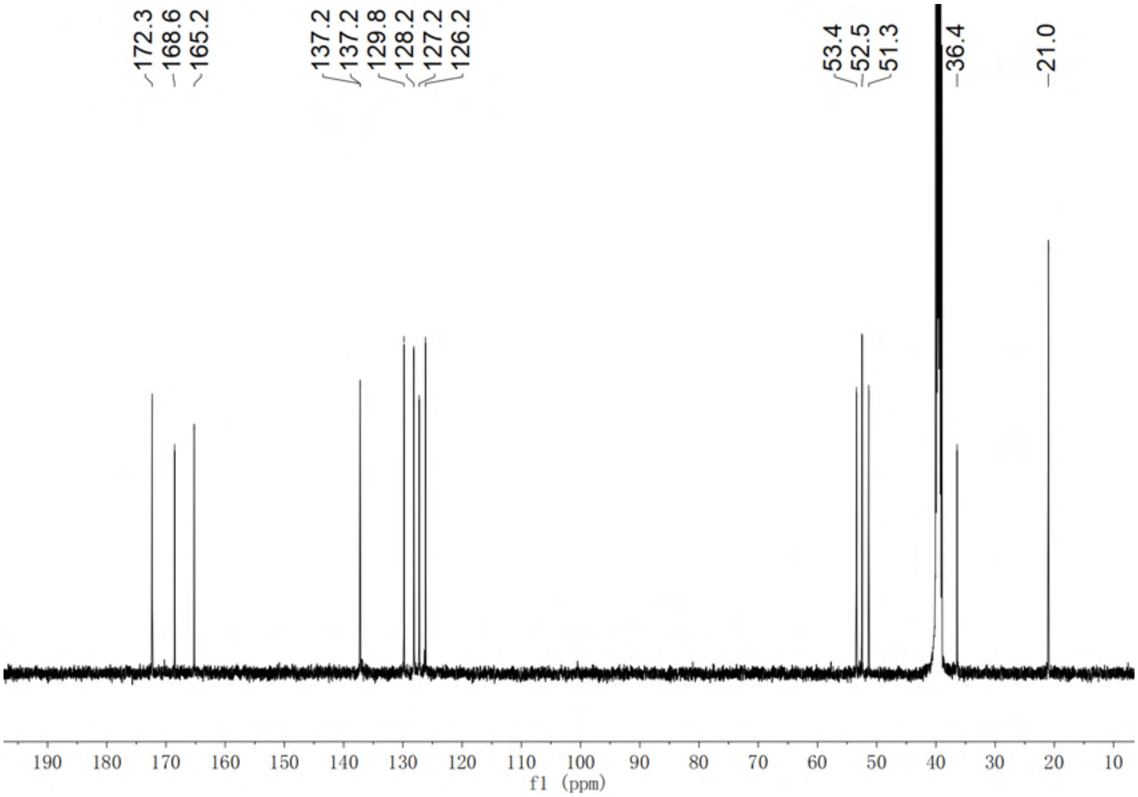

Supplementary Fig. 248. ^{13}C NMR spectrum of compound (2*S*,3*S*)-*t*-ES-a26 in DMSO-d_6

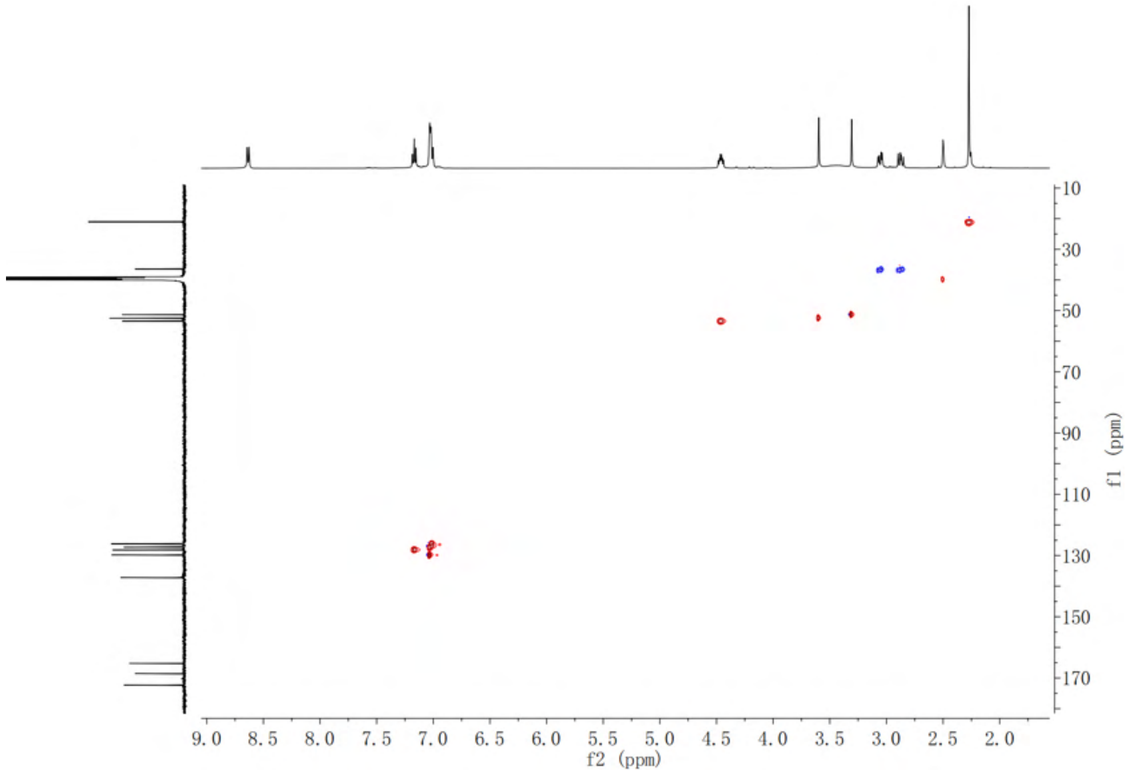

Supplementary Fig. 249. HSQC spectrum of compound (2*S*,3*S*)-*t*-ES-a26 in DMSO-d_6

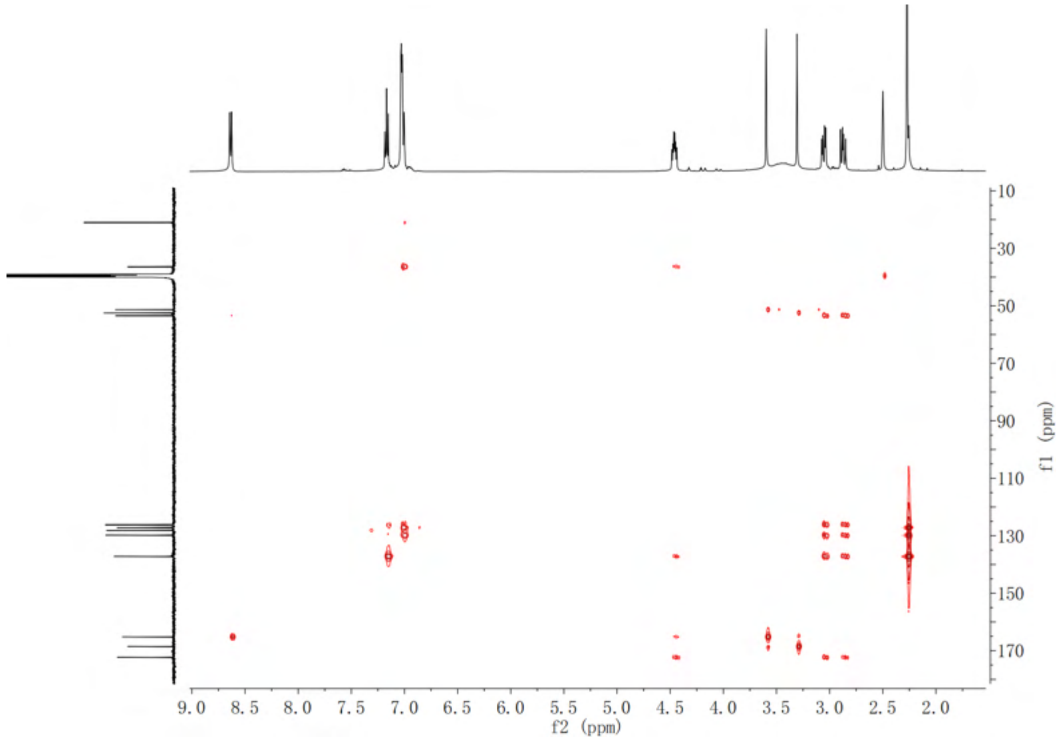

Supplementary Fig. 250. HMBC spectrum of compound (2*S*,3*S*)-*t*-ES-a26 in DMSO-d_6

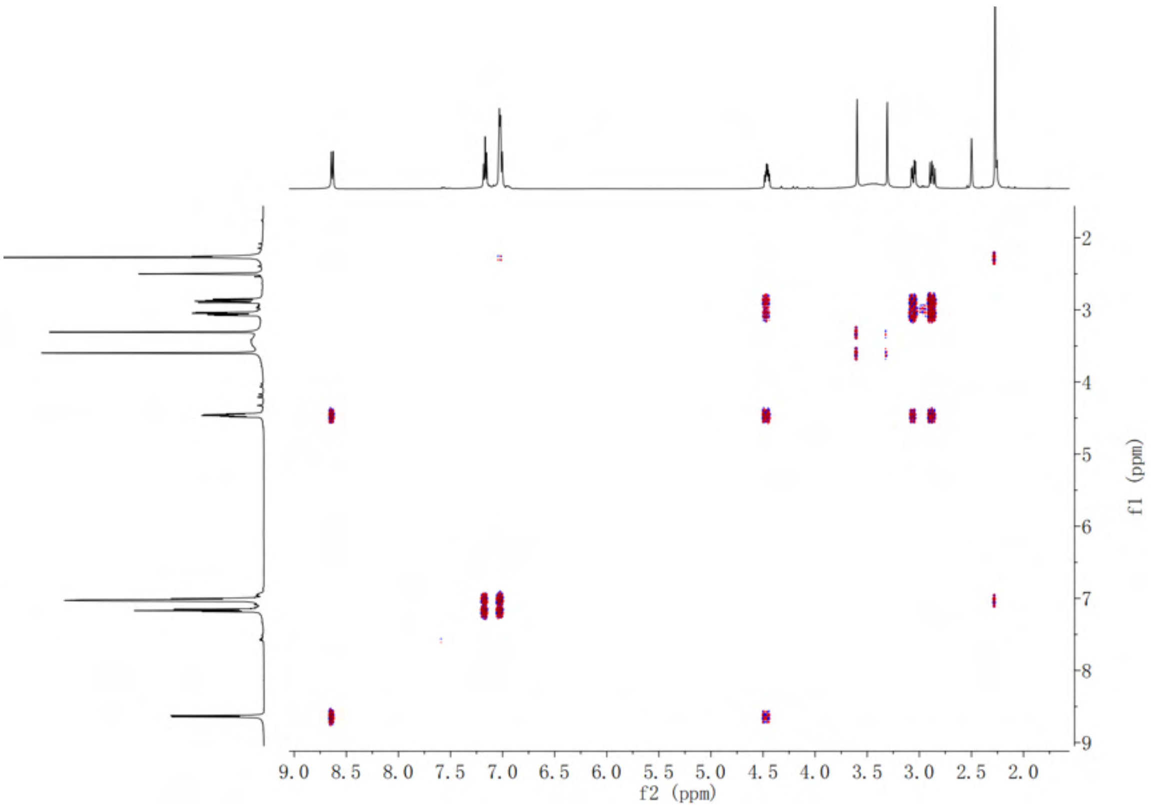

Supplementary Fig. 251. ^1H-^1H **COSY spectrum of compound (2*S*,3*S*)-*t*-ES-a26 in DMSO-*d*$_6$**

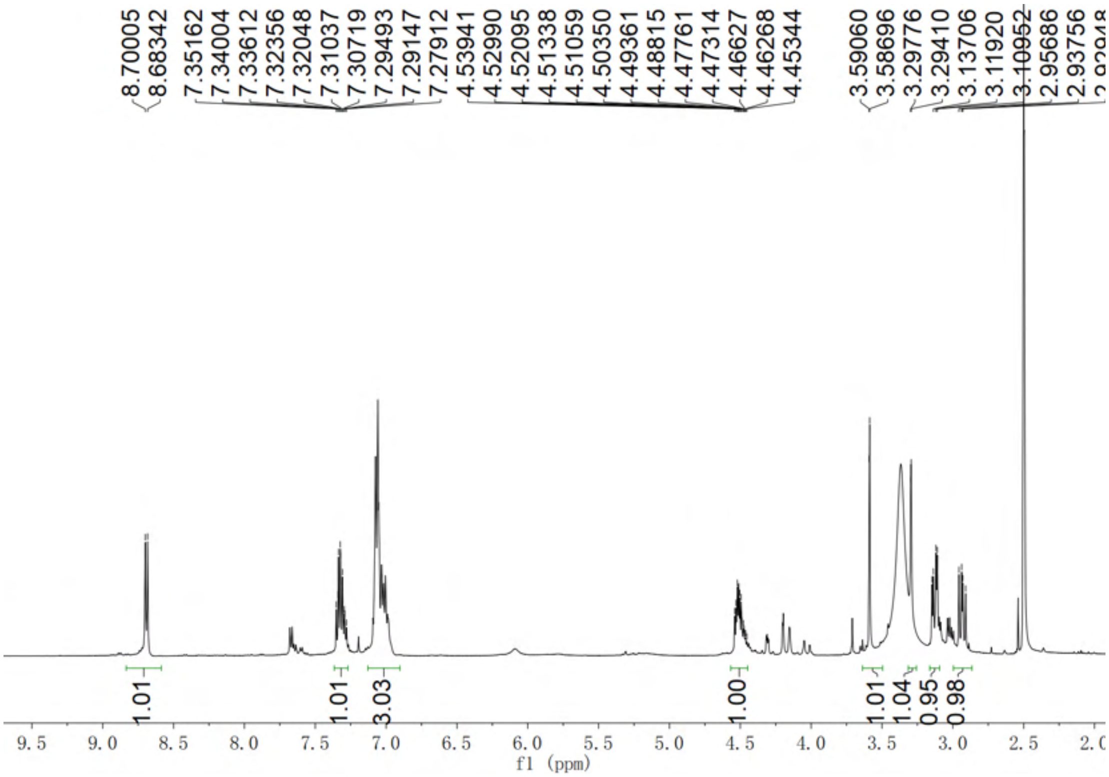

Supplementary Fig. 252. ^1H **NMR spectrum of compound (2*S*,3*S*)-*t*-ES-a27 in DMSO-*d*$_6$**

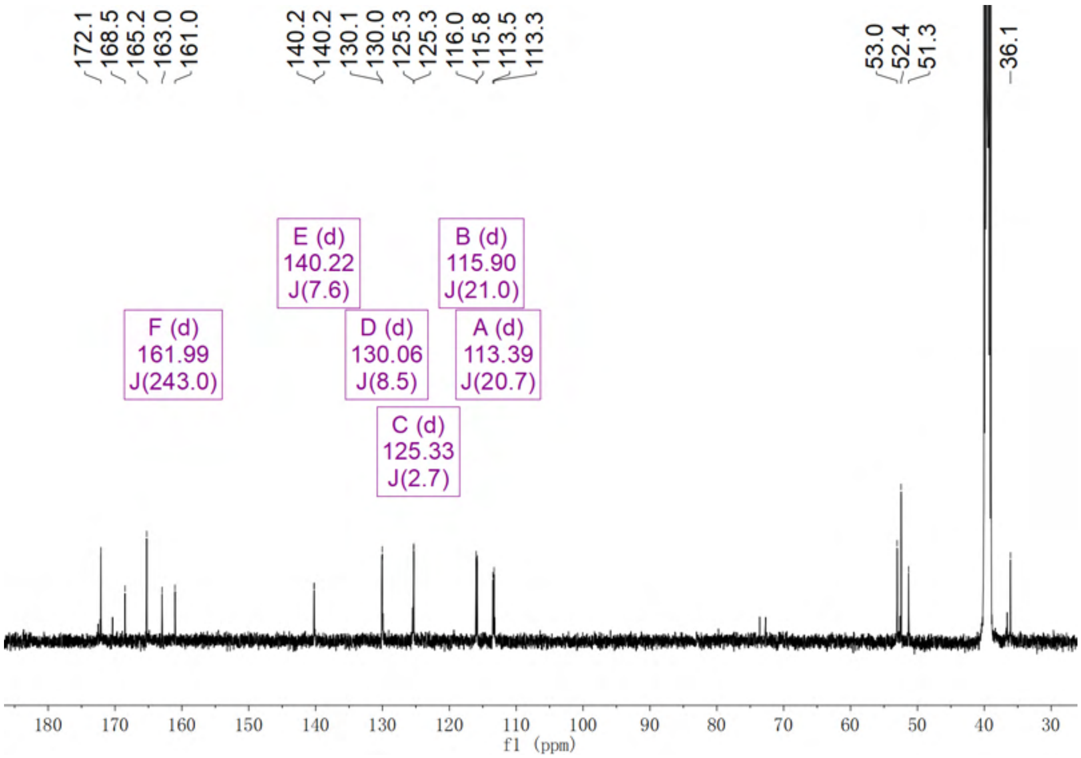

Supplementary Fig. 253. ^{13}**C NMR spectrum of compound (2*S*,3*S*)-*t*-ES-a27 in DMSO-*d*$_6$**

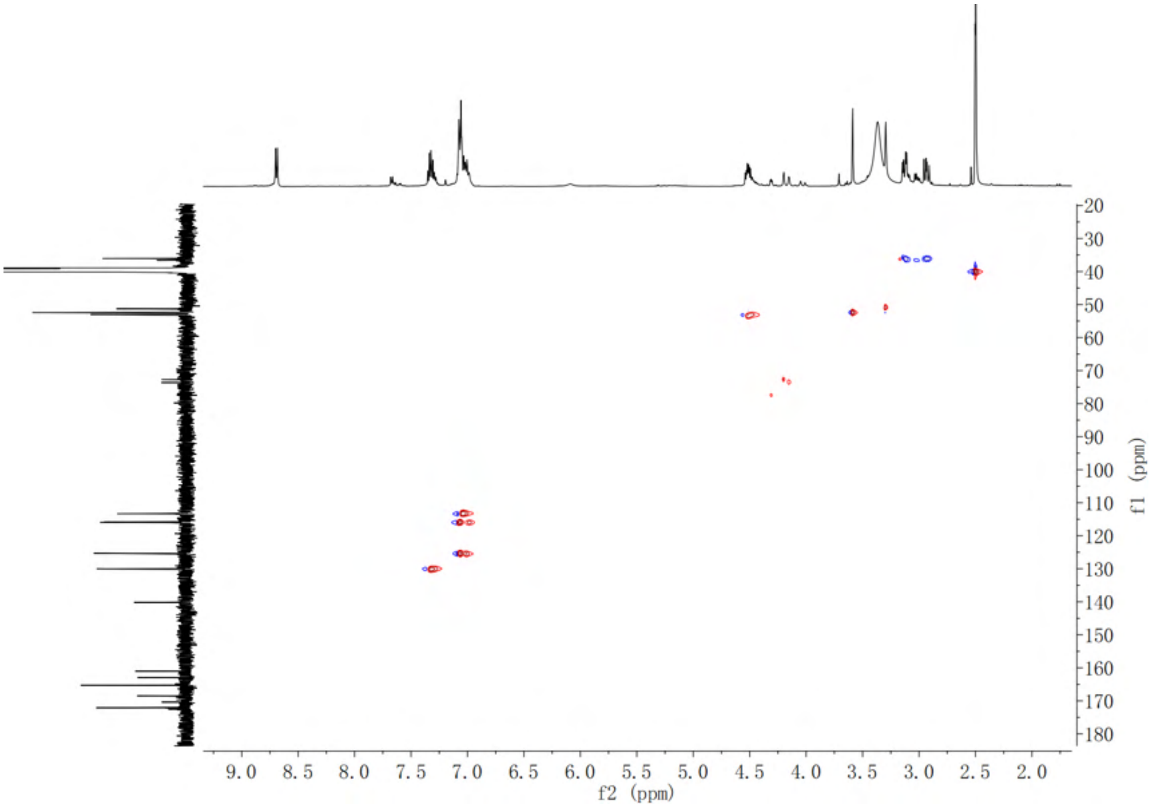

Supplementary Fig. 254. HSQC spectrum of compound (2*S*,3*S*)-*t*-ES-a27 in DMSO-*d*$_6$

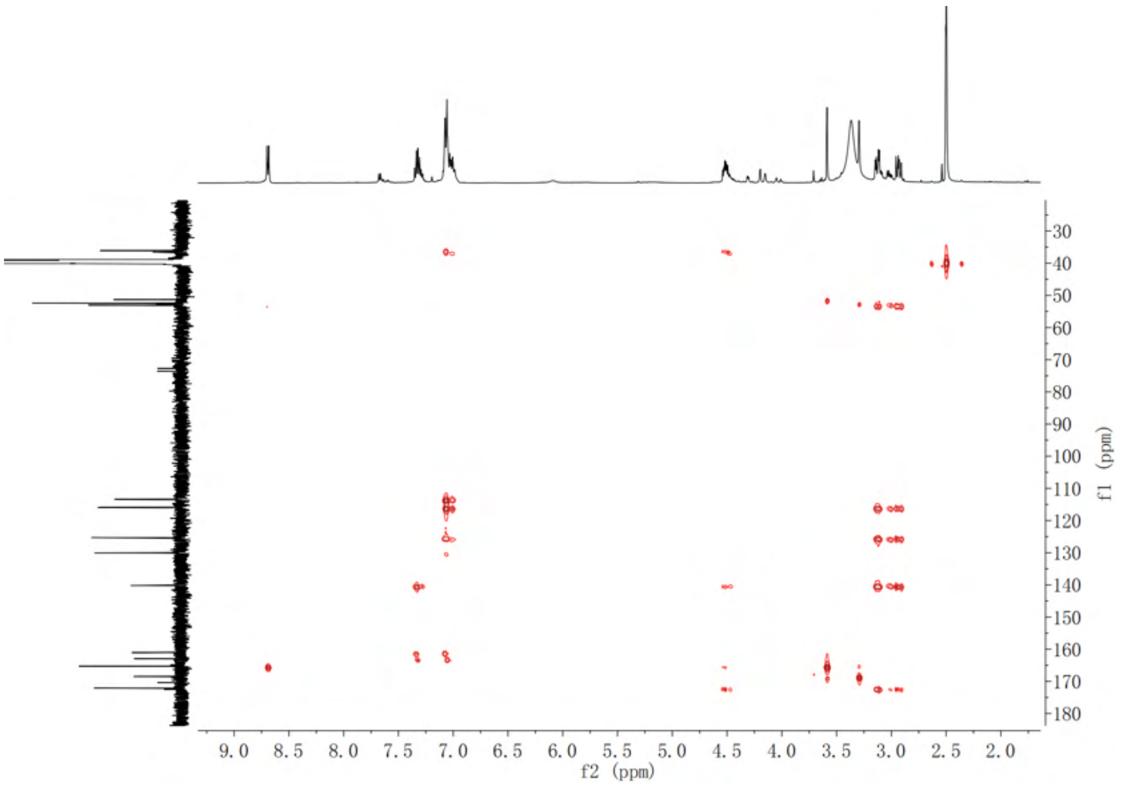

Supplementary Fig. 255. HMBC spectrum of compound (2*S*,3*S*)-*t*-ES-a27 in DMSO-*d*$_6$

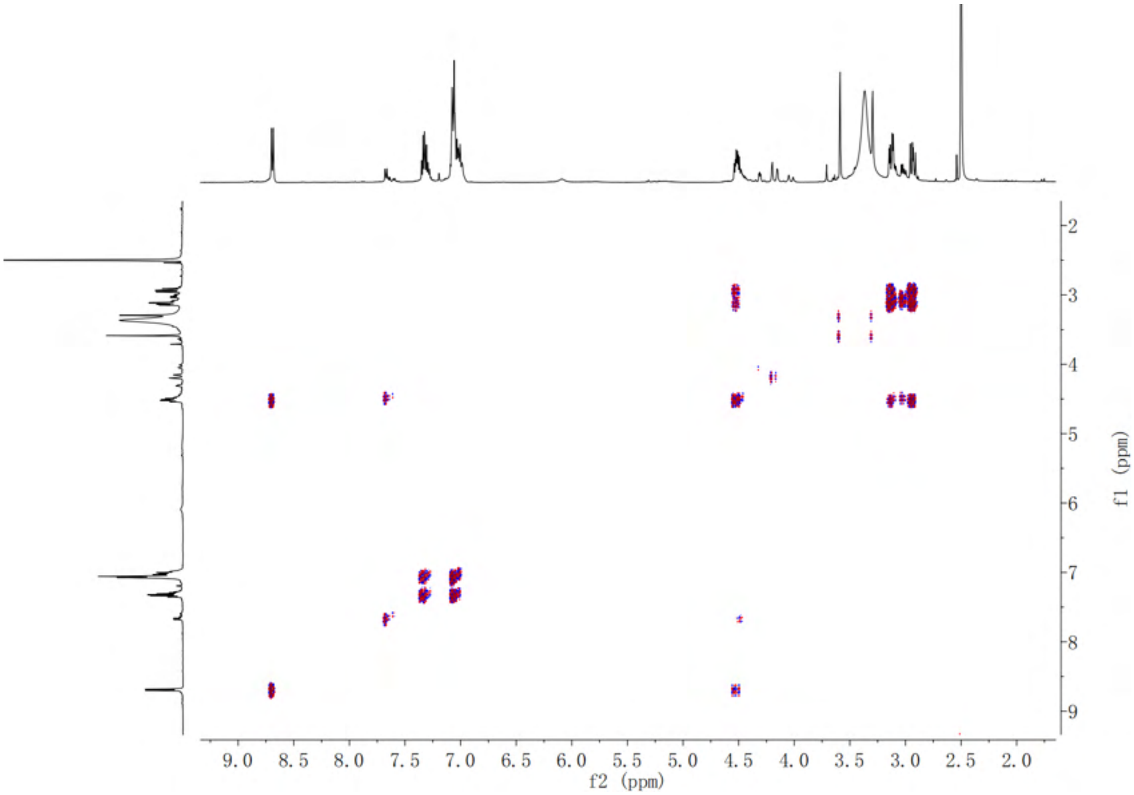

Supplementary Fig. 256. ^{1}H-^{1}H COSY spectrum of compound (2*S*,3*S*)-*t*-ES-a27 in DMSO-*d*$_6$

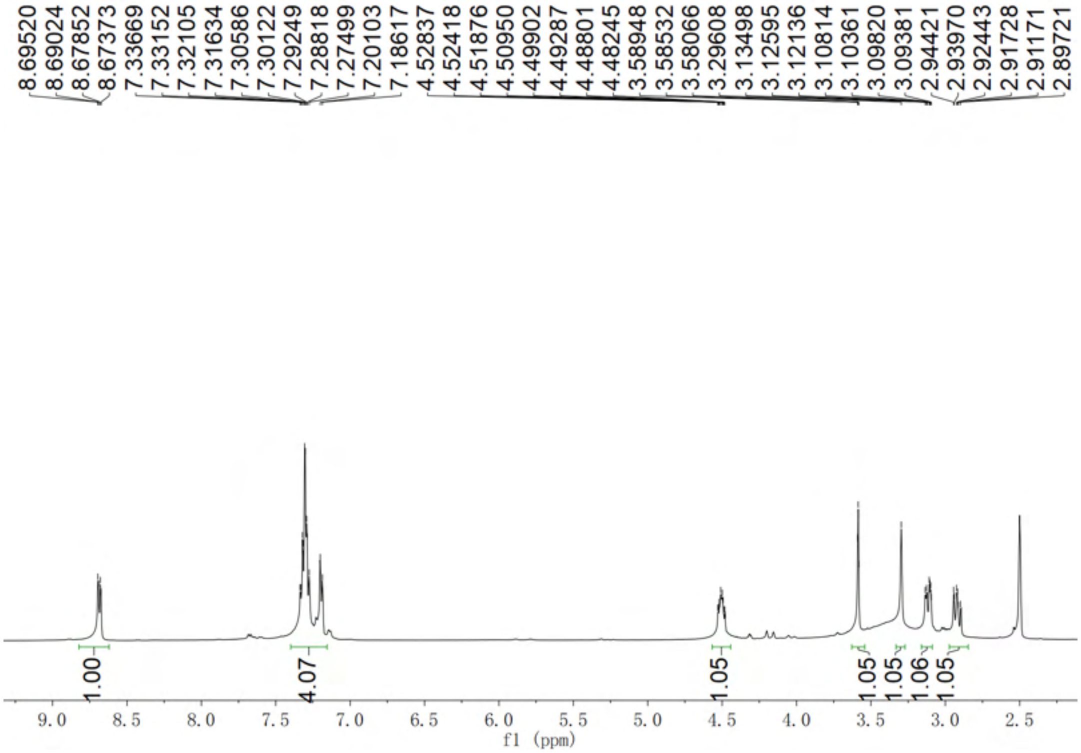

Supplementary Fig. 257. **^{1}H NMR spectrum of compound (2*S*,3*S*)-*t*-ES-a28 in DMSO-d_6**

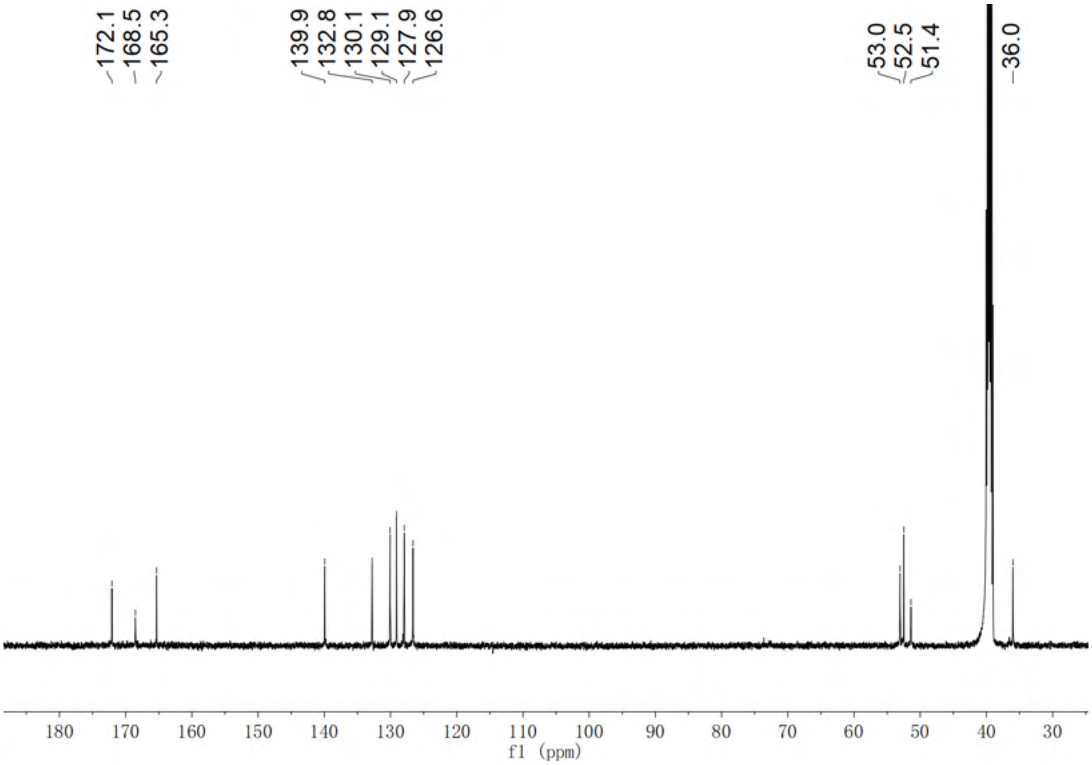

Supplementary Fig. 258. **^{13}C NMR spectrum of compound (2*S*,3*S*)-*t*-ES-a28 in DMSO-d_6**

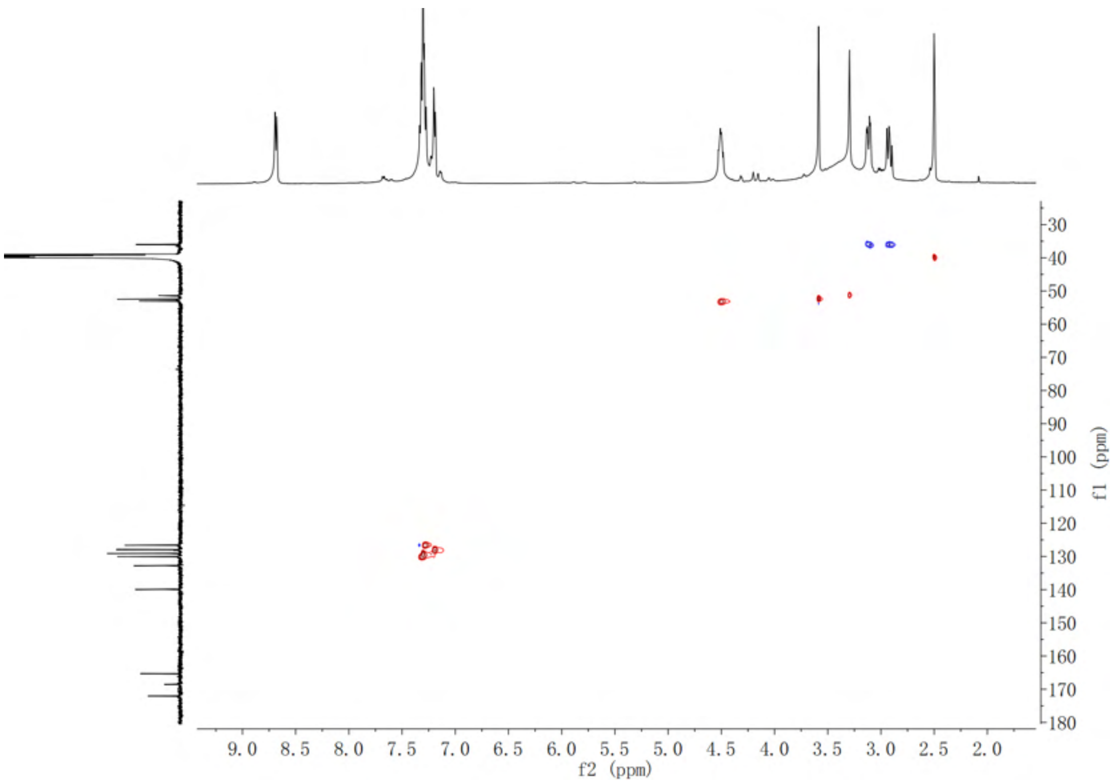

Supplementary Fig. 259. HSQC spectrum of compound (2*S*,3*S*)-*t*-ES-a28 in DMSO-d_6

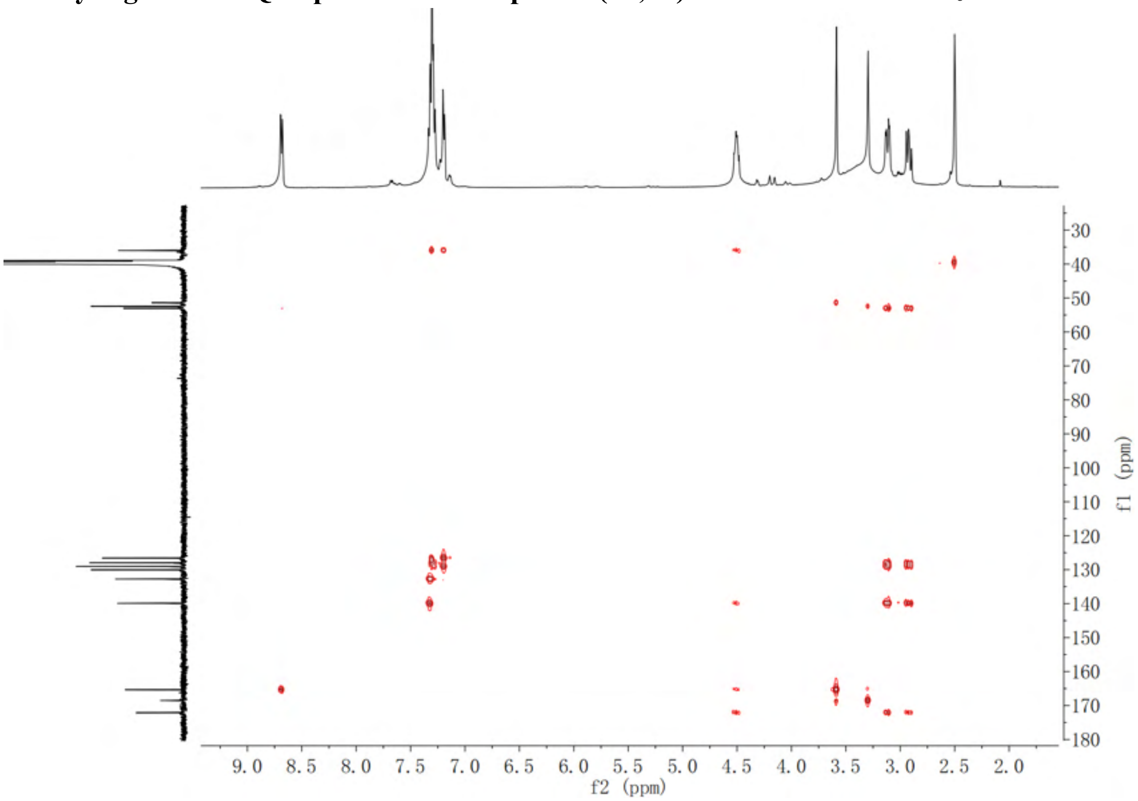

Supplementary Fig. 260. HMBC spectrum of compound (2*S*,3*S*)-*t*-ES-a28 in DMSO-d_6

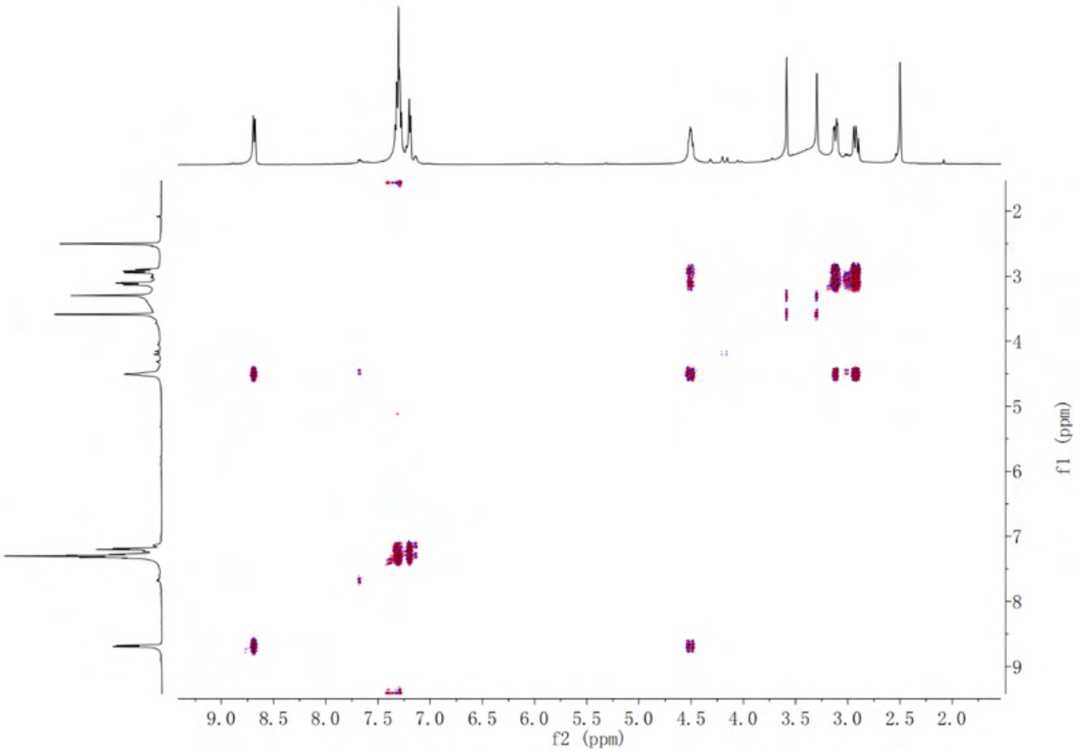

Supplementary Fig. 261. **^{1}H-^{1}H COSY spectrum of compound (2*S*,3*S*)-*t*-ES-a28 in DMSO-*d*$_6$**

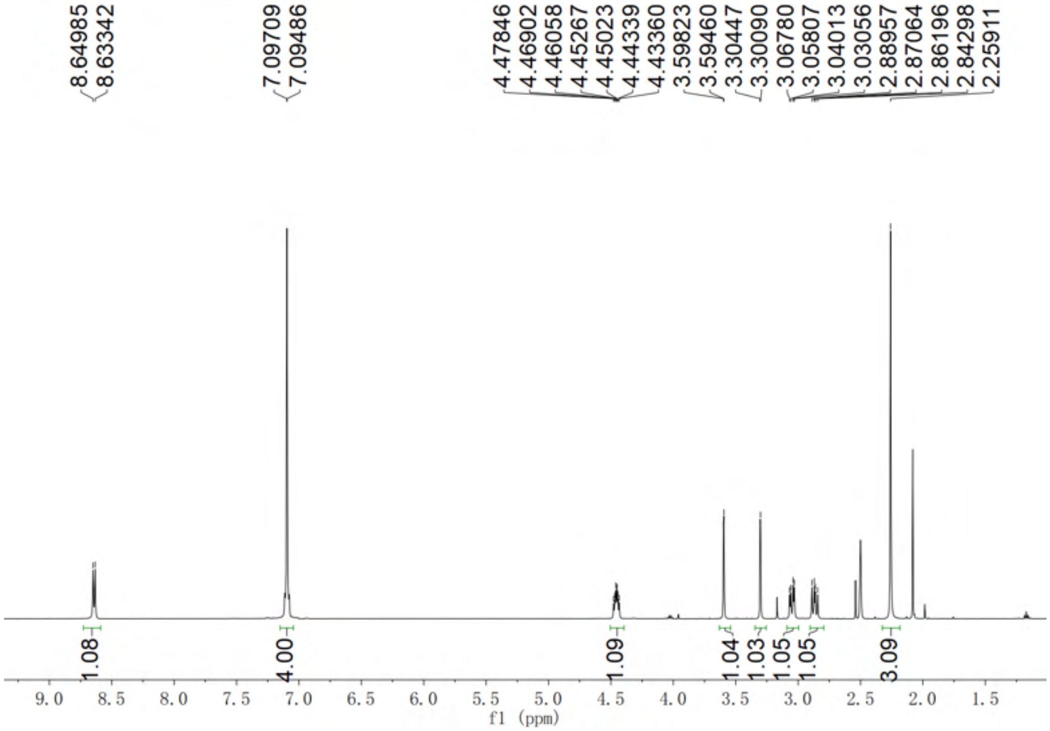

Supplementary Fig. 262. **^{1}H NMR spectrum of compound (2*S*,3*S*)-*t*-ES-a29 in DMSO-*d*$_6$**

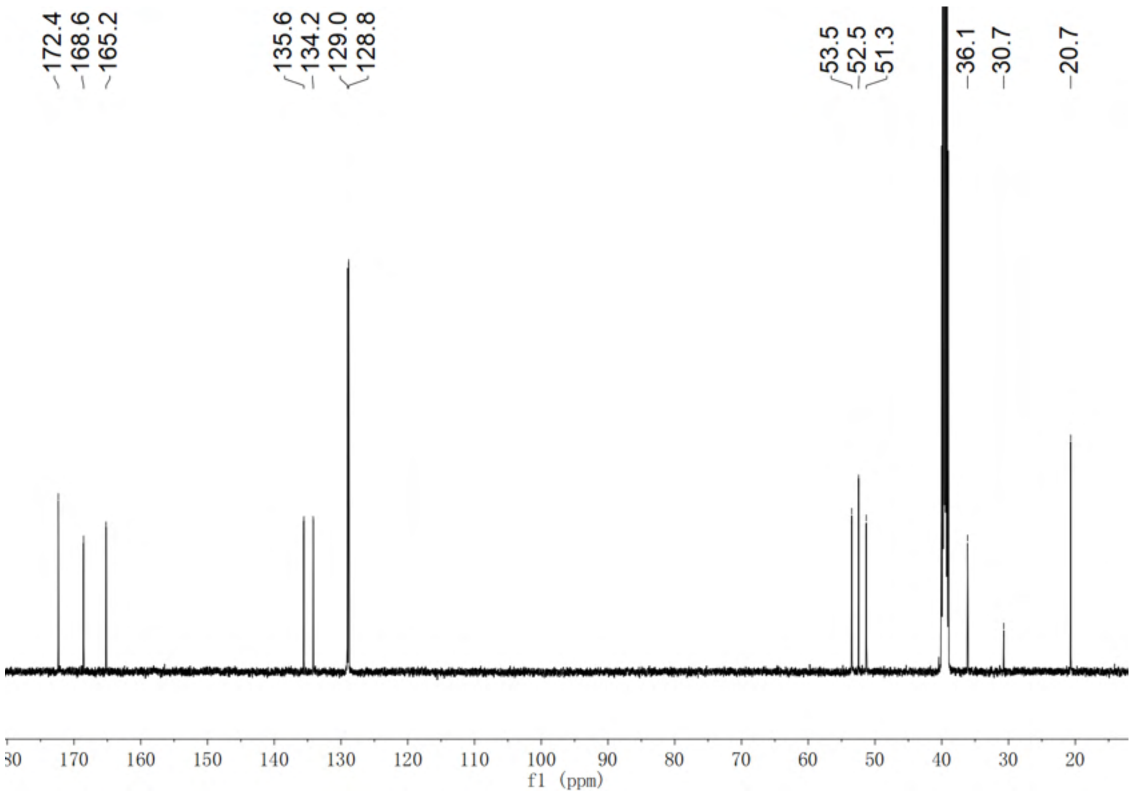

Supplementary Fig. 263. ^{13}C NMR spectrum of compound (2*S*,3*S*)-*t*-ES-a29 in DMSO-*d*$_6$

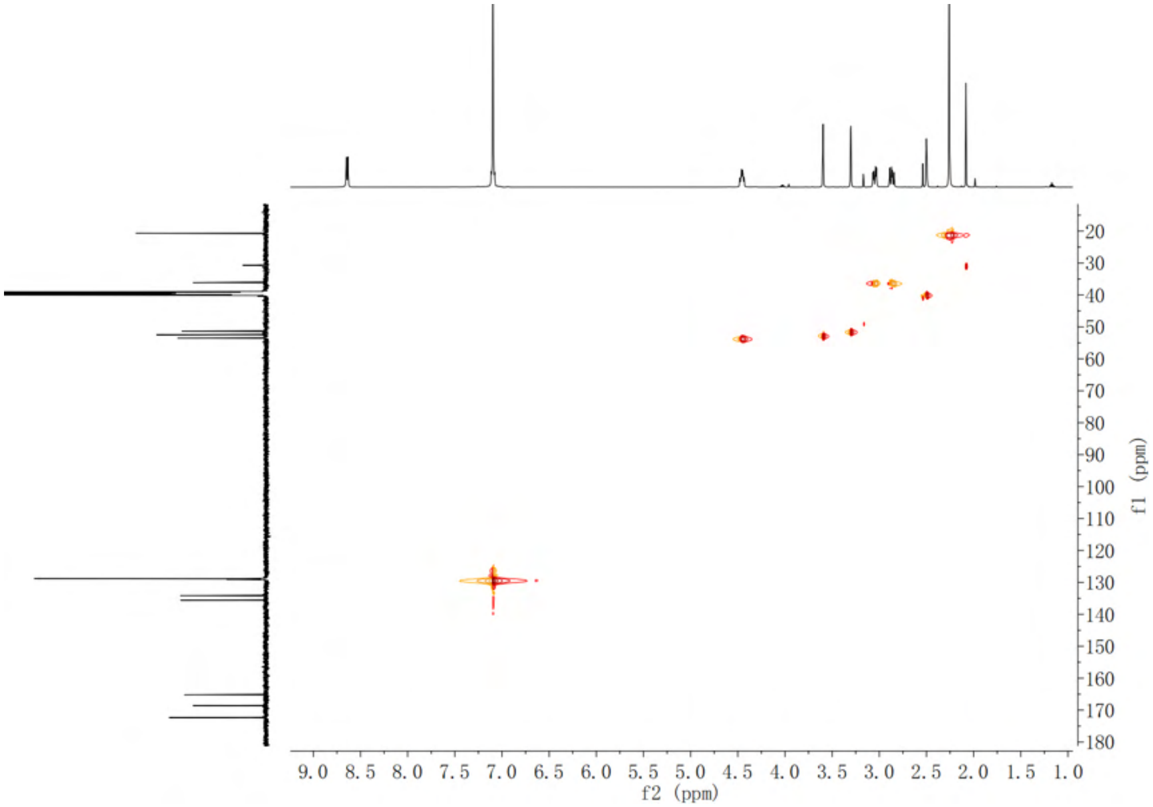

Supplementary Fig. 264. HSQC spectrum of compound (2*S*,3*S*)-*t*-ES-a29 in DMSO-*d*$_6$

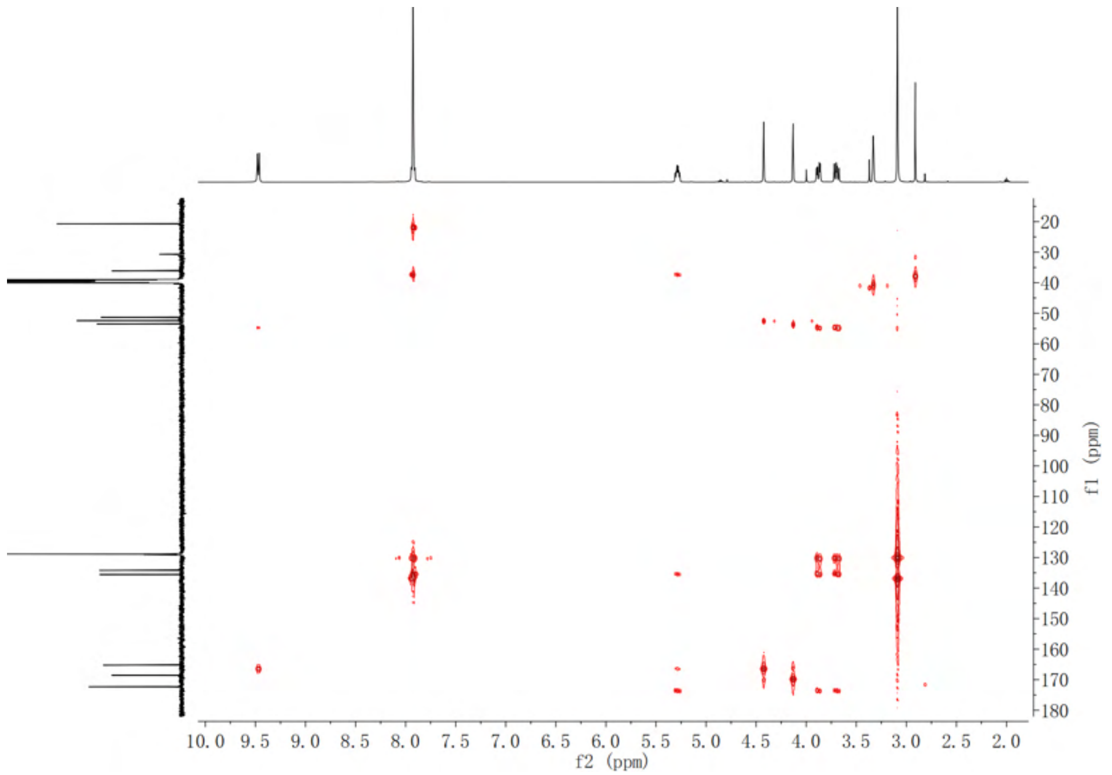

Supplementary Fig. 265. HMBC spectrum of compound (2*S*,3*S*)-*t*-ES-a29 in DMSO-*d*$_6$

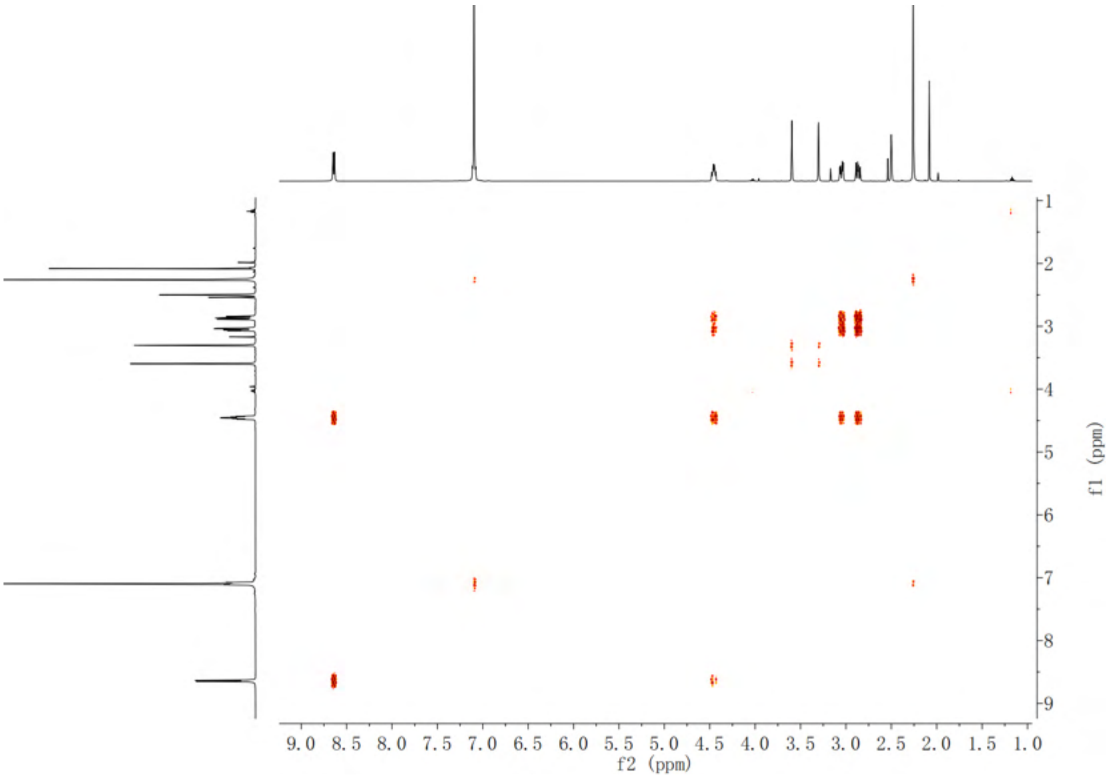

Supplementary Fig. 266. ^{1}H-^{1}H COSY spectrum of compound (2*S*,3*S*)-*t*-ES-a29 in DMSO-*d*$_6$

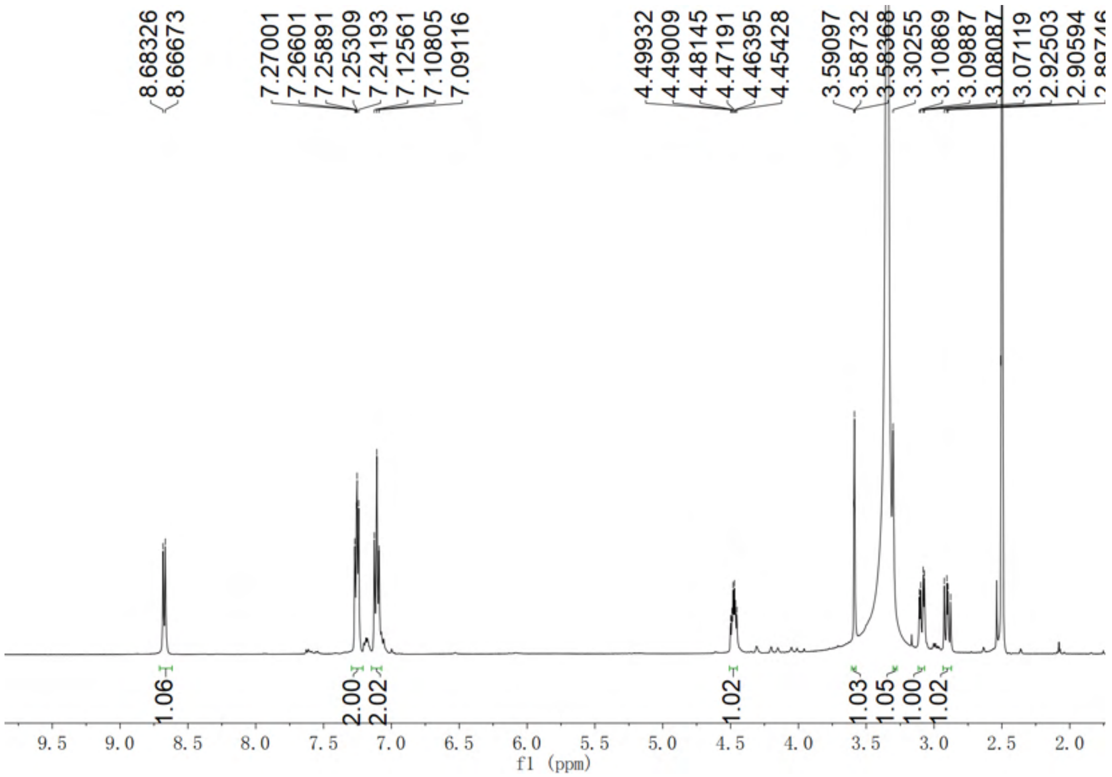

Supplementary Fig. 267. ^{1}H NMR spectrum of compound (2*S*,3*S*)-*t*-ES-a30 in DMSO-d_6

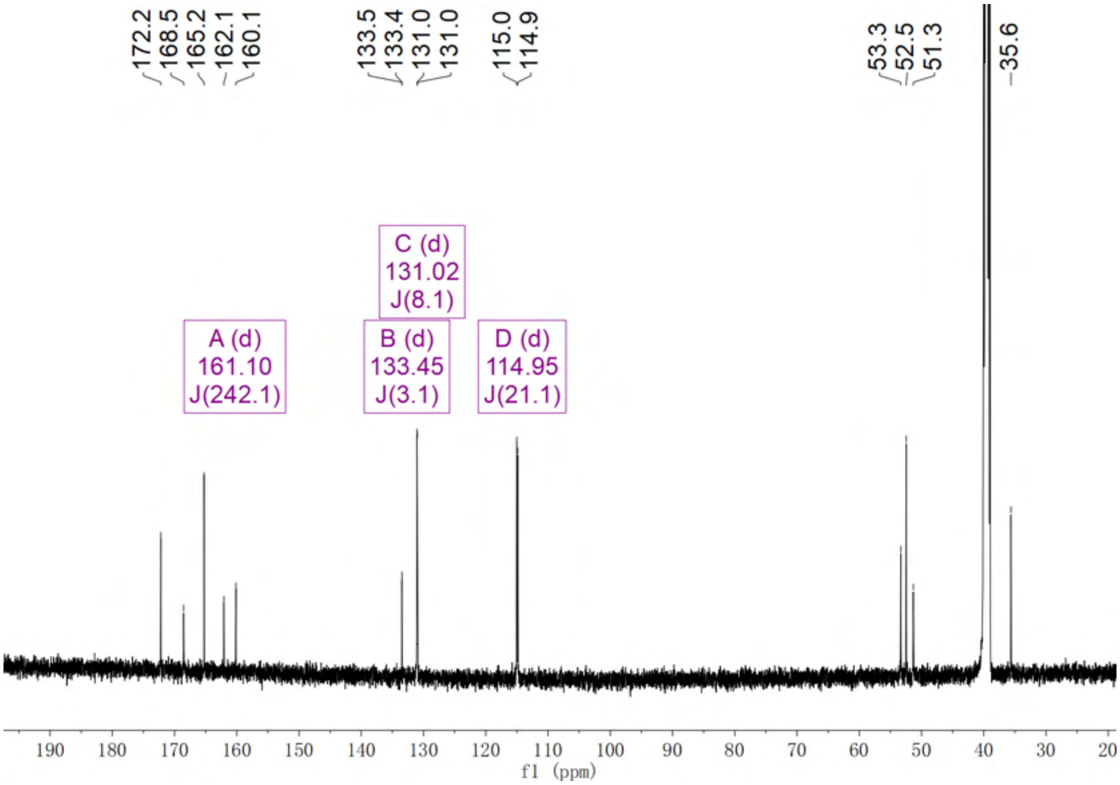

Supplementary Fig. 268. ^{13}C NMR spectrum of compound (2*S*,3*S*)-*t*-ES-a30 in DMSO-d_6

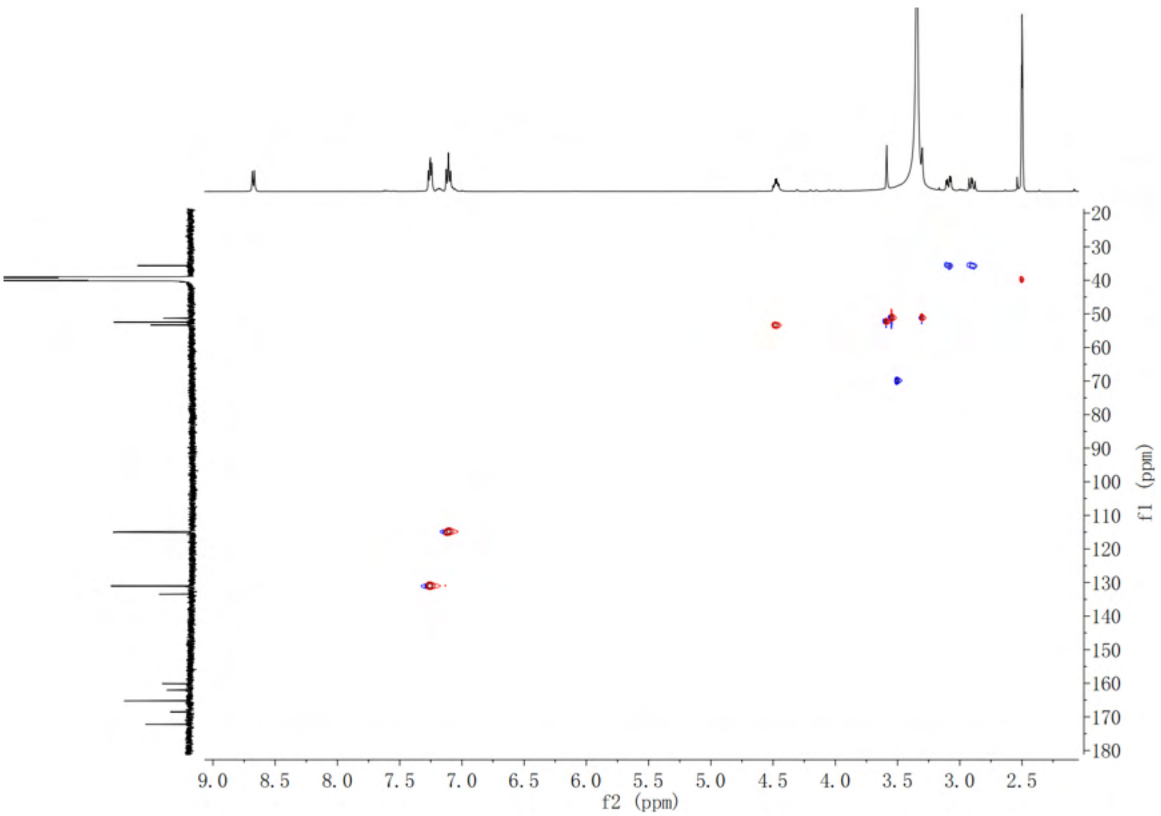

Supplementary Fig. 269. HSQC spectrum of compound (2*S*,3*S*)-*t*-ES-a30 in DMSO-d_6

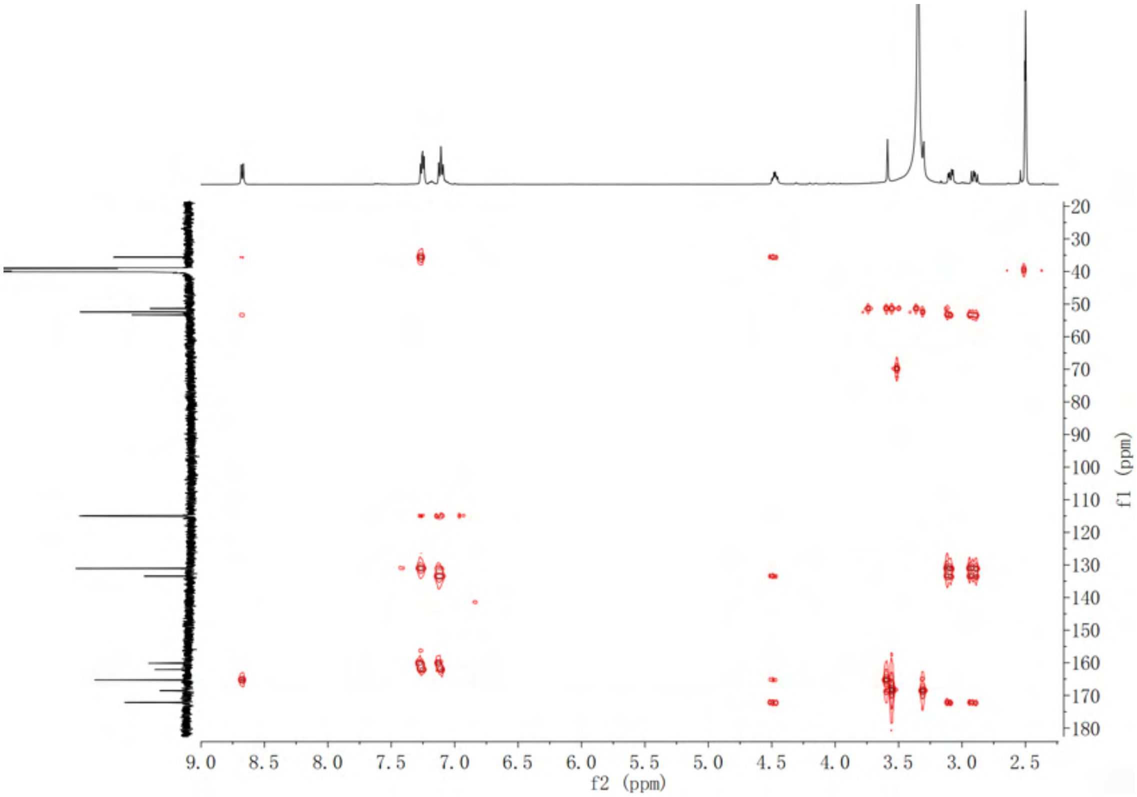

Supplementary Fig. 270. HMBC spectrum of compound (2*S*,3*S*)-*t*-ES-a30 in DMSO-d_6

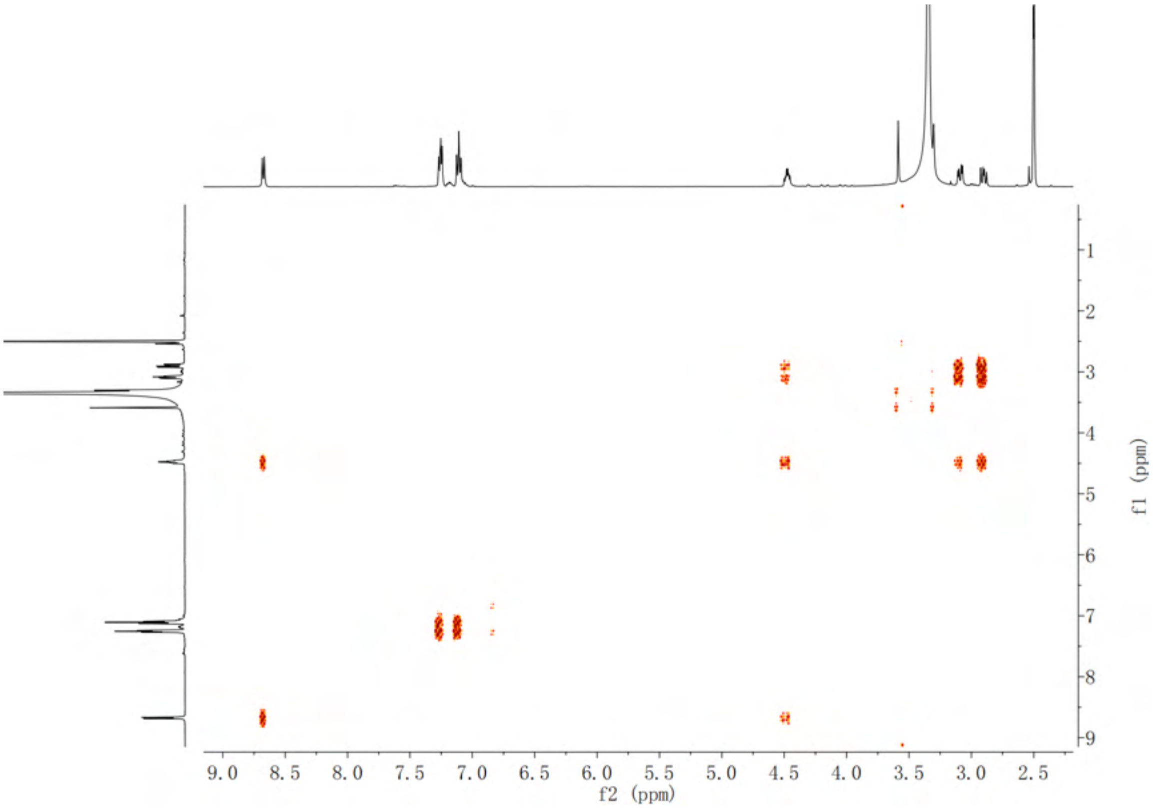

Supplementary Fig. 271. **^{1}H-^{1}H COSY spectrum of compound (2*S*,3*S*)-*t*-ES-a30 in DMSO-d_6**

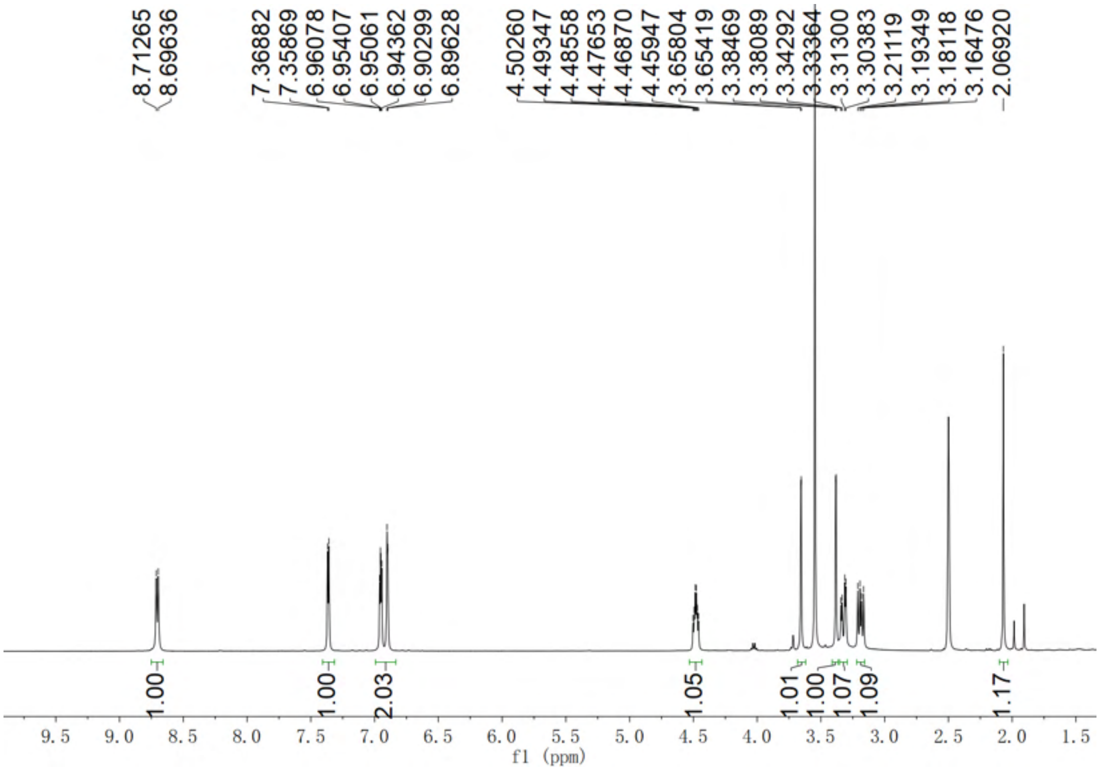

Supplementary Fig. 272. **^{1}H NMR spectrum of compound (2*S*,3*S*)-*t*-ES-a31 in DMSO-d_6**

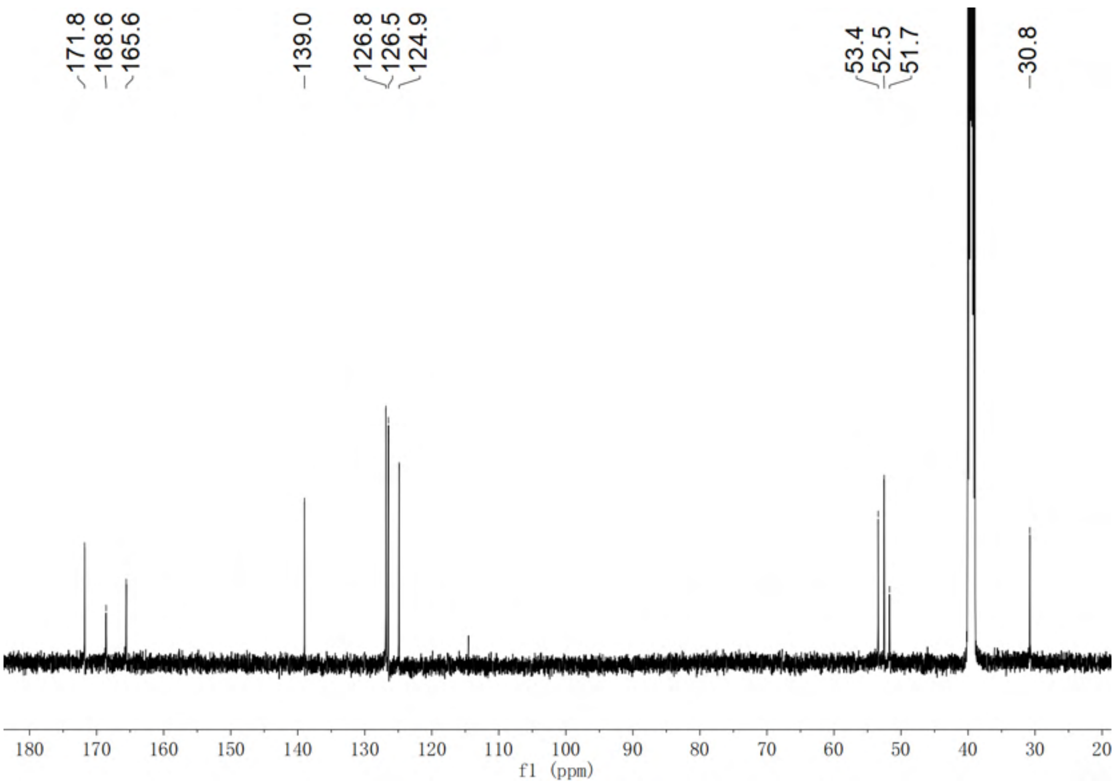

Supplementary Fig. 273. ^{13}**C NMR spectrum of compound (2*S*,3*S*)-*t*-ES-a31 in DMSO-*d*$_6$**

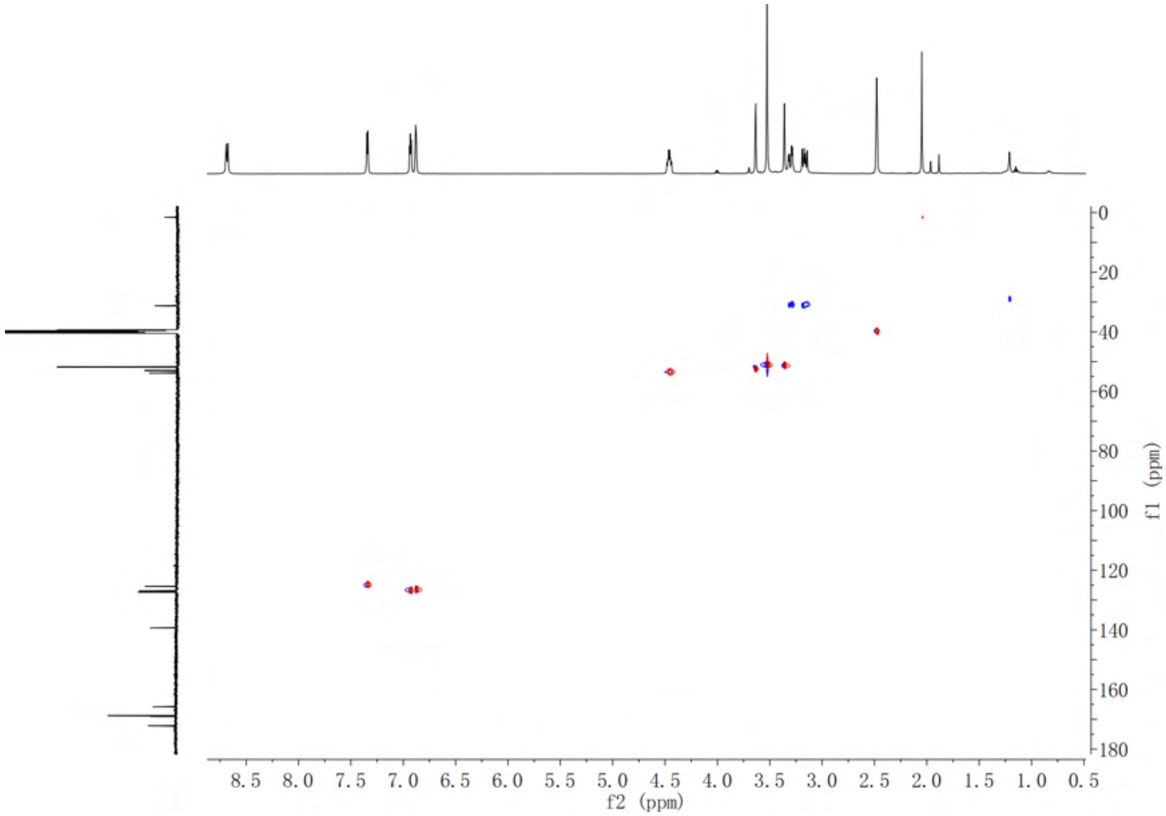

Supplementary Fig. 274. HSQC spectrum of compound (2*S*,3*S*)-*t*-ES-a31 in DMSO-*d*$_6$

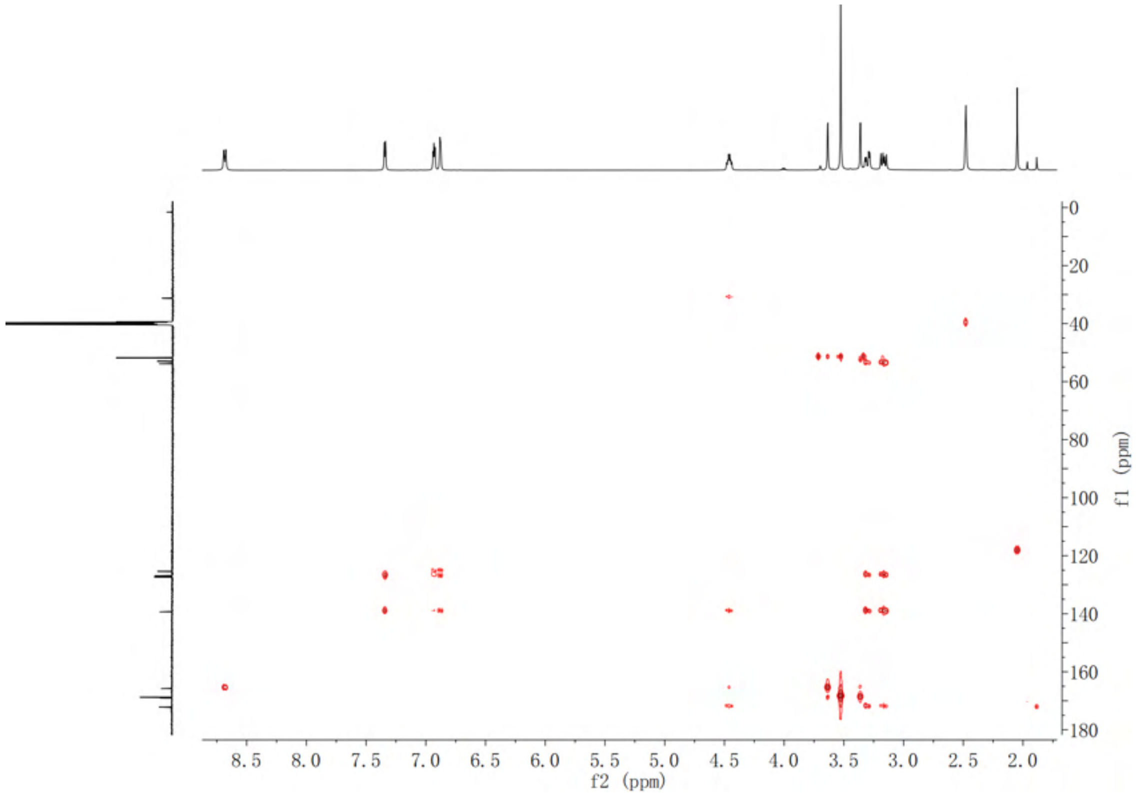

Supplementary Fig. 275. HMBC spectrum of compound (2*S*,3*S*)-*t*-ES-a31 in DMSO-d_6

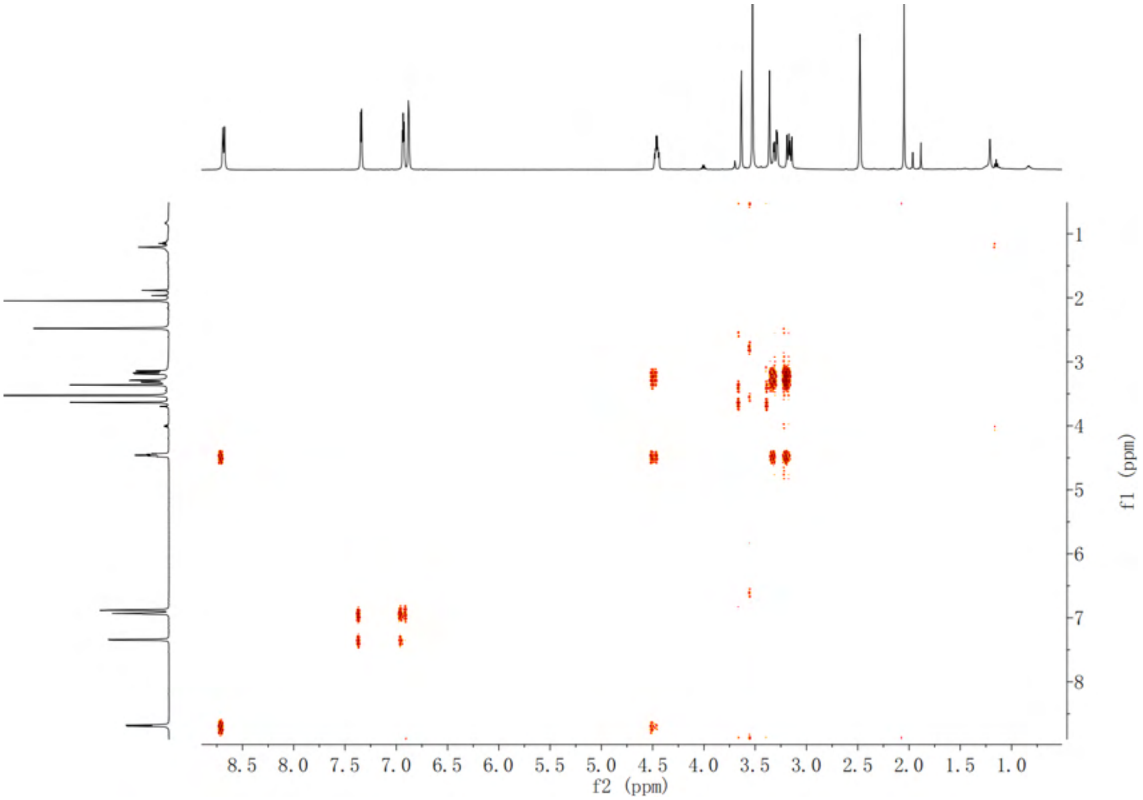

Supplementary Fig. 276. ^{1}H-^{1}H COSY spectrum of compound (2*S*,3*S*)-*t*-ES-a31 in DMSO-d_6

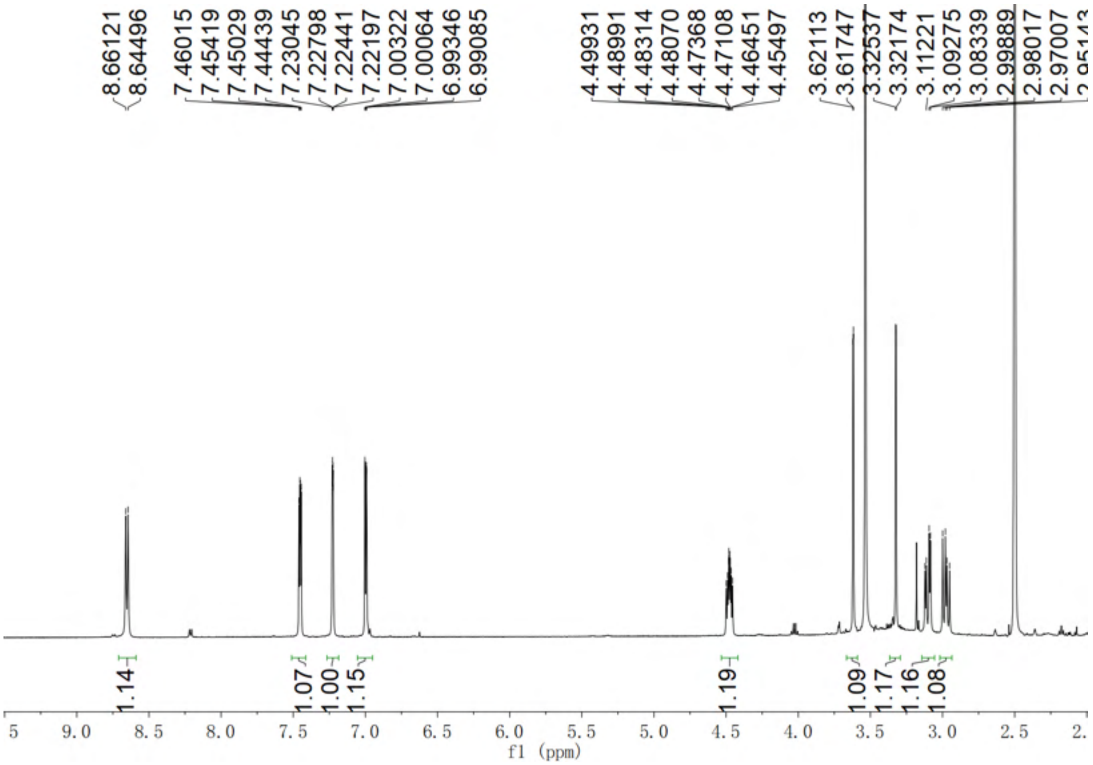

Supplementary Fig. 277. ^{1}H NMR spectrum of compound (2*S*,3*S*)-*t*-ES-a32 in DMSO-d_6

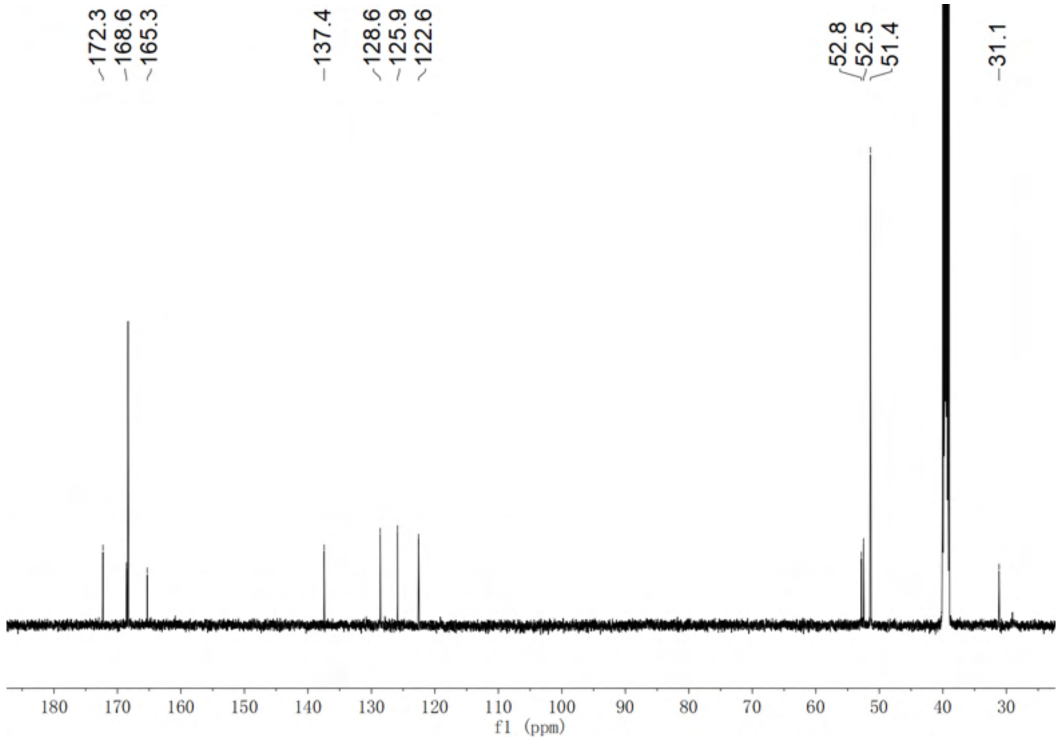

Supplementary Fig. 278. ^{13}C NMR spectrum of compound (2*S*,3*S*)-*t*-ES-a32 in DMSO-d_6

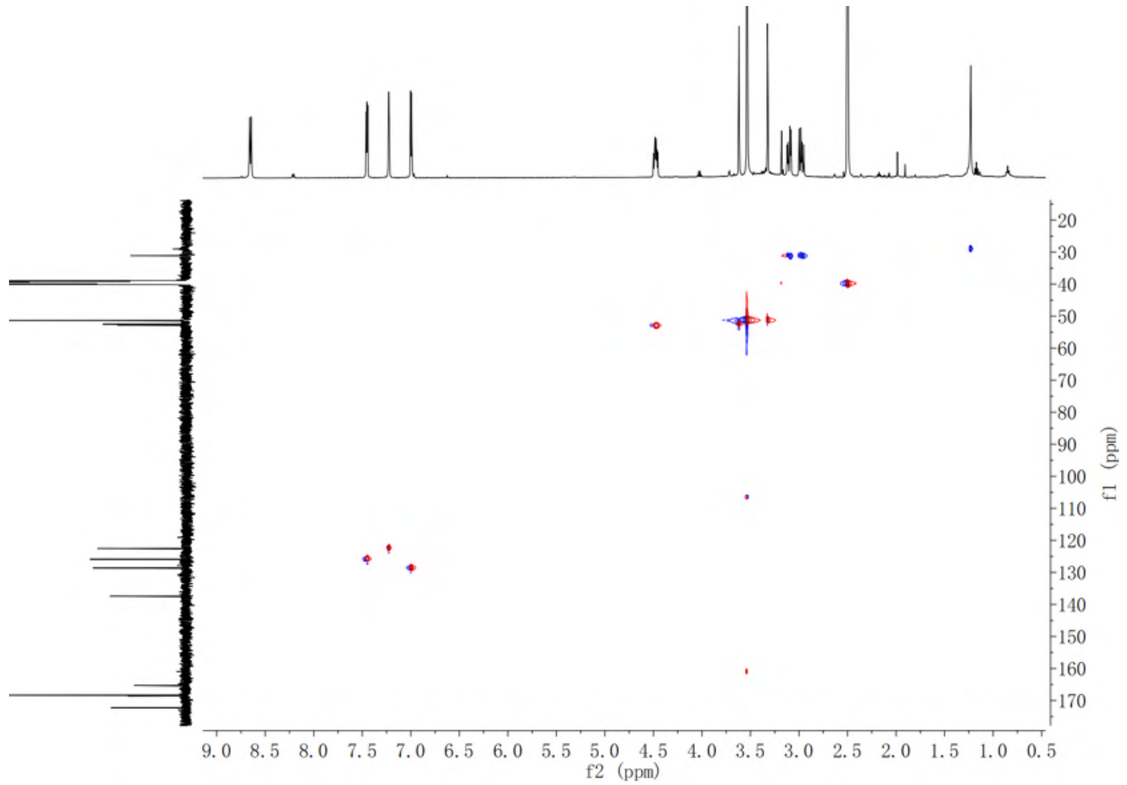

Supplementary Fig. 279. HSQC spectrum of compound (2*S*,3*S*)-*t*-ES-a32 in DMSO-d_6

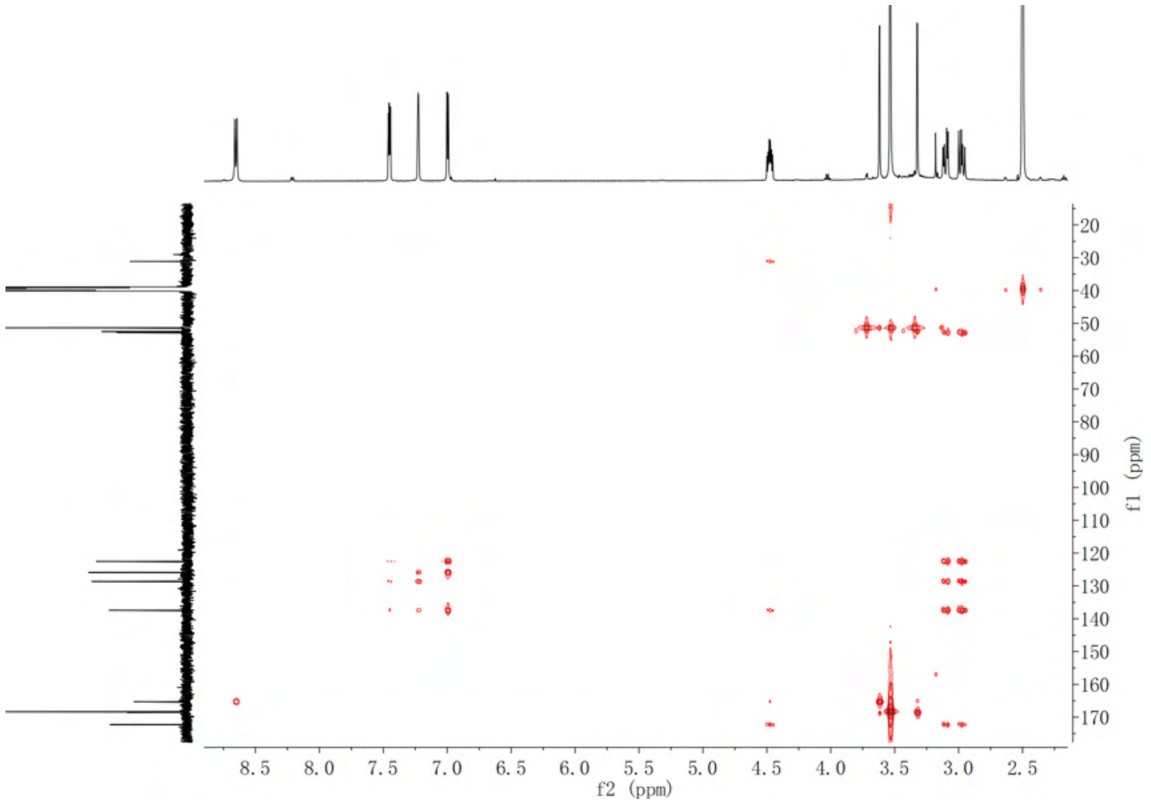

Supplementary Fig. 280. HMBC spectrum of compound (2*S*,3*S*)-*t*-ES-a32 in DMSO-d_6

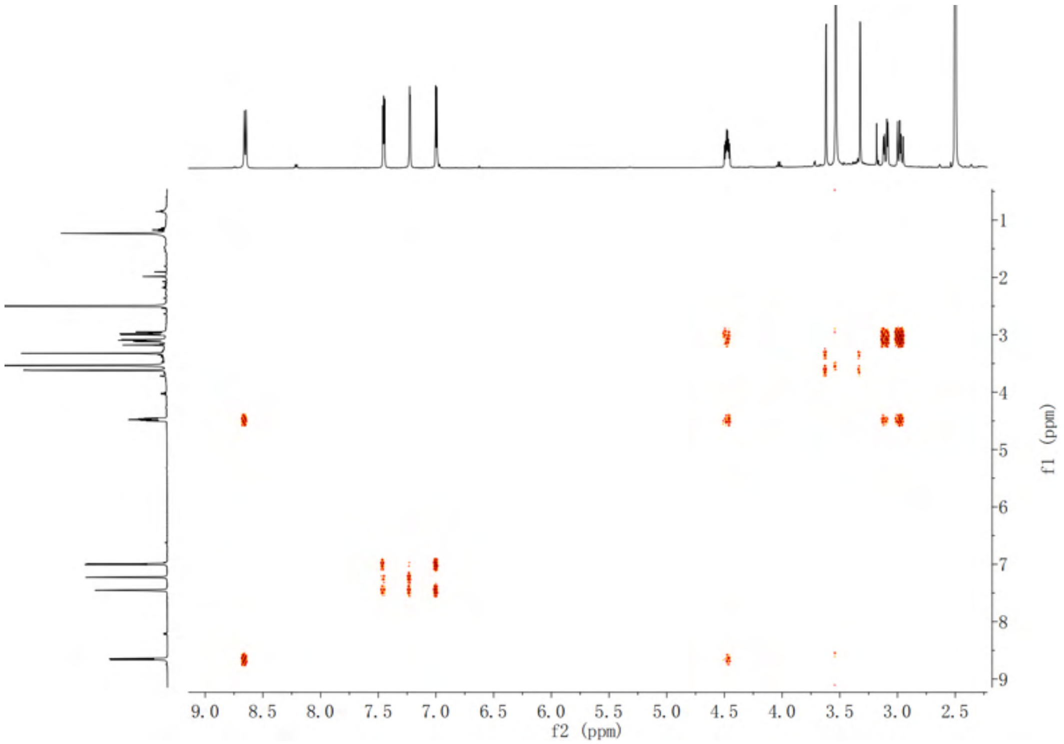

Supplementary Fig. 281. **^{1}H-^{1}H COSY spectrum of compound (2*S*,3*S*)-*t*-ES-a32 in DMSO-d_6**

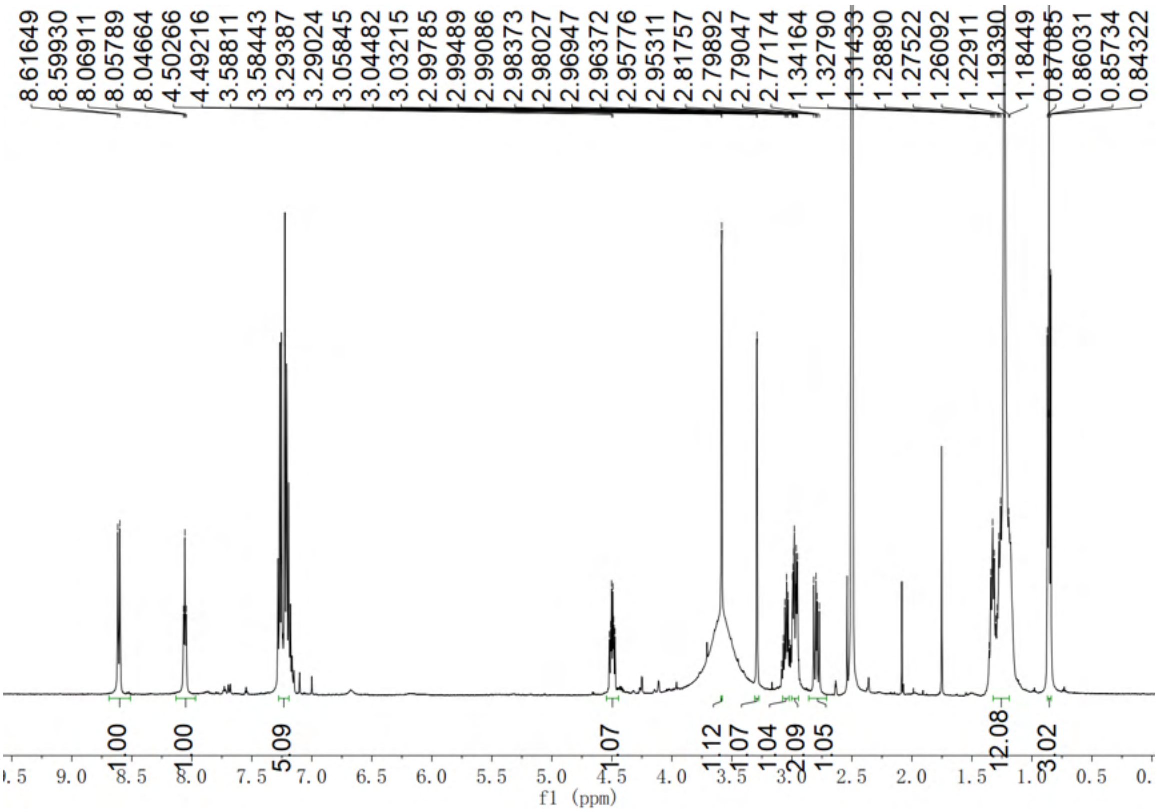

Supplementary Fig. 282. **^{1}H NMR spectrum of compound (2*S*,3*S*)-*t*-ES-Phe-b15 in DMSO-d_6**

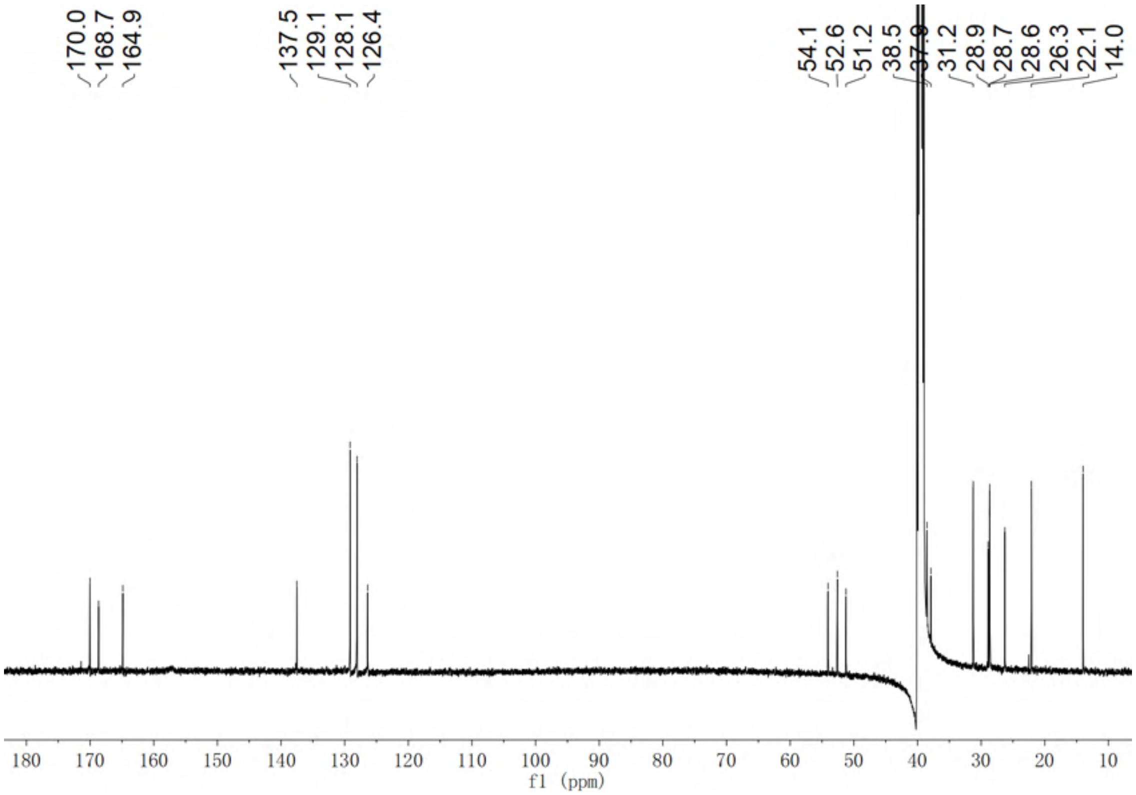

Supplementary Fig. 283. ^{13}C NMR spectrum of compound (2*S*,3*S*)-*t*-ES-Phe-b15 in DMSO-d_6

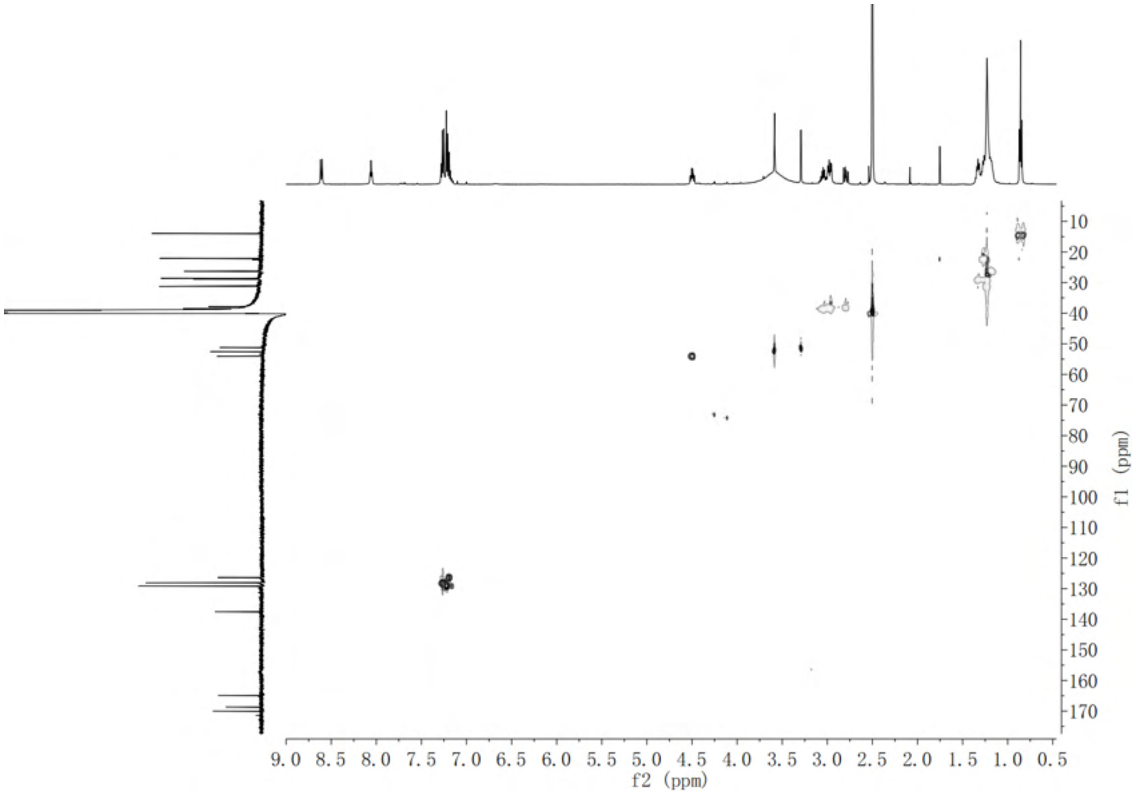

Supplementary Fig. 284. HSQC spectrum of compound (2*S*,3*S*)-*t*-ES-Phe-b15 in DMSO-d_6

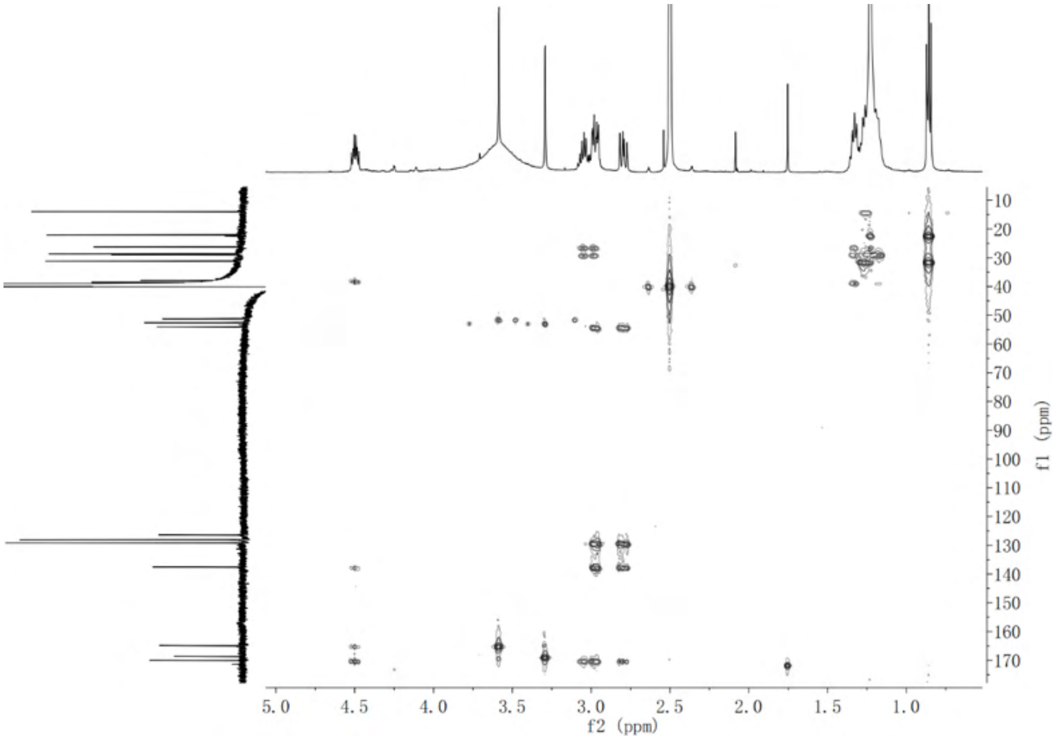

Supplementary Fig. 285. HMBC spectrum of compound (2*S*,3*S*)-*t*-ES-Phe-b15 in DMSO-d_6

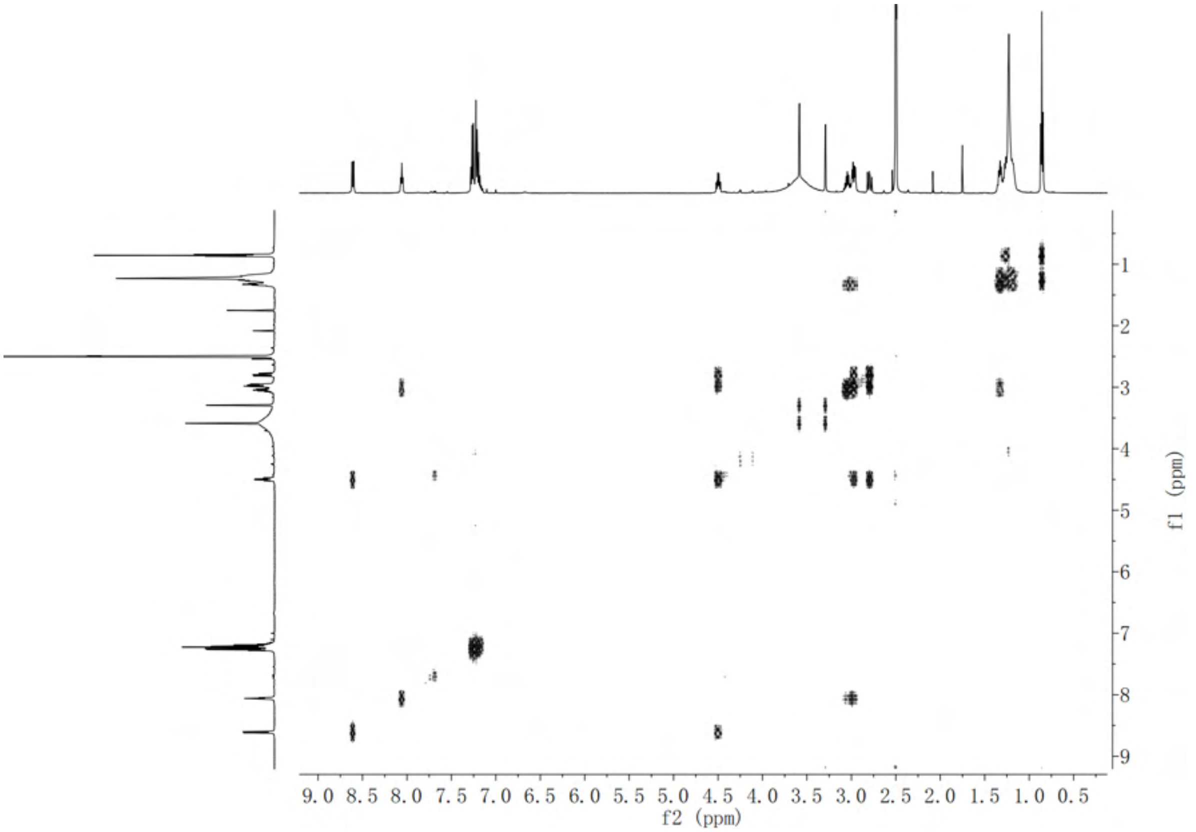

Supplementary Fig. 286. ^{1}H-^{1}H COSY spectrum of compound (2*S*,3*S*)-*t*-ES-Phe-b15 in DMSO-d_6

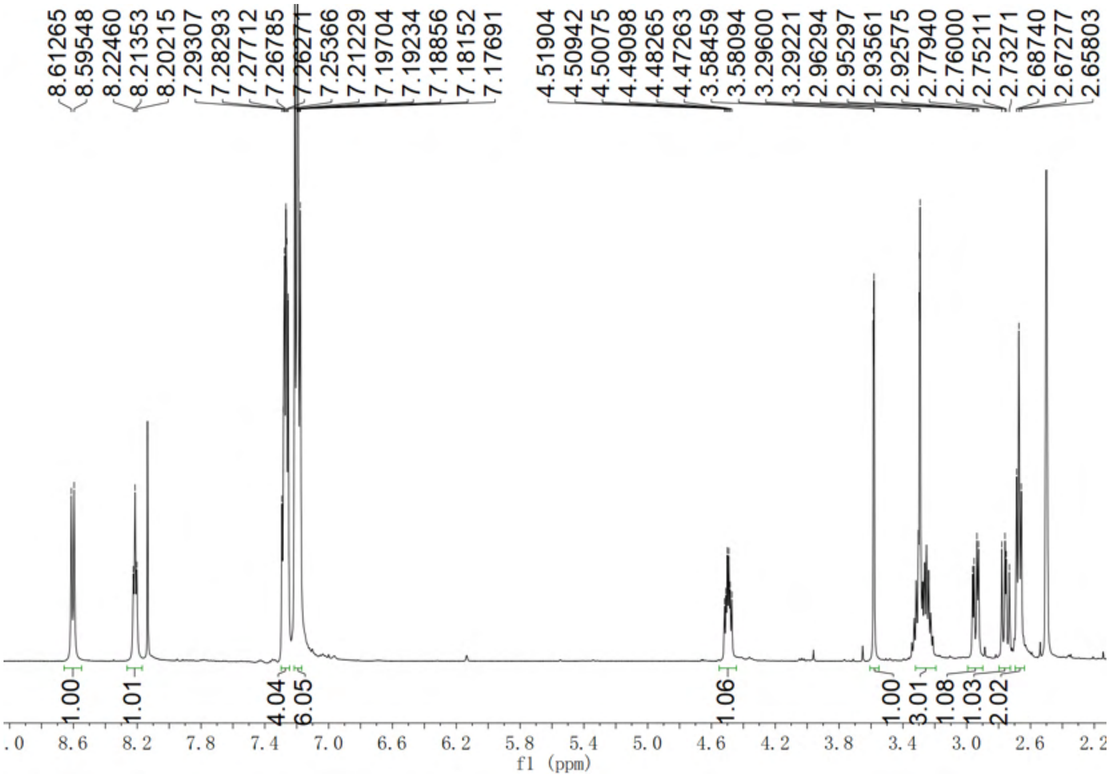

Supplementary Fig. 287. **^{1}H NMR spectrum of compound (2*S*,3*S*)-*t*-ES-Phe-b18 in DMSO-*d*$_6$**

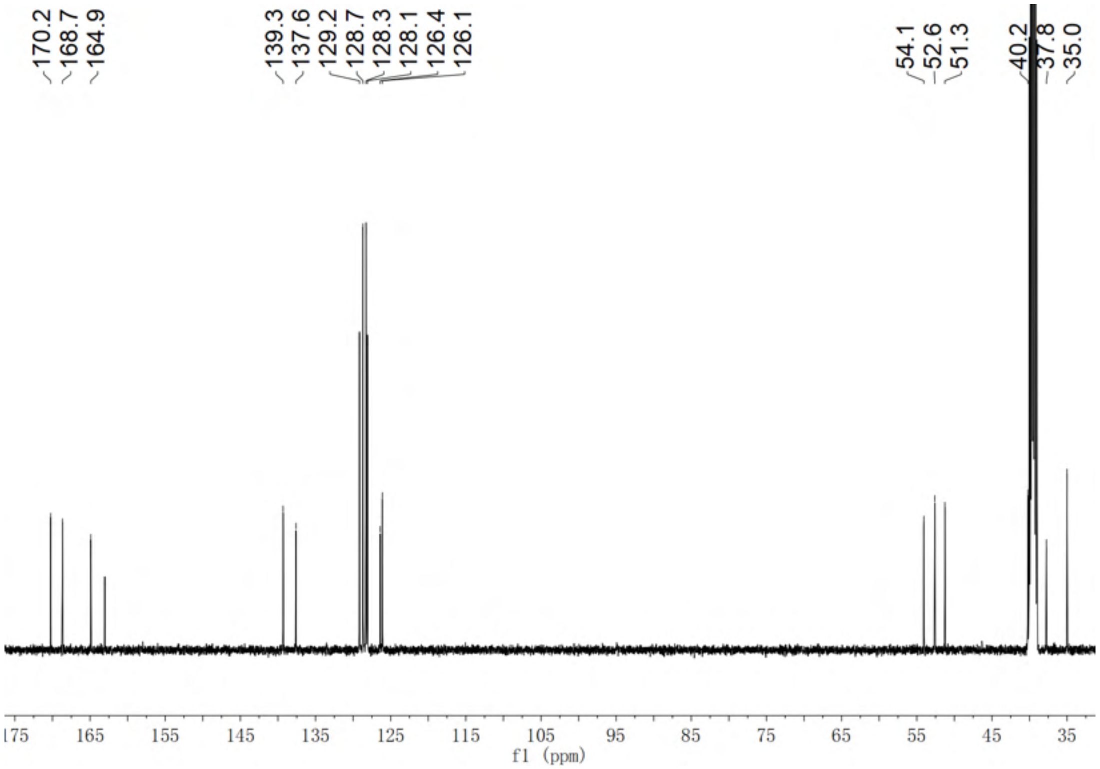

Supplementary Fig. 288. **^{13}C NMR spectrum of compound (2*S*,3*S*)-*t*-ES-Phe-b18 in DMSO-*d*$_6$**

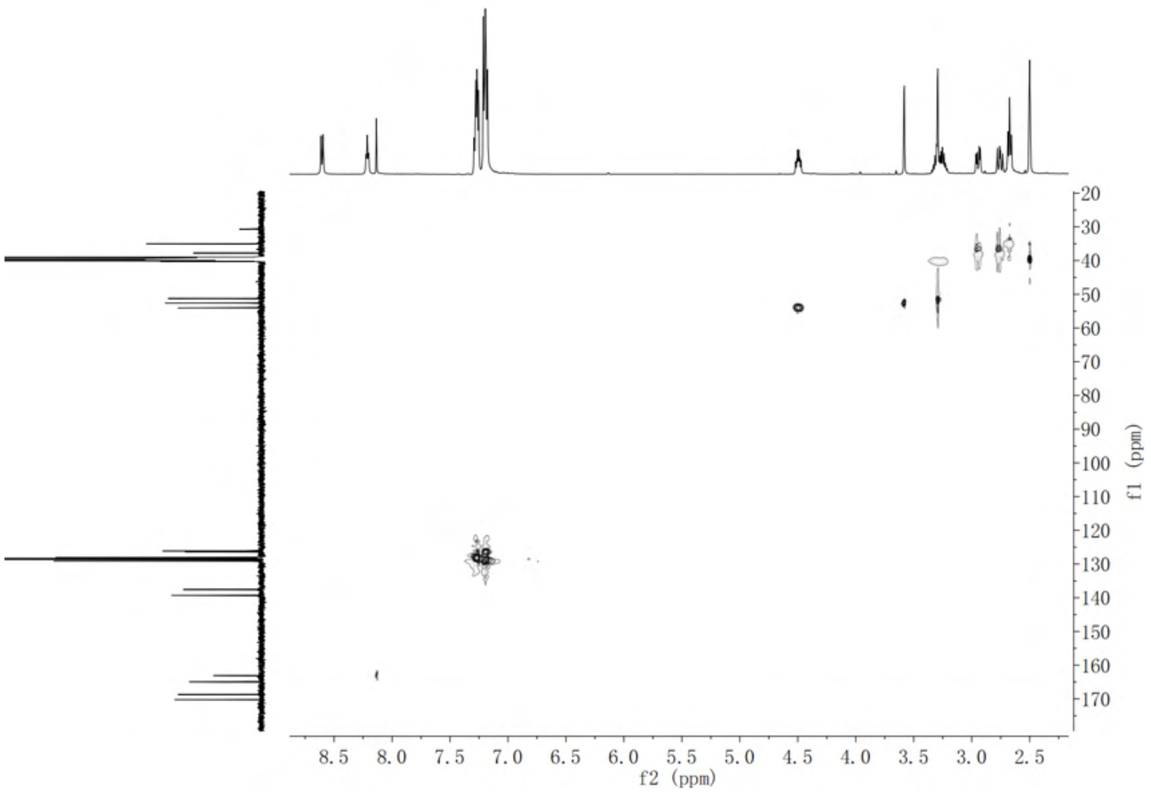

Supplementary Fig. 289. HSQC spectrum of compound (2*S*,3*S*)-*t*-ES-Phe-b18 in DMSO-d_6

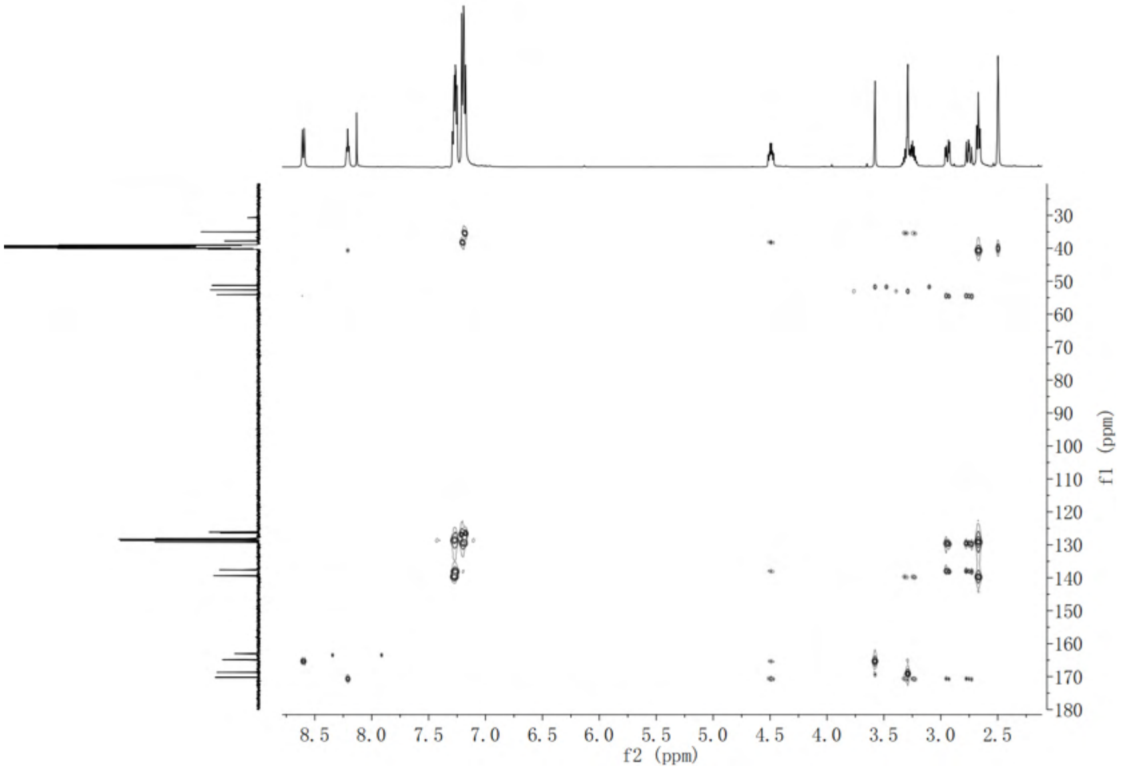

Supplementary Fig. 290. HMBC spectrum of compound (2*S*,3*S*)-*t*-ES-Phe-b18 in DMSO-d_6

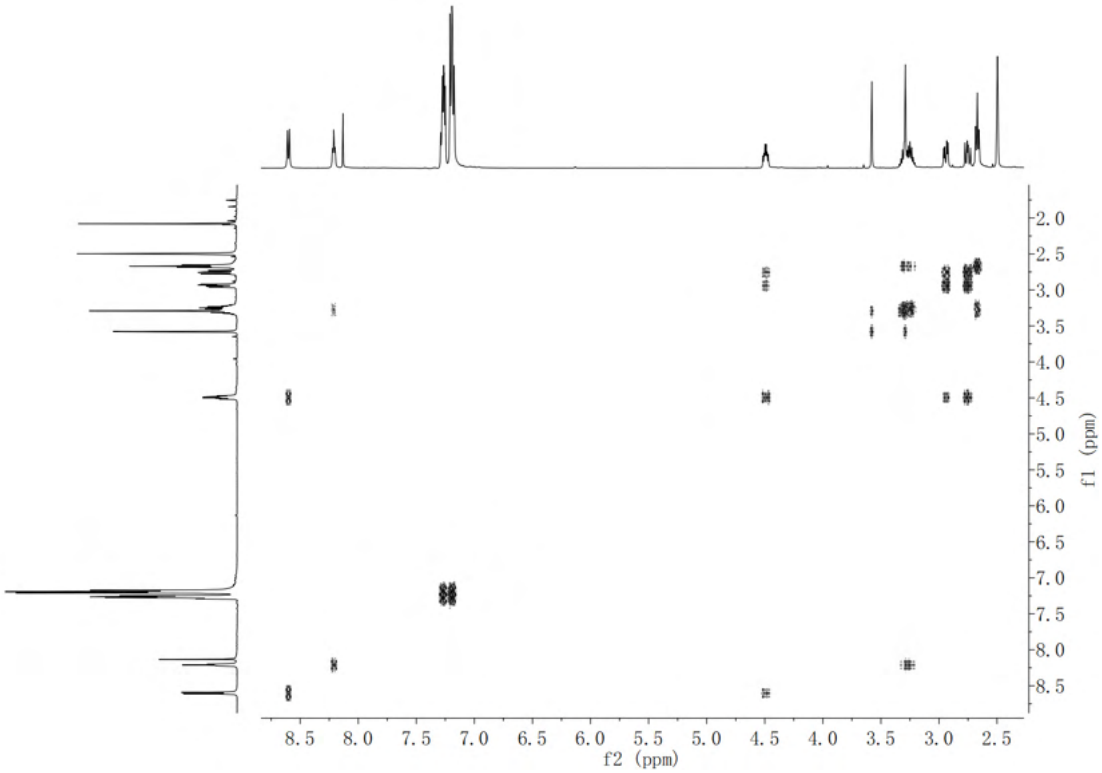

Supplementary Fig. 291. ^{1}H-^{1}H COSY spectrum of compound (2*S*,3*S*)-*t*-ES-Phe-b18 in DMSO-d_6

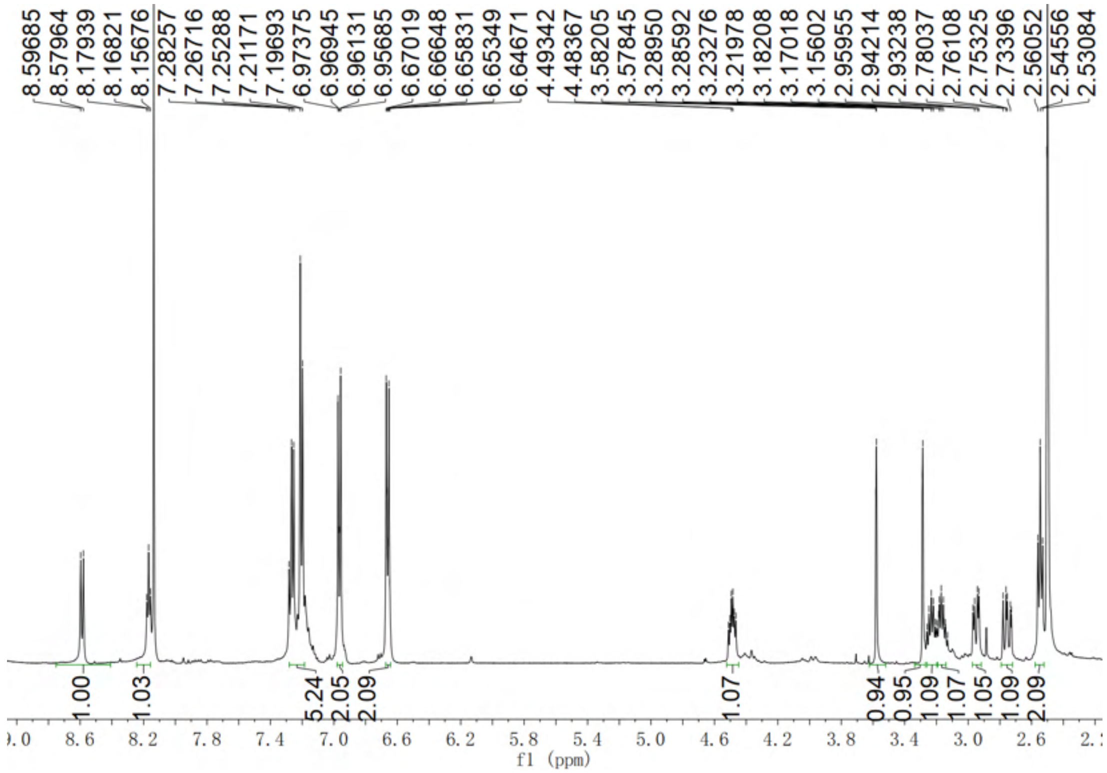

Supplementary Fig. 292. ^{1}H NMR spectrum of compound (2*S*,3*S*)-*t*-ES-Phe-b19 in DMSO-d_6

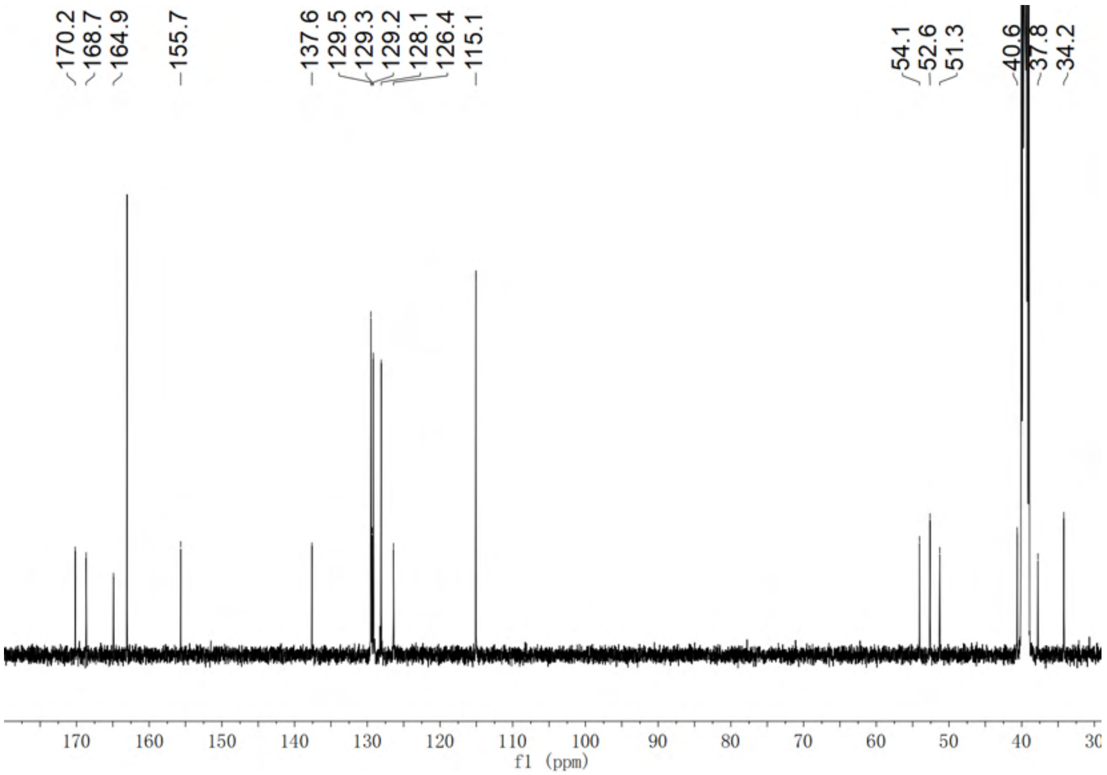

Supplementary Fig. 293. ^{13}**C NMR spectrum of compound (2*S*,3*S*)-*t*-ES-Phe-b19 in DMSO-*d*$_6$**

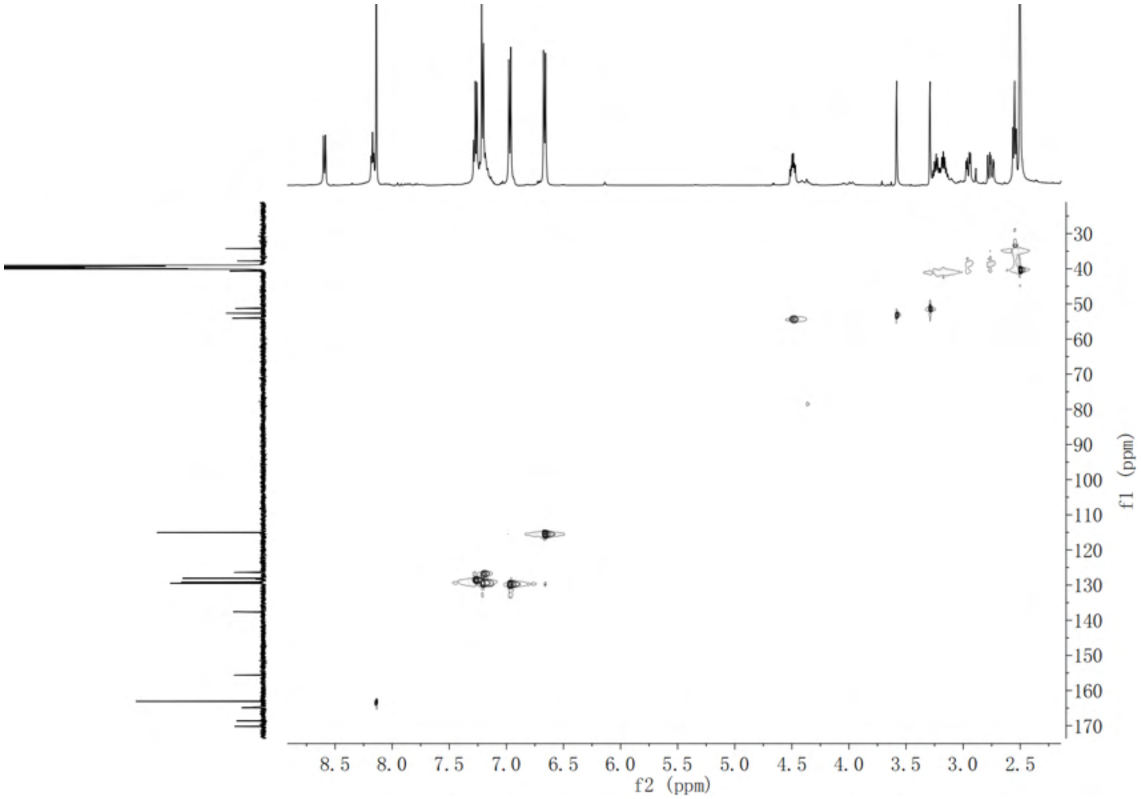

Supplementary Fig. 294. HSQC spectrum of compound (2*S*,3*S*)-*t*-ES-Phe-b19 in DMSO-*d*$_6$

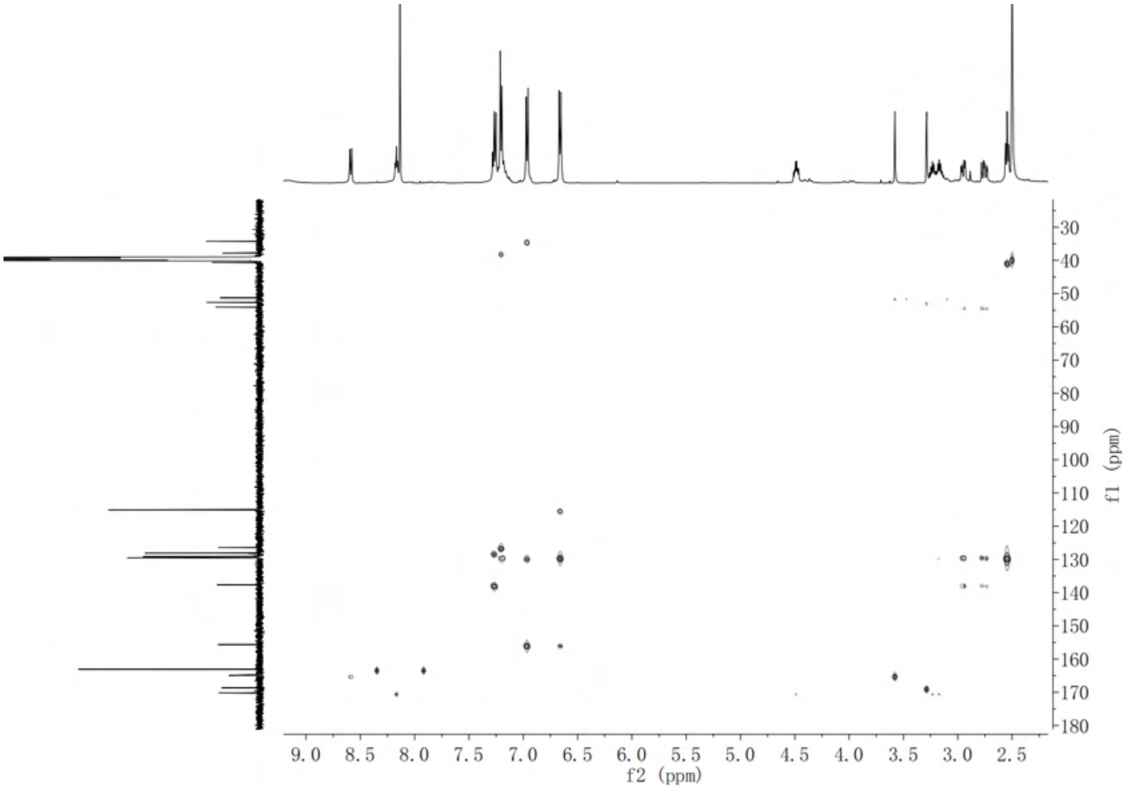

Supplementary Fig. 295. HMBC spectrum of compound (2*S*,3*S*)-*t*-ES-Phe-b19 in DMSO-d_6

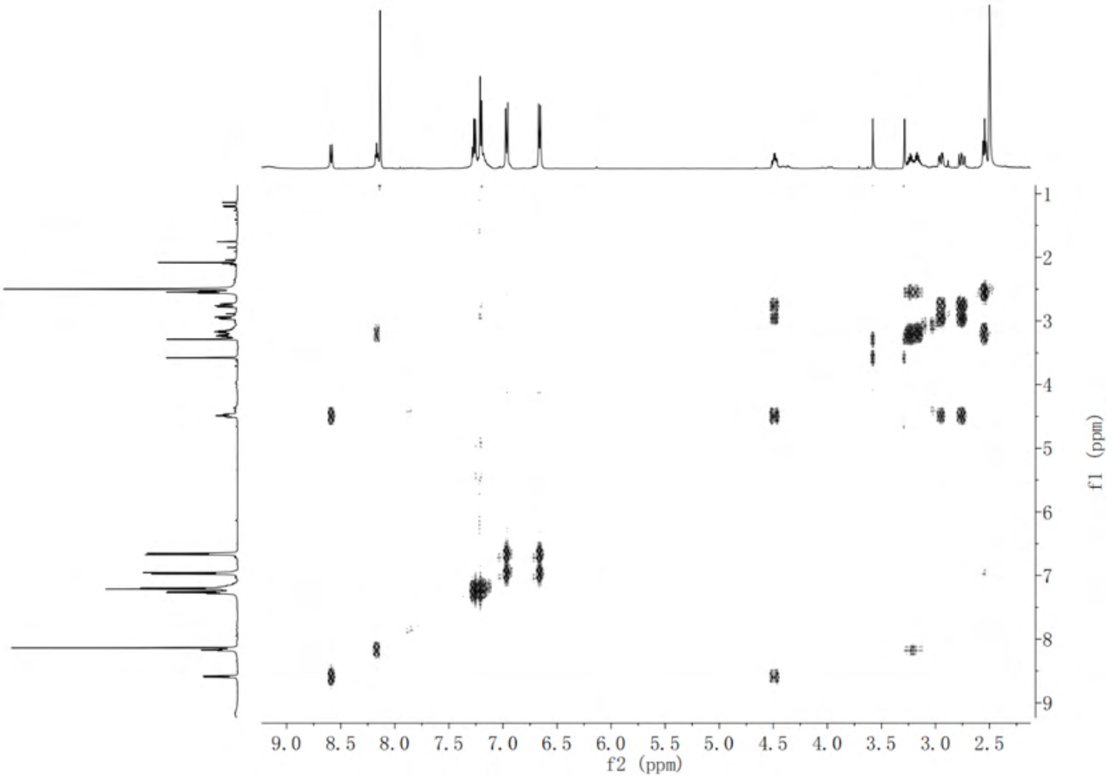

Supplementary Fig. 296. ^{1}H-^{1}H COSY spectrum of compound (2*S*,3*S*)-*t*-ES-Phe-b19 in DMSO-d_6

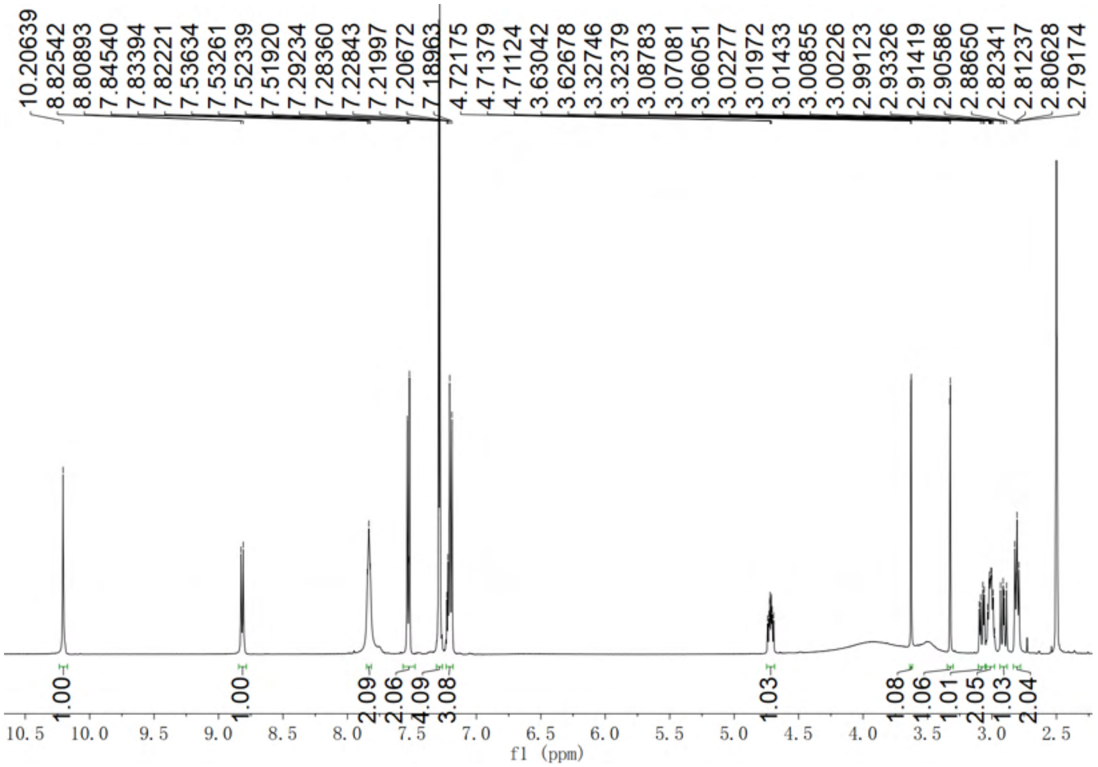

Supplementary Fig. 297. **^{1}H NMR spectrum of compound (2*S*,3*S*)-*t*-ES-Phe-b20 in DMSO-*d*$_6$**

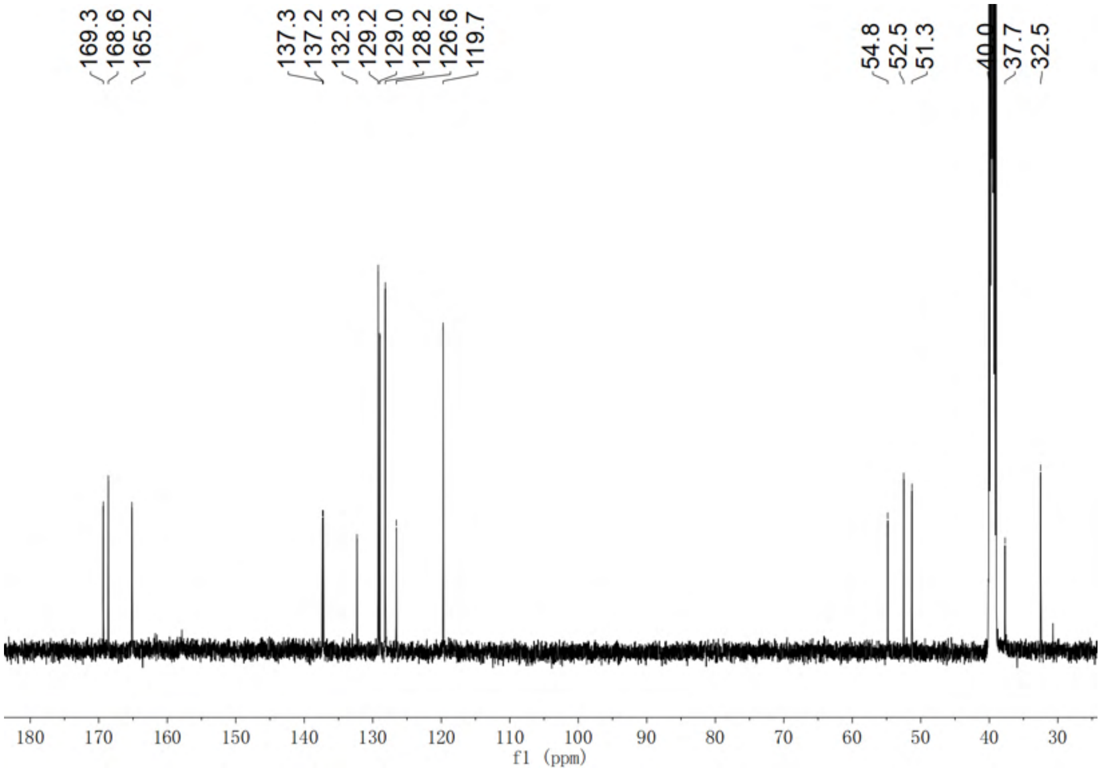

Supplementary Fig. 298. **^{13}C NMR spectrum of compound (2*S*,3*S*)-*t*-ES-Phe-b20 in DMSO-*d*$_6$**

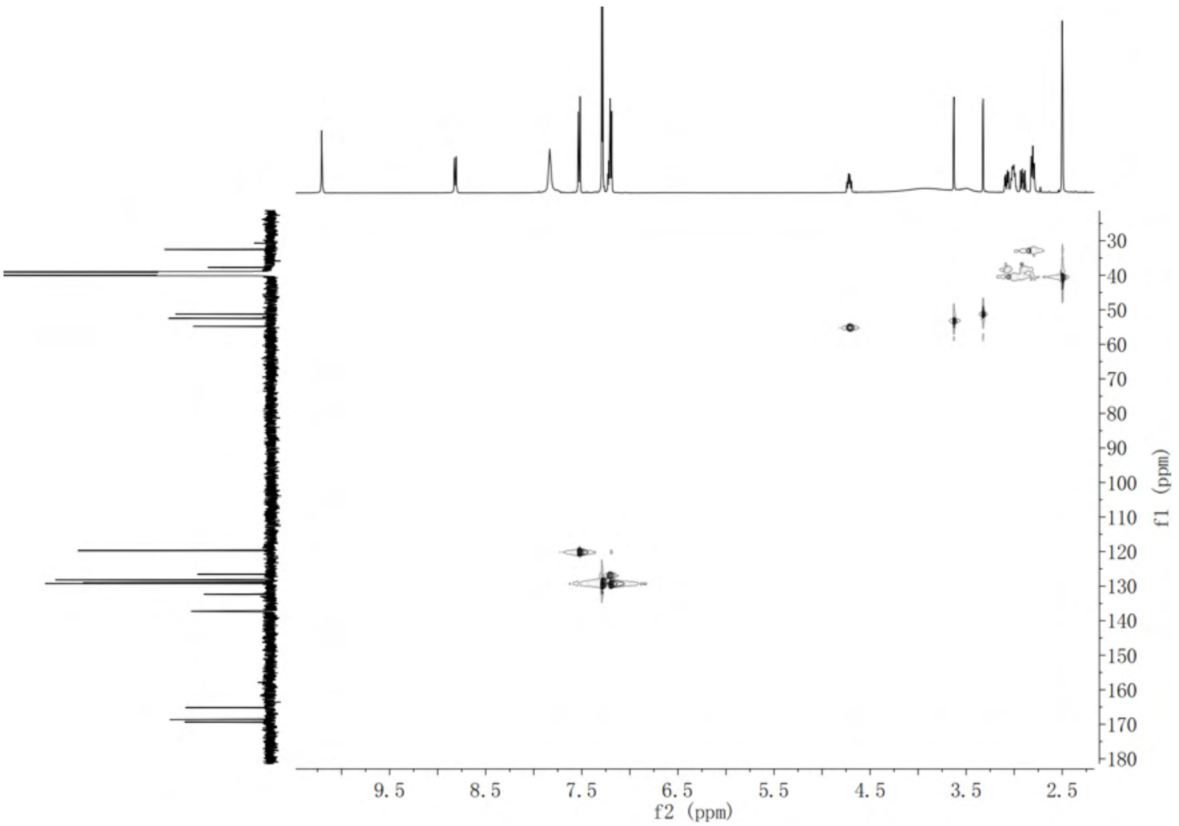

Supplementary Fig. 299. HSQC spectrum of compound (2*S*,3*S*)-*t*-ES-Phe-b20 in DMSO-d_6

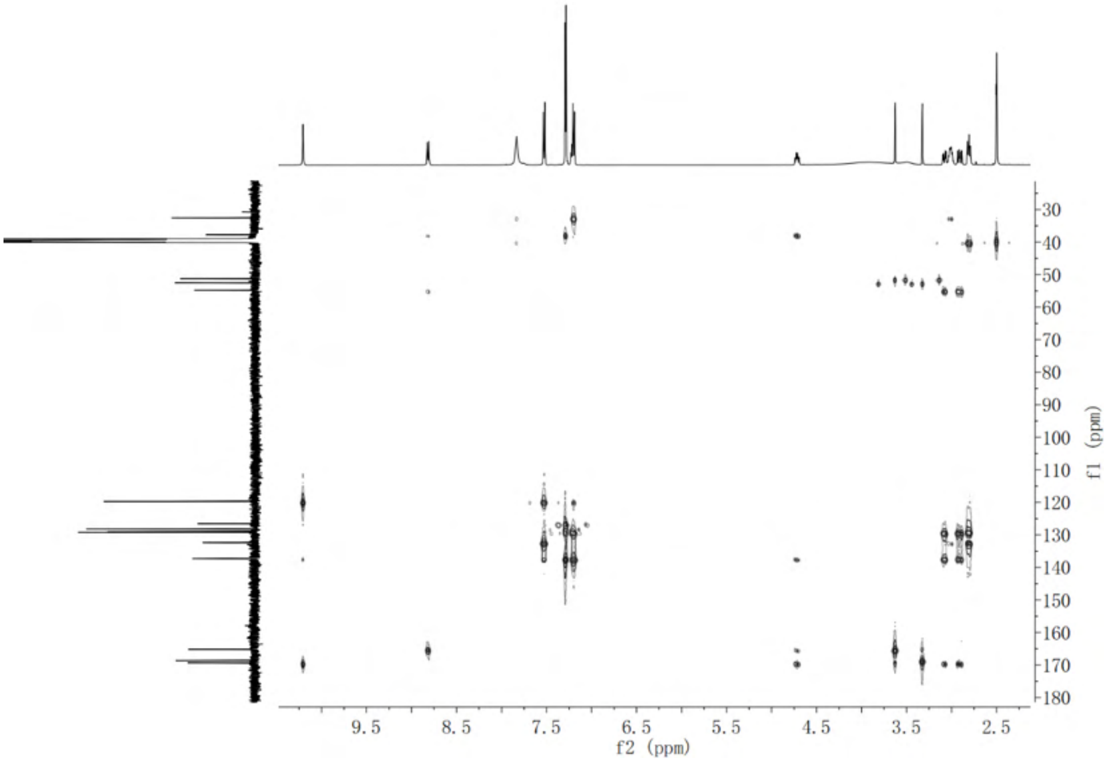

Supplementary Fig. 300. HMBC spectrum of compound (2*S*,3*S*)-*t*-ES-Phe-b20 in DMSO-d_6

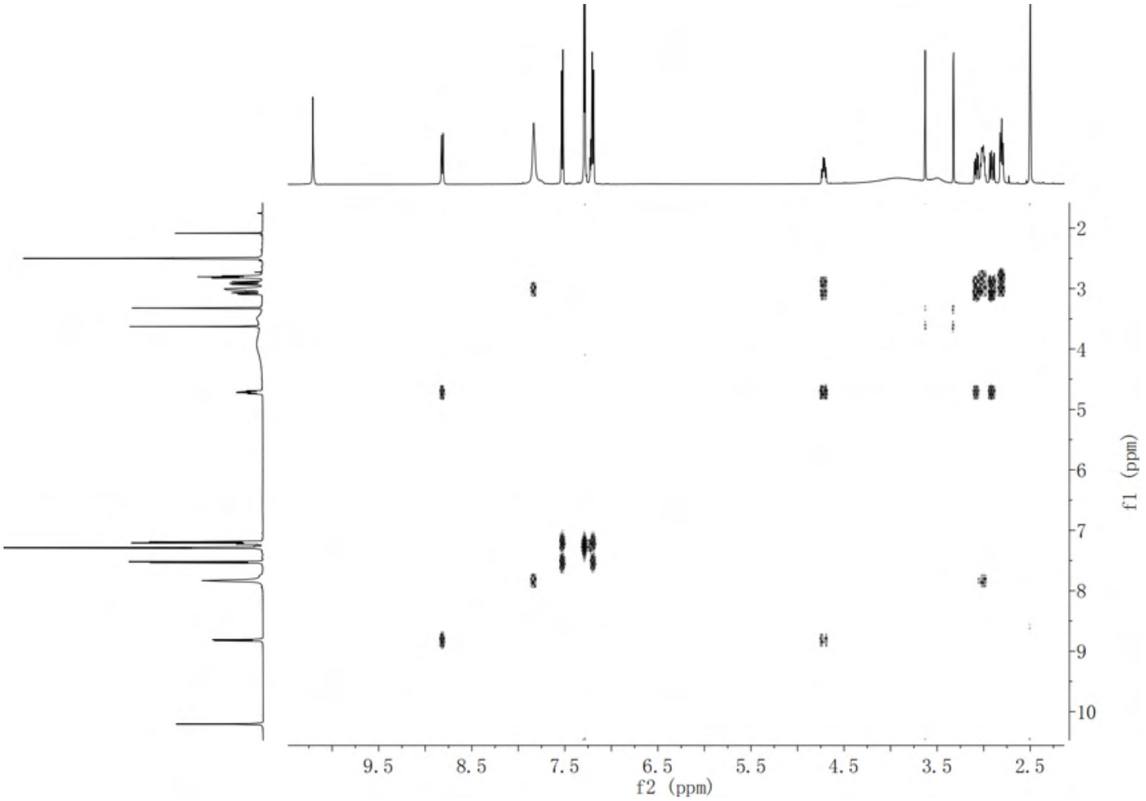

Supplementary Fig. 301. ^{1}H-^{1}H COSY spectrum of compound (2*S*,3*S*)-*t*-ES-Phe-b20 in DMSO-d_6

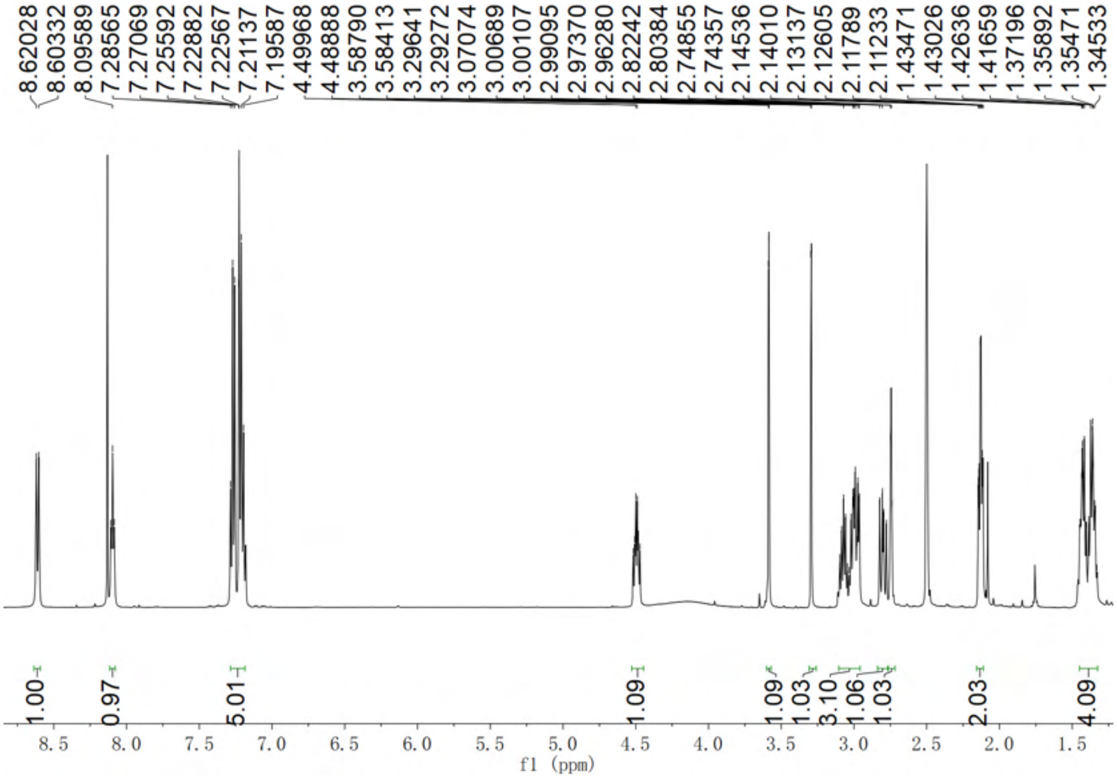

Supplementary Fig. 302. ^{1}H NMR spectrum of compound (2*S*,3*S*)-*t*-ES-Phe-b24 in DMSO-d_6

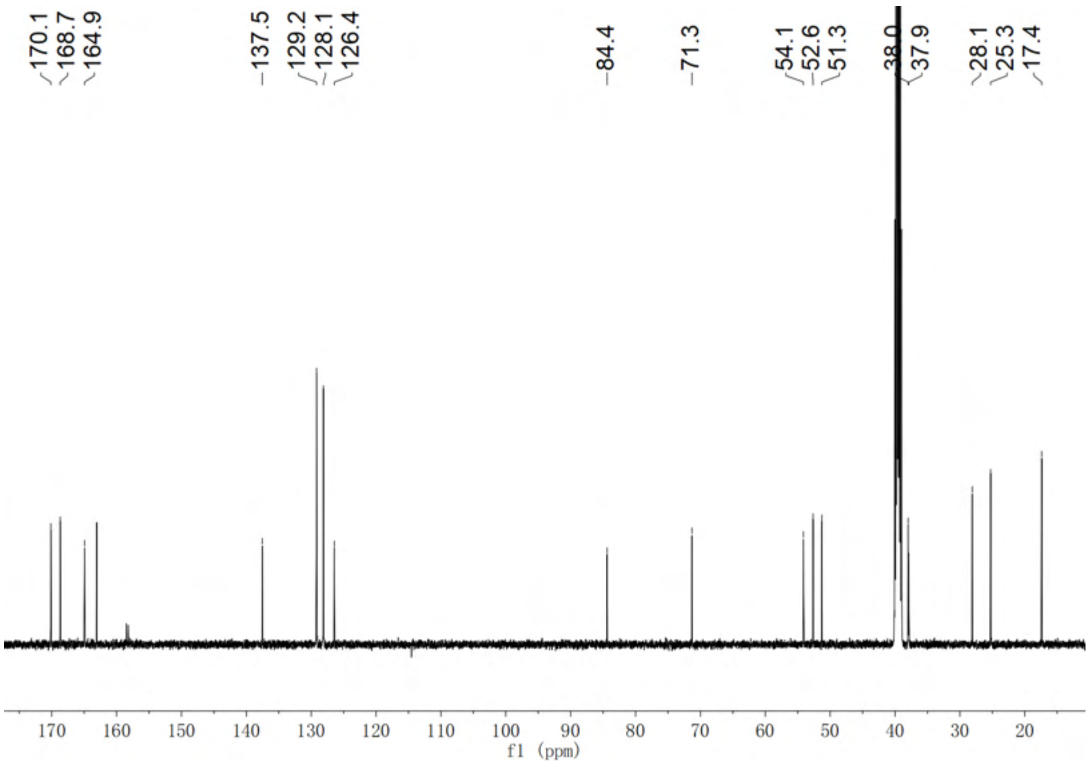

Supplementary Fig. 303. ^{13}**C NMR spectrum of compound (2*S*,3*S*)-*t*-ES-Phe-b24 in DMSO-*d*$_6$**

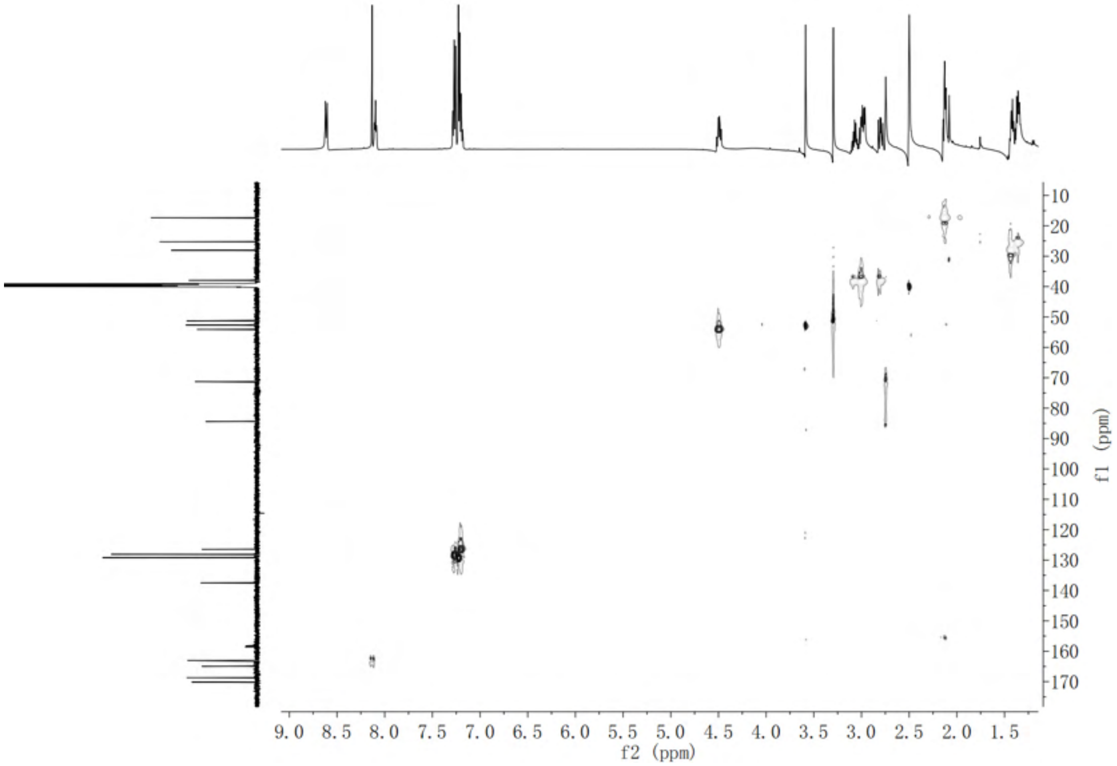

Supplementary Fig. 304. HSQC spectrum of compound (2*S*,3*S*)-*t*-ES-Phe-b24 in DMSO-*d*$_6$

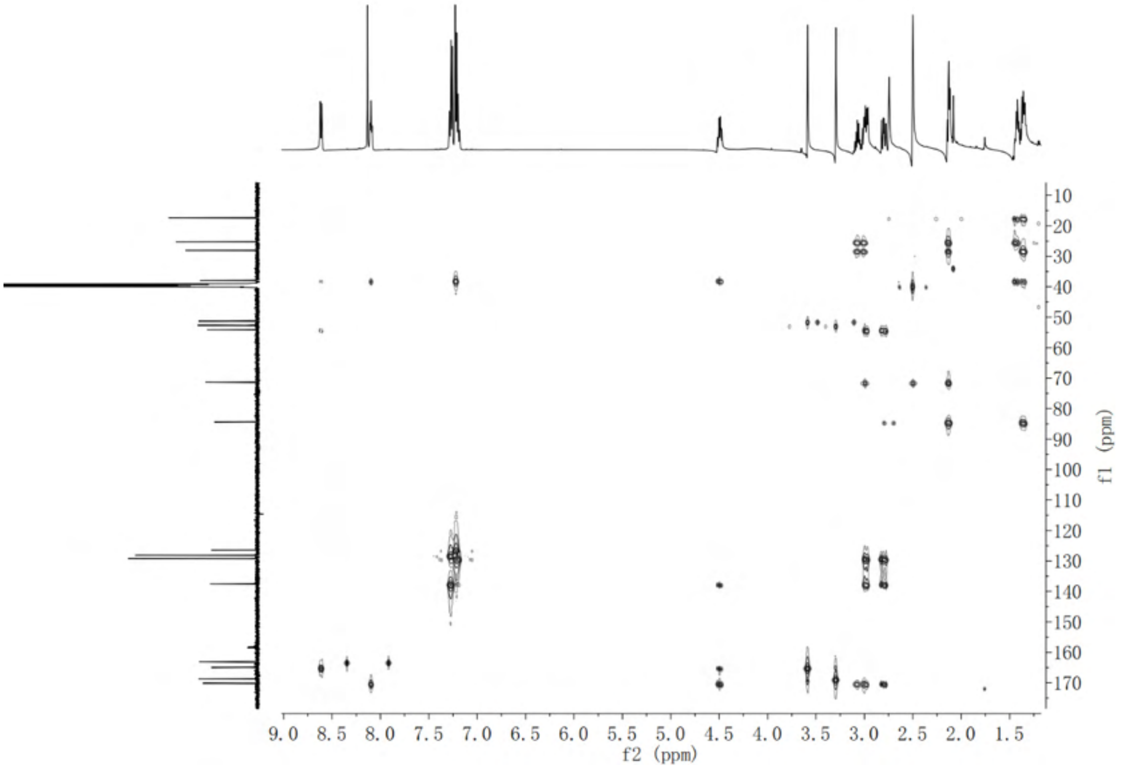

Supplementary Fig. 305. HMBC spectrum of compound (2*S*,3*S*)-*t*-ES-Phe-b24 in DMSO-d_6

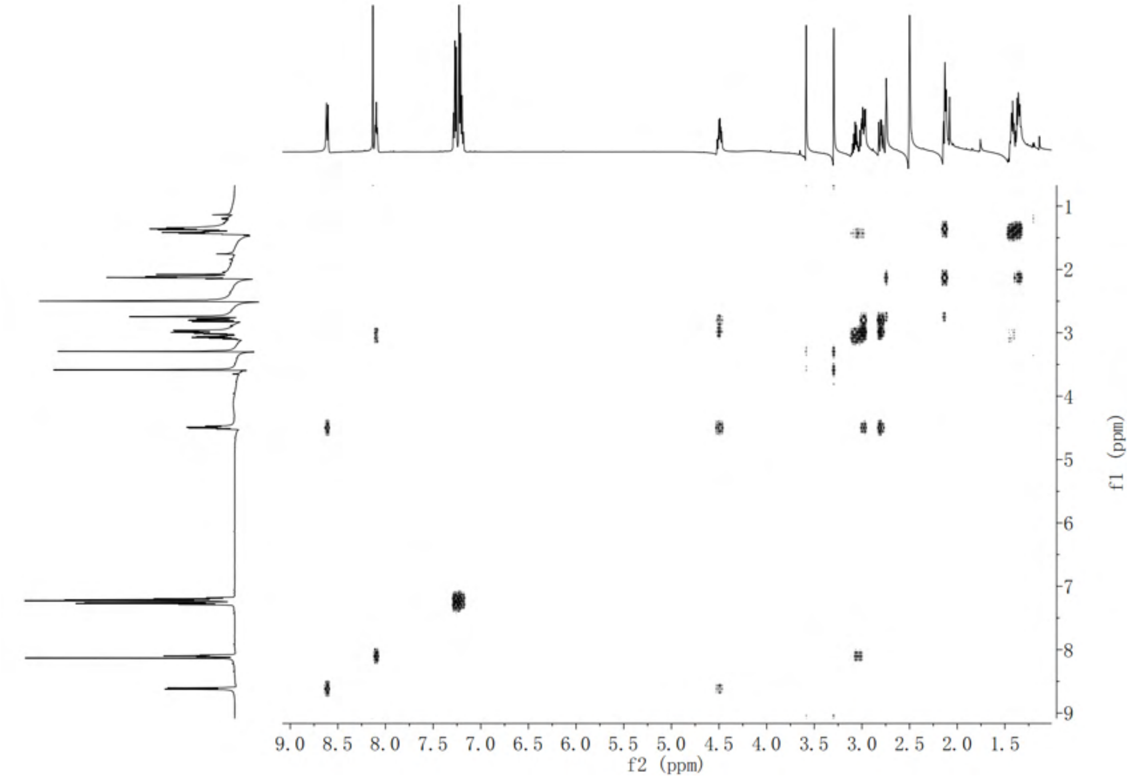

Supplementary Fig. 306. ^{1}H-^{1}H COSY spectrum of compound (2*S*,3*S*)-*t*-ES-Phe-b24 in DMSO-d_6

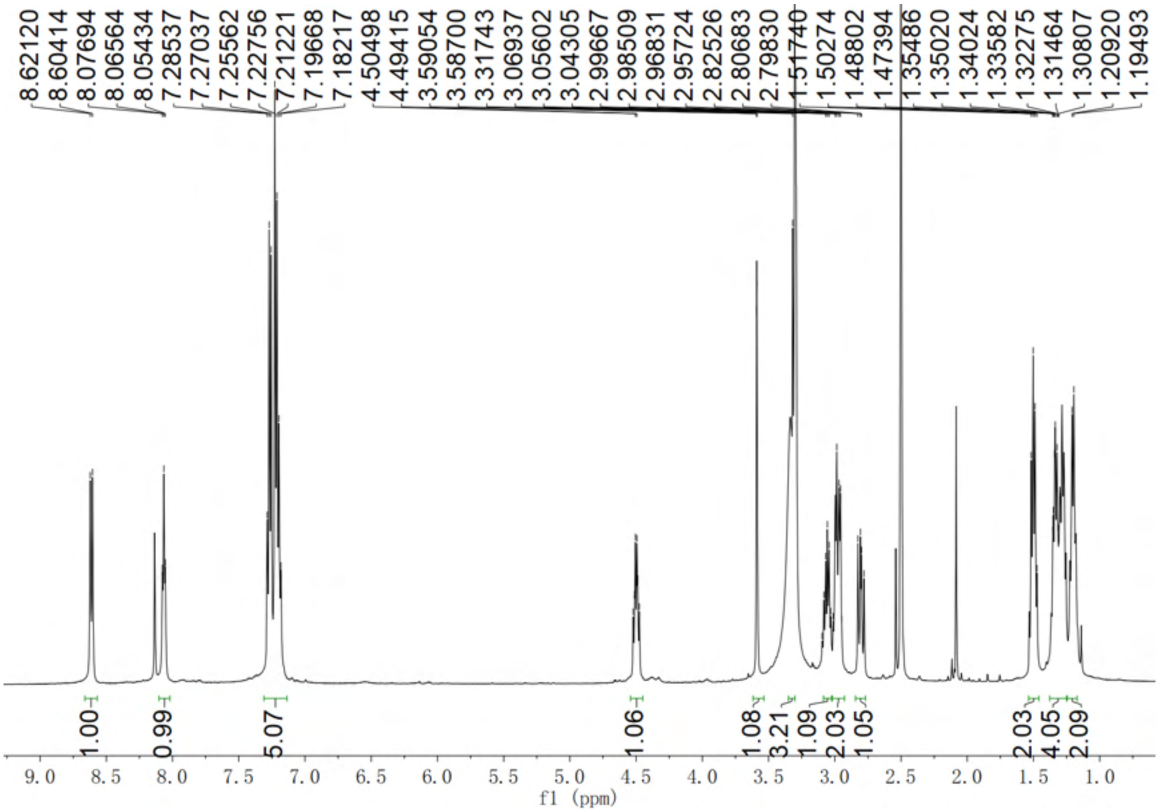

Supplementary Fig. 307. ^{1}H NMR spectrum of compound (2*S*,3*S*)-*t*-ES-Phe-b27 in DMSO-d_6

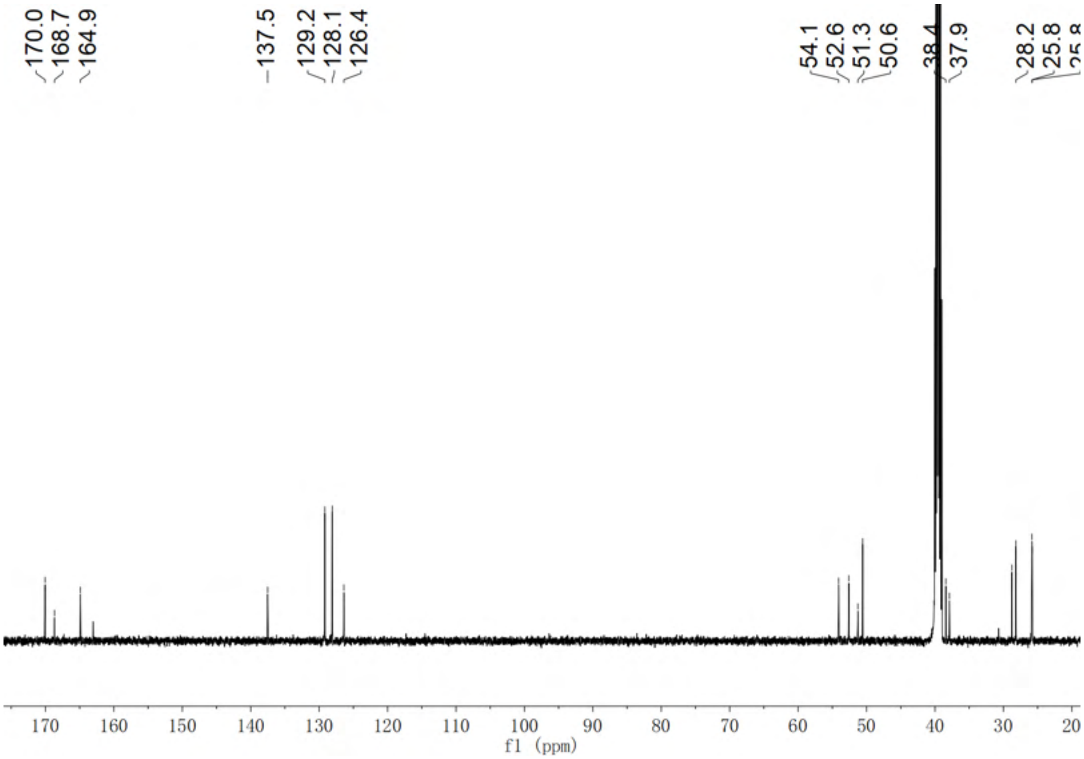

Supplementary Fig. 308. ^{13}C NMR spectrum of compound (2*S*,3*S*)-*t*-ES-Phe-b27 in DMSO-d_6

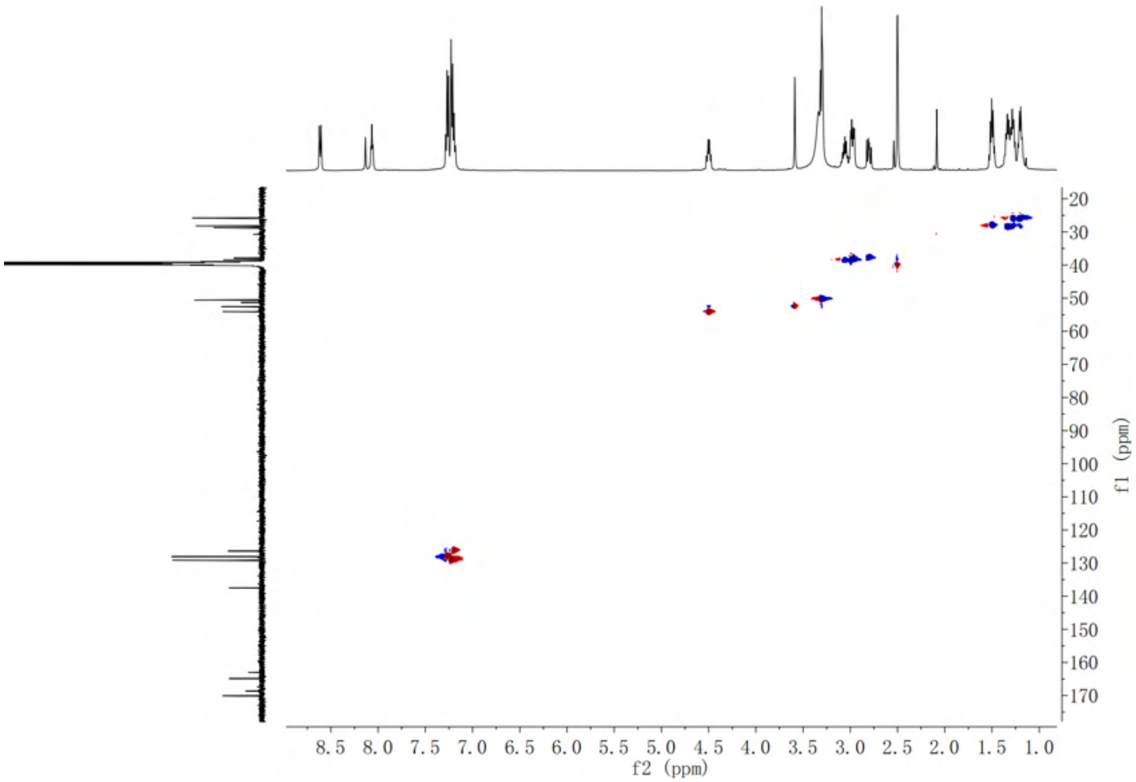

Supplementary Fig. 309. HSQC spectrum of compound (2*S*,3*S*)-*t*-ES-Phe-b27 in DMSO-*d*$_6$

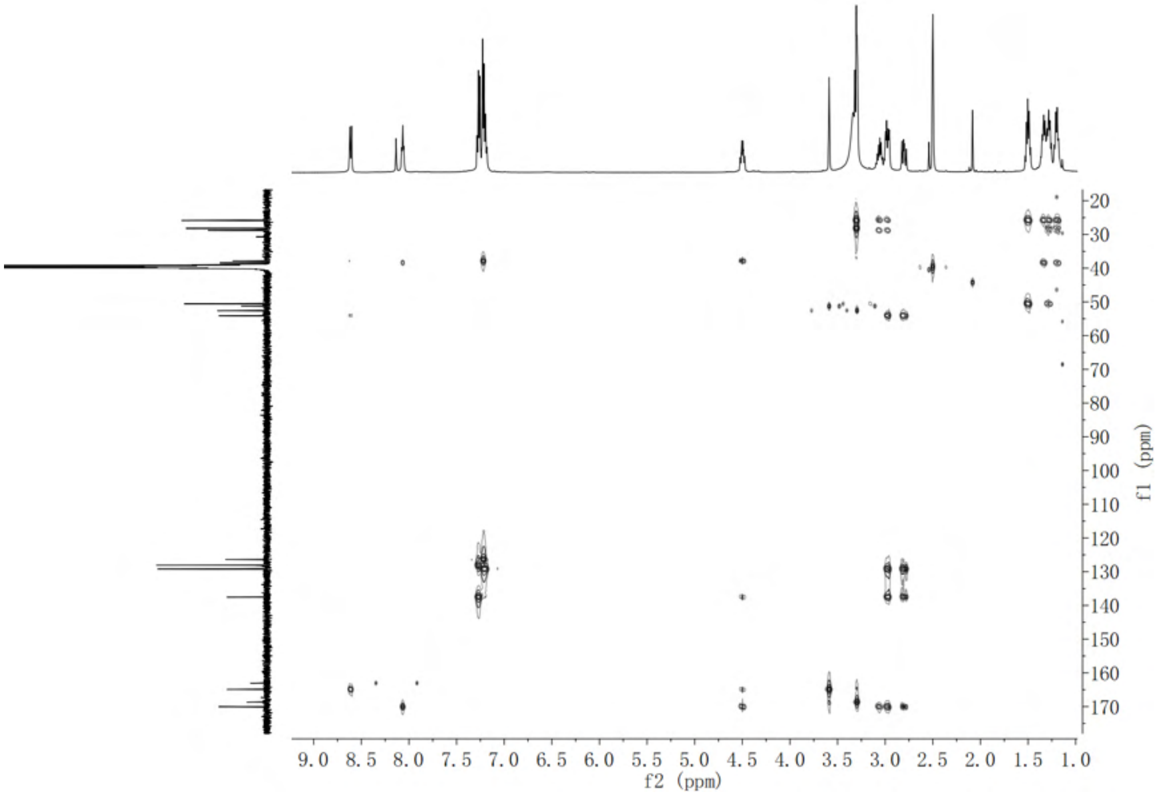

Supplementary Fig. 310. HMBC spectrum of compound (2*S*,3*S*)-*t*-ES-Phe-b27 in DMSO-*d*$_6$

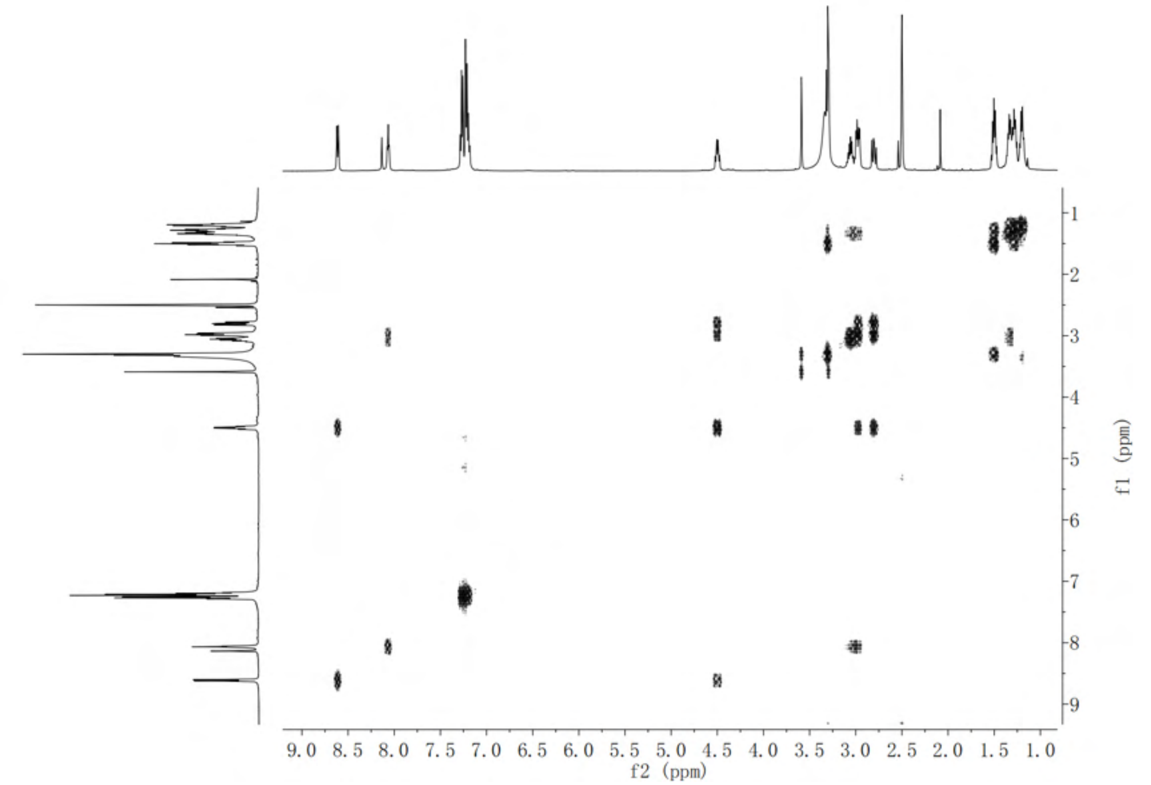

Supplementary Fig. 311. ^{1}**H-**^{1}**H COSY spectrum of compound (2*S*,3*S*)-*t*-ES-Phe-b27 in DMSO-*d*$_6$**

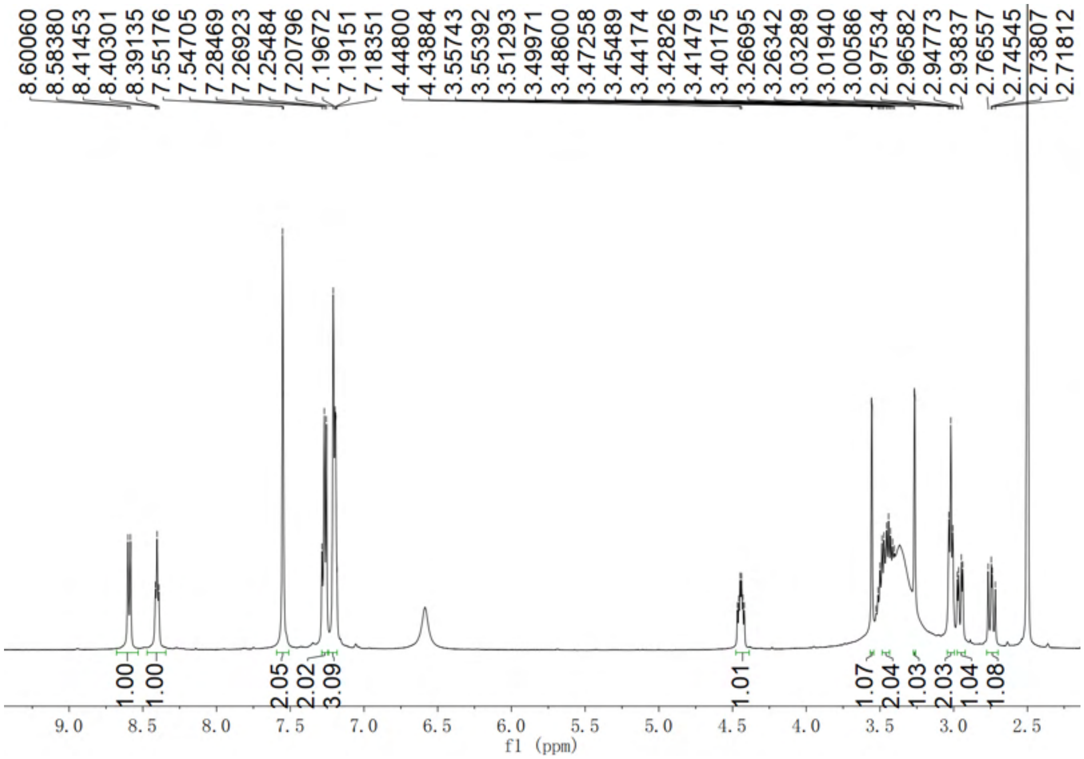

Supplementary Fig. 312. ^{1}**H NMR spectrum of compound (2*S*,3*S*)-*t*-ES-Phe-b29 in DMSO-*d*$_6$**

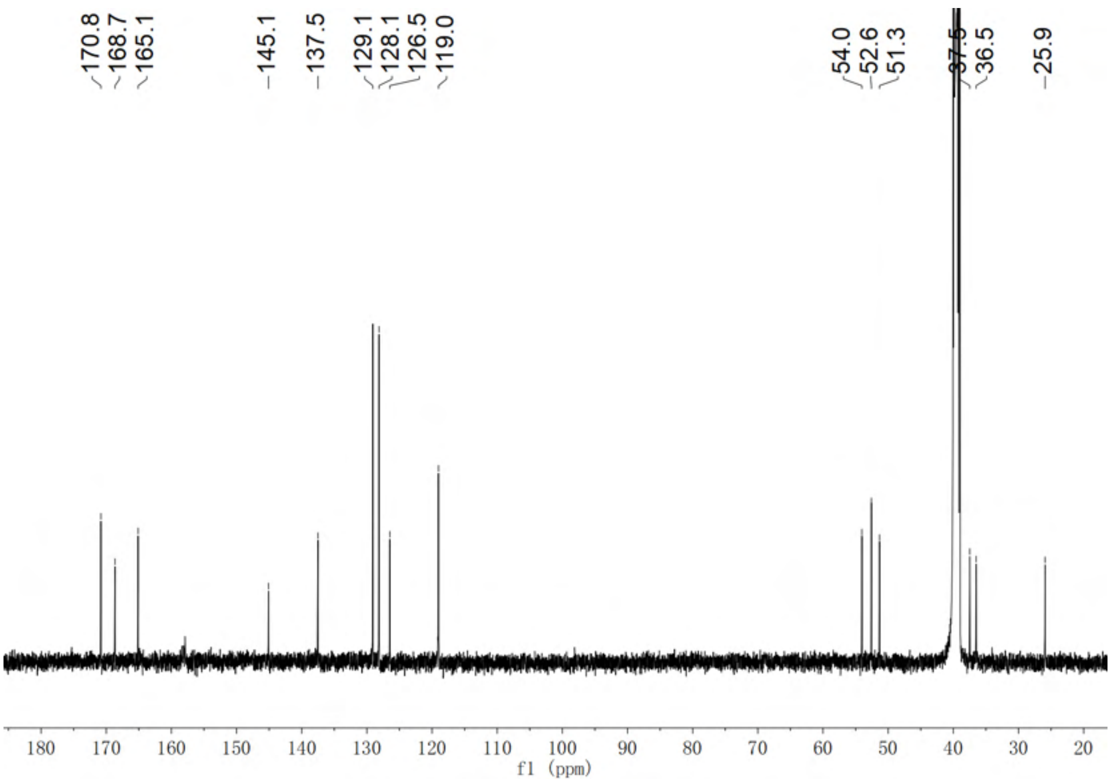

Supplementary Fig. 313. ^{13}C NMR spectrum of compound (2*S*,3*S*)-*t*-ES-Phe-b29 in DMSO-d_6

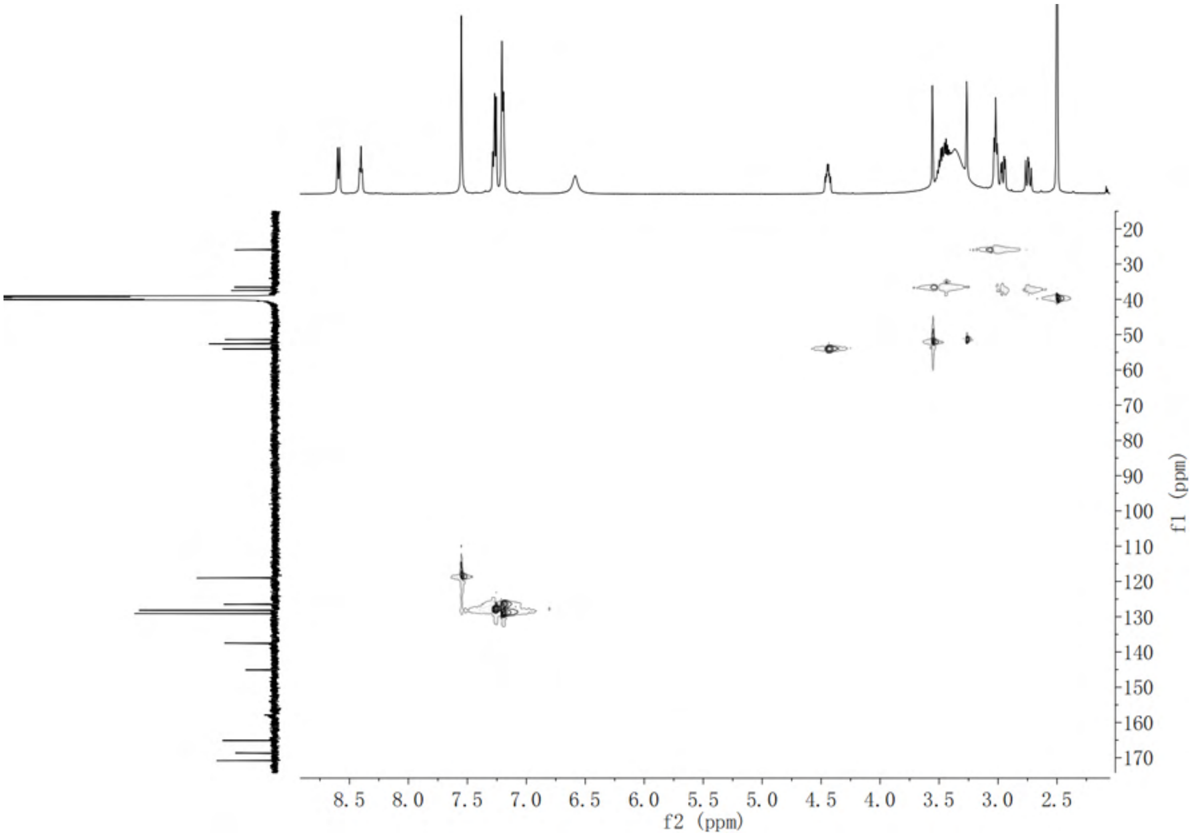

Supplementary Fig. 314. HSQC spectrum of compound (2*S*,3*S*)-*t*-ES-Phe-b29 in DMSO-d_6

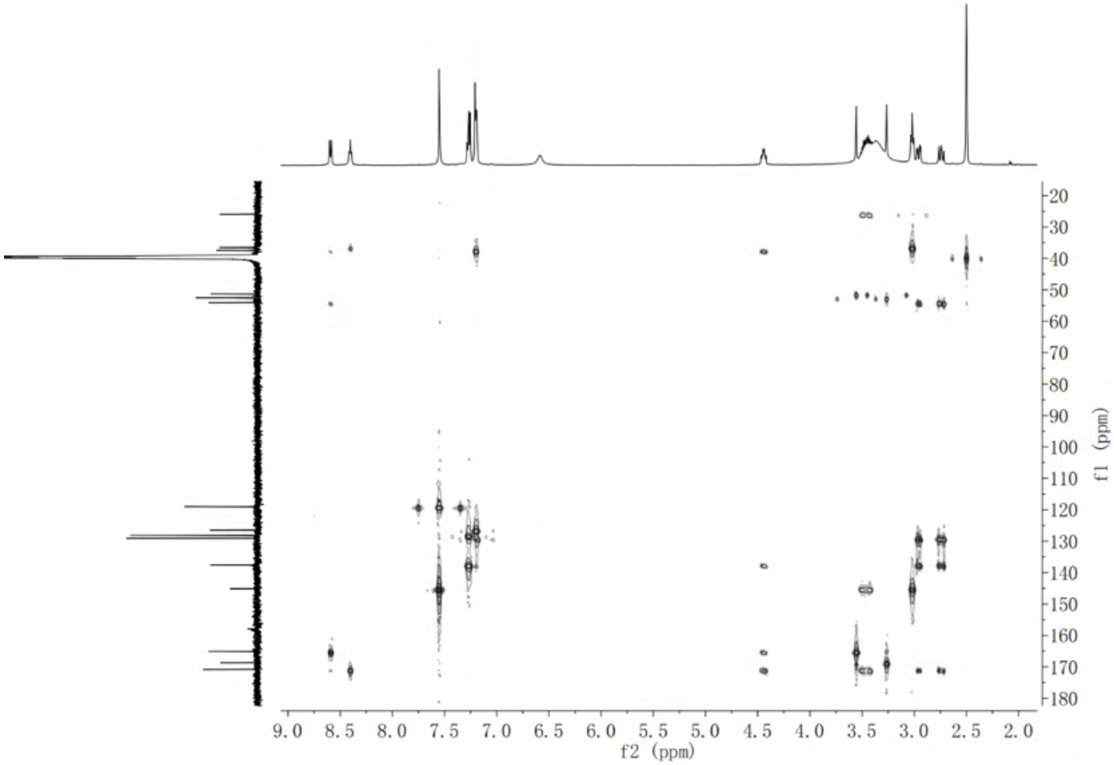

Supplementary Fig. 315. HMBC spectrum of compound (2*S*,3*S*)-*t*-ES-Phe-b29 in DMSO-d_6

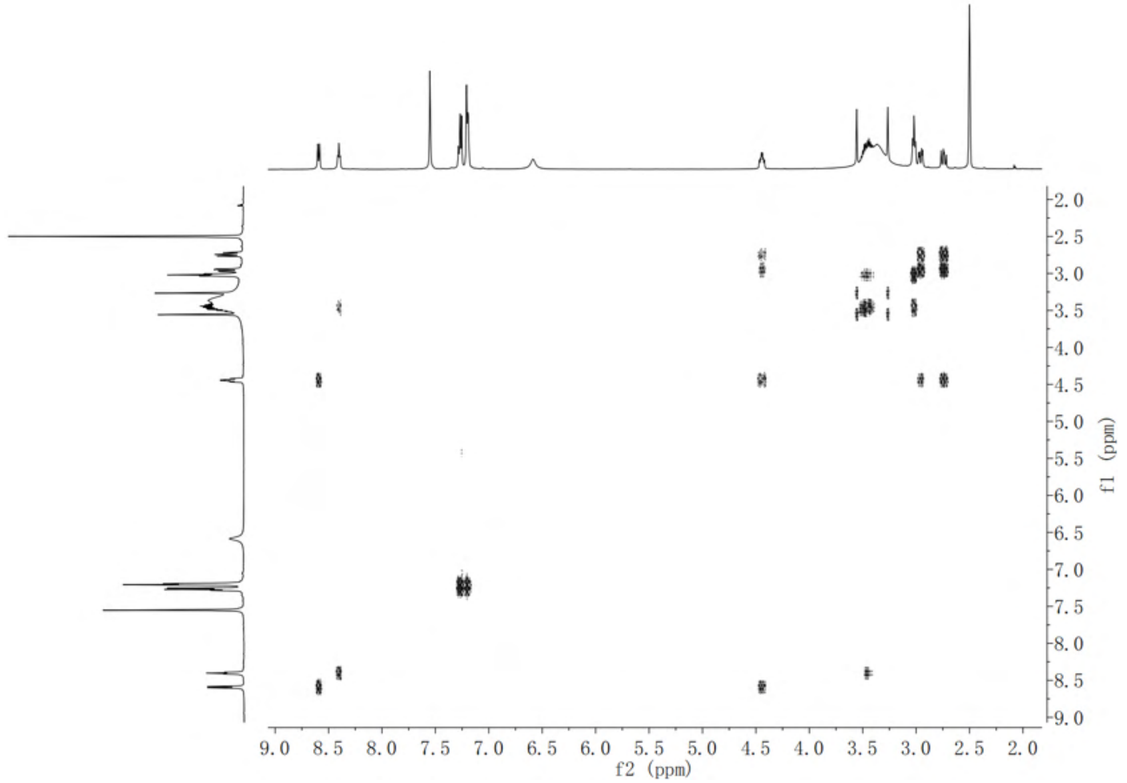

Supplementary Fig. 316. ^{1}H-^{1}H COSY spectrum of compound (2*S*,3*S*)-*t*-ES-Phe-b29 in DMSO-d_6

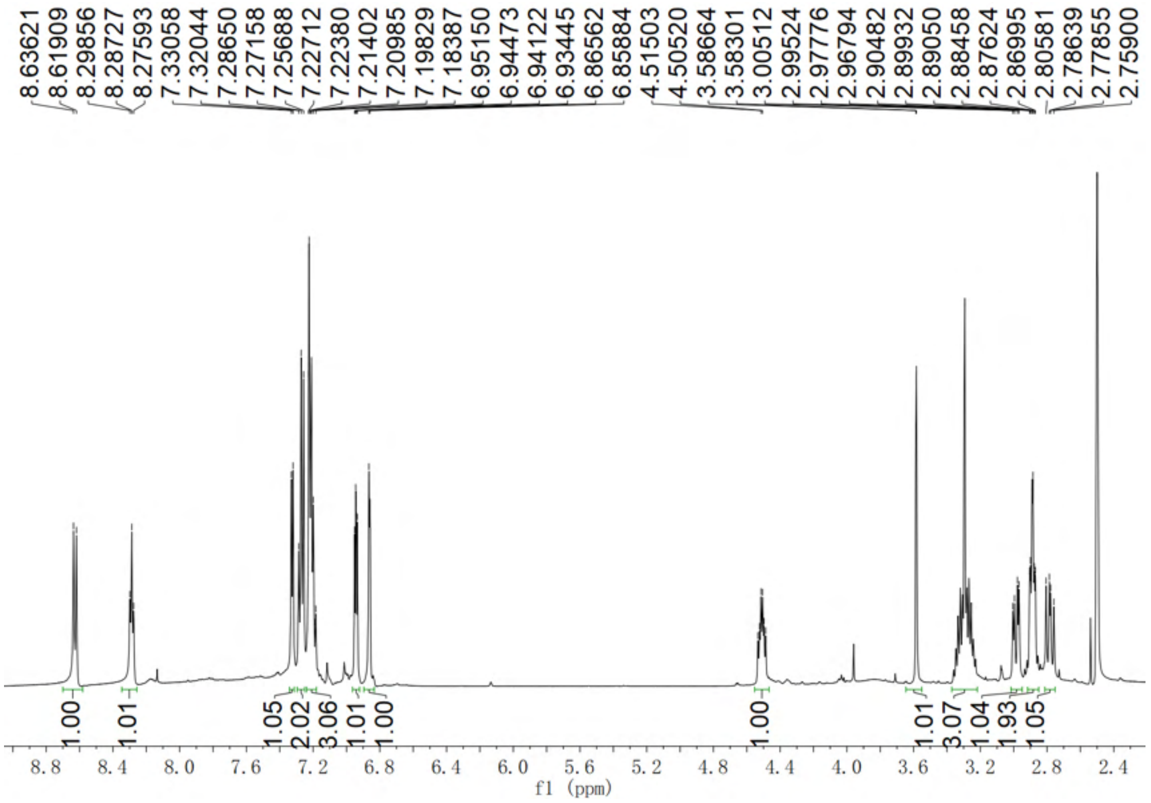

Supplementary Fig. 317. ^{1}H NMR spectrum of compound (2*S*,3*S*)-*t*-ES-Phe-b30 in DMSO-d_6

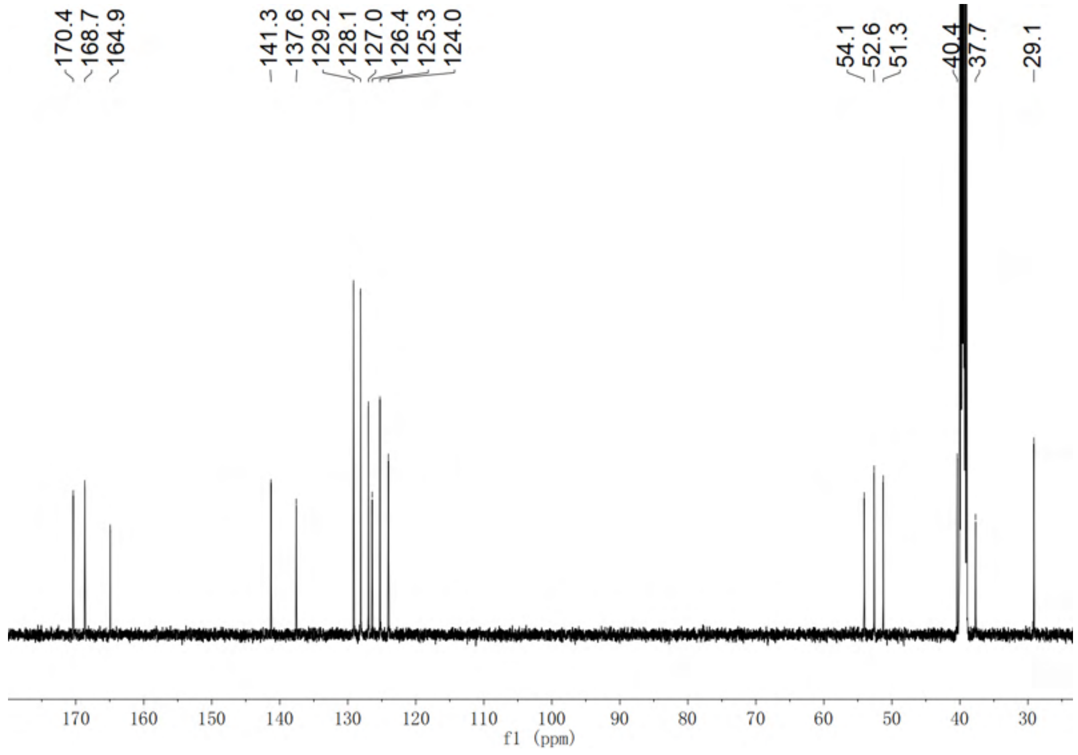

Supplementary Fig. 318. ^{13}C NMR spectrum of compound (2*S*,3*S*)-*t*-ES-Phe-b30 in DMSO-d_6

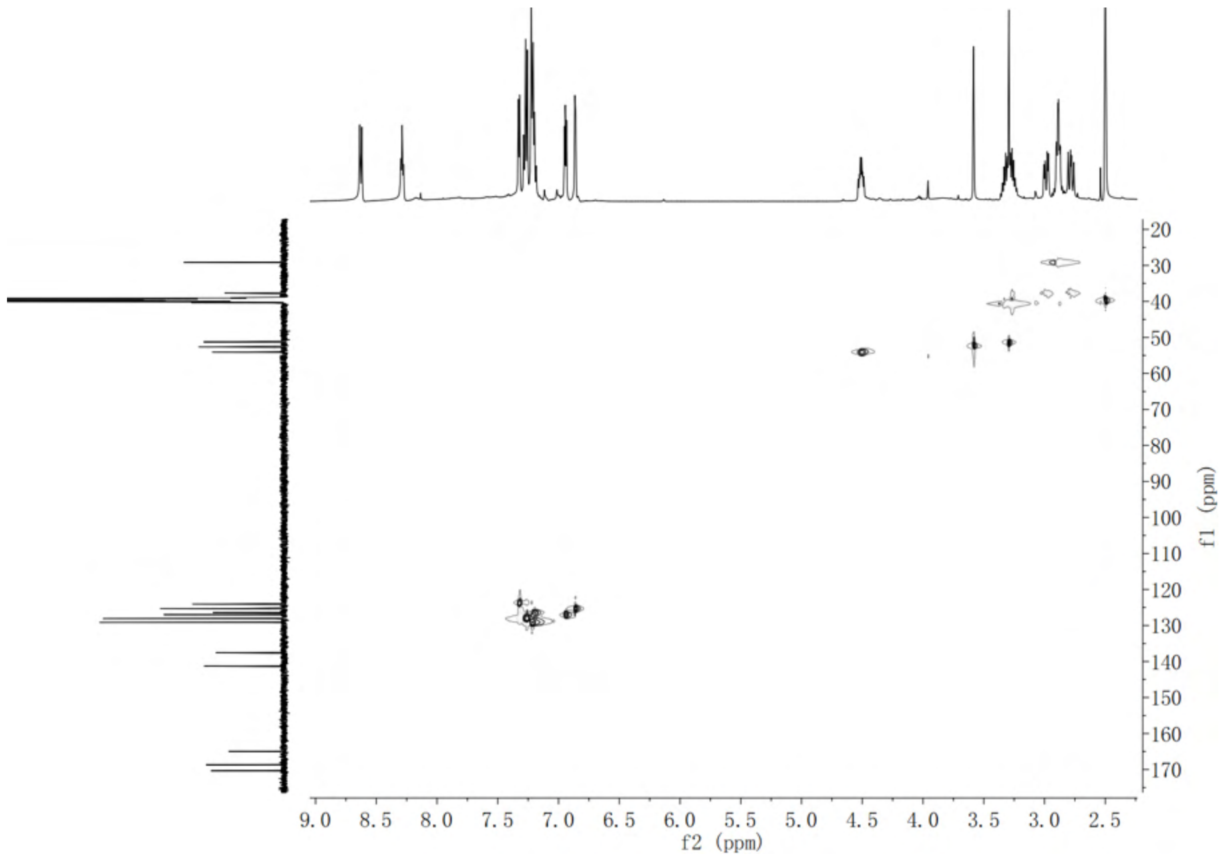

Supplementary Fig. 319. HSQC spectrum of compound (2*S*,3*S*)-*t*-ES-Phe-b30 in DMSO-d_6

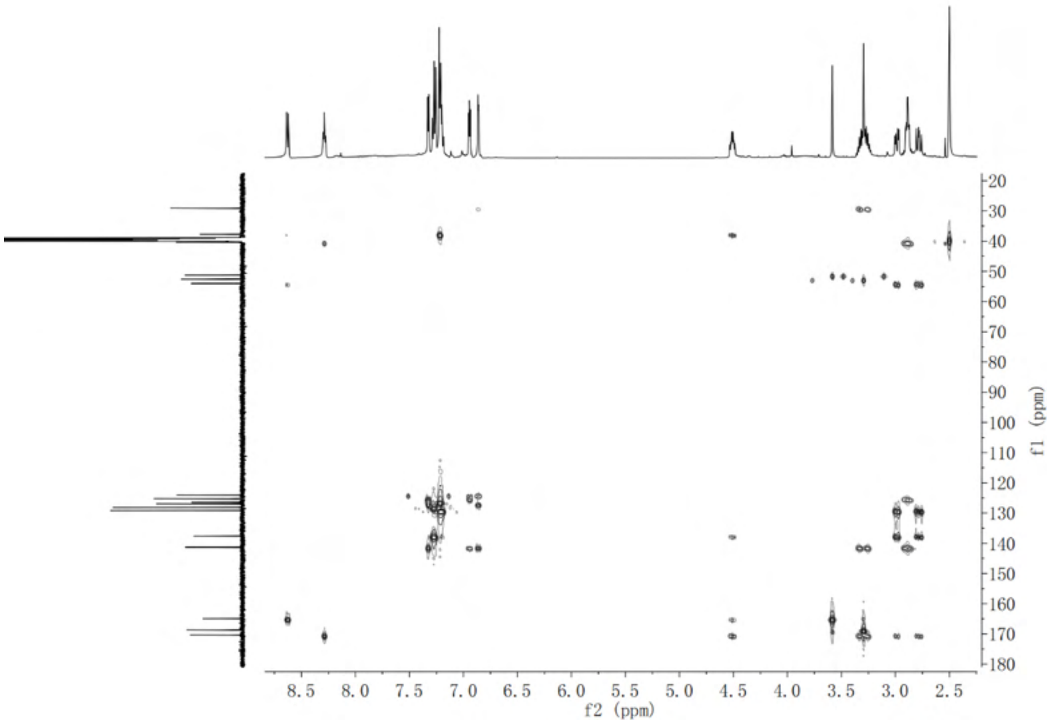

Supplementary Fig. 320. HMBC spectrum of compound (2*S*,3*S*)-*t*-ES-Phe-b30 in DMSO-d_6

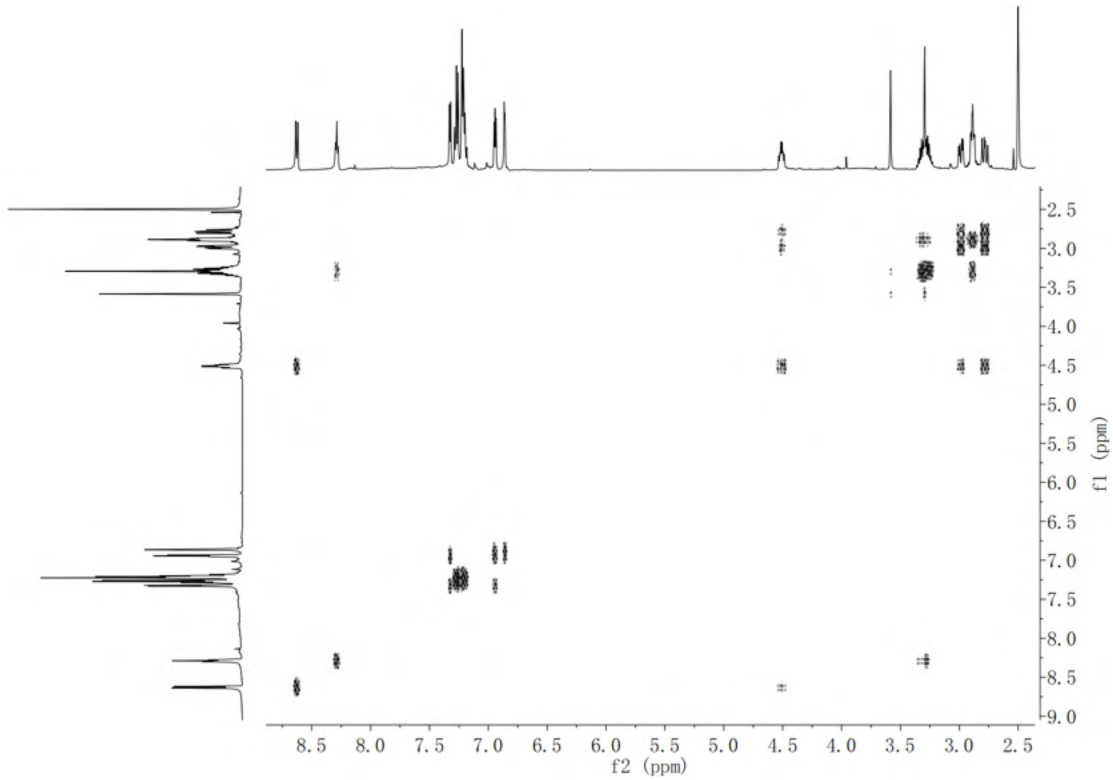

Supplementary Fig. 321. [1]**H-**[1]**H COSY spectrum of compound (2*S*,3*S*)-*t*-ES-Phe-b30 in DMSO-*d*₆**

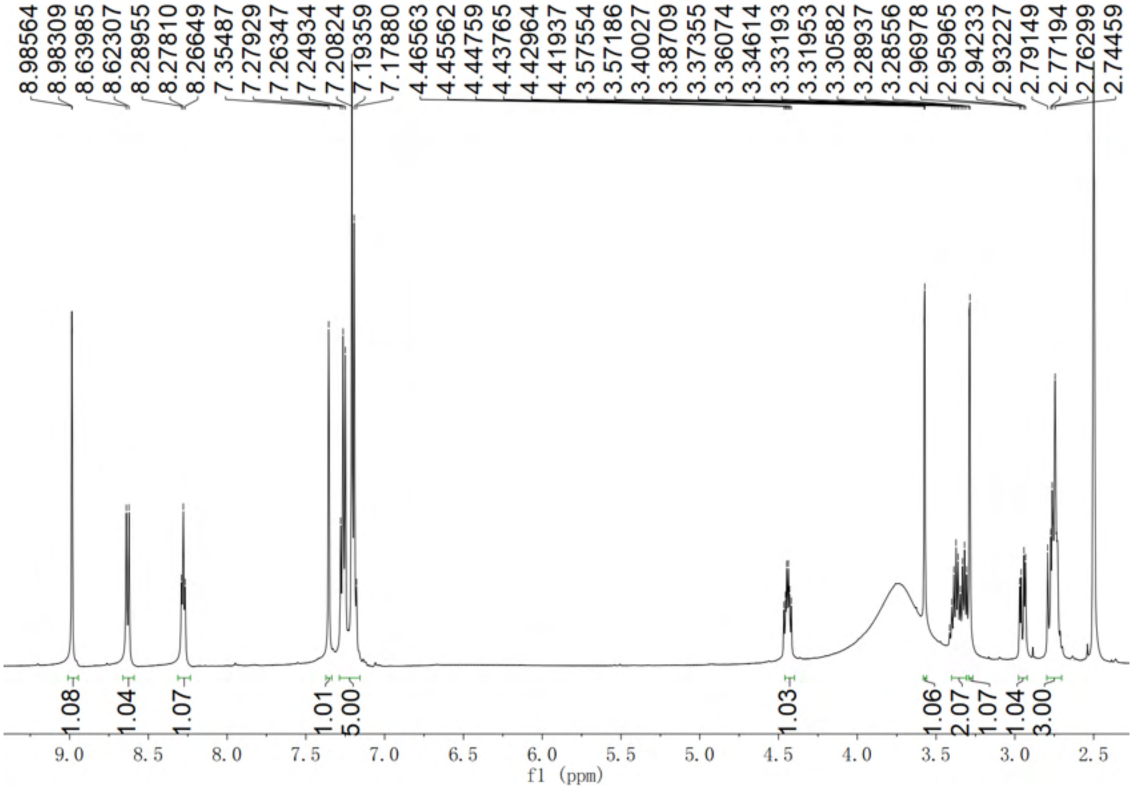

Supplementary Fig. 322. [1]**H NMR spectrum of compound (2*S*,3*S*)-*t*-ES-Phe-b31 in DMSO-*d*₆**

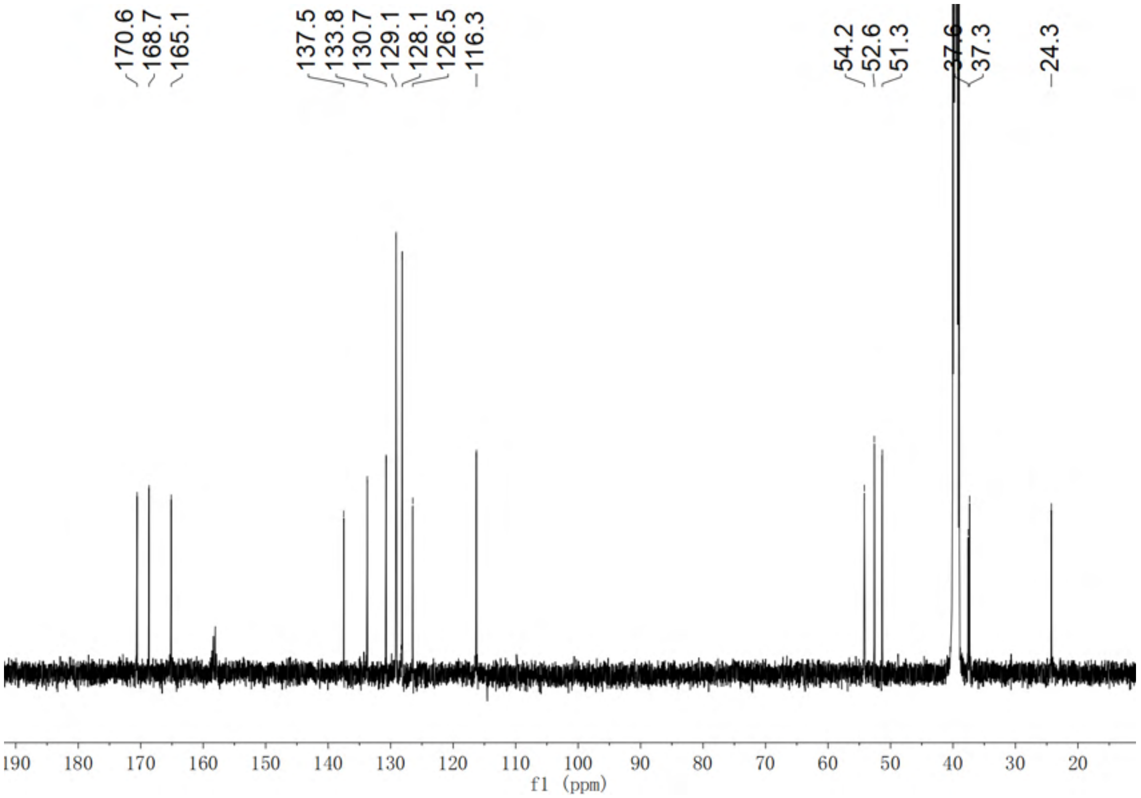

Supplementary Fig. 323. ^{13}**C NMR spectrum of compound (2*S*,3*S*)-*t*-ES-Phe-b31 in DMSO-*d*$_6$**

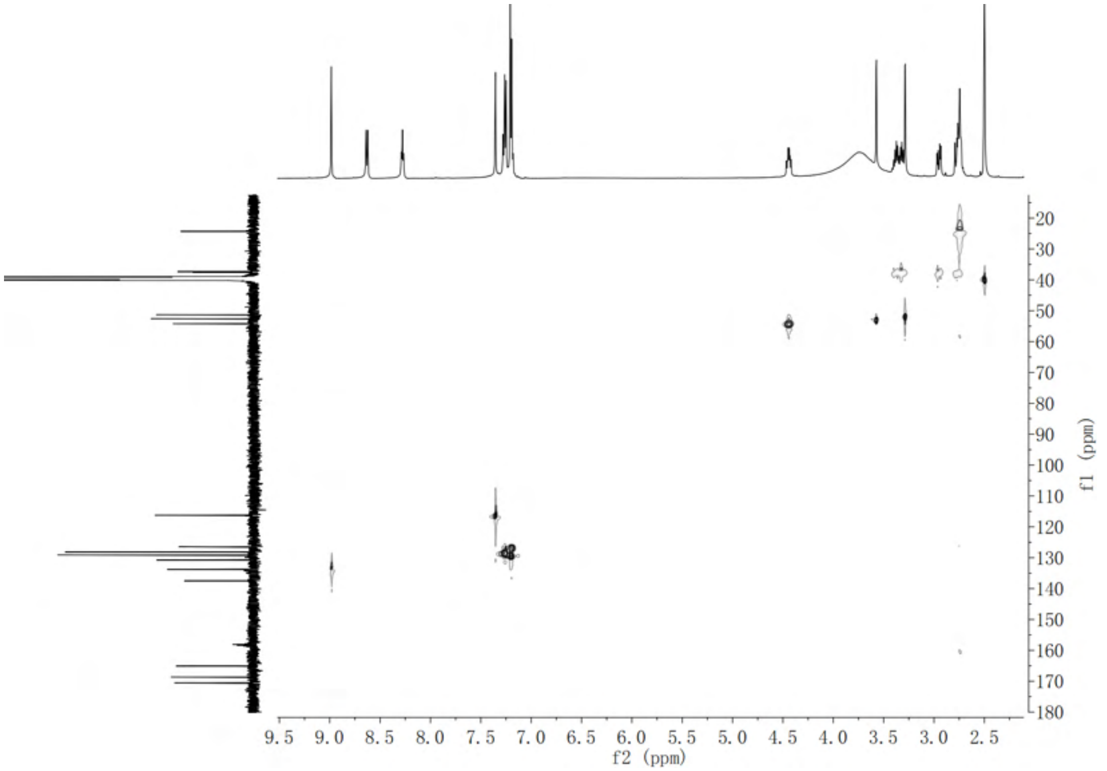

Supplementary Fig. 324. HSQC spectrum of compound (2*S*,3*S*)-*t*-ES-Phe-b31 in DMSO-*d*$_6$

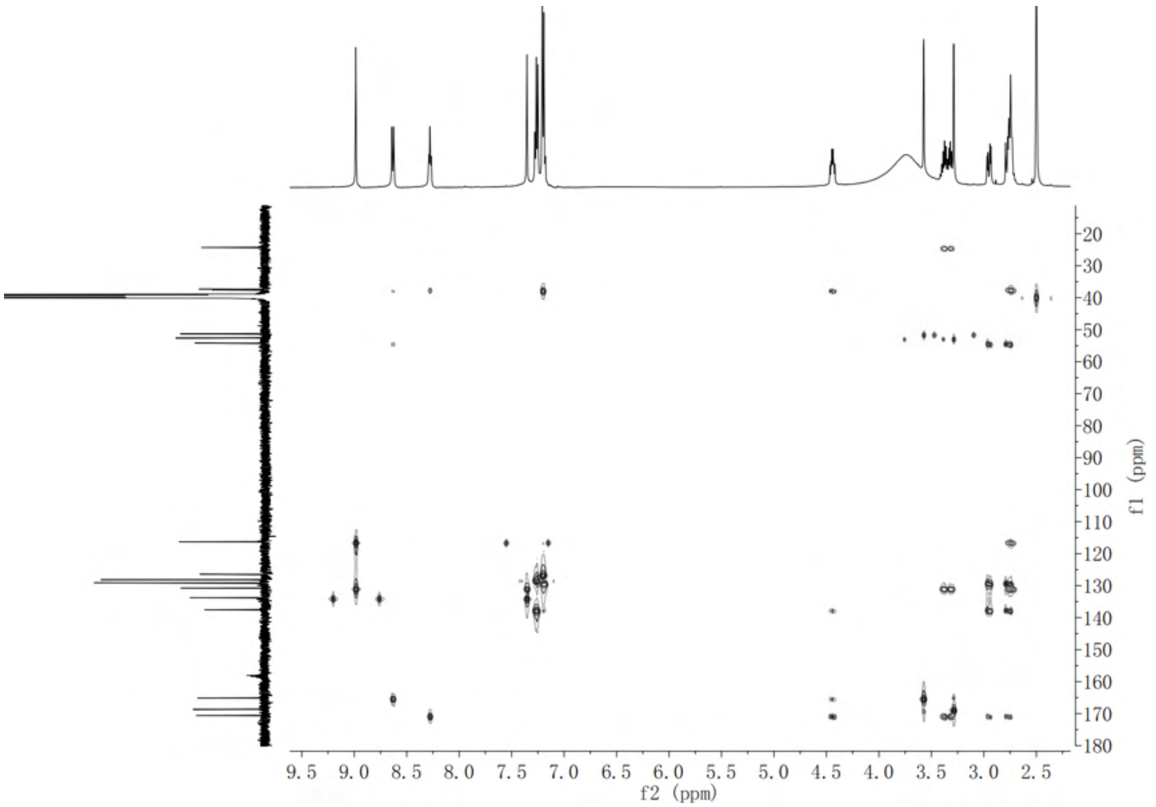

Supplementary Fig. 325. HMBC spectrum of compound (2*S*,3*S*)-*t*-ES-Phe-b31 in DMSO-d_6

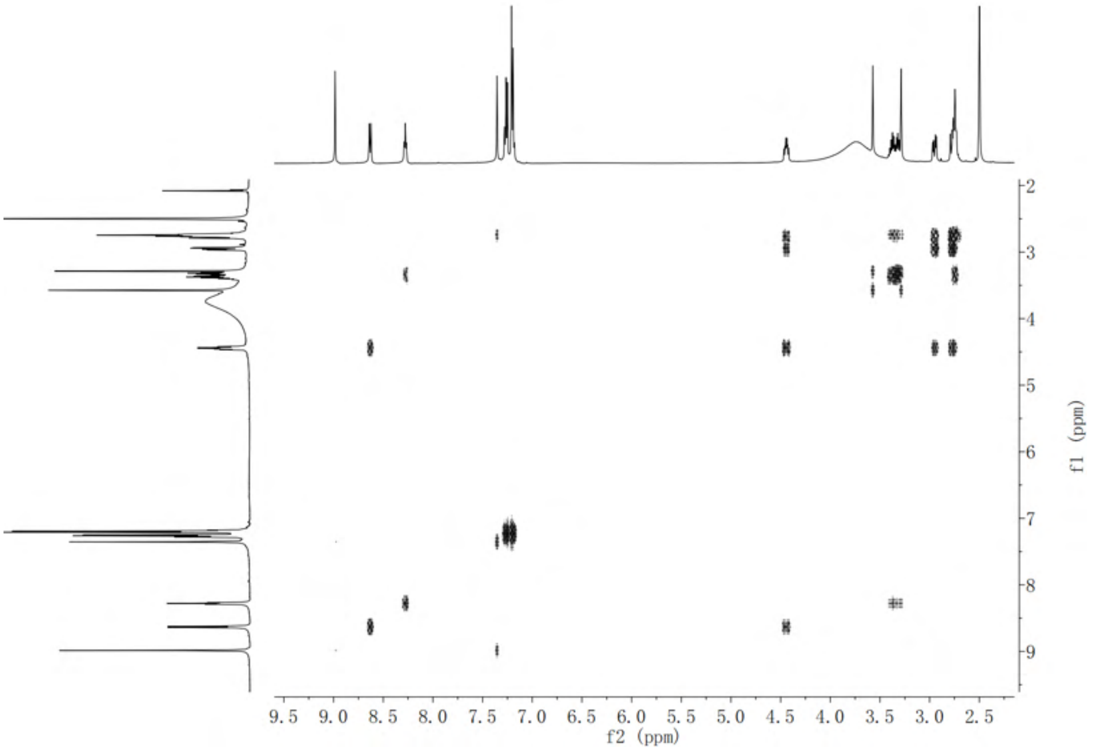

Supplementary Fig. 326. ^{1}H-^{1}H COSY spectrum of compound (2*S*,3*S*)-*t*-ES-Phe-b31 in DMSO-d_6

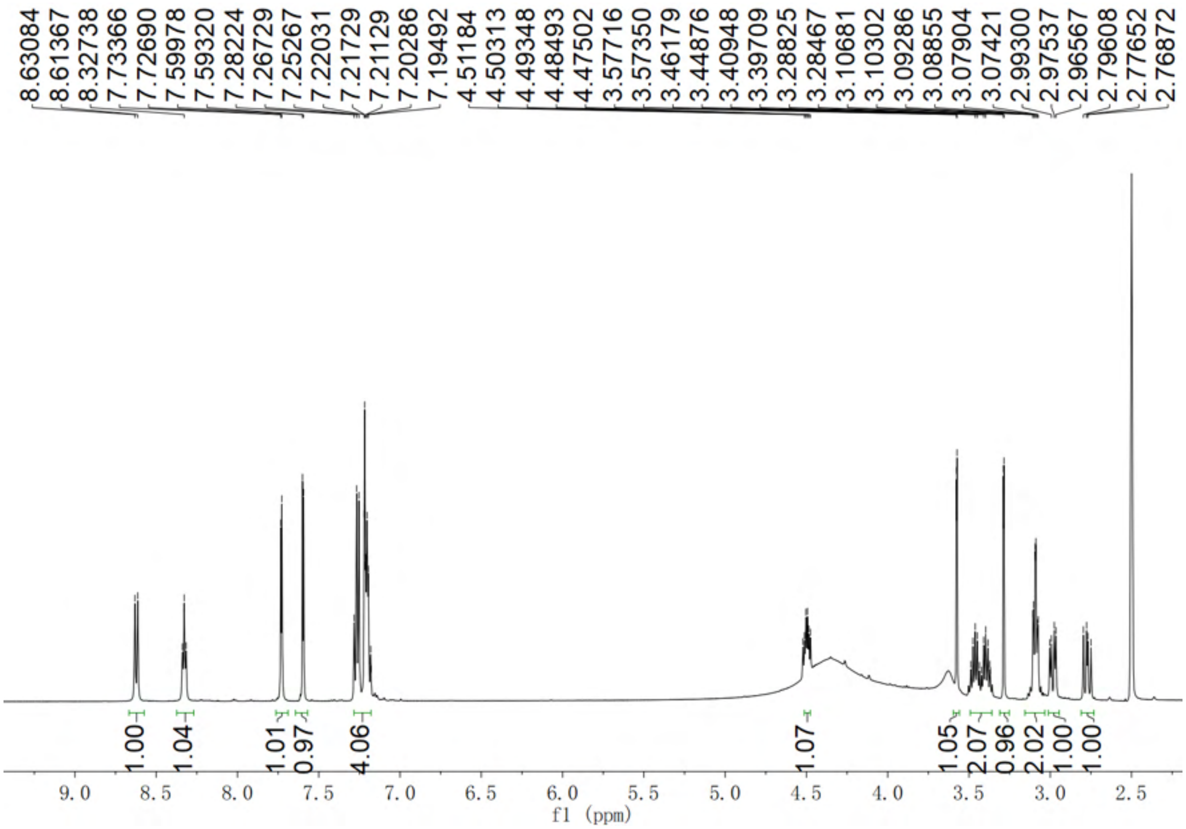

Supplementary Fig. 327. **^{1}H NMR spectrum of compound (2*S*,3*S*)-*t*-ES-Phe-b32 in DMSO-d_6**

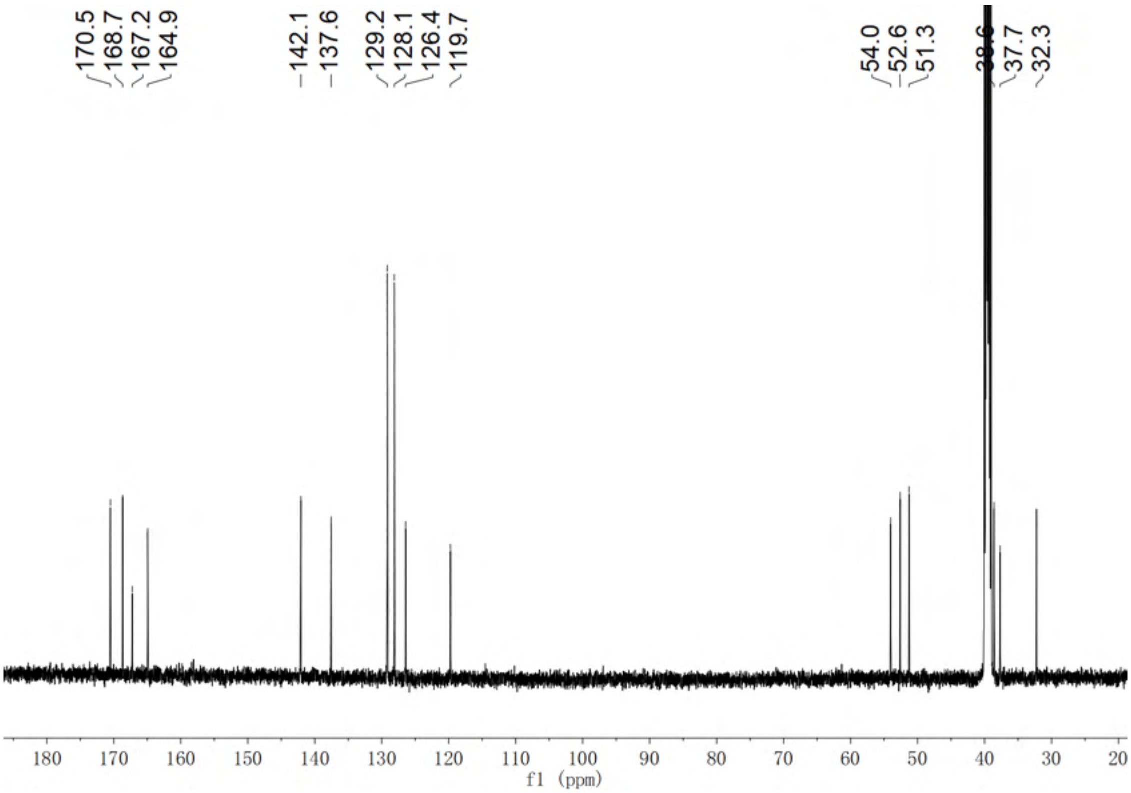

Supplementary Fig. 328. **^{13}C NMR spectrum of compound (2*S*,3*S*)-*t*-ES-Phe-b32 in DMSO-d_6**

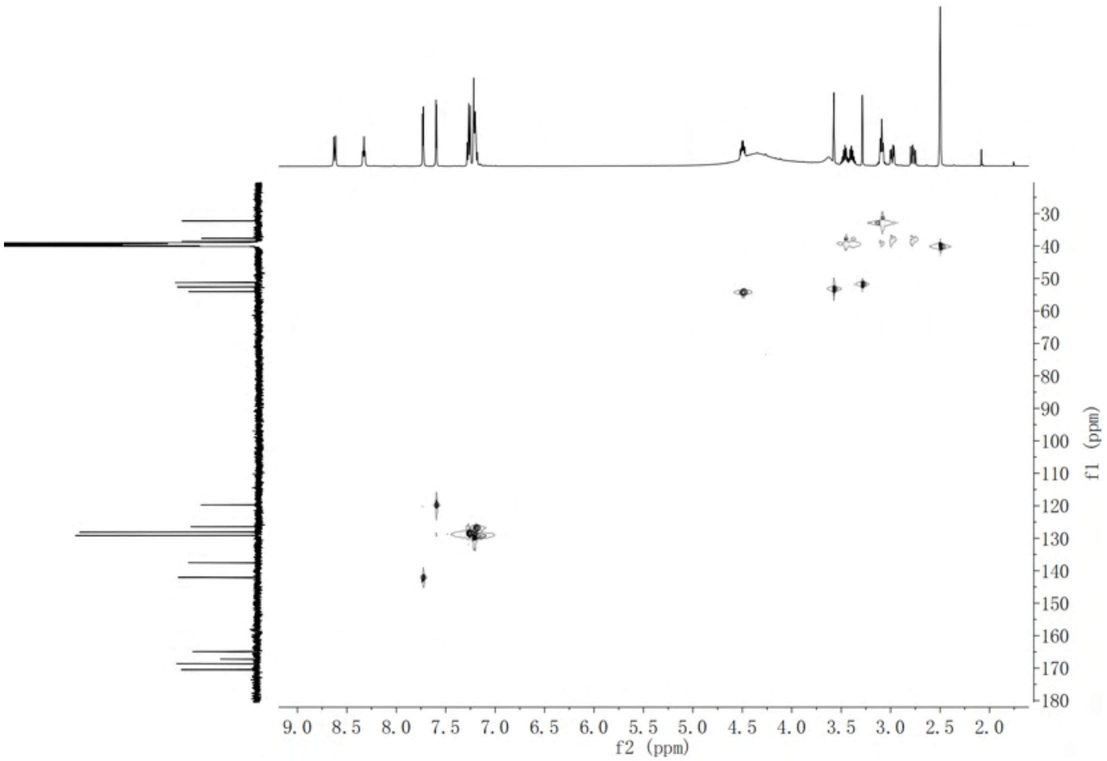

Supplementary Fig. 329. HSQC spectrum of compound (2*S*,3*S*)-*t*-ES-Phe-b32 in DMSO-d_6

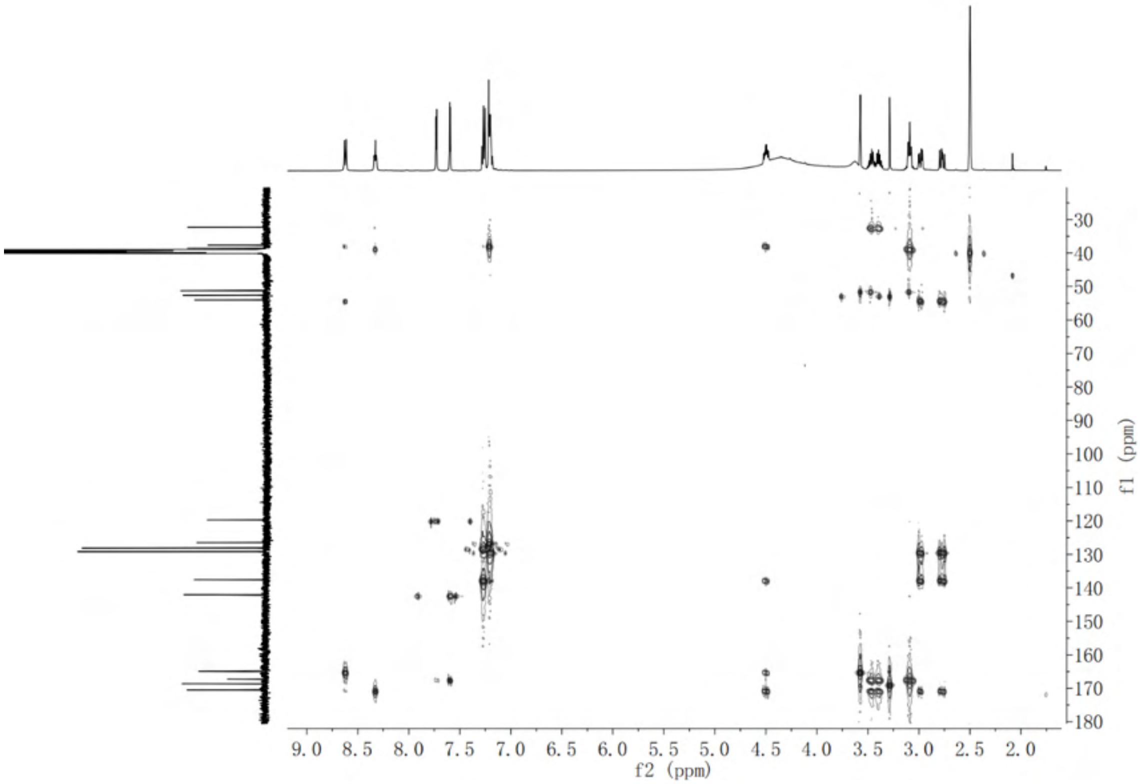

Supplementary Fig. 330. HMBC spectrum of compound (2*S*,3*S*)-*t*-ES-Phe-b32 in DMSO-d_6

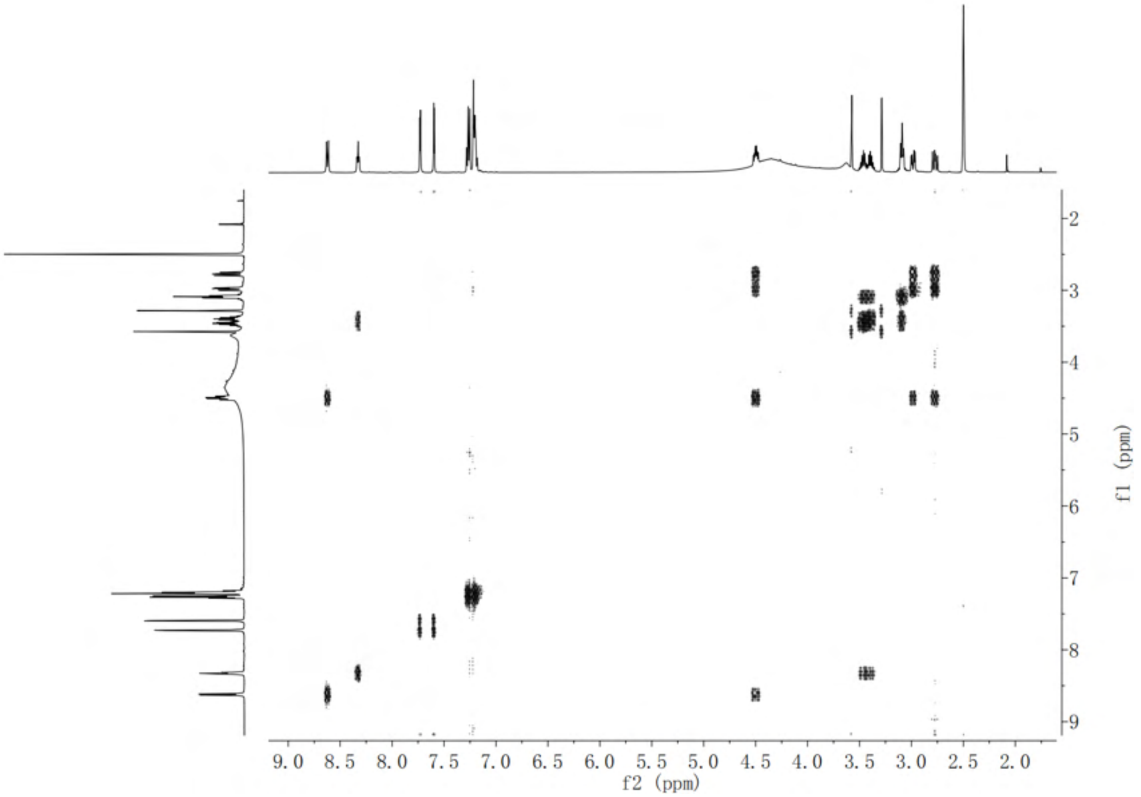

Supplementary Fig. 331. ^1H-^1H COSY spectrum of compound (2*S*,3*S*)-*t*-ES-Phe-b32 in DMSO-d_6

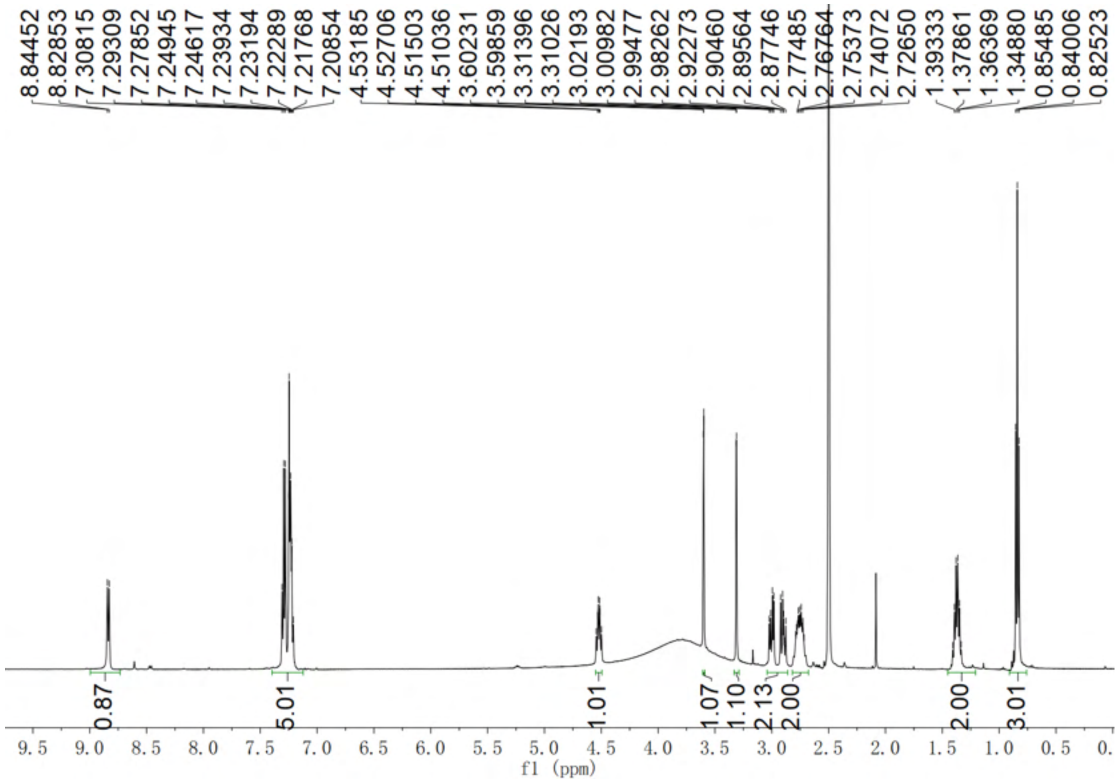

Supplementary Fig. 332. ^1H NMR spectrum of compound (2*S*,3*S*)-*t*-ES-Phe-b33 in DMSO-d_6

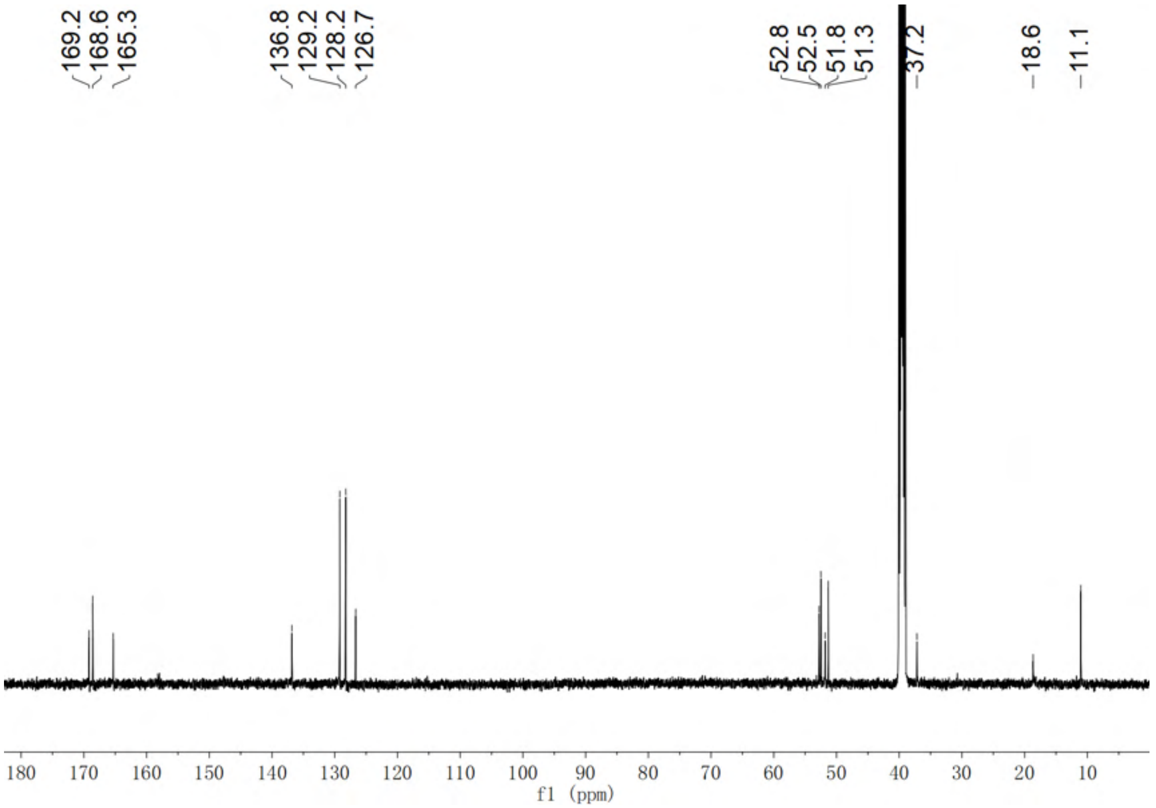

Supplementary Fig. 333. ^{13}C NMR spectrum of compound (2*S*,3*S*)-*t*-ES-Phe-b33 in DMSO-d_6

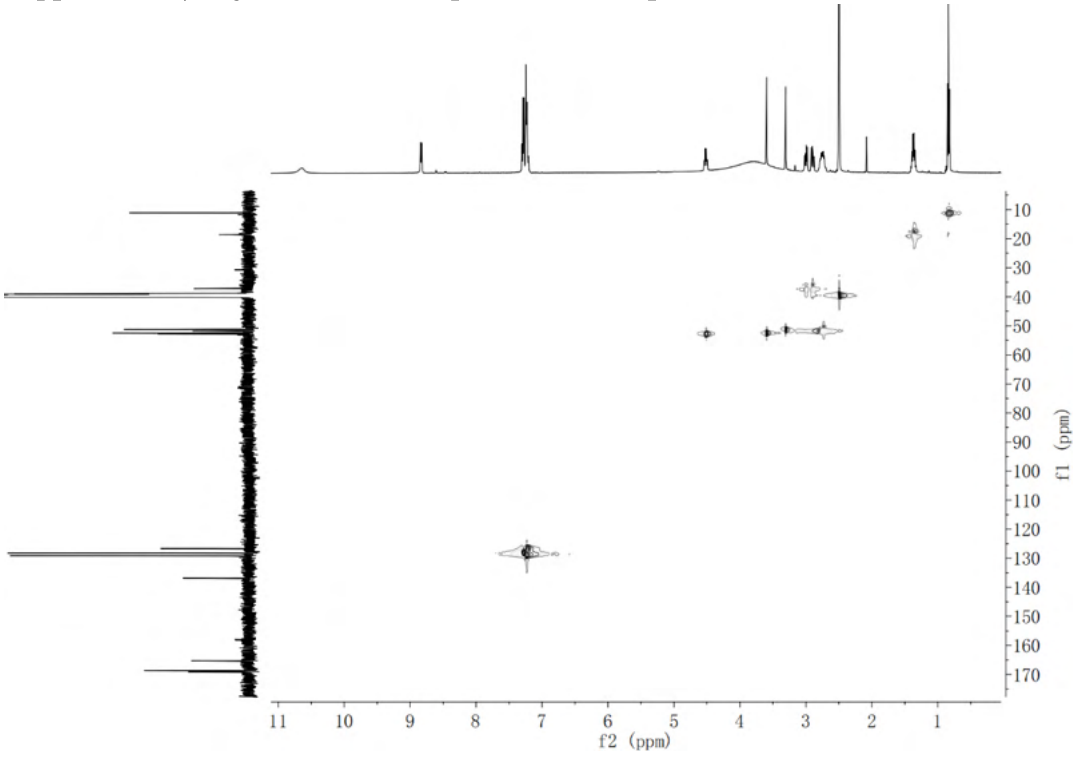

Supplementary Fig. 334. HSQC spectrum of compound (2*S*,3*S*)-*t*-ES-Phe-b33 in DMSO-d_6

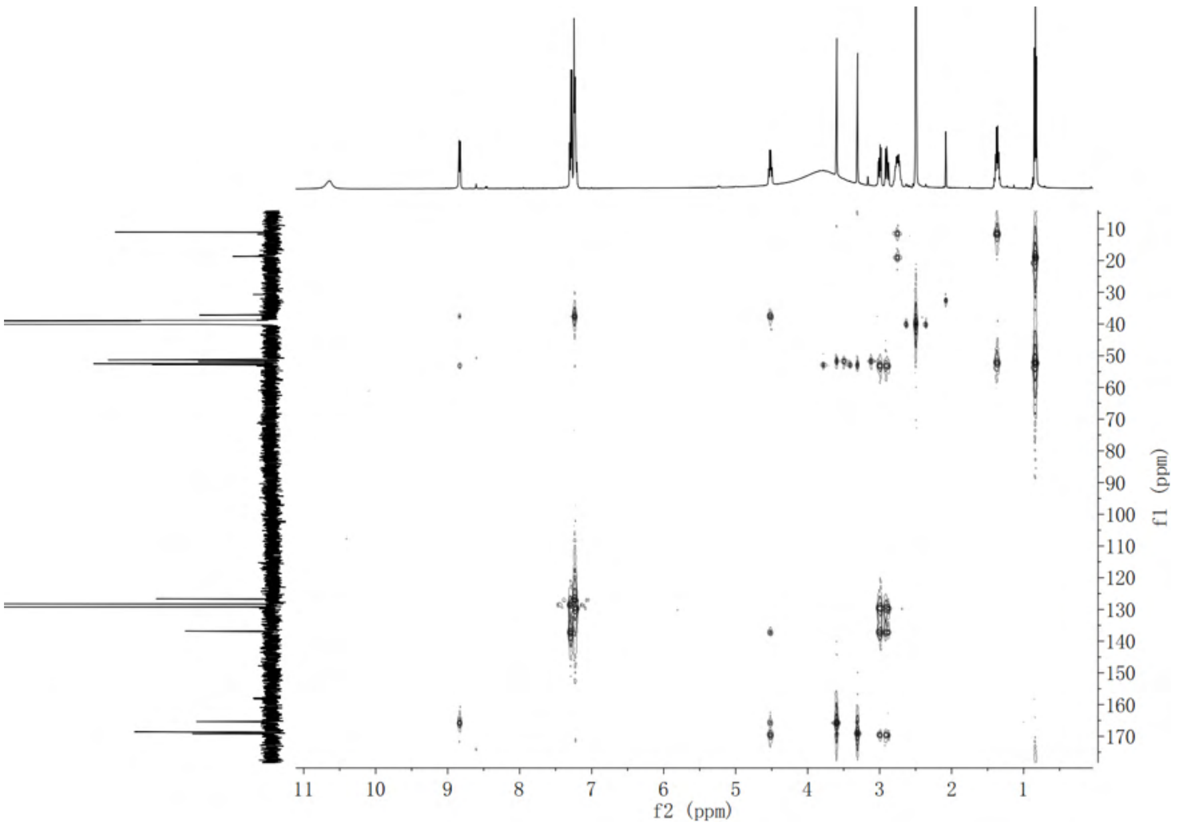

Supplementary Fig. 335. HMBC spectrum of compound (2*S*,3*S*)-*t*-ES-Phe-b33 in DMSO-d_6

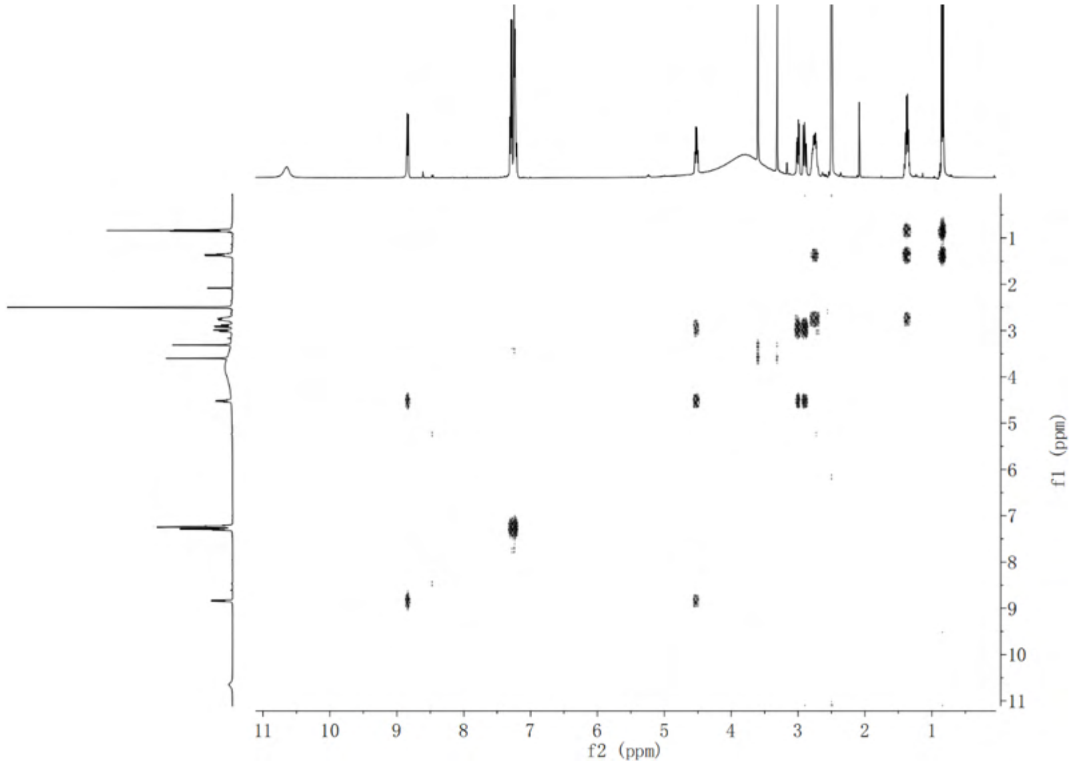

Supplementary Fig. 336. ^{1}H-^{1}H COSY spectrum of compound (2*S*,3*S*)-*t*-ES-Phe-b33 in DMSO-d_6

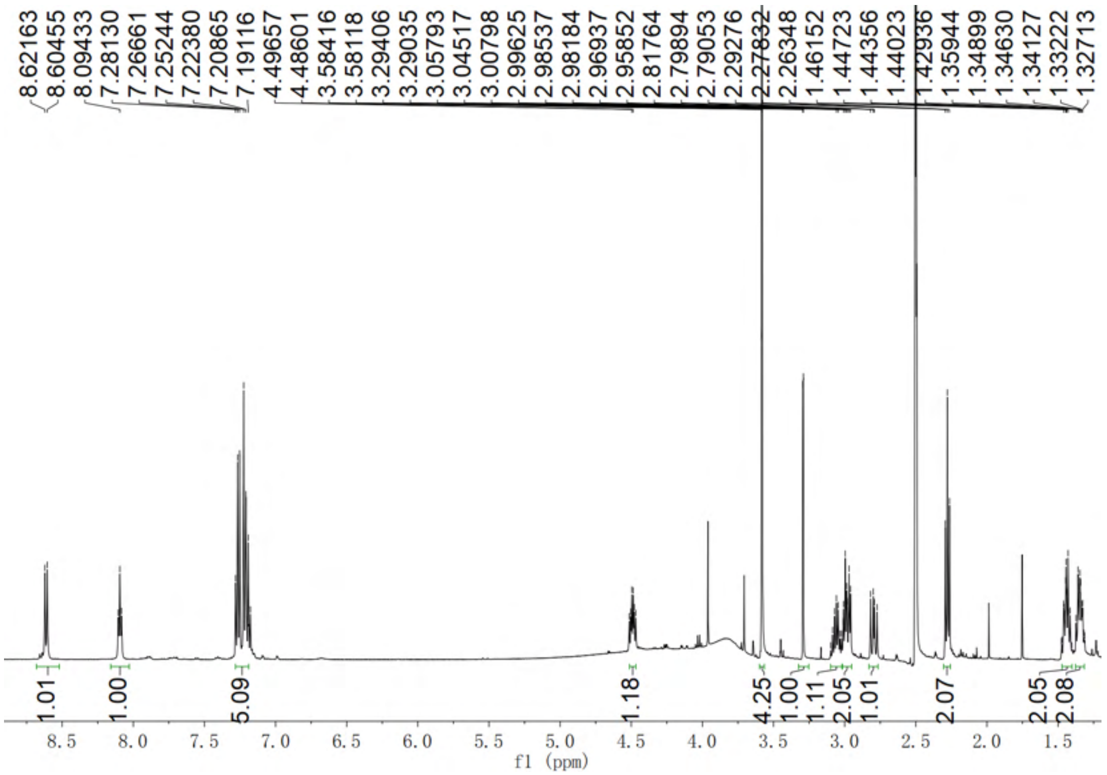

Supplementary Fig. 337. ^{1}H NMR spectrum of compound (2*S*,3*S*)-*t*-ES-Phe-b37 in DMSO-d_6

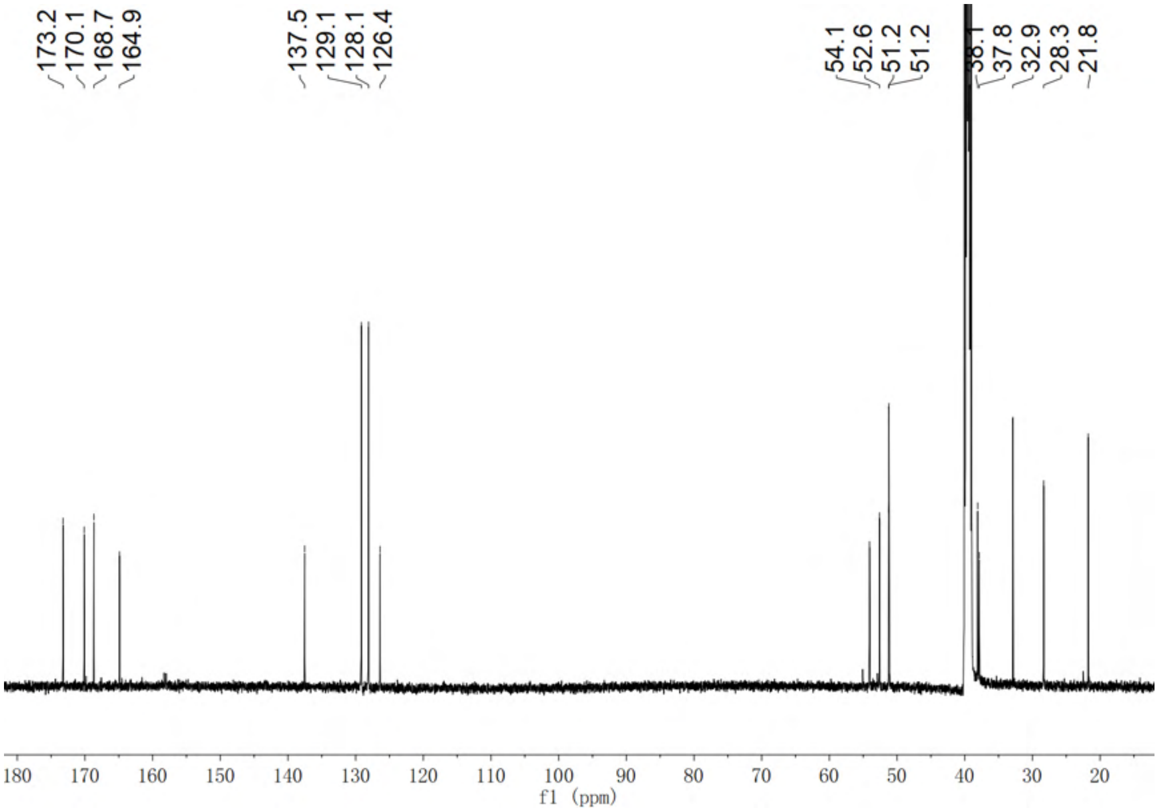

Supplementary Fig. 338. ^{13}C NMR spectrum of compound (2*S*,3*S*)-*t*-ES-Phe-b37 in DMSO-d_6

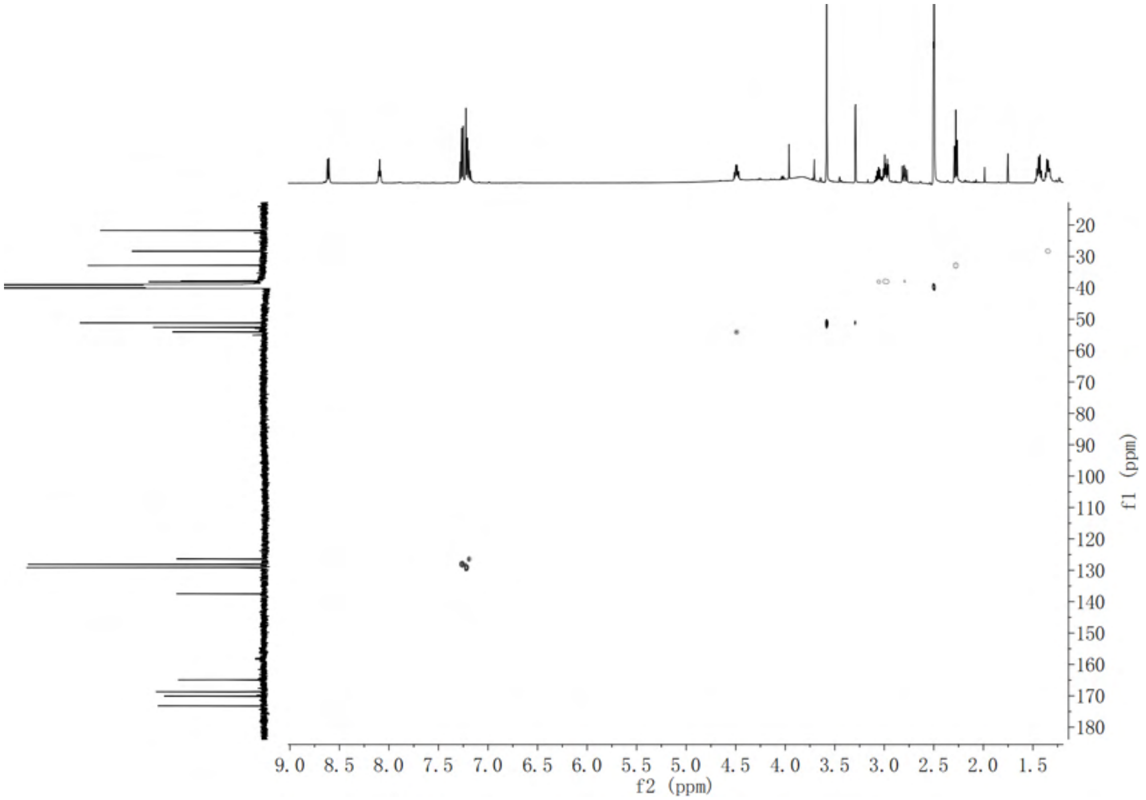

Supplementary Fig. 339. HSQC spectrum of compound (2*S*,3*S*)-*t*-ES-Phe-b37 in DMSO-d_6

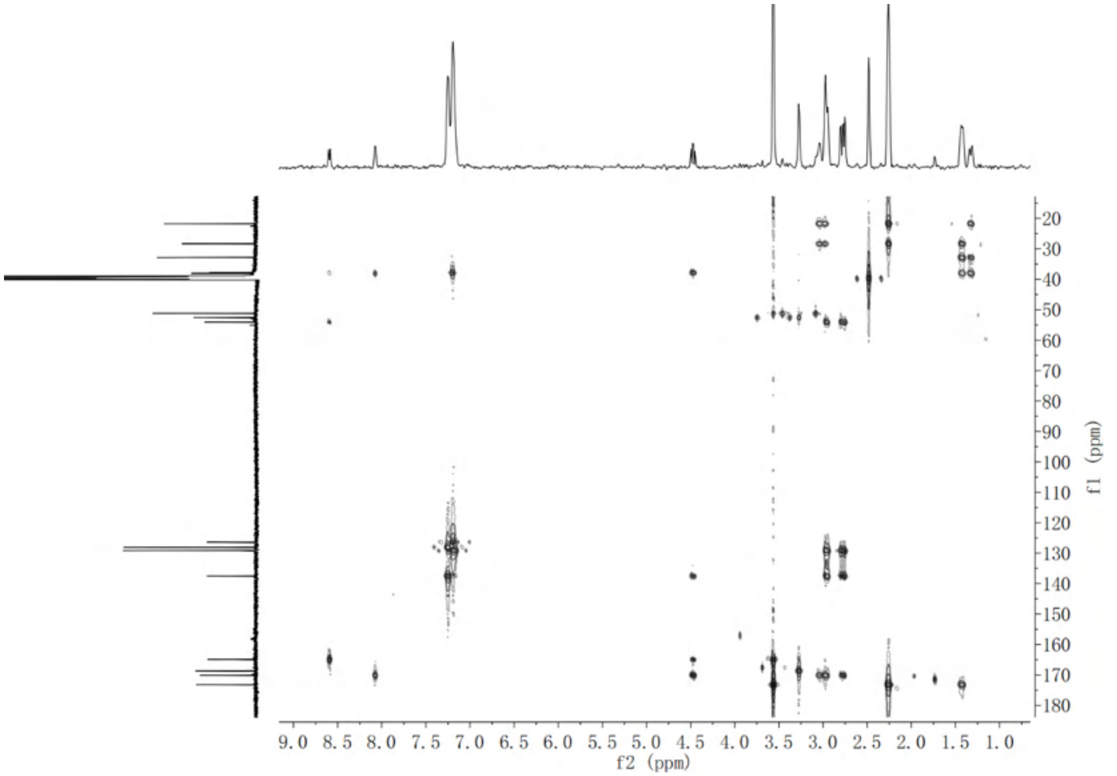

Supplementary Fig. 340. HMBC spectrum of compound (2*S*,3*S*)-*t*-ES-Phe-b37 in DMSO-d_6

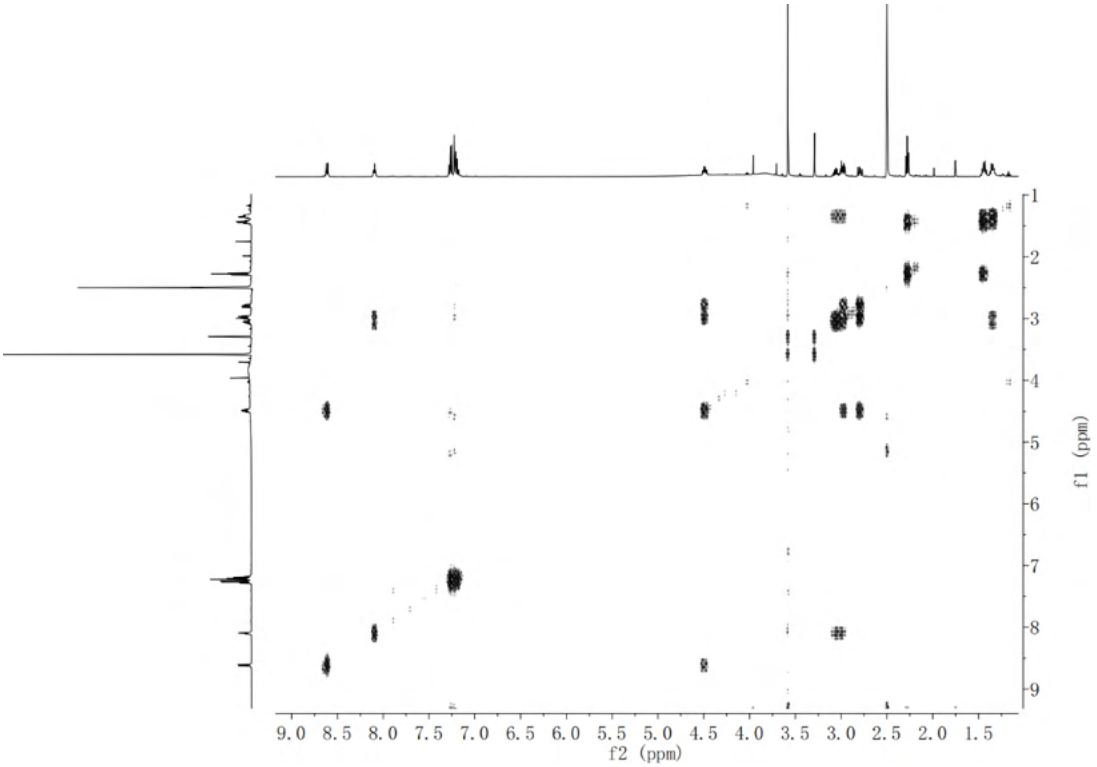

Supplementary Fig. 341. [1]**H-**[1]**H COSY spectrum of compound (2*S*,3*S*)-*t*-ES-Phe-b37 in DMSO-d_6**

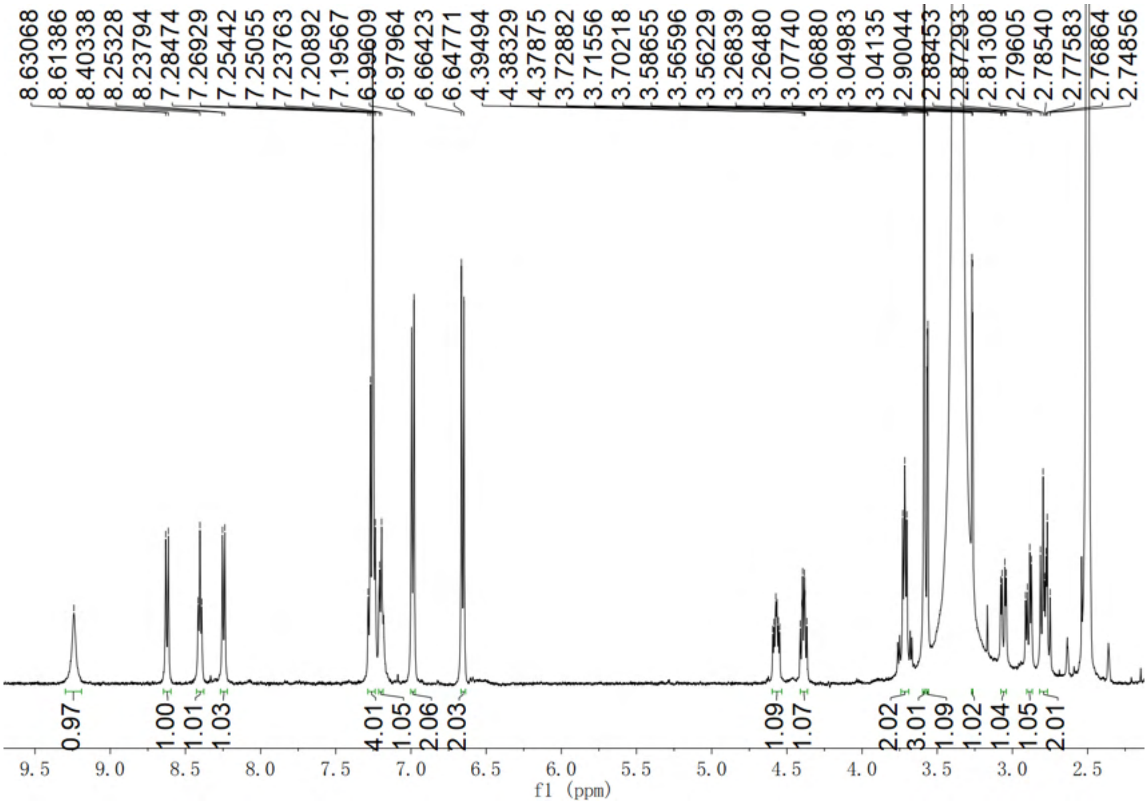

Supplementary Fig. 342. [1]**H NMR spectrum of compound (2*S*,3*S*)-*t*-ES-Phe-b38 in DMSO-d_6**

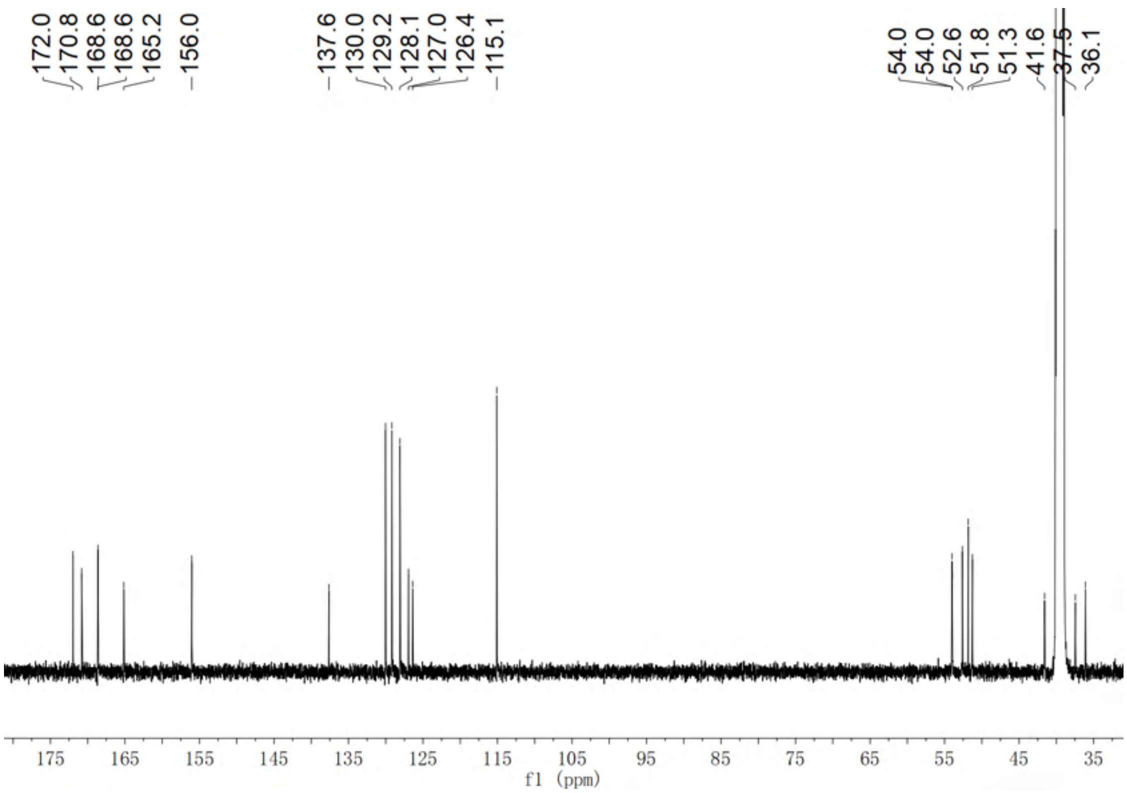

Supplementary Fig. 343. ^{13}C NMR spectrum of compound (2*S*,3*S*)-*t*-ES-Phe-b38 in DMSO-d_6

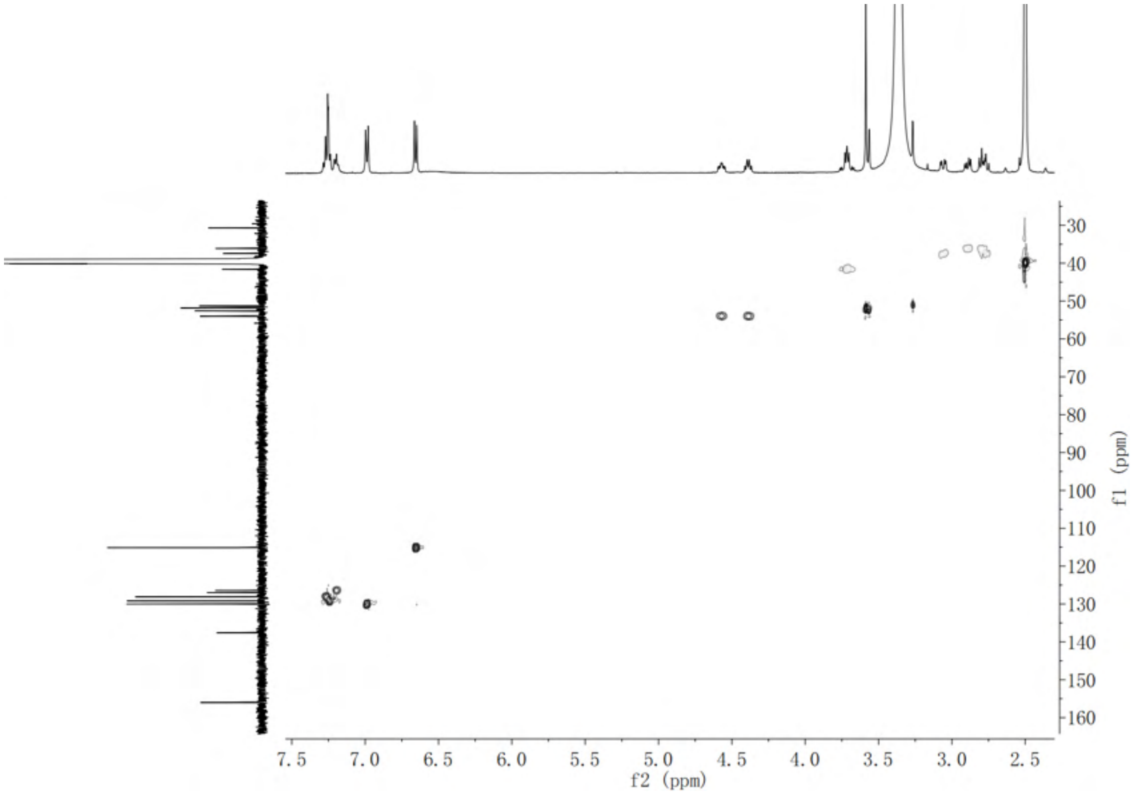

Supplementary Fig. 344. HSQC spectrum of compound (2*S*,3*S*)-*t*-ES-Phe-b38 in DMSO-d_6

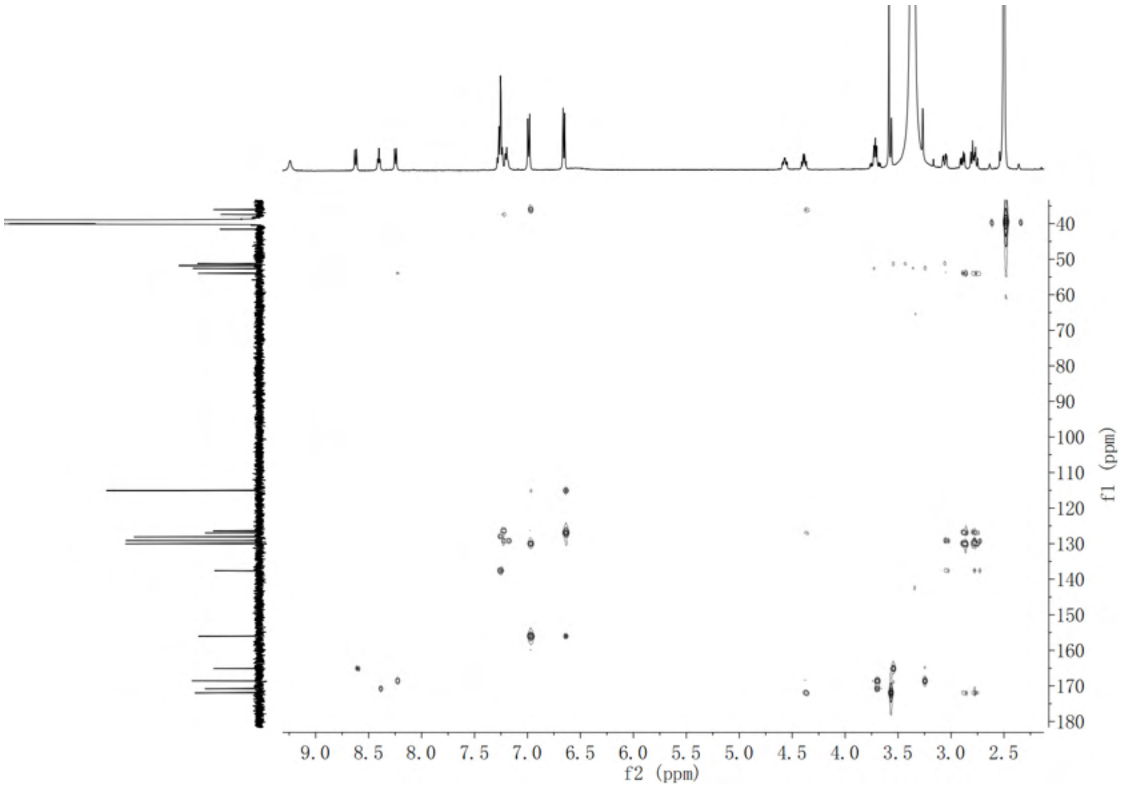

Supplementary Fig. 345. HMBC spectrum of compound (2*S*,3*S*)-*t*-ES-Phe-b38 in DMSO-d_6

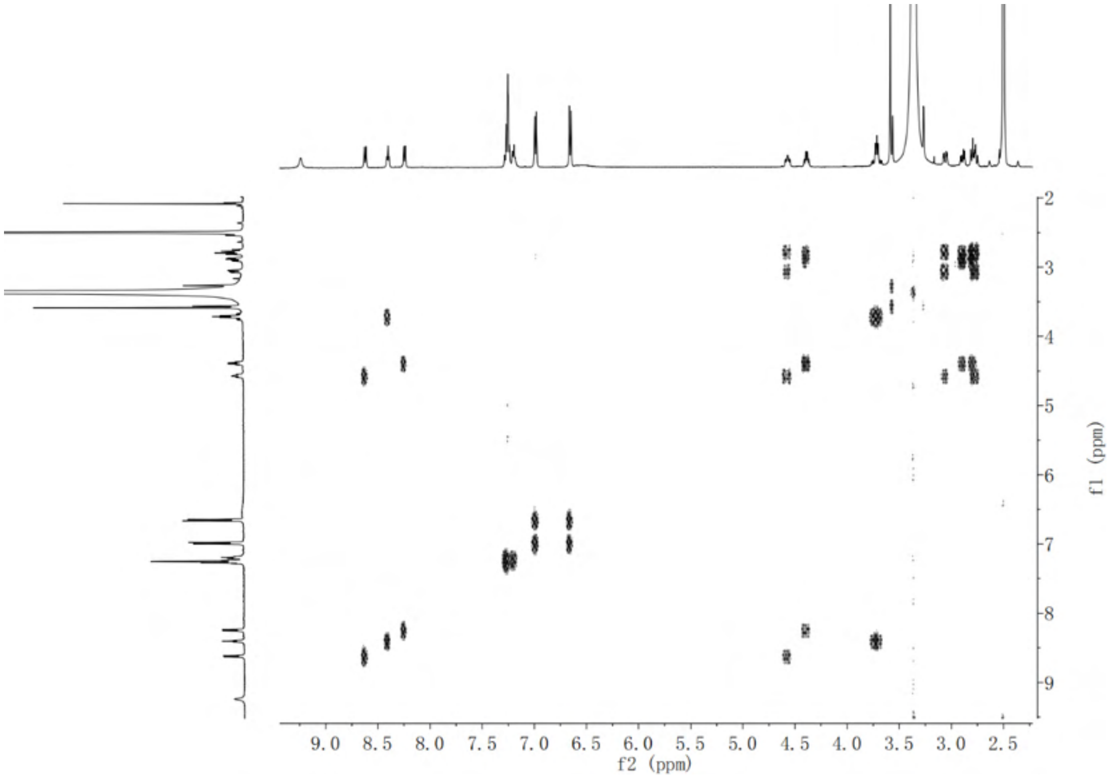

Supplementary Fig. 346. ^{1}H-^{1}H COSY spectrum of compound (2*S*,3*S*)-*t*-ES-Phe-b38 in DMSO-d_6

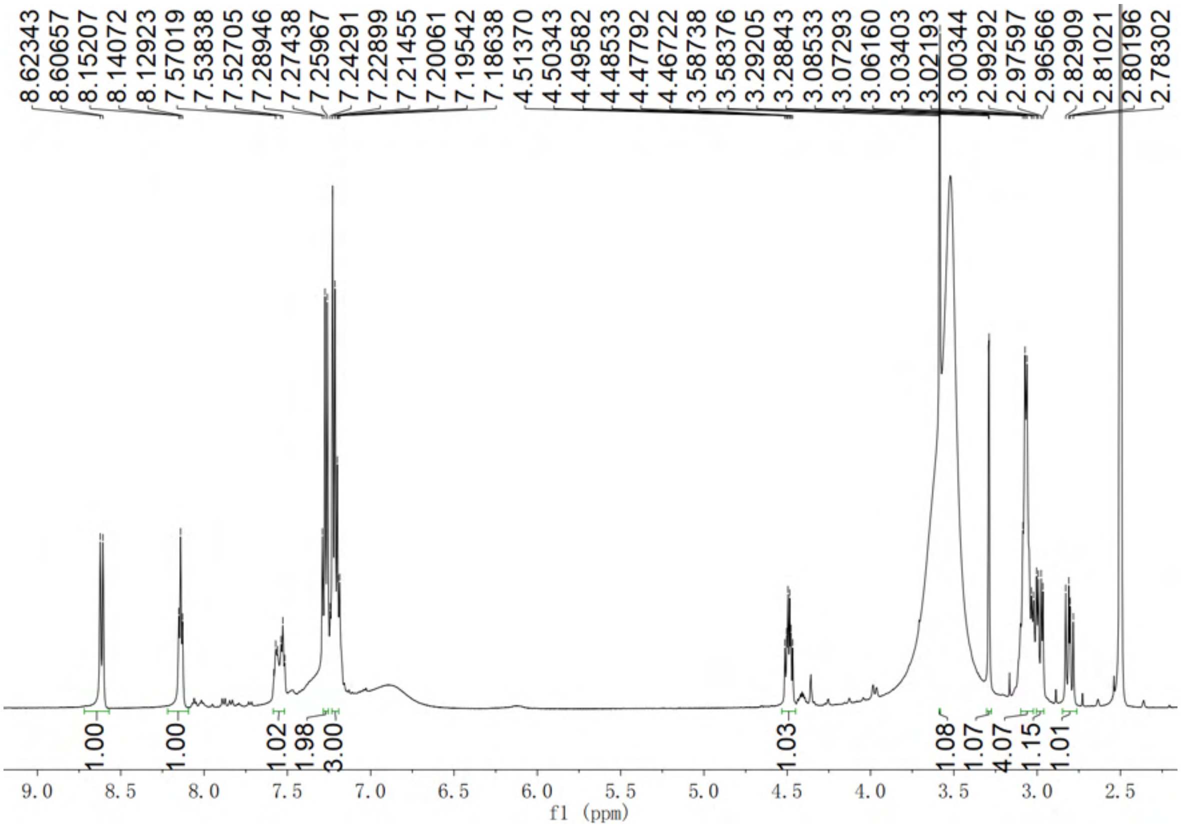

Supplementary Fig. 347. ^{1}H NMR spectrum of compound (2*S*,3*S*)-*t*-ES-Phe-b41 in DMSO-d_6

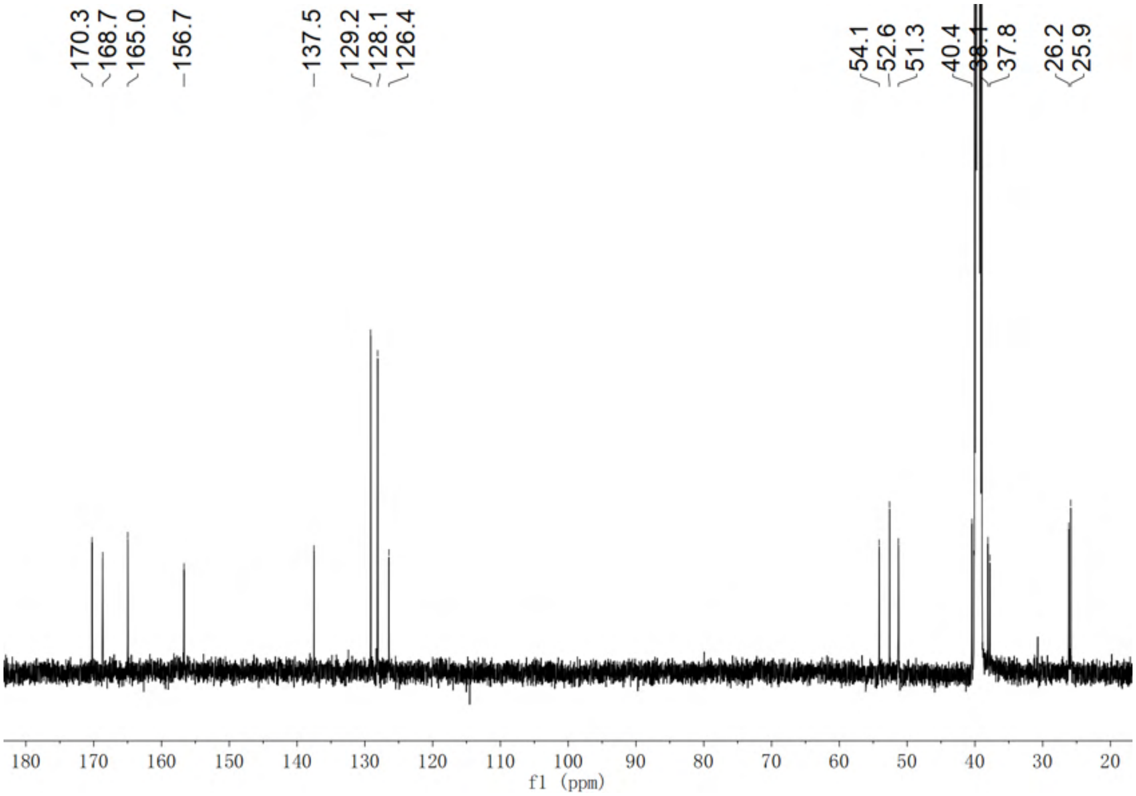

Supplementary Fig. 348. ^{13}C NMR spectrum of compound (2*S*,3*S*)-*t*-ES-Phe-b41 in DMSO-d_6

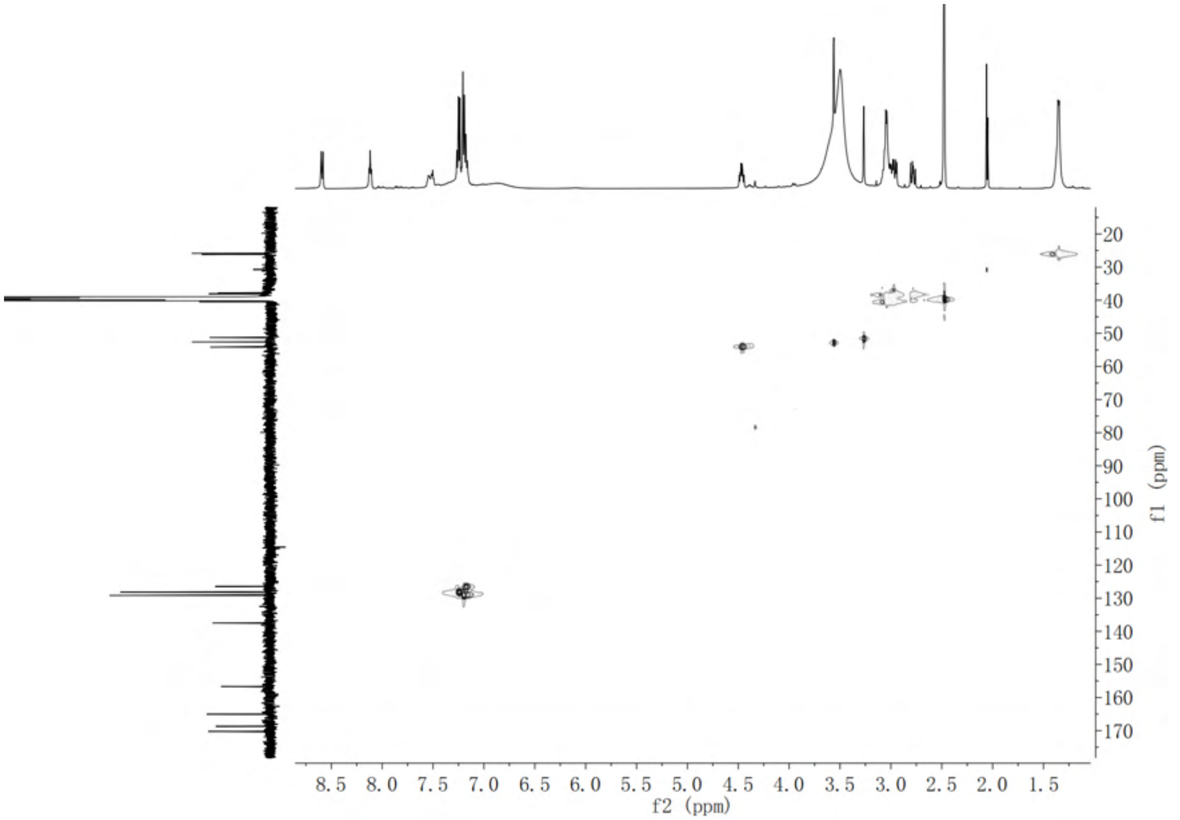

Supplementary Fig. 349. HSQC spectrum of compound (2*S*,3*S*)-*t*-ES-Phe-b41 in DMSO-d_6

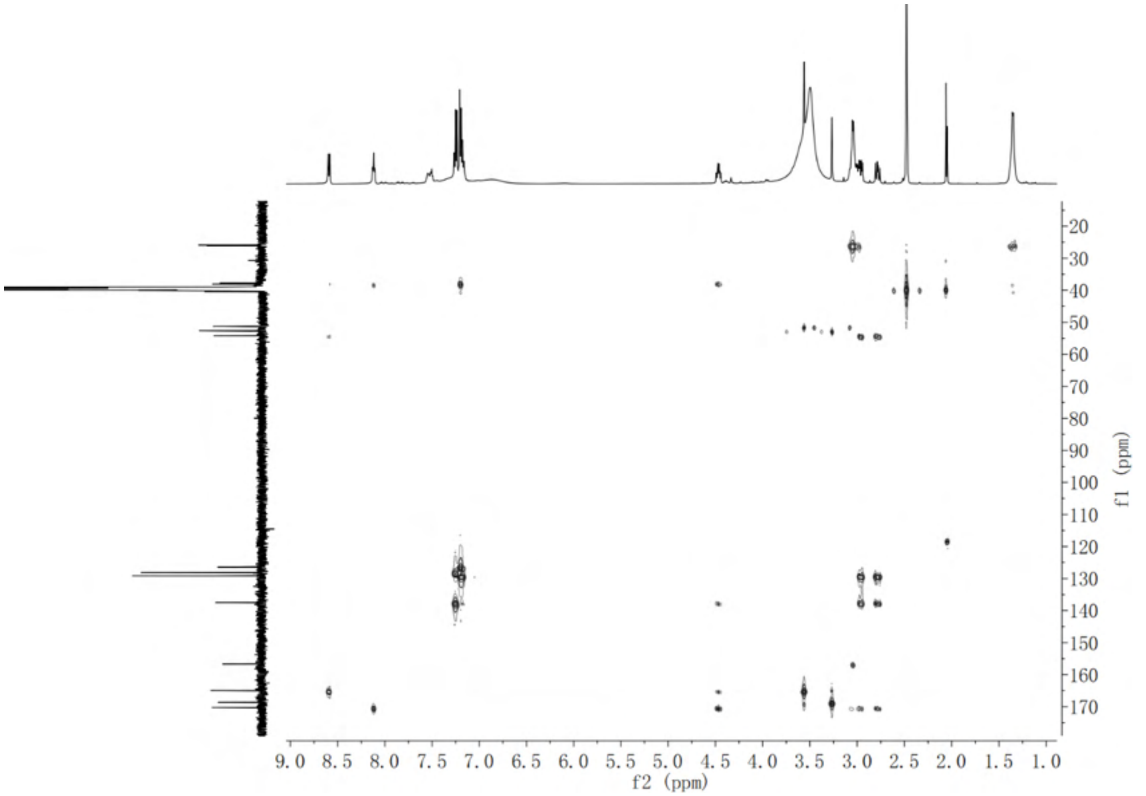

Supplementary Fig. 350. HMBC spectrum of compound (2*S*,3*S*)-*t*-ES-Phe-b41 in DMSO-d_6

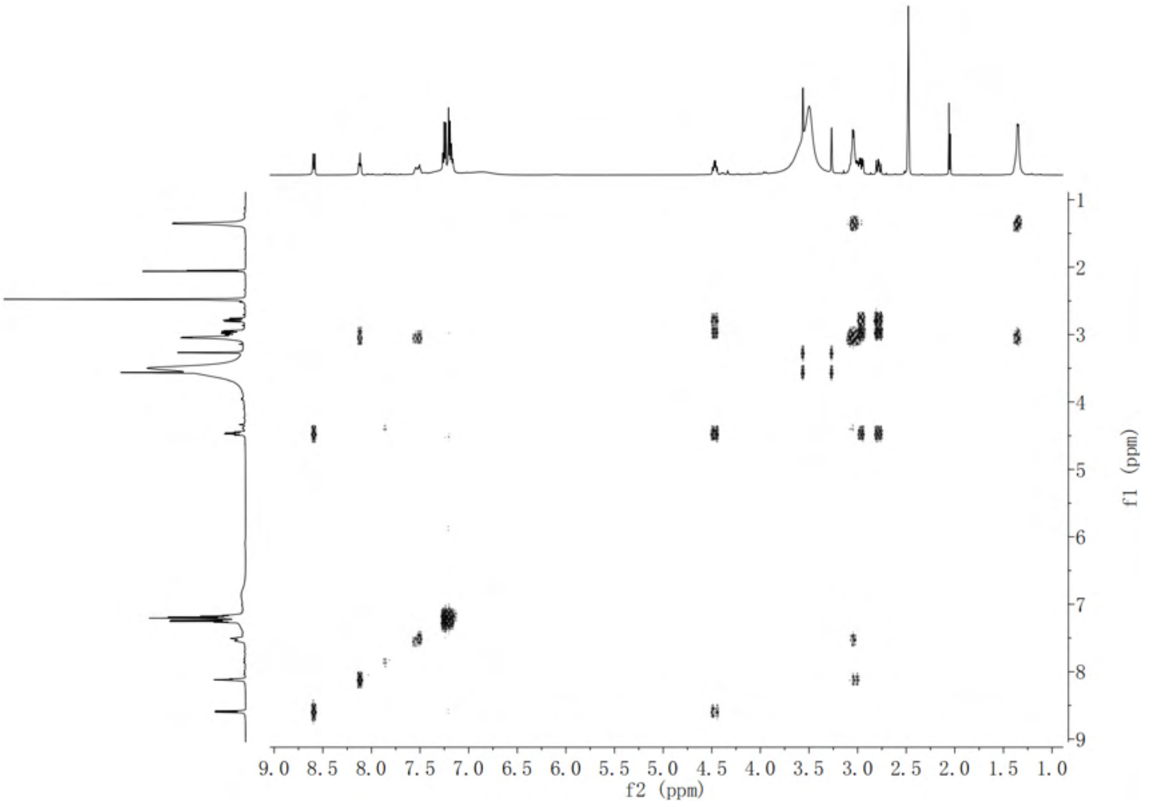

Supplementary Fig. 351. **^{1}H-^{1}H COSY spectrum of compound (2*S*,3*S*)-*t*-ES-Phe-b41 in DMSO-*d*$_6$**

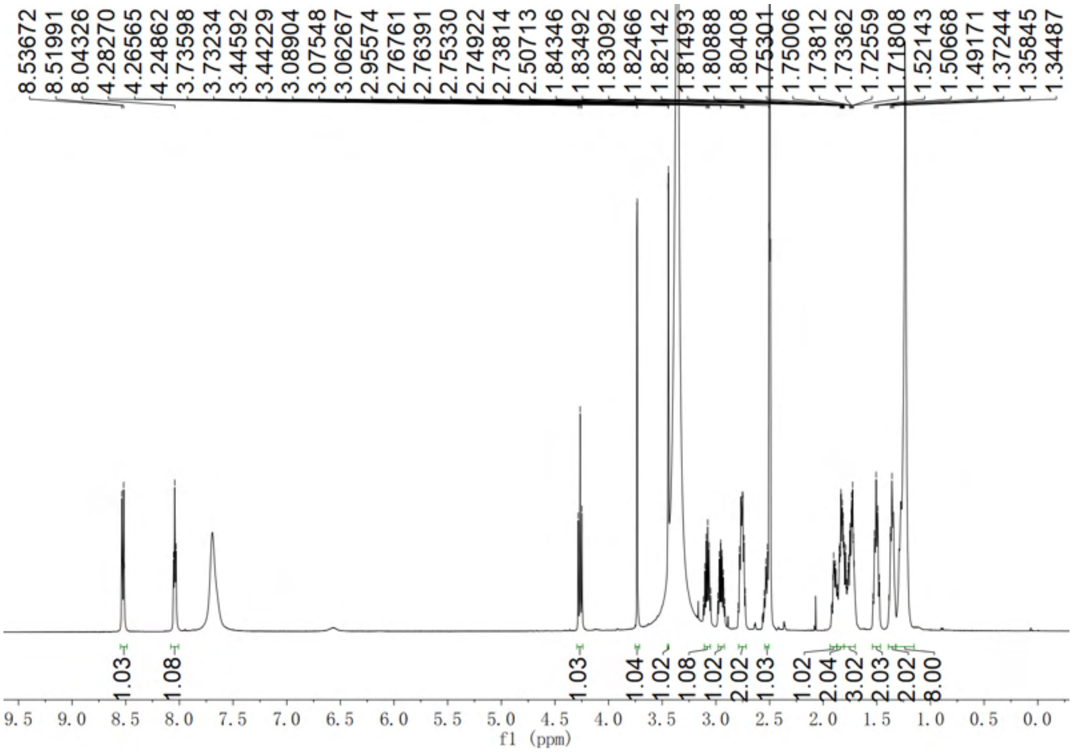

Supplementary Fig. 352. **^{1}H NMR spectrum of compound (2*S*,3*S*)-*t*-ES-a9-b7 in DMSO-*d*$_6$**

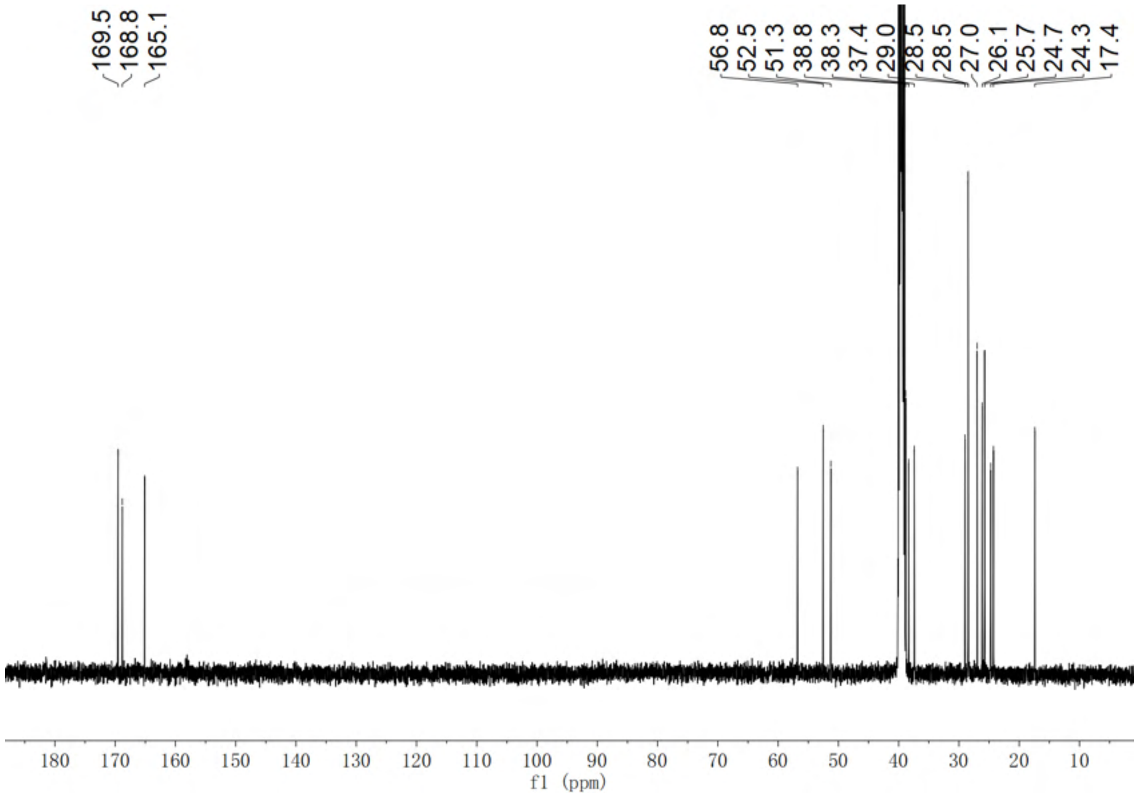

Supplementary Fig. 353. ^{13}C NMR spectrum of compound (2*S*,3*S*)-*t*-ES-a9-b7 in DMSO-d_6

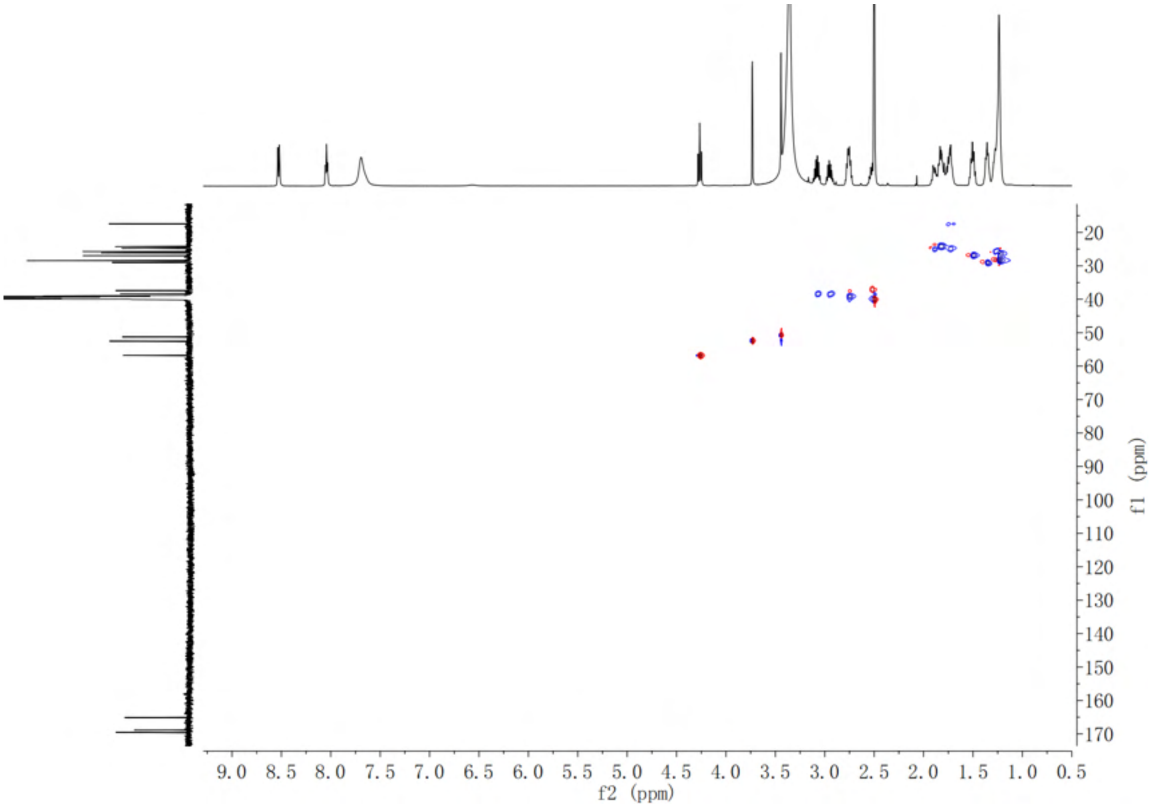

Supplementary Fig. 354. HSQC spectrum of compound (2*S*,3*S*)-*t*-ES-a9-b7 in DMSO-d_6

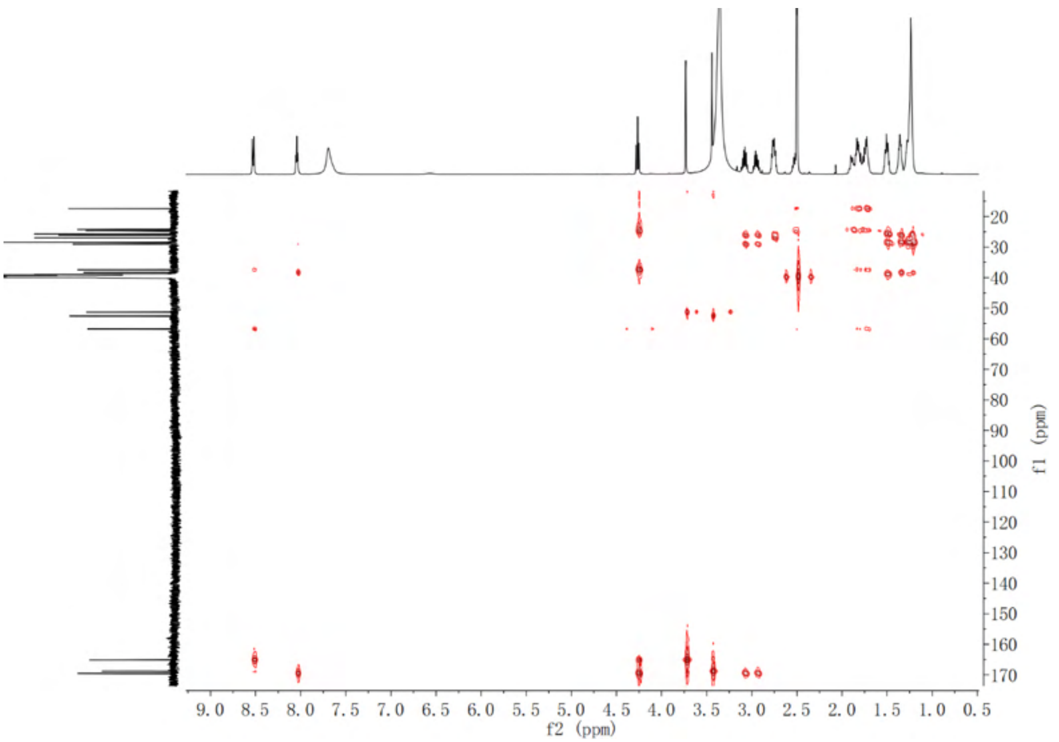

Supplementary Fig. 355. HMBC spectrum of compound (2*S*,3*S*)-*t*-ES-a9-b7 in DMSO-*d*$_6$

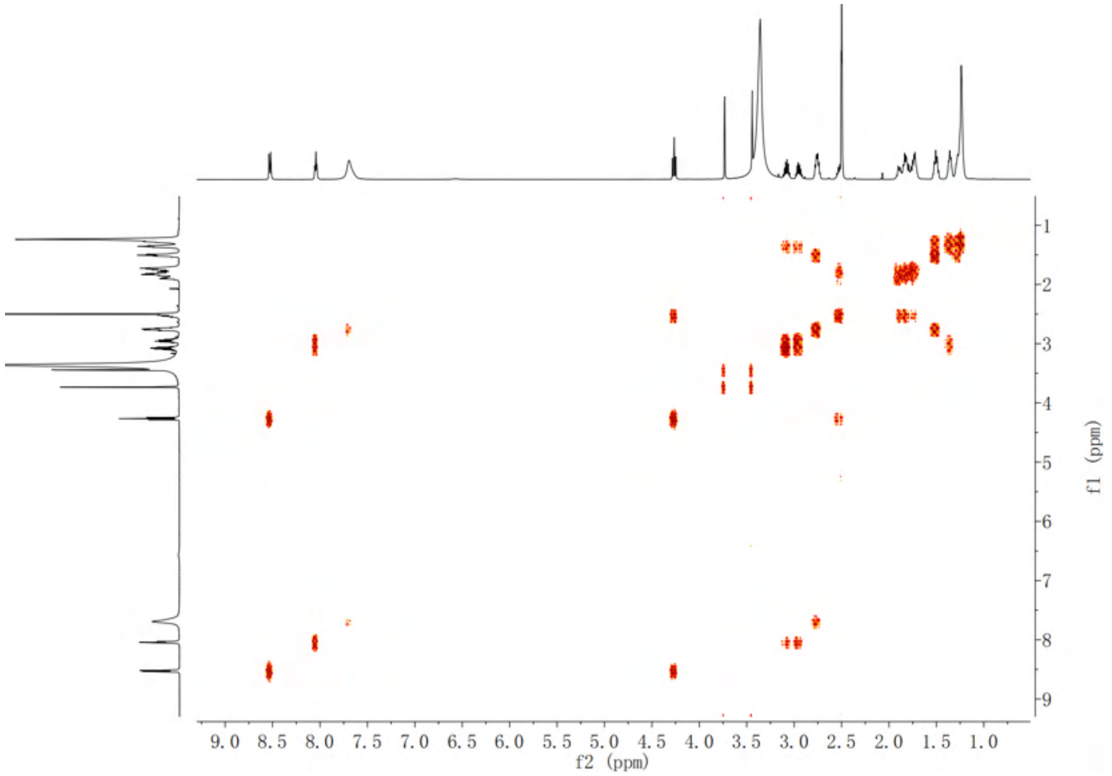

Supplementary Fig. 356. ^{1}H-^{1}H COSY spectrum of compound (2*S*,3*S*)-*t*-ES-a9-b7 in DMSO-*d*$_6$

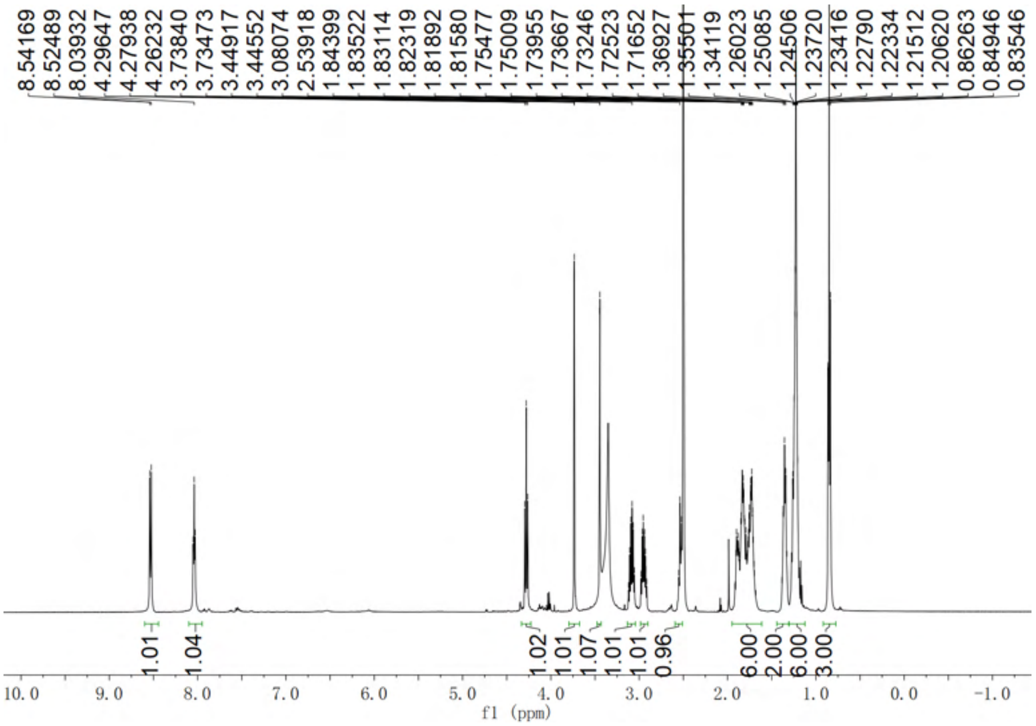

Supplementary Fig. 357. **^{1}H NMR spectrum of compound (2*S*,3*S*)-*t*-ES-a9-b13 in DMSO-*d*$_6$**

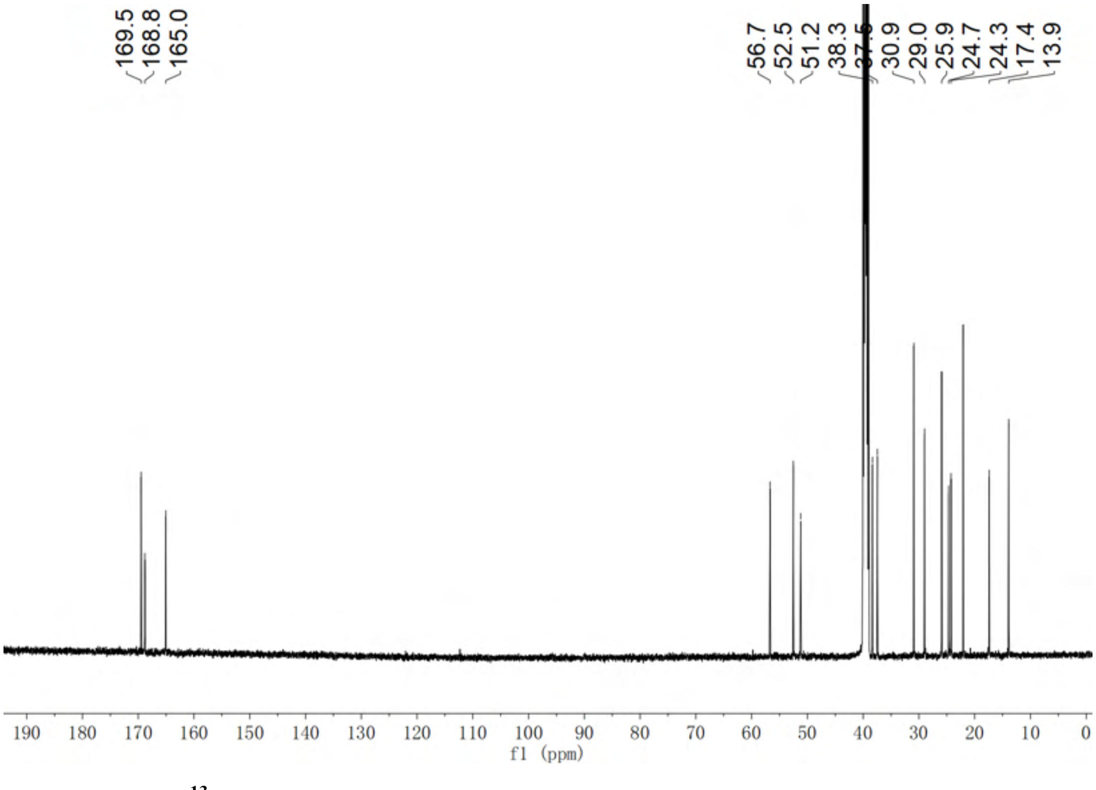

Supplementary Fig. 358. **^{13}C NMR spectrum of compound (2*S*,3*S*)-*t*-ES-a9-b13 in DMSO-*d*$_6$**

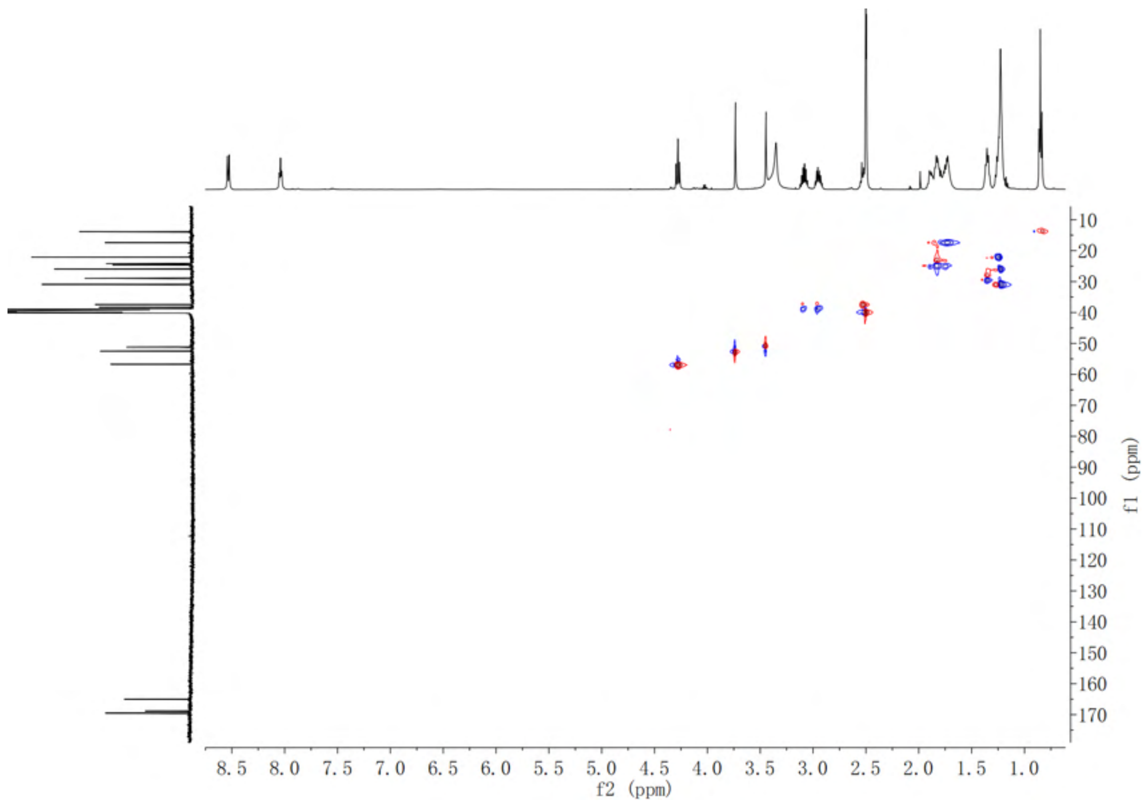

Supplementary Fig. 359. HSQC spectrum of compound (2*S*,3*S*)-*t*-ES-a9-b13 in DMSO-d_6

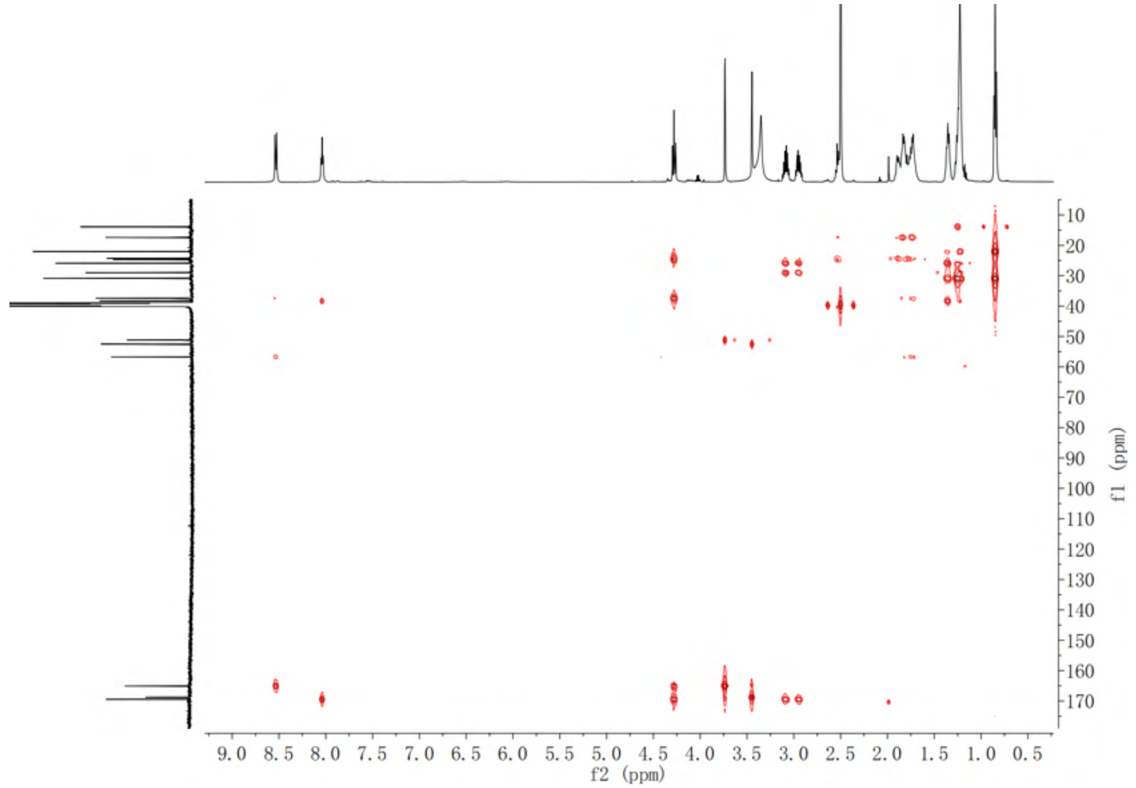

Supplementary Fig. 360. HMBC spectrum of compound (2*S*,3*S*)-*t*-ES-a9-b13 in DMSO-d_6

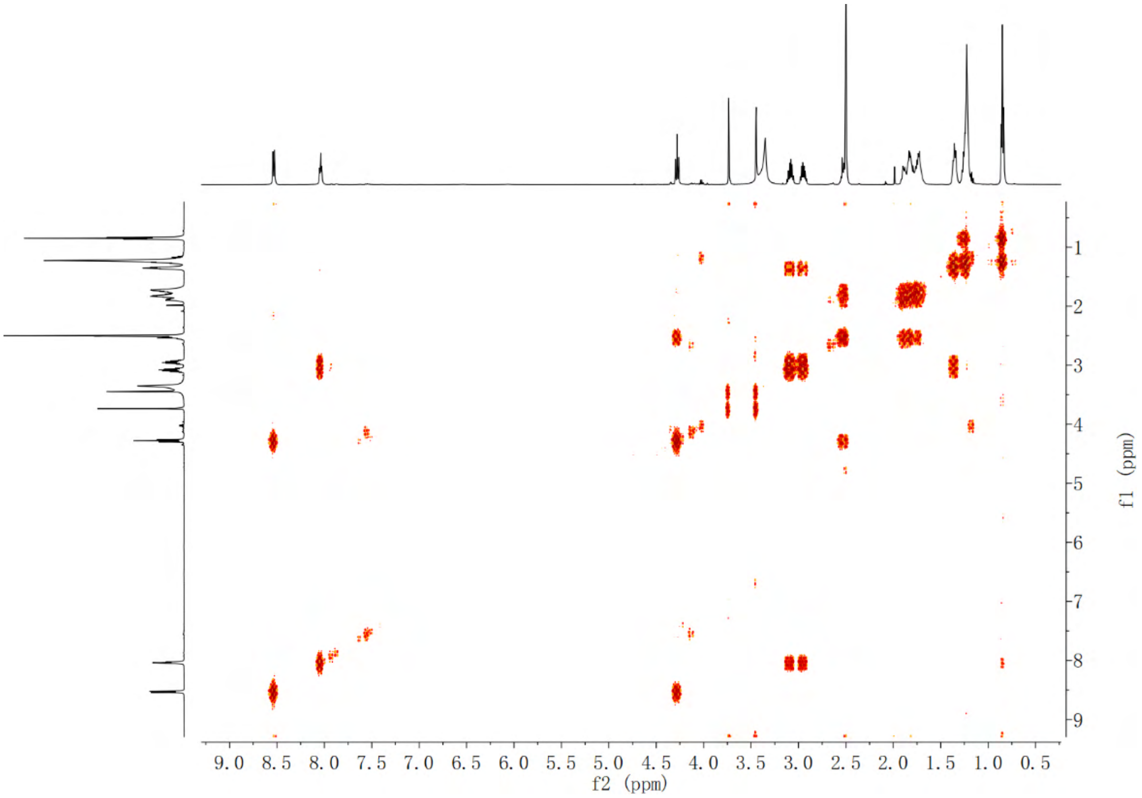

Supplementary Fig. 361. **^{1}H-^{1}H COSY spectrum of compound (2*S*,3*S*)-*t*-ES-a9-b13 in DMSO-d_6**

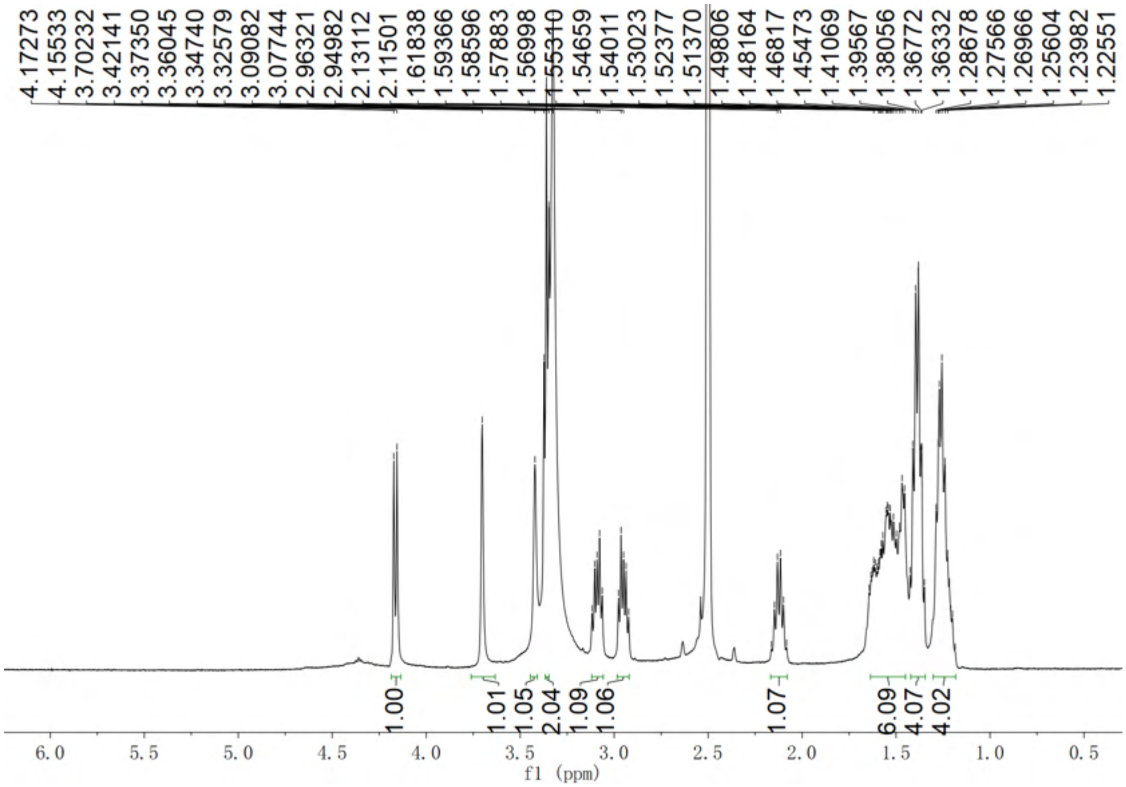

Supplementary Fig. 362. **^{1}H NMR spectrum of compound (2*S*,3*S*)-*t*-ES-a10-b9 in DMSO-d_6**

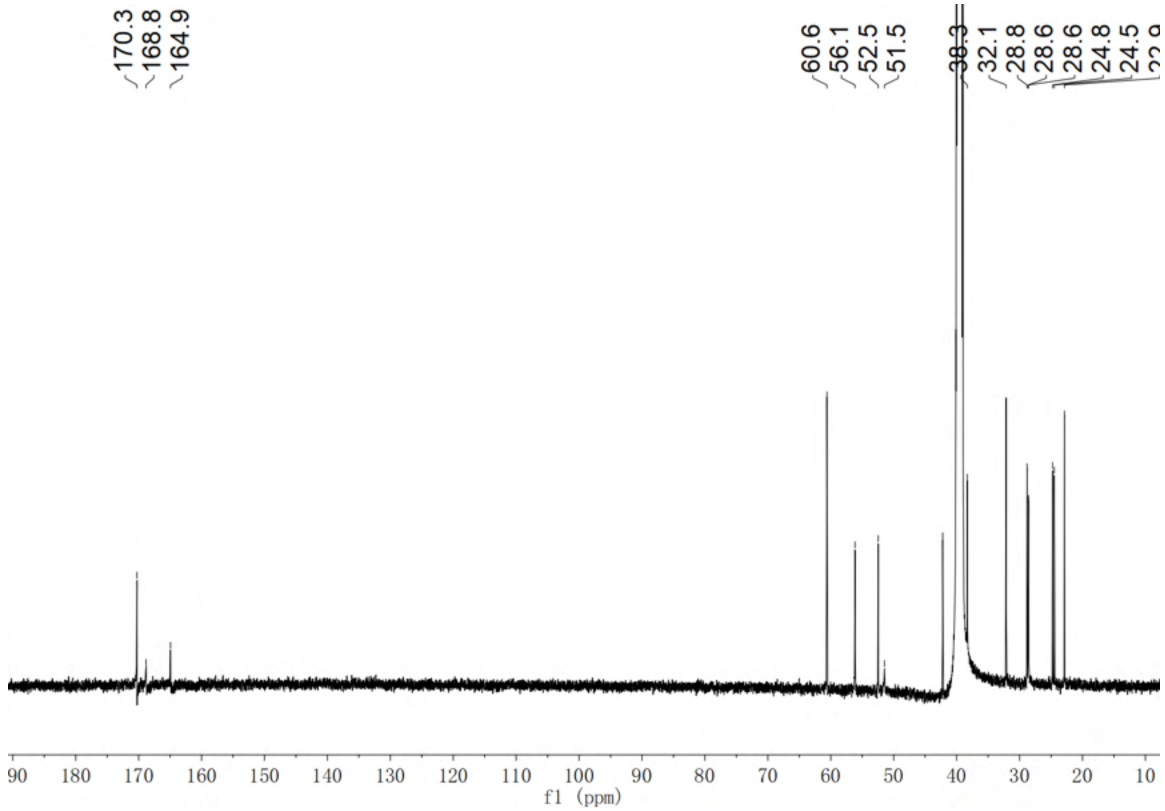

Supplementary Fig. 363. ^{13}C NMR spectrum of compound (2*S*,3*S*)-*t*-ES-a10-b9 in DMSO-d_6

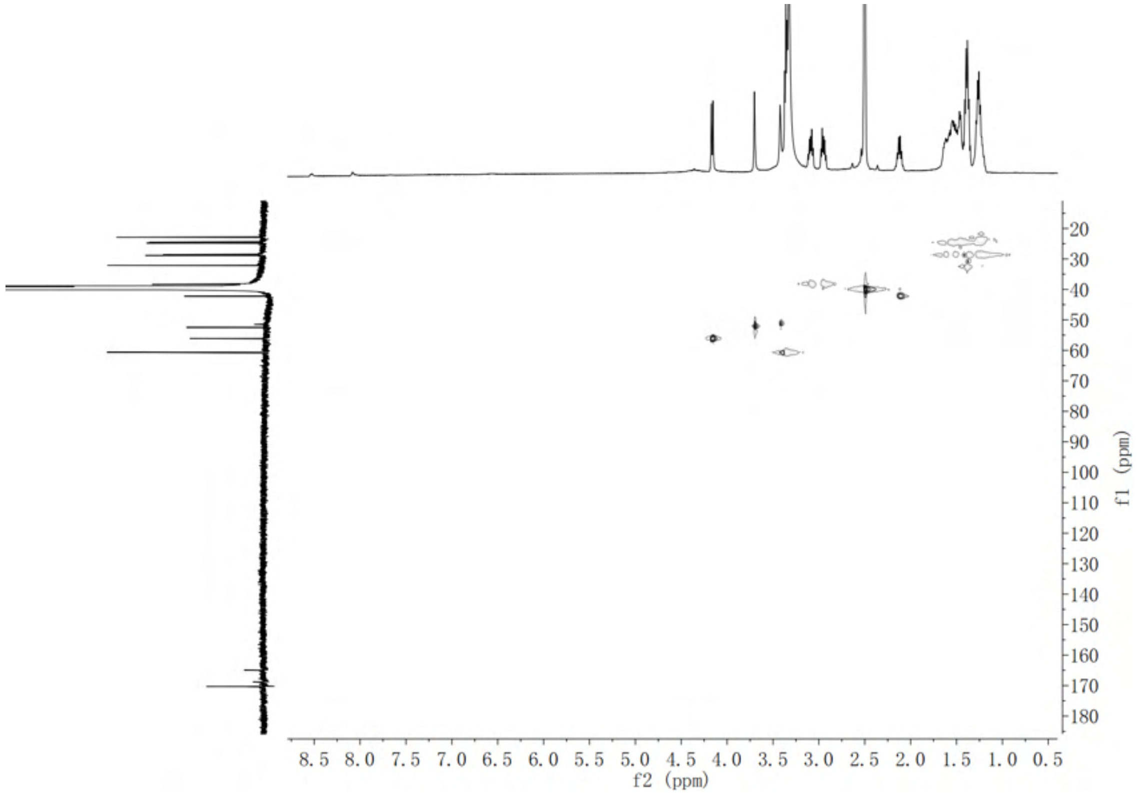

Supplementary Fig. 364. HSQC spectrum of compound (2*S*,3*S*)-*t*-ES-a10-b9 in DMSO-d_6

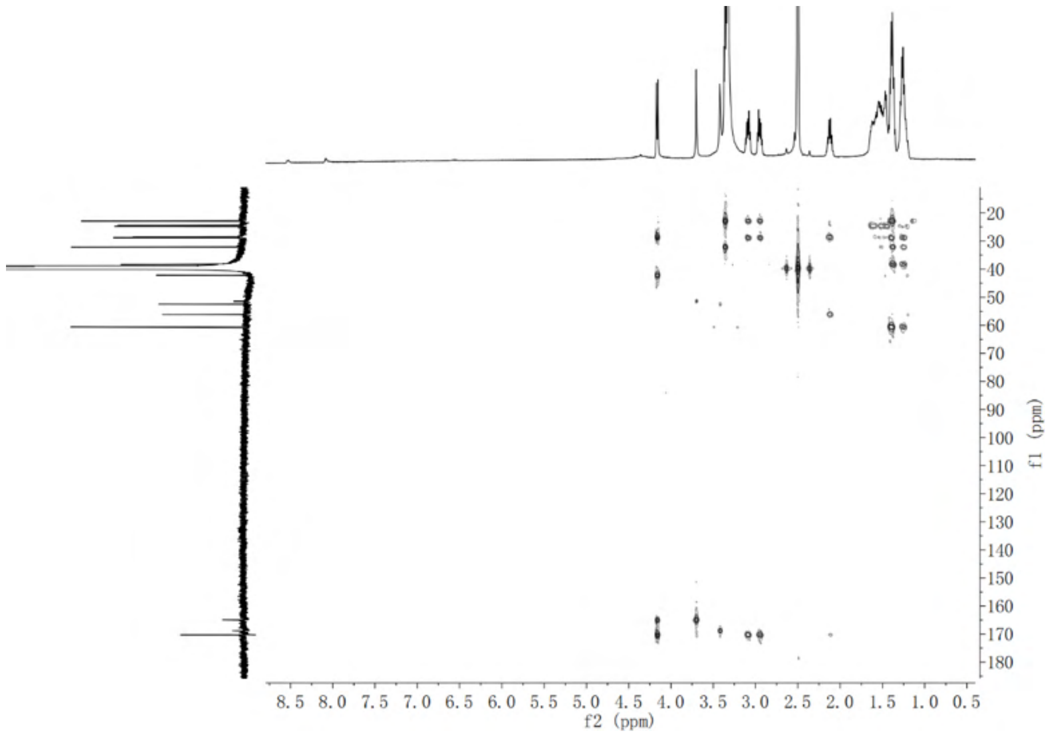

Supplementary Fig. 365. HMBC spectrum of compound (2*S*,3*S*)-*t*-ES-a10-b9 in DMSO-d_6

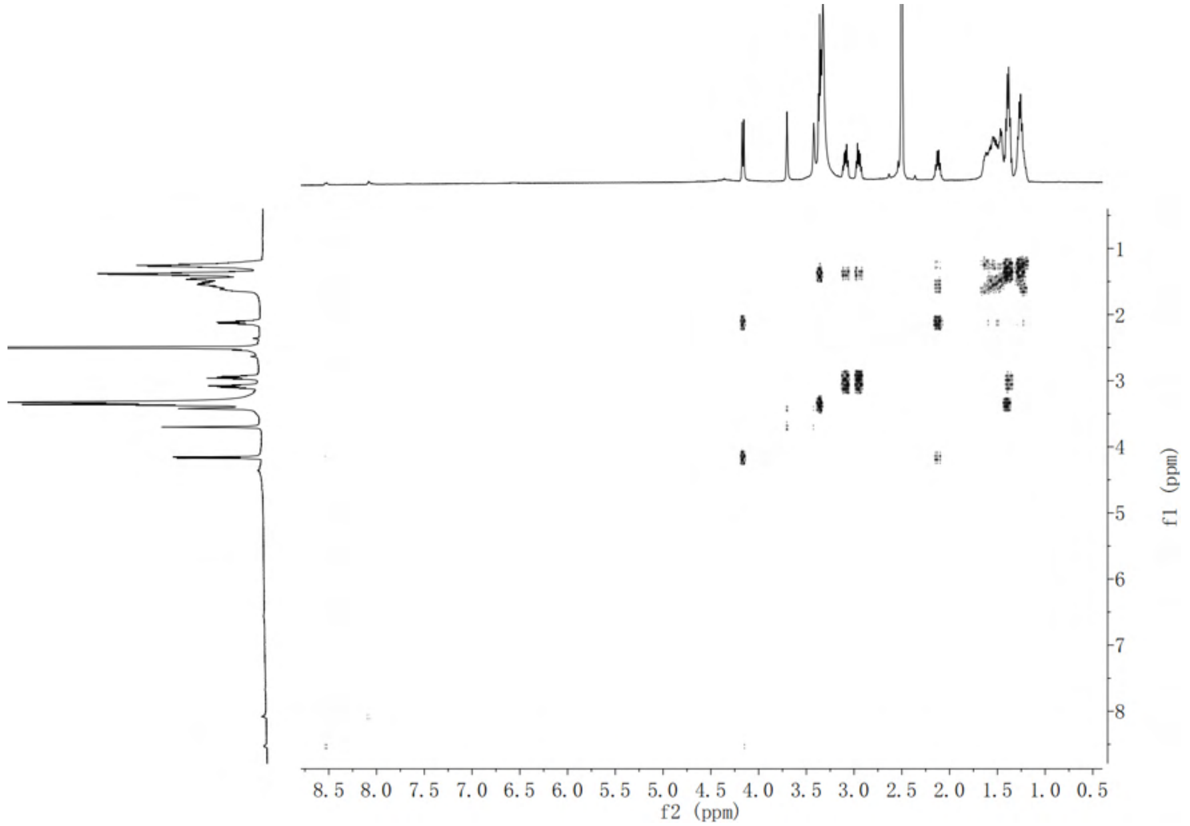

Supplementary Fig. 366. ^{1}H-^{1}H COSY spectrum of compound (2*S*,3*S*)-*t*-ES-a10-b9 in DMSO-d_6

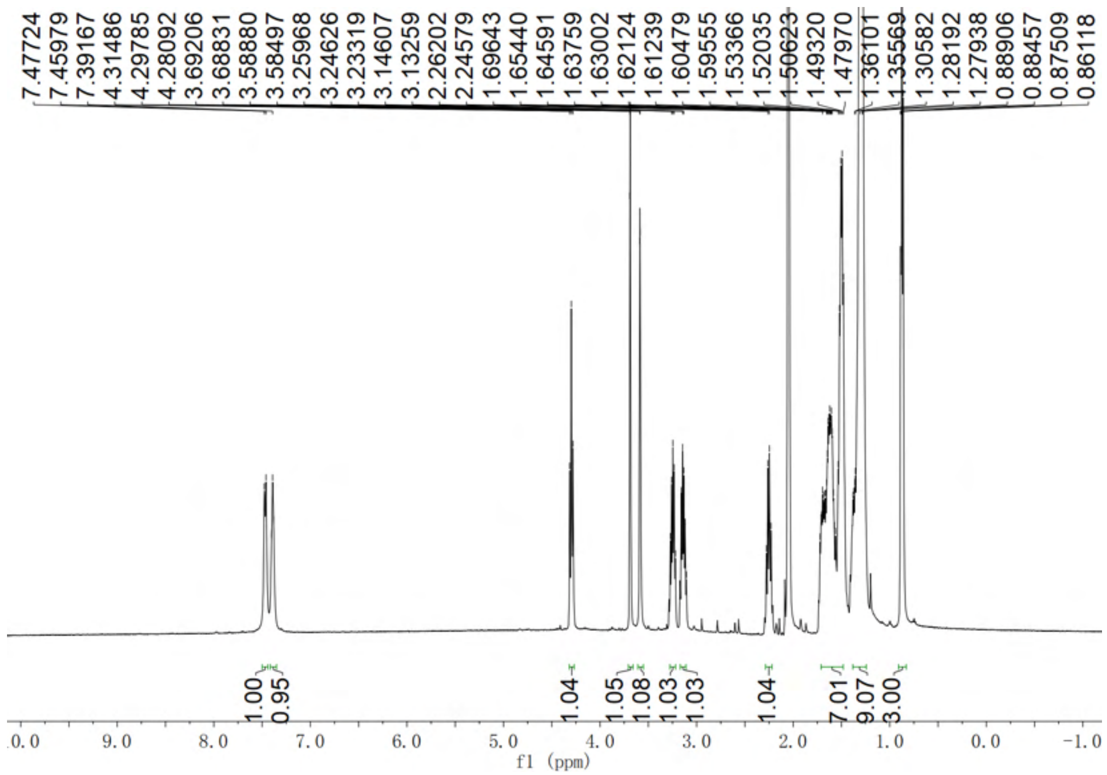

Supplementary Fig. 367. ^{1}H NMR spectrum of compound (2*S*,3*S*)-*t*-ES-a10-b14 in acetone-d_6

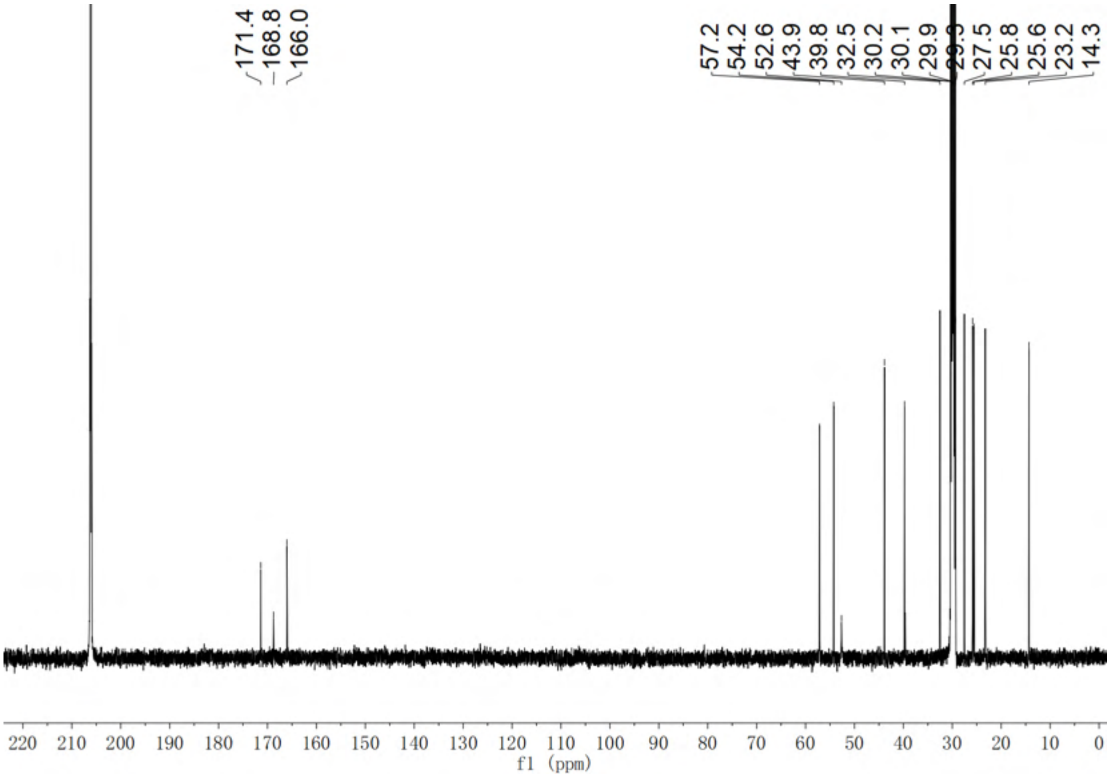

Supplementary Fig. 368. ^{13}C NMR spectrum of compound (2*S*,3*S*)-*t*-ES-a10-b14 in acetone-d_6

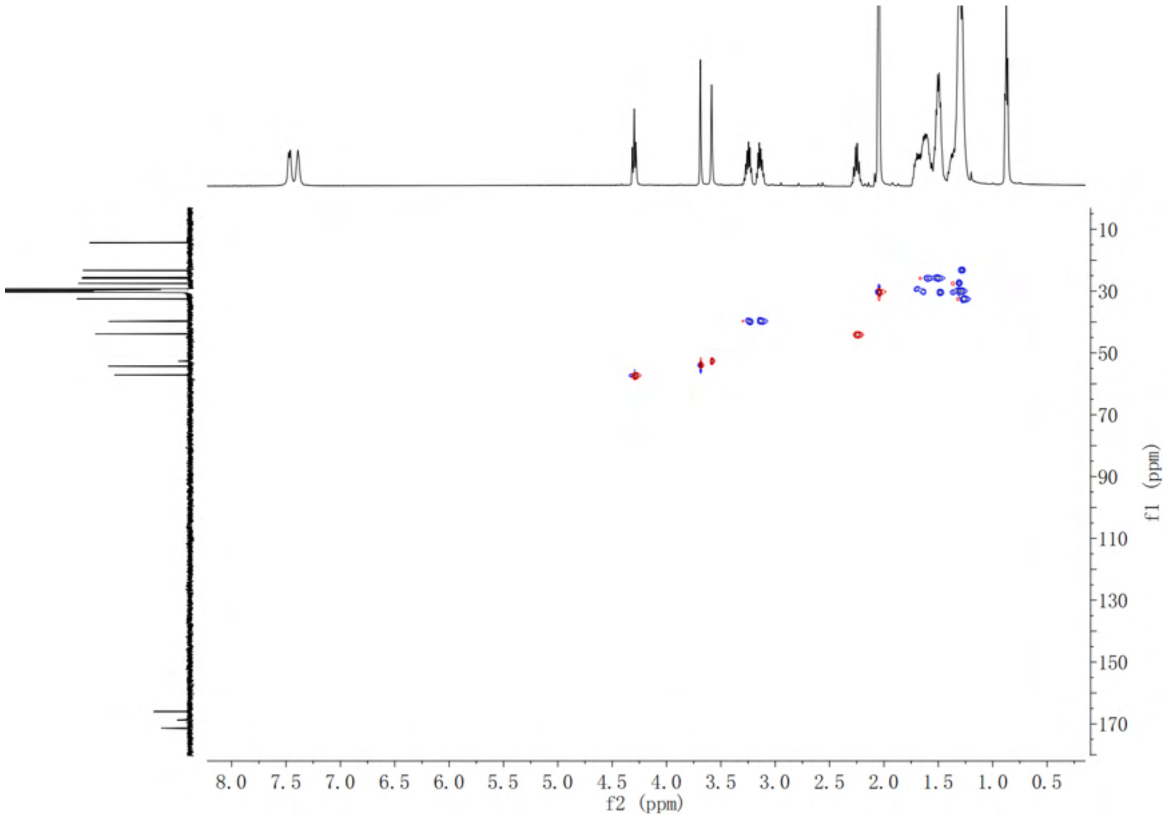

Supplementary Fig. 369. HSQC spectrum of compound (2*S*,3*S*)-*t*-ES-a10-b14 in acetone-d_6

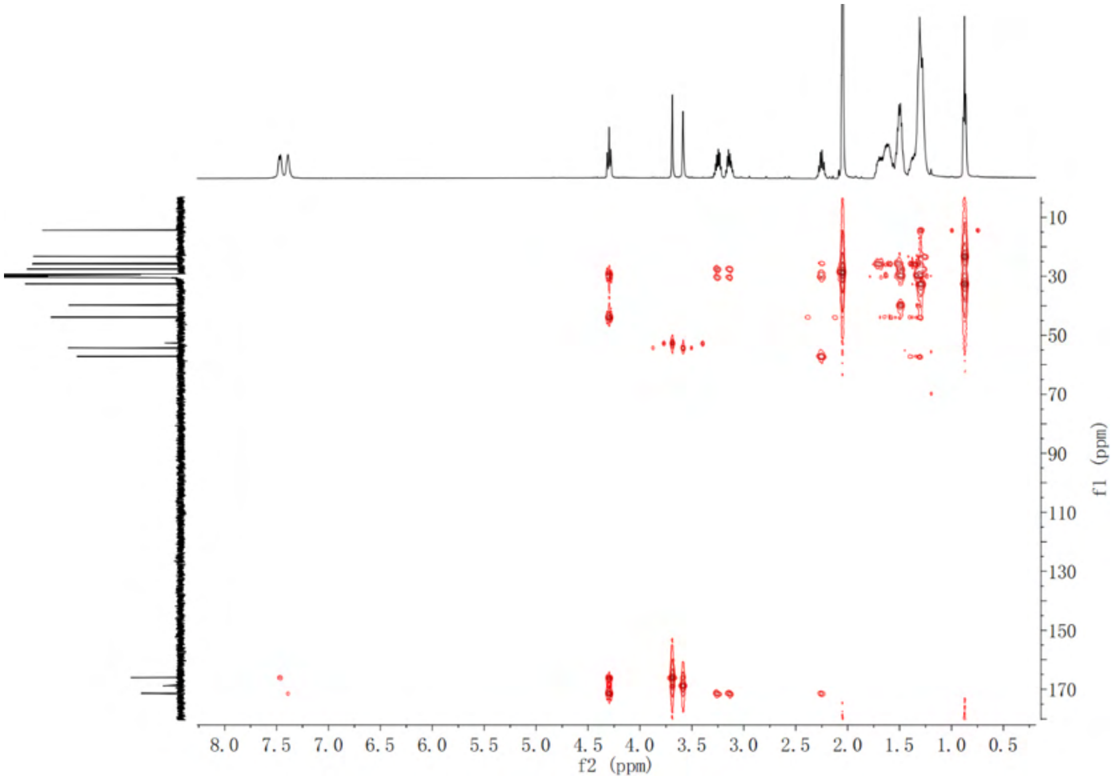

Supplementary Fig. 370. HMBC spectrum of compound (2*S*,3*S*)-*t*-ES-a10-b14 in acetone-d_6

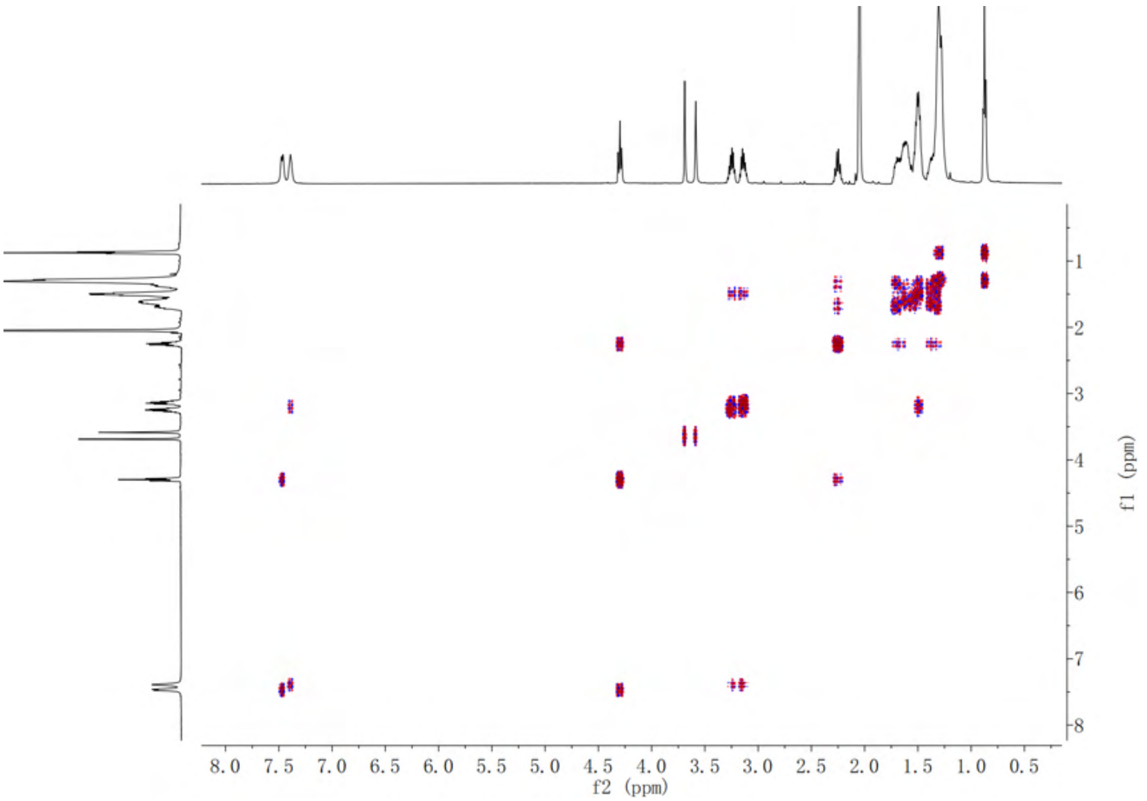

Supplementary Fig. 371. ^{1}**H-**^{1}**H COSY spectrum of compound (2*S*,3*S*)-*t*-ES-a10-b13 in acetone-*d*$_6$**

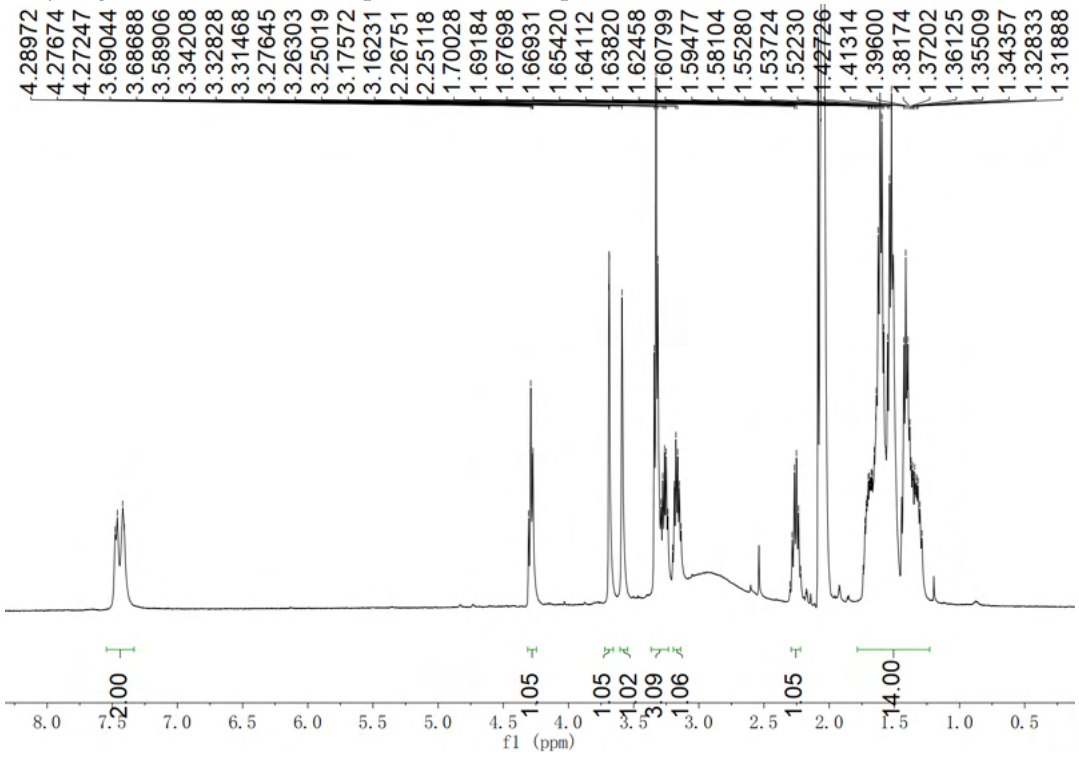

Supplementary Fig. 372. ^{1}**H NMR spectrum of compound (2*S*,3*S*)-*t*-ES-a10-b26 in acetone-*d*$_6$**

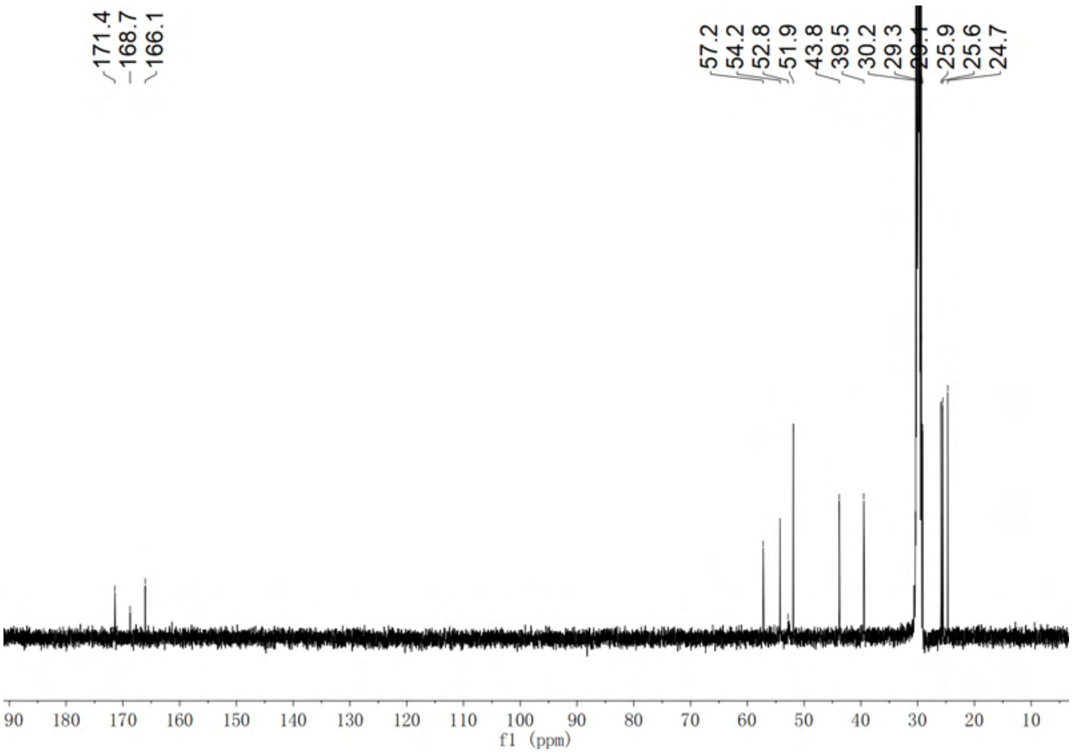

Supplementary Fig. 373. ^{13}C NMR spectrum of compound (2*S*,3*S*)-*t*-ES-a10-b26 in acetone-*d*$_6$

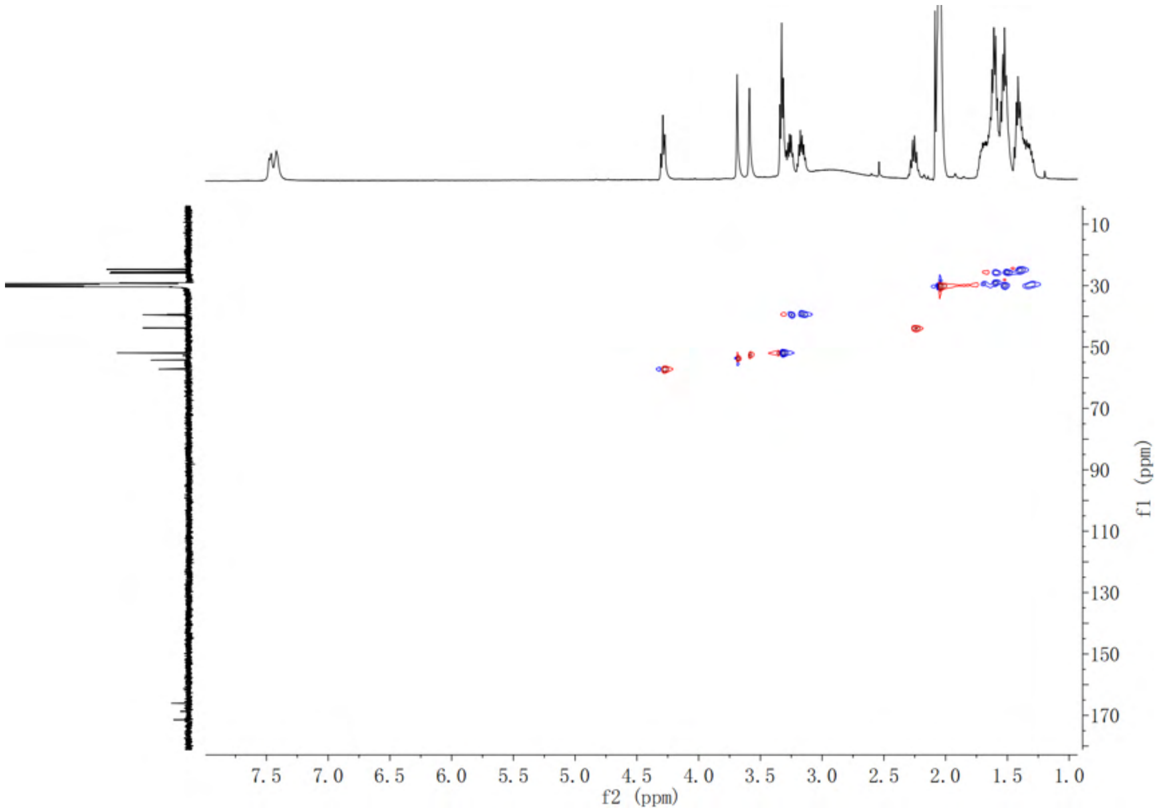

Supplementary Fig. 374. HSQC spectrum of compound (2*S*,3*S*)-*t*-ES-a10-b26 in acetone-*d*$_6$

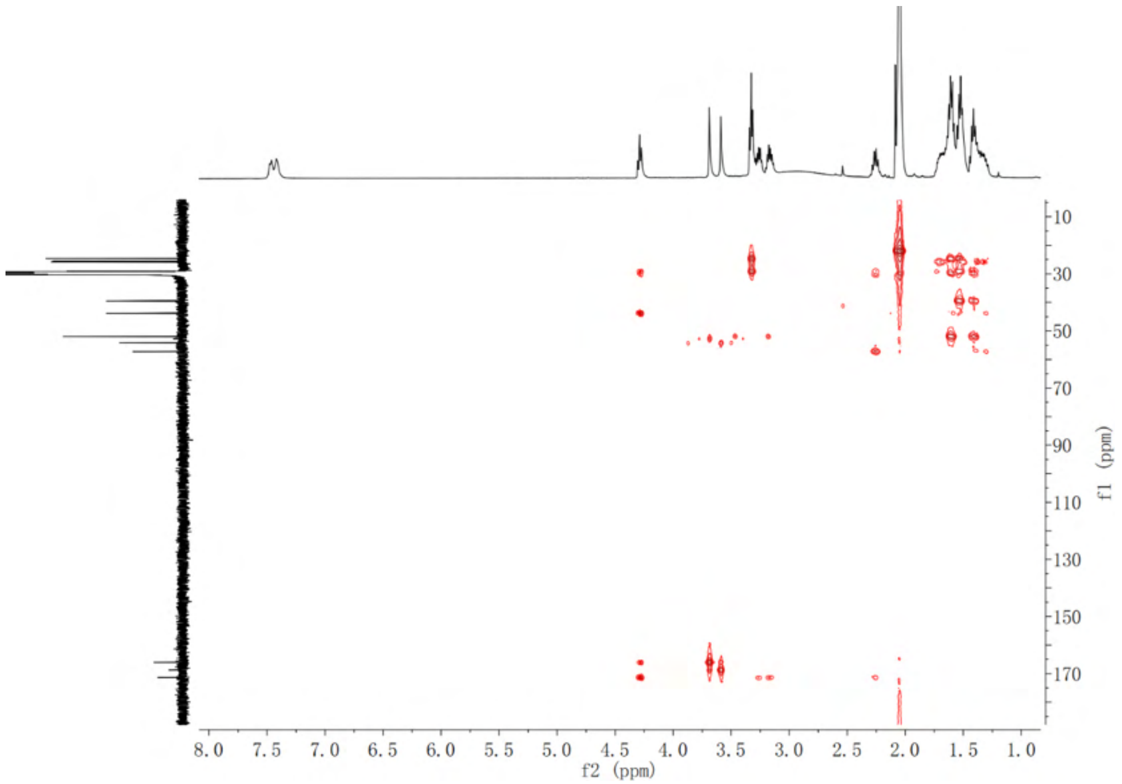

Supplementary Fig. 375. HMBC spectrum of compound (2*S*,3*S*)-*t*-ES-a10-b26 in acetone-d_6

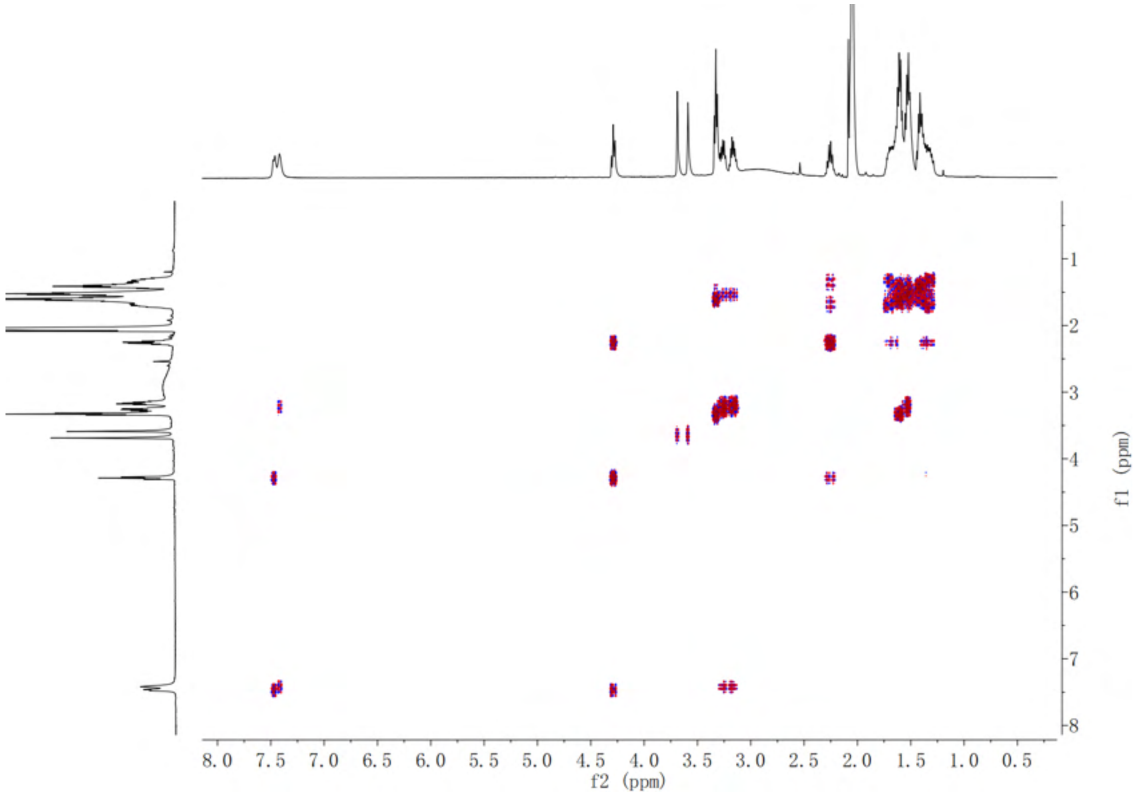

Supplementary Fig. 376. ^{1}H-^{1}H COSY spectrum of compound (2*S*,3*S*)-*t*-ES-a10-b26 in acetone-d_6

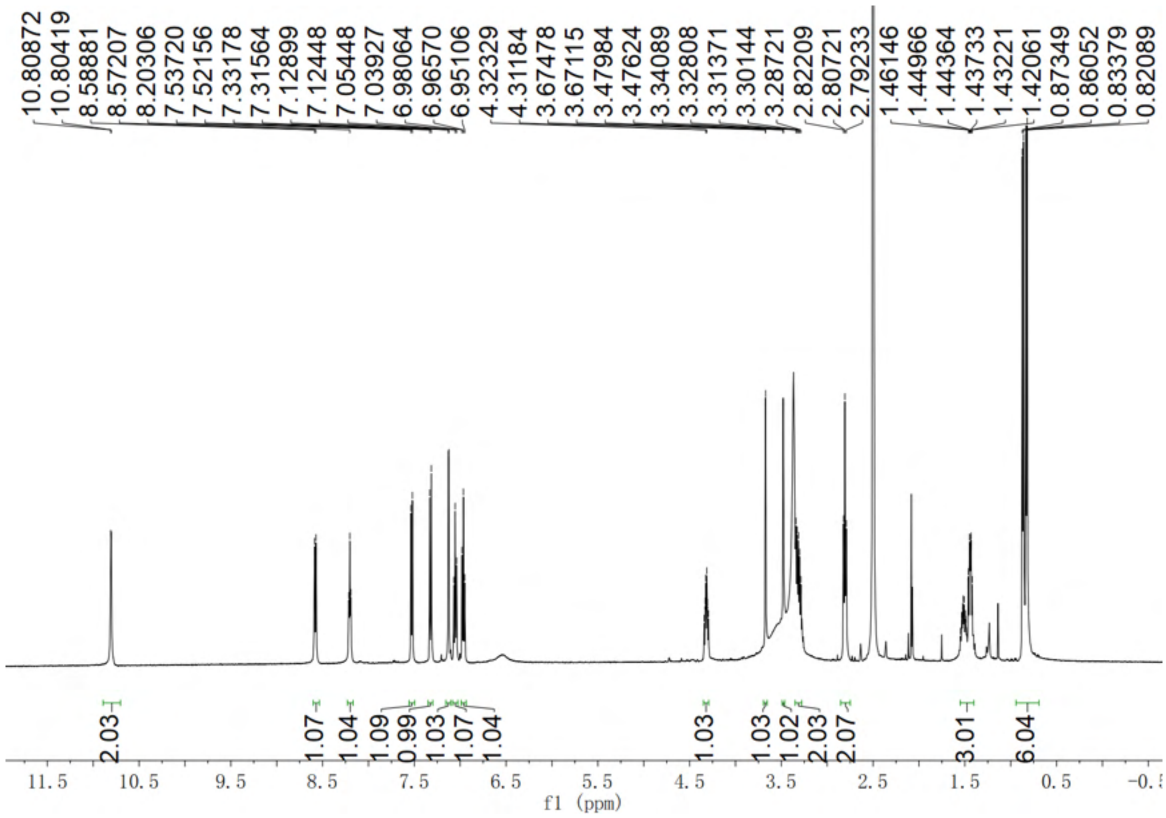

Supplementary Fig. 377. ^{1}H NMR spectrum of compound (2*S*,3*S*)-*t*-ES-Leu-b43 in DMSO-d_6

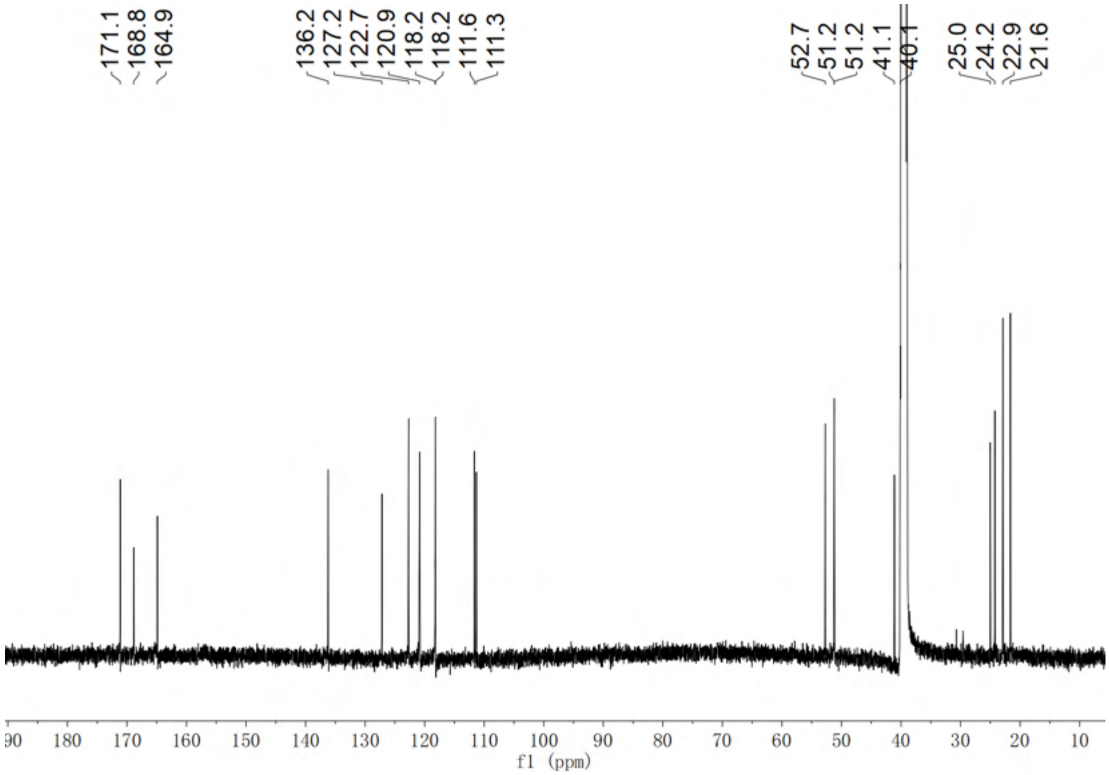

Supplementary Fig. 378. ^{13}C NMR spectrum of compound (2*S*,3*S*)-*t*-ES-Leu-b43 in DMSO-d_6

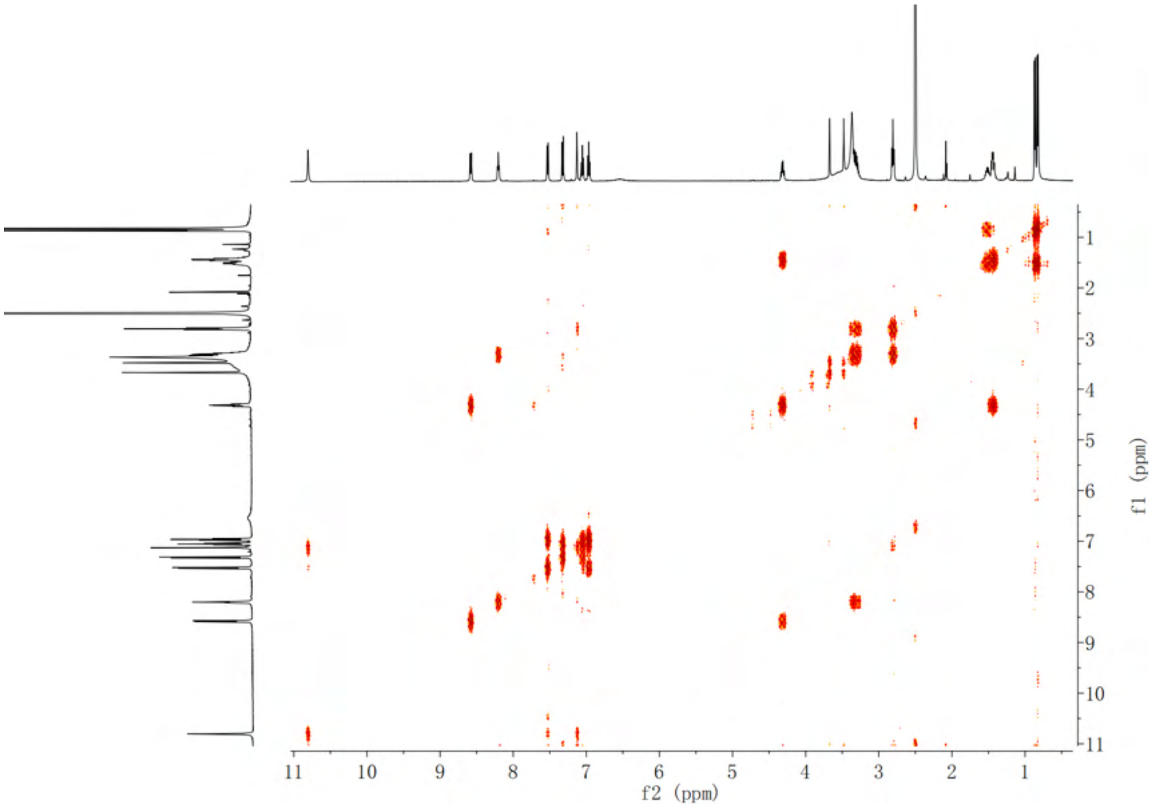

Supplementary Fig. 379. ^{1}H-^{1}H COSY spectrum of compound (2*S*,3*S*)-*t*-ES-Leu-b43 in DMSO-*d*$_6$

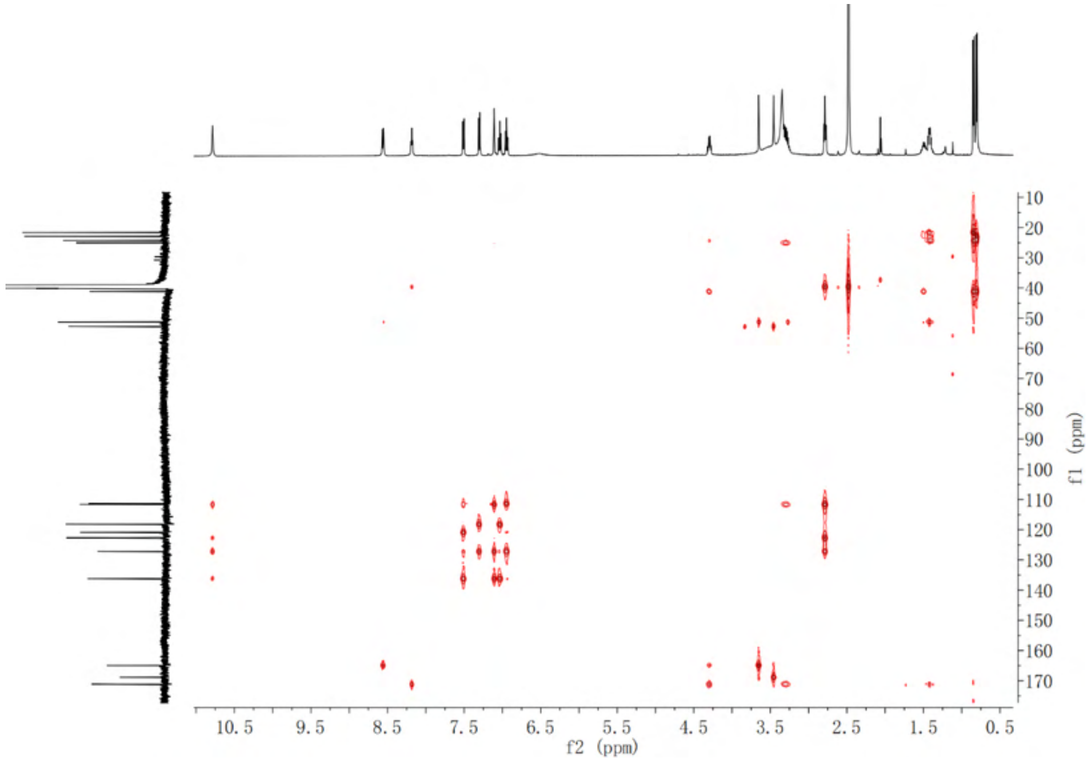

Supplementary Fig. 380. HMBC spectrum of compound (2*S*,3*S*)-*t*-ES-Leu-b43 in DMSO-*d*$_6$

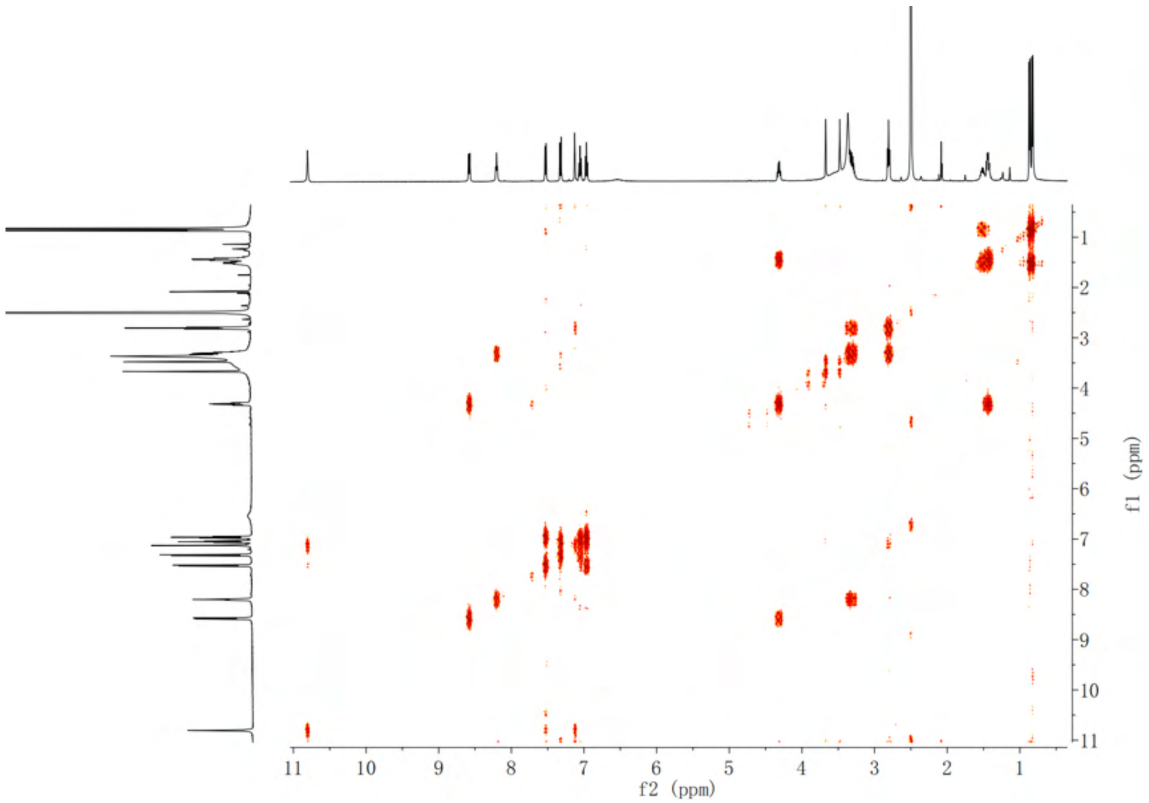

Supplementary Fig. 381. ^{1}H-^{1}H COSY spectrum of compound (2*S*,3*S*)-*t*-ES-Leu-b43 in DMSO-d_6

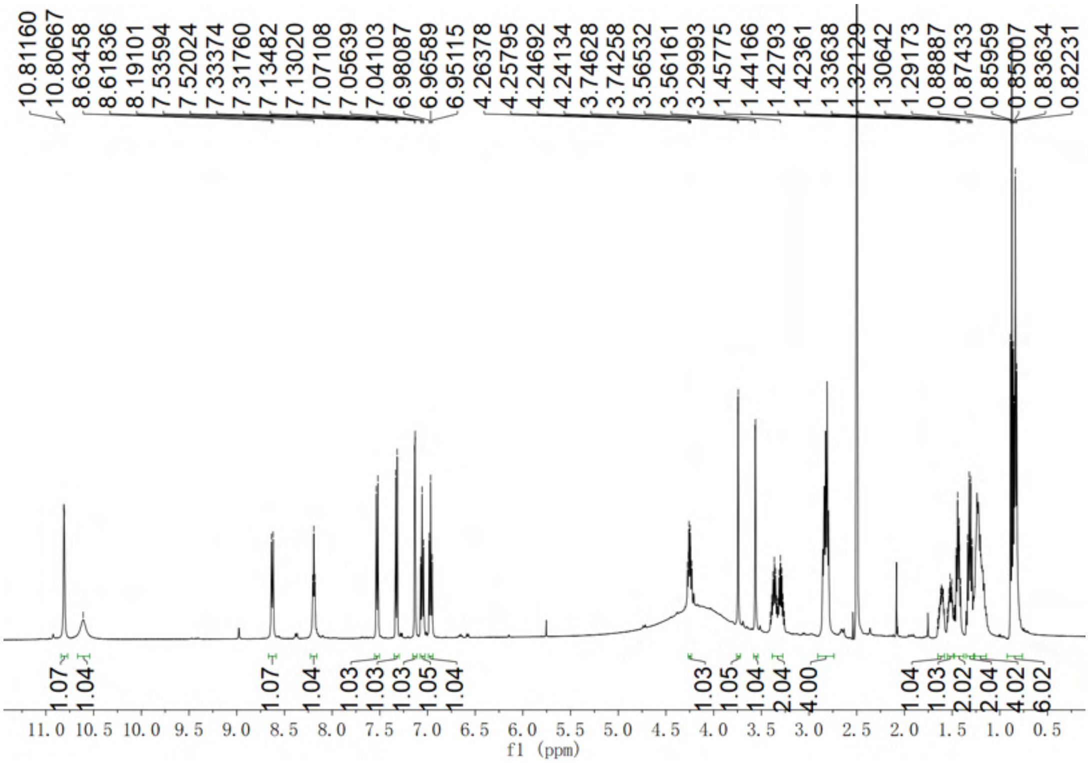

Supplementary Fig. 382. ^{1}H NMR spectrum of compound E-64c-Hydrazide in DMSO-d_6

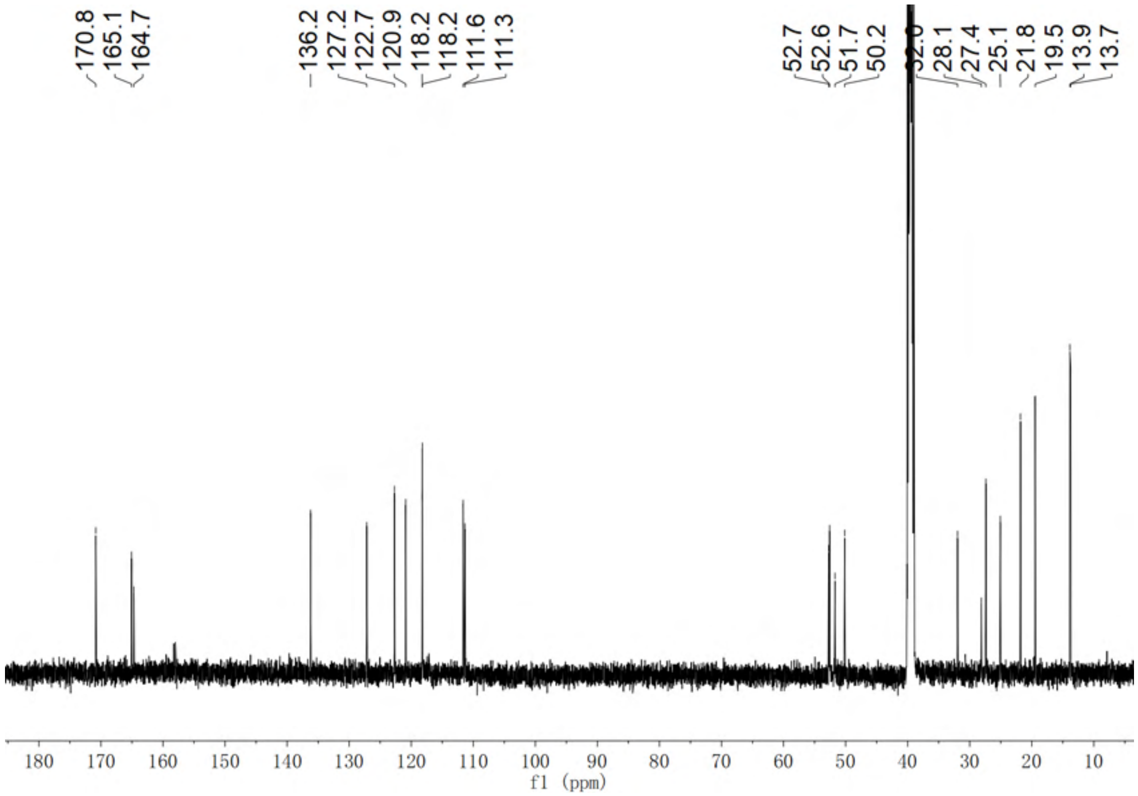

Supplementary Fig. 383. ^{13}C NMR spectrum of compound E-64c-Hydrazide in DMSO-d_6

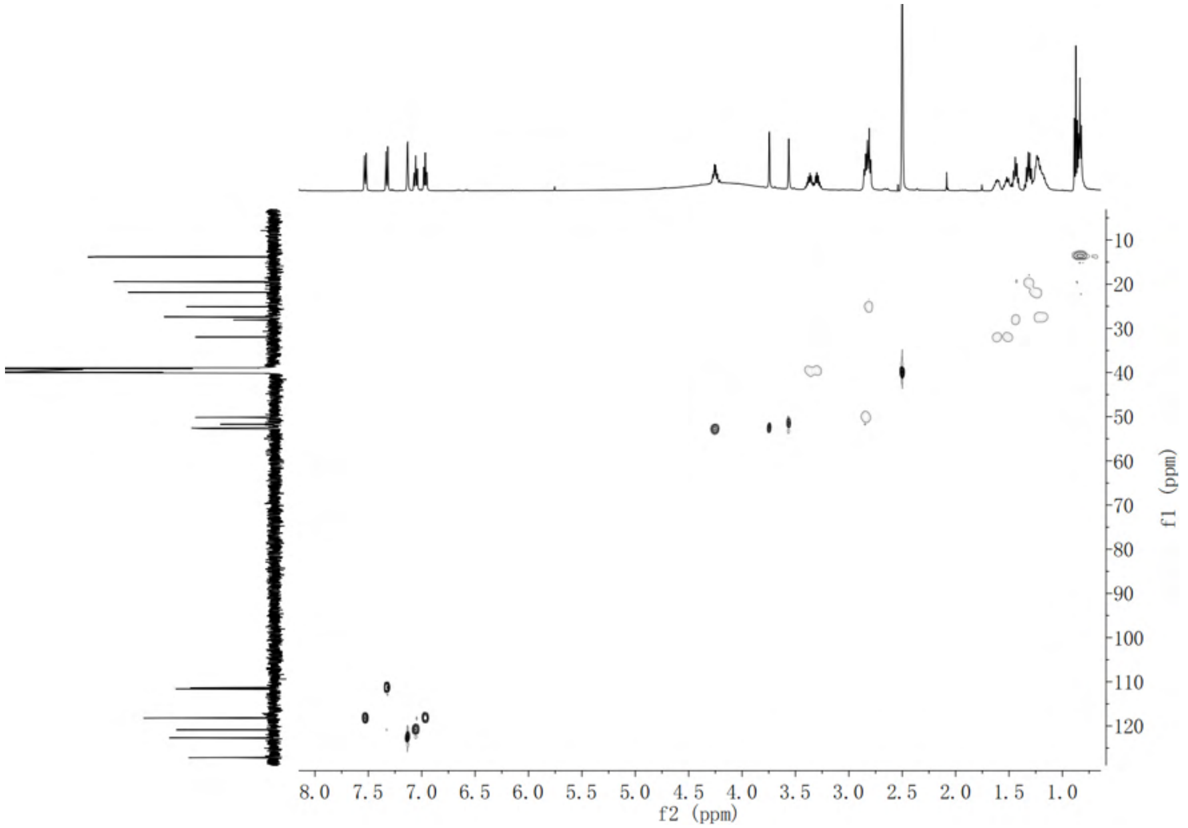

Supplementary Fig. 384. HSQC spectrum of compound E-64c-Hydrazide in DMSO-d_6

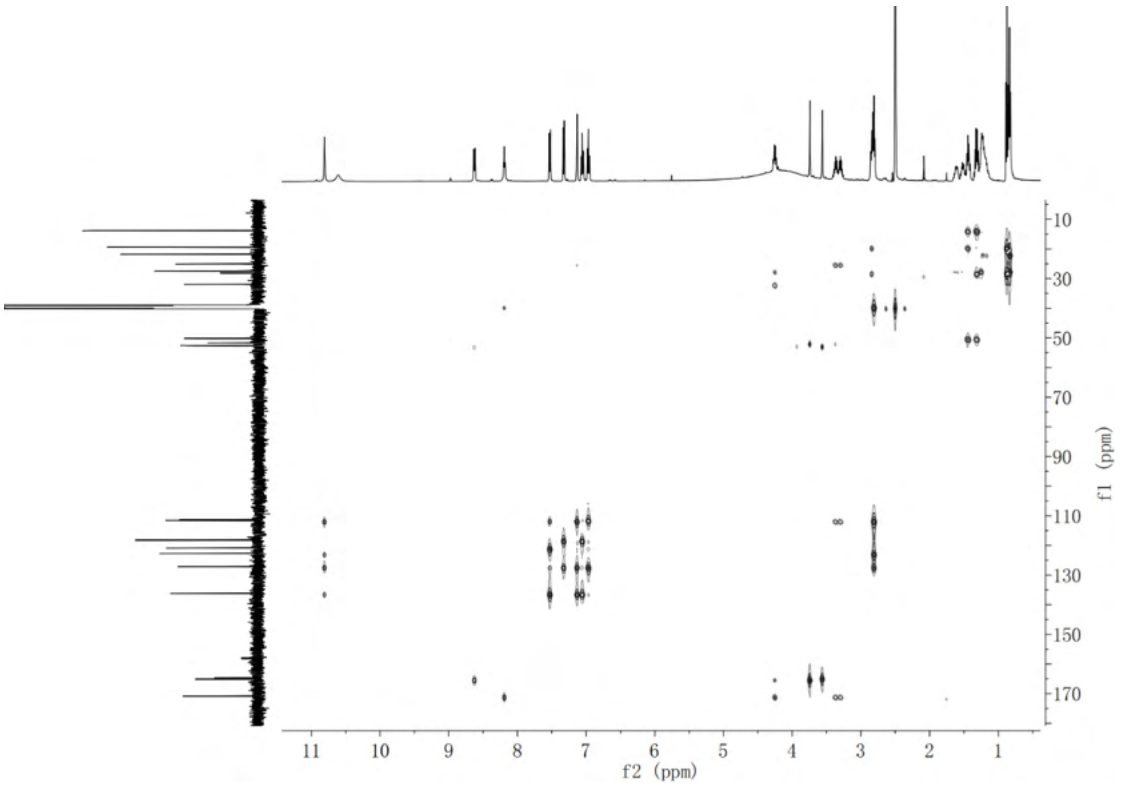

Supplementary Fig. 385. HMBC spectrum of compound E-64c-Hydrazide in DMSO-d$_6$

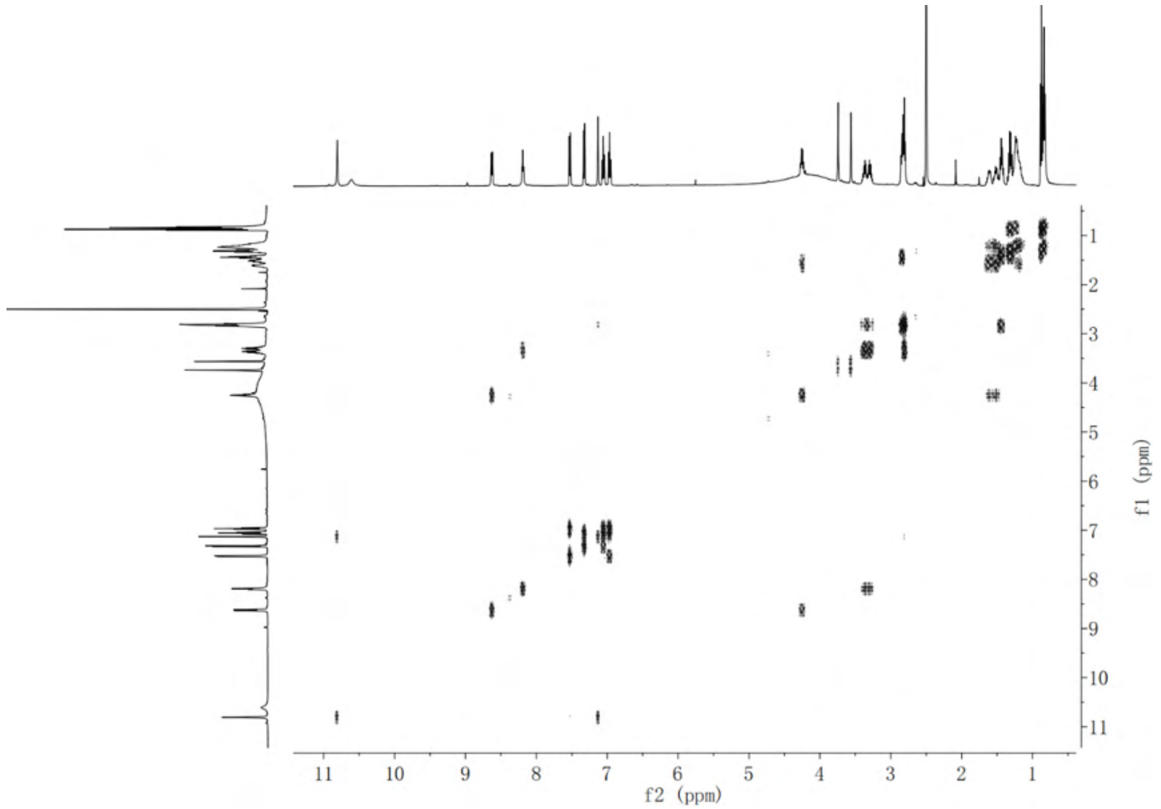

Supplementary Fig. 386. ^{1}H-^{1}H COSY spectrum of compound E-64c-Hydrazide in DMSO-d$_6$

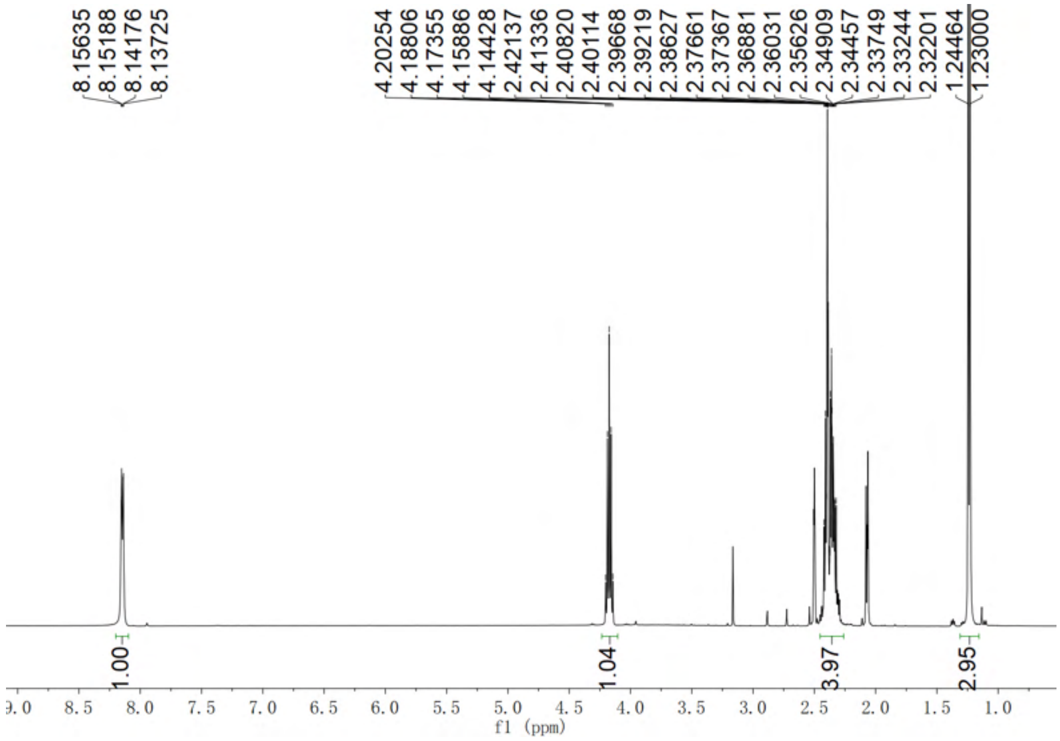

Supplementary Fig. 387. ^{1}**H NMR spectrum of compound** ***N*****-succinyl-L-alanine in DMSO-*d*$_6$**

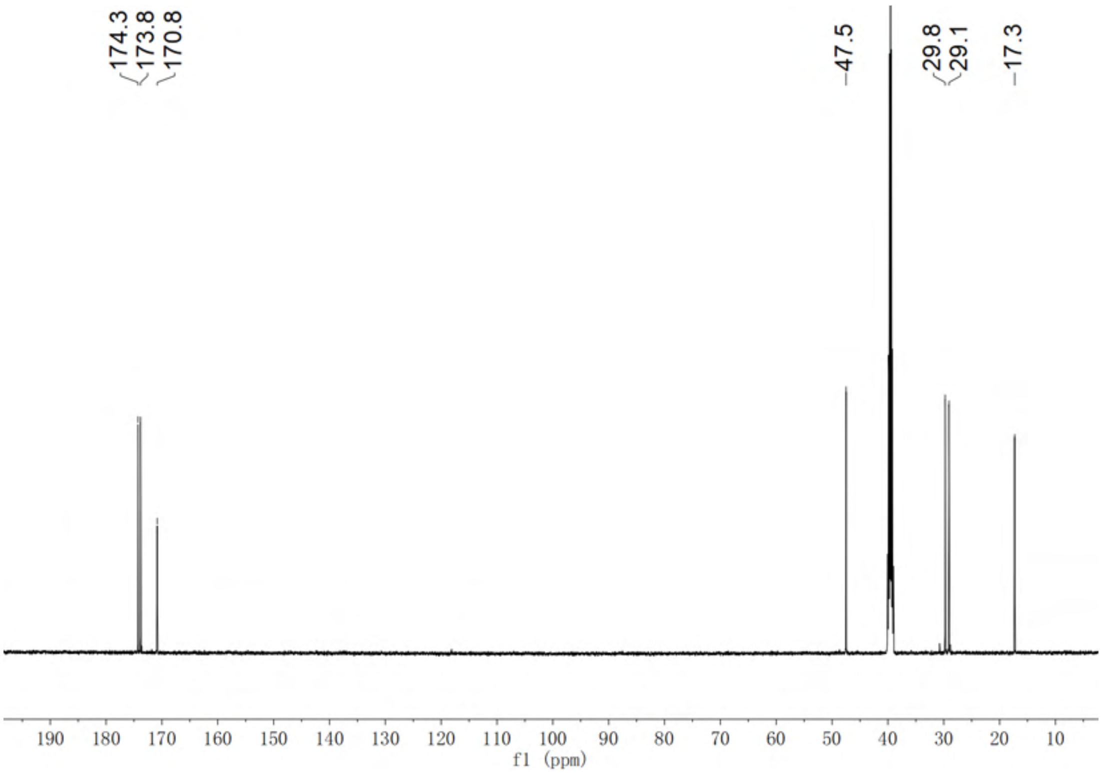

Supplementary Fig. 388. ^{13}**C NMR spectrum of compound** ***N*****-succinyl-L-alanine in DMSO-*d*$_6$**

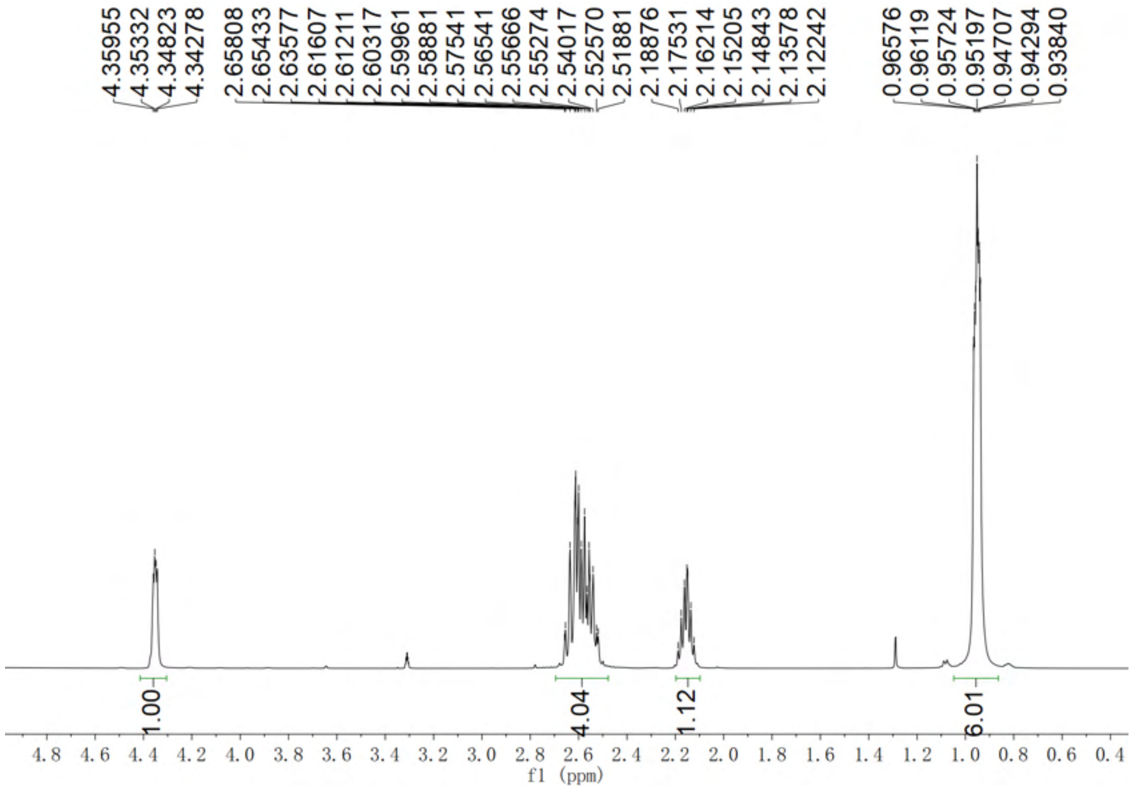

Supplementary Fig. 389. ^{1}H NMR spectrum of compound *N*-succinyl-L-valine in CD_3OD

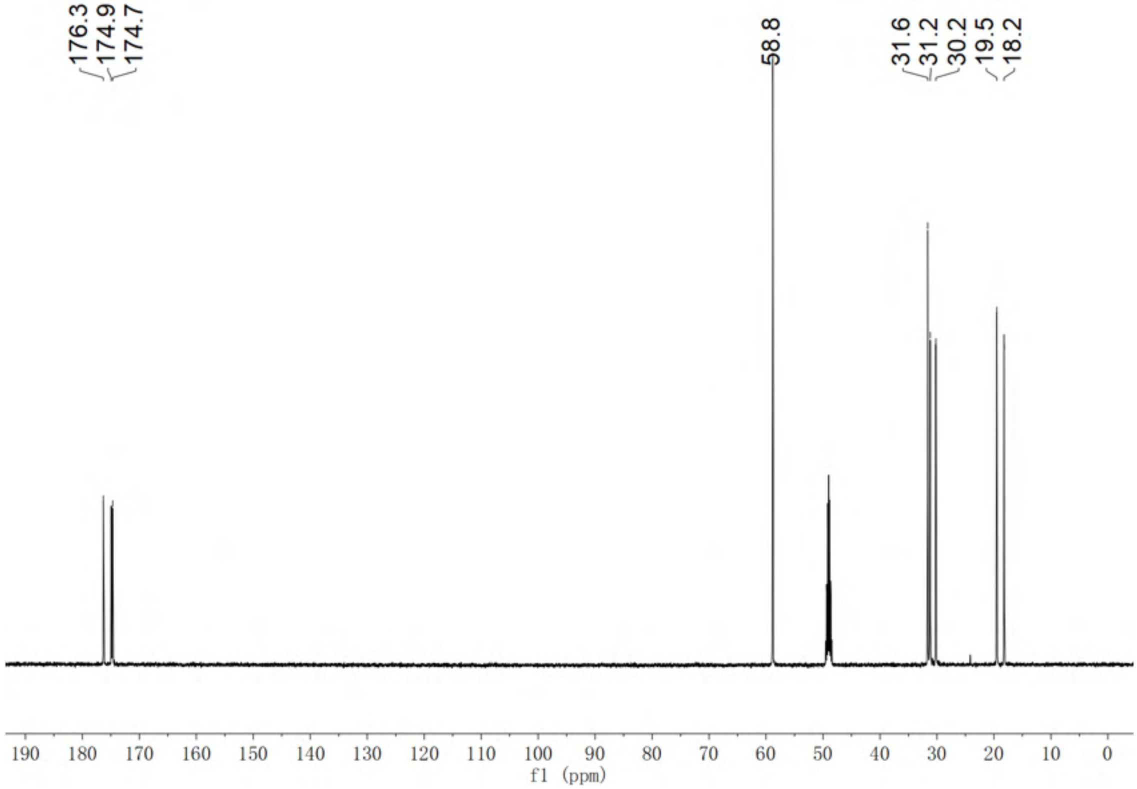

Supplementary Fig. 390. ^{13}C NMR spectrum of compound *N*-succinyl-L-valine in CD_3OD

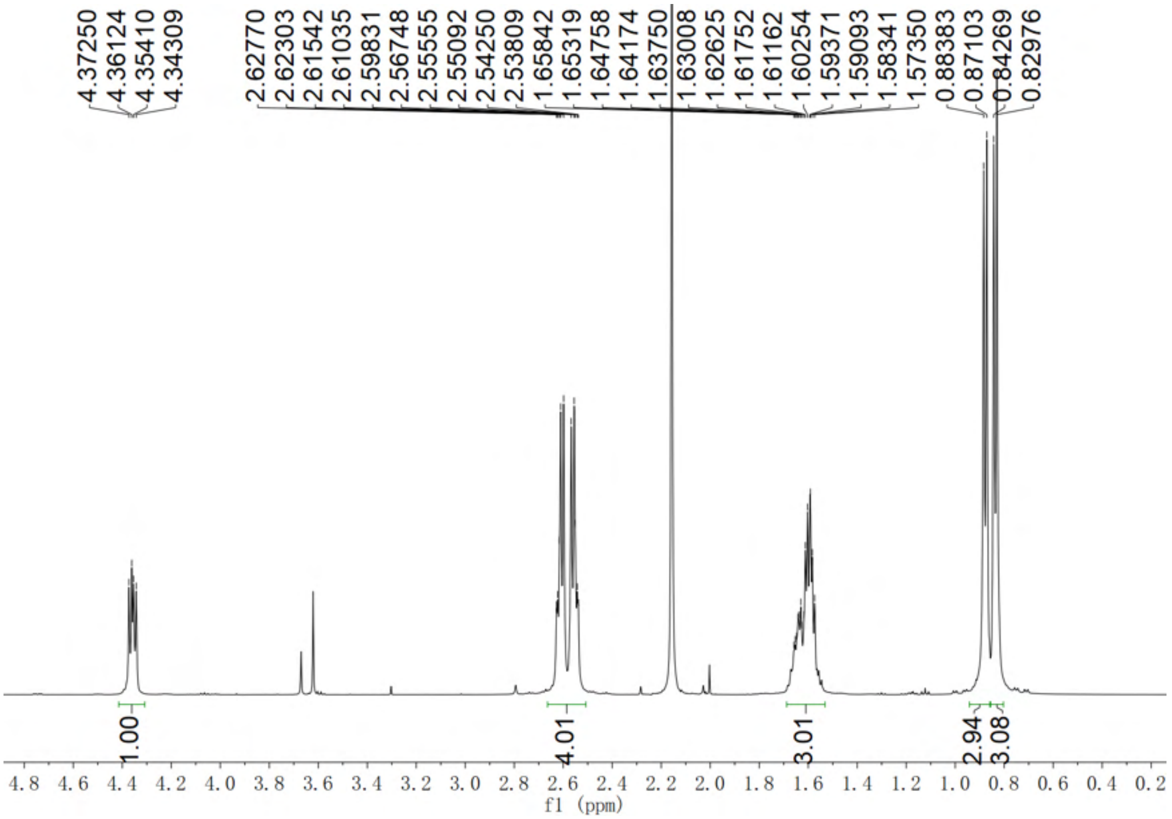

Supplementary Fig. 391. **^{1}H NMR spectrum of compound *N*-succinyl-L-leucine in D_2O**

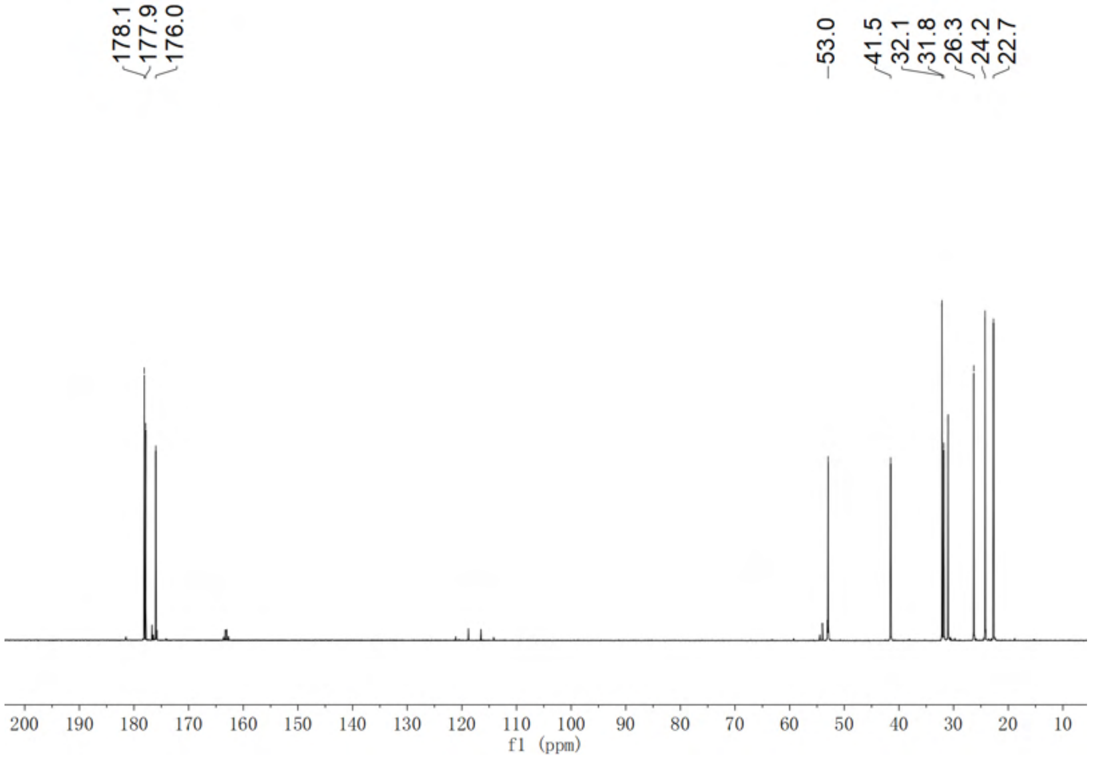

Supplementary Fig. 392. **^{13}C NMR spectrum of compound *N*-succinyl-L-leucine in D_2O**

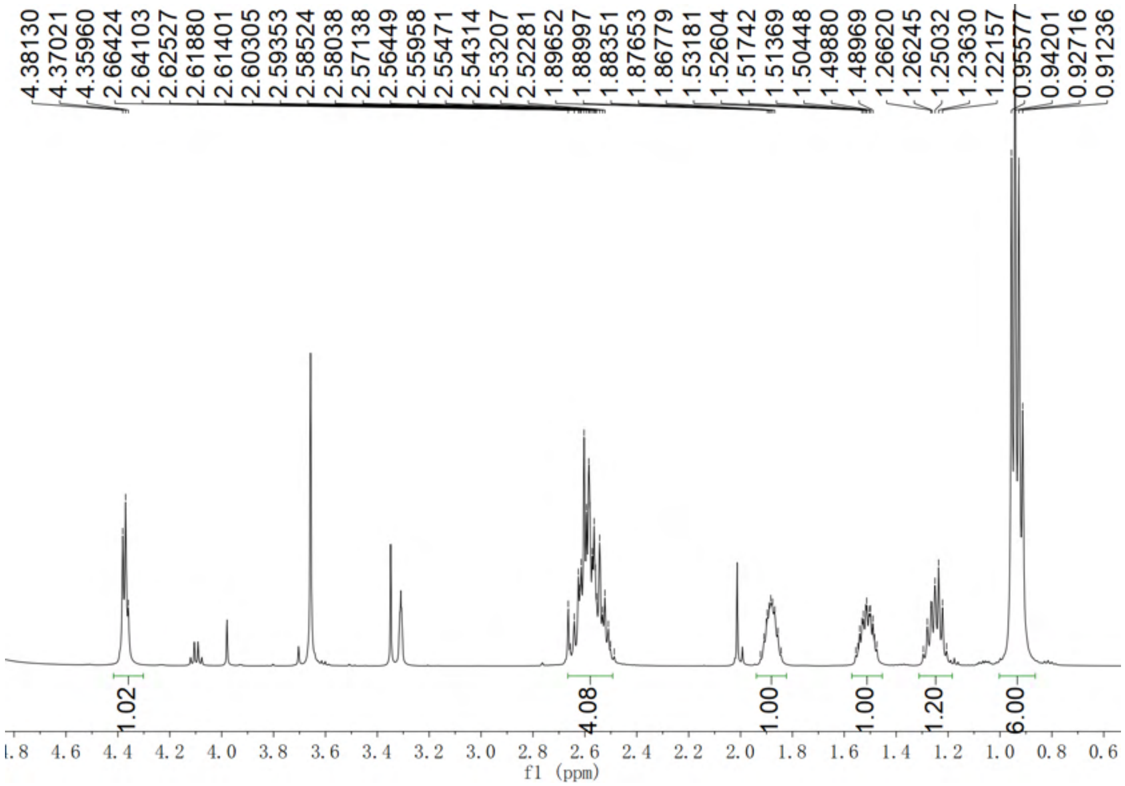

Supplementary Fig. 393. ^{1}H NMR spectrum of compound *N*-succinyl-L-isoleucine in CD_3OD

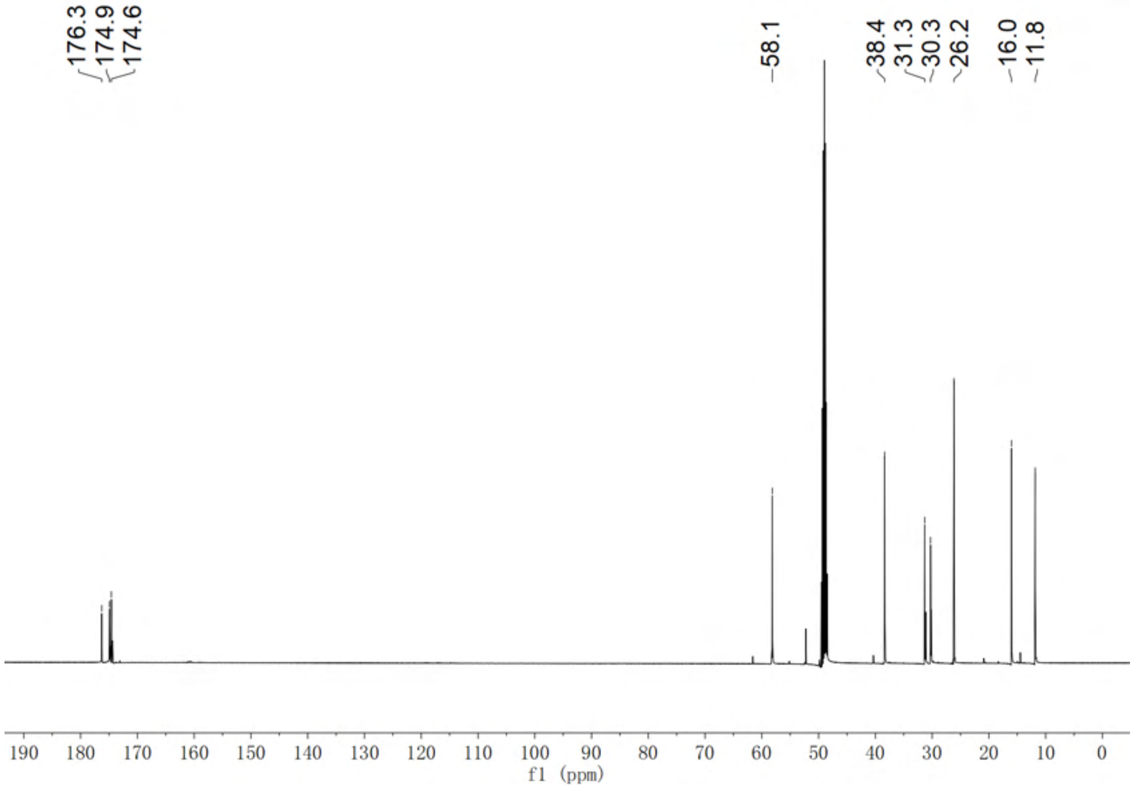

Supplementary Fig. 394. ^{13}C NMR spectrum of compound *N*-succinyl-L-isoleucine in CD_3OD

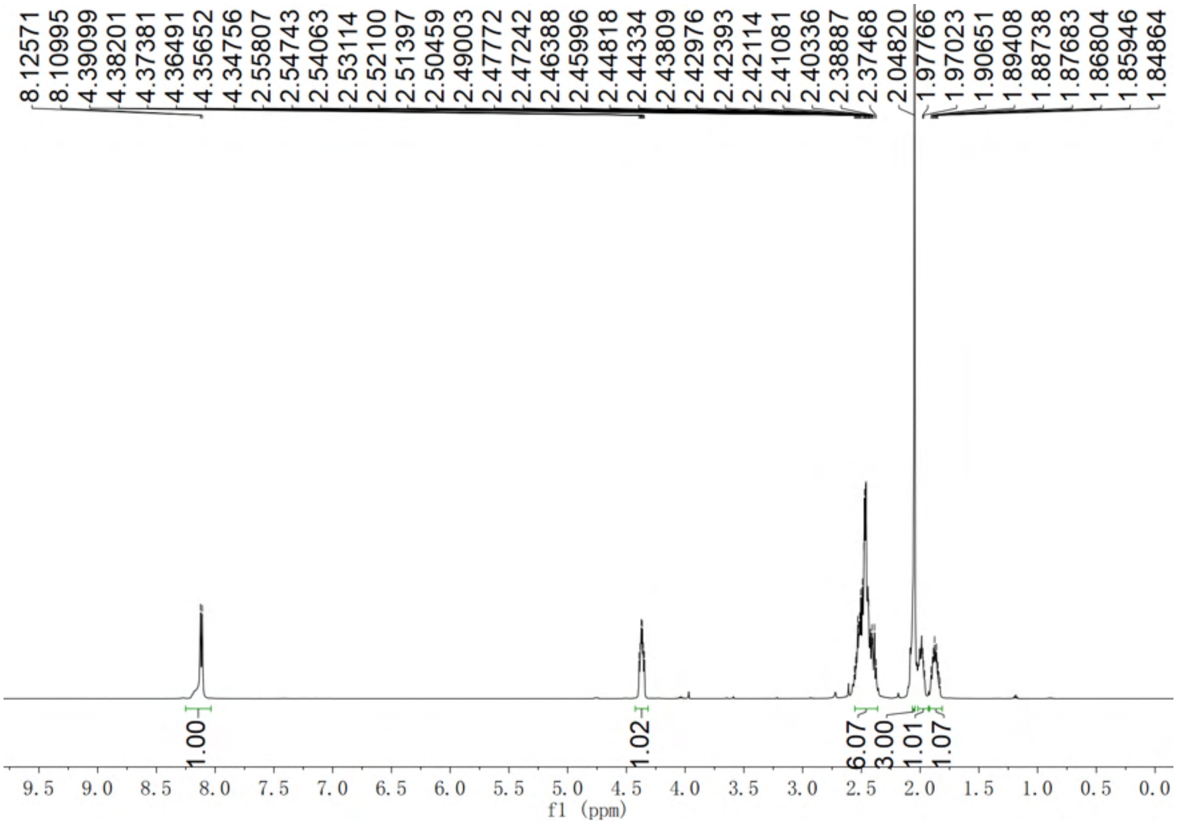

Supplementary Fig. 395. ^{1}**H NMR spectrum of compound** ***N*****-succinyl-L-methionine in DMSO-*****d*****$_6$**

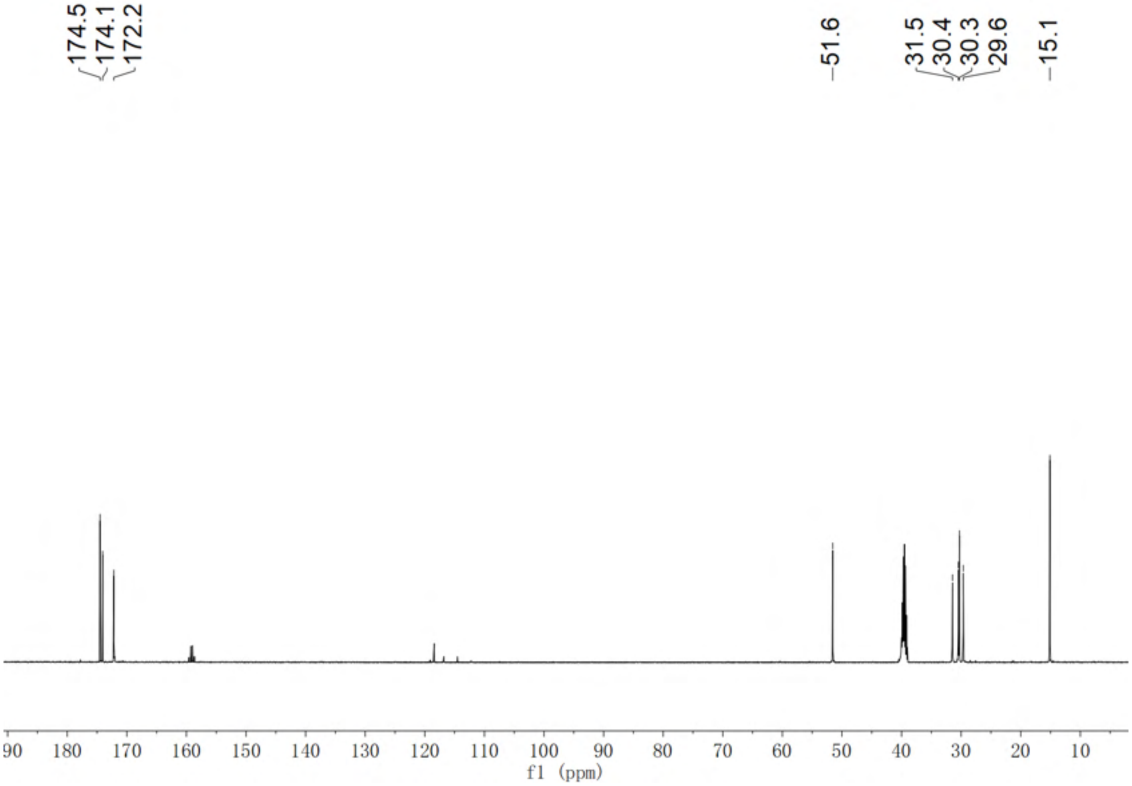

Supplementary Fig. 396. ^{13}**C NMR spectrum of compound** ***N*****-succinyl-L-methionine in DMSO-*****d*****$_6$**

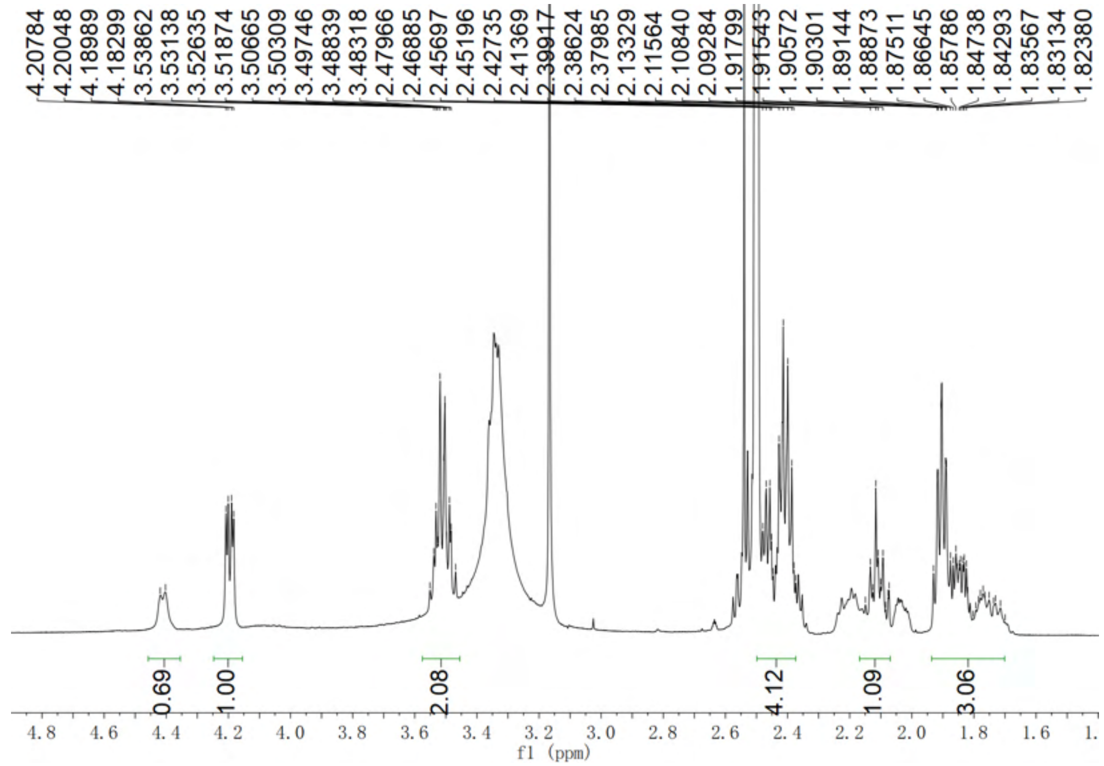

Supplementary Fig. 397. ^{1}**H NMR spectrum of compound** ***N*****-succinyl-L-proline in DMSO-*****d*****$_6$**

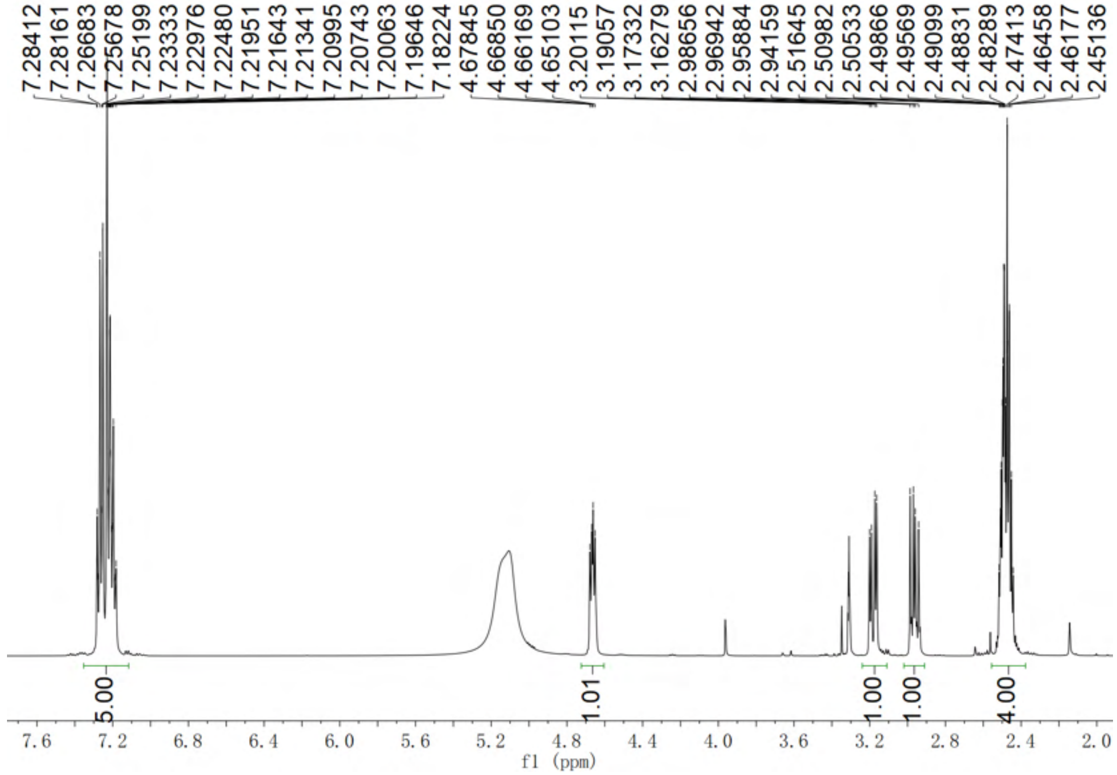

Supplementary Fig. 398. ^{1}**H NMR spectrum of compound** ***N*****-succinyl-L-phenylalanine in CD$_3$OD**

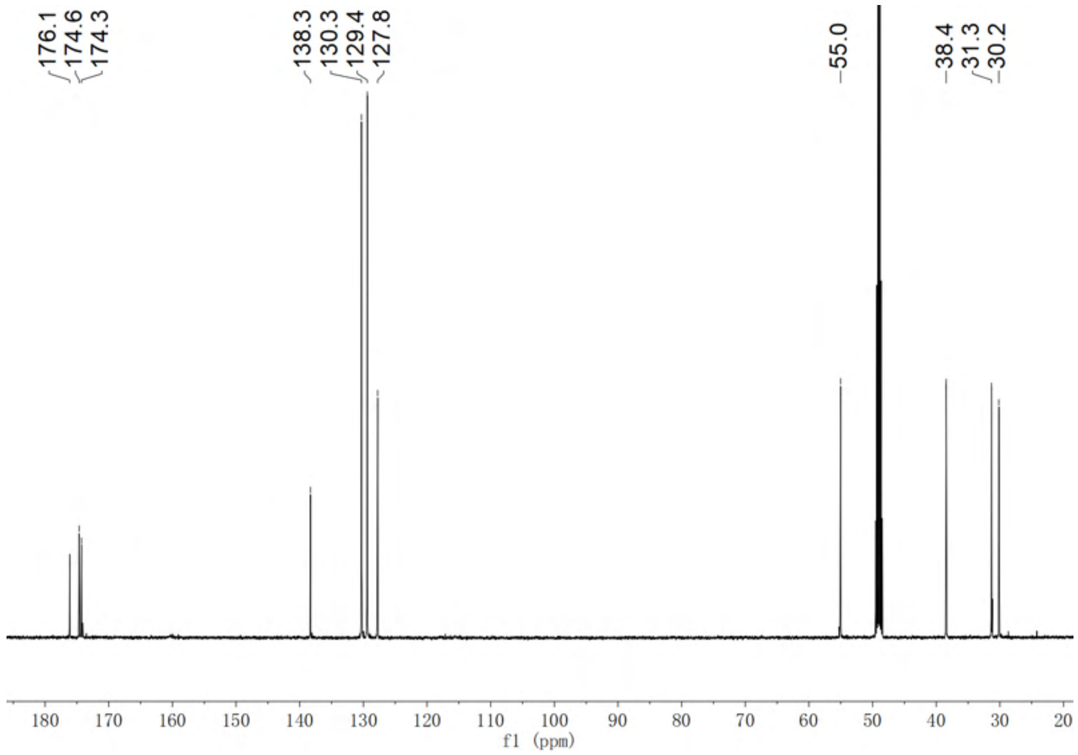

Supplementary Fig. 399. ^{13}**C NMR spectrum of compound *N*-succinyl-L-phenylalanine in CD_3OD**

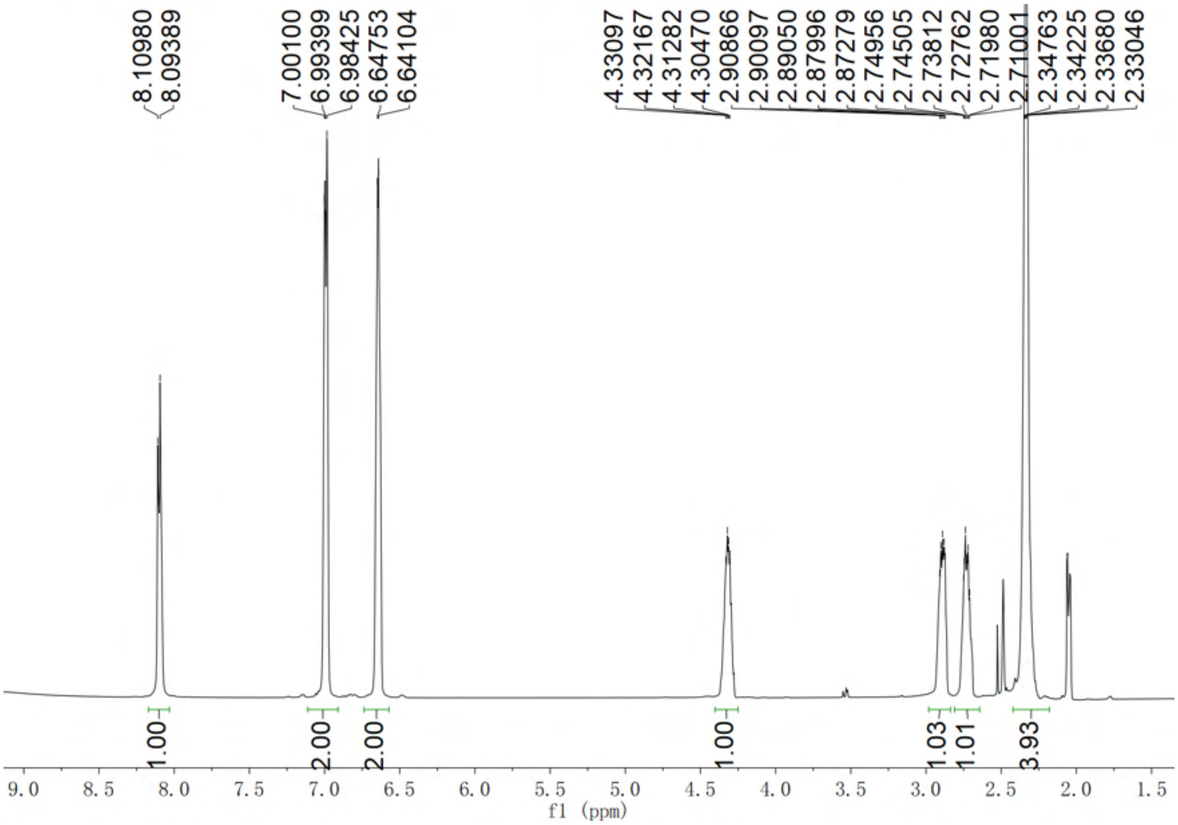

Supplementary Fig. 400. ^{1}**H NMR spectrum of compound *N*-succinyl-L-tyrosine in DMSO-d_6**

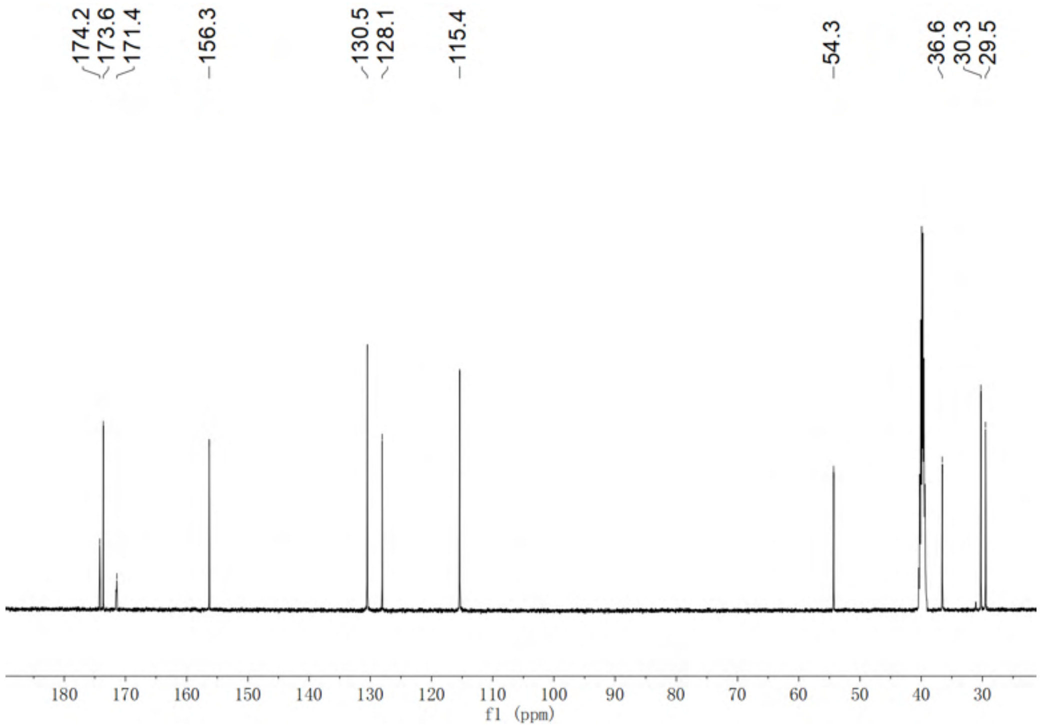

Supplementary Fig. 401. ^{13}C NMR spectrum of compound *N*-succinyl-L-tyrosine in DMSO-d_6

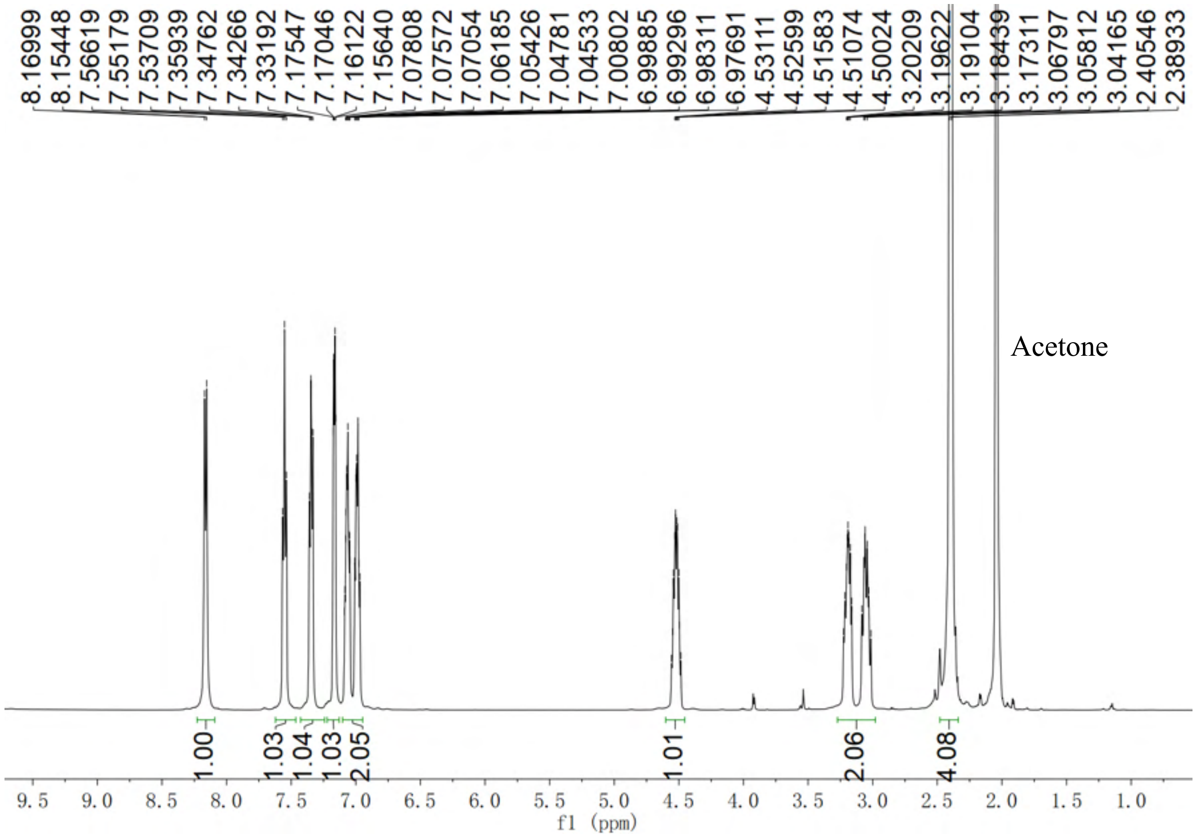

Supplementary Fig. 402. ^{1}H NMR spectrum of compound *N*-succinyl-L-tryptophan in DMSO-d_6

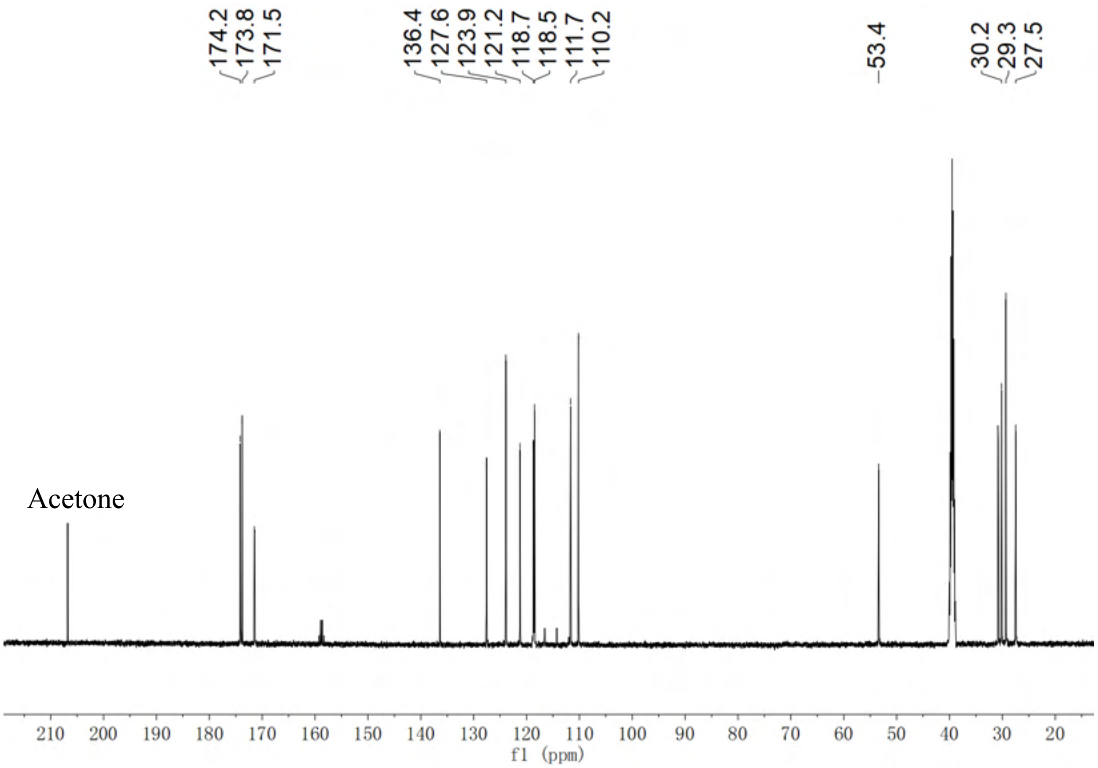

Supplementary Fig. 403. ^{13}C NMR spectrum of compound *N*-succinyl-L-tryptophan in DMSO-d_6

Supplementary References

1. Liu, N. *et al.* Identification and heterologous production of a benzoyl-primed tricarboxylic acid polyketide intermediate from the zaragozic acid A biosynthetic pathway. *Org. Lett.* **19**, 3560–3563 (2017).
2. Sumida, Y. *et al.* Identification and characterization of D-succinylase, and a proposed enzymatic method for D-amino acid synthesis. *Adv. Synth. Catal.* **358**, 2041–2046 (2016).
3. Sawada, J., Hanada, K., Tamai, M., Morimoto, S. & Omura, S. Epoxysuccinic acid derivatives. US4393228A (1983)
4. Christa, P. *et al.* Discovery and identification of tastants and taste-modulating N-acyl amino acid derivatives in traditional korean fermented dish kimchi using a sensomics approach. *J. Agric. Food Chem.* **70**, 7500–7514 (2022).
5. Mitchell, A. J. *et al.* Structure-guided reprogramming of a hydroxylase to halogenate its small molecule substrate. *Biochemistry* **56**, 441–444 (2017).
6. Sarabia, F., Sánchez-Ruiz, A. & Chammaa, S. Stereoselective synthesis of E-64 and related cysteine proteases inhibitors from 2,3-epoxyamides. *Bioorg. Med. Chem.* **13**, 1691–1705 (2005).
7. Hanada, K. *et al.* Isolation and characterization of E–64, a new thiol protease inhibitor. *Agric. Biol. Chem.* **42**, 523–528 (1978).
8. Huisman, M. *et al.* Caging the uncageable: using metal complex release for photochemical control over irreversible inhibition. *Chem. Commun.* **52**, 12590–12593 (2016).
9. Yamada, T. *et al.* Cysteine protease inhibitors produced by the industrial koji mold, Aspergillus oryzae O-1018. *Biosci. Biotechnol. Biochem.* **62**, 907–914 (1998).
10. Tromsdorf, N., Ullrich, F. T. H., Rethmeier, M., Sommerhoff, C. P. & Schaschke, N. E-64c-Hydrazide based

cathepsin C inhibitors: Optimizing the interactions with the S1'-S2' Area. *ChemMedChem* **18**, e202300218 (2023).
11. Morishita, A. *et al.* AM4299 A and B, novel thiol protease inhibitors. *J. Antibiot.* **47**, 1065–1068 (1994).
12. Woo, J.-T., Ono, H. & Tsuji, T. Cathestatins, new cysteine protease inhibitors produced by Penicillium citrinum. *Biosci. Biotechnol. Biochem.* **59**, 350–352 (1995).
13. Yaginuma, S. *et al.* Isolation and characterization of new thiol protease inhibitors estatins A and B. *J. Antibiot.* **42**, 1362–1369 (1989).
14. Otsuka, T. *et al.* WF14865A and B, New cathepsins B and L inhibitors produced by Aphanoascus fulvescens I. taxonomy, production, purification and biological properties. *J. Antibiot.* **53**, 449–458 (2000).
15. Isshiki, K. *et al.* TMC-52A to D, novel cysteine proteinase inhibitors, produced by Gliocladium sp. *J. Antibiot.* **51**, 629–634 (1998).
16. Shin, H. J., Matsuda, H., Murakami, M. & Yamaguchi, K. Circinamide, a novel papain inhibitor from the cyanobacterium *Anabaena circinalis* (NIES-41). *Tetrahedron* **53**, 5747–5754 (1997).
17. Nicolau, I., Hădade, N. D., Matache, M. & Funeriu, D. P. Synthetic approaches of epoxysuccinate chemical probes. *ChemBioChem* **24**, e202300157 (2023).
18. Steinchen, W. *et al.* Bimodular peptide synthetase SidE produces fumarylalanine in the human pathogen Aspergillus fumigatus. *Appl. Environ. Microbiol.* **79**, 6670–6676 (2013).
19. Kalb, D. *et al.* Genetic engineering activates biosynthesis of aromatic fumaric acid amides in the human pathogen Aspergillus fumigatus. *Appl. Environ. Microbiol.* **81**, 1594–1600 (2015).
20. Heard, S. C., Diehl, K. L. & Winter, J. M. Biosynthesis of the fungal nonribosomal peptide penilumamide A and biochemical characterization of a pterin-specific adenylation domain. *RSC Chem. Biol.* **4**, 748–753 (2023).
21. Hollenhorst, M. A., Clardy, J. & Walsh, C. T. The ATP-dependent amide ligases DdaG and DdaF assemble the fumaramoyl-dipeptide scaffold of the dapdiamide antibiotics. *Biochemistry* **48**, 10467–10472 (2009).
22. Hollenhorst, M. A. *et al.* The nonribosomal peptide synthetase enzyme DdaD tethers Nβ-fumaramoyl-l-2,3-diaminopropionate for Fe(II)/α-ketoglutarate-dependent epoxidation by DdaC during dapdiamide antibiotic biosynthesis. *J. Am. Chem. Soc.* **132**, 15773–15781 (2010).
23. Wolf, F. *et al.* Biosynthesis of the β-lactone proteasome inhibitors belactosin and cystargolide. *Angew. Chem. Int. Ed.* **56**, 6665–6668 (2017).
24. Xu, G. *et al.* Cryptic enzymatic assembly of peptides armed with β-lactone warheads. *Nat. Chem. Biol.* **20**, 1371–1379 (2024).
25. Abramson, J. *et al.* Accurate structure prediction of biomolecular interactions with AlphaFold 3. *Nature* **630**, 493–500 (2024).
26. Zahn, M. *et al.* Structures of 2-hydroxyisobutyric acid-CoA ligase reveal determinants of substrate specificity and describe a multi-conformational catalytic cycle. *J. Mol. Biol.* **431**, 2747–2761 (2019).
27. van Kempen, M. *et al.* Fast and accurate protein structure search with Foldseek. *Nat. Biotechnol.* **42**, 243–246 (2024).
28. Galant, A., Arkus, K. A. J., Zubieta, C., Cahoon, R. E. & Jez, J. M. Structural basis for evolution of product diversity in soybean glutathione biosynthesis. *Plant Cell* **21**, 3450–3458 (2009).
29. Tamai, M. *et al.* Efficient synthetic method for ethyl (+) - (2S, 3S) -3- [(S) -3-methyl-1- (3-methylbutylcarbamoyl) butylcarbamoyl] -2-oxiranecarboxylate (EST), a new inhibitor of cysteine proteinases. *Chem. Pharm. Bull.* **35**, 1098–1104 (1987).
30. Detterbeck, R. & Hesse, M. Konfiguration und enantioselektive Synthese des Pilzmetaboliten WF14861. *Helv.*

Chim. Acta **86**, 222–232 (2003).
31. Chehade, K. A. H., Baruch, A., Verhelst, S. H. L. & Bogyo, M. An improved preparation of the activity-based probe JPM-OEt and in situ applications. *Synthesis* **2005**, 240–244 (2004).
32. Trott, O. & Olson, A. J. AutoDock Vina: Improving the speed and accuracy of docking with a new scoring function, efficient optimization, and multithreading. *J. Comput. Chem.* **31**, 455–461 (2010).
33. Gerlt, J. A. *et al.* Enzyme Function Initiative-Enzyme Similarity Tool (EFI-EST): A web tool for generating protein sequence similarity networks. *Biochim. Biophys. Acta, Proteins Proteomics* **1854**, 1019–1037 (2015).
34. Hanada, K. *et al.* Structure and synthesis of E–64, a new thiol protease inhibitor. *Agric. Biol. Chem.* **42**, 529–536 (1978).
35. Lubberink, M. *et al.* Biocatalytic monoacylation of symmetrical diamines and its application to the synthesis of pharmaceutically relevant amides. *ACS Catal.* **10**, 10005–10009 (2020).

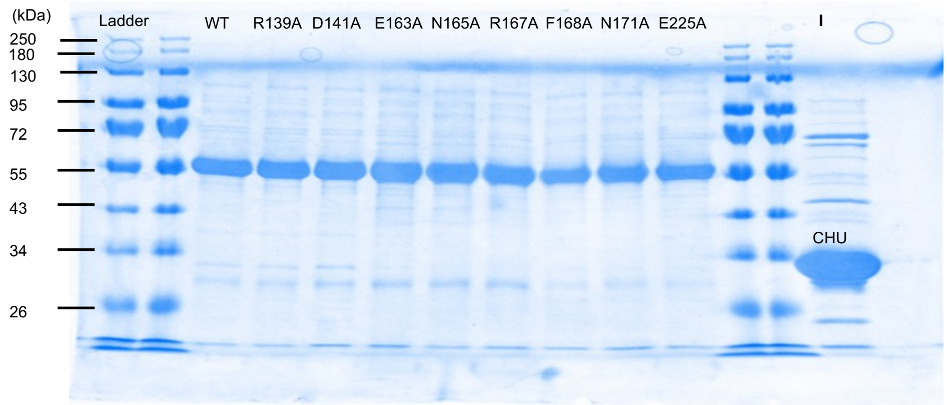

Uncropped SDS−PAGE used in Supplementary Fig. 15. **I**: the lane is used in Supplementary Fig. 7 .

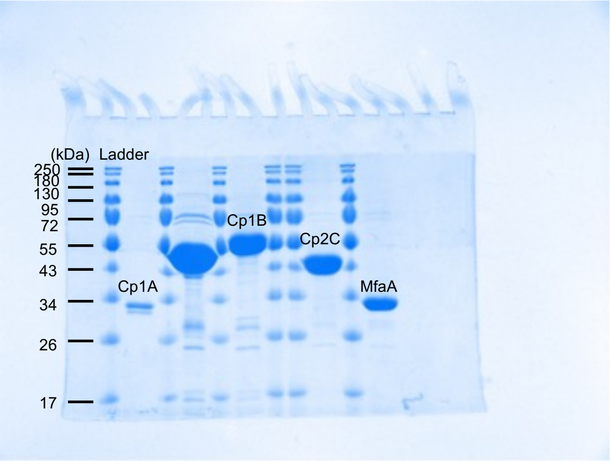

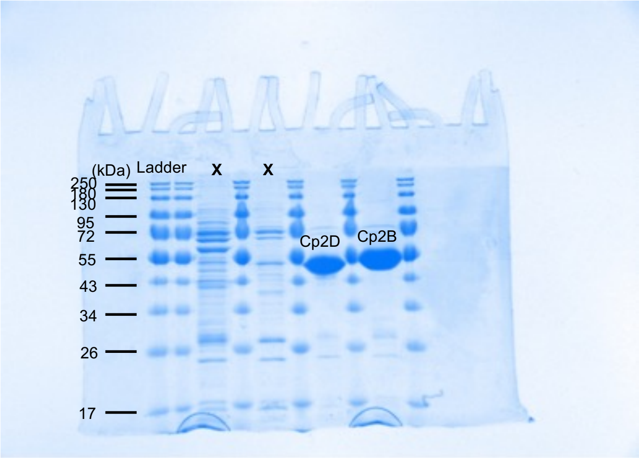

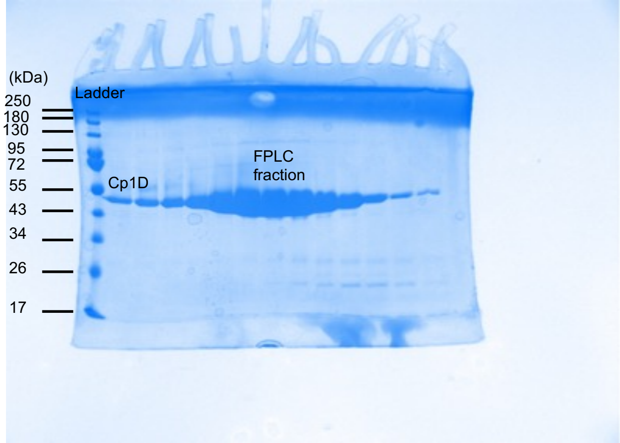

Uncropped SDS−PAGE used in Supplementary Fig. 7.
X: the lane is irrelevant sample.

Uncropped SDS−PAGE.
