# Supplementary material for: Enzymatic combinatorial synthesis of E-64 and related cysteine protease inhibitors
Source: Nat Chem Biol. 2025 May 9;21(11):1783–93. doi: 10.1038/s41589-025-01907-2 (PMC12568646; doi:10.1038/s41589-025-01907-2)
Supplement: Supplementary file 2 — Reporting Summary [file 41589_2025_1907_MOESM2_ESM.pdf]

nature portfolio

Corresponding author(s): Masao Ohashi, Yi Tang

Last updated by author(s): Feb 20, 2025

Reporting Summary

Nature Portfolio wishes to improve the reproducibility of the work that we publish. This form provides structure for consistency and transparency in reporting. For further information on Nature Portfolio policies, see our Editorial Policies and the Editorial Policy Checklist.

Statistics

For all statistical analyses, confirm that the following items are present in the figure legend, table legend, main text, or Methods section.

n/a	Confirmed	
☐	☒	The exact sample size (*n*) for each experimental group/condition, given as a discrete number and unit of measurement
☐	☒	A statement on whether measurements were taken from distinct samples or whether the same sample was measured repeatedly
☒	☐	The statistical test(s) used AND whether they are one- or two-sided<br>*Only common tests should be described solely by name; describe more complex techniques in the Methods section.*
☒	☐	A description of all covariates tested
☒	☐	A description of any assumptions or corrections, such as tests of normality and adjustment for multiple comparisons
☐	☒	A full description of the statistical parameters including central tendency (e.g. means) or other basic estimates (e.g. regression coefficient) AND variation (e.g. standard deviation) or associated estimates of uncertainty (e.g. confidence intervals)
☒	☐	For null hypothesis testing, the test statistic (e.g. *F*, *t*, *r*) with confidence intervals, effect sizes, degrees of freedom and *P* value noted<br>*Give P values as exact values whenever suitable.*
☒	☐	For Bayesian analysis, information on the choice of priors and Markov chain Monte Carlo settings
☒	☐	For hierarchical and complex designs, identification of the appropriate level for tests and full reporting of outcomes
☒	☐	Estimates of effect sizes (e.g. Cohen's *d*, Pearson's *r*), indicating how they were calculated

Our web collection on statistics for biologists contains articles on many of the points above.

Software and code

Policy information about availability of computer code

Data collection	MassHunter Workstation 10.0 by Agilent was used to collect mass spectrometry data from QTOF LC-MS. OpenLab CDS 2.4 by Agilent was used to collect UV and mass spectrometry data from LC-MS. TopSpin 3.5pl4 by Bruker was used to collect NMR data. The crystal structure data of Cp1B were collected at beamline 17-ID-2 at the National Synchrotron Light Source II.
Data analysis	MestReNova-9.0.1 (NMR), MassHunter Qualitative Analysis Software 10.0 (QTOF LC-MS), OpenLab CDS (LC-MS), ChemDraw v20.0 (chemical structures and exact MS calculations), Pymol v3.0.3 (protein structure), AutoDock Vina v1.5.7 (molecular docking), GraphPad Prism 9.0.0, Foldseek, AlphaFold3, XDS package (build 20230630), PHENIX3, COOT5.

For manuscripts utilizing custom algorithms or software that are central to the research but not yet described in published literature, software must be made available to editors and reviewers. We strongly encourage code deposition in a community repository (e.g. GitHub). See the Nature Portfolio guidelines for submitting code & software for further information.

Data

Policy information about availability of data

All manuscripts must include a data availability statement. This statement should provide the following information, where applicable:

- Accession codes, unique identifiers, or web links for publicly available datasets
- A description of any restrictions on data availability
- For clinical datasets or third party data, please ensure that the statement adheres to our policy

The data that support the findings of this study are available within the paper and its Supplementary Information, or are available from the corresponding authors upon request. The atomic coordinates of Cp1B with adenosine and MES, apo-papain, papain with 1, papain with E-64c, papain with E-64d, and papain with (2S,3S)-t-ES-a9-b7 have been deposited in the Protein Data Bank (http://www.rcsb.org) under the accession code 9CJN, 9CLH, 9CKT, 9EG7, 9CKW, and 9CKY, respectively. The predicted structure of Cp1B (version 1), generated by AlphaFold, is available at DOI: 10.5281/zenodo.14911266.

Human research participants

Policy information about studies involving human research participants and Sex and Gender in Research.

Reporting on sex and gender	N/A
Population characteristics	N/A
Recruitment	N/A
Ethics oversight	N/A

Note that full information on the approval of the study protocol must also be provided in the manuscript.

Field-specific reporting

Please select the one below that is the best fit for your research. If you are not sure, read the appropriate sections before making your selection.

☒ Life sciences ☐ Behavioural & social sciences ☐ Ecological, evolutionary & environmental sciences

For a reference copy of the document with all sections, see nature.com/documents/nr-reporting-summary-flat.pdf

Life sciences study design

All studies must disclose on these points even when the disclosure is negative.

Sample size	No sample size calculation was conducted. For all in vitro assays, at least two independent experiments were carried out. Quantitative assays shown as a bar graph as well as kinetic assays for determination of IC50 for each compound toward cathepsin B were performed in three independent replicates as a standard practice.
Data exclusions	No data was excluded
Replication	For in vivo A. nidulans expression, experiments were repeated three times with similar results. For all in vitro enzymatic assays, multiple preparations of purified protein were performed and the assays were repeated at least two times. Quantitative enzymatic assays were performed in three independent replicates. For determination of IC50 for each compound toward cathepsin B, the assays were performed in three independent replicates. All attempts at replication were successful.
Randomization	This is not relevant because the study does not include different experimental groups.
Blinding	Blinding was not relevant as there were no clinical trials or group allocation.

Reporting for specific materials, systems and methods

We require information from authors about some types of materials, experimental systems and methods used in many studies. Here, indicate whether each material, system or method listed is relevant to your study. If you are not sure if a list item applies to your research, read the appropriate section before selecting a response.

Materials & experimental systems

n/a	Involved in the study
☒	☐ Antibodies
☒	☐ Eukaryotic cell lines
☒	☐ Palaeontology and archaeology
☒	☐ Animals and other organisms
☒	☐ Clinical data
☒	☐ Dual use research of concern

Methods

n/a	Involved in the study
☒	☐ ChIP-seq
☒	☐ Flow cytometry
☒	☐ MRI-based neuroimaging
